# Supplementary figures and images for: An automated information extraction system from the knowledge graph based annual financial reports
Source: PeerJ Comput Sci. 2024 May 13;10:e2004. doi: 10.7717/peerj-cs.2004 (PMC11157543; doi:10.7717/peerj-cs.2004)

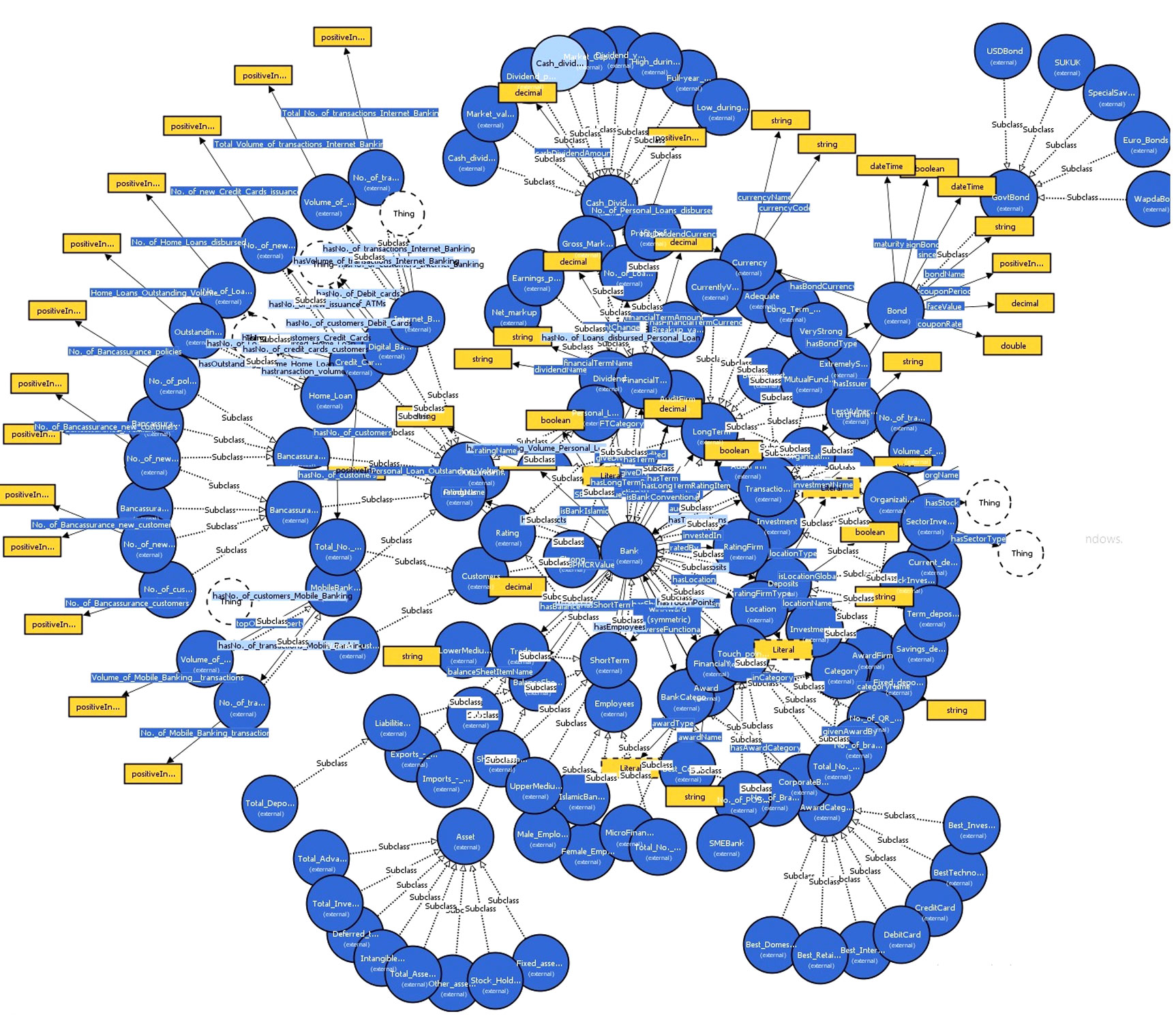

Supplement: Supplemental Information 1 [file peerj-cs-10-2004-s001.jpg]
