# Supplementary material for: An automated information extraction system from the knowledge graph based annual financial reports
Source: PeerJ Comput Sci. 2024 May 13;10:e2004. doi: 10.7717/peerj-cs.2004 (PMC11157543; doi:10.7717/peerj-cs.2004)
Supplement: Supplemental Information 3 [file peerj-cs-10-2004-s003.pdf]

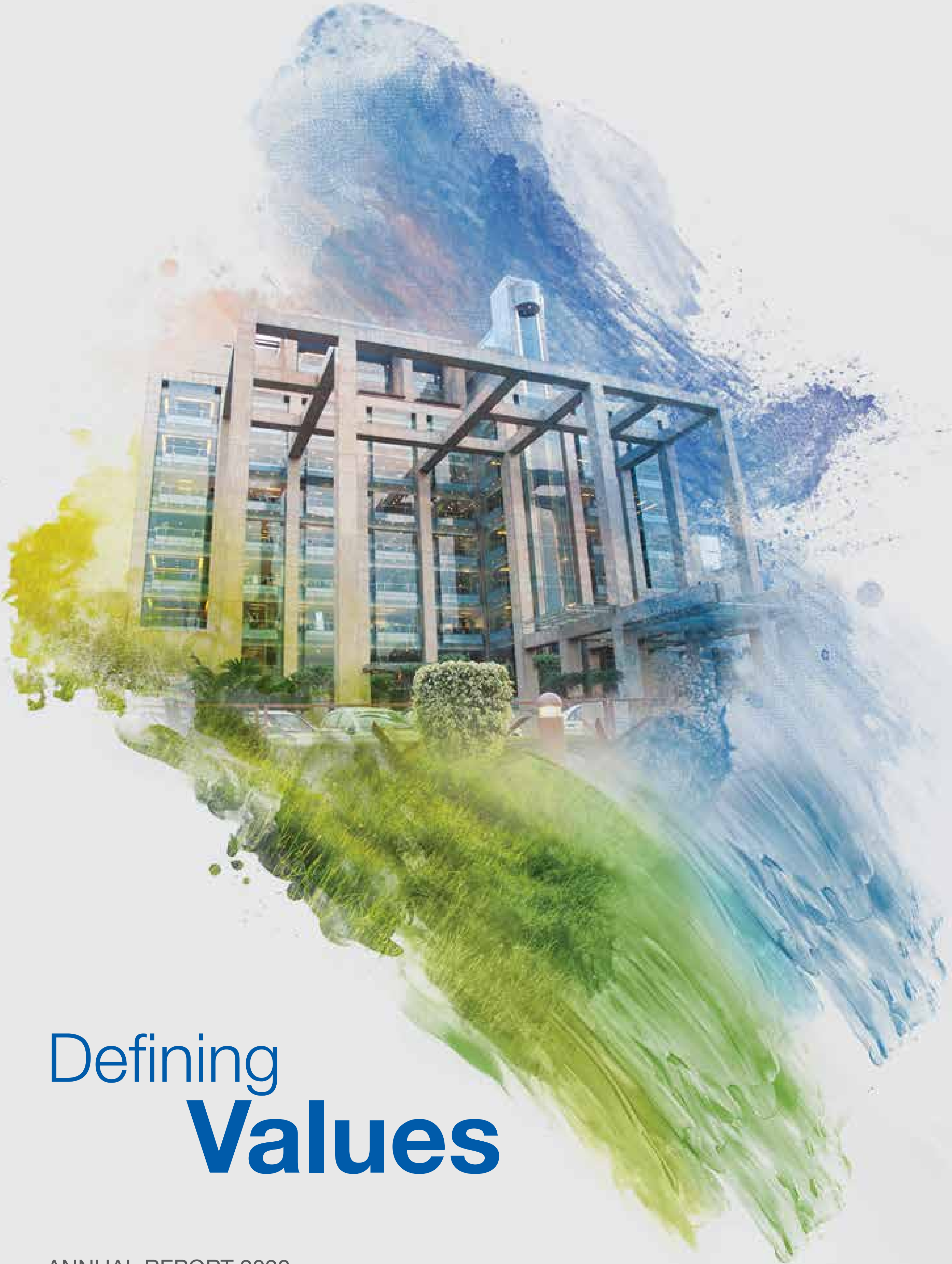

Defining Values

ANNUAL REPORT 2020

MCB

Bank for Life

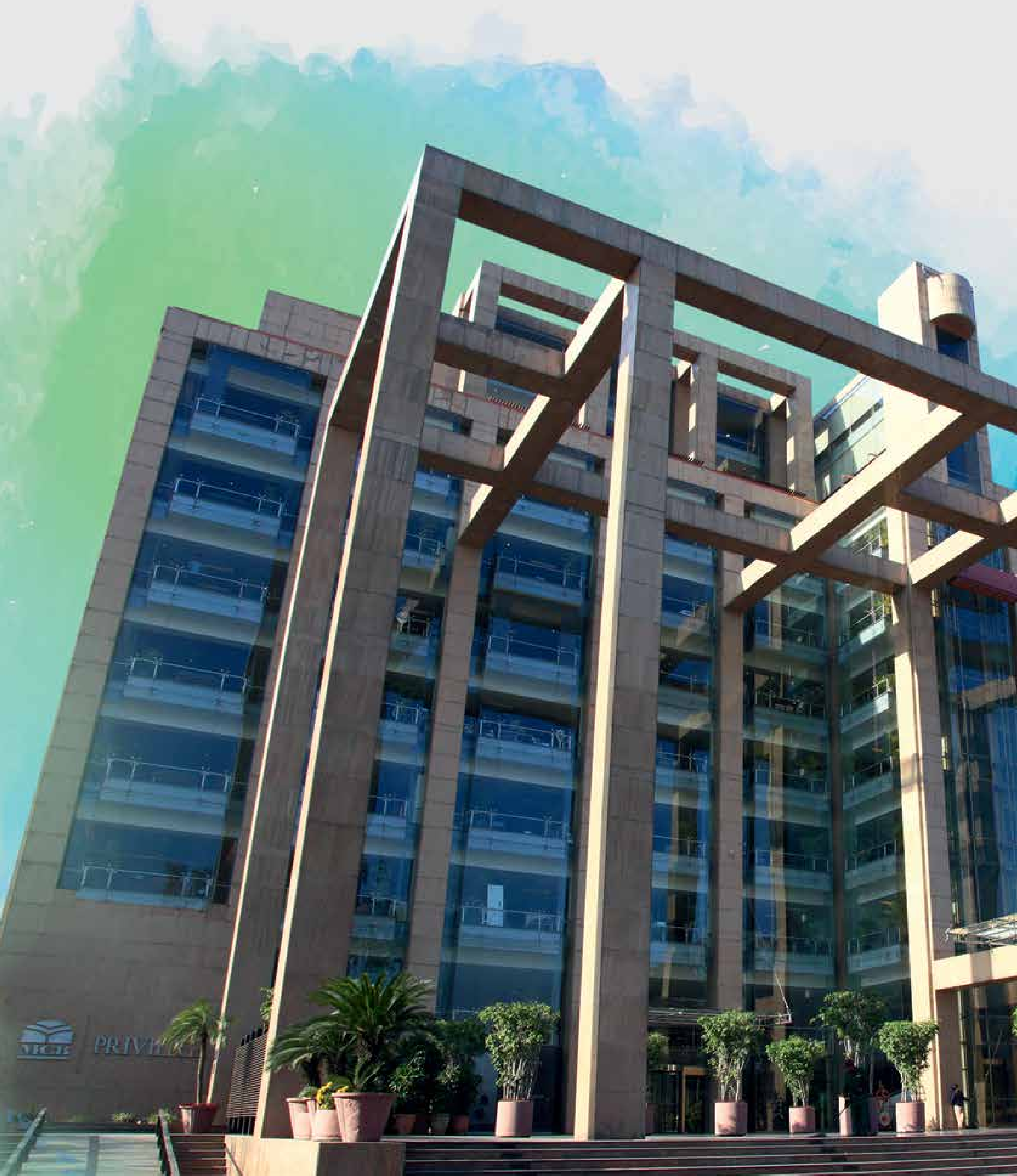

Defining Values

MCB Bank has been with Pakistan since the very beginning, when the country was first drawn on the world map in 1947. As one of Pakistan's oldest and most trusted banks, our identity is deeply woven into the country's history and heritage. We have come a long way together, and this journey full of challenges and accomplishments has strengthened the values we uphold and cherish.

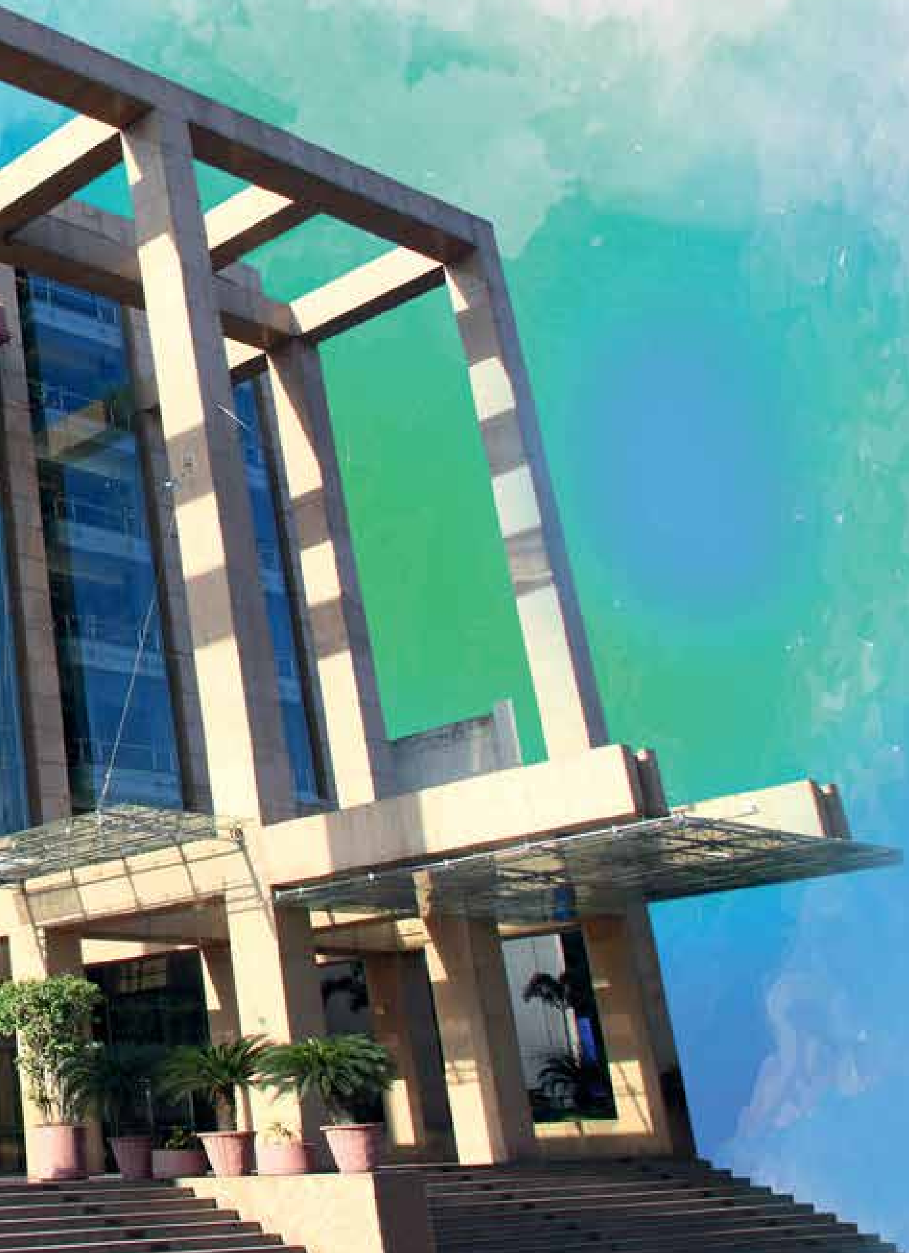

Table of **Contents**

07. Vision
07. Mission
09. Strategic Objectives
21. Core Values
22. Awards

Organizational Overview

25. Products and Services
38. Corporate Information
40. Board of Directors
42. Profile of the Board of Directors
50. Organizational Structure
52. Management Committee
54. Other Senior Management
56. Entity Credit Rating
57. Corporate Profile of the Bank
59. Chairman's Review
61. President's Review

Performance, Position & Outlook

63. Highlights 2020
64. Financial Performance 2010 - 2020
65. Forward Looking Statement
71. Graphical Presentation of Financial Statements
72. Analyses of Financial Performance
74. Analyses of Non-Financial Performance
76. Maturities of Assets and Liabilities
76. Key Interest Bearing Assets and Liabilities
77. Non-Performing Loans
78. Deposits & Advances - Sector Wise
79. Deposits & Advances - Group Wise
80. Investments
81. Capital Structure
82. Quarterly Performance - 2020 & 2019
83. Quarterly Performance Analysis - 2020 & 2019
84. Six Years' - Financial Performance / Financial Ratios 2015 - 2020
86. Six Years' - Non-Financial Performance 2015 - 2020
87. Six Years' - Performance Commentary
90. Six Years' - Graphical Summary of Ratios
91. Six Years' - Concentration of Advances, NPLs and Off Balance Sheet Items
92. Six Years' - Maturities of Assets & Liabilities
93. DuPont Analysis
94. Summary of Cash Flows
94. Free Cash Flows
95. Cash Flow Statement Direct Method
96. Markup & Non Markup Income
97. Operating Expenses
98. Economic Value Added Statement
98. Capital Expenditure
99. Six Years' - Vertical Analysis

100. Six Years' - Horizontal Analysis
101. Commentary on Six Years' Horizontal & Vertical Analysis
103. Segment Analysis
105. Statement of Charity and Donation
106. Market Statistics of MCB's Share
107. Share Price Sensitivity Analysis
108. Other Information
110. MCB Calendar of Major Events during 2020

Strategic & Resource Allocation

111. Strategic Objectives
112. Strategies in Place
112. Methods and Assumptions in Compiling Indicators
112. Change in Indicators & Performance Measures
114. Resource Allocation Plan
115. Business Model
116. Value Chain
117. Comparative Landscape and Market Positioning

Risk and Opportunities

118. Risk Management Framework
121. Risk and Opportunity Report

Directors Report & Groups Review

128. Directors Report
136. Groups Review

Corporate Governance & Stakeholders Engagement

147. Board Composition
151. Board Committees
156. Management Committee
168. Pandemic Recovery Plan by the Management and Policy Statement
169. IT Governance
170. Adoption & Statement of Adherence with the International Integrated Reporting Framework
171. Profile of Shari'ah Advisor Board
172. Role Shari'ah Advisor Board
173. Report of Shari'ah Board - English
178. Report of Shari'ah Board - Urdu
179. Sustainability & Corporate Social Responsibility
189. Certifications Acquired and International Standards Adopted
190. Green Banking Initiative
193. Stakeholders Engagement Process and Frequency
194. Investor Relation Section on Corporate Website
195. Steps to Encourage Minority Shareholders Participation in AGM
195. Stakeholder Engagement Policy & Summary of Analyst Briefing
196. Issues Raised in Last AGM
197. Statement of Value Added
198. Code of Conduct
202. Statement on Internal Controls
203. Statement of Compliance with the Code of Corporate Governance
205. Review Report to the Members on Statement of Compliance with the Code of Corporate Governance
206. Report of the Audit Committee

Unconsolidated Financial Statements

210. Auditors' Report to the Members
214. Statement of Financial Position
215. Profit and Loss Account
216. Statement of Comprehensive Income
217. Statement of Changes in Equity
218. Cash Flow Statement
219. Notes to the Unconsolidated Financial Statements
296. Annexure

Consolidated Financial Statements

318. Directors' Report on Consolidated Financial Statements
320. Auditors' Report to the Members
324. Consolidated Statement of Financial Position
325. Consolidated Profit and Loss Account
326. Consolidated Statement of Comprehensive Income
327. Consolidated Statement of Changes in Equity
328. Consolidated Cash Flow Statement
329. Notes to the Consolidated Financial Statements
410. Annexure

Other Information

422. Branch Network
425. Pattern of Shareholding
425. Categories of Shareholders
429. Notice of 73rd Annual General Meeting
436. Glossary of Terms
439. Form of Proxy
Investors' Awareness

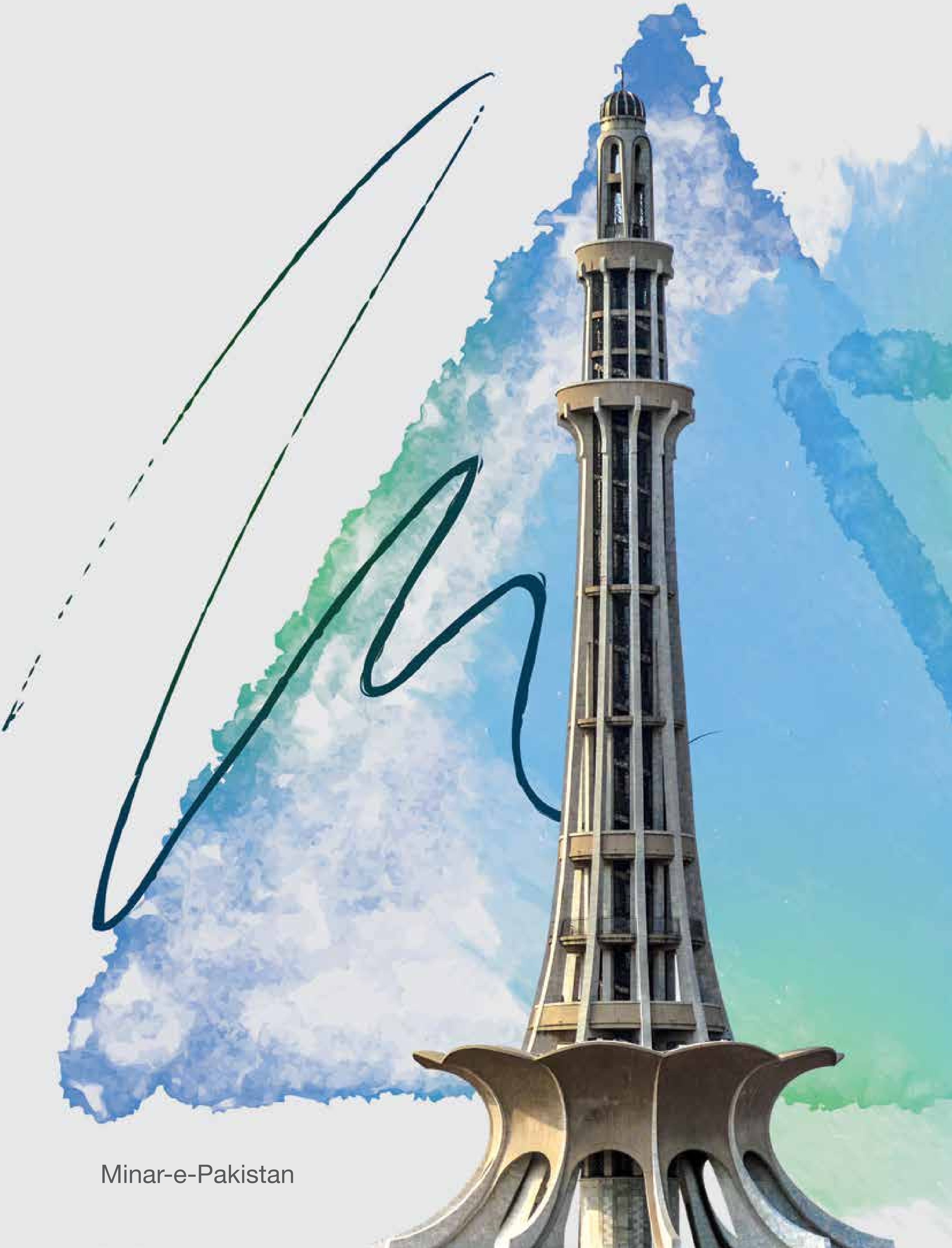

Minar-e-Pakistan

Vision

To be the leading financial services provider, partnering with our customers for a more prosperous and secure future.

Mission

We are a team of committed professionals, providing innovative and efficient financial solutions to create and nurture long-term relationships with our customers. In doing so, we ensure that our shareholders can invest with confidence in us.

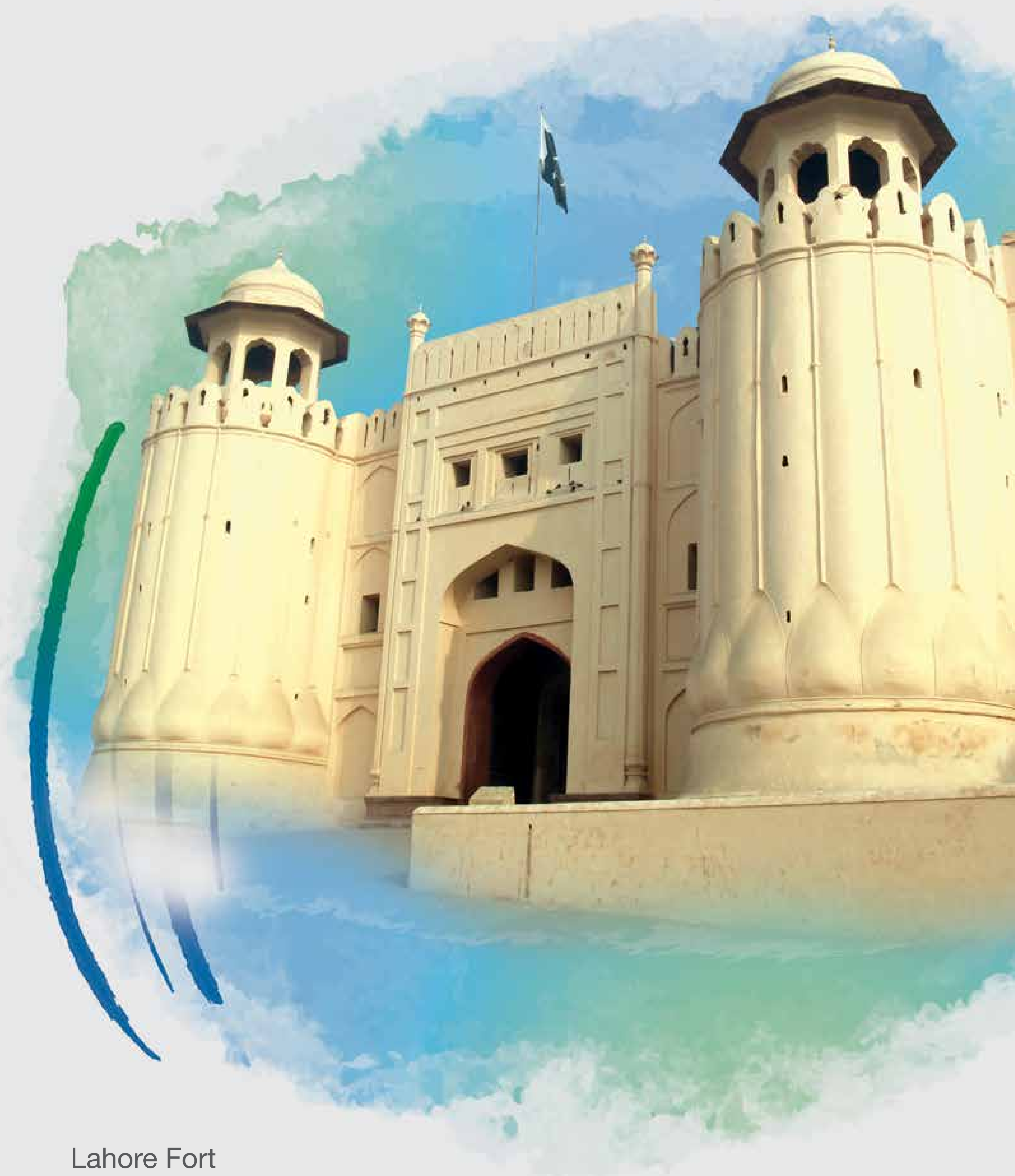

Lahore Fort

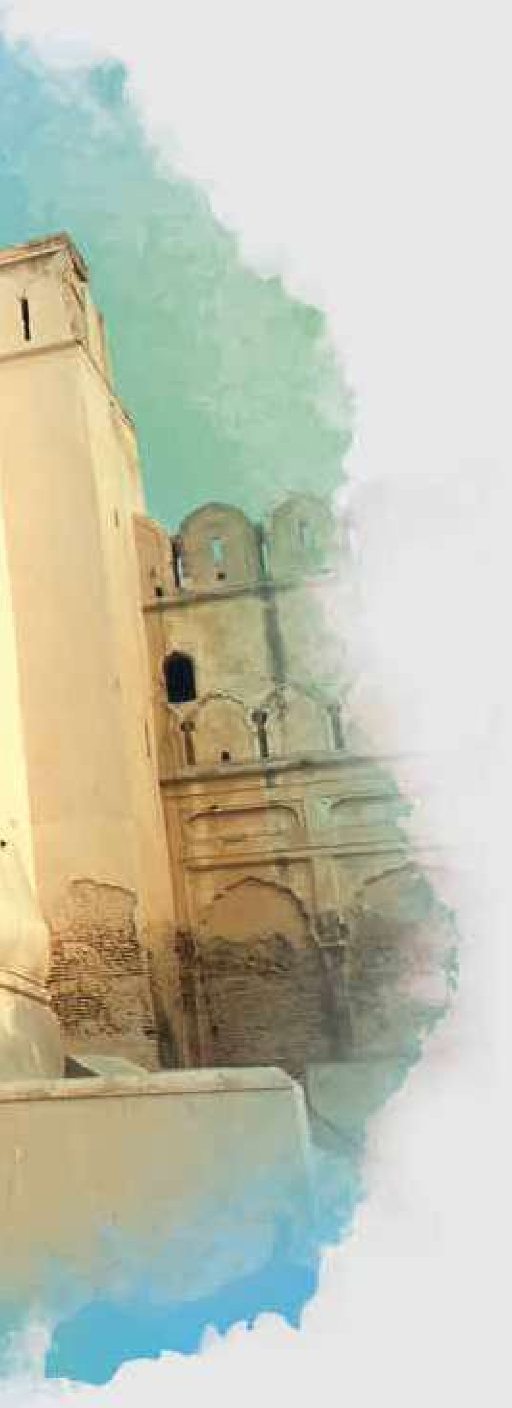

Strategic **Objectives**

Delivering remarkable returns to stakeholders, sustainable performance, exceeding market and shareholder expectations

Providing value added services through operational expansion, geography and upgraded system

Building a corporate culture of equality, trust and team spirit as we remain dedicated to being a socially responsible organization

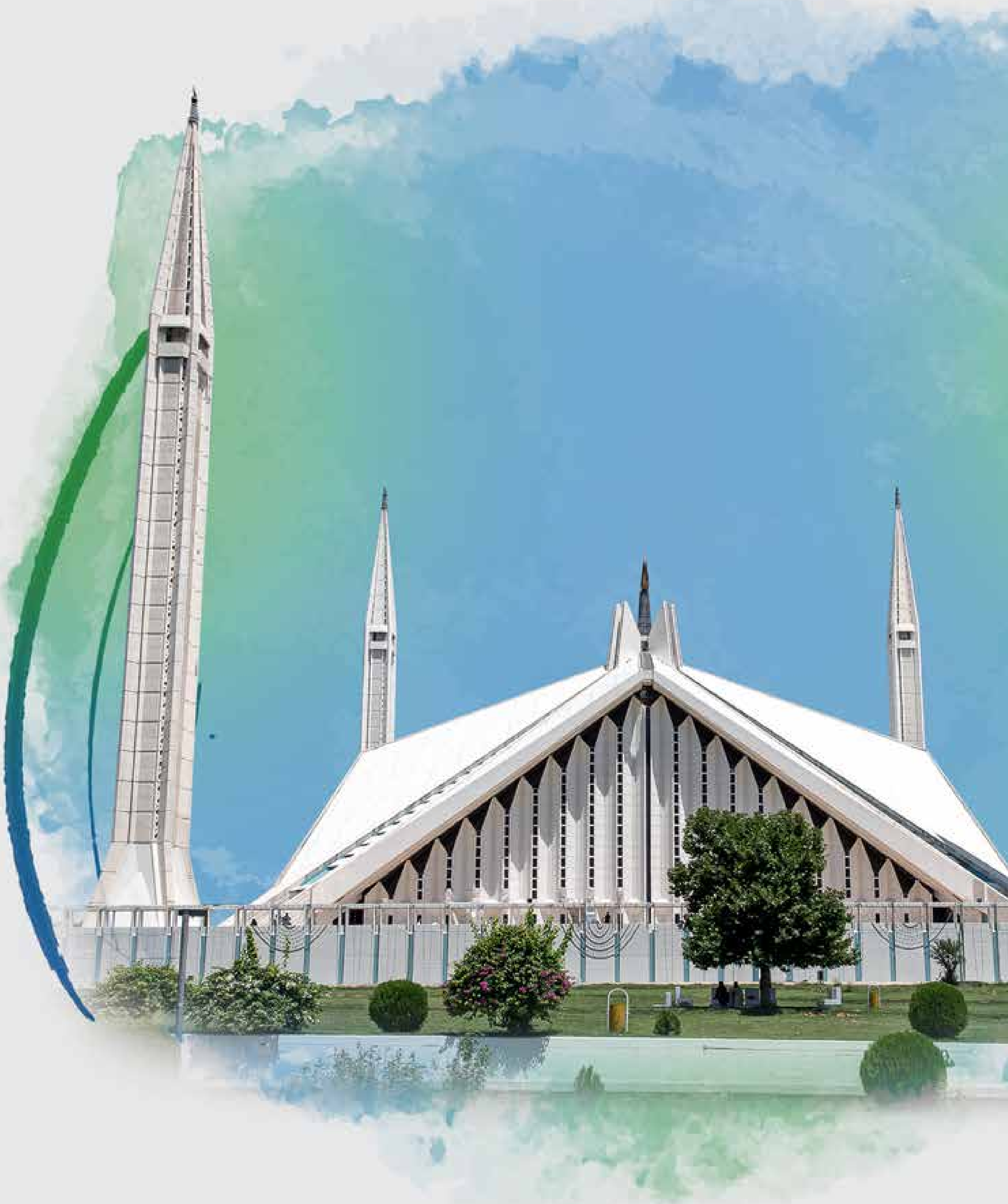

Faisal Mosque

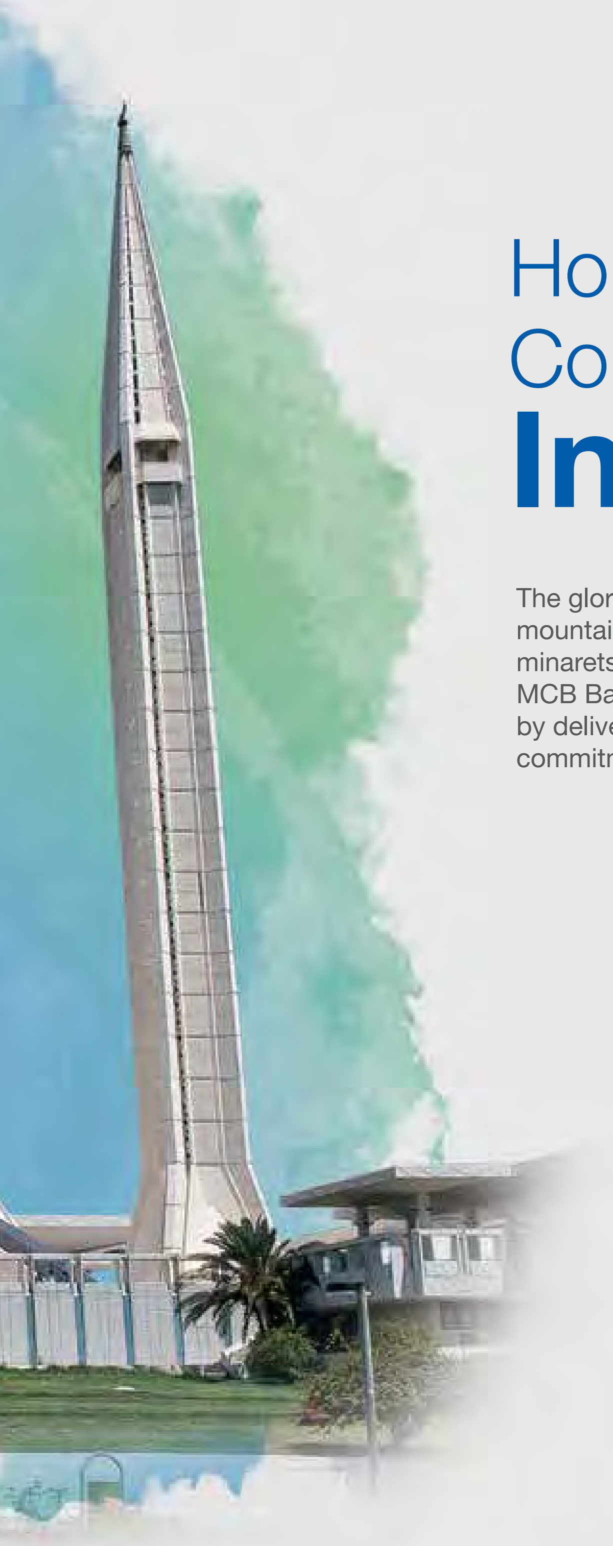

Honouring Commitments with **Integrity**

The glorious Faisal Mosque stands at the foot of the mountains in the capital city of Islamabad, with four minarets rising high as pillars of honour and integrity. MCB Bank continues to uphold these principles by delivering diligently on responsibilities and commitments to ensure our customers' success.

Leading with **Innovation**

Built at the height of the Mughal Empire, the Sheesh Mahal is an architectural marvel that consists of gold, precious stones, marble, glass and mirrors all brought together intricately in perfect harmony. We at MCB Bank strive to lead the market with innovative solutions that offer our customers the best banking services.

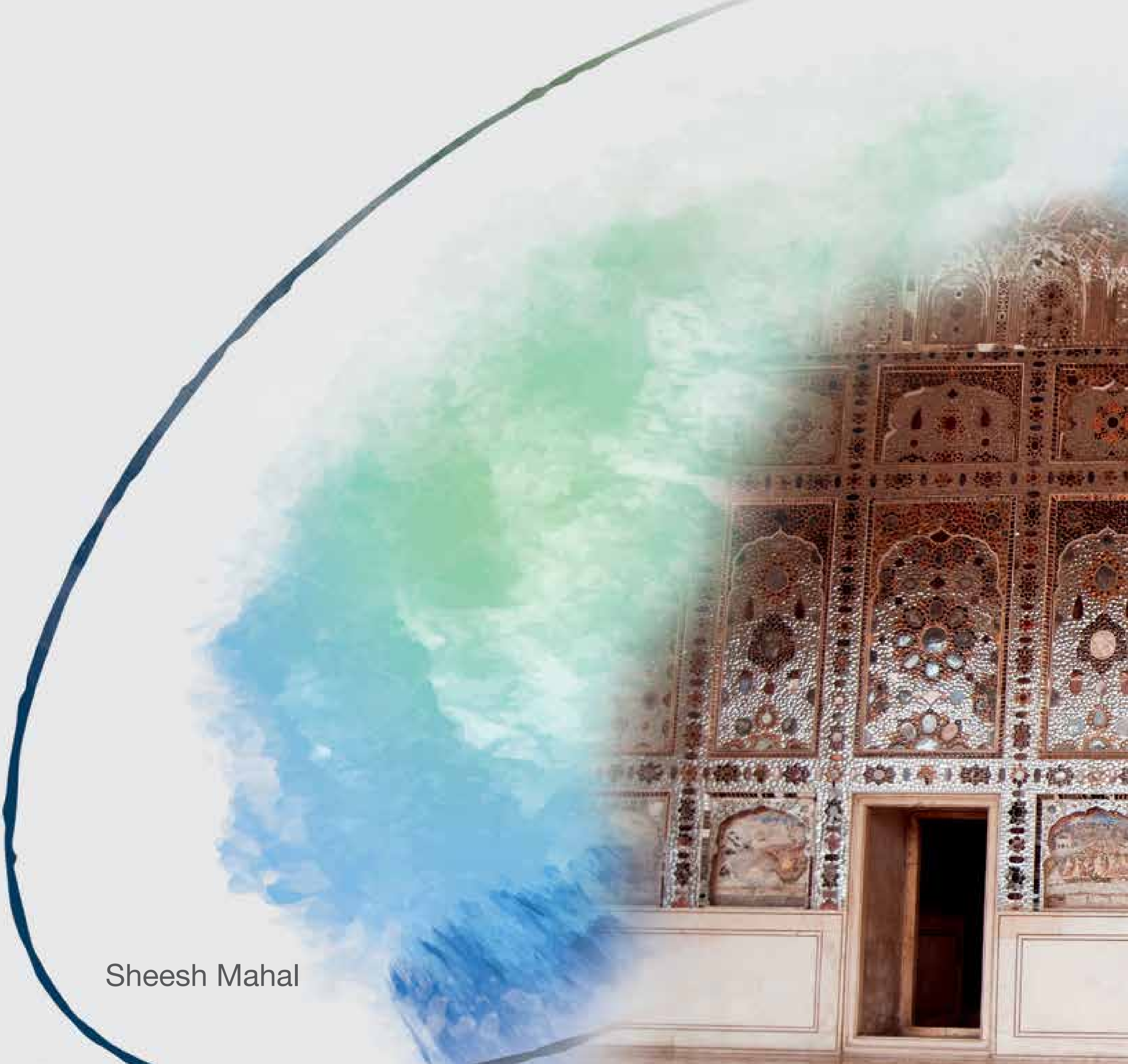

Sheesh Mahal

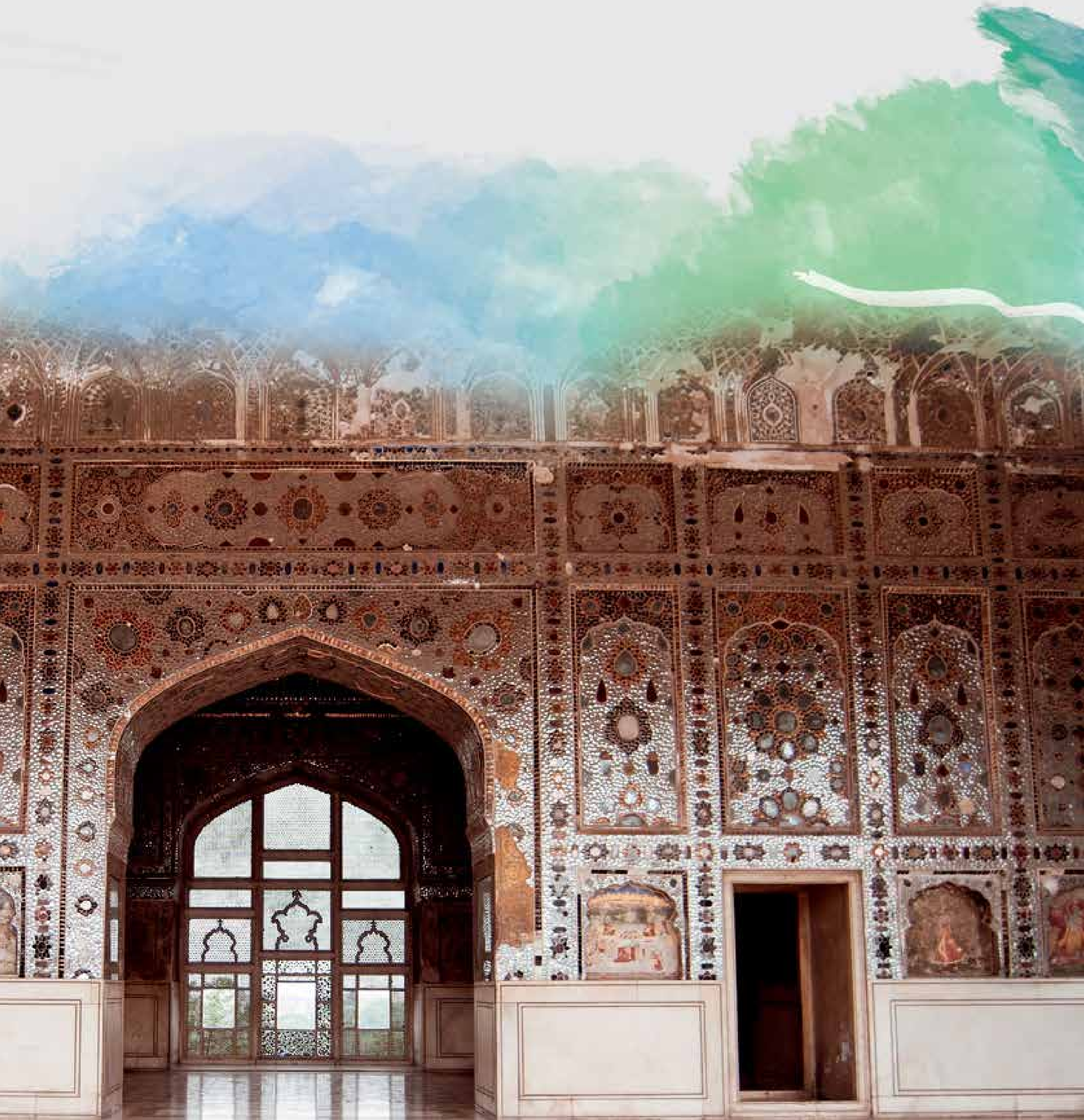

Excellence
is a Standard

A hidden gem in Bahawalpur, Noor Mahal is an elegantly built palace that brings together both Corinthian and Islamic styles of architecture in a magnificent structure. MCB Bank believes in operational excellence and moves with the times to bring our customers' aspirations to life.

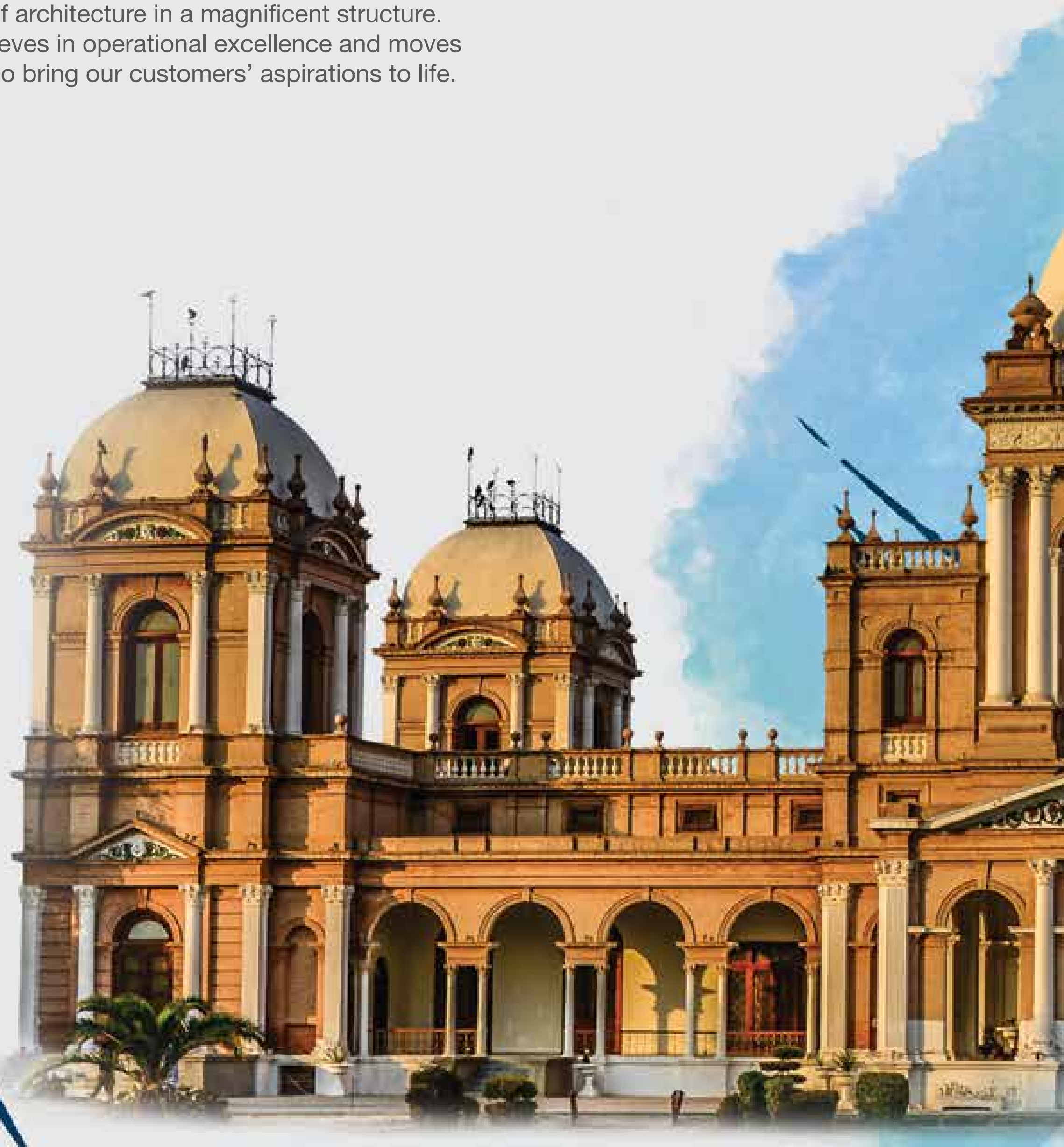

Noor Mahal

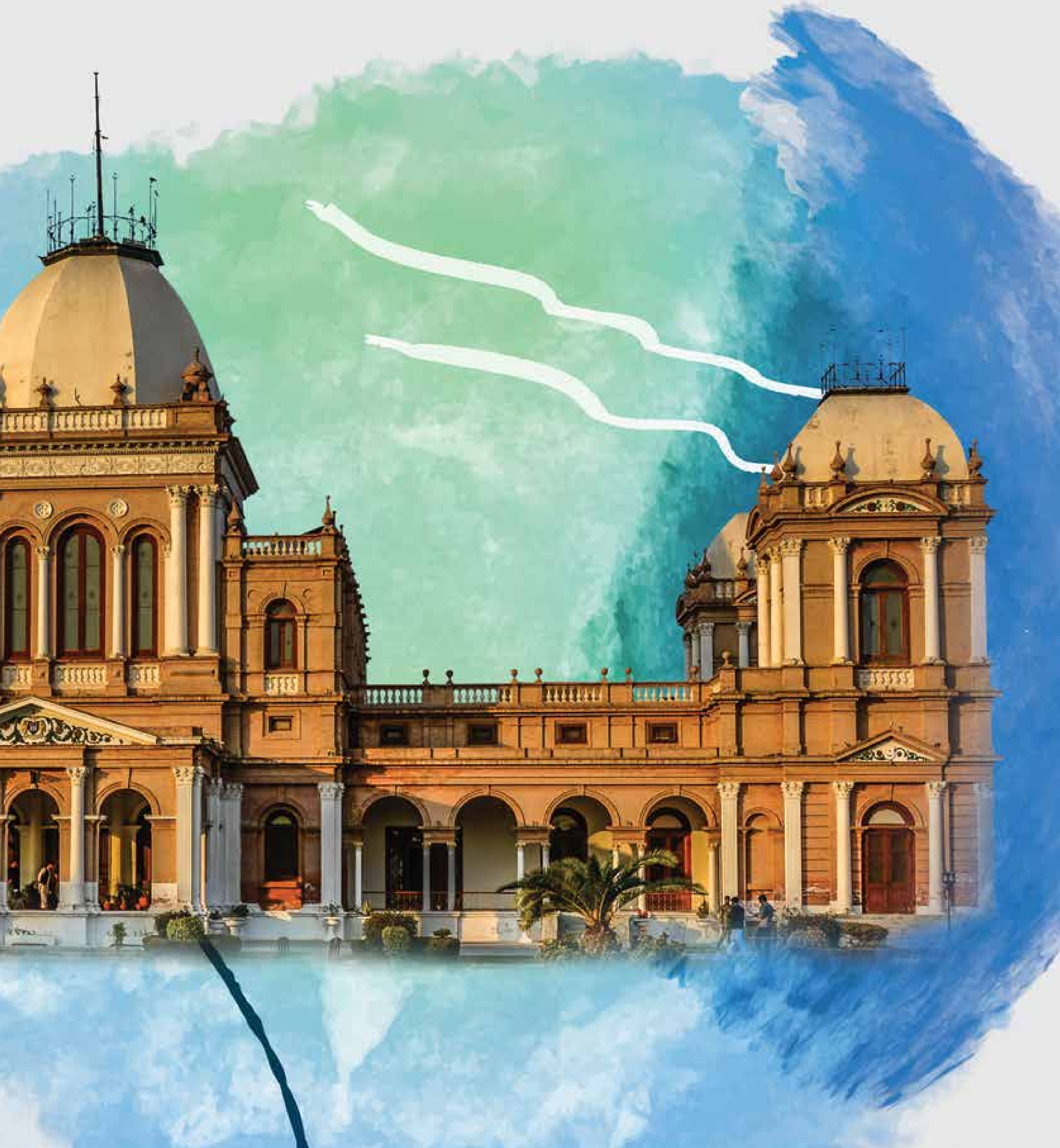

Our
Customers
are Always Connected

Built more than a century ago, the General Post Office Building in Lahore is a conduit that manages about 20,000 pieces of mail a day, delivering them throughout the city. With over 1400 branches across the nation, MCB Bank is always connected to our customers, ensuring safe and easy access.

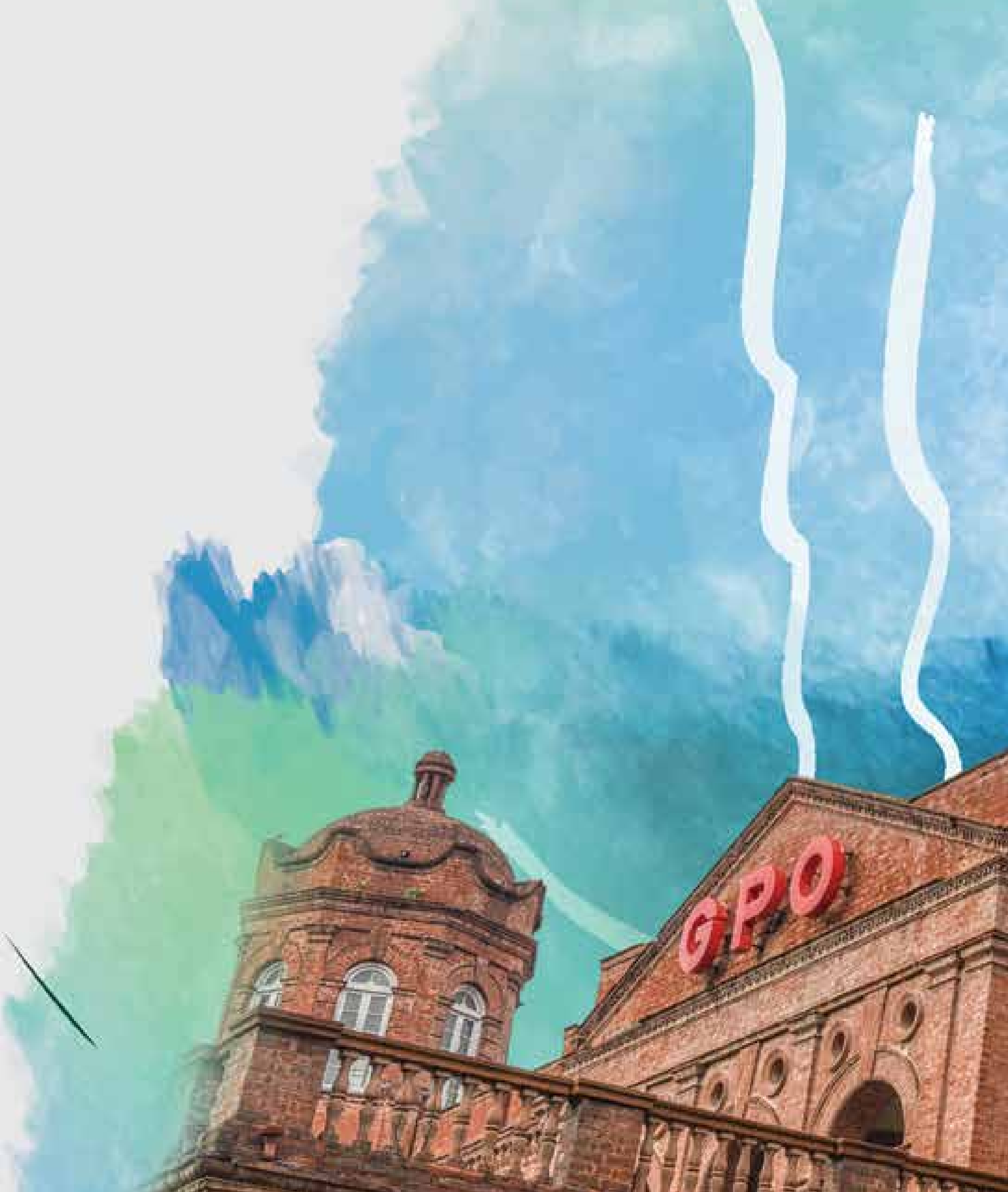

General Post Office

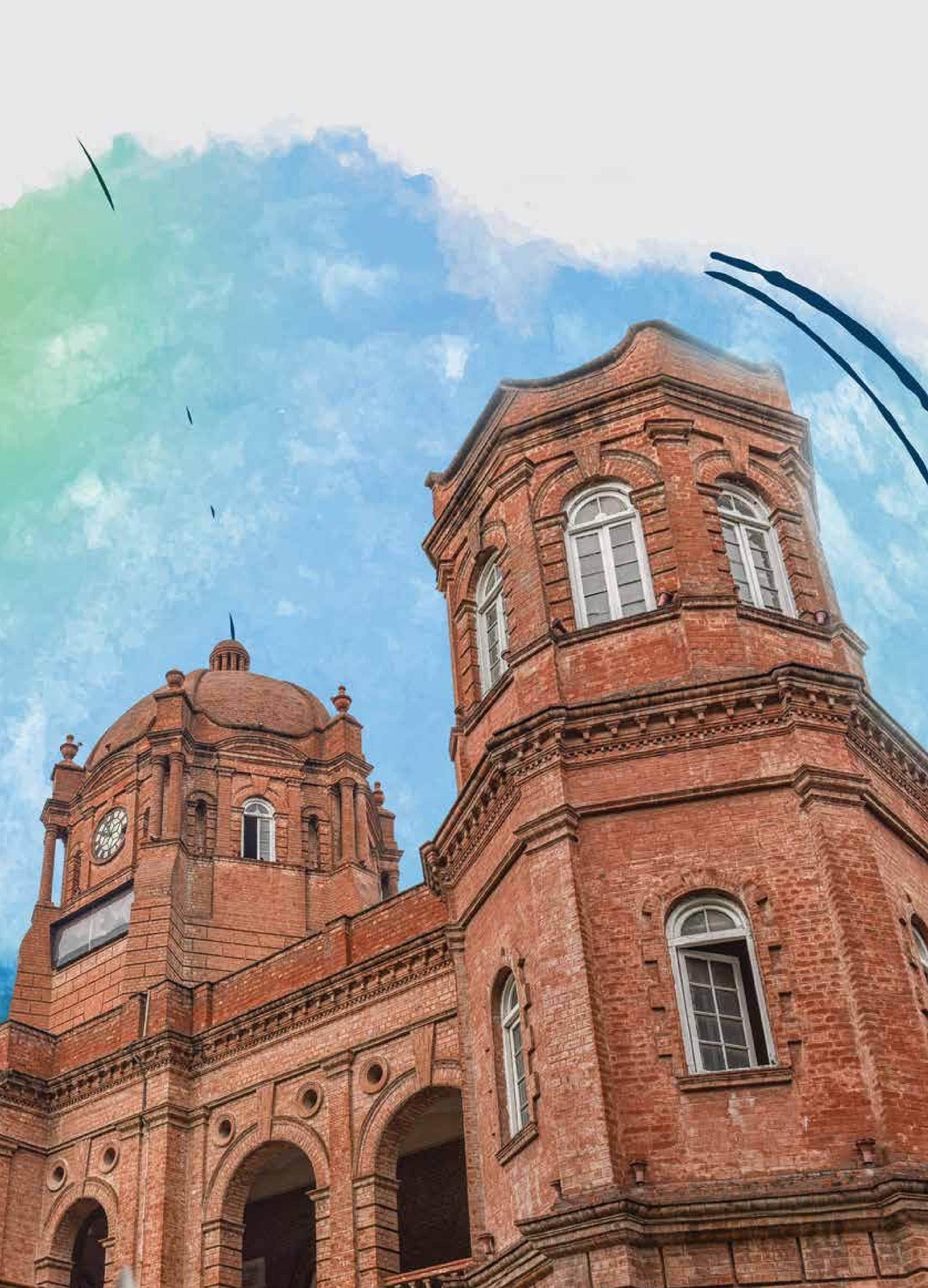

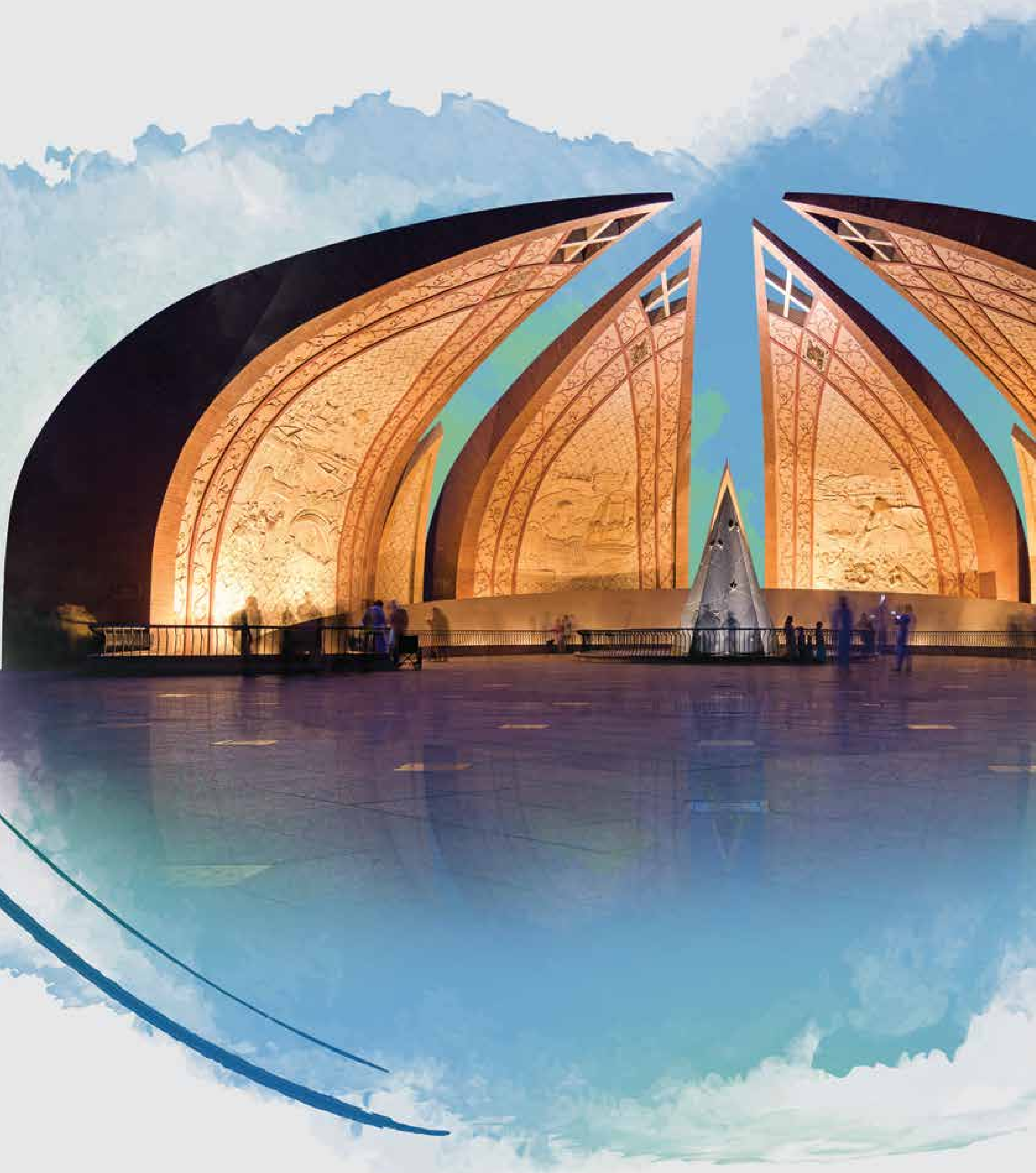

Pakistan Monument

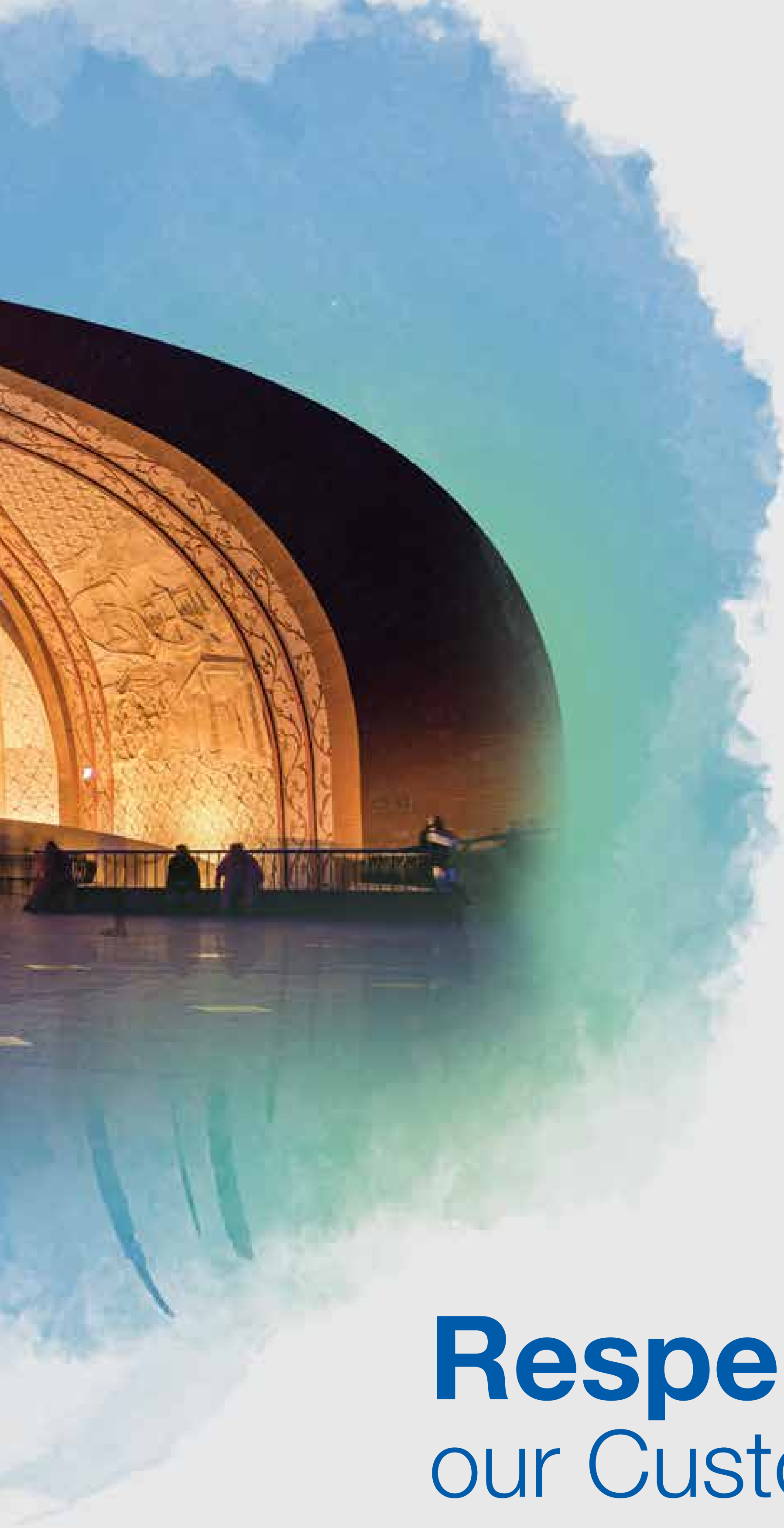

Respecting
our Customers

A striking structure in Islamabad, the Pakistan Monument was built to symbolize the unity of the country's people while also giving each province its due respect just as MCB Bank respects the needs and requirements of our customers.

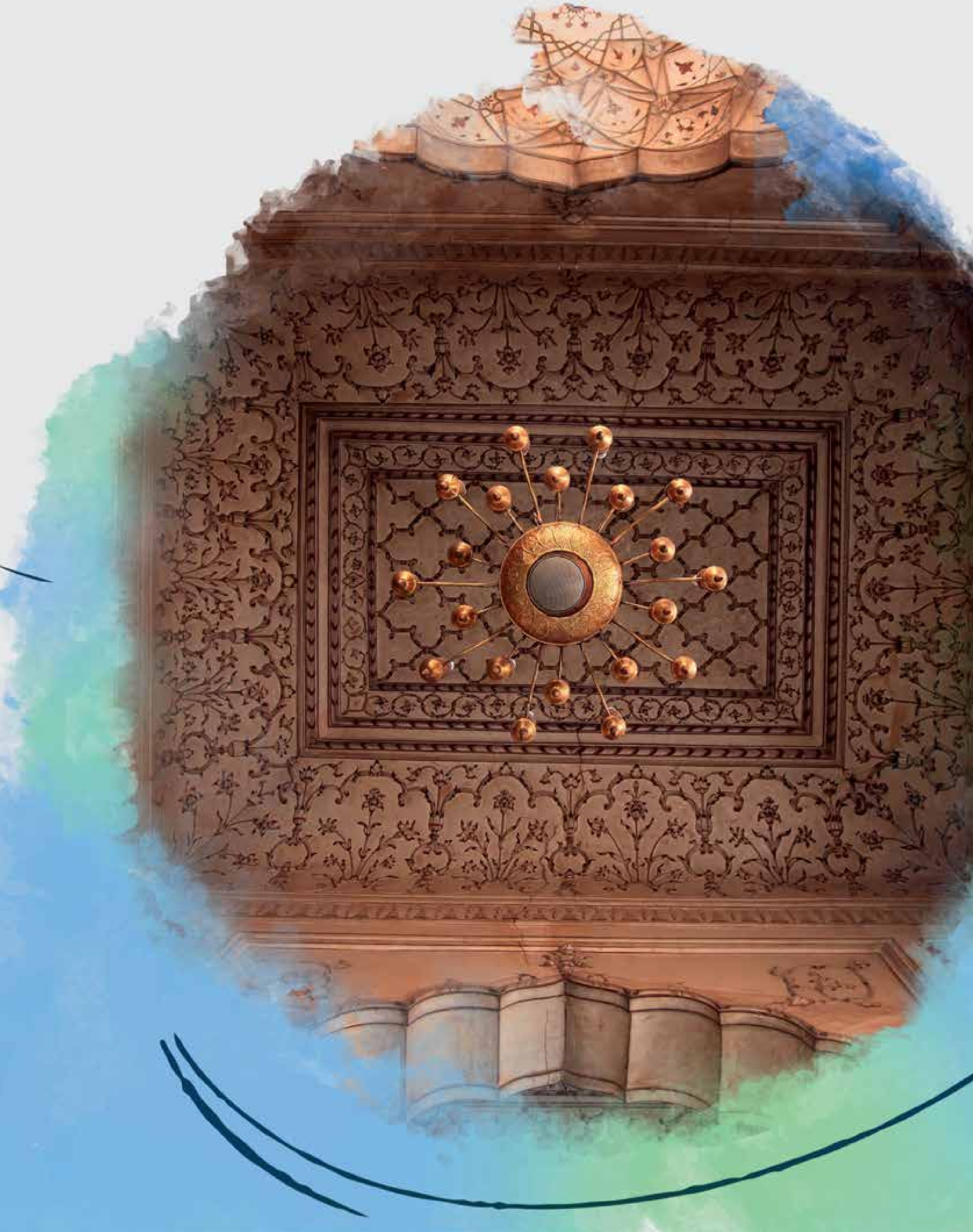

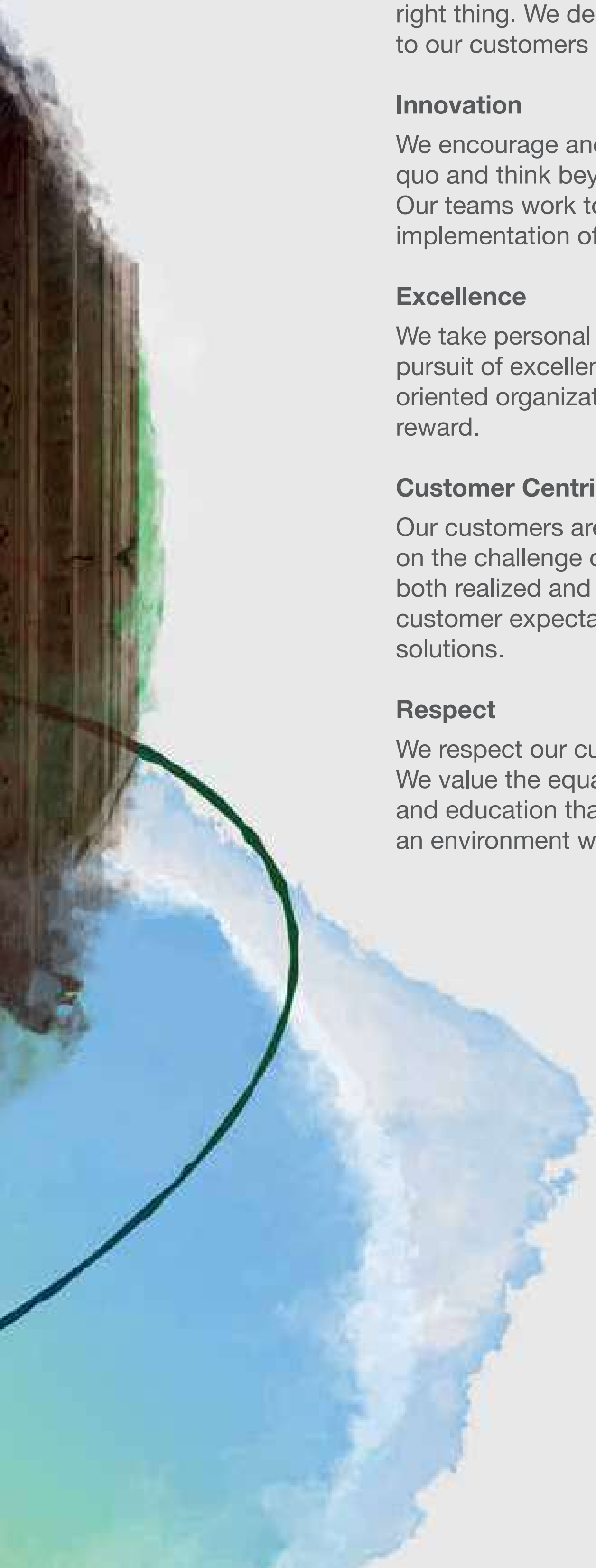

Core **Values**

Integrity

We are the trustees of public funds and serve our community with integrity. We believe in being the best at always doing the right thing. We deliver on our responsibilities and commitments to our customers as well as our colleagues.

Innovation

We encourage and reward people who challenge the status quo and think beyond the boundaries of the conventional. Our teams work together for the smooth and efficient implementation of ideas and initiatives.

Excellence

We take personal responsibility for our role as leaders in the pursuit of excellence. We are a performance driven, result oriented organization where merit is the only criterion for reward.

Customer Centricity

Our customers are at the heart of everything we do. We thrive on the challenge of understanding their needs and aspirations, both realized and unrealized. We make every effort to exceed customer expectations through superior services and solutions.

Respect

We respect our customers' values, beliefs, culture and history. We value the equality of gender and diversity of experience and education that our employees bring with them. We create an environment where each individual is enabled to succeed.

Awards

2020 ABF Corporate & Investment Banking Awards	Best Equity Deal of the Year- Pakistan
2020 ABF Corporate & Investment Banking Awards	Syndication Loan of the Year- Pakistan
2020 Asset AAA Infrastructure Awards	Telecom Deal of the Year in Pakistan
2020 Asset AAA Islamic Finance Awards	Best Corporate Sukuk
2020 Asset AAA Islamic Finance Awards	Best Acquisition Financing
2020 Asset AAA Islamic Finance Awards	Best Syndicated Loan
2020 Asiamoney Awards	Overall Most Outstanding Company in Pakistan
2020 Asiamoney Awards	Most Outstanding Company – Financials Sector in Pakistan
2020 ICAP and ICMAP	Best Corporate Report Award 2019 – Winner
2020 SAFA Awards	Joint 1st Runner up Best Presented Annual Accounts 2019 - Banking Sector

2019 Asset Triple A Infrastructure Awards	Transport Deal of the Year
2019 Asset Triple A Infrastructure Awards	Utility Deal of the Year
2019 Asian Banking & Finance Corporate & Investment Banking Awards	Equity Deal of the Year – Pakistan
2019 Asian Banking & Finance Corporate & Investment Banking Awards	Mergers and Acquisitions Deal of the Year – Pakistan
2019 Asiamoney	Most Outstanding Company in Pakistan – Financial Sector
2019 FinanceAsia	FinanceAsia Country Awards – Best Bank Pakistan
2019 ICAP and ICMAP	Best Corporate Report Award 2018 – Winner
2019 SAFA Awards	Certificate of Merit 2018 – Private Bank Category

2018 Asset Triple A Islamic Finance Awards	Best Islamic Loan Adviser, Pakistan
2018 Asset Triple A Infrastructure Awards	Project Finance House of the Year, Pakistan
2018 Asset Triple A Infrastructure Awards	Renewable Energy Deal of the Year – Solar, Pakistan to MCB Bank Limited, MCB Bahrain & MCB Dubai
2018 Asset Triple A Infrastructure Awards	Oil and Gas Deal of the Year, Pakistan
2018 Asset Triple A Infrastructure Awards	Telecom Deal of the Year, Pakistan
2018 Asiamoney Best Bank Awards	Best Domestic Bank
2018 FinanceAsia Country Awards for Achievement	Best Bank in Pakistan
2018 ICAP & ICMAP	Best Corporate Report Award 2017 - Winner

2017 Euromoney Awards	Best Investment Bank in Pakistan
2017 ICAP and ICMAP	Best Corporate Report Award 2016 - Winner
2017 Assets AAA Islamic Finance Awards	Best Project Finance House in Pakistan
2017 Assets AAA Infrastructure Awards	Project Finance House of the Year in Pakistan
2017 Assets AAA Infrastructure Awards	Renewable Energy Deal of the Year
2017 Assets AAA Infrastructure Awards	Transport Deal of the Tear
2017 Asiamoney Silk Road Finance Awards	Best Regional Bank in South Asia for Belt & Road Initiative (BRI)
2017 Asiamoney Silk Road Finance Awards	Best Bank in South Asia for Belt & Road Initiative (BRI)
2017 Asian Banker Awards	Strongest Bank in Pakistan
2017 FinanceAsia Achievement Awards	Best Pakistan Deal, IPO of Pakistan Stock Exchange
2017 Assets AAA Country Awards	Best Equity Pakistan IPO of Pakistan Stock Exchange
2017 CFA 14th Excellence Awards	Runner Up - Corporate Finance House of the Year
2017 SAFA Awards	Certificate of Merit Best Presented Accounts 2016 - Banking Sector
2017 SAFA Awards	Certificate of Merit SAARC Anniversary Awards for Corporate Governance

2020 Asiamoney Awards
Overall Most Outstanding Company in Pakistan

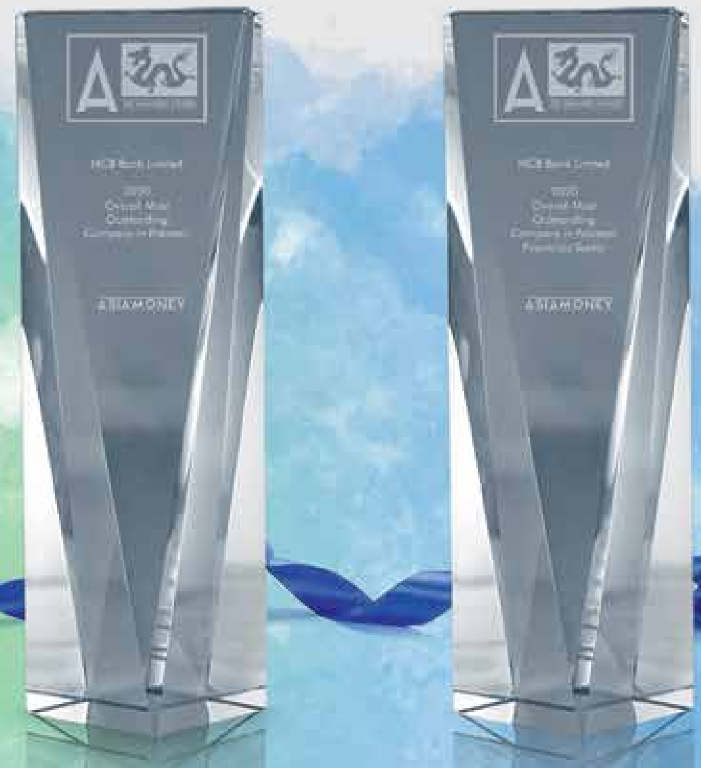

2020 Asiamoney Awards
Most Outstanding Company Financial Sector in Pakistan

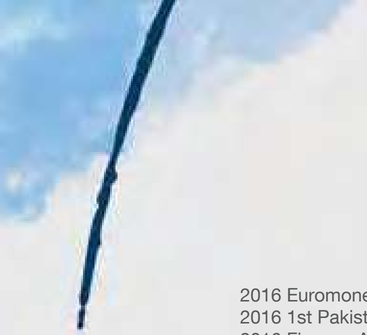

2016 Euromoney Awards	Best Bank in Pakistan 2016
2016 1st Pakistan Banking Awards	Best Bank for Corporate Finance & Capital Market Development
2016 Finance Asia Country Awards	Best Bank in Pakistan 2016
2016 ICAP and ICMAP	Best Corporate Report Award 2015 - Winner
2016 First Global Awards	Most Innovative Investment Bank for Islamic Finance
2016 Assets AAA Country Awards	Best Micro Finance Deal for National Rural Support Programme
2016 SAFA Awards	Certificate of Merit Best Presented Accounts 2015 - Banking Sector
2016 SAFA Awards	Certificate of Merit SAARC Anniversary Awards for Corporate Governance
2015 CFA 12th Excellence Awards	Most Stable Bank of the Year 2014
2015 Finance Asia Country Awards	Best Bank in Pakistan 2015
2015 ICAP and ICMAP	Best Corporate Report Award 2014 - Winner
2015 SAFA Awards	Winner of Best Presented Annual Accounts 2014 - Banking Sector
2014 The Asset Triple A	Best Bank - Pakistan
2014 The Asset Triple A	Best Domestic Bank - Pakistan
2014 CFA 11th Excellence Awards	Best Bank of the Year 2013 – Large Bank
2014 CFA 11th Excellence Awards	Most Stable Bank of the Year 2013
2014 Asiamoney Awards	Best of the Best Domestic Bank
2014 The Asian Banker (USA)	Strongest Bank in Pakistan 2014
2014 ICAP and ICMAP	Best Corporate Report Award 2013 - Winner
2014 SAFA Awards	1st Runner up Best Presented Annual Accounts 2013 - Banking Sector
2013 The Asset Triple A	Best Domestic Bank - Pakistan
2013 The Asset Triple A	Best Islamic Deal
2013 ICAP and ICMAP	Best Corporate Report Award 2012 - Winner
2013 LK Domain Registry Sri Lanka	Best Website Award
2013 Lanka Clear Pvt. Limited	T+1 Cheque Clearing Award
2013 SAFA Awards	Certificate of Merit Best Presented Annual Accounts 2012 - Banking Sector
2012 The Asset Triple A	Best Domestic Bank - Pakistan
2012 Euromoney	Best Bank in Pakistan
2012 NFEH	CSR Business Excellence Award “Best Media Coverage”
2012 ICAP and ICMAP	2nd Best Corporate Report Award 2011 - Banking Sector
2012 World Finance	Best Commercial Bank - Pakistan
2012 Pakistan Centre for Philanthropy	PCP Corporate Philanthropy Award
2012 SAFA Awards	2nd Runner up Best Presented Annual Accounts 2011 - Banking Sector
2011 CFA Association Pakistan	Most Stable Bank of the Year
2011 CFA Association Pakistan	Best Bank of the Year
2011 Euromoney	Best Bank in Pakistan
2011 ICAP / ICMAP	Best Corporate Report Award 2010 - Winner
2011 SAFA Awards	Joint 2nd Runner up Best Presented Annual Accounts 2010 - Banking Sector
2010 The Asian Banker	Strongest Bank in Pakistan
2010 The Asian Banker	Leadership Achievement Award
2010 MMT	Best Bank Led MMT Service
2010 ICAP / ICMAP	Best Corporate Report Award 2009 - Winner
2010 SAFA Awards	Certificate of Merit Best Presented Annual Accounts 2009 - Banking Sector
2009 Asiamoney	Best Domestic Bank in Pakistan
2009 The Asset	Best Domestic Bank in Pakistan
2008 Euromoney	Best Bank in Asia
2008 Euromoney	Best Bank in Pakistan
2008 Asiamoney	Best Domestic Bank in Pakistan
2006 Asiamoney	Best Domestic Bank in Pakistan
2006 Euromoney	Best Bank in Pakistan
2005 Asiamoney	Best Domestic Bank in Pakistan
2005 Euromoney	Best Bank in Pakistan
2004 Euromoney	Best Bank in Pakistan
2004 Asiamoney	Best Domestic Bank in Pakistan
2003 Euromoney	Best Bank in Pakistan
2001 Euromoney	Best Bank in Pakistan
2000 Euromoney	Best Bank in Pakistan

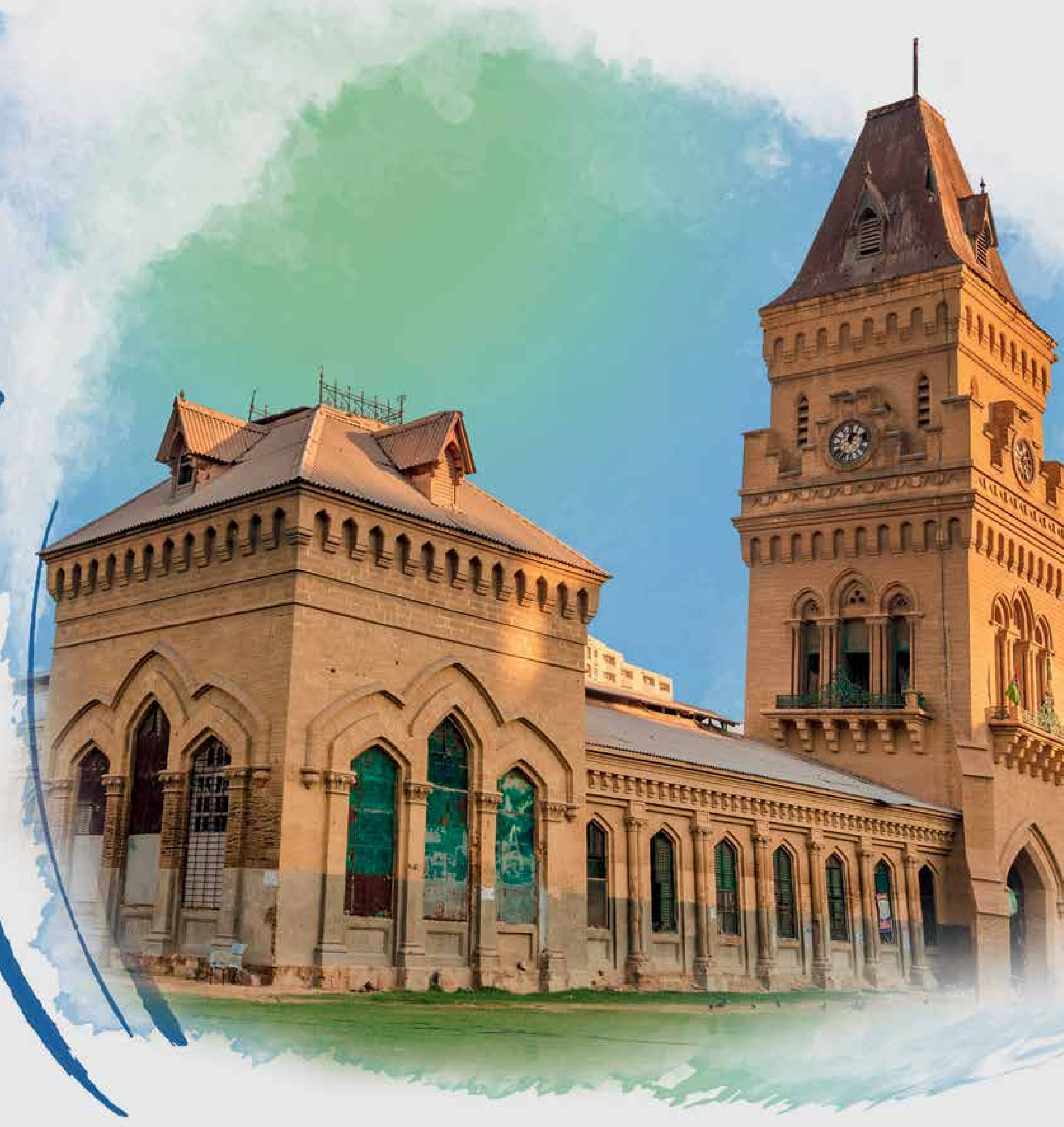

Empress Market

Products
& Services

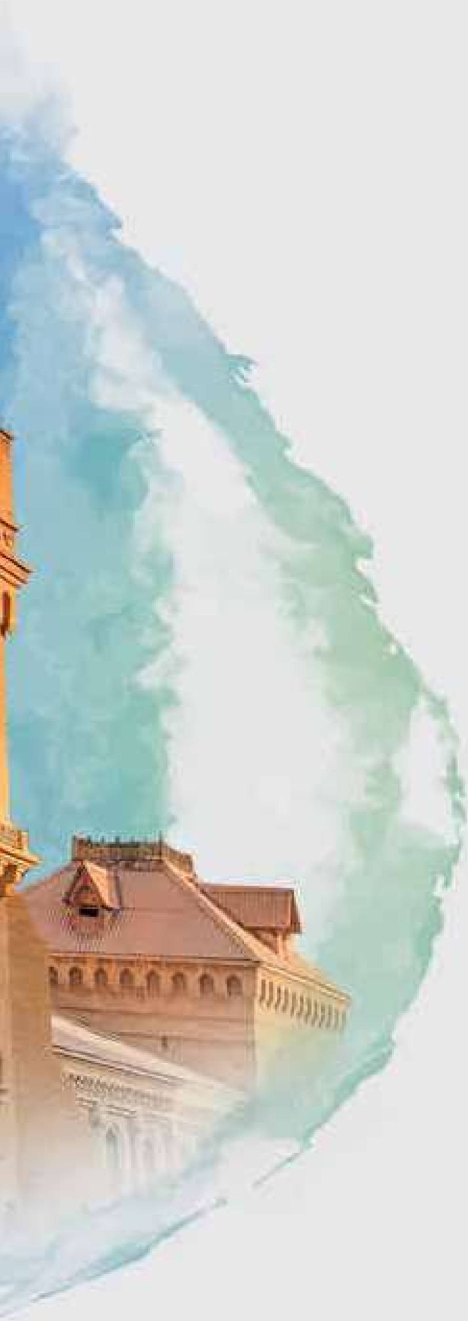

MCB Liability Products

MCB Bank offers a wide variety of products and services, hence ensuring ease and freedom for the customer to bank from its branch network compromising of 1,400+ locations geographically spread across the country. The product suite caters to all types of customer segments' banking needs ranging from individuals to corporate entities. Below are the main categories of liability products followed by product wise details.

MCB Current Deposit Products Category

For complete day-to-day banking needs, MCB Current Deposit menu is offered in local and foreign currency and is designed to provide valued customers with transactional accessibility and flexibility for all their financial dealings.

MCB Savings Deposit Products Category

MCB Bank offers a wide array of local and foreign currency savings products that cater to their daily saving and transactional needs. With multiple profit payment options, the savings deposit menu offers attractive profit rates on various savings products.

MCB Term Deposit Products Category

MCB Term Deposits offer attractive short / medium / long term investment options with flexibility, convenience and security. With various tenors, multiple currency and profit payout options, customers can choose the one that best suits their needs. For further convenience, facility of rollover and renewal is also available. Additionally, these term deposits can also be collateralized to avail credit facilities.

In order to meet the needs of MCB Bank's diverse clientele, the Bank is offering a plethora of products perfectly suited for each segment's needs:

- **MCB One Current Account:** A unique all-in-one tiered product that caters to the checking account needs of all customer segments and demographics. MCB One Current Account is a holistic financial solution that provides free services (banker's cheque, cheque book, debit card, intercity transactions, SMS facility, e-statement). These unique benefits vary based on average monthly account balances.

- **Roshan Digital Account:** Roshan Digital Account (RDA) is a flagship initiative of State Bank of Pakistan. It is a tailor-made digital financial solution, designed to facilitate Non-Resident Pakistanis (NRPs) and Resident Pakistanis (with declared foreign assets). The account provides innovative banking services in Pakistan including but not limited to digital payments, savings and investments. RDA is available in both current and savings variants. For the first time in Pakistan, NRPs are being provided the opportunity to remotely open an account through a digital process without the need to visit a bank/ branch in-person. The customer can open RDA in PKR and multiple foreign currencies and with minimal documentation. Through this account, customers can invest in lucrative Naya Pakistan Certificates, Government of Pakistan securities, real estate and Pakistan Stock Exchange among many other facilitations. Most of the products and services associated with Roshan Digital Account are free of charge.
- **MCB Smart Business Account:** A business account variant offering free services & transaction facilities by maintaining a minimal average balance requirement. The account is targeted towards business entities in a more segmented and focused manner.
- **MCB Salary Club Account:** A unique product offering targeted towards institutions / companies to manage payroll by getting employee accounts opened with MCB Bank. MCB Salary Club Account has both Current and Savings variants through which employees can avail various free benefits & discounts on different services.
- **MCB Ladies Account:** MCB Ladies Account is targeted specifically towards women with the main objective to create a niche for females of Pakistan and give them the freedom of managing their own finances with a sense of security and independence. This product offers various benefits including free insurance with a unique blend of health, accident, critical illness and death coverage along with attractive discounts on other services. The merchandise is feminist in its design / colors and the customer is given multiple options to choose a cheque book or a debit card of choice.
- **MCB Senior Citizens Account:** MCB Senior Citizens Account comes in both Current and Savings variants and aims to provide exclusive privileges in order for our elderly customers to take care of their financial needs with ease, while providing discounts on a wide array of services. 50% discount on numerous services can be availed by opening up a MCB Senior Citizens Account.
- **MCB Pensioners Account:** MCB Pensioners Account comes in both Current and Savings variants and allows account holders to live their lives to the fullest and face the future with confidence. This account is designed especially to cater to the financial needs of pensioners and is in compliance with regulatory governance. 50% discount on numerous services can be availed by opening up a MCB Pensioners Account.
- **MCB Asaan Account:** A current deposit account with simplified account opening requirements is designed to extend benefits of financial services to unbanked segments of society. This product aims to improve economic growth of potential customers under the financial inclusion initiative of State Bank of Pakistan.
- **MCB 365 Savings Gold Account:** This account enables customers to enjoy attractive returns on their deposits on a monthly basis. Special saving rates are offered to entities / institutions / corporates on maintaining large deposits intermittently.
- **MCB Burqraftaar Remittance Account:** This is a promising product to serve home remittance consumers with security, convenience, and accessibility. Remitters from various countries are provided access to multiple money exchange companies from where they can remit directly in MCB Burqraftaar Remittance Account instantly. Also, the bank provides more than 100 dedicated remittance centers across the country where the beneficiaries can collect cash in person. Further, the account offers withholding tax exemptions on cash withdrawal, free debit card, insurance coverage to both the remitter and beneficiary.
- **MCB Asaan Remittance Account** is another initiative by the SBP under the financial inclusion program to provide secure home remittance inflows. The account is available in the Current variant only and is targeted towards unbanked/under-banked remittance beneficiaries of Pakistan, with simplified account opening requirements. This product not only increases the Bank's product portfolio but also fulfills its fiduciary responsibility of documenting the economy and inculcating a saving habit in its customers.
- **MCB E-Statements:** MCB Bank's E-Statement initiative adds an additional layer of convenience for our digital savvy customers. This service is free for all customers and provides easier access to banking information when needed without visiting the branch. This service also augments Bank efforts to reduce use of paper and facilitate Green Banking guidelines issued by SBP.

MCB Privilege Banking

Privilege Banking takes pleasure in taking you on a journey of superior high-end customer services, a rewarding in branch experience, a wide array of financial services, investment opportunities and transactional convenience, via dedicated, contemporary and service-oriented sales force. These multi-dimensional banking relationships experience positions privilege segment customers at

unparalleled advantages that put them ahead of others. MCB Bank has eight dedicated Privilege Centers waiting to welcome you in Karachi, Lahore, Islamabad, Faisalabad, Rawalpindi and Multan with plans to expand to even more locations.

MCB Agri Financing Products

Agriculture finance business of the Bank has embraced a new & progressive outlook as a result of various initiatives. The bank has strengthened its Agri. financing structure in terms of required delegation of approving authority and deployment of dedicated Human Resources at the branch level. A well-equipped, trained & experienced team of agriculture marketing officers has been put in place to facilitate farmers on their door steps, for completion of documents and revenue related formalities, along with providing them awareness on banking facilities, products and financial management.

The health and size of the Bank's Agri. portfolio is gradually expanding with a focus on encouraging mechanized farming. Moreover, insurance arrangements are in place to provide risk coverage to crops/tractors & equipment. Farmers are availing credit facilities to meet input needs for poultry, dairy and fish farms apart from crops. All credit proposals are processed as per standard guidelines of credit policy of the bank and approved on merit.

MCB Bank fully supports all Government and State Bank of Pakistan initiatives for promotion and steady flow of credit to the farmers. This lends support to the national cause of food security for the people of Pakistan and to exploit the potential of agriculture sector. Efforts are made to enhance outreach to growers through innovative lending including value chains and processing units. MCB Bank will continue to support agriculture sector as a matter of its policy by remaining an active partner supporting progressive farmers by providing credit for all types of farm and non-farm activities. The microcredit needs of small farmers are met through extending credit lines to NGOs/MFIs supported through digital services, thus serving the cause of financial inclusion.

The Agri. financing products offered cover requirements of both production and development needs of farm & non-farm activities. The farmers may need long term finance to undertake development projects or there may be working capital requirements. Long term financing needs are met through Term Finance whereas working capital requirements are met by production finance.

Shadabi Plan: Covers agriculture loan products for the production requirements of farm & non-farm activities of the farming community. Financing products extended under this category are Agriculture Running Finance-Revolving (ARF-R), Agriculture Production Finance (APF) and Agriculture Production Finance-Growers (APF-G). All working capital needs of nonfarm are also covered under Shahdabi Plan through APF/ARF.

Khushali Plan: Agri Development Finance (ADF) caters to the credit needs of farmers, generally long term, pertaining to the development projects related to both farm & non-farm sectors. Under Khushali Plan, different products are offered to cater to sector specific credit needs. The products offered are ADF (Tractor Finance), ADF (Aabiari Finance), ADF (Dairy & Meat finance), ADF (Murghbani Finance), ADF (Baghbani Finance), ADF (Mahigeri Finance) and ADF (Zari Technology Finance).

The amount of finance sanctioned depends upon the genuine credit requirement of the farmer and collateral. Non-farm credit (poultry, dairy[including value chains], fisheries, & others) and financing for land leveling/ development, heavy equipment, agriculture machinery, vehicles/transport for Agriculture purpose are covered under this plan.

High Efficiency Irrigation System (HEIS): The Financing Product for "High Efficiency Irrigation System (HEIS)" facilitates farmers in availing the subsidy provided under the provincial government schemes aimed at conservation of water and avoiding wastage of the precious resource. Drip and sprinkler irrigation systems are referred to as High Efficiency Irrigation Systems, (HEIS) which enable timely application of water and other inputs i.e. fertilizers, nutrients etc. as per plant requirements at various stages of growth. The HEISs are versatile in their applicability and provide complete control in irrigation operations. HEISs can be practiced on a variety of soil conditions e.g. uneven topography, odd field configurations, rolling sandy areas, etc. and are best suited for variety of crops such as orchards, vegetables, cotton, maize, sugarcane, wheat, fodder, gram etc.

Governments of Punjab and Sindh are subsidizing these high efficiency irrigation systems to farmers by contributing 60% of the total project cost and remaining 40% cost sharing by the farmers. Under HEIS Financing, MCB will provide financing to the extent of 80% of farmer's share towards installation of HEIS. The purpose of HEIS financing scheme is to facilitate farmers in adoption of high efficiency irrigation system, which in turn would help them in efficient utilization of water & other resources to improve per acre yield. The solar/renewable energy requirements of farmers for agriculture purposes are also fully supported.

There may be other development projects proposed by the farmers falling within the ambit of agriculture financing. MCB Bank is fully committed to meet all type of genuine credit needs of the farmers as a strong financial institution, concerned for and aligned with, the national cause of supporting Agriculture Sector of Pakistan.

Digital Access to record of Punjab Land Record Authority (PLRA): MCB has signed a Service Level Agreement with Punjab Land Record Authority (PLRA) to provide online access to data for a defined scope of services under the SLA. Digital access is aimed at enabling the fast track provision of documents/ information related to land record of farmers for quick decision making and sanctioning of financing facilities. The scope of services covers issuance of Fard, charge creation, and redemption. The branches are being equipped with necessary infrastructure/equipment in a phased manner for availability of online access to PLRA records. The initiative has been implemented and major Agri lending branches have started providing services to farmers. As a result of this initiative, the dream of a strong digitalized platform for dedicated support to famers of Punjab Province for quick access to banking facilities is close to realization. MCB Bank is one of the leading banks which have started offering services to the farmers under the digitalized access to PLRA record.

Loan Origination System:

MCB Bank has procured and implemented a Loan Origination System (LOS), a parameterized solution for efficient processing in order to automate the end-to-end credit approval process, optimize the loan processing activity and to gain efficiencies. LOS has been implemented for Agri Financing across the bank covering all Agri. Lending Branches. This has greatly helped in the fast processing and approval of farmers' requests for finance.

MCB is fully committed to support agriculture financing in line with SBP/Government policies and taking steps to enhance credit flow to the farming community through strengthening of internal systems and to ensure availability of required resources.

MCB Consumer Lending Products

MCB Consumer Banking offers a full suite of consumer lending products to its valued customers. The Bank's current product portfolio consists of credit cards, auto loans, home loans, personal loans, secured personal loans, unsecured overdraft facility and student personal loan (for LUMS MBA and MPHIL ELM students). At MCB Bank, the ideology behind our innovative Consumer financing products focuses on meeting three of our client's objectives simultaneously, i.e. affordability, convenience and lifestyle. MCB Consumer Finance products enhance the overall life experience of our customers. MCB Bank works to collaborate with targeted brands and organizations to ensure that we provide premium value added services to our clients. MCB Consumer Banking is ably assisted by strong back office support which includes Operations, Service Quality, Internal Audit, Compliance and a Legal Team that work together to ensure that the Bank operates efficiently and in the best interest of its customers under the regulatory framework.

MCB Car4U

The Bank's auto financing product, MCB Car4U, provides a one-stop financing solution to help our customers obtain the automobile of their dreams. Customers are free to choose between used and new vehicles, both locally manufactured and imported. MCB Car4U also allows customers to finance up to three cars simultaneously from MCB Bank in line with their unique needs and requirements. Both loan and lease variants are offered under Car4U financing. MCB Car4U offers auto loans to customers in 1,400+ of our branches across 85 cities. Both self-employed and salaried customers can apply for a MCB Car4U Loan with ease. The Bank offers a competitive markup rate to all customers and also offers a discounted markup rate to its existing Branch Customers. Over the years, the Bank has formed a strong network of auto-dealers, engaged in sale of both new/used and imported cars enabling us to facilitate our customers evolving needs. MCB Bank values its credible and expansive dealer network as one of the key pillars of its success in the auto finance business.

MCB Home Loan

Owning a home is a dream for many. At MCB Bank, we aim to help our customers fulfill this lifelong ambition and turn their dream into reality. MCB Home Loans provides financing solutions for the purchase of a home as well as for a plot and construction thereon. MCB also offers a subsidized home loan product under Government's Markup Subsidy Scheme and Low Cost Housing Scheme for special segments. Customers also have an opportunity to transfer their existing loan from any other bank to MCB through a balance transfer facility. MCB Home Loan product also caters to housing needs of non-resident Pakistanis in Pakistan. MCB Home Loan product is now available across country through a network of branches supported by a team of skilled home finance specialists placed at multiple locations in the country.

MCB Credit Cards

MCB Credit Cards are secured with Chip & PIN functionality and carry world class features that provide transactional & payment convenience to our customers across the globe. The instant SMS and E-Alert facility enables our customers to monitor their credit card transactions and be alert of potential misuse of their Credit Card. The cards are available in three different variants i.e. Classic, Gold and Platinum that cater to the diverse needs of our distinguished customers.

Besides transactional convenience, these cards also offer payment flexibility / financial convenience to the customer, such as 0% service fee on Installment Plans, the ability to transfer balances on a lower rate or even request for a Banker's Cheque in the customer's own name or direct credit in their own MCB account & accounts in other Banks.

Credit Card Bill payments are made more convenient for customers, who can now make their Credit Card bill payments through Cash, MCB Cheque, Cheques of other Banks, over the MCB Branch counters and MCB Contact Center. They can also make payments through MCB Bank and other Bank's Internet Banking, Mobile Banking & ATM services.

MCB Personal Loan:
MCB Personal Loan is a Fast, Affordable and Easy option to meet our customers' immediate financing needs. This is an unsecured product and does not require any security. This product is only available to salaried customers.

MCB Wealth Management:

MCB Bank is among the market leaders in providing Wealth Management Services to its customers. The business is constantly evolving and expanding its product suite for both Insurance and Investment products to cater to our clients' financial requirements. We are partners with some of the leading Insurance and Asset Management companies in Pakistan to deliver innovative solutions based on customer needs, future plans and risk appetite.

MCB Bancassurance:
MCB Bank strives to help the dreams of its customers come true. Your dreams may be to give your child the best education, live a dignified life after retirement or to keep your loved ones financially secure and protected.

MCB Bancassurance plans provide you with the underlying financial security you and your family deserve. We work with our insurance partners to understand your needs based on your stage in life and provide tailor-make plans which are affordable and realistic. In addition, you and your family enjoy the peace of mind which comes from being 'Har Pal Mehfooz' in case of any unforeseen eventuality. Together with our insurance partners, we have a wide range options available which ensure that you and your funds are in good hands. All plans are underwritten by leading insurance providers and all funds are managed by experts in the field to give you the optimal returns based on your risk preferences and stage of life. Combining the best of banking and financial solutions, MCB Bancassurance provides a one-stop solution for your convenience and security.

MCB Investment Services:
Investment Services operate with the aim to help you make the most of your wealth with investment opportunities that match your unique financial aspirations. MCB Investment Services offers mutual funds managed by leading fund managers of Pakistan. We can suggest products most suited for your needs, or work with you to create a personalized solution completely focused on your expectations of the capital markets. These products offer you financial liquidity with comfort and ease and assist you meet both short and long term investment needs.

MCB Digital Banking Products and Services

MCB Debit Card
With global acceptance at more than 20 million merchants and 1.5 million ATMs worldwide; MCB Debit Cards is a way forward into the changing future of payments. MCB Bank is proud to have the latest dynamic Chip & PIN based Debit Cards which allow customers to have unmatched convenience, enhanced security and round-the-clock accessibility to their funds. MCB Debit Cards are accepted at 12,000+ ATMs and 45,000+ merchants nationwide, with promotional discount schemes designed to reward our users for shopping, dining, fuel, travel, health and much more. With an MCB Debit Card, one can forget the need to carry cash. The product proposition has been further enhanced with the launch of card variants like Visa Platinum, Gold, Gold Local and supplementary cards. MCB Debit card also offers multiple international and local payment scheme cards which include Visa, MasterCard and PayPak fulfilling various customer segment needs, MCB Bank is proud to be chosen as one of the main banks to provide services to overseas Pakistani through Roshan Digital Account (RDA). MCB has started issuance of debit cards to RDA account holders as well. MCB Bank is one of the handful of banks in Pakistan who have ventured to comply themselves with the PCI DSS which is considered to be the international benchmark for card data security thus improving the overall product value proposition.

MCB ATMs
MCB Bank has one of the largest ATM networks in the country with 1,400+ ATMs which includes 154 off-site ATMs placed at commercial locations like malls, workplaces and hospitals etc. The network covers 500+ cities across the country and is steadily growing. MCB Bank has one of the best ATM uptime availability across the industry, ensured by the presence of ATM monitoring teams working 24/7, periodic ATM health checks and hardware replacements. MCB ATMs provide our customer with convenience to withdraw cash, make utility bill payments, mobile top-ups, funds transfer through its countrywide network.

MCB Mobile
MCB Mobile is the Flagship application of MCB Banks' innovative digital services. MCB Mobile aims to provide the convenience of conducting financial and nonfinancial transactions with the safest and easiest steps. Now MCB accountholders can access mobile banking with their registered mobile number by downloading MCB Mobile application from the ANDROID or IOS store. MCB Mobile is providing its customers the user-friendly experience of conducting payments hassle-free.

MCB Internet Banking
MCB Internet Banking is a user-friendly and convenient online banking platform that provides secure financial and non-financial transactions for its customers. MCB Internet

Banking has enabled the bank to deepen its Digital Banking footprint while allowing its users to make 24/7 payments and transactions.

MCB Lite

MCB Bank received its branchless banking license 8 years ago, and formally started its branchless journey with the launch of MCB Lite in 2014. MCB Lite mobile wallet allows customers to handle their daily transactions and payments in an efficient and real time manner from one's mobile phone. Wallet can be linked with any Lite debit card (local or international payment scheme) for local, international and E-commerce use. Moreover, to support foreign remittances, the enablement of Lite Remit feature has allowed customers to receive money directly into their Lite mobile wallet from anywhere in the world. MCB Lite is all set to explore new fronts and expand its overall footprint across the branchless banking arena.

MCB Card Discounts

Caring for and giving back to our esteemed customers is what MCB Card Discounts is all about. MCB Bank is proud to have the best discount offers for our prized MCB (Debit, Credit, Prepaid and Lite) cardholders. MCB offers a wide variety of discounts across all top retail merchants and brands ranging from dining, shopping and lodging. MCB Bank's motto i.e. 'Bank for Life' is truly personified through our MCB Card Discounts program. The year 2020 saw the bank partnering with around 30 top of the line discount partners for multiple strategic and long term discount deals.

MCB Alerts

MCB Bank is proud to offer its valued customers with alerts services that comprise of SMS alerts. This service allows customers to keep track of all of their banking transactions through SMS notifications. By applying to this service, customers receive real time updates whenever they conduct a transaction on any of their MCB bank accounts. It is a great way of staying updated about transactions conducted on one's account(s).

Card Acquiring (POS)

MCB Bank has a network of approximately 3,000 POS terminals deployed at key merchant locations across Pakistan. Our Point of Sale (POS) network is fully EMV compliant and accepts Visa, MasterCard, UPI, JCB and Pay-Pak card transactions from all over the world. MCB's POS Acquiring business continues to grow due to deepening of merchant relationships, round the clock support, increased POS productivity and quality of deployments.

Internet Acquiring (MCB eGate)

MCB e-Gate is a world-class online payment gateway service, formed as a result of MCB's partnership with MasterCard International. MCB e-Gate equips online businesses to accept payments reliably and securely from both credit as well as debit cards and currently facilitates more than 600+ online merchants, directly and indirectly. The online card acquisition footprint has grown and continues to grow substantially worldwide and MCB Bank is playing an integral part in developing the e-commerce market in the country. Further, to improve online shopping and merchant experience for e-commerce, MCB e-Gate payment system has now been upgraded from Mastercard Internet Gateway Service (MIGS) to Mastercard Payment Gateway Services (MPGS) which is fast, reliable and supports multiple payment modes. It also comes with the range of other value added features including Real time fraud prevention and supports Mobile Commerce.

MCB Contact Center

The human voice is still valued as the most trusted and relevant form of communication medium by a majority of our clients. This has inspired us to constantly improve our service and upgrade our MCB Contact Center to the world's best Platform. This proactive upgrade has enabled us to enhance service delivery across multiple channels while allowing us to address specific needs of our esteemed customers. MCB Contact Center is equipped with 200+ trained professionals who offer a wide array of financial and non-financial services in multiple regional languages around the clock. The customers are further facilitated through self-services modes like Interactive Voice Response (IVR), Self Service Channel and Live Chat for RDA customers. The Contact Center not only serves MCB Bank's customers but also cross-sells and up-sells a number of products and services as well.

WBG Products and Services

MCB Bank's Home Remittance brand, MCB Burqraftaar was amongst the largest remittance payout brands in Pakistan in 2020. Our contribution through MCB Burqraftaar is not only limited to the bank's growth but we are also proud to be one of the biggest contributors to the national interest of the country by promoting remittances through legal channels. MCB has managed to build an extensive network of partners across the globe to ensure overseas Pakistanis can send money to their families back home.

- **MCB Burqraftaar Cash (Cash in Hand)** is available via MCB's entire branch network. Payments can be made from any of MCB's 1,400+ branches to walk-in customers.
- **MCB Burqraftaar Transfer (Straight to Account)** enables overseas Pakistanis to send their remittances to their loved ones in Pakistan through our hassle-free straight to account credit service.
- **MCB Lite Remit** allows beneficiaries to receive money from their loved ones directly into their Mobile Wallet, from our global network of remittance partners. Money can be conveniently withdrawn from MCB Lite Card through MCB branches, MCB ATMs, 1Link ATMs, or

use the funds through the MCB Lite mobile App and POS terminals.

- **MCB Asaan Remittance Account** is targeted towards unbanked / under-banked Remittance Beneficiaries of Pakistan with simplified/relaxed account opening requirements and procedures.
- **MCB Burqraftaar Remittance Account** is targeted towards inward Remittance Beneficiaries in Pakistan with a unique feature of exemption on withholding tax on all cash withdrawal transactions of any amount via cheques from all MCB Bank branches & country-wide ATMs.

MCB Home Remittance is continuously working to design new products and facilities for the ease and convenience of remitters and beneficiaries.

MCB Transaction Banking: Transaction Banking (TBD) provides a wide range of value-added collection and payment services to large corporations through the Bank's vast network of real-time branches. TBD provides structured and customized collection products enabling customers to realize their sales proceeds swiftly from across the country, supported by real-time MIS reports.

Corporate Collection and Payment

- **CollectPlus:** Collection through a deposit of cash, same branch and local clearing instruments in designated MCB Branches
- **RemitPlus:** Collection through a confirmed and secured receipt, without the involvement of the clearing process
- **SIDA:** Collection through direct debit of dealer's account maintained at the branch.
- **DebitPlus:** Collection through direct debit of dealer's account maintained at the branch by TBD via a one-time instruction from the dealer/distributor and email instruction from the customer.
- **PayPlus:** Payment by IFT/IBFT/Bankers cheques/ RTGS by emailing the relevant file/instructions to TBD
- **TBD - FI Cash Management:** We offer unique cash management solutions to our local Correspondent Banks and Non-Bank Financial Institutions to meet their particular requirements for fund collection, payment, reconciliation, and reporting.
- **Online Fund Transfer (OFT):** This web-based electronic fund transfer facility has been designed to enable a large network of franchises/dealers/distributors to conduct real-time branchless transactions through a secured MCB website.
- **Dividend Warrant Management:** The Bank provides a complete and comprehensive dividend solution to customers through Electronic Transfers to Shareholders Accounts & processing of Warrants through MCB branches, followed by complete reconciliation. Foreign dividends are also managed end to end by TBD.
- **Sub Clearing Arrangement:** Microfinance banks that do not have operating licenses for clearing can now rely on MCB to act as their sub-clearing agent for processing transactions through NIFT.
- **Payment Station:** Corporate Payment Station "Pay Direct" offers a real-time comprehensive payment and transfer mechanism for corporate and large business entities. This allows our customers to virtually execute A2A, Cash Payments, Bankers Cheque, IBFT, RTGS, and Report Printing.

Our Corporate Banking team works in conjunction with Transaction Banking to facilitate customer requirements for collections, payments, dealer finance, electronic dividends, and bulk salary processing.

- **Corporate IVR for Collections:** MCB introduced a new platform for Dealers, Distributors, and Franchisees to conduct transactions without the need to visit MCB branches, by using our existing CRM and IVR channel. The product provides security in terms of cash-carrying and also allows the dealers/distributors to conduct transactions even after banking hours i.e. 24/7.

- **Tax & Duties Payments to FBR:** MCB Customers can pay their FBR Tax and Duties securely and conveniently through MCB Transaction Banking "Over-the-Counter" at any MCB branch as well as through "TBD PayPlus" by sending us their tax payment details on a secured file. Our Corporate customers can also pay their taxes and duties through "TBD-PayDirect" 24x7 and from anywhere in the world. Under FBR payments, MCB offers real-time integration with 1-Link and CBS with complete security and a two-step authorization process. Complete transaction details are ensured through computer-processed receipt, instant SMS alert, and detailed MIS for the branch / back office.

Banker to the Issue for IPO/SPO's & Right Shares: We provide efficient & effective processing for both IPO/ SPOs & Right Share Mandates. Our branches are well-trained in handling collections for both these products. The information mentioned in the forms is matched with funds collected and after reconciliation, these are transferred to the respective company. MCB's TBD team works jointly with Investment Banking Team on various IPO/SPO's & Rights Shares to facilitate clients.

MCB Corporate Banking Finance: MCB's Corporate Banking Finance is equipped with a professional and devoted relationship management team having a presence in 5 cities (namely Karachi, Multan, Faisalabad, Lahore, and Islamabad) across Pakistan is providing structured financial services through dedicated Corporate Centers/Branches.

The corporate Team operates in 3-dimensional approaches within the defined goals of the organization.

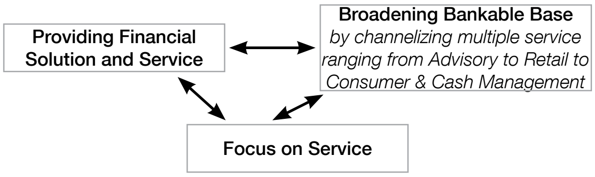

- **Financial Services and Solutions:** MCB Corporate Banking provides access to diversified financing options, including working capital loans, term loans, trade based finance services (bank guarantees, Import Letter of Credits, import and export services, bill discounting / negotiation, Export finance both in USD and PKR, State Bank of Pakistan export refinance and Long Term Finance, Import finance both in USD and PKR), dollar-based loans, financing under SBP schemes and depository options are also offered under various schemes.
- **Broadening Bankable Base:** Our Corporate Banking team works in close liaison with our Investment Banking team to facilitate clients with advisory and arrangement services for equity, debt and project finance offered through our Investment Banking Division. Similarly, our Corporate Banking team works in conjunction with Transaction Banking, Retail, and Consumer Team to facilitate their requirements for collections, payments, dealer finance, electronic dividends, bulk salary processing, and various consumer-related products for their employees and shareholders.
- **Focus on Service:** Dedicated Corporate Branches are available in 5 cities Karachi, Multan, Faisalabad, Lahore, and Islamabad to cater business needs of corporate relationships through the support of the Corporate Relationship Management Team.

MCB Investment Banking: MCB Investment Banking offers a full suite of Investment Banking services ranging from equity & advisory, syndications and debt capital markets to Project and structured finance. MCB Investment Banking works in close coordination with Corporate and Retail Banking to facilitate their clients with its services.

MCB Project and Structured Finance: MCB Project and Structured Finance is a 'Non-recourse' or 'Limited Recourse' financing, where the lenders base their credit decision primarily on the cash flows of the project, concerning repayment of the project debts. Risks are carefully allocated amongst various stakeholders.

MCB Syndicated Loans and Debt Capital Markets: MCB Syndicated Loans and Debt Capital Markets involve arrangement, underwriting and placement services for debt financing requirements by large corporate and institutional clients to other financial institutions or through the debt capital markets.

MCB Quasi Equity / Hybrid Instruments: MCB Quasi Equity/Hybrid Instruments are structured and tailor-made products incorporating specific customer requirements based on debt and/or equity components including unsecured nature instruments, subordinated nature types, cumulative/noncumulative dividend payments types, equity play component instruments, etc.

MCB Equity Capital Raising: MCB Equity Capital Raising relates to raising capital for our clients by offering common or preferred equity to the public or private investors, through initial public offers, offers for sale, rights issues and private equity placements and underwriting of equity issues in the Capital Market.

MCB Advisory Services: Financial and Capital Raising Advisory provides our clients with financial advisory services for mergers and acquisitions, privatization, project finance, commercial structuring support and access to capital resources to assist companies successfully finance their business/project.

MCB Trade Products

Trade Products provide a wide range of standard as well as tailor-made products and solutions to trade customers from all walks of life. Despite having an inventory of standard to market products in refined shape, specific new-to-market products include:

- **Quick-LC:** A simple and easy to use mechanism, internal design of desktop application, allowing customers to type-in, e-mail and print out an LC application form, reducing cost and hassle while improving TAT and input accuracy.
- **X-Flex**: A solution which facilitates external export business for customers in cases where transport documents (Bill of Lading) are not available at the counters of MCB's discounting / financing branch on the date of extending financing.
- **TRIMS:** Facilitates the financing of inland trade through the involvement of MCB Bank at both ends thereby allowing an exporter to get receivables discounted on non-recourse basis, i.e. the exporter receives payment in a given time (in less than 48 hours) after performing obligations.
- **Econ-LC:** A product program which allows drafts/bills of exchange to be waived as a requirement under LCs available by negotiation resulting in reduction in overall transaction cost for importers.
- **Avalization (Export):** A global product designed to facilitate the financing of export by allowing an Exporter (Seller) to discount the receivables under credit granted to a counter party, i.e. Importer (Buyer), without taking payment risk on the Importer (Buyer) under a contract (non-LC transactions).

- **Avalization (Import):** A product which facilitates delivery of import documents (and goods) against importer's acceptance under contracts (without opening of LC) hence offers savings associated with fees and commissions.
- **China LC Confirmation Programme:** Under this program the branches of MCB's partner bank located in Hong Kong and China add "Confirmation" to MCB LCs on "Free of Cost" basis thus increasing acceptability of MCB's Import LCs and facilitate import customers.
- **Europe LC Routing Program:** Under this program the branches of MCB's partner bank located in UK will be handling LC transaction originated by MCB under special arrangements which includes advising, confirmations, bill discounting and UPAS transactions.

MCB Sri Lanka - Products and Services

Current Account - Take account of things that matter!
MCB Bank Current account allows our customers to distinguish themselves in the financial market with a secure and faster cheque clearing process, allowing them to carry out their transactions with a greater level of confidence.

Privilege Current Account – Feel prioritized and privileged!
MCB Bank Privilege Current Account offers a range of personal and business banking solutions that are specially tailored to meet our customers' emerging needs, with a whole array of value additions coupled with our unparalleled customer services.

Savings Account - The smarter your savings, the higher your returns!
MCB Savings Account is designed to encourage customers to maintain a healthy account balance, which keeps growing day by day. Tier based interest rates enable customers to enjoy an attractive return on their funds. Customers have the freedom to utilize their funds to meet their day to day financial needs with no restrictions to the number of transactions they perform.

Fixed Deposits -The safe way to save!
Fixed Deposit Accounts are designed to suit the diversified investment requirements of our customers. Starting from 7 Days Call Deposits, our product range extends to Term Deposits that can be fixed up to 4 Years. Further, we offer the option of obtaining the interest at maturity or specific interim periods (monthly/quarterly) depending on the customer's requirement for funds.

MCB Kidz Club - Pave the future for your child!
Children are the wealth of our nation. We offer ample encouragement and incentives to assure them of a secure future. MCB Kidz Club Account offers an attractive interest rate coupled with a wonderful gift scheme based on the account balance. This encourages parents and children to grow their account balance to enjoy the financial return, as well as to collect gift items on reaching specific account balances.

Foreign Currency Accounts - When you need financial diversity!
MCB customers can save in any designated foreign currency and enjoy attractive returns. Further, we offer a wide range of account types for personal and business clients under special schemes approved by the Department of Foreign Exchange of the Central Bank of Sri Lanka.

MCB Debit Card – Introducing more convenience!
MCB Bank has been providing financial services to its valued customers since 1994. To further enhance this relationship, MCB signed up with the partnership of Lanka Pay and JCB for the introduction of Debit Card, providing our valuable customers the facility to shop with convenience. Debit Card also provides access to customer accounts through a shared network of over 4,500 ATMs Island wide.

Virtual Banking – Smart & secure access 24/7!
Virtual Banking helps customers stay updated on their account activities from wherever they are, and carry out their banking transactions at a time that is convenient for them, instead of restricting themselves to standard banking hours.

Safe Deposit Lockers - Where safety is a promise!
We pride ourselves in offering our customers ease of mind and this is yet another service that highlights our commitment to providing everything necessary to accommodate their needs. We offer various types of lockers depending on customer requirements to protect their documents, jewelry, or any other valuables.

Home Remittance – Transferring happiness to your Homeland!
MCB Home Remittance is a simple and dependable way of sending money to your loved ones in Sri Lanka from anywhere in the world. With a wide network of worldwide remittance partners (including the partners of MCB Bank Pakistan), we ensure the fastest and safest money transfer.

Trade Services – Trading becomes convenient & faster!
MCB Trade Services empower individuals and businesses to reach their highest potential by streamlining and customizing processes and product portfolio. We ensure that a comprehensive range of trade products and services will enable you to do your business successfully. A new product "Avalization Imports" was launched in 2020 facilitating trade transactions under Collection terms.

SME & Corporate Banking
We offer diversified finance options for Small/Medium Entrepreneurs and Corporate customers to achieve success in their business. These financing options range from Overdrafts, Leasing for vehicle and machinery,

Working Capital Loans, Term Loans, Trade Finance, and Structured Project Financing to cater to the evolving needs of our Business Banking customers. A wide range of Treasury products including Forward Contracts, Repurchase Agreements, Spot Contracts, and Treasury Bills are also offered under investment options.

UAE - Products and Services

MCB UAE has an array of customized business products for its wholesale customers that include Business Accounts, Finance & Trade products. These services are aimed at fulfilling the day to day needs of the businesses at competitive yet flexible prices.

Avalization

The "Avalization (Export)" Product has been designed to facilitate the financing of exports by allowing an Exporter (Seller) to discount its receivables under credit granted to a counterparty i.e. Importer (Buyer), without taking payment risk on the Importer (Buyer); through the involvement of banks at both ends, the Exporter (Seller) receives its payment a few days after performing its obligations under a contract (and not under a letter of credit) entered into with the Importer (Buyer) instead of waiting for the full tenor of the credit period granted to the Importer (Buyer). The receivables are secured by a bill of exchange or promissory note accepted by the obligor (the person who is liable for the payment of the receivable). By way of credit enhancement, these receivables are further backed by the guarantee or commitment of the obligor's bank who becomes the end Obligor.

MCB Current Deposit Account

It is designed to provide our wholesale customers with transactional convenience and flexibility. Choices of local (AED) and International currencies (USD/GBP/EUR) are available at attractive options for our business customers with a low minimum balance.

365 Savings Business Gold Account

Transactional flexibility in a Savings account, 365 days Gold Account provides the choice of local (AED) and International currencies (USD/GBP/EUR). This account will grow savings at an attractive interest rate for 365 days while giving the flexibility to use these funds for business transactional needs as well. An attractive option for business customers with a high balance and with requirements of transactional flexibility.

Term Deposit

Term Deposit products are available in a choice of local (AED) and International currencies (USD/GBP/EUR). Tenor options can be chosen between 1, 3, 6, and 12 months. It is an attractive option for our business customers with short to mid-term investment opportunities.

Demand Finance

It is financing for a fixed period repayable either in periodic installments or in a lump sum, at a future date. An attractive option for business customers that require financing against fixed assets such as Plant and Machinery, Land, Building, etc.

Overdraft

Overdraft facility allows businesses to access additional funds for day to day business expenses. The overdraft facility will charge interest only on the daily outstanding balance from the Current Account above the credit amount available.

Bills Discounting

Our discounting solution is for clients who are looking to fund their working capital requirements. While discounting, the bank buys the bill (i.e. bill of exchange or promissory note) before it is due. The transaction is practically an advance against the security of the bill and the discount represents the interest on the bill from the date of purchase of the bill until it is due for payment.

Post-dated Cheque Discount Facility

Cheque Purchase Discount Facility from MCB Bank takes away the hassle of waiting for post-dated cheques (30-120 days) to clear while letting businesses concentrate on completing their transactions.

Finance against Trust Receipts (FATR)

Financing against Trust Receipts enables our customers to honor payments to their suppliers by letting the bank pay on their behalf without reducing customer's credibility. The customer simply has to submit a Trust Receipt (TR) Letter which contains a statement of receiving goods on the Bank's behalf and promising to pay the Bank on a deferred basis.

Financing against Receivables

Financing against Receivables is available in the form of Invoice discounting. Invoice Discounting is a form of asset-based finance that enables a business with evidence to release cash tied up in an invoice to its debtors/buyers.

- **Discounting of Export Bills for Collection** – A borrowing mechanism available to raise finances for an agreed specific tenor. Where the bank buys the export bill at a discounted price, the exporter gets the amount from the bank while submitting export documents.
- **Discounting of Bills under Export LC** - A borrowing mechanism available to raise finances against documents drawn on buyers for an agreed specific tenor. Discounting of Bills under Export LC is available for customers who wish to sell such bills to the bank at a discount rate under the condition of not claiming anything from customers (Without Recourse).

Guarantees

The Bank stands as a guarantor for its client ensuring that the liabilities of a debtor will be met. Generally, a Guarantee is issued on cash collateral or against some security as collateral.

Letter of Credits
A bank guaranteeing on behalf of its customer that a buyer's payment to a seller will be received on time and for the correct amount. Generally, a Letter of Credit is issued for Sight and Usance terms against some security/collateral.

Internet Banking
MCB Bank has implemented Oracle's Flex Cube Direct Banking which comes with enhanced securities, a user-friendly interface, and a host of functionalities/services. Our state of the art Internet Banking allows clients, through a maker checker concept, the convenience of conducting Inter-Bank Funds Transfer as well as Cross Border Funds Transfer at anytime from anywhere in the world simply by logging on to www.online.mcbae.com

Wages Protected System
MCB Bank has offered Wages Protected system (WPS) in MCB UAE, that enables our customers to route their salary transactions for their employees by using MCB's services.

Bahrain – Products and Services

MCB-Bahrain offers the following Products and services to its clients:

MCB Current Account
MCB Bahrain's current account is structured to provide our customers with transactional convenience and flexibility. Accounts are opened in international currencies (USD/GBP/EUR) with a low minimum balance.

MCB Savings Account
MCB Bahrain offers saving accounts in International currencies (USD/GBP/EUR) to customers. It offers an attractive interest rate on deposits while giving them the flexibility to use the funds for transactional needs. An attractive option for customers with a high balance and require transactional flexibility.

Term Deposit
Term Deposits are available in international currencies (USD/GBP/EUR). Customers can choose tenor based on their needs. It is an attractive option for customers with short to mid-term investment opportunities.

Syndicated Transactions
MCB Bahrain can participate in both Islamic and conventional syndicated transactions. The branch participates in various regional transactions for corporates, financial institutions, and sovereign entities. Moreover, the bank is engaged in risk-sharing transactions with other reputed banks in the region for confirmation/discounting of trade instruments.

Loans and Advances
MCB-Bahrain provides loans and trade facilities to its bilateral clients. MCB Bahrain financed for short term or long term funded facilities to its clients. Also, the branch can structure project-specific financing for its clients in the region.

Trade Finance
MCB-Bahrain provides all types of funded & non-funded trade finance facilities to its clients including all types of letters of Credits, advising, confirmation, discounting of credit, documentary collection, bill discounting, and issuance of bank guarantees.

Treasury
MCB Bahrain has been an active treasury that has been investing in various fixed income securities. The branch can invest in both Islamic and conventional instruments. Our portfolio in different points in time traded sovereign euro bonds, International Sukuk and Treasury bills. Bahrain branch can provide plain-vanilla FX solutions to its trade clients. Quote for other liquidity instruments such as SWAPS, short term borrowing/placements, and forwards can be inquired from the treasury front office.

MCB Islamic Bank Limited

Products and Services:
MCB Islamic Bank Ltd. offers a wide array of Halal and RIBA free Islamic Banking products and services to meet the requirements of individuals and businesses alike.

Deposit Products:
MCB Islamic Bank offers a wide variety of Saving, Current and Term Deposit accounts such as the following:

Current	Saving	Term Deposits
MIB Hidayat Current Account	MIB Imaan Saving Account	MIB Na'mat Term Deposit
MIB Basic Banking Account	MIB Barkat Saving Account	MIB Na'mat Plus Term Deposit
MIB Asaan Current Account	MIB Barkat Saving Plus Account	MIB Na'mat Premium Plus Term Deposit
MIB Hidayat Foreign Currency Current Account	MIB Barkat Saving Premium Account	MIB Aasoodgi Term Deposit
	MIB Barkat Super Saving Account	MIB Financial Institutions Term Deposit
	MIB Asaan Saving Account	MIB Na'mat Plus Foreign Currency Term Deposit
	MIB Atfaal Saving Account	
	MIB Imaan Foreign Currency Saving Account	

Digital Banking:

In order to meet growing needs of customers, MCB Islamic Bank offers the following Digital Banking products and services:

MIB Qadar Cards	MIB Phone Banking
MIB Internet Banking	MIB Mobile App
SMS Notification Service	MIB ATMs network
MIB Corporate Internet Banking	

Consumer Finance:

MCB Islamic Bank offers Halal and RIBA Free Housing Finance (Rihayesh Finance) & Car Finance (MiCar) products to provide Shari'ah compliant housing and vehicle financing solutions to Individuals.

In order to enable middle and low-income segments of the population to meet their housing needs, MCB Islamic Bank Limited is also offering Shari'ah compliant low and medium cost housing finance options to our customers at affordable rates under State Bank of Pakistan's Profit Rate Subsidy Scheme for Housing Finance 'Mera Pakistan – Mera Ghar'.

Microfinance:

For fulfilling the needs of growing small / micro businesses in both Urban and Rural areas of Pakistan, MCB Islamic Bank Limited offers Murabaha Microfinance (Sharai Karobar) for men and women operating small businesses in diverse sectors. Through this financing facility, they can avail financing of up to PKR 500,000.

SME Banking:

MCB Islamic Bank offers Shari'ah Compliant financing solutions for Small and Medium sized enterprises. These products have been designed to cater to the specific needs of SME industry. These products include Murabaha, Musawamah, Ijarah, Diminishing Musharakah, Istisna, Finished Goods Financing, and Commodity Salam.

MCB Islamic Bank offers following SME Banking services:

- Short Term / Working Capital Financing
- Medium / Long Term Financing
- Trade Finance
- Bank Guarantee
- Risk participation arrangement with financial partners
- SBP refinance schemes
- 'Prime Minister's Kamyab Jawan Youth Entrepreneurship Scheme' to enable youth to avail affordable financing for establishing new business or strengthening an existing one
- Awareness sessions for SME Customers on financial and non-financial advisory services with SMEDA, Chamber of commerce, and trade associations.

Commercial Banking:

Commercial Financing segment focuses on building strong and long-lasting relationships with the Bank's customers by delivering satisfactory Shari'ah compliant solutions. To deliver excellent services, commercial financing segment facilitates its valued customers by synergizing its specialized team's effort with bank's outreach across the country for increased product servicing range for branch customers.

Commercial financing business offers following range of products to customers:

- Trade based financing (funded & non-funded).
- Cash Management & Remittance services.
- SBP's Refinance schemes.
- Working Capital financing
- Medium & Long term financing.
- Letter of Guarantees.

Corporate Banking:

MCB Islamic Bank Corporate Banking team is focused on providing a range of diverse financial services (including tailor made solutions) to corporate clients (including multinational and public entities) by partnering with them and building long-term sustainable relationships.

Our dedicated teams within Corporate Banking are situated in offices located in Karachi, Lahore, Islamabad, Multan and Faisalabad. Our in-house expertise of product specialists and Shari'ah scholars are well versed in providing Shari'ah-compliant financing solutions to meet our customer's financing, trade, foreign exchange, investment banking and various other business requirements in a hassle free, effective and efficient manner across a diverse range of industries.

Our Corporate Banking relationship teams in liaison with Product Management & Shari'ah Structuring, Treasury, Cash Management, Trade, Investment Banking and Consumer Banking departments develop and deliver offerings that are used across diverse businesses.

MCB Islamic Bank offers a complete range / array of Shari'ah compliant products for both Corporate and Commercial banking sectors which can be classified as follow:

- **Working Capital Finance**
  A complete product suite for extending Shari'ah Compliant products for working Capital Finance including Murabaha, Musawamah, Istisna, Commodity Salam, Finished Goods Financing and Musharakah Running Finance for catering to balance sheet (working capital) needs of Corporates / Commercials / MNCs for managing their day-to-day funding needs.

- **Medium & Long-Term Finance**
  For catering to Balance Sheet requirements under long-term financing, MCB Islamic Bank has an array of medium to long term financing products which include Ijarah and Diminishing Musharakah.
- **Trade Services**
  MCB Islamic Bank also offers a range of import and export services to effectively manage local and global supply chain needs of our customers, which provides them a competitive edge. These facilities extend for both raw materials and fixed assets. Services that are offered under the umbrella of Trade Finance includes:

o Export Services
o Bills for Collection under Export
o Letter of Credit Advising & Confirmation services
o Currency Salam (Shari'ah compliant alternate for Export Bill Discounting)
o Pre-Shipment and Post-Shipment financing on Islamic modes of financing
o Import Services
o Letter of Credit
o Import Financing
o Bank Guarantees

SBP Financing / Refinancing Schemes:
State Bank of Pakistan (SBP) offers various Financing / Refinancing facilities to support industrial growth and exports with the aim to promote the overall economic development of the country. These schemes provide financing to targeted industries at subsidized rates for increasing their production capacity and meeting their working capital requirements. Moreover, SBP also introduced certain schemes to support the industry with regards to the challenges being faced in post-pandemic scenario. Accordingly, the Schemes available through MCB Islamic Bank are:

- Islamic Export Refinance Scheme (IERS)
- Islamic Long Term Financing Facility (ILTFF)
- Islamic Refinance Scheme for Working Capital Financing of Small Enterprises and Low-End Medium Enterprises (IWCF)
- Islamic Financing Facility for Storage of Agricultural Produce (IFFSAP)
- Islamic Refinance Facility for Modernization of SMEs (IRFMS)
- Islamic Financing Facility for Renewable Energy (IFRE)
- Islamic Temporary Economic Refinance Facility (ITERF)
- Islamic Refinance Facility for Combating COVID-19 (IRFCC)
- Islamic Refinance Scheme for Payment of Wages and Salaries

Cash Management Services:
MCB Islamic Bank has a significantly enhanced capability of receiving collections on behalf of Bank's customers, whereby we can support diverse requirements of our clients for receiving payments from their payers throughout the bank's branch network. This product encompasses the entire paper-based accounts receivable function.

Other Services:
MCB Islamic Bank Ltd. also offers various services to meet growing needs of its customers, some of which are as follows:

- Remittance Service
- Forex Services
- PRISM Services

Corporate **Information**

**Board of Directors**	Mian Mohammad Mansha	Chairman
	Mr. S. M. Muneer	Vice-Chairman
	Mr. Muhammad Tariq Rafi	Director
	Mian Umer Mansha	Director
	Mrs. Iqraa Hassan Mansha	Director
	Mr. Muhammad Ali Zeb	Director
	Mr. Mohd Suhail Amar Suresh bin Abdullah	Director
	Mr. Yahya Saleem	Director
	Mr. Salman Khalid Butt	Director
	Mr. Masood Ahmed Puri	Director
	Mr. Shahzad Hussain	Director
	Mr. Shariffuddin Bin Khalid	Director
	Mr. Imran Maqbool	President & CEO
**Audit Committee:**	Mr. Shahzad Hussain	Chairman
	Mian Umer Mansha	Member
	Mr. Muhammad Ali Zeb	Member
	Mr. Shariffuddin Bin Khalid	Member
**Chief Financial Officer:**	Mr. Hammad Khalid	
**Company Secretary:**	Mr. Fida Ali Mirza	
**Auditors:**	M/s. KPMG Taseer Hadi & Co.<br>Chartered Accountants	
**Legal Advisors:**	M/s. Khalid Anwer & Co.<br>Advocates & Legal Consultants	
**Registered /Principal Office:**	MCB Building, 15-Main Gulberg,<br>Jail Road, Lahore, Pakistan.	
**Contact us:**	UAN: + 92 42 111 000 622<br>E-mail: investor.relations@mcb.com.pk<br>Visit us: www.mcb.com.pk	
**Registrar's and Share Registration Office(s):**	**Head Office:**<br>M/s. THK Associates (Pvt.) Limited<br>Plot No. 32-C, Jami Commercial Street 2,<br>D.H.A., Phase VII, Karachi, Pakistan.<br><br>**Branch Office:**<br>M/s. THK Associates (Pvt.) Limited<br>Siddique Trade Centre,<br>Office No. PL-29, PL Floor,<br>72 Main Boulevard Gulberg -2,<br>Lahore, Pakistan.	

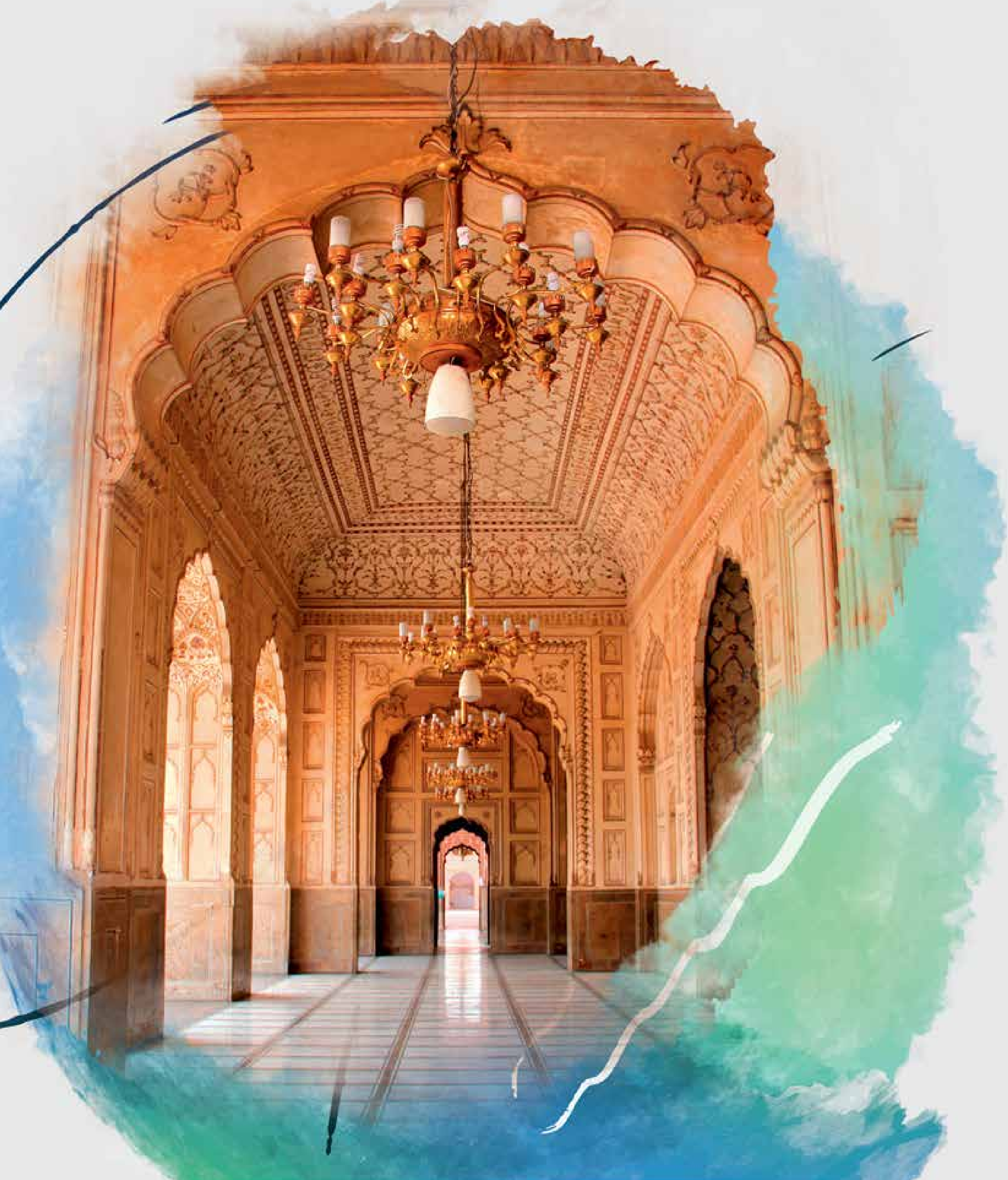

Board of **Directors**

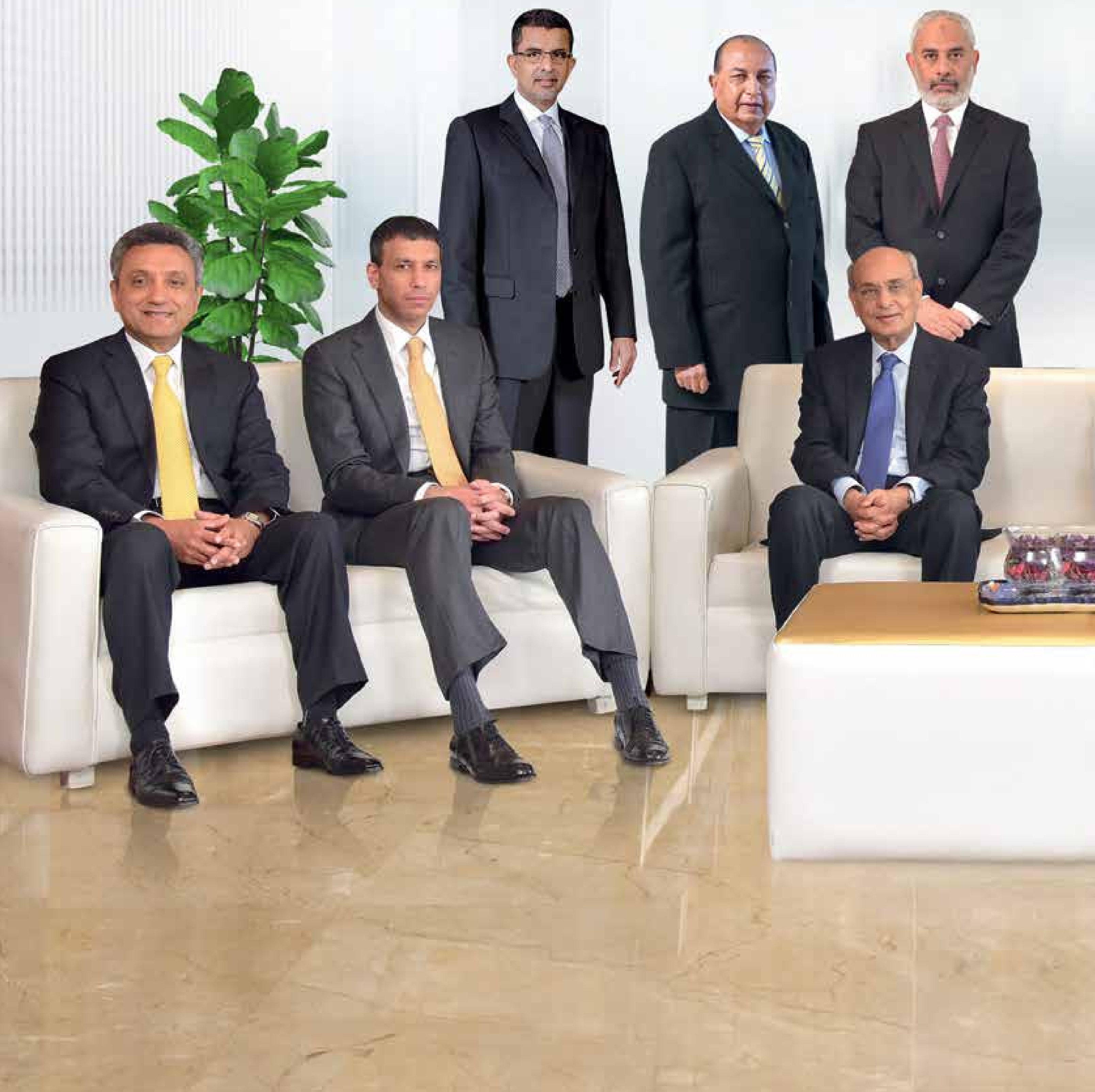

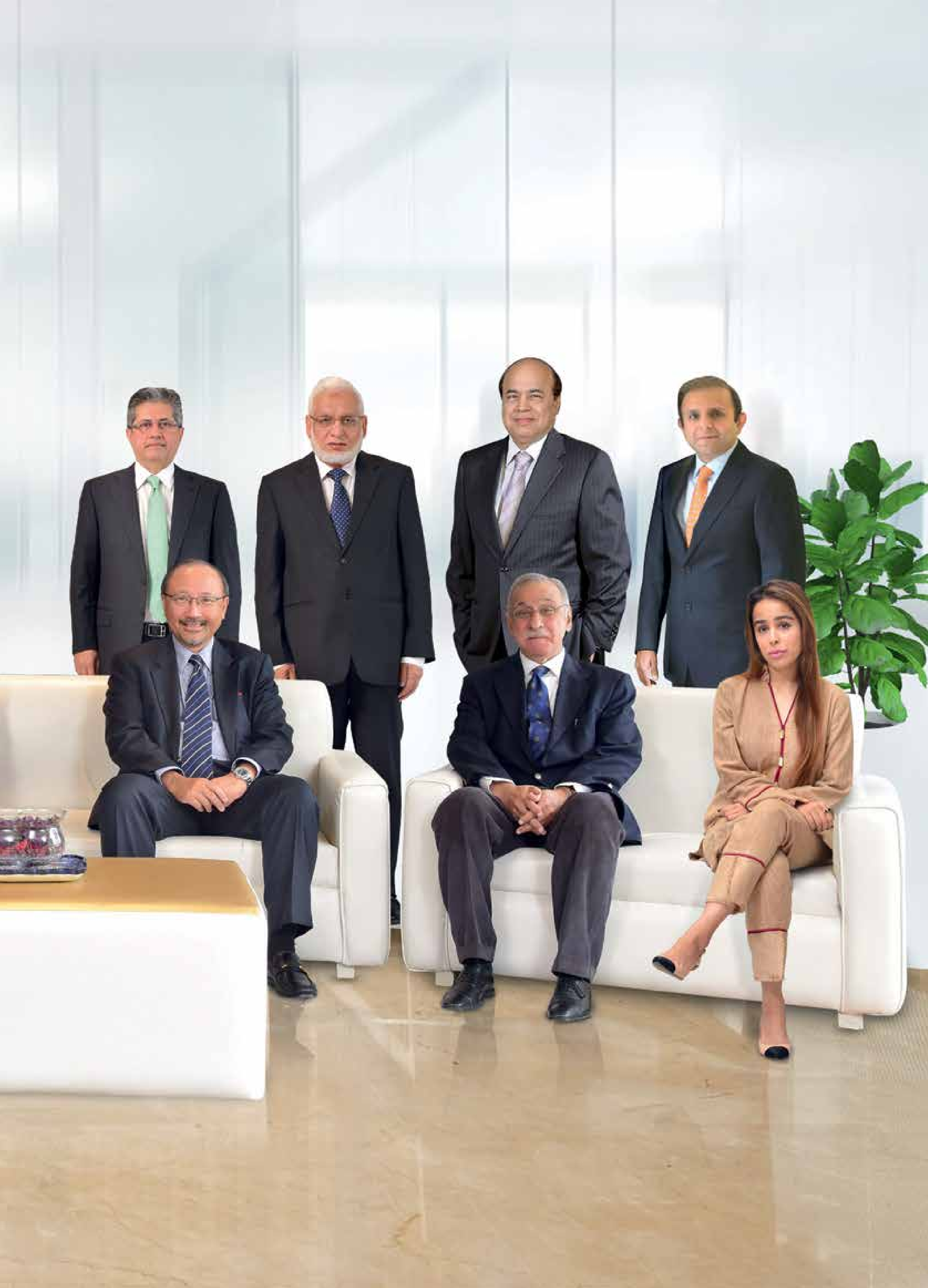

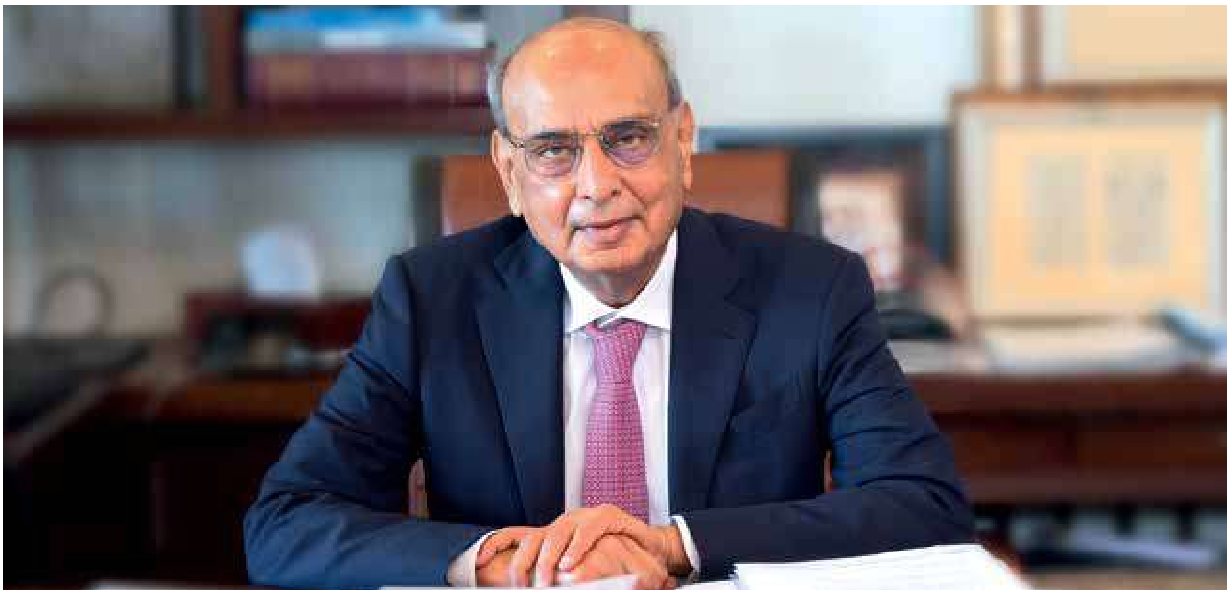

Mian Mohammad Mansha
Chairman

Mian Mohammad Mansha started his career at the age of 24 as the CEO for Nishat Mills Limited. At present, the business group is one of the leading and most diversified in South East Asia, having presence in Textile, Cement, Insurance, Banking, Financial Services, Power Generation, Hotel & Hospitality, Dairy, Paper Products, Retail Commerce, Real Estate, Agriculture, Aviation and Automotive sectors. It operates in various countries across the globe including Sri Lanka, Azerbaijan, UAE, USA, Hong Kong and Bahrain. The Nishat Group is one of the largest private sector employers, exporters and tax contributors in Pakistan.

Mr. Mansha has served as Chairman of MCB Bank, after its privatization, from 1991 to mid-1995 and then from 1997 till date. Presently, he is also a member of the Board's Human Resource & Remuneration Committee, Committee on Physical Planning & Contingency Arrangements and Business Strategy & Development Committee at MCB Bank Limited. Previously, he was associated with Punjab Mineral Company (Pvt) Limited, Civil Aviation Authority, Pakistan International Airlines, Board of Investment, Punjab Board of Investment & Trade, Pakistan Industrial Development Corporation (Pvt) Limited, Commonwealth Business Council UK, Int'l Advisory Board Babson College USA, National Management Foundation, Textile College Faisalabad and Government College of Faisalabad. He has also served as the Chairman for All Pakistan Textile Mills Association (APTMA) and APTMA, Punjab.

Mr. Mansha is also a committed philanthropist and provides regular support to numerous Pakistani charities and social causes. He has made significant contributions to Saleem Memorial Trust Hospital and to the Children's Hospital, Lahore for the upgrade of their pediatric cardiac surgery facilities. In the past he has lent personal support to Shaukat Khanum Memorial Trust, Lahore Hospital Welfare Society, Mind Organization, Human Rights Society of Pakistan, Kidney Care Centre, The Citizens Foundation, Friends of Pakistan Institute of Cardiology Trust, Care Foundation, SOS Village, Lahore Model School Sant Nagar, Aziz Jahan Trust for the Blind and Altaf Mehmood Foundation among many others.

He was presented with Pakistan's Civil Award, the Sitara-e-Imtiaz, for his contributions to industrial development, in 2004.

Other Directorships:

- MCB Non-Bank Credit Organization, CJSC, Azerbaijan.

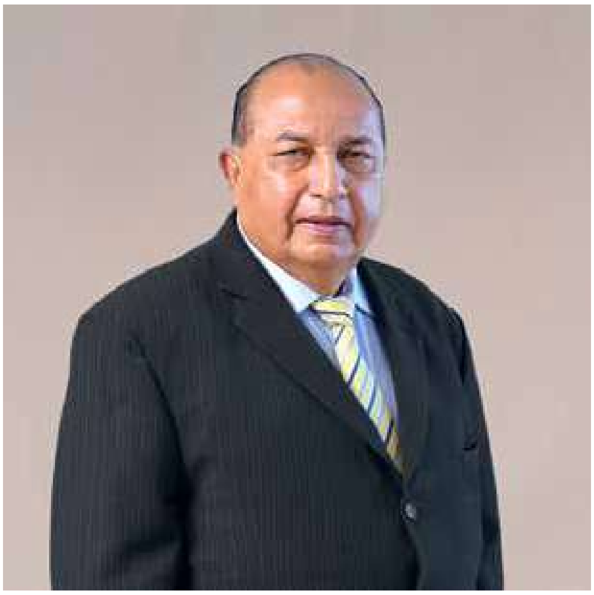

S. M. Muneer
Vice Chairman

With experience in sectors ranging from tanneries, textiles and banking, Mr. Muneer is a consummate industrialist and a certified director. He has been awarded with The Best Export Performance trophy by the Federation of Pakistan Chamber of Commerce & Industry (FPCCI), the Gold Medallion Award by the International Export Association, UK and the Best Businessman of the Year Award by Federation of Pakistan Chamber of Commerce & Industry (FPCCI). He is also a former President of FPCCI.

In addition, he has received the 'Sitara-e-Isaar' and the 'Sitara-e-Imtiaz' in 2006 and 2007 respectively by the President of Pakistan in recognition of his outstanding public services for the cause of humanity. His contributions and achievements go beyond the economic sphere and include education as well as health-care sectors. He was awarded an Honorary Ph.D. degree (doctorate of philosophy) in January, 2009 by the Governor of Sindh. He has also been appointed as a member of the Advisory Board of Citizen Police Laison Committee (CPLC) by the Honourable Governor Sindh.

Mr. S. M. Muneer also serves as member of Board of various educational institutions, such as Institute of Business Management and Greenwich University, Karachi, Din College, Chiniot, College of Business Management, Karachi and Institute of Behavioral Sciences (IBS), Karachi and Professional Education Foundation. He is also a member of Advisory Council of Institute of Business Administration (IBA).

He is on the Board of Shaukat Khannum Memorial Trust, Lahore, The Kidney Centre Post Graduate Training Institute and Fatimid Foundation. He is Patron-in-Chief, Korangi Association of Trade and Industry (KATI), Kashif Iqbal Thalassemia Care Centre, Trust (KITCC). Mr. Muneer is the Chairman of Chiniot Anjuman Islamia, running many hospitals, maternity homes, schools and colleges in Karachi, Faisalabad and Chiniot. He is also the Chairman of Husain Foundation (a not-for-profit organization working for improvement of cricket in Pakistan).

Mr. S. M. Muneer was awarded "Life Time Achievement Award" by the President of Pakistan, in 2012, and was also awarded Life Time Achievement Award in the same year in the City of Markham (Canada) by its Mayor Mr. Frank Scarpitti in the presence of members of the Parliament. He has also been awarded Who's Who recognition of Achievement Award in the field of Trade Politics by the National Council of Who's Who Pakistan in Karachi on December 29, 2018.

Other Directorships:

- Din Textile Mills Limited;
- Din Leather (Pvt) Limited;
- Din Farm Products (Pvt) Limited;
- Din Industries Limited;
- Arabian Sea Country Club

MCB
Bank for Life

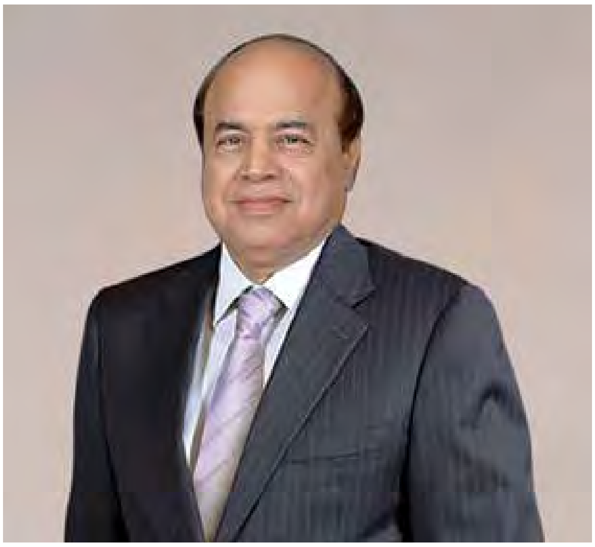

Muhammad Tariq Rafi
Director

Mr. Tariq Rafi is the Chairman of Siddiqsons Group and is a recipient of the coveted Civil Award Sitara-e-Imtiaz. He was awarded the prestigious Best Businessman award for the year 1999 and 2012, best Export Trophies between years 1980 to 2005. Mr. Tariq Rafi has been awarded the Privilege Card by the Prime Minister of Islamic Republic of Pakistan for being one of the top tax payers. He is also the Honorary Consul General of Republic of Serbia.

He is on the board of MCB Bank since its privatization and presently is the member of Board's Write-off & Waiver Committee.

Other Directorships:

- Siddiqsons Limited;
- Siddiqsons Tin Plate Limited;
- Central Depository Co. of Pakistan Limited;
- Siddiqsons Energy Limited.

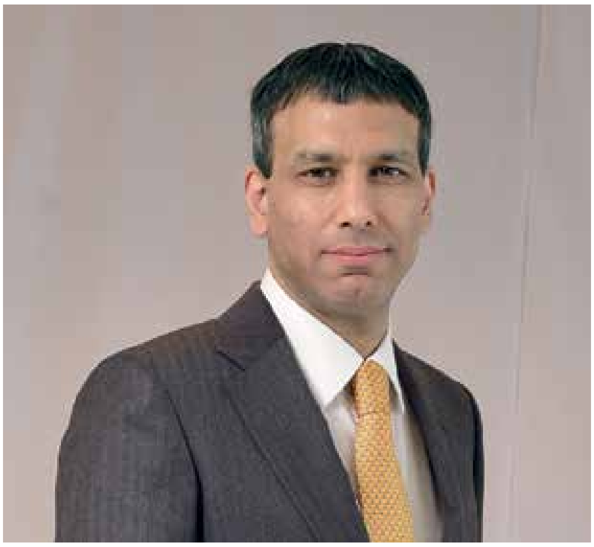

Mian Umer Mansha
Director

Mian Umer Mansha was co-opted as a Director on the Board of MCB Bank in November 1997 and served till September 2007. Then he was elected as a Director in the Bank's 61st AGM held on March 27, 2009.

Presently, at MCB Bank he is the Chairman of the Board's Business Strategy & Development Committee, Information Technology Committee, Committee on Physical Planning & Contingency Arrangements and Write-off & Waiver Committee and is also a member of Audit Committee and Risk Management & Portfolio Review Committee.

In addition, he has been serving on the board of various other businesses. Mr. Umer received his Bachelors degree from Babson College, Boston, USA.

Other Directorships:

- Nishat Mills Limited;
- Adamjee Insurance Company Limited;
- Adamjee Life Assurance Company Limited;
- Nishat Hotels & Properties Limited;
- Nishat Developers (Pvt) Limited;
- Nishat Dairy (Pvt) Limited;
- Nishat (Raiwind) Hotels and Properties Limited;
- Nishat Agriculture Farming (Pvt) Limited;
- Hyundai Nishat Motor (Pvt) Limited;
- Nishat Agrotech Farms (Pvt) Limited;
- Nishat Sutas Dairy Limited.

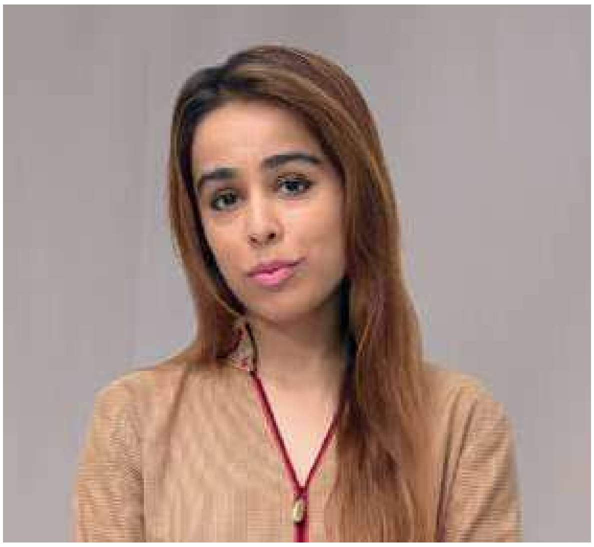

Iqraa Hassan Mansha
Director

Mrs. Iqraa Hassan Mansha has more than 9 years diversified professional experience in Hotel Industry. She received her B.Sc. Degree in International Politics from London School of Economics and M.Sc. degree in International Relations from the University of London School of Oriental and African Studies (SOAS).

She is serving in the capacity of Executive Director of Nishat Hotels and Properties Limited. She is also acting as Director on the Board of the following companies:

Other Directorships:

- Nishat (Raiwind) Hotels and Properties Limited;
- Nishat Real Estate Development Company (Pvt) Limited;
- Emporium Properties (Pvt) Limited.

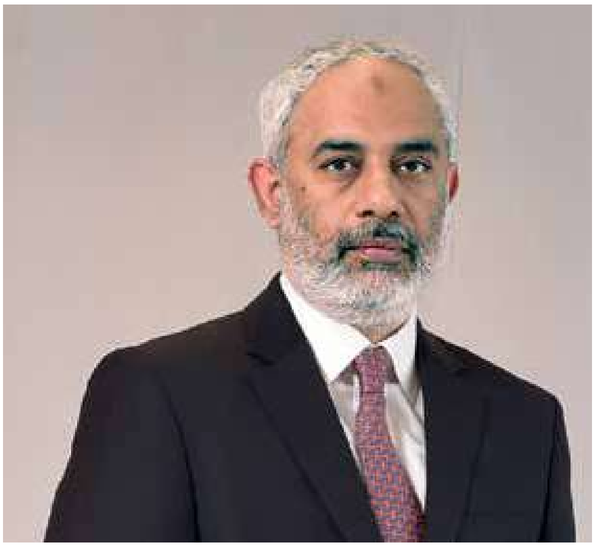

Muhammad Ali Zeb
Director

Mr. Zeb is currently the CEO and Managing Director of Adamjee Insurance Company Limited. He is a fellow member of the Institute of Chartered Accountants of Pakistan and has over 22 years of diversified professional experience in the fields of Finance, Insurance & Manufacturing. He also served as council member of Insurance Association of Pakistan and Pakistan Insurance Institute. Insurance Association of Pakistan elected him as the Chairman for the year 2014 in terms of its Constitution.

He was co-opted as a Director on the Board of MCB Bank in June 2013. At MCB Bank, presently he is the Chairman of the Board's Risk Management & Portfolio Review Committee and a member of Board's Audit Committee, Human Resource & Remuneration Committee, Compliance Review & Monitoring Committee, Committee on Physical Planning & Contingency Arrangements and Write-Off & Waiver Committee.

Other Directorships:

- Adamjee Insurance Company Limited;
- Adamjee Life Assurance Company Limited;
- Nishat Sutas Dairy Limited.

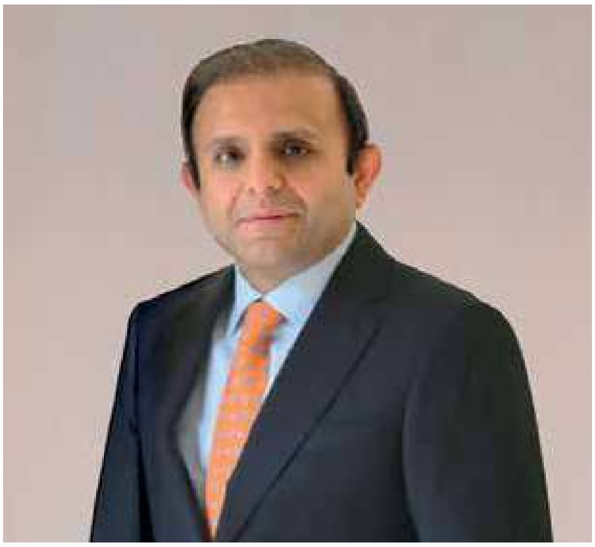

Yahya Saleem
Director

After graduating from the Lahore University of Management Sciences (LUMS), Mr. Yahya joined the family business as director of the Nishat Chunian Group with setting up a spinning mill in 1990. The company has since diversified into weaving, home textile, power generation and entertainment sectors. Today with a turnover of PKR 39.337 billion, NCL ranks amongst the top 5 textile companies in Pakistan.

The group ventured into the power business in 2007 with a 200 MW Independent Power Plant (IPP), Nishat Chunian Power Ltd. With a turnover of PKR 15.021 billion in 2019, the IPP provides electricity to the national grid. Both the companies are listed on Pakistan Stock Exchange.

Together with his family, he has set up a Trust which is in memory of Sheikh Mohammad Saleem and has initiated setting up of a 200 bed tertiary care not-for-profit hospital in Lahore. The hospital will be built to state of the art international standards and will provide subsidized health care to the under privileged section of the city.

In 2015 he started a company by the name of NC Trading USA that is a Cotton trading company based in USA and actively sells US cotton in Pakistan. In 2017 NC Trading USA sold cotton worth USD 50 million to the leading textile mills in Pakistan.

In 2019, he was appointed as CEO and Director of NC Entertainment Private Limited which owns two Multiplexes, widely known as “Universal Cinemas”, including largest multiplex in Pakistan.

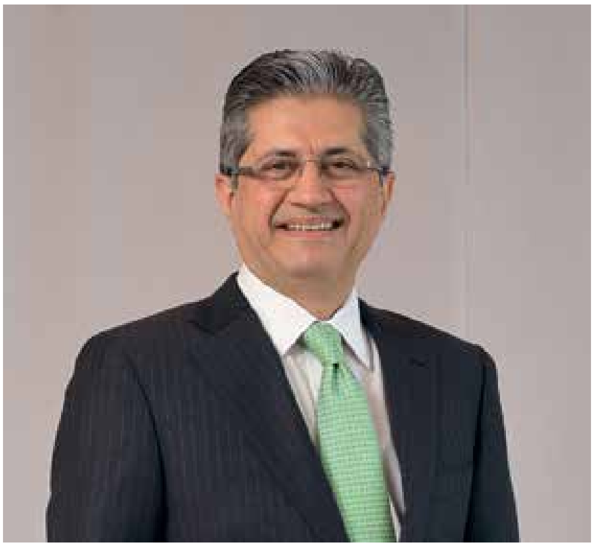

Salman Khalid Butt
Director

Mr. Salman Butt is an accomplished international business executive and ex-banker. He is currently a Dubai, U.A.E. based Entrepreneur.

Mr. Salman Butt started his career with Citibank N.A. in 1985 and worked for 20 years as an international banker with Citibank N.A. and Samba Financial Group, holding several senior positions in Corporate and Investment Banking in Pakistan, Hong Kong, UK, Egypt and Saudi Arabia.

Mr. Salman Butt joined Orascom Construction Industries, Egypt (OCI Egypt) as Group CFO in 2005. OCI Egypt was a leading Egyptian multinational listed on Cairo Stock Exchange and involved in Construction, Fertilizers and Cement operations. In 2013, OCI Egypt was re-domiciled as OCI N.V. Netherlands and listed on Amsterdam Stock Exchange, where he continued as Executive Director and Group CFO from 2013 to 2017.

Mr. Salman Butt has a Masters of Business Administration degree from the University of Texas at Austin, Texas, U.S. and a B.Sc. Industrial Engineering degree from Middle East Technical University, Ankara, Turkey.

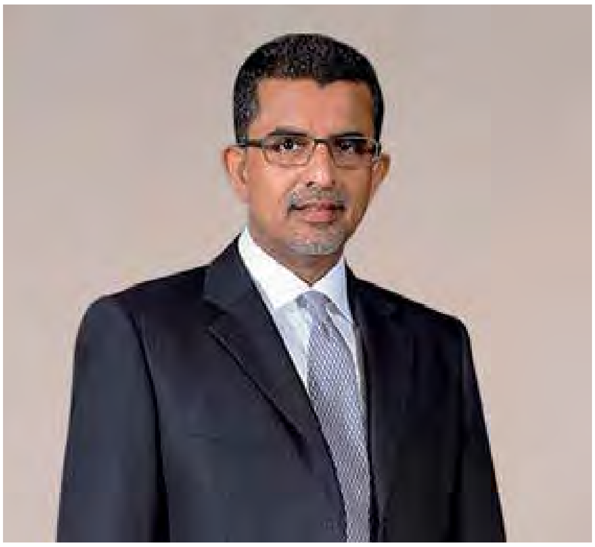

Mohd Suhail Amar Suresh Bin Abdullah
Director

Mr. Suhail has over 30 years of combined global experience in the telecommunications and financial services sector specialising in IT architecture, systems and application development, regional implementation and business development. His passion in technology drives him to effectively utilise technology as a competitive advantage for enhanced customer experience.

Mr. Suhail is currently the Group Chief Technology Officer for Maybank, Malaysia's largest Financial Services Institution. His previously held positions include Managing Director of Bank Negara Malaysia's wholly-owned subsidiary Malaysian Electronic Clearing Corporation Sdn Bhd (MyClear) and Group Managing Director of Malaysian Electronic Payment System Sdn Bhd (MEPS).

Notable awards received include CIO Excellence Award 2016 by PIKOM, Bank Technology Leadership Achievement in Asia Pacific for outstanding technology leadership by The Asian Banker, 2017 and he was named as one of the top financial services CIOs in South East Asia by International Data Group (IDG), CIO.com 2018

Presently, Mr. Suhail is a Director on the Board of MCB Bank Limited and a member of the Board's Business Strategy & Development Committee, Risk Management & Portfolio Review Committee and Information Technology Committee. Other directorships include Maybank Shared Services Sdn Bhd, MBB Labs Pvt. Ltd. and Technology Park Malaysia Corporation, Sdn. Bhd.

His credentials including being a Fellow of the Malaysian Institute of Management and Associate of the Asian Institute of Chartered Bankers Association, Chartered Banker from the Asian Institute of Chartered Banker as well as holding a Master of Business Administration from Charles Sturt University, Australia and an Advanced Management Program from Harvard Business School.

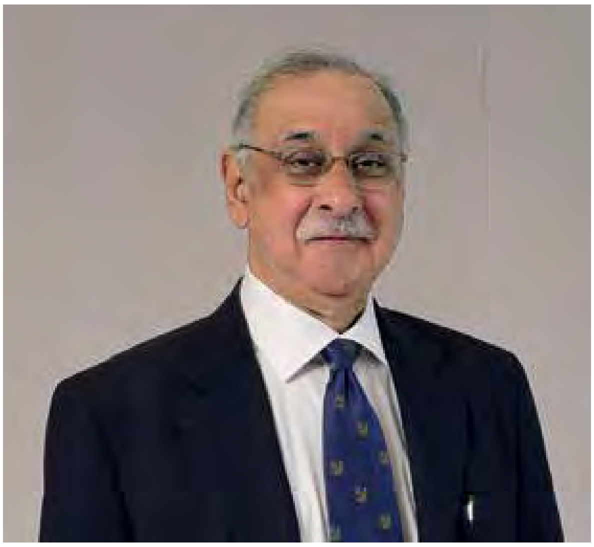

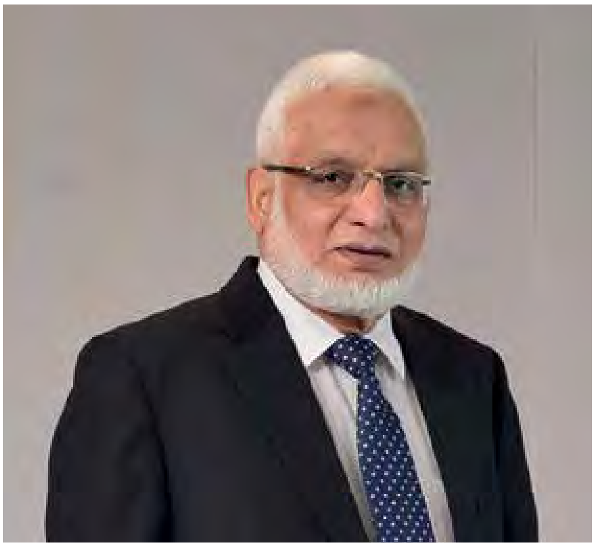

Shahzad Hussain
Director

After passing the final exam of England Institute of Chartered Accountants, i.e., The Institute of Chartered Accountants in England and Wales (ICAEW), Mr. Shahzad Hussain returned to Pakistan from UK, in early 1980s. In Pakistan, he became a member of the Institute of Chartered Accountants of Pakistan i.e. ICAP. Later, he was elected as a member of its Council for a term of four years and also served as Vice President (North), where he chaired the ICAP's Discipline Committee, reporting to the Council.

In 1980, after serving briefly in SNGPL, he joined A. F. Ferguson and Co., a network firm of Price Waterhouse Coopers (PwC). In early 1990's he was admitted to partnership and served the firm in Audit, Tax and consultancy. In 2003, he was made Partner In-Charge of Lahore Office, where he served until his retirement in 2015.

He has vast experience in Audit, Tax practice and in consultancy. He headed many assignments, including Asian Development Bank funded assignment for Punjab Government Resource Mobilization, where he gained considerable experience in Provincial Government organization structures and procedures in various fields.

Masood Ahmed Puri
Director

Mr. Masood Ahmed Puri is a multifaceted and accomplished senior executive with competencies in strategic and business planning, risk management and sound decision-making. He is a CEO and owner of different conglomerates in the field of logistics, shipping, supply chain, restaurants and textile within the GCC region for almost 45 Years. He started his career with Vegetable Ghee Industry in Pakistan but later on switched to logistics and shipping in the year 1976. He was hired as General Manager Finance in a logistics company in Saudi Arabia from where he took off and managed the overall operations of the Company. One after another, he kept on developing new businesses in the same field as well as diversified into textiles and restaurants all within the GCC region. Overall, he carries immense experience and exposure in various fields such as finance, corporate strategy, management, operations and most importantly on business start-ups.

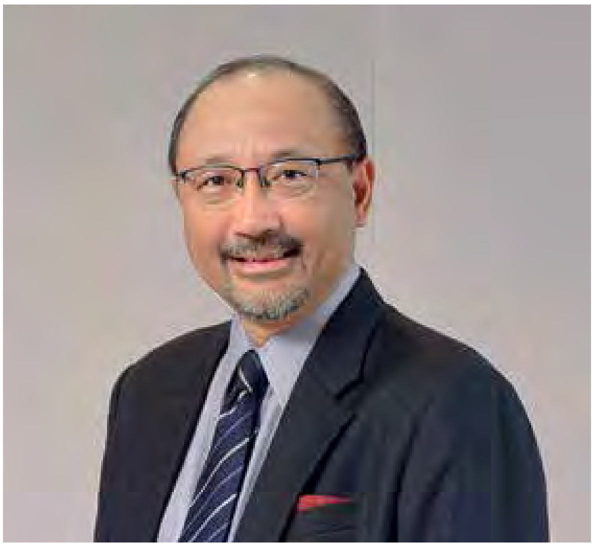

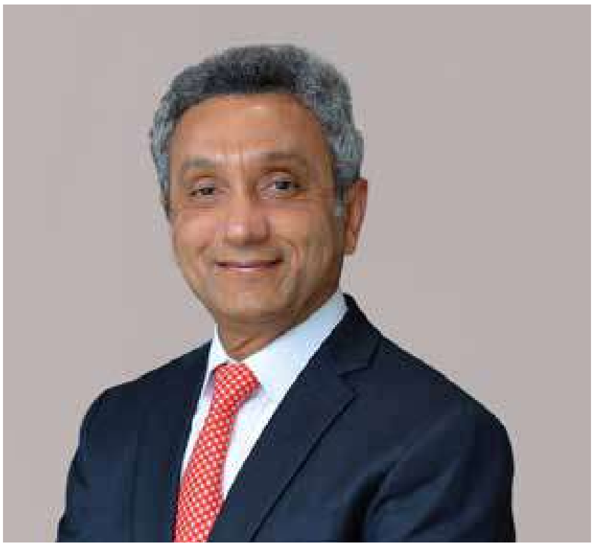

Shariffuddin Bin Khalid
Director

Mr. Shariffuddin Khalid was appointed as an Independent Non-Executive Director of Maybank on June 14, 2018. He also serves as Chairman of the Audit Committee and member of the Compliance Committee of the Board. He is a Fellow Member of the Chartered Institute of Management Accountants, United Kingdom. He has over 30 years' experience in the banking and corporate sector. He had served in key positions in the corporate services, business development, corporate communications and human resource functions. He was part of the pioneer management team tasked with the establishment of Pengurusan Danaharta Nasional Berhad ("Danaharta") during the 1998 Asian financial crisis. He served as General Manager, Communications and Human Resource, Danaharta, from its establishment in 1998 until 2005. He joined Bank Negara Malaysia (BNM), the Malaysian Central Bank and banking industry regulator in 2008, as the pioneer Director of the Malaysia International Islamic Finance Center. In this position, he was responsible for planning and execution of strategies to position and brand Malaysia as an international Islamic financial center. He spent nearly 10 years at BNM and his final position was Director, Strategic Communications. This entailed the provision of strategic communication advice to the BNM Governor and senior leaders, planning and execution of major media and PR campaigns as well as Parliamentary responses and all media relations and e-communications work. His current directorship within the Maybank Group includes Maybank (Cambodia) Plc and Maybank Islamic Bhd. Currently he also sits on the Board of Marine & General Berhad, a public company listed on the Main Market of Bursa Malaysia, as well as its subsidiaries.

Imran Maqbool
President & CEO

Mr. Imran Maqbool serves as President & Chief Executive Officer of MCB Bank Limited. He is a seasoned professional with over three decades of diverse, international banking experience. Prior to his current role, he was the Head of Commercial Branch Banking Group, where he successfully managed the largest group of the Bank in terms of market diversity, size of the workforce, number of branches, on a countrywide basis and diversified spectrum of products. His earlier, multi-faceted Group-Head assignments included heading Wholesale Banking Group–North, Special Assets Management and Islamic Banking. He was also posted as Country Head, MCB Sri Lanka.

Before joining MCB Bank in 2002, Mr. Maqbool was associated with local banking operations of Bank of America and Citibank for over seventeen years, where he worked in various senior management roles in the respective banks.

Mr. Maqbool holds an MBA degree from the Institute of Business Administration (IBA-Karachi) as well as an MS in Management from the renowned Sloan School of Management (Massachusetts Institute of Technology) in the USA.

Mr. Maqbool also serves as Trustee/ Chairman of MCB Employees Foundation.

Other Directorships:

- Adamjee Insurance Company Limited;
- Adamjee Life Assurance Company Limited.

Organizational **Structure**

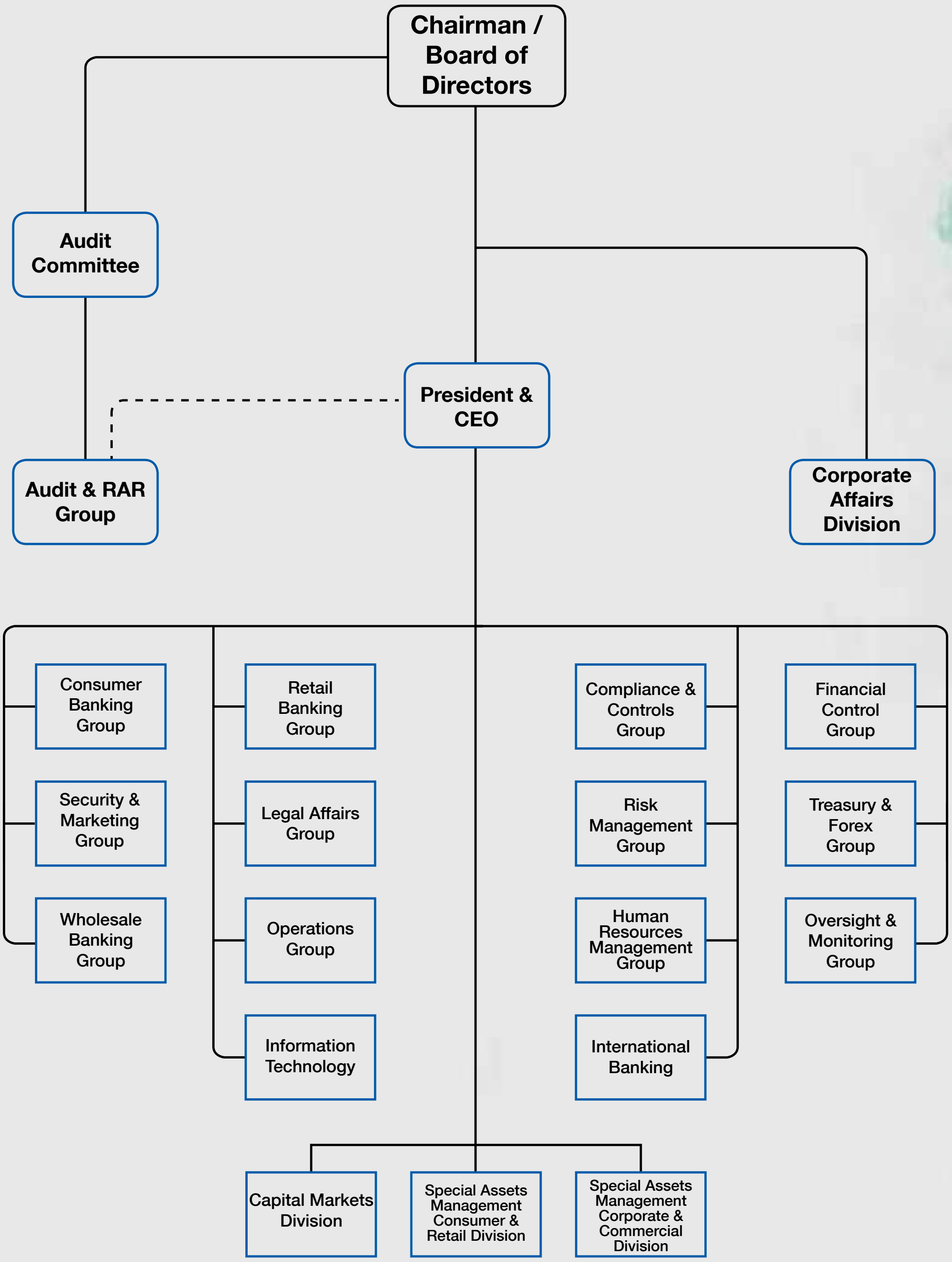

- - - - Administrative Reporting
——— Functional Reporting

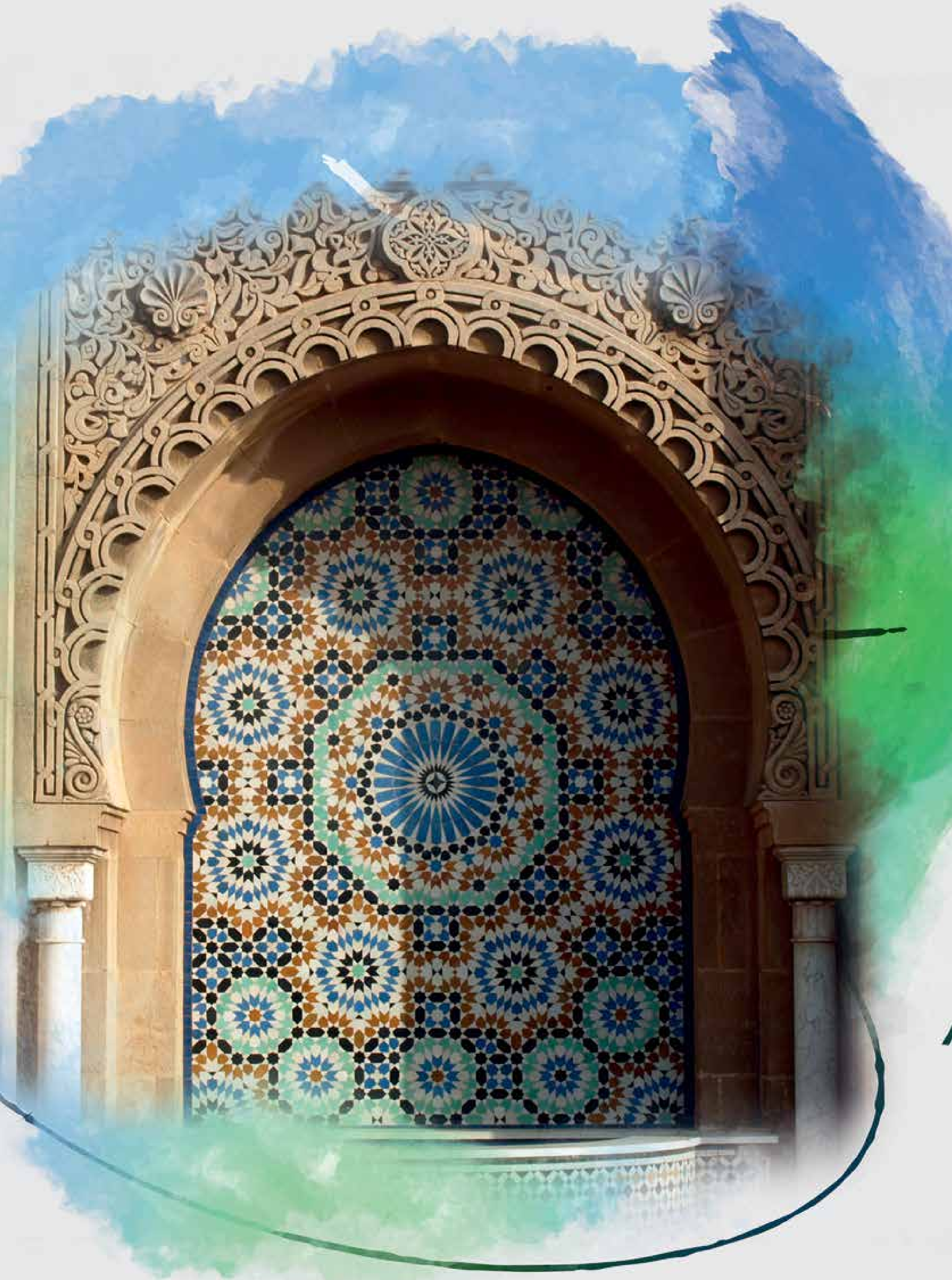

Management **Committee**

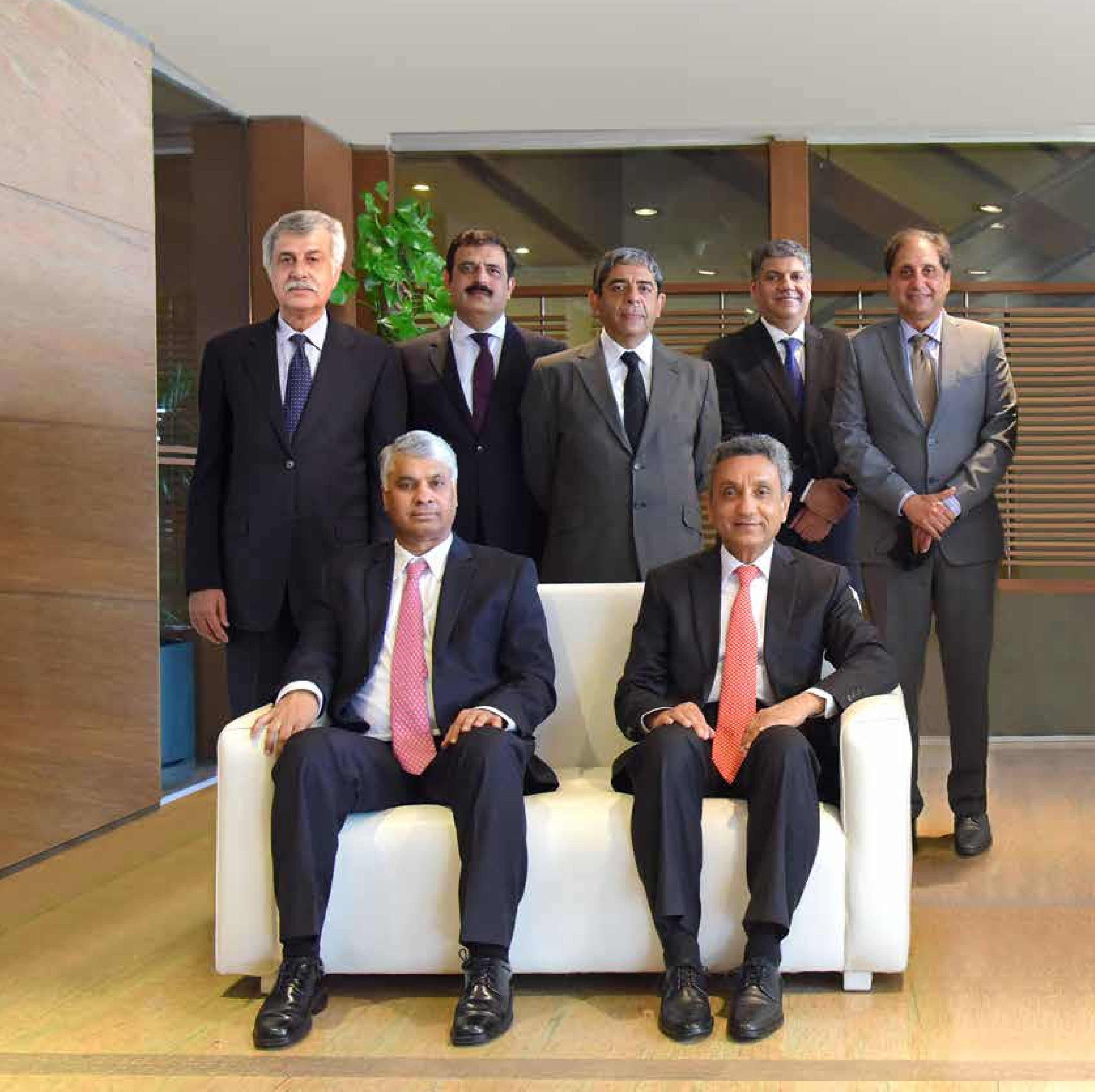

Standing L to R: Syed Mudassar Hussain Naqvi, Abrar Aleem, Usman Hassan, Shahzad Ishaq, Farid Ahmad
Sitting L to R: Hassan Nawaz Tarar, Imran Maqbool

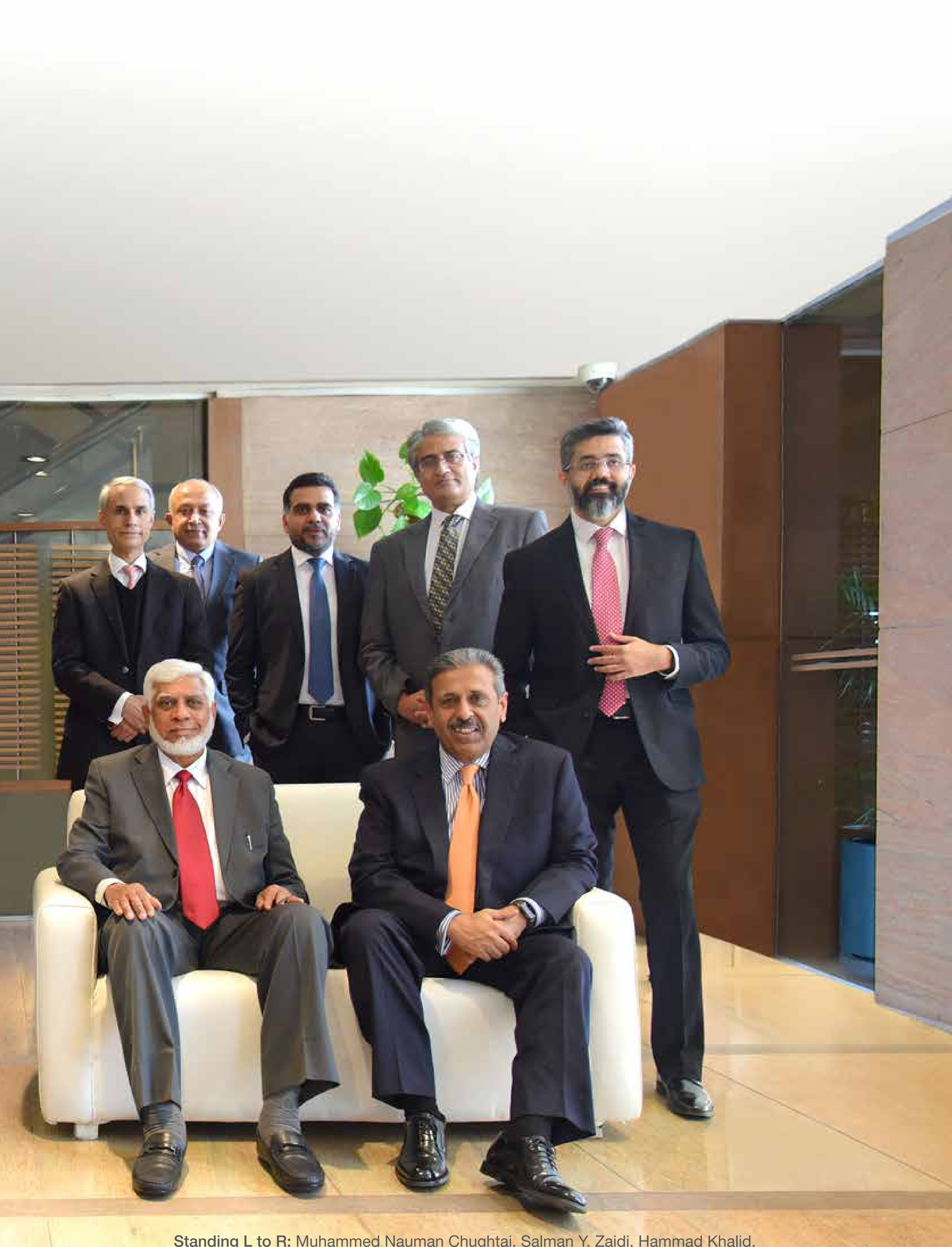

Standing L to R: Muhammed Nauman Chughtai, Salman Y. Zaidi, Hammad Khalid,
Zargham Khan Durrani, Kamran Zaffar Muggo,
Sitting L to R: Malik Abdul Waheed, Shoaib Mumtaz

Other Senior
Management

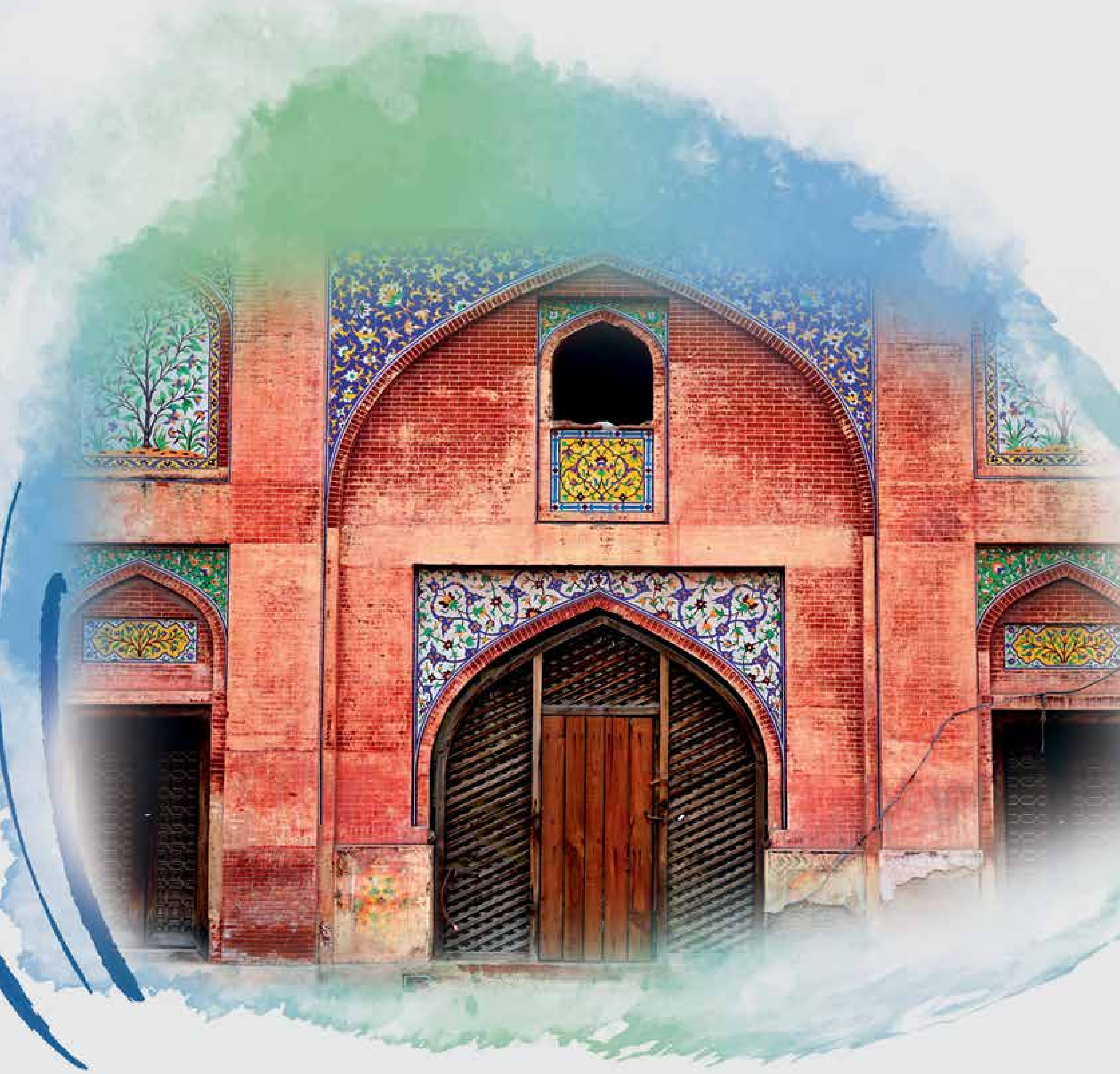

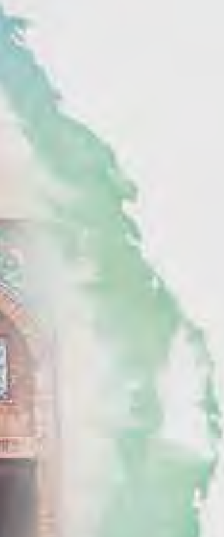

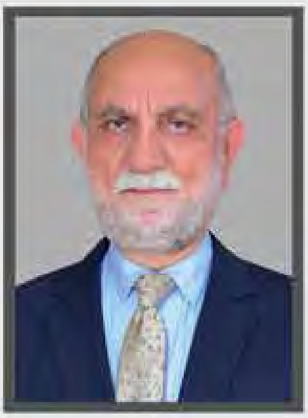

Fida Ali Mirza
Company Secretary

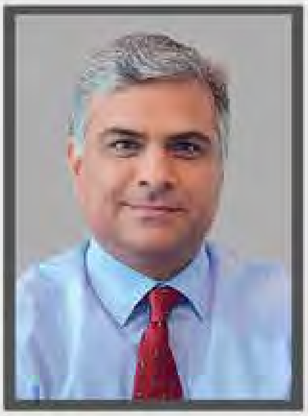

Aamir Khanzada
Country Manager, Bahrain

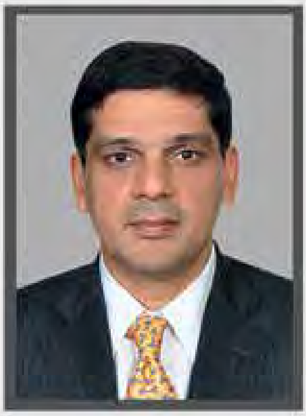

Aali Shafi
Country General Manager, Sri Lanka

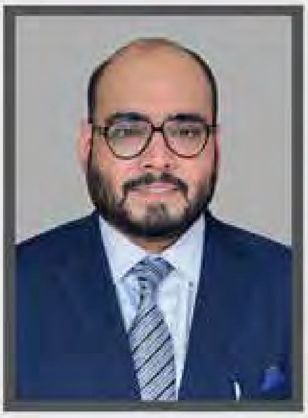

Muhammad Farooq Wasi
Group Head Audit

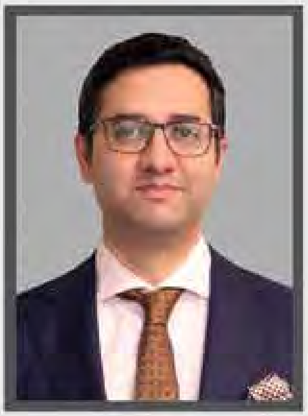

Omair Safdar
Head Capital Markets

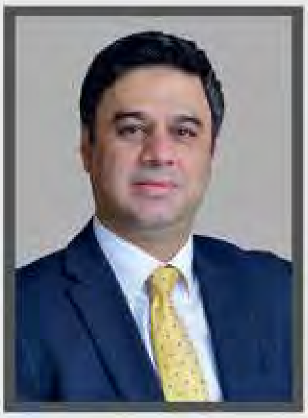

Muhammad Ali
Division Head
SAM - Consumer & Retail

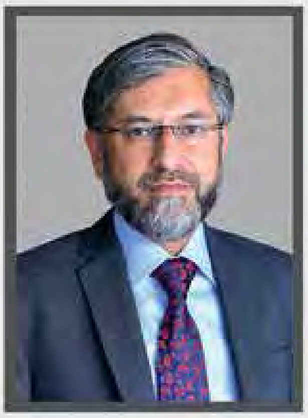

Muhammad Haris Hasan
Division Head
SAM - Corporate & Commercial

Entity Credit
Rating

Long Term

AAA

Short Term

A1+

Corporate Profile
of the Bank

MCB Bank is one of the oldest banks of Pakistan, incorporated in private sector in 1947. It was nationalized in 1974 and privatized in 1991. MCB Bank's major shareholding is owned by Nishat group a prominent business conglomerate, having diversified interests in Textiles, Cement, Banking, Insurance, Power Generation, Hotel Business, Agriculture, Dairy, Auto Manufacturing and Paper Products. To enter in international capital markets, the Bank launched its Global Depositary Receipts (GDRs) in 2006. It was the first Pakistani Bank that got its GDRs listed on the London Stock Exchange. In 2008, the Bank entered into a strategic partnership with Maybank, Malaysia, which owns 18.78% stake in MCB through Maybank International Trust (Labuan) Berhad. In 2017, Fullerton Financial Holdings (International) of Singapore through Bugis Investments (Mauritius) Pte Ltd acquired 5.49% stake in MCB under merger scheme of NIB Bank with and into MCB Bank Limited. MCB is the first Pakistani Bank which incorporated a wholly owned Islamic Banking subsidiary, MCB Islamic Bank Limited, to meet requirements of a significant segment of society for financial solutions that conform to Shari'ah rulings and demonstrate our confidence in the potential of the Islamic Banking industry in the country.

The Bank operates a strong and vast network of over 1,400 Branches and over 1,400 ATMs in Pakistan and 11 branches overseas with a footprint in UAE, Bahrain and Sri Lanka. With a customer base of over 7 million, MCB leads the banking & financial services sector in Pakistan and customers across the globe have 24/7 access to MCB Bank via our World Class Internet Banking. The Bank on consolidated basis is operating the 2nd largest network of more than 1,600 branches in Pakistan. The Bank enjoys highest local credit ratings of AAA / A1+ categories for long term and short term respectively, based on PACRA notification dated June 26, 2020.

Subsidiaries

MCB Islamic Bank Limited

Holding: 100%
Profile: Objective of the Bank is to carry on Islamic Banking Business in Pakistan in accordance and in conformity with the principles of Islamic Shari'ah and in accordance with regulations and guidelines of the State Bank of Pakistan.

MCB - Arif Habib Savings & Investments Limited

Holding: 51.33%
Profile: Asset management, investment advisory, portfolio management, equity research and underwriting.

MCB Non-Bank Credit Organization Closed Joint Stock Company

Holding: 99.94%
Profile: It leases various types of industrial equipment, public transports, real estate and retail auto.

Financial & Management Services Pvt. Limited

Holding: 95.90%
Profile: The Company is in dormant status and transferred to MCB from Ex. NIB under merger scheme. The Bank's investment in the company is fully provided.

Associates

With reference to significant holding, the following entities are associates of the Bank:

Adamjee Insurance Company Limited

Holding: 20%
Profile: The Company is engaged in the general insurance business.

Euronet Pakistan (Private) Limited

Holding: 30%
Profile: To provide outsourcing services to banks and financial institutions for Automated Teller Machine (ATM) network and managed services for Point of Sales (POS) terminal networks.

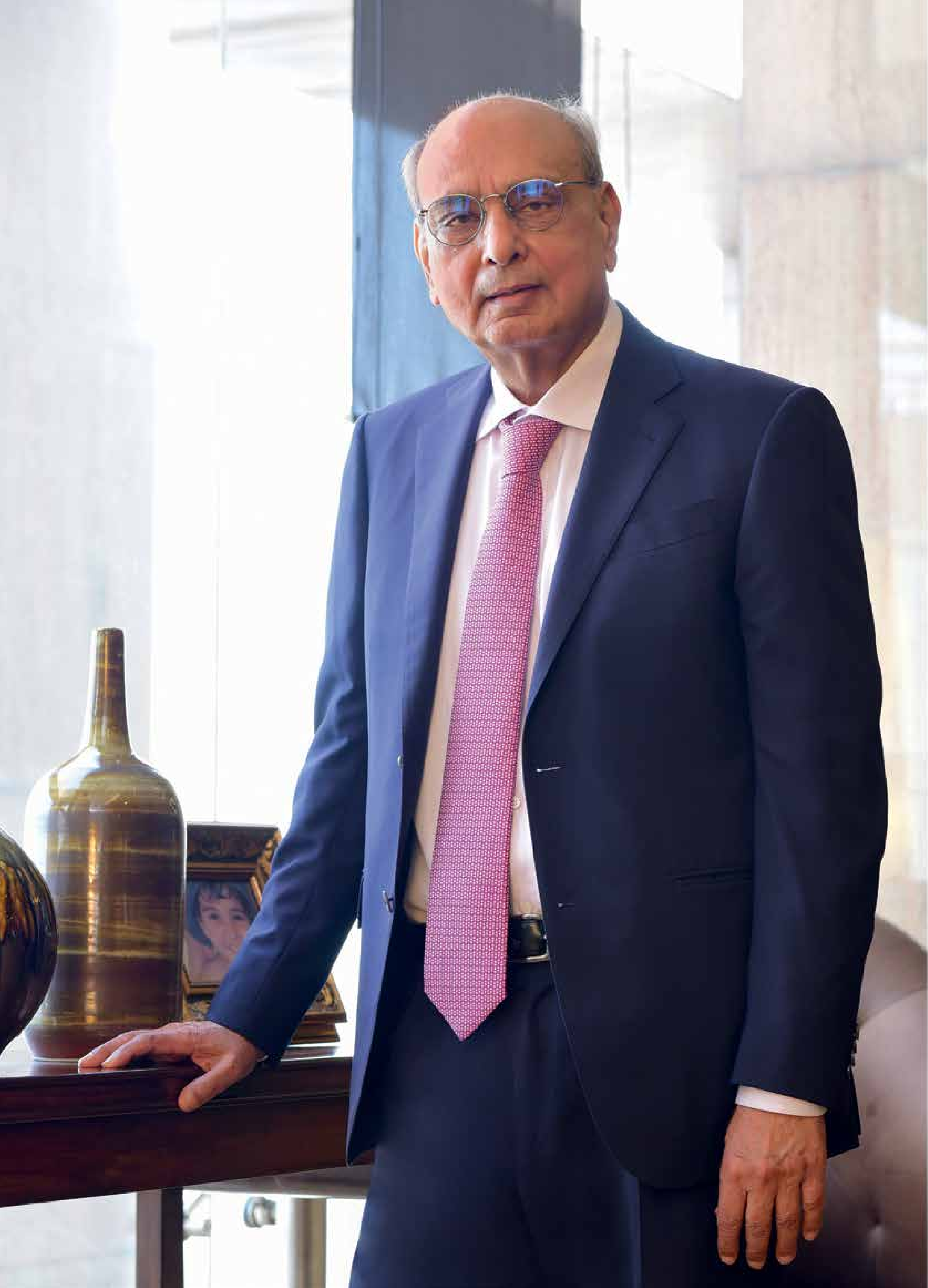

Chairman's Review

I am pleased to present this report to the shareholders of MCB Bank Limited on the overall performance of the Board of Directors and effectiveness of the role played by the Board in achieving Bank's strategic objectives.

The Board set the Bank's strategic aims to uphold our vision, mission and core values. It exhibited high standards of business and professional conduct in managing and supervising affairs of the Bank. During the year, the Board performed an in-house performance evaluation of the Board as a whole, its Committees, the Chairman, the President & CEO and Individual Directors. Last year, such performance evaluation was made by M/s Pakistan Institute of Corporate Governance ("PICG") as an external independent evaluator.

The overall rating of the Board is highly encouraging, particularly in respect to its composition, expertise, effective risk management, adequate system of internal controls and audit function. The Board has always focused on the preservation of the interests of Bank Shareholders and has strived to maintain a balance between regulatory obligations and operational requirements. As part of this effort, the Board's properly structured Committees are in place, with each one having well-defined objectives and appropriate Terms of Reference; performing their respective roles effectively and efficiently.

The past year has been difficult in both national and international contexts due to the effects of the Coronavirus pandemic. As in many other countries around the world, Pakistan's economy has also experienced limitations to growth. Under the Board's strategic advice and direction, the Bank's management followed a pragmatic approach to steer the Bank through uncertainty. The Bank has played its role in the fight against Coronavirus pandemic with targeted donations in the healthcare sector, such as the donation of protective equipment for frontline health workers to the Government of Punjab and the timely provision of rations to needy families affected by the COVID-19 lockdown. In such difficult circumstances, the MCB Bank team has shown resilience and fortitude.

We have consolidated avenues for growth and efficiency. The Bank has kept a close eye on identifying opportunities that promote cross-functional synergies and minimize costs. The Bank has recorded highest ever profit in 2020. MCB Bank has promoted the use of digital banking channels to reduce customer branch visits in the interest of public health. In partnership with our industry regulators, our Bank has successfully helped propagate the Roshan Digital Account initiative aimed at overseas Pakistanis to boost the volume of inward remittances and reduce the use of informal channels. These efforts have borne fruit in ensuring the profitability of the Bank and encouraged the inflow of much needed foreign exchange to our economy. It is self-evident that the positive trajectory of the Bank would not have been possible without the dedication and hard work of our employees. The Bank has always promoted cultivation of talent from within the organization and it is noteworthy that much of the Bank's senior management has ascended from the ranks in line with their commitment to our shared vision. Their valuable contributions have been central to taking the organization to the next level.

Mian Mohammad Mansha
Chairman
MCB Bank Limited

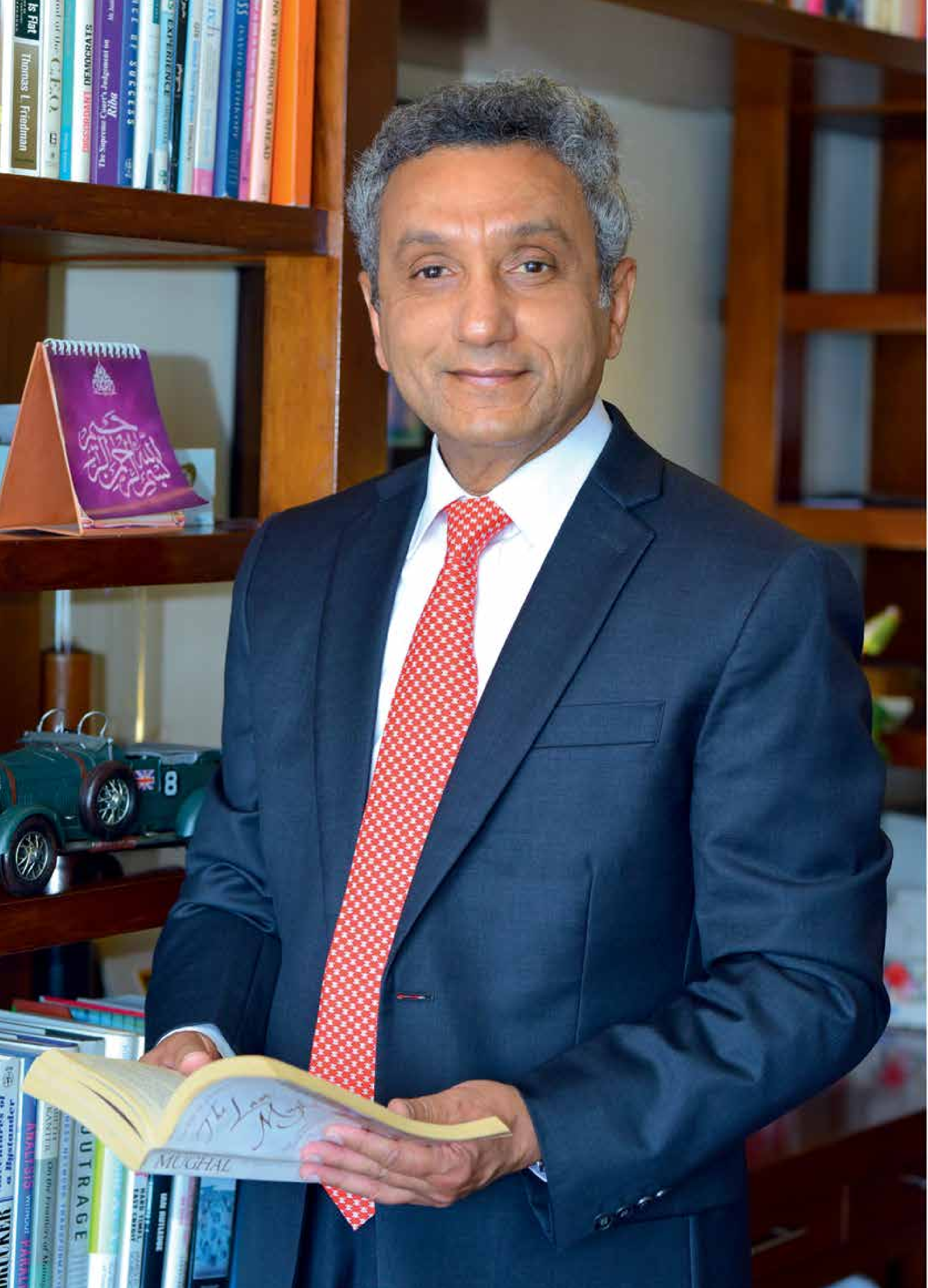
Thomas L. Friedman
DEMOCRATS
The Supreme Court's Judgement on Riba
MUGHAL
OUTRAGE

President's Review

The world around us changed significantly in 2020 due to the COVID-19 pandemic and heightened business instability that resulted in a record drop in interest rates. Despite many challenges presented by the pandemic, MCB team performed remarkably to support our valued customers and deliver a solid financial performance in unprecedented times.

MCB Bank reported its highest ever Profit Before Tax of Rs. 48.25 Billion (+20.3%) and declared a 200% cash dividend for the year 2020 despite extremely restrictive challenges posed by COVID-19 pandemic. Assets of the Bank grew by 15.99% over last year and stretched to Rs. 1.76 trillion. Sizeable growth in current deposits, timely shift in maturity profiling of investments and favorable re-pricing lag enabled the Bank to increase its net interest income by 19.7% to Rs. 71.33 billion, improving Return on Assets (ROA) and Return on Equity (ROE) to 1.77% and 18.88%, respectively. In comparison with last year Non-Fund Based income registered a considerable growth of 9%.

In spite of the challenges posed by low interest rate environment, we managed to achieve excellent growth in our deposit base, which demonstrates the loyalty of our customers, earned through providing quality service. Growth in deposits was of Rs. 144.7 Billion (12.6%) against last year, with increased Current Account-Savings Account (CASA) concentration of 93% as of December 31, 2020.

Despite sustained inflationary pressures, the Bank's strategic focus of balancing short term tactical cost reductions with long term cost initiatives has assisted in containing growth in administrative expenses; in turn registering a decline of Rs. 129 million during the year. Thus the cost to income ratio of the Bank significantly improved to 36.87% from 43.4% reported last year. In anticipation that customers affected by the pandemic might require provisioning once SBP's relaxations and waivers expire in 2021, the management has exercised prudence and booked a general provision of Rs. 4.0 billion during the year under review, hence providing insulation and loss absorption capacity against any risks to asset quality.

While complying with the regulatory capital requirements, the Bank's total Capital Adequacy Ratio (CAR) is 20.98% against the requirement of 11.50% (including capital conservation buffer of 1.50% as reduced under BPRD Circular Letter No. 12 of 2020). Quality of the capital is evident from Bank's Common Equity Tier-1 (CET1) to total risk weighted assets ratio which comes to 17.03% against the requirement of 6.00%. Bank's capitalization also resulted in a Leverage Ratio of 7.03% which is well above the regulatory limit of 3.0%. The Bank reported Liquidity Coverage Ratio (LCR) of 237.72% and Net Stable Funding Ratio (NSFR) of 174.86% against requirement of 100%.

MCB actively participated in State Bank of Pakistan's policy measures to support the economy during COVID-19, by offering loan deferment and/or loan rescheduling, offering liquidity in Rozgar Scheme and partnered with the Central Bank in extending concessionary "Temporary Economic Refinance Facility" (TERF) to facilitate investment in new industrial projects, as well as, capacity expansion.

MCB Bank is proud to be one of the leading banks that offered the SBP mandated Roshan Digital Account, a digital banking solution for Non-Resident Pakistanis (NRPs) and Resident Pakistanis with declared foreign assets. Banking industry is undergoing significant transformation which has been facilitated through digitization of banking services and propulsion of e-Banking and e-Commerce. Progress in this rapidly evolving environment lies in cross-industry collaboration, cash minimization and digitization. We are determined to increase the pace at which we digitize MCB through greater investments in technology. Our Digital Banking team's proactive and innovative initiatives have created industry wide impact and have elevated MCB Bank and its digital offerings to emerge as a strong force in the payment industry, all translating into strong customer relationships. The bank witnessed a substantial increase in Mobile Banking transactions during 2020. With a cumulative number of 500,000+ App downloads, the business is geared up to explore new possibilities along different verticals. During the year, we welcomed more than 500,000 new customers, raising our customer base to 8.2 million accounts, which effectively demonstrates our customers' trust in MCB. We offered accessible banking infrastructure for differently–abled persons to support equitable access to banking and financial services. We also performed well in our Consumer Lending and Wealth Management businesses. Consumer lending book size at the end of year 2020 was over Rs. 28 billion, serving over 134,000+ customers across all lending products. 13,944 credit cards were issued during the year while 5,549 auto loans were disbursed in 2020. A key highlight of Consumer Banking strategy was our joining national drive on providing low cost housing facility to masses. Specifically, we aligned our organizational direction and added substantial resources to drive Government's Markup Subsidy Scheme for Housing Finance, as we aim to continue building our efforts on this front. Likewise our Investment Services and Bancassurance registered fresh sales of Rs. 5.7 billion and Rs. 2.6 billion, respectively.

Our commitment to improve our conduct and controls is factored into every strategic and operational decision we make. We remain on the front line in the fight against financial crime and take this responsibility most seriously. Every activity of MCB Bank passes through a compliance filter which serves as the backbone of the institution for regulatory compliance.

Compensation and benefits are our largest expense that accounts for almost half of all administrative expenses; reflecting our sincerity and commitment to recognizing the performance of our hardworking and professional human resource capital. At MCB we are building an inclusive organization to address the gender gap and allow everyone an opportunity to fulfill their career aspirations and professional goals. We set clear compelling directions, engage employees to Bank's vision, ensure open and honest communication and recognize high performance to retain and attract best talent in the market. During the year, employees' development and trainings remained our foremost priority as our committed and professional team is our strategic advantage.

We continued to explore investment opportunities within and outside Pakistan in order to further expand our local as well as international footprint and strengthen our outreach beyond our existing network. We believe that there are significant opportunities for MCB in the medium to long term across its footprint. Our strategic focus helped increase customer confidence as MCB Home Remittances volume surged to Rs. 519 billion with an impressive growth of 13.8% over last year. MCB Cash Management crossed the annual volume milestone of Rs. 2.0 trillion in 2020 and remains one of the leading Banks in corporate cash management.

Our efforts to improve internal operational environment of MCB Bank were rightly recognized at different forums. The strength of our Bank was also reassured by the Pakistan Credit Rating Agency (PACRA), which maintained MCB's credit ratings at AAA [Triple A] for long term and A1+ [A one plus] for short term. Our performance was acknowledged by various local and international entities. Asiamoney declared MCB as the "Overall Most Outstanding Company in Pakistan 2020" while for the eighth consecutive year, the joint committee of Institute of Chartered Accountants of Pakistan & Institute of Cost and Management Accountants of Pakistan (ICAP/ICMAP) awarded the "Best Corporate Report" award in Financial Sector category to MCB's annual report for 2019. These and many other awards encourage us to pursue the strategies that have served us and our stakeholders well throughout.

For 2021 outlook, we are cautiously optimistic for the year ahead. On COVID-19 pandemic front, development of multiple vaccines and their gradual roll-out have created hope of healthy recovery during 2021. In MCB Bank, we're all focused on how to best protect and support our employees, customers, families and communities in 2021 & beyond.

Our pride is not in our numerous landmark buildings spread across the country but is in the millions of relationships we have forged over generations with customers, team members and shareholders. Our Capital strength and earnings power are as strong as they have ever been. Challenges still exist, and there's always room for improvement. As we head into 2021, we remain committed to serving our customers, colleagues and shareholders while being steadfast in our compliance with regulations.

Finally, I would like to acknowledge the hard work and dedication of all staff members of MCB, who are undoubtedly the core asset of our Bank. We are fortunate to have a diverse group of qualified, trained and dedicated employees across Pakistan who view MCB Bank as a great institution to begin, build and sustain their careers.

I am also greatly indebted to the guidance of the Chairman, Board of Directors, our foreign partners and the staff members who helped in making 2020 yet another profitable year.

In closing, I feel humbled to be given the opportunity to not only represent one of the leading banks of Pakistan but to also be a member of this team. With our energy, team work, unity of purpose and faith in GOD, we will continue to operate as "One Bank One Team" to meet the high expectations of our shareholders, customers, employees and regulators in coming years.

Imran Maqbool
President & CEO
MCB Bank Limited

Highlights 2020

PBT
PKR 48.25 billion
(+20%)

PAT
PKR 29.04 billion
(+21%)

Assets
PKR 1,757 billion
(+16%)

ROA
1.77%

ROE
18.88%

Deposits
PKR 1,290 billion
(+13%)

Investments
PKR 1,016 billion
(+36%)

Net Markup Income
PKR 71.33 billion
(+20%)

Highest Market Capitalization
In the Industry
PKR 220 billion
(December 31, 2020)

Overall Most Outstanding Company in Pakistan 2020
Award by Asiamoney

Winner of BCR - 2019 by ICAP/ICMAP
(Banking Sector)

MCB
Bank for Life

Financial Performance 2010 - 2020

10 Years Trend - Rupees in Billion

Total Assets

CAGR 11.9%

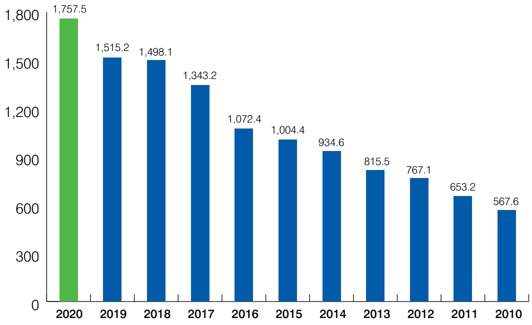

Deposits

CAGR 11.6%

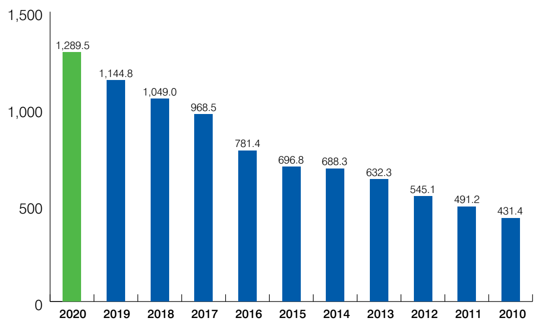

Gross Advances

CAGR 6.5%

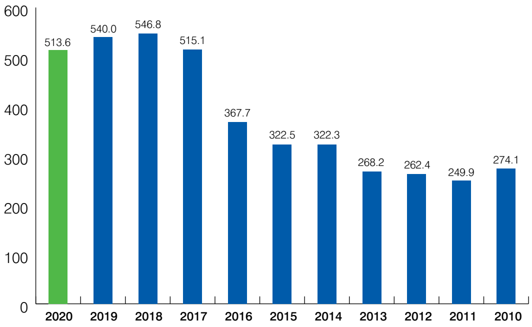

Investments

CAGR 16.9%

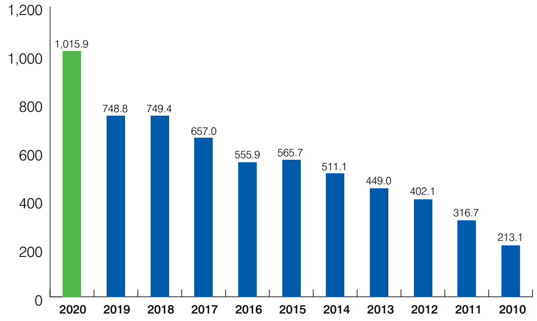

Fund Based Income

CAGR 6.8%

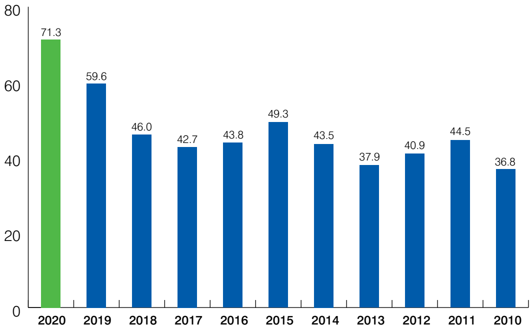

Non Markup Income

CAGR 11.2%

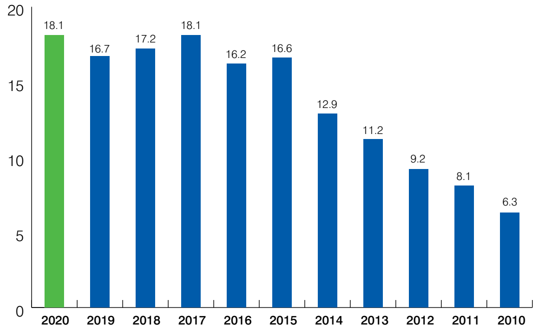

Profit Before Tax

CAGR 6.3%

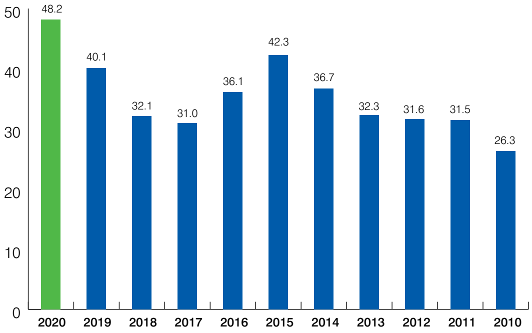

Profit After Tax

CAGR 5.6%

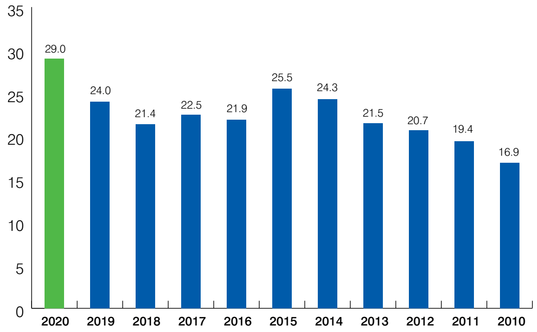

Forward Looking Statement

The Annual Report of MCB Bank Limited carries forward looking statements in its different sections. Since there are uncertainties related to the occurrence of future events, these should be read in conjunction for decision making by the users of the Annual Report. Forward looking statements contain words such as expect, anticipate, believe, seek, will, may, would, presume, assure, hope, so on and so forth. A forward-looking statement naturally addresses matters that are, to certain degrees, uncertain and may not happen. In most cases, a forward-looking statement is made in respect of Bank's expected income, earnings, business growth, horizontal expansion, cost structure, capital structure, dividends etc.

Pakistan's Economic Outlook

Timely measures taken by the government to curtail the spread of novel COVID-19 outbreak and to support the economy has made the outlook for 2021 less challenging. Amidst a subdued forecast for GDP growth, the government faces the challenge of stimulating the economic activity without significantly increasing the inflationary pressures.

Moreover, all eyes are currently focused on the re-initiation of the IMF Program which was stalled when the Covd-19 pandemic hit the country. The IMF Program bodes well for international confidence in Pakistan and adds to the government's capability to tap international markets. In addition, it also holds significance on account of the follow through impact it has on fiscal management, especially through the elimination of subsidies and inefficiencies. Hence, any positive outcome of the negotiations with IMF will lead to the betterment of the economy.

MCB's Future Outlook

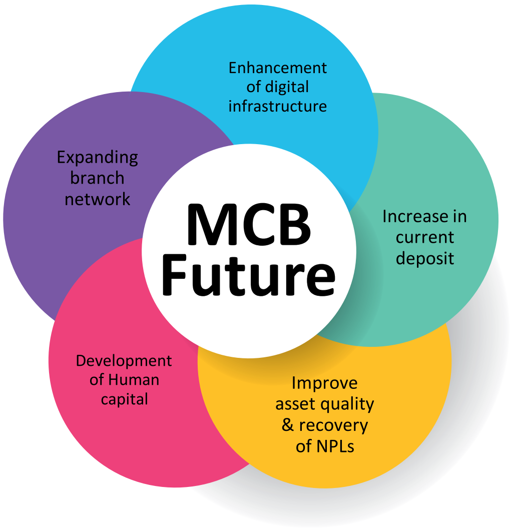

Pakistan's banking sector will continue to navigate through the tough economic situation in the year 2021 on account of falling earning margins due to current policy rate regime and compounding risks to the asset quality given a subdued outlook for business activity and uncertainty surrounding the COVID-19 outbreak. Considering the existing disruptions, complexities and uncertainties, the socio-economic and regulatory landscape will continue to transform rapidly; hence, further exacerbating the challenges surrounding the operating environment.

Irrespective of the above captioned challenges, the Bank will strive hard to deliver results for its stakeholders in 2021. The Bank's strategic plan, centered on the key pillars of customer centricity, geographical expansion, technology and cyber security and people development (among others), takes into consideration the evolving operating and economic scenario and paves Bank's future road map.

The widespread norms to contain the spread of COVID-19, including social distancing and limiting the size of gatherings, is expected to fundamentally alter the consumer behavior of banking customers. At MCB, our proactive stance to further augment branch outreach while sustaining parallel focus on creating safe and secure digital and alternative banking channels shall hold us in good stead as the customers increasingly look to access non-physical banking modes.

We would continue to improve our asset quality, increase low cost deposit base, inculcate operational efficiencies and increase contribution from the non-markup segment through materialization of cross sell business opportunities with corporate clients.

Credit appetite being a mainstream business line of the Bank, instigates us to avail all righteous credit extension opportunities that fall within the defined risk appetite of the institution; while proactively monitoring watch listed portfolio of the bank to minimize any further infection and ensure that recoveries are made in line with the agreements.

On the investments side, the Bank is committed to optimally manage excess liquidity through strategic re-profiling of the investment book in an evolving yield curve scenario.

We would continue to lead the market position through focused initiatives targeting new products, new markets, branchless banking and effective cost management. The Bank is investing in developing a workforce for the 'Digital Banking Age' by attracting and retaining the right skillset and competence; hence, aligning it with the evolving business dynamics.

Driving customer centricity remains a key area of focus for the next year; we will direct investments towards empowering employees to drive a pleasant customer experience across all customer touch points. Also, in all our capacity and skills enhancement programs for the employees, we will continue emphasizing the need for the staff to serve customers as a means of consolidating our position as the most sought after bank in the industry. To sum up, employees' development and trainings would remain at the forefront of our strategic focus. We would acutely remain conscious in attracting and retaining the best talent pool.

We are committed in maintaining our unique positioning as a diversified financial institution with a robust heritage and strong reputation through enriched service offerings and financially viable tailored products to meet requirements of our esteemed customers; hence, enabling us to serve both our existing and next-generation customers in 2021 and beyond while translating the underlying financial strength of the entity into profits.

Key Projects to Support Future Performance

Details of Projects
Compliance Risk Management -For Domestic Operations.
MPG – Micro Payment Gateway
CAMS Upgrade (New Loan Origination System for Auto Loan)
AIMS (Audit Interactive Management System)
FCCM Upgrade & TBML Implementation
3D card security protocol implementation
Compliance Risk Management for Overseas Operations
e-kyc application updating with respect to Trade Based Money Laundering
ATM Insourcing Project (ATM Migration & Payment Scheme Certifications)
iSheild - Fraud management system.
Rosetta Integration with Safewatch

Quantitative Projections

Outlook	Key Risks Going Forward
Deposit growth to be achieved in line with 2020 levels.	Increased competitive rivalry within the industry for mobilizing deposits amidst low differentiation and switching costs.
Net Interest Margin (NIM) expected to fall from the 2020 level.	Risk of slower then expected advances growth and net interest margin decline in response to lower interest rates.
Non Markup Income to achieve double digit growth.	Risk of lower growth as transactional volumes slow and markets decline. Resurgence of the virus resulting in limited physical interaction with the clients.
Expenses growth to be contained in single-digit.	No projected risks; continue to be well managed with a focus on lower discretionary spend, inculcation of operational efficiencies through business process automation and implementation of strong budgetary controls.

CET1 ratio to be adequately kept within prescribed regulatory limits.	Higher Risk Weighted Assets (RWA's).
Constant / stable dividend payout to be maintained.	Regulatory restrictions impacting dividend payout.

Uncertainties that could affect the Bank's Resource, Revenues and Operations

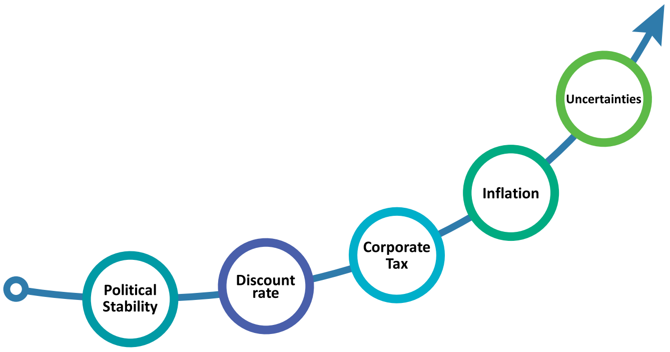

All forward-looking statements are, by nature, subject to risks and uncertainties, many of which are beyond control. Major factors that can affect the Bank's resource, revenues and operations are given below:

Discount rate / Monetary Policy: Based on different assessment parameters, the State Bank of Pakistan may change the monetary policy rate. Any increase in the discount rate will initially have an adverse impact on Bank's net interest income due to the repricing lag between earning assets and liabilities. However, as the rate stabilizes, the net interest margins will improve and have a positive impact on Bank's profitability.

The impact of interest rate sensitivity on the banks profitability has been disclosed in note 45.2.4 of the financial statements.

Inflation: Inflation is considered to be a key determinant of policy rate change. Any uptick in the inflation statistics will have a material impact on the monetary policy rate along with other drivers.

Political Stability & Law and order situation: Political stability and controlled law & order situation is a pre-requisite for any economy. This, in turn, reposes investor confidence in the soils of Pakistan, making our corporates a potential investment opportunity. However, any act of terrorism or political instability can negatively impact the economy /equity market, thus resulting in decreased profitability.

Corporate Tax rate: Any increase in the corporate tax rate or continuation of super tax will adversely impact the profitability of the Bank.

External Environment

The Bank's external environment, including political, economic, social, technological, environmental and legal factors have an impact on business performance, strategic objectives and availability, quality and affordability of capitals.
Details have been disclosed in the risks and opportunities section of this report.

Status of Projects Disclosed in the Forward-Looking Statement of Previous Year:

Detail of last year projects	Status
Loan Originating System	Completed
Issuance of Dual Interface Card	Completed
Privilege Identity Management	Completed
Mobile Device Management (MDM) Tools	Completed
Automation of Intercity Charges	Completed
Reuters Integration with Treasury system	Completed
3D card security protocol implementation	In process
e-kyc application update	In process

Performance of the Bank against Forward-Looking disclosure of 2020 as Presented in the Annual Report 2019

Forward-looking disclosure for 2020 as presented in annual report 2019	Performance of the Bank in 2020 against forward-looking disclosure
Pakistani banking sector will continue to face headwinds in 2020, on the back of rising interest rates and slowing economic growth; irrespective of these facts, the Bank is committed to deliver remarkable results to its investors in 2020. Our strategic agenda for 2020 is guided by the Bank's five year corporate plan. The Plan is centered on strategic pillars including customer centricity, geographical expansion, technology and cyber security and people development (among others) which are aimed at building a competitive and future fit.<br><br>We are committed in expanding our network of branches to meet our valuable customers' expectations with parallel investment on the digital infrastructure. We would continue to improve our asset quality, increasing low cost deposit base, deploying cost efficient techniques and increasing contribution from non-markup segment. We will exceedingly focus in increasing low cost CASA base to increment our net interest margins.<br><br>On credit side the Bank is geared up to continue with its growth strategy in 2020, with continued focus on book building, albeit with cautious approach, given overall slowdown in the economy which is expected to continue into 2020; while proactively monitoring watch listed portfolio of the bank to minimize any further infection, while ensuring that recoveries are made in line with agreements. We will also continue to focus on cross sell initiatives with corporate clients for additional business opportunities to augment fee income.<br><br>On investment side, the Bank is committed on strategic profiling of the investments based on the interest rate calls which will be a gradual shift from shorter to longer term investments.<br><br>Recovery from infected portfolio would remain one of the key targets in 2020. We would continue adding products and services to our menu and anticipate significant growth in our non-fund based income. The Bank will continue to invest in developing workforce for the 'Digital Banking Age' to align ourselves with the ever changing business dynamics.<br><br>Driving customer centricity will again be a key area of focus in next year; we will direct investments towards empowering employees to drive a pleasant customer experience across all our touch points. Also, in all our capacity and skills enhancement programmes to the	MCB's profit before tax grew to Rs. 48.24 billion which reflects a tremendous growth of 20% over 2019, despite a tough operating and macro-economic environment due to the COVID-19 outbreak. The key highlights were impressive increase in net interest margins through a gradual shift in the maturity profiling of the investment base amidst evolving yield curve expectations along with a more refined cost structure.<br><br>During the past few years, the lack of credit opportunities resulted in the banking sector liquidity being deployed in Government Papers as the Government's borrowing appetite has continued to grow. Net interest income rose to Rs. 71.33 billion, 20% higher than the last year primarily due to the increase in investment income. Analysis of the interest earning assets highlights that income on advances decreased by Rs. 14.45 billion, primarily on account of decrease in yield of 236bps. On the investment side, gross markup income increased by Rs. 16.50 billion, primarily due to increased average volume by Rs. 195.78 billion. On the interest bearing liabilities side, the cost of deposits decreased by 146bps over the corresponding year.<br><br>The non-markup income block of the Bank was reported at Rs. 18.13 billion with major contributions coming in from fee commission and foreign exchange income. One of the major revenue line supplementing the fee growth was branch banking customer fee. During the year 2020, consumer lending function underwent a series of challenges, mostly brought about by the COVID-19-pandemic. Despite the impact of COVID in 2020, Bank booked fresh auto loans of Rs. 8.97 billion. Credit card business has exhibited growth with closing the book standing at Rs. 3.15 billion; depicting a growth of 17% over the last year. Bancassurance business achieved sales volume of Rs. 2.6 billion.<br><br>Despite the inflationary surge during the year, growth in the operational network and constant investment in digital, cyber security and information technology related platforms, the operating expense (excluding PF reversal) was decreased by Rs. 129 million as efficient cost management remained one of the key strengths for MCB Bank Limited.

Forward-looking disclosure for 2020 as presented in annual report 2019	Performance of the Bank in 2020 against forward-looking disclosure
employees, we will continue emphasizing the fact that, It is undoubtedly our ability to delight customers that is key to retaining our position as the most sought after bank in the industry. To sum up, employees' development and trainings would remain our foremost priority as committed and professional team is our strategic advantage. We would acutely remain conscious in retaining and attracting the best talent.<br><br>We are committed in maintaining our status of one of the leading banks operating in Pakistan through enriched service quality, financially viable tailored products to meet requirements of our esteemed customer and translating the underlying financial strength of the entity into profits.	MCB Home Remittances surged to generate a volume of PKR 519 billion, registering a growth of 14% over last year while the trade volumes decreased by 1.2%, over the corresponding year, as a result of the slow down witnessed in economic activity.<br><br>On the provision side, Bank made continued progress on its strategic path and recorded a cumulative recovery of Rs.2.4 billion by settling a large number of hardcore and protracted defaults.<br><br>On the liabilities side, the deposit base of the Bank registered a significant increase of Rs. 144.739 billion (+13%) over December 2019.<br><br>Return on Assets and Return on Equity were reported at 1.77% and 18.88% respectively, whereas the book value per share was reported at Rs. 137.02.<br><br>MCB remained active throughout Pakistan, UAE and Sri Lanka through diverse network of 1,441 branches (including 12 sub-branches) and more than 1,430 ATMs.<br><br>To enhance the knowledge and skillset of its workforce, a number of trainings were held during the year. Participants from all over the country were trained through different programs including in-house, ex-house, mobile, and E-learning training programs. A segment comprising senior management was engaged through a Management Development Program as part of the Talent Management Scheme of the Bank.<br><br>Detailed analysis covering performance and achievements of respective groups against their targets for 2019 is included in the Groups' review section of this annual report.

Sources of Information and Assumptions used for Projections and Forecast

The Bank gathers and compiles internal business data, external economic indicators and industry specific analysis from various sources and utilizes in-house developed tools and functional expertise to process these items through a calculation; in turn laying the foundation for its operational and financial forecasts and projections.

The Bank assumes that discount rate cycle has bottomed and expects it to slightly increase after a period of pause. With respect to PKR, we expect it will devalue by further 5%; however, the volatility observed in 2020 is not expected to be repeated and the exchange rate movement will be comparatively stable. With regards to overall economic activity, results from economic actions undertaken by the Government will positively impact overall economic cycle in the country. The recovery is evident, as large-scale manufacturing has increased, current account surplus has been achieved, and exports have shown recovery in recent months.

Our Response to Critical Challenges and Uncertainties

MCB remains well poised to respond to all critical challenges and uncertainties emanating from the realization of various systematic and idiosyncratic risks by capitalizing on its stable funding structure, ample liquidity buffers, resilient capital base and a pragmatic business strategy.

During the year 2020, proactively monitoring the scenario and analyzing the ever changing and dynamic situation (both domestically and internationally) amidst the COVID-19 outbreak, the Bank exhibited its institutional readiness by successfully invoking its Business Continuity Plan (BCP) for all critical areas; while simultaneously supporting Governments key pandemic responses for credit extension and provision of uninterrupted and essential banking services to the general public.

For details on Bank's readiness to respond to critical challenges and uncertainties, please refer to the Risk Management Framework, Business Continuity Management and Pandemic Recovery Plan in the Annual Report.

Graphical Presentation of Financial Statements

Statement of Financial Position

(Rupees in Million)

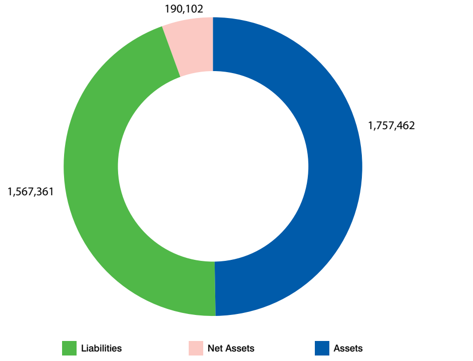

Variance from YE 19

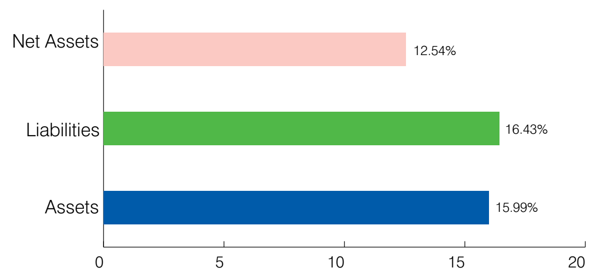

Profit & Loss Account

(Rupees in Million)

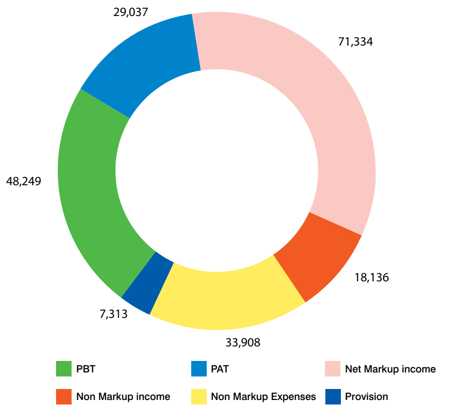

Variance from 2019

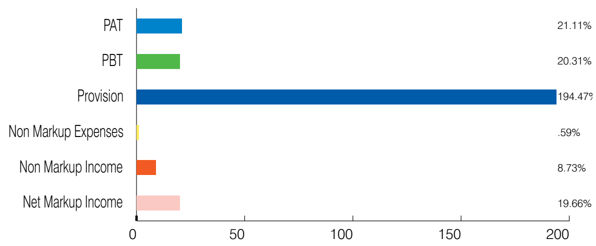

Cash Flows 2020

(Rupees in Million)

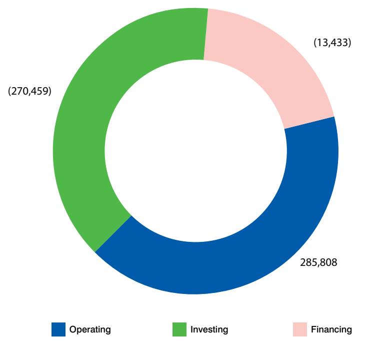

Cash Flows 2019

(Rupees in Million)

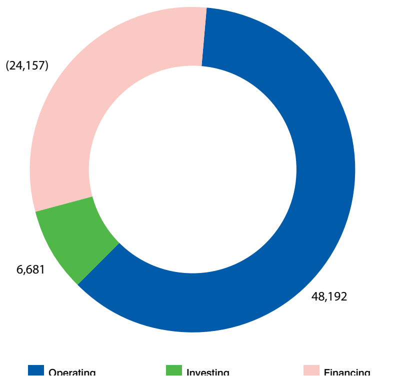

MCB
Bank for Life

Analyses of Financial Performance

Gross markup income reported a decrease of Rs. 2.216 billion for the year 2020 when compared with 2019. Income on advances decreased by Rs. 14.451 billion, primarily on account of decline in yield by 236bps coupled with lower average advances volume of Rs. 30.741 billion; whereas, volumetric growth of Rs. 195.780 billion in average investments resulted in increased markup income on investments which grew by Rs. 16.502 billion.

Rs in million

Mark-Up/Return/ Interest Earned	2020	2019	Variance Amount	Variance % age
Loans and advances	42,879	57,330	(14,451)	-25%
Investments	91,983	75,481	16,502	22%
Lendings to financial institutions	998	4,982	(3,984)	-80%
Balances with banks	215	499	(284)	-57%
	136,075	138,292	(2,217)	-2%

The Bank reported a decrease of Rs. 13.935 billion over last year in markup expense. Mark up expense on deposits decreased by Rs. 10.249 billion, whereas markup expenses on borrowings decreased by 2.825 billion. Significant drop of 625bps in the discount rate during the year resulted in a sharp decrease in deposit cost by 146bps as compared to the last year.

Rs in million

Mark-Up/Return/Interest Expensed	2020	2019	Variance Amount	Variance % age
Deposits	55,095	65,344	(10,249)	-16%
Borrowings	6,152	8,977	(2,825)	-31%
Subordinated debt	–	214	(214)	-100%
Cost of foreign currency swaps	2,387	3,009	(622)	-21%
Unwinding cost of liability against right-of-use assets	1,107	1,132	(25)	-2%
	64,741	78,676	(13,935)	-18%

The non-markup income block of the Bank was reported at Rs. 18.135 billion with major contributions coming in from fee commission, foreign exchange income and gain on sale of securities. Fee income reported a decline of 3% for the year, primarily due to reduced transaction volumes and subdued business activity amidst enforced lockdowns. The captioned decline in fee income was set off by higher capital gains, amounting to Rs. 3.3 billion, realized during the year amidst proactive duration management of the investments portfolio within the evolving yield curve scenario.

Rs in million

Non Mark-Up / Interest Income	2020	2019	Variance Amount	Variance % age
Fee and commission income	10,936	11,288	(352)	-3%
Dividend income	1,210	1,377	(167)	-12%
Foreign exchange income	2,525	2,895	(370)	-13%
Income from derivatives	4	15	(11)	-73%
Gain on securities	3,332	833	2,499	300%
Other income	128	272	(144)	-53%
Total non-markup / interest Income	18,135	16,679	1,455	9%

On the operating expenses (excluding PF reversal) side, despite sustained inflationary pressures, expansion in branch outreach and continued investment in technological infrastructure together with regular performance and merit adjustments for the Human Capital, Bank's strategic focus of balancing short term tactical cost reductions with long term cost initiatives has assisted in containing growth in expenses; in turn registering a decline of Rs. 129 million.

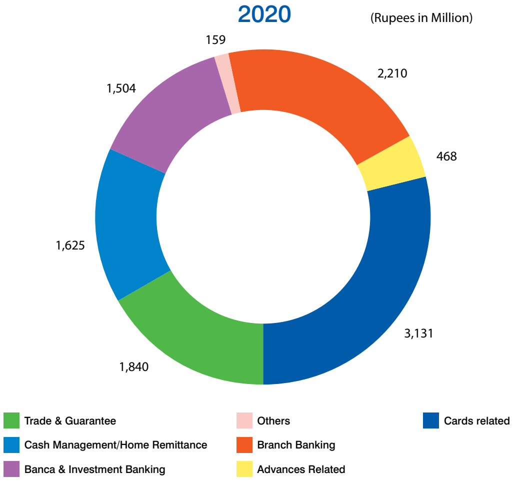

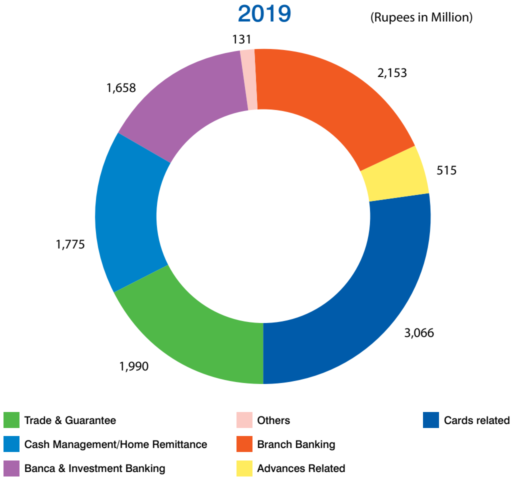

Performance against Targets

During 2020, the Bank has achieved budget of deposits and profit. Further, Bank's current year's performance against targets disclosed in the Annual Report of 2019 is covered in "Forward Looking" section of the Annual Report.

Objectives to Assess Stewardship of Management

The Bank strives to maximize shareholder value through delivering remarkable returns and achieving sustainable performance that exceeds market and shareholder expectations. Key Performance Indicators (KPI's) to measure Bank's performance against its short, medium and long term objectives along with corresponding strategies have been disclosed in the "Strategic and Resource Allocation" section of the Annual Report.

Future Prospects for Profit

Future prospects about Bank's profitability have been covered in the Directors Report and "Forward Looking" section of the Annual Report.

Explanation of negative change in the performance against prior year

For the year under review, profit before tax of the Bank increased by 20% over last year. However Fee commission & FX income declined over the previous year on account of reduced transaction volumes and subdued business activity amidst enforced lockdowns. With the lifting of lockdowns and flattening of COVID-19 infection curve, fee income normalized in Q3 & Q4 2020. Further, in anticipation that portfolios affected by the pandemic might require provisioning once SBP's relaxations and waivers expire in 2021, the management has exercised prudence and booked a general provision of Rs. 4.0 billion during the year under review, hence providing insulation and loss absorption capacity against any risks to asset quality.

Analyses of Non Financial Performance

Non-Financial Highlights

Number of
Branches - 1,429
(Absolute)

Number of
ATMs - 1,434
(Absolute)

Number of
accounts - 8,217,065
(Absolute)

Human
Resources - 13,643
(Absolute)

Training
Participant - 30,163
(Absolute)

Training
days - 25,277
(Absolute)

CSR
Investments - 19,422
Rs. in mln

Human capital

Total number of permanent employees	Investment in training (Rs. in Mln)	Total days of Training	New Recruitments	Promotions
13,643	36.51	25,277	1,430	2,356

Our employees, numbering 13,643 receive well remunerated, secure and satisfying employment with generous retirement benefits.

Our strategy is to align what is best for the employees with what is best for the Bank. Our performance management system has been designed to motivate employees to pursue goals that will enable the Bank to achieve its strategic objectives. Our development and training activities also contribute to the same objective in the longer term. Thus, we have built a performance-based culture that will support both short term and long-term value creation.

Our human resources remain the key asset to our success and growth which is evident from the below mentioned analysis.

		2020	2019
Staff strength	Absolute	13,643	13,596
New recruitments	Absolute	1,430	2,235
Average number of employees	Absolute	13,345	13,568
Promotions	Absolute	2,356	1,978
Investment in training	Rs. In Mln	36.51	57.23
Number of training participants	Absolute	30,163	30,442
Training days	Absolute	25,277	30,255

Manufactured Capital

Capital expenditures on physical & digital infrastructure	Branches excluding sub-branches	ATMs	Internet Banking customers	Mobile Banking customers
PKR 3,090 million	1,429	1,434	198,939	1,396,475

Manufactured capital consists of our physical branch network and other tangible and intangible items that support our operations outreach such as equipment, IT systems and network.

During the year, Bank has expanded its network by 19 branches. In 2020, account base of the Bank expanded to 8.2 million accounts.

The banking model is in gradual transformation from traditional banking to digital era. Hence, to cater to the growing segment of millennials among our customers, the Bank has been continuously investing on the digital banking platforms. We are increasing our digital touch points on a gradual pace, providing our customers transactional convenience while ensuring financial security.

		2020	2019
Capital expenditure	Rs. In Mln	3,090	5,022
Branches	Absolute	1,429	1,410
ATMs	Absolute	1,434	1,360
Internet Banking	Absolute	198,939	180,326
Mobile Banking	Absolute	1,396,475	1,909,712

Intellectual capital

Cumulative service experience of more than 73 years	Strong Governance	One of the most valuable brand

Intangibles associated with the Bank – culture, ethics, values, organizational knowledge, systems, procedures and brand value. These intangibles, while not reflected in the balance sheet, are indeed the real assets of the Bank. They permeate the Bank's operations at all levels – whether it is high level decision-making or day-to-day functions.

In 2020 we have focused on following points to enhance of our intellectual capital:

- Explore customers' views and expectations with regard to selected Deposit products on features, processes and service delivery
- Evaluate customer satisfaction to understand the service level of the Bank
- Explore the new trends in consumer banking to ascertain future banking preferences

During the year, the total investment on intangible assets is Rs. 298.880 million as compared to Rs. 625.800million in FY19.

Social and Relationship Capital

No. of accounts	Dividends to shareholders (Rs. in Mln)	CSR funds (Rs. In Mln)
8,217,065	23,701	19,425

The Pakistan banking industry is more competitive than it has ever been, and factors such as customer service and convenience are distinguishing features that customers look for. With a strong network of branches across the Country, MCB maintains strength in geographic reach that few can match. Analysis of social and relationship capital as compared to prior year is as follows:

		2020	2019
No. of accounts	Absolute	8,217,065	8,223,038
Dividends to shareholders	(Rs. In Mln)	23,701	20,146
CSR funds	Rs. in Mln		
Education Allowance		33	38
Staff Capacity Building & Trainings		37	57
Employee Hajj Expense		–	13
Contribution to National Exchequer		19,212	16,125
Contribution To Staff Welfare Fund		5	5
Donation		113	-
Plantation		22	24
Total		19,422	16,262

Maturities of Assets and Liabilities

Rs. in Million

	2020	Upto 3M	3M to 1Y	1Y to 3Y	3Y to 5Y	5Y & above
**Assets**						
Cash and balances with treasury banks	122,181	122,181	–	–	–	–
Balances with other banks	24,030	24,030	–	–	–	–
Lendings to financial institutions	17,139	17,139	–	–	–	–
Investments - net	1,015,869	503,984	180,703	103,484	36,564	191,134
Advances - net	462,942	101,814	76,738	146,477	82,944	54,969
Fixed assets	58,028	984	2,951	7,091	4,047	42,955
Intangible assets	938	178	535	226	–	–
Deferred tax assets	2,783	694	2,089	–	–	–
Other assets - net	56,334	34,151	2,665	8,680	10,838	–
	1,760,245	805,155	265,681	265,958	134,393	289,058
**Liabilities**						
Bills payable	23,981	23,981	–	–	–	–
Borrowings	164,001	129,481	6,858	10,954	4,832	11,876
Deposits and other accounts	1,289,502	120,576	114,596	632,303	316,784	105,243
Deferred tax liabilities	9,758	251	1,007	2,566	2,432	3,502
Other liabilities	82,901	34,090	17,673	11,837	15,388	3,913
	1,570,143	308,379	140,134	657,660	339,436	124,534

*Based on expected maturities

Key Interest Bearing Assets and Liabilities

	2020			2019		
	Avg. Vol (Mln)	Effective interest rate %	Interest (Mln)	Avg. Vol (Mln)	Effective interest rate %	Interest (Mln)
**Interest Earning Assets**						
Lendings to Financial Institutions	13,735	7.27	998	45,660	10.91	4,982
Gross Advances (excluding NPLs)	459,219	9.34	42,879	489,960	11.70	57,330
Gross Investments (excluding equity investments)	854,012	10.77	91,983	658,232	11.47	75,481
**Interest Bearing Liabilities**						
Deposits (excl. current deposits)	765,997	7.19	55,095	693,156	9.43	65,344
Borrowings	116,155	5.30	6,152	114,512	7.84	8,977

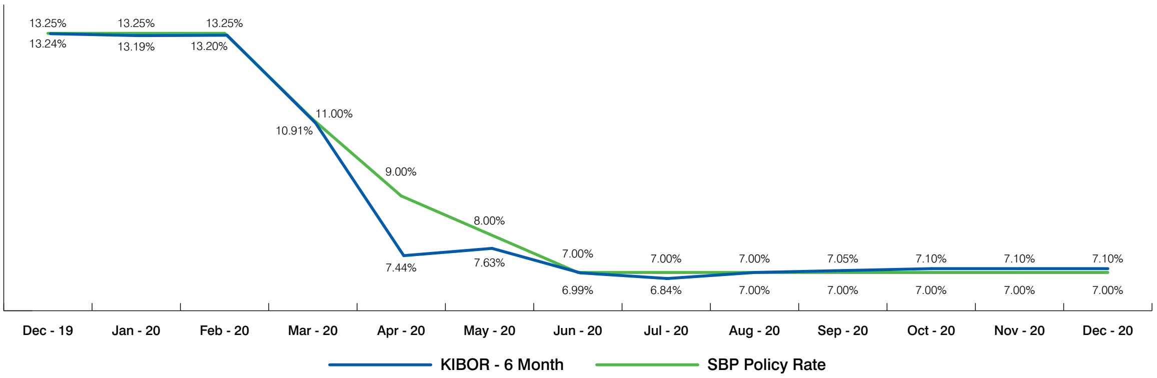

Non Performing Loans

Rs. in Mln

	2020		2019		Variance		2020
	NPLs	Provision	NPLs	Provision	NPLs	Provision	Coverage
**Categorywise**							
OAEM	44	2	124	4	-64.8%	-43.8%	4.6%
Substandard	214	53	616	153	-65.2%	-65.5%	24.6%
Doubtful	285	142	2,702	1,351	-89.5%	-89.5%	50.0%
Loss	50,646	44,946	45,982	40,427	10.1%	11.2%	88.7%
Total	51,189	45,143	49,424	41,934	3.6%	7.7%	88.2%

	NPLs	Provision	NPLs	Provision	NPLs	Provision	Coverage
**Groupwise**							
Commercial & Consumer	6,449	6,100	7,985	6,797	-19.2%	-10.3%	94.6%
Corporate	4,695	4,692	3,230	1,993	45.3%	135.4%	99.9%
Overseas	8,444	3,470	8,190	3,353	3.1%	3.5%	41.1%
Others	31,601	30,880	30,019	29,791	5.3%	3.7%	97.7%
	51,189	45,143	49,424	41,934	3.6%	7.7%	88.2%

Non Performing Loans (2015-2020)

(Rupees in Billion)

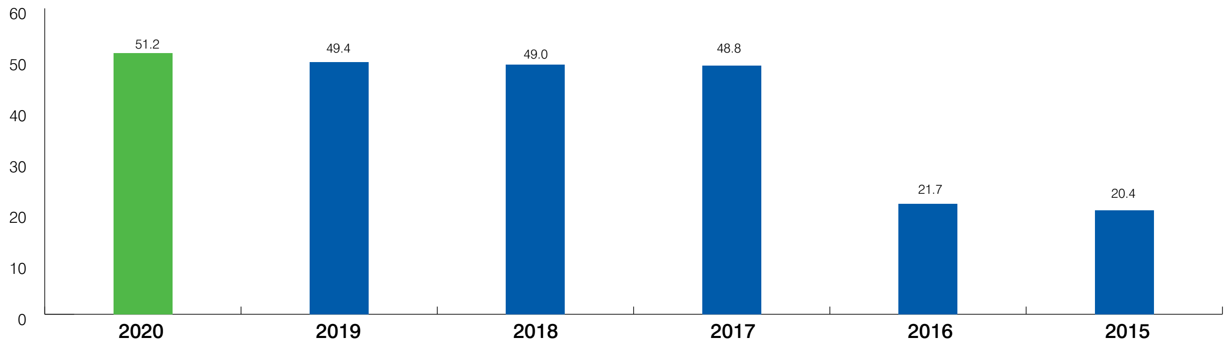

*Infection and Coverage Ratios (2015-2020)

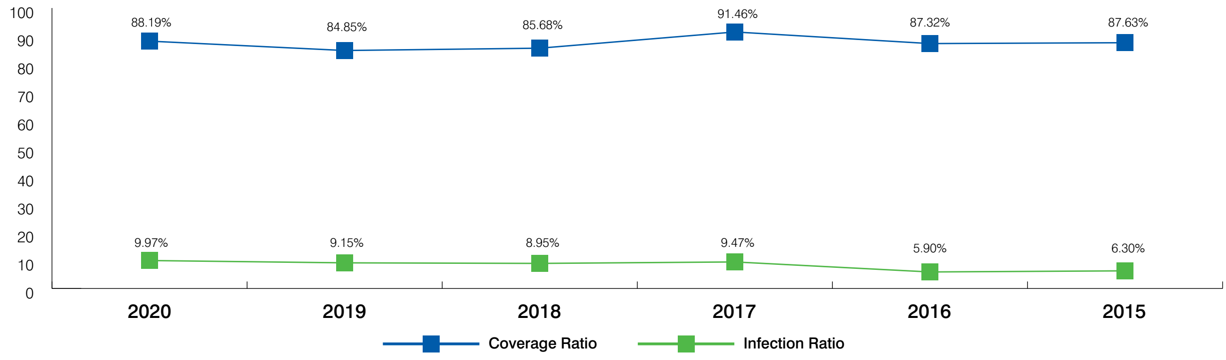

*Based on specific provision only

Deposits & Advances - Sector wise

December 31, 2020

Sector	Deposits	Rupees in Million<br>Advances
Agriculture, forestry and fishing	25,506	5,122
Construction	44,354	18,082
Electricity, gas, steam and air conditioning supply	55,408	37,521
Financial	31,071	17,534
Individuals	829,945	43,906
Manufacture of basic metals and metal products	2,101	14,914
Manufacture of cement	5,275	9,742
Manufacture of chemicals and pharmaceutical products	14,674	42,385
Manufacture of food & beverages products	30,012	39,632
Manufacture of sugar	5,306	33,283
Manufacture of textiles	7,122	76,794
Transportation and storage	2,740	53,062
Telecommunications	2,053	18,287
Services	38,947	10,353
Wholesale and retail traders	49,487	53,692
Others	145,500	39,240
	**1,289,502**	**513,550**

Deposits

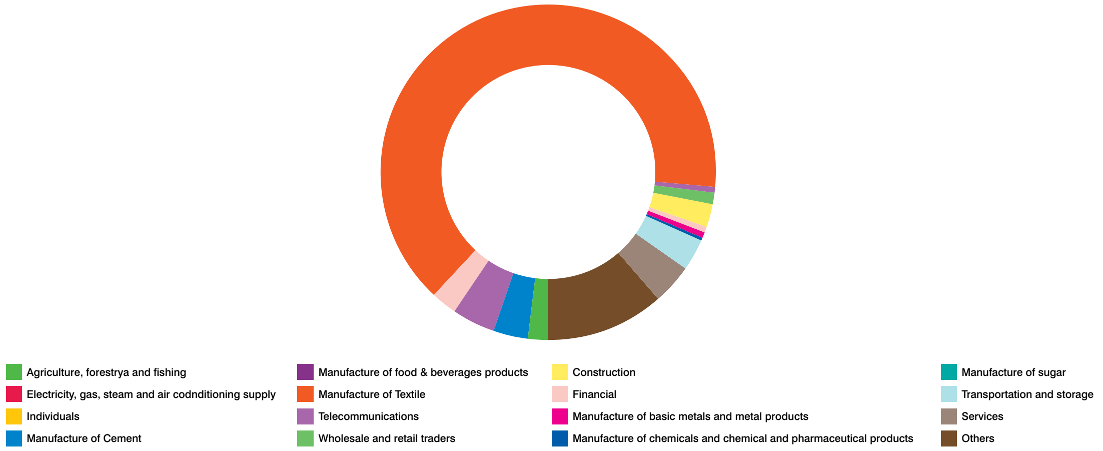

Advnaces

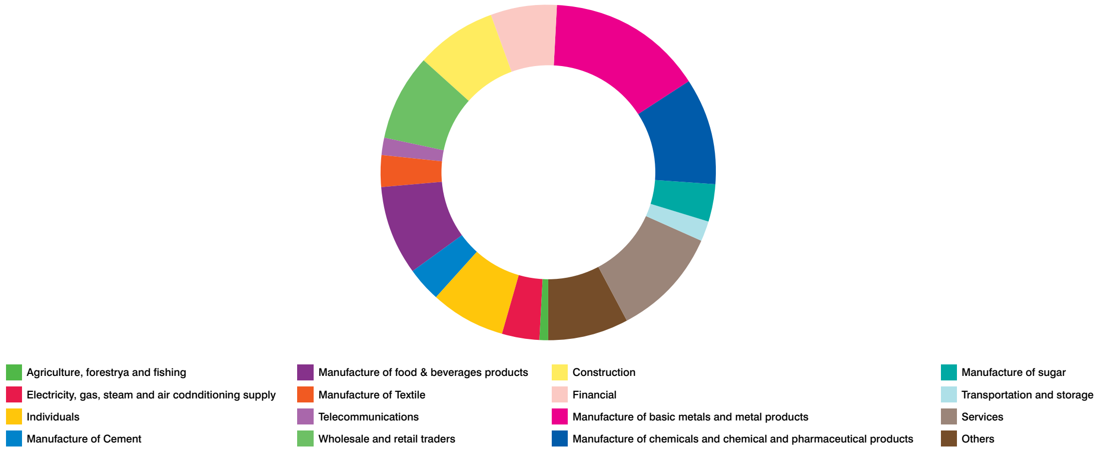

Deposits & Advances - Group wise

Rs. in Billion

	Groupwise Deposits				Groupwise Gross Advances			
	2020	2019	Variance		2020	2019	Variance	
			Amount	%			Amount	%
Commercial & Consumer	1,168.6	1,034.2	134.4	13.00%	113.7	119.3	-5.6	-4.69%
Corporate	66.3	55.5	10.8	19.51%	338.8	355.2	-16.4	-4.62%
Overseas	54.6	55.0	(0.4)	-0.75%	30.1	33.4	-3.2	-9.63%
Others	-	-	-	-	30.9	32.2	-1.3	-3.90%
Total	1,289.5	1,144.7	144.8	12.65%	513.5	540.0	-26.5	-4.90%

CASA Mix

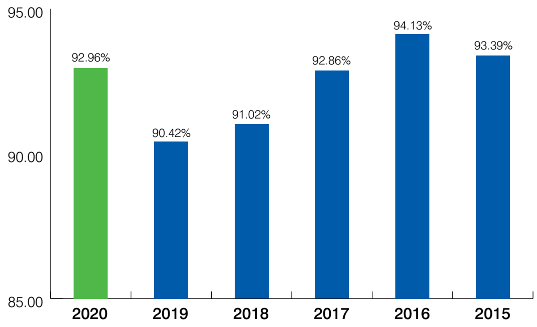

Weighted Average Cost of Deposits

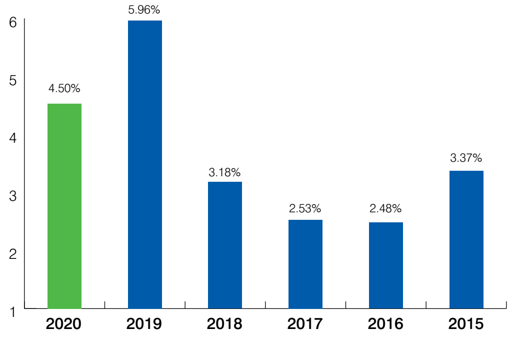

Weekly Trend of MCB Deposits and Advances - 2020

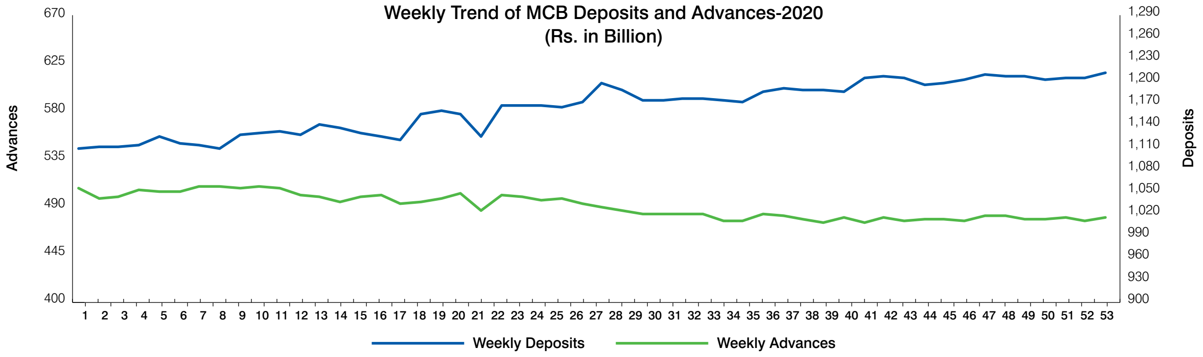

MCB's Industry share in Deposits and Advances - 2020

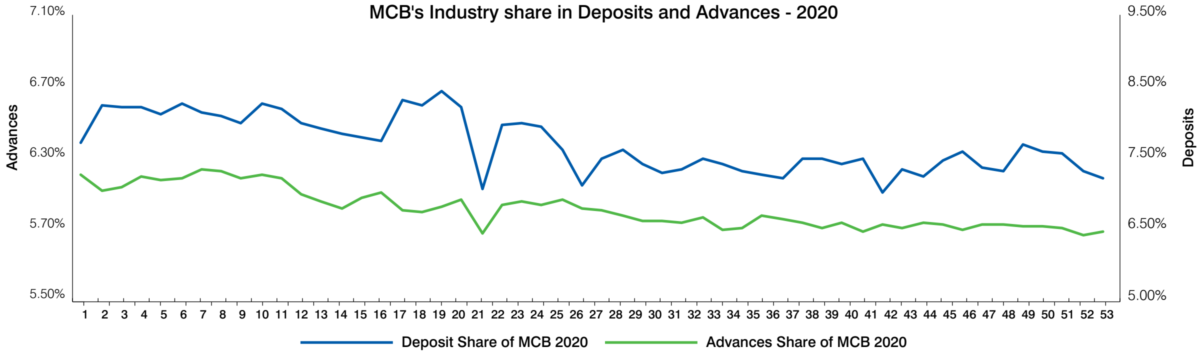

Investments

Top 10 Listed Equity Holdings as on December 31, 2020

Company Name	Total Shares (number)	Book Value (Rs. Mln)	Market Value (Rs. Mln)
Oil & Gas Development Company Limited	11,115,535	1,814	1,153
Kot Addu Power Company	15,662,000	1,344	426
Fauji Fertilizer Company Limited	10,844,000	1,195	1,177
Pakistan Petroleum Limited	6,946,102	933	627
Habib Bank Limited	4,667,700	813	617
Millat Tractors Limited	838,950	780	918
United Bank Limited	4,613,107	774	581
HBL Growth Fund-Class ‘’A”	43,482,858	744	370
National Bank of Pakistan	14,208,500	739	610
Engro Fertilizers Limited	8,936,000	718	565

Investment in Associates and Subsidiaries

Company Name	Holding	Total Shares (numbers)	Book Value (Rs. Mln)
**Associates**			
Adamjee Insurance Company Limited	20.00%	70,000,000	647.88
Euronet Pakistan (Pvt.) Limited	30.00%	52,521	52.52
**Subsidiaries**			
MCB - Arif Habib Savings & Investments Limited	51.33%	36,956,768	320.12
MCB Islamic Bank Limited	100.00%	1,155,000,000	11,550.00
MCB Non Bank Credit Organization closed joined stock company	99.94%	4,281,105	448.19

Category of Investments (2015-2020) (Rupees in Billion)

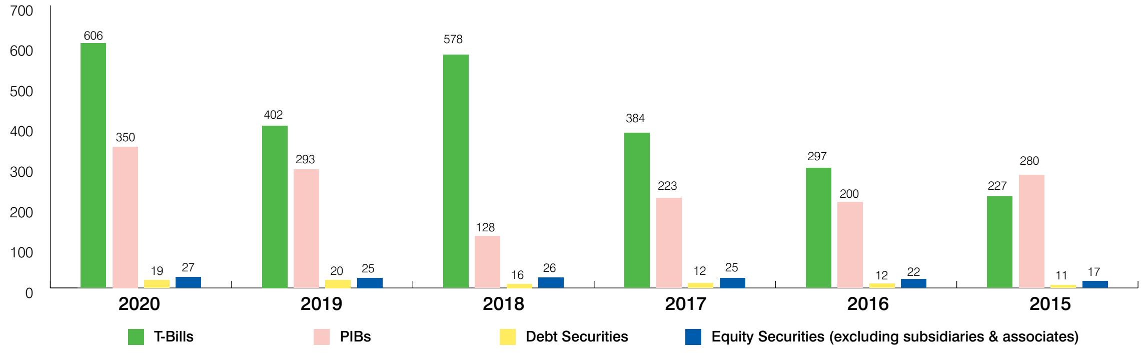

Investments to Deposits Ratio (2015-2020)

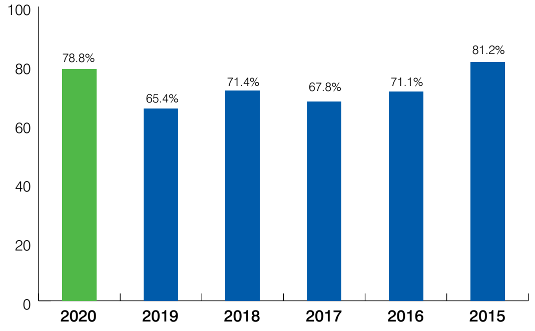

Investments to Total Assets (2015-2020)

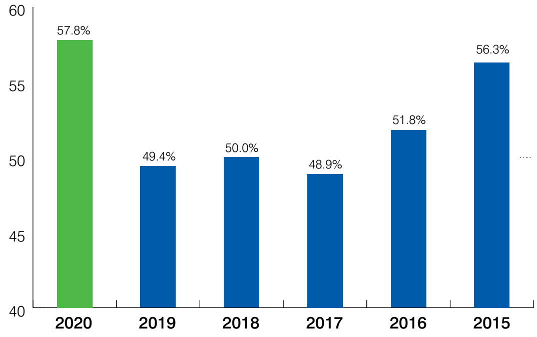

Capital Structure

	2020	Rupees in Million<br>2019
**Capital Structure**		
**Tier 1 Capital**		
Shareholders equity /assigned capital	11,851	11,851
Share premium	23,751	23,751
Reserves	53,160	50,257
Unappropriated profits	69,835	55,777
	158,597	141,636
**Deductions:**		
Book value of intangible and advances given for intangible	938	958
Defined benefit pension fund assets - net	2,191	2,343
Other deductions	2,566	2,078
	5,695	5,379
**Total Tier 1 capital**	152,901	136,257
**Tier 2 Capital**		
General provisions subject to 1.25% of total risk weighted assets	5,465	1,424
Revaluation reserves	27,165	23,255
Foreign exchange translation reserves	2,876	2,675
	35,507	27,354
**Deductions:**		
Other deductions	–	–
**Total Tier 2 Capital**	35,507	27,354
**Total Regulatory Capital Base**	**188,409**	**163,611**
**Capital Adequacy**		
**Risk Weighted Assets**		
Credit Risk	635,599	638,493
Market Risk	122,604	108,276
Operational Risk	139,735	120,887
**Total RWA**	**897,938**	**867,656**
**Capital Adequacy Ratio**		
Total eligible regulatory capital held	188,409	163,611
Total Risk Weighted Assets	897,938	867,656
**Capital Adequacy Ratio**	**20.98%**	**18.86%**

Capital Adequacy Ratio (2015-2020)

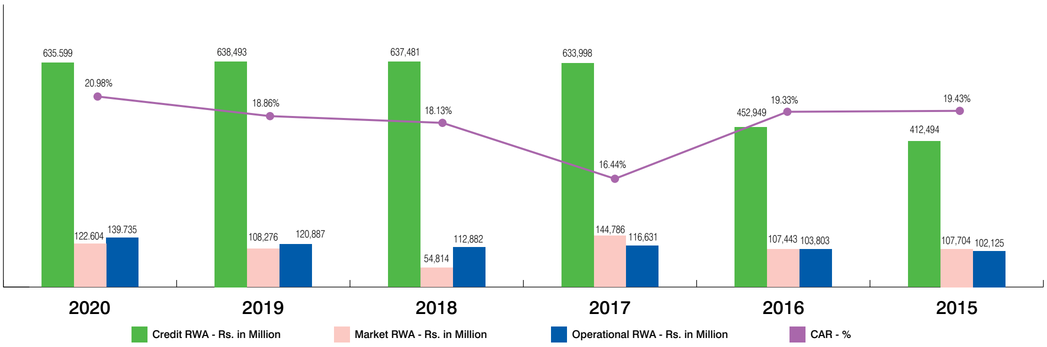

Quarterly Performance - 2020 & 2019

Rupees in Million

	2020				2019			
	4th Quarter	3rd Quarter	2nd Quarter	1st Quarter	4th Quarter	3rd Quarter	2nd Quarter	1st Quarter
**Profit & Loss Account**								
Mark-up earned	29,040	31,824	36,112	39,100	39,842	39,317	31,751	27,382
Mark-up expensed	(13,052)	(12,490)	(16,407)	(22,792)	(23,220)	(24,123)	(17,430)	(13,903)
Net mark-up income	15,988	19,333	19,705	16,308	16,622	15,194	14,321	13,479
Non-mark-up income	4,577	6,476	3,195	3,888	5,226	3,493	4,445	3,514
Total Income	20,565	25,810	22,900	20,195	21,849	18,688	18,766	16,993
Non-mark-up expenses	(8,465)	(8,565)	(8,290)	(8,588)	(8,385)	(8,574)	(8,353)	(8,398)
Profit before provisions	12,100	17,245	14,610	11,607	13,464	10,114	10,413	8,595
Provisions & write off	(2,204)	(1,145)	(3,221)	(742)	(867)	(856)	(1,244)	484
Profit before taxation	9,895	16,099	11,389	10,865	12,596	9,257	9,169	9,079
Taxation	(3,793)	(6,374)	(4,699)	(4,346)	(4,906)	(3,646)	(3,574)	(3,999)
**Profit after taxation**	6,102	9,725	6,690	6,519	7,690	5,612	5,595	5,080
**Statement of Financial Position**								
**Assets**								
Cash and balances with treasury banks	122,181	127,513	144,168	94,130	132,705	106,039	136,930	107,291
Balances with other banks	24,030	29,693	14,701	15,170	12,542	13,882	15,521	11,889
Lendings to financial institutions	17,139	2,140	2,057	9,577	1,090	6,102	4,307	201,883
Investments	1,015,869	964,412	928,708	836,660	748,765	864,430	793,510	450,253
Advances	462,942	445,039	460,611	480,925	496,679	490,255	514,143	500,205
Fixed assets	58,028	57,739	57,586	58,020	58,271	47,614	47,489	41,779
Intangible assets	938	836	881	941	958	912	627	677
Other assets	56,334	47,498	58,893	59,795	64,143	52,259	60,870	51,086
	1,757,462	1,674,869	1,667,604	1,555,216	1,515,152	1,581,493	1,573,398	1,365,064
**Liabilities**								
Bills payable	23,981	9,951	10,504	7,972	11,822	9,315	10,514	10,378
Borrowings	164,002	112,373	113,230	88,652	89,506	200,398	183,982	70,235
Deposits and other accounts	1,289,502	1,274,870	1,274,682	1,184,139	1,144,763	1,145,140	1,148,631	1,066,013
Sub-ordinated loan	-	-	-	-	-	-	-	3,891
Deferred tax liabilities	6,975	6,497	11,804	10,425	5,851	2,327	820	2,203
Other liabilities	82,901	85,803	72,498	85,382	94,296	70,627	79,072	61,905
	1,567,361	1,489,494	1,482,719	1,376,570	1,346,237	1,427,807	1,423,018	1,214,626
**Net assets**	190,102	185,376	184,886	178,646	168,915	153,686	150,380	150,438
**Represented by:**								
Share capital	11,851	11,851	11,851	11,851	11,851	11,851	11,851	11,851
Reserves	80,696	80,558	79,712	78,843	77,591	76,968	76,833	74,984
Unappropriated profit	69,835	62,924	54,122	55,742	55,777	53,486	53,162	53,377
Surplus on revaluation of assets - net of tax	27,720	30,043	39,201	32,211	23,695	11,382	8,534	10,227
	190,102	185,376	184,886	178,646	168,915	153,686	150,380	150,438

Quarterly PBT (Rupees in Billion)

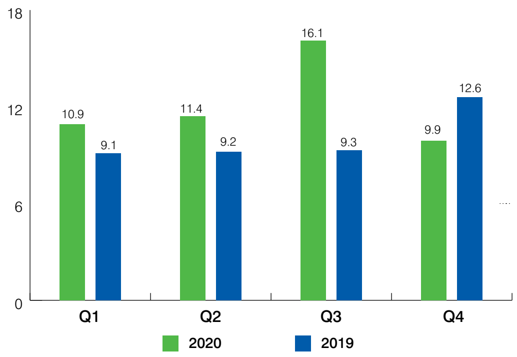

Quarterly NIM (Rupees in Billion)

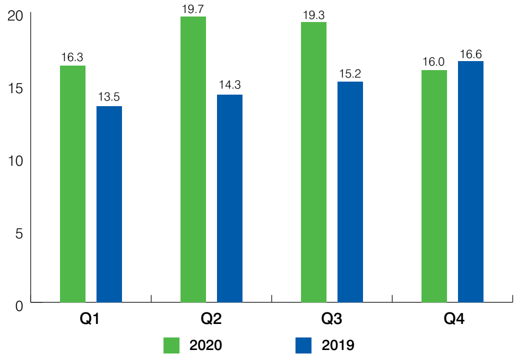

Quarterly Performance Analysis - 2020 & 2019

Quarter	Net Interest Income	Non Interest Income	Non Markup Expenses	Profit
Quarter 1	During the 1st quarter of 2020, Net Interest Income (NII) of the Bank grew by 21% and was reported at Rs. 16.308 billion. Markup income on investments depicted a significant increase of 80% over the corresponding period due to volumetric increase in average balances and higher market yields amidst a rising yield curve; in turn contributing 63% to the total gross markup income and amounting to Rs. 24.598 billion. The income on advances contributed 36% to the total mix and amounted to Rs. 14.221 billion.<br><br>On the markup expense side, Rs. 19.669 billion was reported on account of markup on deposits; representing an increase of 56% over the corresponding period owing primarily to higher minimum deposit rates and volumetric growth in average outstanding balances."	Non Mark up Income for the 1st quarter was reported at Rs. 3.888 billion against Rs. 3.514 billion reported in the corresponding period; representing an increase of 11%. FX income contributed 76% to the total increase whereas higher gain on sale of securities contributed 16% to the increase.	Non Markup expenses grew by 2% over the corresponding period. Despite surge in inflationary pressures, the Bank was able to contain growth in operating expenses and reported a net decrease of Rs. 24 million as compared to corresponding period. The control in the operating expense base is resultant of the expense management drive undertaken by the Bank in 2019.	Profit before tax grew by 20% in 1st quarter primarily on the back of improving margins. Profit after tax grew by 28% and was recorded at Rs. 6.519 million.
Quarter 2	NII during the 2nd quarter was reported at Rs. 19.705 billion; higher by 38% as compared to the corresponding period on the back of improving net interest margins. Gross markup income increased by 14%, owing to rising income on investments, whereas the gross mark up expense declined slightly by 6% as the easing monetary policy stance adopted by the SBP amidst the COVID-19 outbreak led to a lowering of the minimum deposit rates.	Non Mark up Income for the 2nd quarter was reported at Rs. 3.195 billion; posting a decline of 28% over the corresponding period. The decline was mainly on account of lowering dividend and foreign exchange income amounting to Rs. 310 million and Rs. 703 million respectively (Q2 2019: 521 million and 1,286 million) as realization of various systematic and idiosyncratic factors amidst the COVID-19 outbreak, including branch closures and a slowing economic activity, impacted banks income stream.	Non markup expenses closed the period in line with the 1st quarter; witnessing a slight decline of 0.76%.	Profit before tax for the 2nd quarter was reported at Rs. 11.389 billion as compared to Rs. 9.169 billion for the corresponding period; higher by 24% mainly due to increasing NII and sustained flat trend in non markup expenses. The increase was offset by the recording of higher general provision against advances and a declining non markup income. Profit after tax hence grew by 19.6% and was recorded at Rs. 6.690 billion.
Quarter 3	NII during the 3rd quarter was reported at Rs. 19.333 billion; higher by 27% as compared to the corresponding period. Amidst a declining yeild curve, the repricing lag between earning assets and liabilities contributed to the improved net interest margins.	Non Mark up Income posted a significant increase of 85%, amounting to Rs. 2.982 billion, as proactive duration management of the investment portfolio resulted in capital gains of Rs. 2.861 billion recognized during the quarter under review.	Non markup expenses closed the period in line with the 2nd quarter; witnessing a slight decline of 0.1%.	Profit before Tax has increased to Rs. 16.099 billion as compared to Rs. 9.257 billion in the comparitive period. The significant factors contributing to the higher profit in 3rd quarter was increasing NII and realization of capital gains on the investment portfolio. The increase was slightly offset by higher provisioning against advances and investments. Profit after tax hence grew by 73% and was recorded at Rs. 9.725 billion.
Quarter 4	NII during the 4th quarter is 4% lower than the NII reported in the comparitive period. The decline is on account of lowering interest margins as the benefits of repricing lag between earning assets and liabilities subsided. Gross markup income declined by 27% whereas the markup expense declined by 44%.	Non Mark up Income declined to Rs. 4.577 billion compared to Rs. 5.226 billion reported in the corresponding period mainly on account of lower capital gains; hence depicting a decline of 12%.	Non markup expenses posted a slight increase of 1% and closed the quarter at Rs. 8.465 billion.	Declining NII and non markup income resulted in decrease in reported profit before tax; which closed the quarter at Rs. 9.895 billion against Rs. 12.596 billion reported in the corresponding period. Profit after tax hence reached Rs. 6.102 billion with a reported decline of 21%

Non Markup Expense (Rupees in Billion)

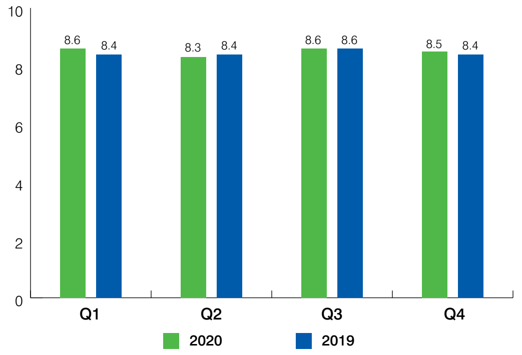

Non Markup Income (Rupees in Billion)

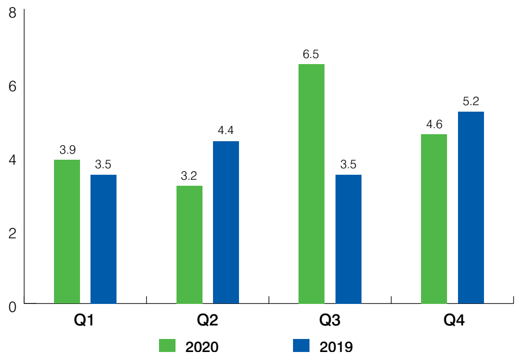

MCB
Bank for Life

Six Years' Financial Performance / Financial Ratios 2015 - 2020

		2020	2019	2018	2017	2016	2015
**PROFIT AND LOSS ACCOUNT**							
Mark-up/ return earned	Rs. Mln	136,076	138,292	83,319	74,091	67,400	80,393
Mark-up/ return expensed	" "	64,741	78,676	37,305	31,429	23,586	31,077
Fund based income	" "	71,334	59,616	46,014	42,662	43,814	49,316
Fee, Commission, brokerage & FX income	" "	13,594	14,469	14,625	11,435	9,040	10,871
Dividend and capital gains	" "	4,542	2,210	2,573	6,682	7,135	5,695
Total income	" "	89,470	76,295	63,212	60,780	59,989	65,882
Operating expenses	" "	33,908	33,709	32,902	28,721	22,989	22,895
Operating profit before tax and provision	" "	55,562	42,586	30,310	32,059	36,999	42,987
Provisions / write-offs	" "	7,313	2,484	(1,753)	1,045	925	659
Profit before tax	" "	48,249	40,102	32,064	31,014	36,075	42,329
Profit after tax	" "	29,037	23,977	21,360	22,459	21,891	25,546
Cash Dividends	" "	23,701	20,146	18,961	18,673	17,808	17,808
**Statement of Financial Position**							
Authorised capital	" "	15,000	15,000	15,000	15,000	15,000	15,000
Paid up capital	" "	11,851	11,851	11,851	11,851	11,130	11,130
Reserves	" "	80,696	77,591	74,148	70,866	53,347	51,309
Unappropriated Profit	" "	69,835	55,777	53,532	53,776	53,469	50,747
Shareholder's equity	" "	162,382	145,219	139,531	136,493	117,946	113,186
Surplus on revaluation of assets - net of tax	" "	27,720	23,695	9,747	17,073	23,680	24,616
Net Assets	" "	190,102	168,915	149,278	153,566	141,627	137,802
Total Assets	" "	1,757,462	1,515,152	1,498,130	1,343,238	1,072,365	1,004,410
Earning Assets	" "	1,544,536	1,294,096	1,343,378	1,175,352	911,163	872,594
Gross Advances	" "	513,550	540,037	546,792	515,058	367,678	322,529
Advances - net of provisions	" "	462,942	496,679	503,581	469,356	348,117	304,122
Non-Performing Loans (NPLs)	" "	51,189	49,424	48,956	48,753	21,688	20,368
Investments	" "	1,015,869	748,765	749,369	656,964	555,929	565,696
Total Liabilities	" "	1,567,361	1,346,237	1,348,852	1,189,672	930,739	866,608
Deposits & other accounts	" "	1,289,502	1,144,763	1,049,038	968,483	781,430	696,805
Current & Saving Deposits (CASA)	" "	1,198,785	1,035,063	954,813	899,364	735,550	650,739
Borrowings	" "	164,002	89,506	216,019	133,070	74,515	118,040
Interest bearing Liabilities	" "	964,119	809,717	867,048	728,361	557,913	557,056
Contingencies and Commitments	" "	714,038	851,147	584,434	448,135	307,566	320,888
**Profitability ratios**							
Profit before tax ratio	%	35.46%	29.00%	38.48%	41.86%	53.52%	52.65%
Gross Yield on Average Earning Assets	" "	9.59%	10.49%	6.41%	7.10%	7.56%	9.51%
Gross Yield on Avg. Earning Assets (incl. dividend & capital gains)	" "	9.91%	10.65%	6.61%	7.74%	8.36%	10.18%
Gross Spread	" "	52.42%	43.11%	55.23%	57.58%	65.01%	61.34%
Non interest income to total income	" "	20.27%	21.86%	27.21%	29.81%	26.96%	25.14%
Return on average equity (ROE)	" "	18.88%	16.84%	15.48%	17.65%	18.94%	23.21%
Return on average assets (ROA)	" "	1.77%	1.59%	1.50%	1.86%	2.16%	2.63%
Return on Capital Employed (ROCE)	" "	18.88%	16.84%	15.48%	17.65%	18.94%	23.21%
Cost to income ratio	" "	36.49%	42.82%	50.77%	46.00%	36.80%	32.17%
Admin Exp to Profit before Tax	" "	68.37%	82.58%	98.07%	91.99%	61.19%	51.90%
**Investment ratios\Market Ratios**							
Earnings per share (after tax)	Rs.	24.50	20.23	18.02	19.56	19.67	22.95
Earnings per share (before tax)	" "	40.71	33.84	27.06	27.02	32.41	38.03
Breakup value per share							
- without surplus on revaluation of fixed assets & investments	" "	137.02	122.54	117.74	115.18	105.97	101.69
- without surplus on revaluation of fixed assets	" "	144.35	126.47	115.68	119.17	116.10	114.09
- with surplus on revaluation of fixed assets & investments	" "	160.42	142.54	125.97	129.59	127.24	123.81
- with surplus on revaluation of fixed assets & investments & investment in related party at fair / market value	" "	162.77	144.89	128.41	132.90	132.90	128.63
Cash Dividend	%	200%	170%	160%	160%	160%	160%
Dividend Yield ratio (based on cash dividend)	" "	10.79%	8.30%	8.27%	7.54%	6.73%	7.38%
Dividend Payout ratio	" "	81.62%	84.02%	88.77%	81.86%	81.35%	69.71%
Price to book value ratio	Times	1.35	1.67	1.64	1.84	2.24	2.14
Price to earning ratio	" "	7.56	10.13	10.74	10.85	12.09	9.45
Dividend cover ratio	" "	1.23	1.19	1.13	1.18	1.23	1.43

Six Years' Financial Performance / Financial Ratios 2015 - 2020

		2020	2019	2018	2017	2016	2015
**Share Information**							
Market value per share - Dec 31	Rs.	185.28	204.94	193.57	212.32	237.82	216.85
High - during the year	" "	224.53	216.20	236.56	262.10	244.82	338.82
Low - during the year	" "	132.89	154.04	177.16	190.43	190.20	205.34
Market Capitalisation	Rs. Mln	219,568	242,866	229,392	251,612	264,701	241,361
**Asset Quality and Liquidity ratios**							
Gross Advances to deposits ratio	%	39.83%	47.17%	52.12%	53.18%	47.07%	46.29%
Net Advances to deposits ratio	" "	35.90%	43.39%	48.00%	48.46%	44.55%	43.65%
Investments to deposits ratio	" "	78.78%	65.41%	71.43%	67.83%	71.14%	81.18%
Weighted Average Cost of Deposits	" "	4.53%	5.70%	3.18%	2.53%	2.48%	3.37%
CASA to total deposits	" "	92.96%	90.42%	91.02%	92.86%	94.13%	93.39%
NPLs to Gross advances ratio	" "	9.97%	9.15%	8.95%	9.47%	5.90%	6.32%
NPLs to Shareholders Equity	" "	31.52%	34.03%	35.09%	35.72%	18.39%	18.00%
Coverage Ratio (specific provision/ NPLs)	" "	88.19%	84.85%	85.68%	91.46%	87.32%	87.63%
Coverage Ratio (total provision/ NPLs)	" "	98.87%	87.73%	88.26%	93.74%	90.82%	90.37%
Earning assets to total assets ratio	" "	87.88%	85.41%	89.67%	87.50%	84.98%	86.88%
Investments to total assets ratio	" "	57.80%	49.42%	50.02%	48.91%	51.84%	56.32%
Cash & Cash Equivalents to Total Assets	" "	8.30%	9.59%	7.61%	8.16%	7.31%	6.34%
Cash to Current Liabilities	" "	6.30%	5.39%	3.44%	5.07%	7.78%	5.96%
Earning assets to interest bearing Liabilities	Times	1.60	1.60	1.55	1.61	1.63	1.57
Deposits to shareholder equity	" "	7.94	7.88	7.52	7.10	6.63	6.16
Assets to Equity	" "	10.82	10.43	10.74	9.84	9.09	8.87
Current / Quick Ratio	" "	2.38	2.29	1.91	2.01	3.05	2.44
**Risk Adequacy**							
Tier I Capital	Rs. Mln	152,901	136,257	128,999	129,130	111,999	107,067
Total Eligible Capital	" "	188,409	163,611	145,987	147,227	128,968	120,930
Risk Weighted Assets (RWA)	" "	897,938	867,478	805,177	895,415	667,195	622,323
Tier I to RWA	%	17.03%	15.71%	16.02%	14.42%	16.79%	17.20%
RWA to total assets	" "	51.09%	57.25%	53.75%	66.66%	62.22%	61.96%
Capital Adequacy Ratio	" "	20.98%	18.86%	18.13%	16.44%	19.33%	19.43%
Net Return on Average RWA	" "	3.29%	2.87%	2.51%	2.87%	3.40%	4.26%
**Duo Pont Analysis**							
Net Operating Margin	%	32.45%	31.43%	33.79%	36.95%	36.49%	38.78%
Asset Utilization	%	5.47%	5.06%	4.45%	5.03%	5.78%	6.80%
Leverage Ratio / Equity Multiplier	Times	10.64	10.58	10.29	9.49	8.99	8.81
**Industry Share**							
Deposits	%	7.21%	7.82%	7.86%	7.83%	6.98%	7.20%
Advances	" "	6.04%	6.62%	6.93%	7.89%	6.60%	6.58%
Market Capitalisation	" "	16.87%	17.17%	17.85%	14.86%	17.91%	
**Consolidated**							
Total Assets	Rs. Mln	1,891,276	1,612,215	1,585,210	1,389,492	1,097,281	1,020,980
Shareholders' Equity	" "	163,409	145,854	140,196	138,100	120,152	115,253
Net Assets	" "	192,991	171,347	151,323	156,543	145,960	142,177
Profit before tax	" "	49,318	40,154	30,806	30,614	36,721	42,789
Profit after tax	" "	29,562	23,947	20,415	22,048	22,174	25,035
Return on Average Assets	%	1.69%	1.50%	1.37%	1.77%	2.14%	2.55%
Return on Average Equity	" "	19.02%	16.66%	14.60%	16.98%	19.18%	22.12%
Earnings per share	Rs.	24.82	20.14	17.17	19.13	19.82	22.38
Breakup value per share	" "	162.85	144.59	127.69	132.10	131.14	127.74
Capital Adequacy Ratio	%	19.69%	17.84%	17.02%	16.34%	19.68%	20.07%
**Per Branch**							
Gross Advances	Rs. Mln	359.38	383.01	394.23	356.69	297.10	263.72
Deposits	" "	902.38	811.89	756.34	670.69	631.20	569.75
CASA	" "	838.90	734.09	688.40	622.83	594.14	532.08
PBT	" "	33.76	28.44	23.12	21.48	29.14	34.61

Six Years' Non-Financial Performance 2015 - 2020

		2020	2019	2018	2017	2016	2015
No. of accounts	Absolute	8,217,065	8,223,038	7,854,928	7,607,277	6,549,452	6,074,103
No. of branches	"	1,429	1,410	1,387	1,444	1,238	1,223
No. of permanent employees	"	13,643	13,596	12,860	13,155	11,088	10,532
**Digital Banking**							
No. of ATMs	Absolute	1,434	1,360	1,321	1,377	1,191	1,073
No. of Debit cards/smart cards issued during the year	"	577,406	652,440	783,233	772,314	666,999	642,649
**Internet Banking**							
No. of customers	"	198,939	180,326	176,210	163,273	144,069	125,621
No. of transactions	"	691,553	479,278	481,137	509,569	450,333	358,547
Volume of transactions	Rs. Mln	29,200	18,452	14,859	12,306	7,971	6,149
**Mobile Banking**							
No. of customers	Absolute	1,396,475	1,909,712	1,363,304	1,232,258	931,965	656,485
No. of transactions - financial	"	2,793,156	2,074,367	2,354,765	1,689,324	1,487,899	1,377,762
Volume of transactions	Rs. Mln	78,674	50,261	48,623	24,597	15,018	14,842
**Credit Cards**							
No. of new issuance	Absolute	13,944	16,907	15,245	13,006	11,060	10,647
No. of customers	"	84,542	83,070	77,190	70,246	64,075	59,343
Total spend (transaction volume)	Rs. Mln	8,327	8,927	7,597	7,054	5,967	5,079
**Auto Loan**							
No. of Loans disbursed	Absolute	5,549	5,999	8,266	8,977	6,751	5,565
Outstanding Volume	Rs. Mln	19,777	17,929	18,134	16,416	10,811	8,256
**Home Loan**							
No. of Loans disbursed	Absolute	67	62	108	64	44	21
Outstanding Volume	Rs. Mln	4,733	4,110	4,116	2,909	1,887	1,710
**Personal Loan**							
No. of Loans disbursed	Absolute	1,764	2,435	2,766	1,313	316	231
Outstanding Volume	Rs. Mln	1,912	2,262	2,707	2,630	531	518
**Bancassurance**							
No. of customers	Absolute	232,035	196,633	152,145	119,474	95,434	72,553
No. of new customers	"	35,402	44,021	32,671	24,040	22,881	17,142
No. of policies	"	35,791	44,208	33,110	26,590	23,223	17,189
Bancassurance Premium	Rs. Mln	9,654	8,927	7,060	6,133	4,953	4,193
**Trade**							
Imports - volume	Rs. Mln	577,281	563,914	483,932	416,489	371,233	359,860
Exports - volume	"	332,396	356,549	302,500	220,912	162,899	170,032
**Home Remittance**							
Volume of home remittance	USD Mln	3,206	3,051	3,064	2,281	2,220	2,096
Volume of home remittance	Rs. Mln	518,882	455,862	374,431	240,478	232,340	213,755
Home Remittance MCB Market Share	%	12.35%	13.74%	14.88%	11.64%	11.30%	10.90%
**Cash Management**							
Volume of cash collection	Rs. Mln	2,082,095	1,884,135	1,673,812	1,500,553	1,210,303	1,024,595

Six Years' - Performance Commentary 2015 - 2020

In this section, commentary on the six years' performance of the Bank is being provided, covering key highlights;

Statement of Financial Position

Total Assets:

The asset base of the Bank has registered a remarkable compounded annual growth rate (CAGR) of 11.10% over the last 6 years; growing to Rs. 1,757 billion as at December 31, 2020. Prime contributors to the said increase have been advances and investments, with investments growing annually by approximately 12.42% while gross advances growing by 9.75%. The earning asset mix of the Bank has been prudently managed to ensure maximization of returns to the stakeholders. In 2017, based on the strategic move, NIB Bank was merged with and into MCB Bank Limited resulting in a significant increase in assets of 25%. Furthermore in 2018, 90 branches of the Bank were transferred to a wholly owned subsidiary of the Bank i.e. MCB Islamic Bank Limited. In 2020, the Bank recorded a net growth of Rs.242 billion in total assets (16%) over 2019.

Advances:

Amidst the COVID-19 outbreak, evolving macro-economic factors made the operating environment more challenging; the discount rate registering a decrease of 625bps in the year 2020. A subdued domestic credit appetite coupled with growing risks surrounding asset quality and a limited number of credit extension opportunities within the Bank's defined risk appetite resulted in a decline in the gross advance base during the year 2020. Over the cumulative period of 6 years, however, the expansionary economic cycle witnessed in the middle part of the time analysis together with realization of MCB's merger with the NIB bank has reflected in positive growth numbers for MCB. Gross advances have grown at a CAGR of 9.75 % over the last 6 years.

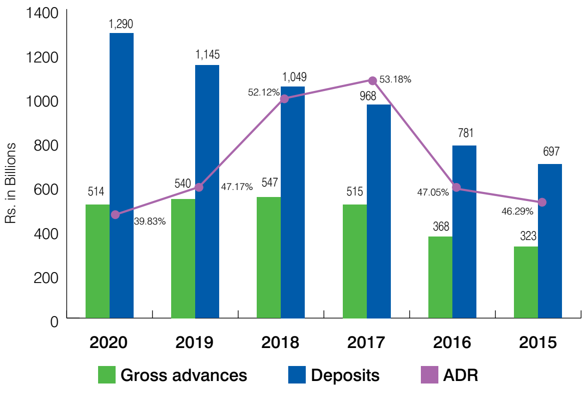

Non-performing Loans:

Strengthened risk management policies coupled with refined appetite has enabled the Bank to keep a check on the quality of its assets. Bank continued with the trend of posting significant recoveries and posted a recovery of Rs. 2.4 billion in 2020. The infection ratio of the Bank was 8.68% as at December 31, 2013 which improved to 5.90% as at December 31, 2016. However, transfer of NPL stock from NIB Bank i.e. Rs. 29.650 billion had increased the infection ratio to 9.47% as at December 31, 2017. In 2020, the infection ratio deteriorated slighted to 9.97% on account of subjective classifications undertaken by the Bank on a prudent basis and decrease in gross advances base. The coverage ratio of the Bank has improved from 90.37% as at December 31, 2015 to 98.87% as at December 31, 2020. NPLs classified in "loss" category constitute more than 98.94% of the NPLs base as at December 31, 2020. This specifies the adequacy of provision held in the books of the Bank.

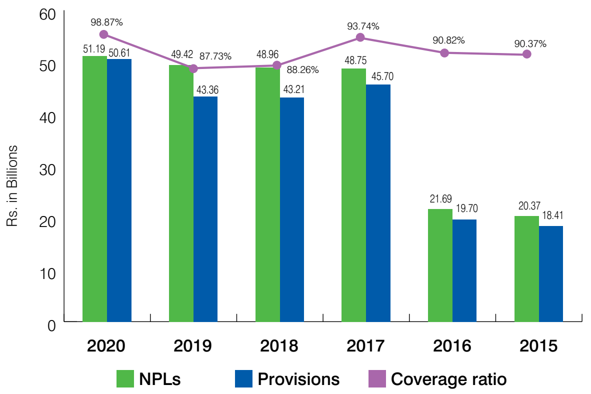

Investments:

During the past few years, the lack of credit opportunities resulted in banking sector liquidity being deployed in Government Papers as Government's borrowing appetite has continued to grow. With an average annual growth rate of 12.42% over the last six years, the investment base of the Bank has grown from Rs. 566 billion as at December 31, 2015 to Rs. 1,016 billion as at December 31, 2020. The duration of the investment portfolio has been proactively monitored amidst the evolving yield curve scenario to maximize returns and optimize liquidity management. The equity book of the Bank consists of investments in diverse companies with strong fundamentals with a view to earn stable dividends

Deposits:

The deposit base of the Bank has nearly doubled over the last six years, surpassing the landmark of PKR 1 trillion in 2018, with the absolute number increasing from Rs. 697 billion as at December 31, 2015 to Rs. 1,290 billion as at December 31, 2020. CAGR of 13.10% has been maintained over the past 6 years. CASA base has registered a remarkable increase in the last 6 years, increasing from Rs. 651 billion as at December 31, 2015 to Rs. 1,199 billion as at December 31, 2020. This has been strategically achieved through service excellence, strategically placed touch points for the customers and transactional convenience provided through a vast range of diversified products.

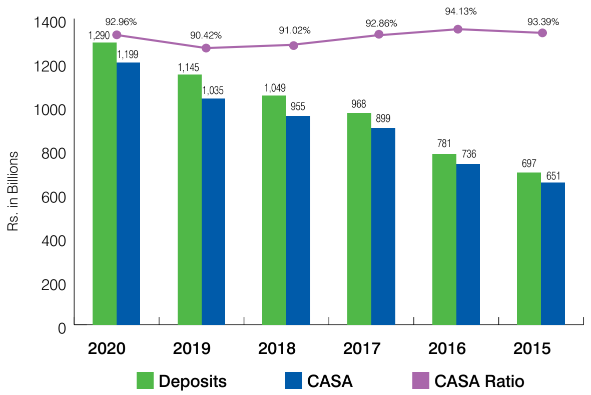

Equity and Dividends

- The paid-up capital of the Bank has grown from Rs. 11.13 billion as at December 2015 to Rs. 11.8 billion as at December 31, 2020, meeting the statutory capital requirements set by the State Bank of Pakistan (SBP).
- In 2008, most affluent strategic partnership occurred in Pakistan where the largest Bank of Malaysia, Maybank Berhad, acquired 20% holding in MCB Bank Limited (current holding of 18.78% as holding diluted due to issuance of shares under the merger scheme). In 2017, Fullerton Financial Holdings (International) of Singapore through Bugis Investments (Mauritius) Pte Ltd acquired 5.49% stake in MCB under merger scheme of NIB Bank with and into MCB Bank Limited.
- The equity base of the Bank is reflective of the outstanding financial results achieved through consistent performance posted over the years. The capital base of the Bank is rated as strong which is substantiated by the reported CAR of 20.98% as at December 31, 2020 against the statutory requirement of 11.50%. The Shareholders' equity has grown significantly from Rs. 113 billion as at December 31, 2015 to Rs. 162 billion as at December 31, 2020.
- The Bank has the highest cash dividend per share in the industry with regular interim dividends and remains one of the prime stocks preferred in the Pakistani equity markets.

Profit and Loss account

Net Interest Margin

- The composition of markup income has seen a shift in the last six years on the back of concentration shift in the earning asset base of the Bank. The contribution from markup income earned on advances in the total income mix had increased from 32.50% in 2015 to 41.46% in 2019 while the contribution from markup income on investments declined from 67.18% to 54.58% over the same period. However, diversion of excess liquidity towards the investment book due to lack of good credit opportunities has diluted the concentration of advances related markup income in the total income mix; the contribution falling down to 31.51% in 2020 while the contribution from markup income on investments has increased to 67.60 % in 2020.
- The investment to total asset ratio has increased slightly from 56.32% in 2015 to 57.80% in 2020; whereas, the advances to total asset ratio has decreased by 4% from the base year. Gross yield on average assets has also improved over the years and closed at 9.59% in 2020.
- The regulatory revisions covering minimum deposit rate introduced in the earlier years of this six years analysis has adversely affected the Bank's net interest margins. The said impact was strategically diluted by improving the CASA base during the period; taking it to over 92.96% as at December 31, 2020

Non-Markup Income

- During the last six years, fee & commission income and capital gains have been the major drivers behind non-fund income growth. Apart from the exception of current year wherein the realization of various systematic and idiosyncratic factors emanating from the COVID-19 outbreak impacted Banks income streams, the fee & commission income has witnessed a broad based growth over the years on the back of new products and services added to the menu coupled with remote banking and branchless initiatives taken by the Bank. The product development teams of the Bank have been tapping the unbanked segment of the population and offering them tailored products to meet their specific financial requirements. Different types of products have been offered in order to meet the needs of the time like credit card, mobile banking, visa debit card, MCB Lite, prepaid cards, IBFT etc.
- The fee, commission and brokerage block of the Bank have been constant contributors to its bottom line growth; major thrust to the income has been provided by commission earned on card business, bancassurance business, remittances and intercity / intra-city cash transfers.
- The equity book of the Bank consists of investments in diverse companies with strong fundamentals and has been a stable contributor to the non-markup income by providing outstanding dividend yields.

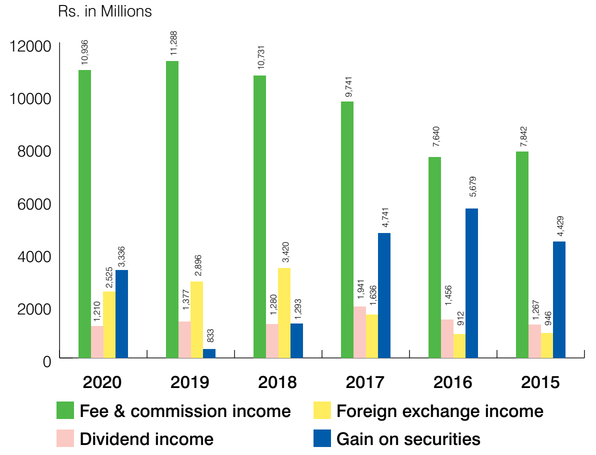

Provisions and write offs

- The Bank's risk management platform is being driven by an augmented framework of sound risk principles, optimum organizational structure, robust risk assessment models and effective monitoring systems in an IT enabled environment to effectively identify, evaluate and mitigate all risks undertaken in the achievement of its long-term strategic objectives. The captioned platform has formed the basis for a declining trend in provision charge booked against advances over the years; in fact, the Bank's has been able to post significant provision reversals up till year 2019 on the back of extensive recovery efforts undertaken.

  During 2020, however, the Bank has witnessed a trend reversal in recognition of provision against advances. The subjective classification of obligor accounts on a prudent basis coupled with an additional recognition of a general provision charge, amounting to Rs. 4 billion, in anticipation of the impact realization of COVID-19 pandemic post expiration of SBP's relaxations and waivers in 2021 have been the prime contributors to the captioned increase. The Bank has not taken any benefit of FSV in its provision calculation.

  The provision recognized against equity portfolio over the last 3 years amidst ensuing market volatility also witnessed a trend reversal in 2020 as the KSE index gained momentum on the back of improving market sentiment.

Operating expense:

- Considering expansion in Bank's branch outreach, continued investment in technological infrastructure, regular performance merit adjustments for the Human Capital, rising regulatory compliance costs and sustained inflationary patterns witnessed over the period of six years, the posted growth in the administrative block of the Bank has been nominal. Automation led saving initiatives, centralization of operations and imposition of annual capping's have been the key pillars of the cost management drive undertaken by the Bank; additionally laying the basis for the Bank to boast one of the lowest cost to income ratios in the industry.

Profit before tax and Profit after tax:

- MCB Bank has been able to post outstanding profitability numbers over the period of last six years as substantiated by one of the highest industry EPS and a remarkable return on assets ratio.
- The aggregate profit after tax for the last six years has crossed Rs. 144 billion.
- Profitability ratios have been one of the best in the banking industry which are reflective of the effective management of the affairs and adoption of prudent strategies.

Other statistics

- The Bank has added almost 2 million bank accounts to its base during the past 6 years with the current statistics highlighting the bank accounts to be over 8 million.
- With active participation in trade, MCB Bank has been able to improve trade volume in last 6 years which has increased to Rs. 577 billion for imports and Rs. 332 billion for exports.
- The Bank has been able to increase its market share in the remittance business growing from Rs. 214 billion as at December 31, 2015 to the volume of Rs. 519 billion as at December 31, 2020.
- In 2008, the Bank launched Bancassurance which was a unique cross functional service to the customers of the Bank. With only 131 policies and 129 customers in 2008, MCB Bank issues 35,791 new policies with premium amount of more than Rs. 2.59 billion in FY 2020.
- MCB Bank launched mobile banking in 2009 with approximately 53K customers reported by the end of the 2009. As of 2020, the mobile customers have grown to 1.39 million and above with transaction volume of over Rs. 78 billion.
- In 2015, MCB Annual Report had been honored to receive 1st place by South Asian Federation of Accountant (SAFA) for best presented Annual Accounts 2014. MCB was the 1st Pakistani bank to receive this accolade. In the past years, we have been awarded merit certificate and runner up awards for the same.
- In 2016, the Bank was awarded Best Bank in the “Corporate Finance and Investment Banking” by EuroMoney Awards. We have also been awarded Best Bank in the FinanceAsia Country Awards during the recent past years.
- China-Pakistan Economic Corridor (‘CPEC’) is beginning to show greater momentum with several energy and infrastructure projects in the pipeline, which will fuel growth in the medium term. Bank is continuously focusing on CPEC related infrastructure projects and bank has been able to achieve various awards on outstanding performance under CPEC arrangements. In this regard bank received award for Best Regional Bank in South Asia for BRI (Belt & Road Initiative) - Asiamoney New Silk Road Finance Awards 2017.
- In 2019, the Bank was declared as the “Most Outstanding Company in Pakistan” in the financial sector category by Asiamoney.
- During the year 2020, MCB's Annual Report was adjudged winner of the “Best Corporate Report” award in the financial sector category for the 8th consecutive year. MCB Bank has been awarded as winner by ICAP and ICMAP for the captioned award 10 times in the past 11 years.

Six Years' - Graphical Summary of Ratios 2015 - 2020

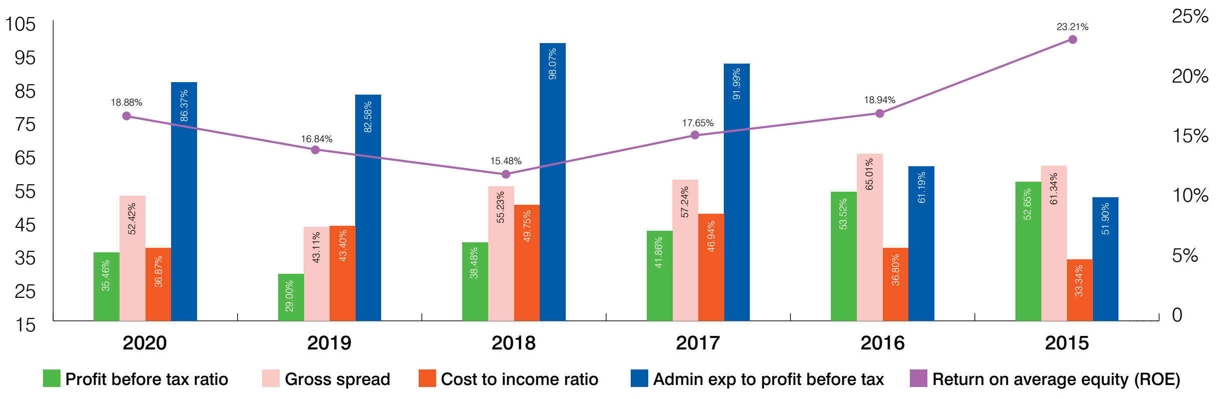

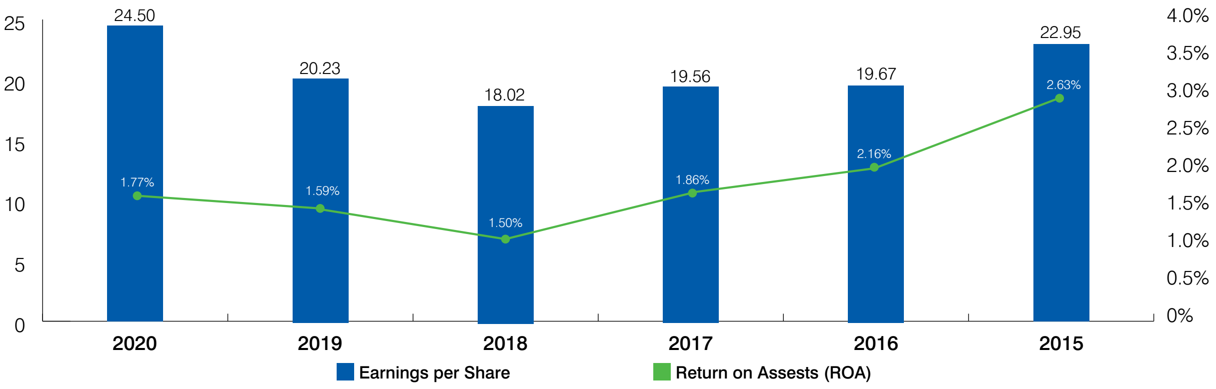

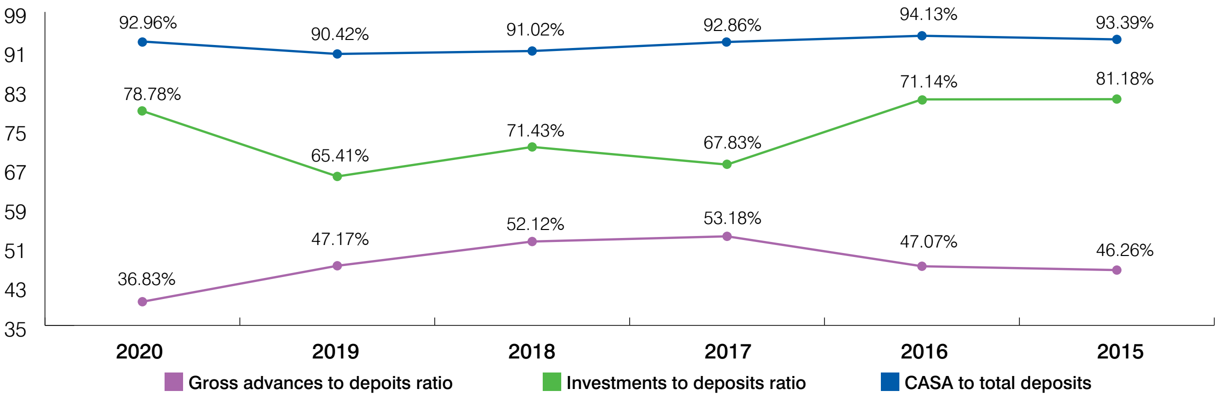

Six Years' - Concentration of Advances, NPLs and Off Balance Sheet Items 2015 - 2020

Gross Advances

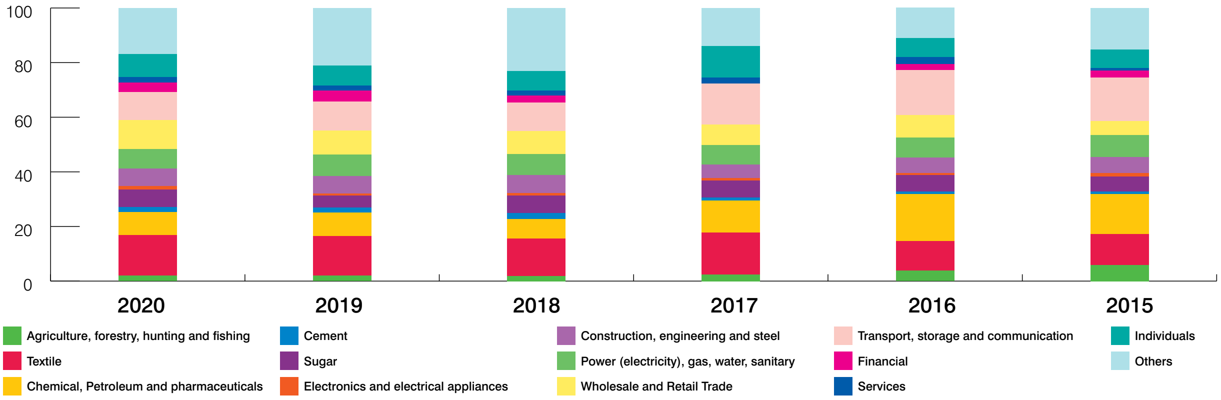

Classified Advances

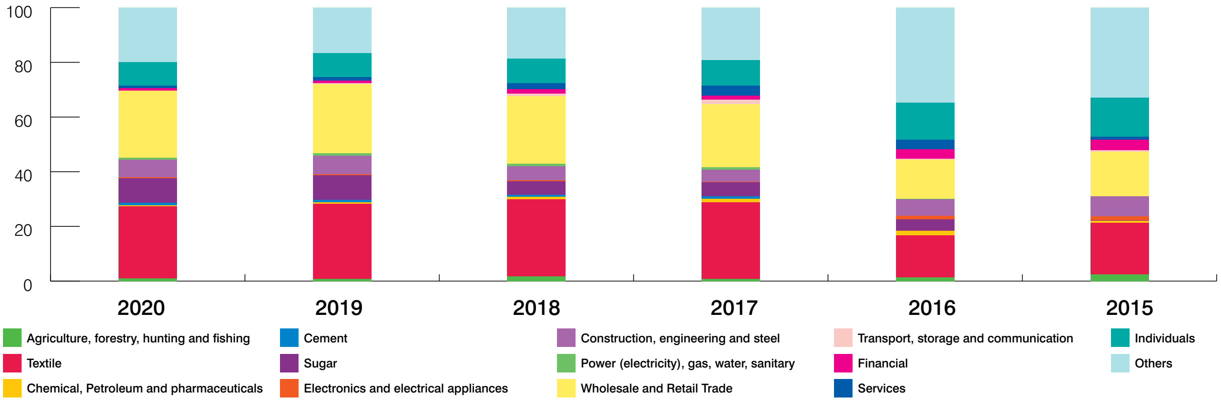

Off Balance Sheet Item

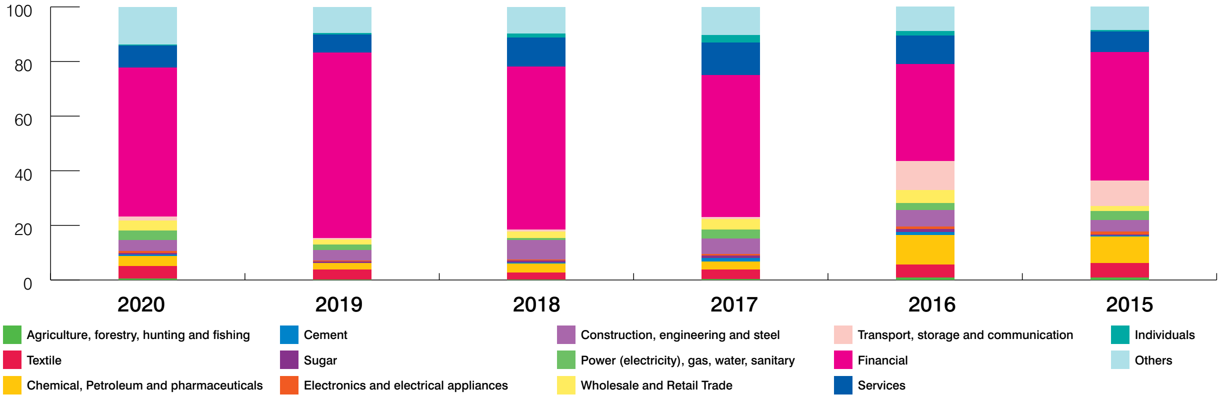

Six Years' - Maturities of Assets & Liabilities 2015 - 2020

Maturities of Assets

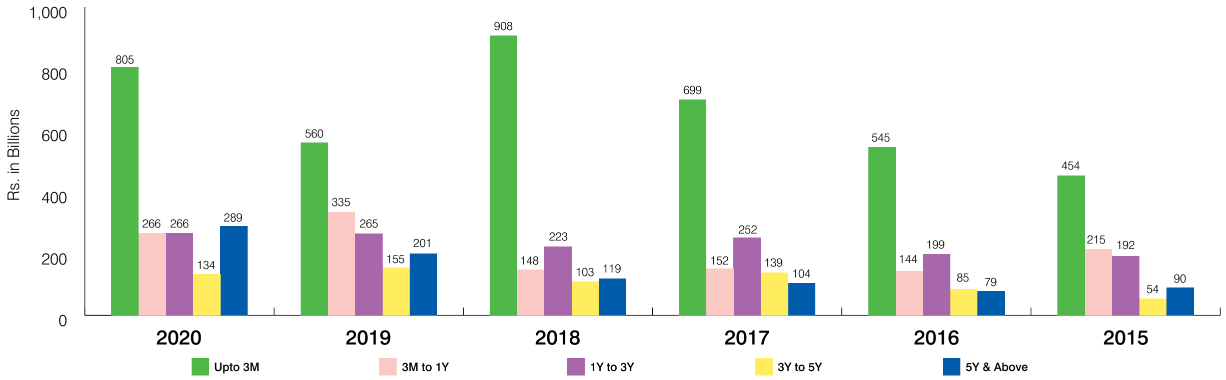

Maturities of Liabilites

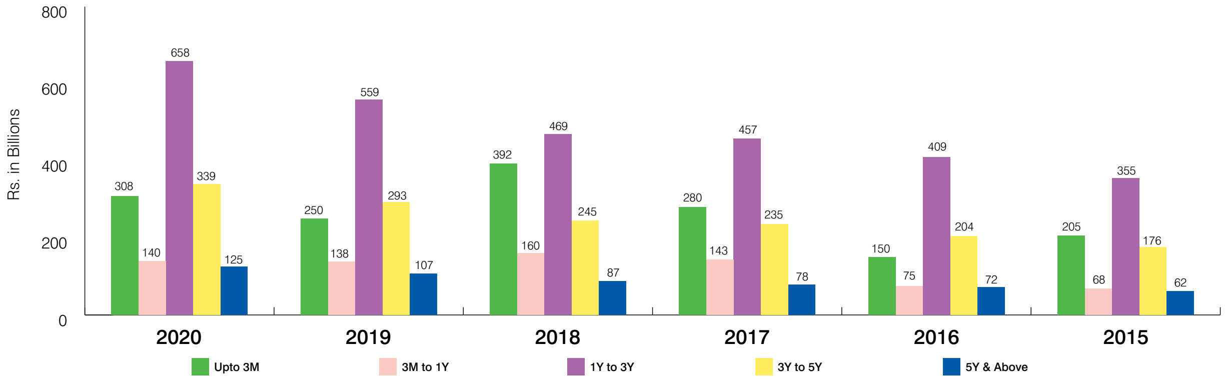

* Based on expected maturities

DuPont Analysis

			2020	2019	2018	2017	2016	2015
Net Operating Margin	PAT / Total Income	A	32.45%	31.43%	33.79%	36.95%	36.49%	38.78%
Asset Utilization	Total Income / Average Assets	B	5.47%	5.06%	4.45%	5.03%	5.78%	6.80%
Return on Assets		C = A x B	1.77%	1.59%	1.50%	1.86%	2.11%	2.63%
Equity Multiplier	Average Assets / Average Equity	D	10.64	10.58	10.29	9.49	8.99	8.81
Return on Equity		C X D	18.88%	16.84%	15.48%	17.65%	18.94%	23.21%

Following are the main DuPont analysis highlights:

1) Net operating margin has dropped during 2015-2019, with a increase in 2020.

2) Asset utilization has dropped during 2015-2018, and improved in 2019 & 2020.

3) Equity Multiplier showing increasing trend since 2015.

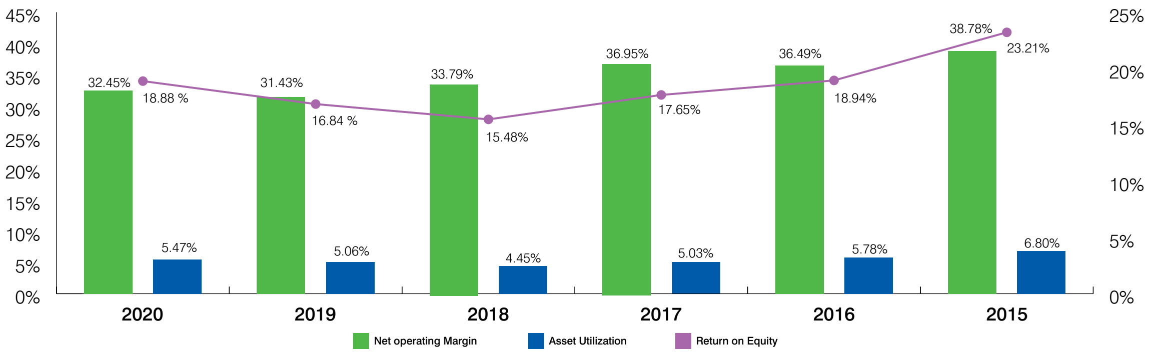

MCB
Bank for Life

Summary of Cash Flows

Rupees in million

	2020	2019	2018	2017	2016	2015
Cash flows from operating activities	285,808	48,192	143,221	121,010	21,593	82,440
Cash flows from / (used in) investing activities	(270,459)	6,681	(118,767)	(72,671)	11,043	(50,942)
*Cash flows used in financing activities	(13,433)	(20,603)	(18,794)	(22,777)	(17,912)	(17,243)
Cash & cash equivalents at beginning of the year	143,898	109,628	103,968	78,406	63,682	49,427
Cash & cash equivalents at end of the year	145,814	143,898	109,628	103,968	78,406	63,682

Commentary - Cash Flow Statement

Operating cash flows depict cash inflows generated from core activities of the Bank i.e. Deposit generation. The operating cash flows increased from Rs. 48.2 billion in 2019 to Rs. 285.8 billion in 2020 as greater contribution from deposits and borrowings from financial institutions added Rs. 144.7 billion and Rs. 75.4 billion to the inflows respectively.

Despite a restrained operating environment in 2020 amidst the COVID-19 outbreak, the Bank's strategic focus on improving deposit mobilization and optimizing its cost mix culminated into a significant growth of 12.6% in its deposits base; CASA was reported at 93%. A moderating economic activity coupled with limited credit extension avenues within the Bank's defined risk appetite meant that excess liquidity was diverted towards Bank's investment book; the investing cash flows hence reporting a massive outflow of Rs. 270.5 billion during the year.

Cash outflow from financing activities primarily reflect payments on account of dividends to the shareholders. During the year 2020, financing cash flows declined to Rs. 13.4 billion against an average outflow of Rs. 19.3 billion posted in the preceding 5 years as the State Bank of Pakistan (SBP) directed banks to suspend dividend distribution for two quarters of 2020 as a precautionary measure to conserve capital and enhance the liquidity and stress absorption capacity of the banks amidst the ongoing COVID-19 outbreak.

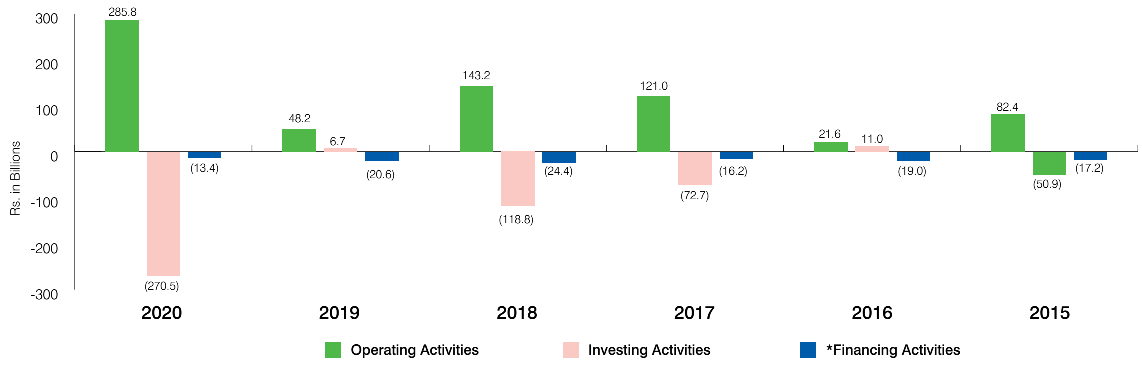

*Net Cash from financing activities include effects of exchange rate changes

Free Cash Flows

Rupees in million

	2020	2019	2018	2017	2016	2015
Profit before taxation	48,249	40,102	32,064	31,014	36,075	42,329
Adjustment for non-cash items	11,687	6,174	264	882	1,525	1,419
Operating assets/ liabilities changes	225,872	1,916	110,893	89,114	(16,007)	38,692
Net cash generated from operating activities	285,808	48,192	143,221	121,010	21,593	82,440
Capital expenditure	(3,090)	(5,022)	(4,483)	(4,745)	(3,485)	(942)
Free cash flows	288,898	43,170	138,738	116,265	18,108	81,498

Cash Flow Statement

Direct Method

	2020	2019
	(Rupees in Million)	
**Cash flows from operating activities**		
Mark-up / return / interest and commission receipts	159,662	140,715
Mark-up / return / interest payments	(84,595)	(67,306)
Payments to employees, suppliers and others	(29,162)	(29,592)
	**45,905**	**43,817**
**Decrease / (increase) in operating assets**		
Lendings to financial institutions	(16,049)	34,016
Net investments in 'held for trading' securities	8,180	(129)
Advances - net	26,262	7,225
Other assets	2,977	(3,554)
	**21,370**	**37,558**
**(Decrease) / increase in operating liabilities**		
Bills payable	12,159	(3,878)
Borrowings	75,448	(125,990)
Deposits and other accounts	144,739	95,726
Other liabilities	2,265	3,266
	**234,611**	**(30,876)**
	**301,886**	**50,499**
Income tax paid	(16,078)	(2,307)
**Net cash flows from operating activities**	**285,808**	**48,192**
**Cash flows from investing activities**		
Net investments in 'available for sale' securities	(286,438)	8,628
Net investments in 'held to maturity' securities	17,363	310
Investment in a subsidiary company	–	(350)
Dividends received	1,180	1,395
Investments in operating fixed assets	(2,791)	(4,396)
Investments in intangible assets	(299)	(626)
Sale proceeds of operating fixed assets and intangible assets disposed off	187	158
Proceeds from sale of non-banking assets acquired in satisfaction of claims	39	540
Investments in non-banking assets acquired in satisfaction of claims	–	(64)
Net cash inflow from amalgamation of a subsidiary	–	41
Proceeds from divestment in a subsidiary	100	–
Exchange differences on translation of the net investment in foreign branches	201	1,046
**Net cash flows from / (used in) investing activities**	**(270,458)**	**6,682**
**Cash flows from financing activities**		
Payments of Subordinated debt	–	(3,891)
Dividend paid	(11,751)	(18,838)
Payment of lease liability against right-of-use-assets	(1,683)	(1,428)
**Net cash flows used in financing activities**	**(13,434)**	**(24,157)**
Effects of exchange rate changes on cash and cash equivalents	1,595	3,553
**Increase in cash and cash equivalents**	**3,511**	**34,270**
Cash and cash equivalents at beginning of the year	142,303	109,628
**Cash and cash equivalents at end of the year**	**145,814**	**143,898**

Cash flow statement in annual financial statement is required to be prepared in line with the format prescribed by State Bank of Pakistan under BPRD Circular No .2 dated 25, 2018, 'Revised Forms of Annual Financial statements.

Markup & Non Markup Income

	2020	2019	2018	2017	Rupees in Million<br>2016	2015
**Markup Income**						
Loans and advances	42,879	57,330	36,964	26,931	22,956	26,128
Investments	91,983	75,481	44,719	46,876	44,226	54,010
Lendings to Financial Institutions	998	4,982	1,390	174	122	176
Balance with banks	215	499	246	111	97	79
	136,076	138,292	83,319	74,091	67,400	80,393
**Markup Expense**						
Deposits	55,095	65,344	32,081	22,105	18,313	23,326
Borrowings	6,152	8,977	4,253	8,837	4,556	7,185
Subordinated loan	–	214	308	138	–	–
Unwinding cost of liability against right-of-use assets	2,387	1,132	–	–	–	–
Others	1,107	3,009	663	349	717	566
	64,741	78,676	37,305	31,429	23,586	31,077
**Net Markup Income**	71,334	59,616	46,014	42,662	43,814	49,316
**Non Markup Income**						
Fee & commission Income	10,936	11,288	10,731	9,741	7,640	7,842
Dividend Income	1,210	1,377	1,280	1,941	1,456	1,267
Foreign exchange income	2,525	2,895	3,420	1,636	912	946
Gain on securities	3,336	833	1,293	4,741	5,679	4,429
Other Income	128	286	474	58	488	2,083
	18,136	16,679	17,198	18,118	16,175	16,566

Markup Income from Advances and Investments (2015-2020)

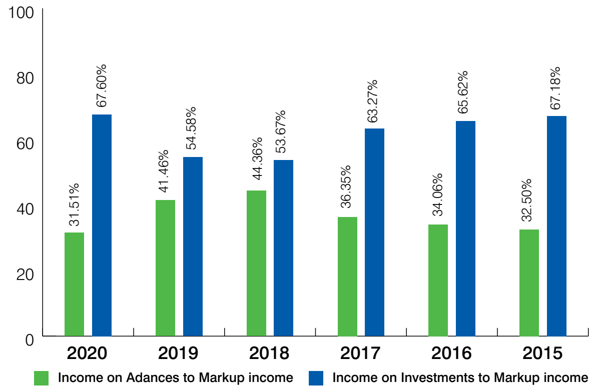

Income Composition (2015-2020)

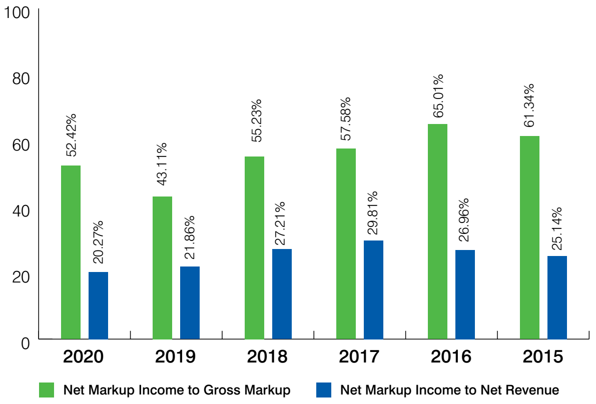

Operating Expenses

	2020	2019	2018	2017	Rupees in Million<br>2016	2015
**Total compensation expense**	15,806	14,585	14,053	12,301	9,111	8,962
**Property expense**						
Rent & taxes	161	227	1,959	1,526	1,047	1,044
Insurance	23	21	26	27	24	25
Utilities cost	1,218	1,203	1,095	911	739	745
Fuel Expense	335	534	514	459	368	545
Security (including guards)	1,240	1,382	1,603	1,392	1,178	1,034
Repair & maintenance (including janitorial charges)	653	812	945	790	660	516
Depreciation on right-of-use assets	1,217	1,162	-	-	-	-
Depreciation	642	504	458	383	385	390
	5,488	5,845	6,599	5,487	4,400	4,300
**Information technology expenses**						
Software maintenance	1,148	1,188	1,151	863	763	732
Hardware maintenance	212	299	364	349	239	239
Depreciation	548	616	721	690	644	706
Amortization	318	301	256	244	366	364
Network charges	598	601	616	621	554	631
Insurance	4	3	4	5	6	5
	2,829	3,009	3,112	2,772	2,572	2,677
**Other operating expenses**						
Directors' fees and allowances	50	57	41	39	35	35
Legal & professional charges	268	350	302	328	265	212
Outsourced services costs	692	690	1,119	1,167	818	830
Travelling & conveyance	261	321	343	293	396	236
NIFT clearing charges	166	152	146	136	126	138
Depreciation	816	797	802	656	544	475
Depreciation on non-banking assets	30	45	49	30	29	-
Training & development	37	57	51	60	51	45
Postage & courier charges	234	303	271	323	247	267
Communication	326	373	317	384	306	308
Stationery & printing	563	639	704	646	543	609
Marketing, advertisement & publicity	695	625	518	531	483	362
Donations	113	0	1	12	13	-
Auditors Remuneration	30	30	34	41	24	39
Cash transportation charges	709	799	744	631	551	545
Repair & maintenance	402	416	460	513	403	331
Subscription	21	20	24	18	27	16
Entertainment	184	232	233	235	186	174
Credit Card Related Expenses	829	1,182	738	562	415	348
CNIC verification charges	129	207	138	107	76	62
Insurance	1,483	1,441	821	254	236	294
Others	486	493	473	431	215	286
	8,523	9,232	8,327	7,397	5,991	5,611
**Operating excluding compensation**	16,840	18,086	18,038	15,657	12,963	12,589
**Total operating expenses**	32,646	32,671	32,091	27,958	22,074	21,551

Cost to income ratio

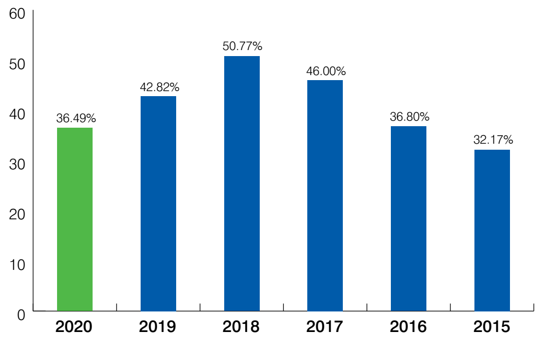

Economic Value Added Statement

	2020	2019
	(Rupees in Million)	
**Invested Capital**		
Average shareholders' equity	153,800	142,375
Add: Cumulative provisions against assets	63,844	56,652
	217,644	199,027
Return on Invested Capital		
Profit after taxation	29,037	23,977
Add: Provisions and write offs - net	7,313	2,484
Total return on invested capital	36,350	26,460
Economic cost	11.37%	12.89%
Opportunity cost of invested capital	24,746	25,655
Economic Value Added	11,604	806

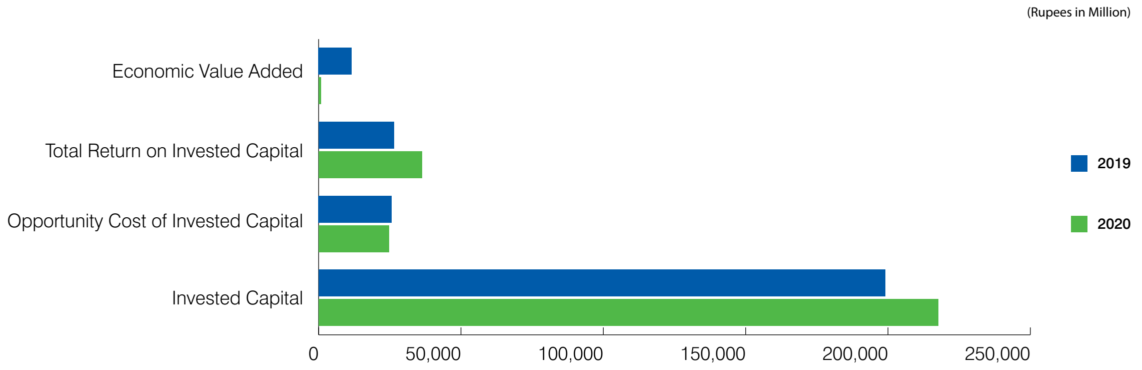

Comments:

Increase in EVA as compared to last year is due to increase in profitability and decrease in economic cost due to revision in policy rates during the year.

Capital expenditures

Capital expenditure during the year:

The total capital expenditure during 2020 was Rs. 3.09 billion for business expansion, renovation and improvement of IT infrastructure.

Capital expenditures planned for next year:

The Bank has budgeted capital expenditure of Rs. 6.9 billion for the next year. This would primarily be invested in increasing our operational outreach, continuous improvement in our Information Technology platforms, safeguarding our existing infrastructure / relationships from growing threats on cyber security front along with normal replacements to ensure smooth operations.

Six Years' Vertical Analysis
Statement of Financial Position/Profit & Loss

	2020		2019		2018		2017		2016		2015	
	Rs. Mln	%	Rs. Mln	%	Rs. Mln	%	Rs. Mln	%	Rs. Mln	%	Rs. Mln	%
**Statement of Financial Position**												
**Assets**												
Cash and balances with treasury banks	122,181	7%	132,705	9%	103,175	7%	106,072	8%	74,222	7%	60,568	6%
Balances with other banks	24,030	1%	12,542	1%	11,879	1%	4,579	0%	4,344	0%	3,611	0%
Lendings to financial institutions	17,139	1%	1,090	0%	35,106	2%	4,398	0%	2,810	0%	3,080	0%
Investments	1,015,869	58%	748,765	49%	749,369	50%	656,964	49%	555,929	52%	565,696	56%
Advances	462,942	26%	496,679	33%	503,581	34%	469,356	35%	348,117	32%	304,122	30%
Fixed assets	58,028	3%	58,271	4%	40,812	3%	39,170	3%	32,409	3%	29,227	3%
Intangible assets	938	0%	958	0%	630	0%	404	0%	343	0%	723	0%
Other assets	56,334	3%	64,143	4%	53,578	4%	62,295	5%	54,191	5%	37,384	4%
	1,757,462	100%	1,515,152	100%	1,498,130	100%	1,343,238	100%	1,072,365	100%	1,004,410	100%
**Liabilities**												
Bills payable	23,981	1%	11,822	1%	15,699	1%	22,681	2%	12,844	1%	11,889	1%
Borrowings	164,002	9%	89,506	6%	216,019	14%	133,070	10%	74,515	7%	118,040	12%
Deposits	1,289,502	73%	1,144,763	76%	1,049,038	70%	968,483	72%	781,430	73%	696,805	69%
Sub-ordinated loan	-	-	-	-	3,891	0%	3,893	0%	-	-	-	-
Deferred tax liabilities	6,975	0%	5,851	0%	1,532	0%	4,625	0%	11,260	1%	11,377	1%
Other liabilities	82,901	5%	94,296	6%	62,673	4%	56,921	4%	50,690	5%	28,498	3%
	1,567,361	89%	1,346,237	89%	1,348,852	90%	1,189,672	89%	930,739	87%	866,608	86%
**Net Assets**	190,102	11%	168,915	11%	149,278	10%	153,566	11%	141,627	13%	137,802	14%
**Represented by**												
Share capital	11,851	1%	11,851	1%	11,851	1%	11,851	1%	11,130	1%	11,130	1%
Reserves	80,696	5%	77,591	5%	74,148	5%	70,866	5%	53,347	5%	51,309	5%
Surplus on revaluation of assets - net of tax	27,720	2%	23,695	2%	9,747	1%	17,073	1%	23,680	2%	24,616	2%
Unappropriated profit	69,835	4%	55,777	4%	53,532	4%	53,776	4%	53,469	5%	50,747	5%
	190,102	11%	168,915	11%	149,278	10%	153,566	11%	141,627	13%	137,802	14%
**Profit & Loss Account**												
Mark-up earned	136,076	88%	138,292	89%	83,319	83%	74,091	80%	67,400	81%	80,393	83%
Mark-up expensed	(64,741)	-42%	(78,676)	-51%	(37,305)	-37%	(31,429)	-34%	(23,586)	-28%	(31,077)	-32%
Net mark-up income	71,334	46%	59,616	38%	46,014	46%	42,662	46%	43,814	52%	49,316	51%
Non-mark-up income	18,136	12%	16,679	11%	17,198	17%	18,118	20%	16,175	19%	16,566	17%
Total income	89,470	58%	76,295	49%	63,212	63%	60,780	66%	59,989	72%	65,882	68%
Non-mark-up expenses	(33,908)	-22%	(33,709)	-22%	(32,902)	-33%	(28,721)	-31%	(22,989)	-28%	(22,895)	-24%
Profit before provisions	55,562	36%	42,586	27%	30,310	30%	32,059	35%	36,999	44%	42,987	44%
Provisions & write off	(7,313)	-5%	(2,484)	-2%	1,753	2%	(1,045)	-1%	(925)	-1%	(659)	-1%
Profit before taxation	48,249	31%	40,102	26%	32,064	32%	31,014	34%	36,075	43%	42,329	44%
Taxation	(19,212)	-12%	(16,125)	-10%	(10,704)	-11%	(8,555)	-9%	(14,184)	-17%	(16,782)	-17%
Profit after taxation	29,037	19%	23,977	15%	21,360	21%	22,459	24%	21,891	26%	25,546	26%

Six Years' Horizontal Analysis

Statement of Financial Position/ Profit & Loss

	2020<br>Rs. Mln	20 Vs 19<br>%	2019<br>Rs. Mln	19 Vs 18<br>%	2018<br>Rs. Mln	18 Vs 17<br>%	2017<br>Rs. Mln	17 Vs 16<br>%	2016<br>Rs. Mln	16 Vs 15<br>%	2015<br>Rs. Mln	15 Vs 14<br>%
**Statement of Financial Position**												
**Assets**												
Cash and balances with treasury banks	122,181	-8%	132,705	29%	103,175	-3%	106,072	43%	74,222	23%	60,568	30%
Balances with other banks	24,030	92%	12,542	6%	11,879	159%	4,579	5%	4,344	20%	3,611	20%
Lendings to financial institutions	17,139	1472%	1,090	-97%	35,106	698%	4,398	57%	2,810	-9%	3,080	117%
Investments	1,015,869	36%	748,765	0%	749,369	14%	656,964	18%	555,929	-2%	565,696	11%
Advances	462,942	-7%	496,679	-1%	503,581	7%	469,356	35%	348,117	14%	304,122	0%
Operating fixed assets	58,028	0%	58,271	43%	40,812	4%	39,170	21%	32,409	11%	29,227	-4%
Intangible assets	938	-2%	958	52%	630	56%	404	18%	343	-53%	723	4%
Other assets	56,334	-12%	64,143	20%	53,578	-14%	62,295	15%	54,191	45%	37,384	0%
	1,757,462	15.99%	1,515,152	1.14%	1,498,130	11.53%	1,343,238	25.26%	1,072,365	7%	1,004,410	7%
**Liabilities**												
Bills payable	23,981	103%	11,822	-25%	15,699	-31%	22,681	77%	12,844	8%	11,889	-29%
Borrowings	164,002	83%	89,506	-59%	216,019	62%	133,070	79%	74,515	-37%	118,040	98%
Deposits	1,289,502	13%	1,144,763	9%	1,049,038	8%	968,483	24%	781,430	12%	696,805	1%
Sub-ordinated loan	-	–	-	-100%	3,891	0%	3,893	100%	-	-	-	-
Deferred tax liabilities	6,975	19%	5,851	282%	1,532	-67%	4,625	-59%	11,260	-1%	11,377	9%
Other liabilities	82,901	-12%	94,296	50%	62,673	10%	56,921	12%	50,690	78%	28,498	-4%
	1,567,361	16%	1,346,237	0%	1,348,852	13%	1,189,672	28%	930,739	7%	866,608	8%
**Net Assets**	190,102	13%	168,915	13%	149,278	-3%	153,566	8%	141,627	3%	137,802	6%
**Represented by**												
Share capital	11,851	0%	11,851	0%	11,851	0%	11,851	6%	11,130	0%	11,130	0%
Reserves	80,696	4%	77,591	5%	74,148	5%	70,866	33%	53,347	4%	51,309	5%
Surplus on revaluation of assets - net of tax	27,720	17%	23,695	143%	9,747	-43%	17,073	-28%	23,680	-4%	24,616	6%
Unappropriated profit	69,835	25%	55,777	4%	53,532	0%	53,776	1%	53,469	5%	50,747	8%
	190,102	13%	168,915	13%	149,278	-3%	153,566	8%	141,627	3%	137,802	6%
**Profit & Loss Account**												
Mark-up earned	136,076	-2%	138,292	66%	83,319	12%	74,091	10%	67,400	-16%	80,393	4%
Mark-up expensed	(64,741)	-18%	(78,676)	111%	(37,305)	19%	(31,429)	33%	(23,586)	-24%	(31,077)	-8%
Net mark-up income	71,334	20%	59,616	30%	46,014	8%	42,662	-3%	43,814	-11%	49,316	13%
Non-mark-up income	18,136	9%	16,679	-3%	17,198	-5%	18,118	12%	16,175	-2%	16,566	28%
Total income	89,470	17%	76,295	21%	63,212	4%	60,780	1%	59,989	-9%	65,882	17%
Non-mark-up expenses	(33,908)	1%	(33,709)	2%	(32,902)	15%	(28,721)	25%	(22,989)	0%	(22,895)	6%
Profit before provisions	55,562	30%	42,586	40%	30,310	-5%	32,059	-13%	36,999	-14%	42,987	23%
Provisions & write off	(7,313)	194%	(2,484)	-242%	1,753	-268%	(1,045)	13%	(925)	40%	(659)	-135%
Profit before taxation	48,249	20%	40,102	25%	32,064	3%	31,014	-14%	36,075	-15%	42,329	15%
Taxation	(19,212)	19%	(16,125)	51%	(10,704)	25%	(8,555)	-40%	(14,184)	-15%	(16,782)	35%
**Profit after taxation**	29,037	21%	23,977	12%	21,360	-5%	22,459	3%	21,891	-14%	25,546	5%

Commentary on Six Years' Horizontal & Vertical Analysis

Horizontal Analyses

The asset base of the Bank has increased considerably over the past 6 years and has crossed multiple milestones; including the landmark achievement of crossing Rs. 1.5 trillion in 2019. On an annualized basis, the asset base of the Bank has recorded an increase of 11.84% over the base year while the investments and gross advances have posted an increase of 12.42% and 9.75% respectively. Highest increase in the asset base has been observed in 2017 as the transfer of portfolio on account of merger of NIB Bank with and into MCB Bank Limited added to the asset base; the assets hence growing by 25% on a year on year (YoY) basis in 2017.

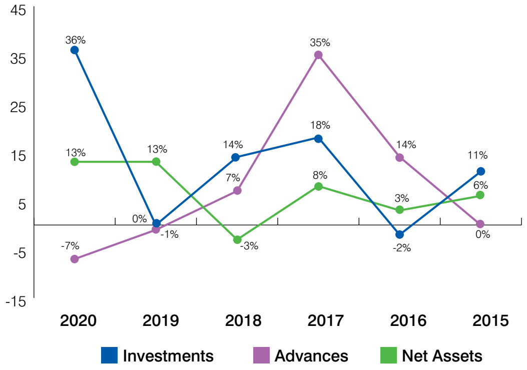

The deposit base of the Bank has increased considerably over the years; growing from Rs. 697 billion to Rs. 1,290 billion in 2020 and translating into a CAGR of 13.10% over the past 6 years. Highest increase has been reported in 2017, primarily on account of splendid volumetric growth coupled with deposits transferred under merger of NIB Bank into MCB Bank Limited. During the year 2018, MCB transferred business of its 90 branches to MCB wholly owned subsidiary i.e. MCB Islamic Bank Limited and deposits amounting to Rs. 21.9 billion were transferred under the de-merger scheme. Despite this transfer, the deposits grew by 8% in 2018 and have continued the upward trajectory in subsequent years; posting a healthy growth of 9% and 13% in 2019 and 2020 respectively.

Equity base of the bank has also posted a healthy increase due to higher profitability in the past 6 years; translating into a CAGR of 7.49% over the base year.

On the Profit and Loss side, gross markup earned has posted an average increase of 11.10% over a span of six years. This is due to the increase in mark up earned on investments and loan and advances in the last 6 years. The highest gross markup income over the span has been reported in 2019 as the double digit policy rate coupled with healthy volumetric gains in the earning assets base contributed to the significant rise. The increase in markup expense on deposits is on account of regulatory revisions in MDR enacted by the Central Bank and volumetric increase achieved in the deposit base. The cost of deposit for the Bank has been strategically managed by maintaining the CASA base of the Bank. However, the total markup expense has increased by 15.81% over the six year period under coverage.

The non-markup income block of the Bank has remained a steady contributor to its bottom line growth. On the other hand, growth in non-markup expenses has been kept in check through realization of various initiatives implemented as part Bank's cost management drive; hence depicting a meager growth of 6.76% over the period under review despite continuous investment in Banks digital and physical infrastructure along with sustained inflationary pressures.

One of the key strengths of the Bank has been the recovery of its classified portfolio which is clearly reflected in the reduced / reverse credit charge booked over the last few years. However, in 2020, anticipating that customers affected by the pandemic might require provisioning once SBP's relaxations and waivers expire in 2021, the management has proactively booked a general provision charge of Rs. 4.0 billion. This coupled with the subjective provisioning of obligor accounts on a prudent basis has resulted in trend reversal for recognition of credit charge during the year; in turn further supplementing Bank's insulation and loss absorption capacity against rising risks to the asset quality.

MCB enjoys one of the highest spreads in the banking industry which are duly reflected in the profitability ratios of the Bank.

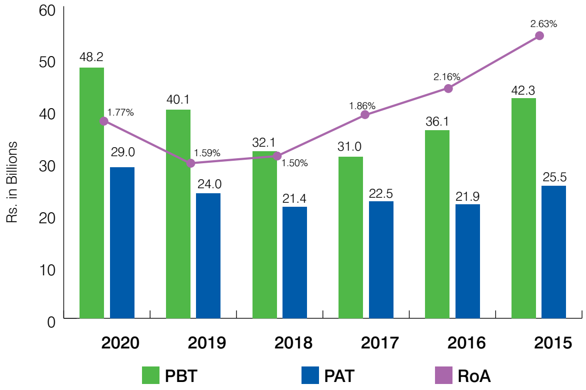

Vertical Analyses

Vertical analysis depicts higher concentration levels of investments and advances in the asset base of the Bank. The advances base of the Bank had posted growth till 2019 with the concentration levels of advances in the total assets mix improving from 30% in 2015 to 33% in 2019. However, the decline in advances witnessed during 2020 due to lack of credit extension opportunities within

the Bank's defined risk appetite and the consequent diversion of excess liquidity towards the investment book has diluted advances concentration in the total asset mix; hence falling to 26% in 2020.

Advances Concentration Level

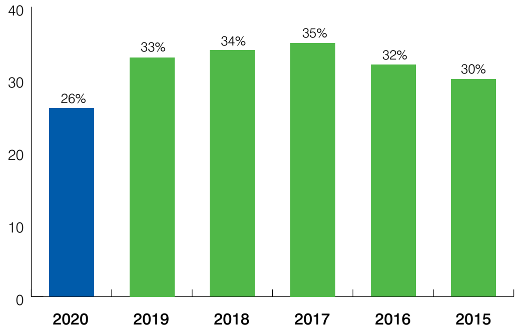

The IDRs of the banking industry registered a huge spike in the year 2015 on account of higher yielding longer term bonds being offered. However, based on the call that interest rate cycle has bottomed out, a gradual shift to shorter term securities with increased focus on credit was observed. Resultantly, the concentration levels of investments declined from 56% in 2015 to 49% in 2019. In 2020, deployment of excess liquidity in the investment portfolio has increased concentration levels of investments to 58%.

Investments Concentration Level

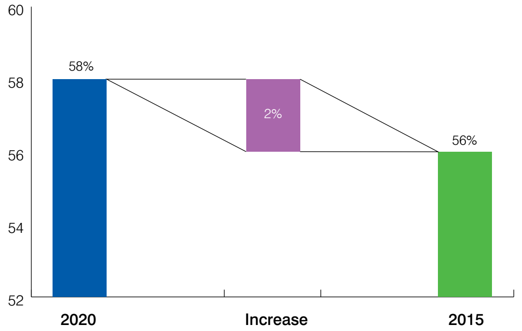

Corresponding to the technological, infrastructural and operational spend by the Bank, the deposit base has increased over the period of six years. Improved service quality levels and tailored products have earned the loyalty of our customers. This can be substantiated by the fact that the CASA base of the bank has remained over 90% over period under review; in turn reflecting management's strategic focus of improving Bank's deposit base and proactively monitoring its cost mix.

Markup income growth has been steady over the last 6 years. The contribution from markup income approximates 88% of the total revenue. Markup expense has increased over the last 6 years based on regulatory revisions enacted over the period and growth registered in the deposit base.

Concentration of non-markup income in the total income has increased from 17% in 2014 to 20% in 2017. However, the last 3 years have witnessed a slight dilution on account of rising gross markup income whose concentration in the total revenue increased form 80% in 2017 to 88% in 2020 on account of volumetric growth and rising interest rates. Non markup expense concentration level has declined from 24% in 2015 to 22% in 2020 despite of increase in business which is due to various cost control initiatives.

Segment Analysis

A segment is a distinguishable component of the Bank that is engaged in providing products or services (business segment) or in providing products or services within a particular economic environment (geographical segment), which is subject to risks and rewards that are different from those of other segments. The Bank's primary format of reporting is based on business segments.

Retail

This includes retail lending and deposits, banking services, cards and branchless banking.

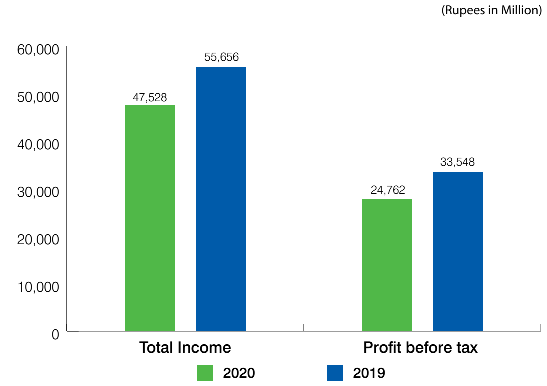

Corporate

This comprises of loans, deposits, project financing, trade financing, investment banking and other banking activities / with Bank's corporate and public sector customers including the Bank's overseas operation.

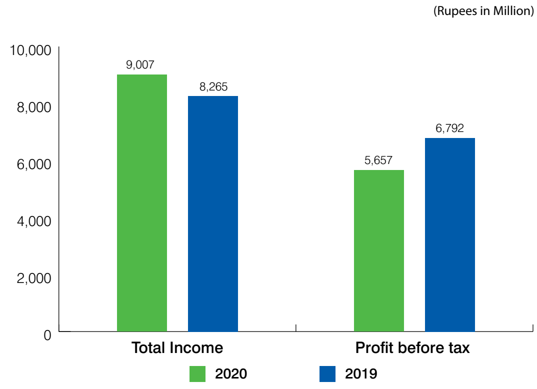

Consumer

This segment primarily constitutes consumer financing activities with individual customers of the Bank. Product suites offered to these customers include credit cards, auto loans, housing finance and personal loans.

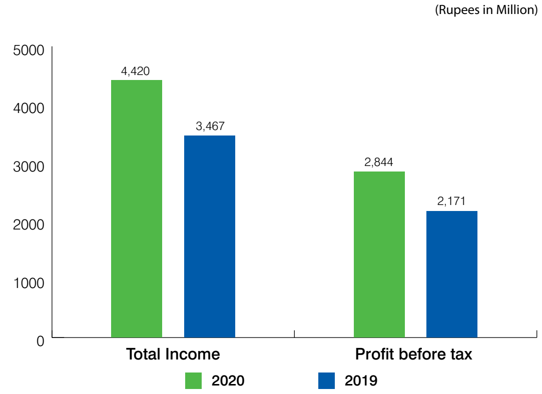

Treasury

This includes fixed income, equity, foreign exchange, credit, funding, own position securities, lendings and borrowings and derivatives for hedging and market making.

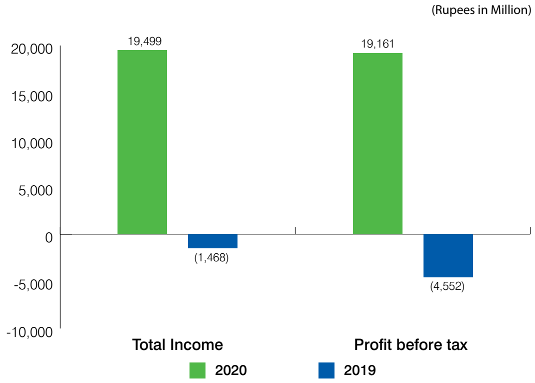

International

This comprises of loans, deposits, project financing, trade financing, investment banking and other banking activities / with Bank's corporate and public sector customers including the Bank's overseas operation.

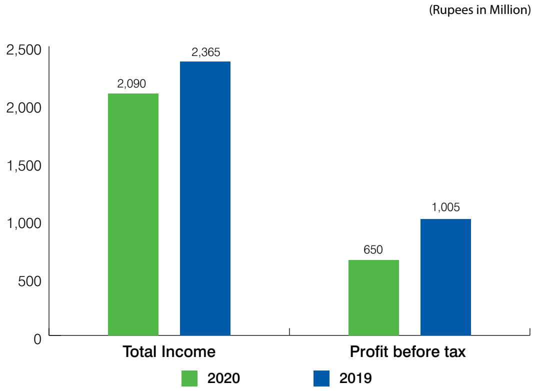

Geographical Segment

The Bank operates in following geographic regions:

Pakistan

(Rupees in Million)

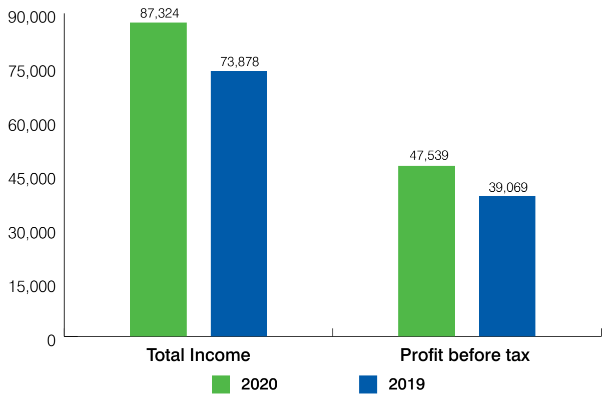

Middle East

(Rupees in Million)

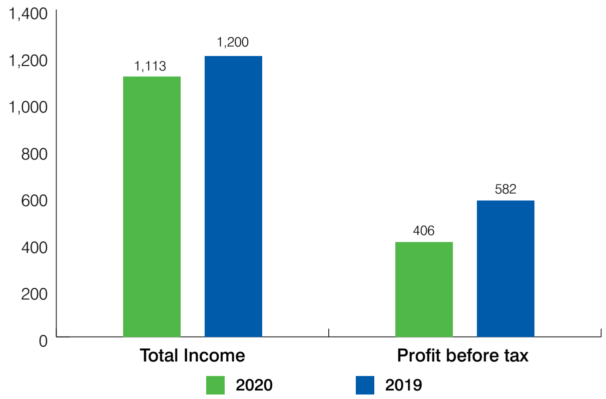

South Asia

(Rupees in Million)

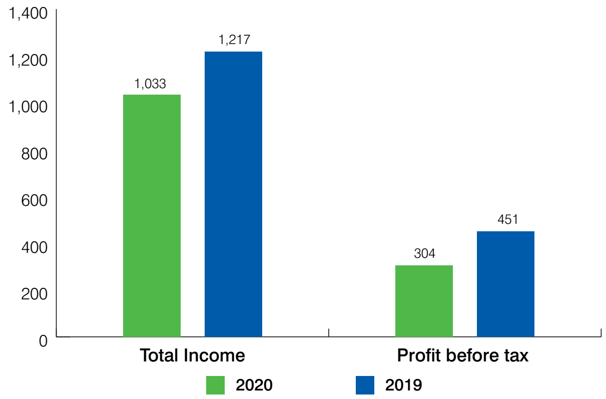

Statement of Charity and Donation

Statement of charity fund management by MCB Islamic Bank Limited (Wholly owned subsidiary of MCB Bank Limited)

	2020	2019
	(Rupees in '000)	
**Reconciliation of charity fund balance**		
Opening balance	57,782	25,402
Additions during the year		
- Received from customers against late payment	42,477	45,958
- Dividend purification amount	771	7,383
- Profit on charity saving account	2,085	1,539
	45,333	54,880
Charity paid during the year	(56,500)	(22,500)
Closing balance	46,615	57,782
**Charity was paid to the following institutions**:		
The Patients' Bahbood Society for AKUH	1,000	1,000
Al-Khidmat Foundation	2,000	–
Arthritis Care	2,000	1,000
Aziz Jehan Begum Trust for the Blind	3,000	1,000
Chiniot Anjuman Islamia	1,000	–
Chiniot Blood Bank and Dialysis Centre	1,000	–
Family Welfare Society	3,000	1,000
Fatmid Foundation	3,000	1,000
Indus Hospital	5,000	2,000
Infaq Memorial Trust	3,000	1,000
Layton Rehmatullah Benevolent Trust	4,000	2,000
Mind Organization	2,000	1,000
Pink Ribbon	2,000	5,000
Saleem Memorial Trust Hospital	6,000	–
Saylani Welfare	2,000	–
Shaukat Khanam Memorial Cancer Hospital	3,000	2,000
Sindh Institute of Urology & Transplantation (SIUT)	3,000	2,000
The Citizens Foundation	4,000	2,000
Jahandad Society for Community Development	1,500	–
The Lahore Hospital Welfare Society	2,000	500
Al - Mustafa Welfare Society	500	–
Frontier Foundation blood transfusion Centre	500	–
Zubaida Medical Center	1,000	–
Mofad e Amma Chiniot Sheikh Association	1,000	–
	56,500	22,500
**In addition to the above charity, detail of donation by the Bank is given below:**		
Murshid Hospital & Health Care Centre	–	100
Saleem Memorial Trust Hospital	95,000	–
Specialized Healthcare and Medical Education Department, Government of Punjab - (COVID 19 relief)	9,996	–
Jahandad Society For Community Development	5,000	–
District Administration Lahore - (COVID 19 relief)	2,600	–
	112,596	100

MCB
Bank for Life

Market Statistics of MCB Share

	Share Prices			Free Float		Market Capitalisation	
	MCB Scrip (Rs.)			Share ('000s)	%	Capital	Value
2020	High	Low	Closing			(Mln)	(Mln)
December 31, 2020	186.22	164.12	185.28	401,668	33.89%	11,851	219,568
September 30, 2020	183.29	160.33	173.82	402,073	33.93%	11,851	205,987
June 30, 2020	166.42	146.13	162.07	402,267	33.94%	11,851	192,063
March 31, 2020	224.53	132.89	149.28	401,560	33.89%	11,851	176,906
2019							
December 31, 2019	216.20	164.12	204.94	401,261	33.86%	11,851	242,866
September 30, 2019	178.31	154.04	169.57	400,424	33.79%	11,851	200,951
June 30, 2019	195.96	168.62	174.45	388,570	32.79%	11,851	206,734
March 31, 2019	212.51	187.98	196.53	389,775	32.89%	11,851	232,900

Dividend and Bonus	2020		2019	
	Mln	%	Mln	%
Final cash dividend	17,775	150	5,925	50
3rd interim dividend	-	-	4,740	40
2nd interim dividend	-	-	4,740	40
1st interim dividend	5,925	50	4,740	40

MCB Turnover

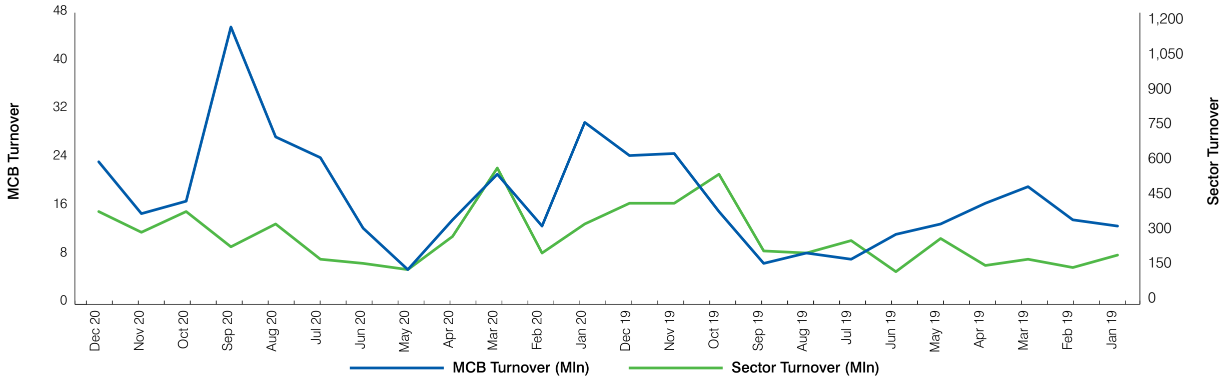

KSE 100 Index

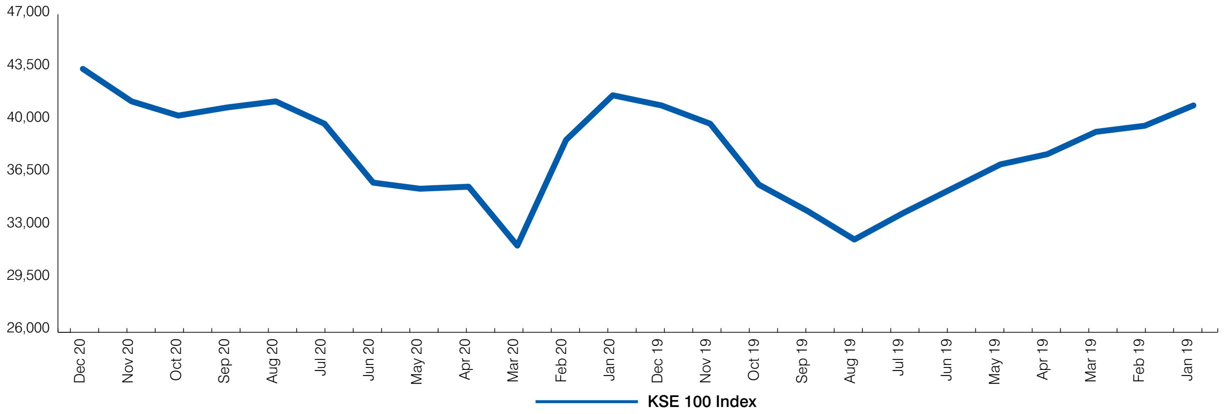

Share Price Sensitivity Analysis

Factors that can influence the share price of MCB Bank Limited are given below:

Discount rate / Monetary Policy

Based on different assessment parameters, the State Bank of Pakistan can change the monetary policy rate. Any volatility in the interest rates might impact revenue and profitability of the Bank.

Minimum Rate of Return on Deposits/Regulatory Risk

Any upward revision in the minimum deposit rate will result in compression in net interest margins earned due to increased cost of deposits. Such revision can negatively impact the earning and correspondingly the share price of the scrip.

Inflation

Inflation is considered as a key determinant for policy rate change. Any uptick in the inflation statistics will have a corresponding impact on the monetary policy rate. With higher discount rates, the Banks will be able to invest in high yielding investments, thus resulting in increased profitability. This, in turn will have a positive impact on the share price.

Political Stability & Law and order situation

Political stability and controlled law & order situation is a pre-requisite for any economic development. This in turn reposes investor confidence in the soils of Pakistan, making our corporates a potential investment opportunity. However, any act of terrorism or political instability can negatively impact the equity market and share prices of traded stocks.

Sensitivity Analysis of Change in Market Capitalization

Share Price as of December 31, 2020	Rs. 185.28
Market Capitalization as of December 31, 2020	Rs. 219,568 Millions
Change in Share Price by	Change in Market Capitalization
+10%	Rs. 21,957 Millions
-10%	Rs. (21,957) Millions

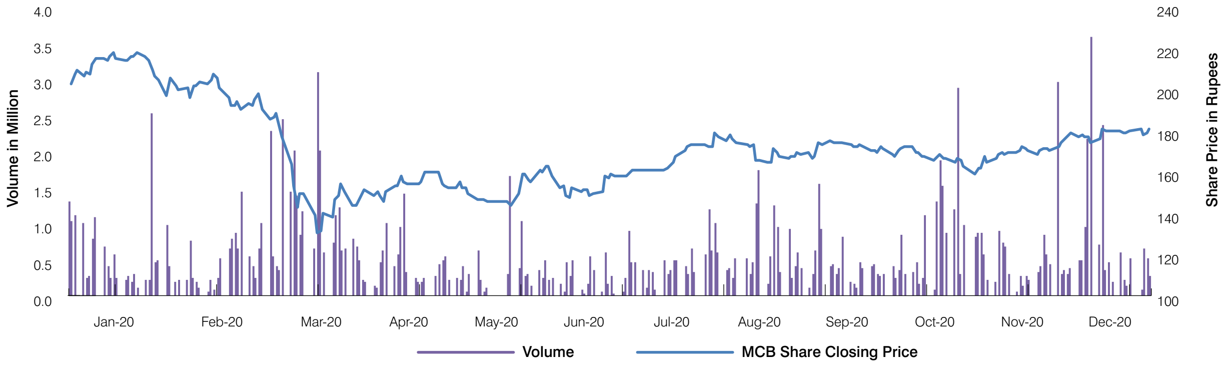

Other Information

Forced Sales Value in case of Revaluation of Property, Plant & Equipment

The Bank engages professionally qualified and independent valuers, with sufficient regularity, for carrying out revaluation of its land and buildings in order to ensure that their net carrying amounts do not differ materially from their fair value.

The latest of such exercises was conducted as at December 31, 2019, wherein an additional revaluation surplus of Rs. 7,290.966 million was recognized against land and buildings. The total market value and FSV of these land and buildings stood at Rs. 43,552.234 million and Rs. 34,841.787 million respectively.

Particulars of Significant/Material Assets and Immovable Property

MCB has sustained a strategic focus on strengthening its network, through branch expansion plan, in order to improve market coverage through increased customer touchpoints and widen the outreach of its service offerings; while simultaneously contributing towards the SBP's objective of expanding financial inclusion in the underbanked and unbanked segments of the society.

The Bank's network, as at December 31, 2020, extends across more than 1400 branches and offices. Of these, 356 premises are owned by the Bank and represent a material infrastructural investment of Rs. 40.016 billion towards immoveable properties (79% of total investment in Property and Equipment).

Reasons for Not Declaring Dividend despite Earning Profits and Future Prospects of Dividend:

The Board of Directors declared a final cash dividend of Rs. 15 per share for the year ended December 31, 2020, which is in addition to the Rs. 5 per share of interim dividend already paid to the shareholders for the quarter ended March 31, 2020, taking the dividend payout ratio to 81.63%. However during the year, as a precautionary measure to conserve capital and enhance the liquidity and stress absorption capacity of the banks during the COVID-19 pandemic, State Bank of Pakistan (SBP) vides its circular BPRD/BA&CPD/006315/20 dated April 22, 2020, advised the banks to suspend paying dividend for the first two quarters of 2020. Banks which had already approved dividend for the first quarter at the time of dissemination of instructions were advised by the SBP to suspend dividend distribution for the next two quarters i.e. ending June 2020 and September 2020. Hence, meticulously complying with the SBP's instructions, MCB did not declare dividend for the second and third quarters of 2020.

Dividend payout is expected for the year 2021 as the Bank manages this from the profitability generated while ensuring that sufficient capital buffers are available with the institution to meet regulatory requirements. Future prospects on the Bank's performance have been covered within the "Forward Looking" section of the Annual Report.

Outstanding & Overdue Payments on account of Taxes, Duties, Levies etc.

The Bank is a regular and timely payer of taxes, duties, levies etc. and there are no outstanding or overdue payments in regards thereof.

Management's Assessment of Sufficiency of Tax

The Bank maintains sufficient provision for taxation as required under the accounting standards and the relevant regulations. Contingencies with respect to the direct or indirect taxation have been disclosed in the Note 24 to these financial statements. Based on the comparisons of tax provision recognized in the financial statements for last three years vis-a-vis tax assessments, the management assesses that the provision of taxation maintained had been sufficient for its purposes.

Particulars of Loans/Advances and Investments in Foreign Companies or Undertakings

The Bank's overseas branches in Bahrain, UAE and Sri Lanka invest and lend to companies and undertakings operating in their jurisdictions. Details are given in note 42 of unconsolidated finance statements. Further the Bank hold 99.94% shareholding in "MCB Non-Bank Credit Organization "Closed Joint Stock Company" Azerbaijan.

Disclosures beyond BCR Criteria:

Over the recent years, there have been significant developments in the corporate reporting domain, particularly in relation to the amount of details included in the entities' annual reports, as well as the importance ascribed by users to the information beyond the audited financial statements and the auditor's report thereon.

There has been an evolution in the manner in which entities disseminate and communicate information to their stakeholders as users attach increasingly greater importance to supplementary information and look for better ways to inform their analysis and confirm understanding of more complex areas in the financial statements.

Considering this emerging necessity, MCB strives to go beyond the realm of essential reporting requirements in order to disclose all relevant information that facilitates the stakeholders in understanding various aspects of the Bank's operational and financial performance. Following is the list disclosures that have been made in addition to the BCR criteria:

- President/CEO review
- Statement on Internal Controls
- Customer Grievance Handling Mechanism
- Groups' Performance Reviews
- Staff Compensation/Remuneration Policy
- Detail of Management Committees

- Risk Management Framework
- Green Banking
- Six Years' – Non Financial Performance 2015-2020
- Six Years summary of operating expenses
- Six Years summary of markup and non-markup income
- Six Years' – Maturity of Assets & Liabilities
- Key interest bearing Assets and Liabilities
- Group-wise Advances and Deposits
- Top 10 Listed equity holding of the Bank
- Statement of Charity & Donations
- Investor Awareness through Jama Punji Initiative by the Securities & Exchange Commission of Pakistan

President's Review

The President/CEO's video message on the Bank's business performance and strategy is available at:
https://www.mcb.com.pk/investor-relations/ceo-message

Group Structure

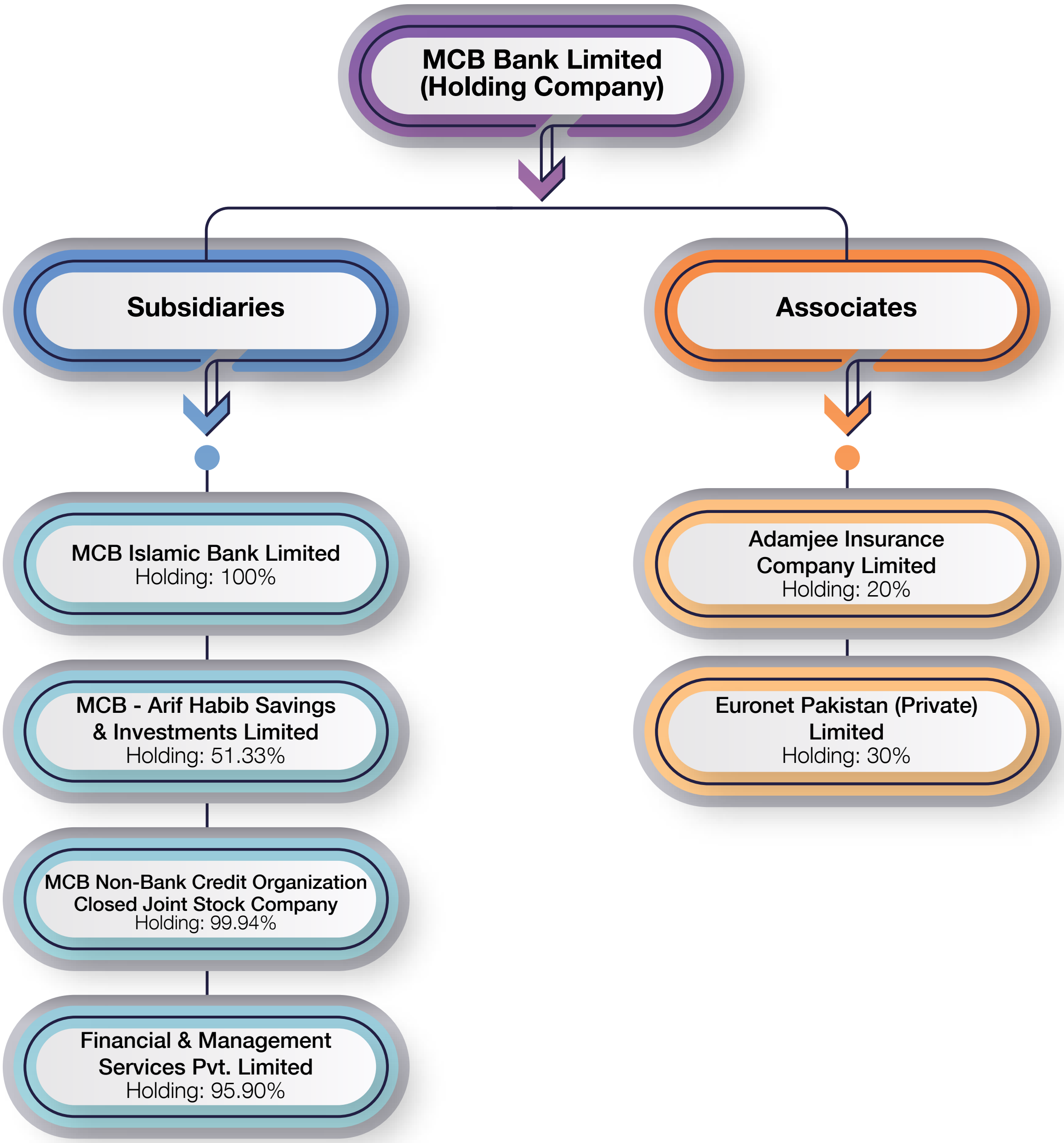

MCB Calendar of Major Events

Factors	Source
Annual Results - 2019 issued on	February 04, 2020
72nd Annual General Meeting held on	March 19, 2020
1st Quarter Results issued on	April 24, 2020
2nd Quarter Results issued on	August 20, 2020
3rd Quarter Results issued on	October 27, 2020
Annual Results - 2020 issued on	February 10, 2021
73rd Annual General Meeting scheduled on	March 27, 2021

Calendar of Major Events	
Incorporation	1947
Nationalisation	1974
Investment in First Women Bank	1989
Privatisation	1991
Incorporation of MCB Finanical Services Limited	1992
Incorporation of MNET Services (Private) Limited	2001
Investment in Adamjee Insurance Company Limited	2004
Incorporation of MCB Trade Services Limited	2005
Incorporation of MCB Asset Management Company Limited	2005
Change of name from Muslim Commercial Bank Limited	2005
Issuance and Listing of Global Depository Receipts on London Stock Exchange	2006
Strategic acquisition by Maybank	2008
Incorporation of MCB Leasing Closed Joint Stock Company	2009
Investment in Euronet Pakistan (Private) Limited	2011
Amalgamation of MCB Asset Management Company with Arif Habib Investment Limited	2011
Incorporation of MCB Islamic Banking Limited - a subsidiary company	2014
Merger of NIB Bank with and into MCB Bank Limited	2017
Merger of MNET Services (Private) Limited with and into MCB Bank Limited	2019
Disposal of MCB Financial Services Limited	2020

Strategic & Resource Allocation

Execution of a well-defined strategy has been the key pillar for our growth momentum over the last many years. Our strategy broadly covers what we want to achieve in the short and long run duly focusing on the challenges posed by the macroeconomic imbalances;

Strategic Objectives:

Short, medium and long term objectives of the Bank to meet its mission statements are as follows:

**Short term**	• Increase focus on digitalization and automation of processes to enhance efficiency, reduce cost and improve customer satisfaction and improve risk/ compliance standards;<br>• To ensure quality asset retention with measures to constantly decrease the NPLs base of the Bank;<br>• To increase the current account concentration levels of the institution by capitalizing on the opportunities presented.
**Medium term**	• To be a top stakeholder value generator in Pakistan's banking sector while remaining a socio-environmentally conscious citizen;<br>• The Bank aims to increase its share in the domestic deposit pie;<br>• To maintain a strong capital base;
**Long term**	• Delivering remarkable returns to stakeholders, sustainable performance exceeding market and shareholder expectations;<br>• Providing value added services through operational expansion, geography and upgraded system;<br>• Building a corporate culture of equality, trust and team spirit as we remain dedicated to being a socially responsible organization;

For strategy formulation, the Bank follows a structured approach to map itself in the industry / operating environment through detailed competitive position assessment, peer group analysis and macro-economic & thematic reviews; in turn laying the foundation for its future road map.

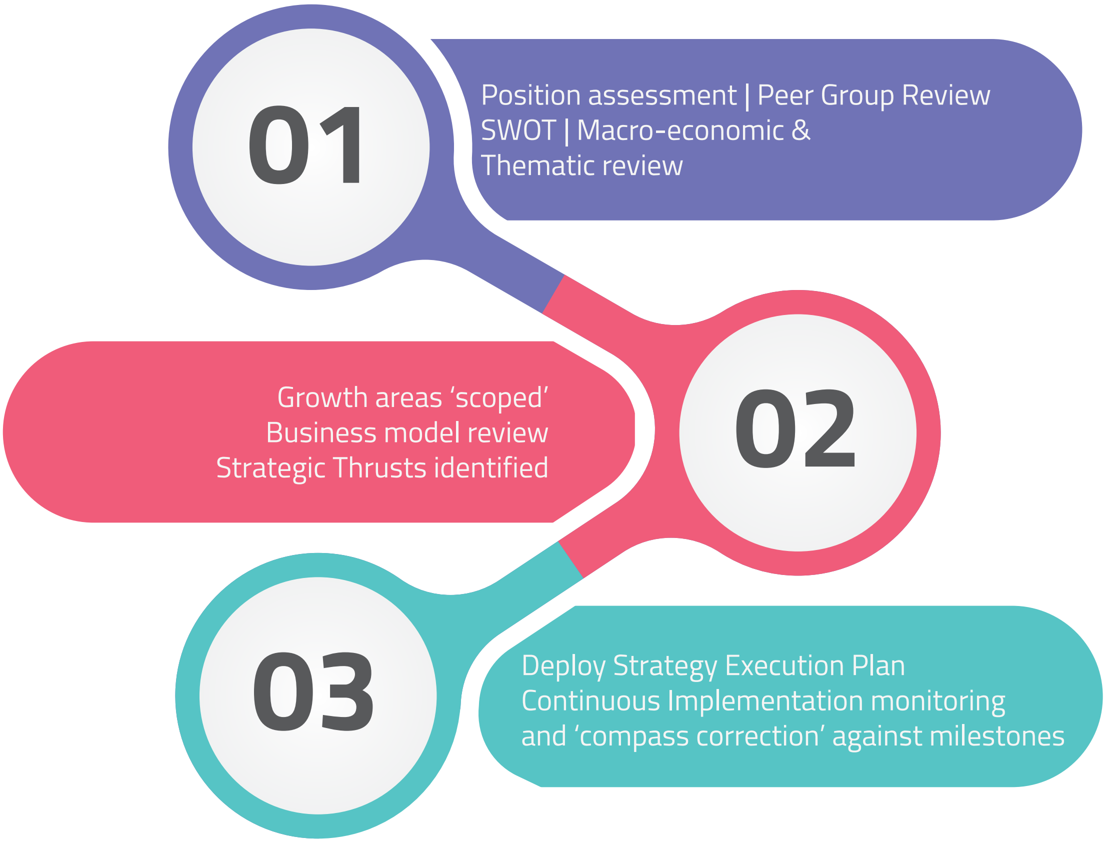

Strategies in place:

From customer service standpoint, special focus remains on improving our service quality and service standards levels. We will integrate service standards across all the channels and outlets in the network to provide a uniform customer experience.

We will endeavor to meet expectations of our valued customer base. Another important aspect remains credit quality and our refined risk appetite. We will thereby give value to our customers across the entire spectrum of retail, corporate and SME while improving our asset quality.

Under the Corporate plan, we also intend to expand our geographical boundaries by being open to potential new business models, innovative processes and delivery channels, enabling provision of 24x7 customer services.

Today, technology is a major component of the competitive edge of any bank. With millennial being an increasing percentage of our customer base, we have to cater to their expectations and it requires leveraging cutting edge technology. The other side of the coin however, is that technology brings risks with it. We will go the extra mile, to ensure our assets and those of our customers are secure and sensitive information is protected.

To ensure efficient and effective operation of the Bank we need systems and processes that operate seamlessly. This is another focus area where we will concentrate on identifying pain points and gaps, and make the necessary modifications.

To safeguard the financial stability and the reputation of the Bank, good governance and ethical conduct are imperative. While we already have high standards in these areas, we have set our benchmarks as the best in class practices in the country. We will renew and re-energise our focus on sustainability by ensuring that we continue to maintain a judicious balance between economic, social and environmental objectives.

The end result of all the above will be the enhancement of our brand. The brand is a mirror of our image in the eyes of our customers, both legacy and millennials. Through the strategies spelt out in our corporate plan we will forge ahead, building our brand, increasing our assets and profitability, while delivering increasing value to all our stakeholders.

Method and Assumptions in Compiling Indicators

The Bank identifies its indicators which effectively reflect Bank's performance.

The bank analyses its market positioning, competitors and general market conditions while compiling its indicators. The Bank analyses deposits, advances, capital and risk adequacy ratios, gross profit after tax and EPS on a regular basis to gauge its performance. These are the basic indicators of Bank's financial performance and profitability.

Market price is a measure of Bank's perception in the market. The difference between book value and market value shows investor's confidence on the script.

The Bank manages its dividend payout in line with the profitability generated during the year while ensuring that sufficient capital buffers are available with the institution to meet regulatory requirements. Dividend is the amount allocated out of profit for paying cash to the shareholders. The dividend payment is an indicator of how well earnings support the dividends. The Bank takes its decisions of cash or stock dividend based on market conditions, share price and governing laws and regulations.

Comparing cash flow from operating activities with profit before tax can give insights into how a Bank generates funds and manage the cash flows. The bank regularly analyses its cash flows and strives to keep it on positive side.

Change in Indicators and Performance Measures:

Key performance indicators (KPIs) provide understanding of the Bank's performance in key areas. These indicators are used as a gauge to analyze current standing of the Bank and likely path the Bank would follow.

MCB has identified KPIs that are critical to its business. While identifying KPIs, the Bank analyzed various indicators, their interpretations and accordingly the extent to which they may correctly and clearly communicate the Bank's performance. Change in important indicators is discussed in performance and position section.

Key performance indicators to measure the objectives are as follows:

Strategic objectives	Strategies for Meeting Objectives	KPI	Future relevance
Increase focus on digitalization and automation of process to enhance efficiency, reduce cost and improve customer satisfaction and improve risk and compliance standards.	Centralization and monitoring of operating expenses to restrain them within conventional limits.<br><br>Work on automation of existing manual systems.<br>Gradual investment on unified digital platform for an overwhelming customer experience.<br><br>Manage Information security risks through development, documentation and implementation of policies, standards, procedures and guidelines that ensure confidentiality and integrity of sensitive information.	Effective and efficient cost control	The KPI will remain relevant in the future.
To ensure quality asset retention with measures to constantly decrease the NPLs base of the Bank.	Increased focus on quality asset growth while maintaining low infection ratio.<br><br>Focus on recoveries of existing NPL stock.	Asset Quality	The KPI will remain relevant in the future.
To maintain a strong capital base	Healthy equity leading to maintain strong capital adequacy ratios.	Capital ratios	The KPI will remain relevant in the future.
The Bank aims to increase its share in the domestic deposit pie; and<br><br>To increase the current account concentration levels of the institution by capitalizing on the opportunities presented	Expansion/Increase in deposit base through new products and markets thereby increasing customer base beyond the prevalent organic growth.<br>Increased focus on current account growth.	Deposit generation	The KPI will remain relevant in the future.
Delivering remarkable returns to stakeholders, sustainable performance exceeding market and shareholder expectations.	Higher profitability to pay higher returns to shareholders.	Shareholder return	The KPI will remain relevant in the future.
Providing value added services through operational expansion, geography and upgraded system.	Lead market position through focused initiatives targeting new products, new markets, branchless banking and effective cost management.<br>Rationalize & optimize usage of existing branch network and network strengthening through branch expansion plan.<br>Service portfolio enhancement of all digital products / channels i.e. call center, internet banking, mobile banking, mobile wallet, SMS alerts, E-statement, ATMs and Debit Cards to increase non markup income.<br>Integrate service standards across all the channels and outlets in the network to provide a uniform customer experience.	Improved services	The KPI will remain relevant in the future.

Building a corporate culture of equality, trust and team spirit as we remain dedicated to being a socially responsible organization.	Improve governance structure and update existing policies as per industry dynamics.<br>Generate economic activity through sustainable focused initiatives.	Corporate culture	The KPI will remain relevant in the future.
To be a top stakeholder value generator in Pakistan's banking sector while remaining a socio-environmentally conscious citizen.	Higher profitability to pay higher returns to shareholders.<br>Introduce socio environmental activities such as green banking to improve the brand name.	Corporate social responsibility	The KPI will remain relevant in the future.

Resource Allocation Plan:

The inputs to the Bank's business processes are capitals, or stores of value, in various forms. Of these capitals, financial, manufactured and intellectual capitals are internal capitals owned by the Bank. The others, human, social, relationship and natural are external to the Bank.

Bank has resources to meet the strategic objectives. A transitory resource allocation plan is as follows:

Nature of capital	Resource allocation plan
Human capital	• Redeploy human capital to enhance productivity through segmentation;<br>• Undertake human capital capacity enhancement and capability building by focusing on trainings, talent management and talent retention;<br>• Onboard versatile staff that can multi task easily.
Manufactured capital	• Branch layout improvement and widening of branch and ATM network;<br>• Re-align the business model through segmentation to increase the customer base.
Financial Capital	• Investment on process automation and IT network improvements;<br>• Invest on infrastructure including buildings and equipment;<br>• Invest in good quality asset base with high yield.
Natural capital	• Introduce green building concept to branch network by introducing paperless environment and install solar energy equipment in branches;<br>• Increase financing to renewable energy projects.
Intellectual Capital	• Reengineering of processes to capture synergies and customer satisfaction;<br>• Improvement in governance culture by utilizing over 73 years of institutionalized knowledge;<br>• Introduce new products as per customer needs.
Social and Relationship Capital	• Invest in a series of initiatives that enhance collaboration and ongoing dialogue with our customers;<br>• Enhance brand image through public awareness campaigns;

Strategy to overcome liquidity problem:

Bank carries a substantial portfolio of marketable securities that can be easily traded and realized in known amounts of cash in the event of liquidity stress. Bank's Liquidity Coverage Ratio and Net Stable Funding Ratio are well over and above the regulatory requirement.

The Bank maintains strong liquidity position which is regularly monitored by the respective units. The liquidity ratios indicate the strong liquidity position of the institution. Liquidity position of the bank is discussed in the risk management section of the financial statements.

Significant Plans and Decisions

During the year, the Bank, in line with the shareholder approval, disposed of its ownership stake in the wholly owned subsidiary namely MCB Financial Services Limited.

Except as mentioned above, there was no other significant event during the year.

Significant changes in objectives and strategies:

MCB Bank objectives & strategies are well planned and are persistently implemented. No significant change occurred during the year to affect the objective and business strategies.

Business Model

Inputs

Financial Capital

- Share Holders Equity: Rs. 162 billion
- Customer Deposits: Rs. 1,290 billion
- Strong CET1 Capital Ratio: 17.03%; well above Minimum Regulatory Requirements.

Human Capital

- A Total of 13,643 Employees that are:
  - Skilled, Experienced and Highly Competent
  - Client Driven & People Centric
  - Increasingly Innovative & Competitive
  - Strong in Compliance & Governance
- Reward Structures Linked to Performance.
- Training and Development Programs.

Manufactured Capital

- Wide Outreach: 1,441 Branches
- Digital Touchpoints: 1,434 ATMs, POS, CDM, Call Centers etc.
- Strong Core Banking Systems
- Market Leading Digital Products, Services and Client Value Propositions
- Other Fixed Assets

Natural Capital

- Green Banking Initiatives.
- Financing of Renewable Energy Projects.
- Renewable and Non-Renewable Resources used by the Bank i.e. Electricity, Fuel, Solar Powered installations (10 Branches) etc.

Intellectual Capital

- Robust Heritage and a Strong Brand Image
- Strong Management Structure and Experienced Leadership Team
- Systems, Processes and Procedures established by the Bank

Social and Relationship Capital

- No. of Accounts: 8,217 Million
- No. of Customers: 7 million+
- Banking Agents, Trade Partners and Merchants across a Diffused Geographical Presence
- Robust relationships Created with all Stakeholders.

Business Activities

- **Customer Centric Focus for an Enriched and Bespoke Experience.**
- **Increased Focus on Digitization and Process Automation .**
- **Providing Value Added Services through Operational & Geographical Expansion, Data Analytics and System Upgrades.**
- **Building a Corporate Culture of Equality, Trust and Team Spirit to Remain a Socially Responsible Citizen**
- **Ensuring Quality Asset Retention through Robust Risk Management and Improving Deposit Mobilization**
- **Generating NIM on Earning Assets and Augmenting Non Markup Avenues for Maximizing Stakeholder Value.**
- **Undertaking Human Capital Capacity Enhancement and Capability Building through Trainings, Talent Management and Talent Retention**
- **Improving Governance Culture by Capitalizing on Institutionalized Knowledge**

Outputs & Outcomes

Financial Capital

- Profit after Tax: Rs. 29 billion
- Earning per share: 24.50 / Share
- Dividend per share: Rs. 20 / Share
- Dividend Payout ratio: 81.62%
- Return on Equity (p.a): 18.88%
- Return on Assets (p.a): 1.77%

Human Capital

- Female Staff Ratio: 16.3%
- New Recruitments: 1,430 Employees
- Investment in Trainings: Rs. 36.51 Million
- No. of Training Participants: 30,163
- No. of Promotions: 2,356 Employees

Social & Relationship Capital

- Contribution to National Exchequer: Rs. 16.08 Billion
- Continued Socio Economic Spending
- Deposit Mobilization: 13% Growth
- Long Term Entity Credit Rating: AAA
- Short Term Entity Credit Rating: A1+
- Total Complaints Resolved: 112,270
- Complaint Resolution Rate: 99.05%
- Low Infection Ratio: 9.97%
- Online Customers: 10.3% Growth

Natural Capital

- Improved Energy Mix and Conservation through reduction in Non-Renewable energy consumption: 13 tons of Co2 Emissions avoided as of 2020
- World Wide Fund for Nature "Green Office Certification" - Head Office Building.

Intellectual Capital

- Digital Initiatives for all Customers
- Responsibly & Actively Contributed to Governments Key Pandemic Responses for Credit Extension and Provision of Essential Banking Services to the General Public.
- Adjudged as the "Overall Most Outstanding Company in Pakistan" by Asiamoney's .

Value Chain

Value Chain Framework

Support Activities

overnance and risk management:

- Maintaining Regulatory compliance
- Increased Corporate governance
- Risk Management Framework
- Implementation of Internal controls

Technological developments:

- Increased focus on digitization
- Decision support system
- Automation of existing manual systems
- Highest security standards

Human capital management:

- Workforce planning
- Fair recruitment and selection policies
- Employee training and development
- Robust performance & reward system

Finance and reporting:

- Compliance with applicable Accounting standards
- Financial reporting on regulatory guidelines
- MIS for improved decision-making

Primary Activities

Deposit mobilization and distribution network:

- Focus on low cost deposits and improving CASA mix
- Diversified portfolio of products
- Effective use of distribution outreach and customer touchpoints

Asset Management and portfolio enhancement:

- Increased focus on quality asset growth
- Recoveries of Non performing loans (NPL)
- Maintaining low infection ratio
- Service portfolio enhancement

Value created for stakeholders:

- Contribution to the national exchequer
- Shareholder value maximization
- Consistent payout ratio
- Corporate social responsibility

Marketing, sales and transactions:

- Strong frontline sales force
- Marketing and communications
- Customer service and complaint management

Competitive Landscape and Market Positioning

Factors	Implication	Corresponding Strategies
**Threat of new entrants and substitute products or services**<br>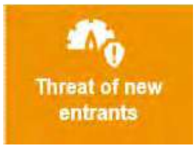	The large amounts of capital required to setup a bank along with the length of time consumed to establish a significant brand loyalty and the need to adhere to strict regulatory stipulations serve as strong entry barrier for new entrants.<br><br>However, the domestic landscape has been evolving to include Fintech participants that are focusing on transactional services and innovative digital solutions for revolutionizing the customer banking experience.	Despite the imminent threat of new entrants and emerging Fintech disruptions, MCB is countering these threats by engaging in the following activities:<br>• improving customer-brand relationship that goes beyond the minimum transactional services to retain customer loyalty;<br>• investing substantially in digital platforms for improving customer convenience;<br>• launching new products and services that cater to a diversified customer base; and<br>• adhering with all regulatory guidelines with a view to ensure the highest levels of compliance.
**Bargaining power of customers**<br>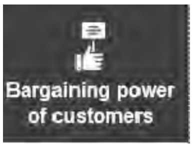	It is reasonably easy for retail customers to switch to other banks fully, or even avail part of their service requirements alternatively due to the low switching costs involved, hence shrinking the size of banking engagement. However, their bargaining power stays limited due to the minimal impact on the Bank's bottom line.<br><br>The bargaining power of larger groups, corporate clients and high net worth individuals is comparatively greater since the rising competition has increased customers price sensitivity and the loss of sizable accounts and sources of revenue from them can substantially impact bank's profitability.	MCB addresses the issue of customer bargaining power primarily by focusing on clearly directed customer retention and acquisition strategies; customer service standards are being continuously augmented, services are being tailored to suit individual needs, innovative solutions are being devised to make customer experiences more enjoyable, convenient and hassle-free and market competitive rates are being offered to effectively increase switching costs for customer
**Bargaining power of suppliers**<br>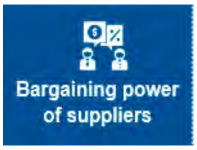	MCB's suppliers primarily comprise of its deposit-holders who are the Bank's key resource for capital and its employees, also known as the resource of labor.<br><br>In an industry scenario with low differentiation, it is easy for the primary deposit-holder group to switch to other banks, tempted by higher rates and better service standards. Further, share of deposits is highly concentrated, which gives them excess bargaining power.<br><br>With a view to fund gaps in the Bank's borrowings, MCB mobilizes debt from other financial institutions, with rates being largely market-driven. Hence, their bargaining power is often considered to be medium to high.<br><br>When it comes to the bargaining power of suppliers of labor, individual employees baring major executives have little bargaining power	MCB has embraced following strategies in order to derive an edge over its supplier relationships:<br>• providing a high degree safety to its capital providers including investors, deposit-holders and other banking partners; A point further re-enforced by Bank's highest local credit ratings of AAA/A1+ for long term and short term debt respectively;<br>• creating mutually-beneficial solutions across the entire engagement spectrum; and<br>• sustaining employee retention focus by offering a challenging, learning and conductive work environment which is duly complemented by career progression opportunities and market competitive salary and benefit packages.
**Intensity of competitive rivalry**<br>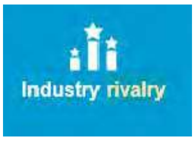	Intensity of competition within the domestic banking sector has grown over the years. Banks have focused on developing a wide range of asset and liability product offerings at competitive rates in order to reinforce their customer acquisition strategies and attract existing market share.<br><br>The relatively low switching cost from one bank to another has further intensified the importance of competition within the industry, especially in the retail and commercial banking spheres.<br><br>Traditional banking operating models have been disrupted as the outbreak of COVID-19 has challenged the existing banking landscape and paved way for the industry to adopt emerging technologies in rendering digital financial services amidst responsibly enforced social distancing and containment measures.	MCB has initiated following measures with a view to consolidating and further improving its market share:<br>• by distinguishing itself in the marketplace primarily on the basis of its history, experience and brand image; and<br>• staying on the cutting edge of offering customer convenience and low-cost banking solutions.

Risk Management Framework

Risk is an inherent part of banking business activities. The risk management framework and governance structure at MCB helps to mitigate and counter any foreseeable risk in its various lines of business. Risk awareness forms an integral part of strategic and operational activities of risk management. Through its Global Risk Management Policy, Bank sets the best course of action under uncertainty by identifying, prioritizing, mitigating and monitoring risk issues, with the goal of enhancing shareholders' value. Bank's risk management structure is based on the following five guiding principles:

- Optimizing risk/return in a controlled manner
- Establishing clear responsibility and accountability
- Establishing & maintaining independent and properly resourced risk management function.
- Promoting an open risk culture
- Adopting international best practices in risk management

The Bank executes its risk strategy and undertakes controlled risk-taking activities within its risk management framework. The Board of Directors (BOD) at MCB Bank Limited actively drive the risk management framework . Under the valuable guidance of BOD, the Bank has a proactive approach in dealing with factors that influence the financial standing of the bank, to generate recurrent earnings and to maximize shareholder's value by achieving an appropriate trade-off between risk and returns. An effective risk management framework along-with a robust risk governance structure, strong capital & liquidity coupled with a good quality of credit portfolio remains a cornerstone of the Bank's risk management goals.

Empowerment and independence are the basic principles in risk management and it is implemented as a fundamental part of BOD's vision. Independence of areas that are responsible for measuring, analyzing, controlling and monitoring risk from the frontline risk takers (i.e. business soliciting groups) is ensured within the Bank. In line with this principle, Group Head-Risk Management functionally reports to the "Risk Management & Portfolio Review Committee" (RM&PRC) which is the sub-committee of the BOD and administratively to the President.

Risk takers and Risk controllers have independent reporting lines, yet work together to increase Bank's value via efficient utilization of capital. Through a four eye principle for credit approval levels for corporate and retail banking, all exposure related requests are approved with the formal consent of at least two authorized individuals including one from the business side having credit approval authority and the other from risk management side having credit review authority. The BOD and its RM&PRC have ensured formulation and implementation of a comprehensive risk management framework. Under the BOD's guidance, the Bank executed an effective risk strategy and continued to undertake controlled risk-taking activities within the risk management framework; combining core policies, procedures and process design with active portfolio management. The risk management framework requires strong integrated risk management practices in key strategic, capital and financial planning processes and day-to-day business processes across the organization, with a goal to ensure that risks are appropriately considered, evaluated and responded to in a timely manner.

As a matter of principle, the Bank constantly endeavors to improve its risk management framework in the light of the international best practices and regulatory guidelines. Accordingly, all policies and procedural documents that form part of the Bank's risk management framework are regularly reviewed to keep them aligned with changing market dynamics, regulatory environment and international standards.

The RM & PRC guides the management on its risk taking activities within the policy framework approved by the BOD. Regular meetings of RM&PRC are convened to oversee the risk exposures and their trends as a result of the various initiatives undertaken by the Bank. The committee reviews different aspects of the loan portfolio which, among others, includes asset growth, credit quality, credit concentration, lending business trend and cross sectional analysis. Review of various aspects of country risk, liquidity risk, market risk covering interest rate risk, foreign exchange risk, equity price risk, technology risk along with the stress-testing is also a regular feature. Operational risk assessments, key risk indicators and major findings of Risk & Control Self-Assessment (RCSA) pertaining to processes, people, systems, technology and reputation are also regularly reviewed by the committee. The committee also reviews in detail the Bank's capital levels under Internal Capital Adequacy Assessment Process (ICAAP) and Capital Adequacy Ratio.

The Management Credit & Risk Committee is the management platform for discussion and deliberation on key risk issues in the portfolio. Regular meetings of the committee are convened to oversee the risk exposures in the portfolio of the Bank.

Credit risk review ensures to minimize credit risk associated at account and portfolio level. During the year 2020, the Bank continued with the policy to remain selective in disbursing its loan to low risk customers across all the industries &

maintains a fairly diversified loan portfolio. Risk Review successfully managed to evaluate and approve increased number of loan requests, within required turnaround time, both for domestic and international operations. An in-house request tracking & turnaround time monitoring software ensures tracking of proposals and monitoring of turnaround-time of credit proposals routed through the Risk Management Group. The Bank's implemented Loan Origination System (LOS) for end to end automation of credit approval process facilitates effective management of internal policies and controls as well as regulatory requirements while also contributing towards its transition to a paperless environment under the Green Banking initiative.

For risk categorized as sovereign/ government risk, the lending exposure is spread over multiple government owned or controlled organizations and departments which are engaged in a variety of tasks that range from different development related works to utility distribution and production. To manage adverse outcomes in terms of unfavorable scenarios, multiple control factors in the lending structure of the Bank provide additional comfort and support. Such controls range from quality of eligible collateral, pre-disbursement safety measures to post disbursement monitoring.

In order to further enhance the credit risk analysis, the bank has in place a probability of default based Internal Credit Risk Rating (ICRR) system which is based on statistical modeling and validation in line with Basel principles. The ICRR is currently focused on corporate and commercial customer categories. Furthermore, another ICRR model is also in place for rating of SE & ME and Agri. customers. A model for facility risk rating has also been implemented which reflects expected loss rate of a credit facility.

In addition to the credit risk, like all financial institutions, MCB is also exposed to market risk through its trading and other investment activities. A comprehensive control structure is in place to ensure that the Bank does not exceed its qualitative and quantitative tolerance for market risk. A number of metrics like VaR methodologies complemented by sensitivity measures, notional limits, and stop loss triggers at portfolio level/asset class and stress testing are used to capture and report the multi-dimensional aspects of market risk.

As an authorized derivatives dealer, the Bank is an active participant in the derivatives market. Overall limits in derivatives are approved by the BOD. Counter party limits structure for derivatives transactions is in place and exposures are monitored and reported on a continuous basis.

Operational Risk is being managed professionally in accordance with the Global Risk Management Policy, Policy on Internal Controls, Operational Risk Management Framework and various regulatory instructions. Operational Risk Inventory database covering losses, control breaches and near miss events is being maintained using professionally developed software. Operational risk events and Key Risk Indicators (KRI's) are captured and management reports are generated. A process of Risk and Control Self-Assessment (RCSA) is in place to assess the operating effectiveness of controls and to implement remedial measures as needed. Updates on operational risk events are presented to the senior management and RM&PRC of the BOD on quarterly basis.

The Bank has developed Information Technology Risk Assessment Framework which enables better management of technology risk managed by IT Risk team. The Bank has an internal operational risk awareness program which is aimed at building capacity and inculcating risk aware culture in the staff through workshops and on-job awareness.

Capital Structure of the Bank

The Bank remained a well-capitalized institution with a capital base well above the regulatory limits and capital requirements under BASEL frameworks. The Bank continues with a policy of sufficient profit retention to increase its risk absorption capacity. Bank's total Capital Adequacy Ratio is 20.98% against the requirement of 11.50% (including capital conservation buffer of 1.50%). Quality of the capital is evident from Bank's Common Equity Tier-1 (CET1) to total risk weighted assets ratio which comes to 17.03% against the requirement of 6%. The Bank maintained the leverage ratio of 7.03% which is well above the regulatory limit of 3.0%.

Liquidity Management and Strategy to Overcome Liquidity Position

The Asset Liability Management Committee of the Bank has the responsibility for formulation of overall strategy and oversight of the Asset Liability Management (ALM) function. BOD has approved a comprehensive Liquidity Risk Policy (part of the Global Risk Management Policy), which stipulates policies regarding maintenance of various ratios, funding preferences and evaluation of Banks' liquidity under normal and stress scenarios. The underlying policies and procedures are reviewed and approved regularly at the senior management and BOD levels covering the Global Risk Management

Policy, Global Treasury Policy, Investment Policy and Liquidity Strategy including Contingency Funding Plan.

Bank's comprehensive liquidity management framework assists it to closely watch the liquidity position through monitoring of early warning indicators and stress testing in order to ensure effective and timely decision making. The liquidity risk management approach at the Bank involves intra-day liquidity management, managing funding sources and evaluation of structural imbalances in the statement of financial position. A large and stable customer deposits base, along with a strong capital base provides strength and support for maintenance of a strong liquidity position. The Bank also has a substantial portfolio of marketable securities that can be realized in the event of liquidity stress.

Further, in line with SBP's directives, the Bank has fully implemented BASEL III required liquidity standards and maintains liquidity ratios. The Bank reported Liquidity Coverage Ratio (LCR) of 237.72% and Net Stable Funding Ratio (NSFR) of 174.86% against a requirement of 100%.

Sensitivity Analysis due to Foreign Currency Fluctuation

The PKR depreciated by around 3.22% against the US Dollar in 2020. Foreign exchange risk exposes the bank to changes in the value of exposure denominated in foreign currencies due to the exchange rate fluctuation and volatility. The types of the instruments exposed to this risk are mainly investments in foreign branches, advances and deposits denominated in foreign currency, cash flows in foreign currencies arising from foreign exchange transactions etc.

The core objective of the foreign exchange risk management is to ensure that the foreign exchange exposure of the Bank remains within defined risk appetite and insulates the Bank against undue losses that may arise due to volatile movements in foreign exchange rates or interest rates.

Limit structure to manage foreign exchange risk including gap limits in different tenors in major currencies are in place to control captioned risk. Bank's net open position and Foreign Exchange Exposure Limits (FEEL) is monitored and reported on intra-day and day end basis. Foreign exchange risk parameters including VaR are generated and monitored on a daily basis. Stress testing of foreign exchange portfolio and its reporting to senior management and RM&PRC of the BOD is also a regular feature.

Impact of 1% change in foreign exchange rates on the Profit and loss account:

2020		2019	
Banking Book	Trading Book	Banking Book	Trading Book
(Rs. 000)			
–	1,792	–	9,267

Risk and Opportunity Report

At MCB, a comprehensive Risk Management Framework around an approved risk appetite is in place. Mechanisms are defined for every identified risk to ensure that the Bank continuously evaluates the associated risk and ensures presence of operational mitigating controls. The Bank remains committed to exploring every possible opportunity to translate it into revenues / returns for the stakeholders, while making sure that the related risk is adequately managed.

Risk Governance Model

- Board of Directors (BOD)
- Risk Management & Portfolio Review Committee (RM&PRC)
- Management Credit & Risk Committee (MC&RC)
- Group Head – Risk Management

Graphical presentation of risk governance structure to is as follows:

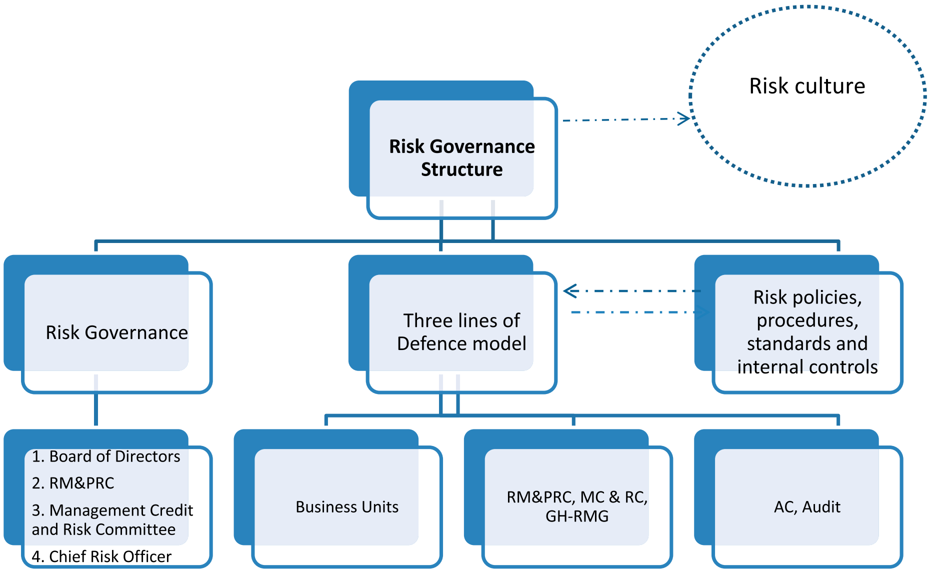

Three lines of Defence model

The Bank has a well-structured Risk Management model based on three lines of defense which are independent of each other. Each line of defense is executed by different organizational units. The first line of defense consists of business divisions and support units from whose activities the risks arise. RM&PRC being the second line of defense develops frameworks, policies, procedures and establishes risk appetite. Periodical stress testing and continuous monitoring are also an integral part of the second line of defense. The third is the audit and compliance functions which offer an independent oversight.

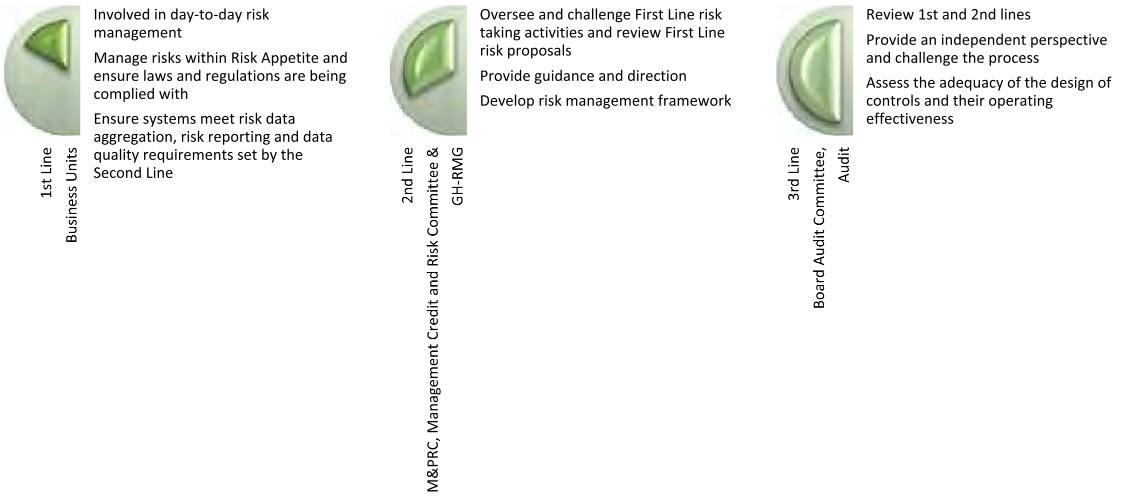

Assessment of the principal risks facing the Bank by the Boards of Directors:

The BOD's have carried out a robust assessment of the principal risks facing the Bank, including those that would threaten the business model, future performance, solvency or liquidity.

Bank has identified the following risks after analyzing the external and internal factors:

Factors	Source	Risks
**Economic**	External	**Market risks:** The risk of loss arising from potential adverse changes in the value of the Bank's assets and liabilities from fluctuation in market variables including, but not limited to, interest rates, foreign exchange, equity prices, commodity prices, credit spreads, implied volatilities and asset correlations.
	External	**Market risks:** The risk of loss arising from potential adverse changes in the value of the Bank's assets and liabilities from fluctuation in market variables including, but not limited to, interest rates, foreign exchange, equity prices, commodity prices, credit spreads, implied volatilities and asset correlations.
	External	**Credit risk:** The risk of loss to the bank from the failure of clients, customers or counterparties, including sovereigns, to fully honour their obligations, including the whole and timely payment of principal, interest, collateral and other receivables.
	External/Internal	**Liquidity risk:** The risk that the bank is unable to meet its contractual or contingent obligations or that it does not have the appropriate amount, tenor and composition of funding and liquidity to support its assets.
**Technology/ Systems**	Internal/ External	**Technological /Information Security Risk:**<br>Technology risks having potential impact due to technology disruption or failure to disrupt bank's business process posing adverse impact on Confidentiality, Integrity and Availability of MCB's technology environment.<br>Information Technology Risk Assessment helps the management to identify and manage the key risks, potential threats and associated vulnerabilities to the critical primary & secondary IT systems and applications of the Bank.<br>**Operational Risk**<br>The risk of loss to the Bank from inadequate or failed processes or systems, human factors or due to internal/external events (e.g. fraud) where the root cause is not due to credit or market risks.
**Political**	External	**Country risk:** Political stability and controlled law & order situation is a pre-requisite for any economic development and reposes investor confidence in the country, providing corporates a potential investment opportunity. However, political instability can negatively impact the economy /equity market, thus resulting in decreased profitability.
**Regulator**	Internal/External	**Regulatory Risk:** The risk of loss or imposition of penalties, damages or fines from the failure of the firm to meet its legal obligations including regulatory or contractual requirements.<br>Key sources of uncertainty include expected regulatory requirements specifically implementation of IFRS 9 in Pakistan, which may have negative impact on the bottom line of the banks.
**Social**	Internal/External	**Reputation risk:** The risk that an action, transaction, investment or event will reduce trust in the Bank's integrity and competence by clients, counterparties, investors, regulators, employees or the public.

Materiality Approach

Matters are considered to be material if, individually or in aggregate, they are expected to significantly affect the reputation, performance and profitability of the Bank. The materiality process helps to navigate the complex landscape of stakeholder expectations, risks and opportunities. The BOD of the Bank has approved Materiality Policy for the Bank.

Summarized risks, opportunities and related mitigating factors are documented below:-

Risk type	Materiality Rating	Probability of Risk Occurrence	Strategy	Impacted CAPITAL
**Market Risk**	High	Medium probability	**Measurement:** Bank is exposed to market risk through its trading and other investment activities. Metrics like VaR methodologies complemented by sensitivity measures, notional limits, loss triggers at a detailed portfolio level and stress testing are used to capture and report the multi-dimensional aspects of market risk.<br><br>**Monitoring:** A comprehensive structure, ensuring the bank does not exceed its qualitative and quantitative tolerance for market risk, is in place.<br><br>**Management:** The bank has followed a conservative and balanced approach towards risk taking in the market risk area. The robust risk management architecture ensures that the exposures remain within the defined risk appetite.<br><br>Furthermore, a comprehensive control structure is in place to ensure that the Bank does not exceed its qualitative and quantitative tolerance for market risk. A number of metrics like VaR methodologies complemented by sensitivity measures, notional limits, stop loss triggers at portfolio level/asset class, and stress testing are used to capture and report the multi-dimensional aspects of market risk.	Financial
**Capital Adequacy Risk**	High	Medium probability	**Measurement:** The Bank is a well-capitalized institution with a capital base well above the regulatory limits and Basel-III requirements.<br><br>**Monitoring:** The Bank regularly assesses the capital requirements and ensures that the minimum capital requirements specified by the State Bank are adhered to. Internal Capital Adequacy Assessment is a regular activity. Stress levels of major risks are assessed against the minimum capital requirement.<br><br>Regular assessment of capital enables an evaluation of the amount, type and distribution of capital required to cover these risks.<br><br>**Management:** The Bank remained a well-capitalized institution with a capital base well above the regulatory limits and capital requirements under BASEL frameworks. The Bank continues with a policy of sufficient profit retention to increase its risk absorption capacity. Bank's total Capital Adequacy Ratio is 20.98% against the requirement of 11.50% (including capital conservation buffer of 1.50%). Quality of the capital is evident from Bank's Common Equity Tier-1 (CET1) to total risk weighted assets ratio which comes to 17.03% against the requirement of 6%. The bank maintained a leverage ratio of 7.03% which is well above the regulatory limit of 3.0%.<br><br>Going-concern capital requirements are assessed on a forward-looking basis – including as part of the annual budgeting process. These assessments consider the resilience of capital adequacy and leverage ratios under a range of hypothetical future states. The assessments incorporate assumptions regarding a range of regulatory and accounting aspects, such as IFRS 9, taking account of a number of factors including economic variables and impairments.<br><br>The Bank will continue the policy of sufficient profit retention to increase its risk taking capacity and capitalize opportunities to protect the interests of stakeholders in the short, medium and long term.	Financial

Risk type	Materiality Rating	Probability of Risk Occurrence	Strategy	Impacted CAPITAL
**Credit Risk**	High	Medium probability	**Measurement:** Credit Risk Management function identifies, measures, manages, monitors and mitigates credit risk. Credit Risk is measured and estimated through detailed financial and non-financial analyses, internal and external credit risk ratings and customers' behavior analysis. Stress testing of top customers in credit portfolio is also carried out regularly.<br><br>**Monitoring:** Credit Risk Management organizational structure ensures pre and post-facto management of credit risk. Credit Review function carries out pre-fact evaluation of counterparties & the credit structures and hindsight reviews, the Credit Risk Control (CRC) function performs post-fact monitoring including security documentation and limits monitoring. Business side continuously keeps in touch with customers to have updated information about the clients.<br><br>**Management:** Bank has been selective in taking exposure on good quality borrowers across all industry segments. Multiple factors in bank's lending structure provide additional comfort and support in mitigating credit risk. These include quality of eligible collateral, pre-disbursement safety measures, post disbursement monitoring, etc. Bank has a fairly diversified loan portfolio. For risk categorized as sovereign/ government risk, MCB's lending exposure is spread over multiple government owned or controlled organizations and departments which are engaged in a variety of tasks that ranges from different development related works to utility distribution and production.<br><br>Credit Risk Review ensures to minimize credit risk associated at account and portfolio level. During the year, the Bank continued with the policy to remain selective in disbursing its loan to low risk customers across all the industries & maintains a fairly diversified loan portfolio. Risk Review successfully managed to evaluate and approve increased number of loan requests, within required turnaround time, both for domestic and international operations. An in-house request tracking & turnaround time monitoring software ensures tracking of proposals and monitoring of turnaround-time of credit proposals routed through Risk Management Group. Bank's implemented Loan Origination System (LOS) for end to end automation of credit approval process facilitated effective management of Bank's internal policies & controls as well as regulatory requirements. LOS has also contributed towards Bank's transition to paperless environment under the Green Banking initiative.<br><br>For risk categorized as sovereign/ government risk, the lending exposure is spread over multiple government owned or controlled organizations and departments which are engaged in a variety of tasks that range from different development related works to utility distribution and production. To manage adverse outcomes in terms of unfavorable scenarios, multiple control factors in the lending structure of the Bank provide additional comfort and support. Such controls range from quality of eligible collateral, pre-disbursement safety measures to post disbursement monitoring.	Financial

Risk type	Materiality Rating	Probability of Risk Occurrence	Strategy	Impacted CAPITAL
**Credit Risk**	High	Medium probability	Through a four eye principle for credit approval levels for corporate and retail banking, all such exposure related requests are approved with the formal consent of at least two authorized individuals including one from business side having credit approval authority and other from risk management side having credit review authority.<br><br>The MC& RC is the management platform for discussion and deliberation on key risk issues in the portfolio. Regular meetings of the committee are convened to oversee the risk exposures in the portfolio of the Bank.<br><br>In order to further enhance the credit risk analysis the Bank has in place a probability of default based Internal Credit Risk Rating (ICRR) system which is based on statistical modeling and validation in line with Basel principles. The ICRR is currently focused on corporate and commercial customer categories. Furthermore; another Internal Credit Risk Rating Model is also in place for rating of SE & ME and Agri Customers. A model for Facility Risk Rating has also been implemented which would reflect expected loss rate of a credit facility.	Financial
**Liquidity Risk**	High	Medium to Low probability	**Measurement:** MCB regularly performs Liquidity Risk Analysis and liquidity stress tests as part of its liquidity monitoring activities. The purpose of the liquidity risk assessments and stress tests is intended to ensure sufficient liquidity for the Bank under both idiosyncratic and systemic market stress conditions.<br><br>**Monitoring:** Liquidity positions are regularly monitored through established early warning Indicators and liquidity risk analysis. Liquidity Coverage Ratio and Net Stable Funding Ratios are monitored regularly.<br><br>**Management:** MCB's Liquidity Risk Management approach involves intraday liquidity management, managing funding sources and evaluation of structural imbalances in balance sheet structure.<br><br>The Bank's large and stable base of customer deposits, along with Bank's strong capital base, indicates strong liquidity position. Bank also has a substantial portfolio of marketable securities that can be realized in the event of liquidity stress. Bank's Liquidity Coverage Ratio (LCR) and Net Stable Funding Ratio (NSFR) are well over and above the regulatory requirement.<br><br>The Asset Liability Management Committee of the bank has the responsibility for the formulation of overall strategy and oversight of the Asset Liability Management (ALM) function. BOD has approved a comprehensive Liquidity Risk Policy (part of Global Risk Management Policy), which stipulates policies regarding maintenance of various ratios, funding preferences, and evaluation of Banks' liquidity under normal and stress scenarios. Underlying policies and procedures are reviewed and approved regularly at the senior management and BOD Levels including Global Risk Management Policy, Global Treasury Policy, Investment Policy and Liquidity Strategy.<br><br>Further, in line with SBP's directives, Bank has fully implemented BASEL III required liquidity standards and maintains liquidity ratios including LCR and NSFR with a considerable cushion over and above the regulatory requirement to mitigate any liquidity risk.	Financial

Risk type	Materiality Rating	Probability of Risk Occurrence	Strategy	Impacted CAPITAL
**Technological/ Information Security Risk**	High	Low probability	**Monitoring & Management:** Through technology risk monitoring process, bank tracks and evaluates the levels of technology and security risk as well as monitoring the risk itself. The findings produced by risk monitoring processes are used to create new risk mitigation and monitoring strategies considering the regulatory compliance in-line with the best practices.<br><br>The Bank has developed Information Technology Risk Assessment Framework which enables better management of technology risk properly. The Information Security Risk Division is headed by a Chief Information Security Officer (CISO) reporting to the Group Head Risk Management.	Financial, Intellectual
**Country Risk**	Low	Medium to low probability	**Measurement:** Bank's Country Risk exposure is assessed against bank's cross border trade and treasury activities.<br><br>**Monitoring & Management:** Monitoring of risk exposure is a regular activity. Country exposure limits both for trade and treasury exposures are in place, which broadly capture direct exposure on sovereigns and foreign domiciled counterparties.	Financial
**Operational Risk**	Medium	Medium to low probability	**Measurement:** A database covering losses, control breaches, near misses & KRIs is being maintained. Operational Effectiveness of controls is assessed using the Risk & Control Self-Assessment (RCSA) process.<br><br>**Monitoring:** Monthly/Quarterly updates on operational risk events are presented to senior management, MC&RC and the RM&PRC of the Board.<br><br>**Management:** The bank has an internal Operational Risk awareness program which is aimed at building capacity and inculcating risk culture in the staff through workshops and on-job awareness. Banks' capacity to capture & report operational risk events and KRIs is further enhanced by implementing more professionally developed Operational Risk Management Software.	Financial, Intellectual
**Regulatory Risk**	Medium	Medium probability	**Measurement:** Management of regulatory risk entails early identification and effective management of changes in legislative and regulatory requirements that may affect the Bank.<br><br>**Monitoring & Management:** The Bank reviews key regulatory developments in order to anticipate changes and their potential impact on its performance.<br><br>Bank endeavors to maintain healthy relationships with regulators and continued compliance with regulatory requirements.	Financial, Intellectual
**Reputation risk**	Medium	Medium probability	**Monitoring & Management:** Reputational risk is managed on an ongoing basis through a policy framework that details expected behavior of the business and employees. It guides us on the monitoring of employee behavior and specific client responses as well as to society in general. This includes precise and transparent reporting through our integrated annual report, annual financial statements and through other public statements. Our risk mitigation strategy includes:<br>• a centralized policy on media;<br>• an escalation process for complaints; and<br>• clear relationships with stakeholders	Financial, Intellectual, Social & Relationship Capital

Information about defaults in payment of any debts and reason thereof

There is no default by the Bank in payments of any debts during the year.

Inadequacy in the Capital structure and plans to address such inadequacy

The Bank is not presently facing any kind of inadequacy in capital structure.

Opportunities:

Source	Opportunity	Strategy to Materialize	Impacted CAPITAL
External	Building strategic national/international alliances to contribute towards China Pakistan Economic Corridor (CPEC) execution.	Re-aligning the business model through segmentation and expansion of branch network.	Financial, Manufactured, Human, Intellectual
Internal	Strong capital base and high Capital Adequacy Ratio provides the opportunity of exploring International avenues in emerging/developed markets to expand Bank's network.	Explore new markets after performing the feasibility studies.	Manufactured, Intellectual
Internal	Developing and launching new deposit products to align & strengthen the existing product menu and to capitalize on the growing branchless and mobile/ digital Banking opportunities.	Increase focus on digitalization and automation of process.<br><br>Introduce new products considering the needs of different segments of the population.	Financial, Manufactured, Human, Intellectual
Internal	Increasing Bank's advances portfolio with enhanced focus on agriculture, SME and other segments.	Widening the scope of branch network in potential / untapped areas.<br><br>Align product expertise with client domicile.<br><br>Streamline and simplify processes for quick disbursement of advances	Manufactured, Human, Intellectual
External	Facilitating non-resident Pakistanis to increase the flow of home remittances.	Entering into new contracts with foreign agents.<br><br>Placement of Bank representatives overseas and increase marketing activities.<br><br>Explore new markets to increase customer base.	Manufactured, Human, Intellectual
External	Adoption of emerging technologies in rendering of premier digital financial services to the customers and playing a positive role in expanding the domestic technology ecosystem.	Forging strategic partnerships with leading technological platforms and strengthening banking relationships with financially capable enterprises proposing competitive solutions.<br><br>Enhancing e-banking proposition for attracting and retaining the technically proficient "millennial" customer segment.	Manufactured, Human, Intellectual, Social & Relationship

Directors' Report

We are pleased to present, on behalf of the Board of Directors, the annual report of MCB Bank Limited (MCB) for the year ended December 31, 2020.

Economy Review

Global Economy:

The year in review was dominated by an unprecedented global health crisis, during which the COVID-19 pandemic battered the world's economy and forced the governments and the central banks across the globe to take drastic fiscal and monetary easing measures. In the United States, the Federal Reserve brought down its policy rate to 0-0.25%, reducing it by a cumulative 150 basis points to support the tempering economic momentum and inflation. Taking the lead from US Federal Reserve, major emerging market central banks slashed their interest rates in an effort to shore up their economies. On the fiscal front, the U.S government signed the largest federal stimulus package, worth USD 2.3 trillion, to help cope with the economic downturn inflicted by the pandemic.

In the UK, the Bank of England similarly reduced its interest rate to 0.1% from 0.75% and expanded its bond purchasing program to combat the impact of the coronavirus pandemic on the economy. On the Brexit front, UK and EU reached a landmark trade deal after 11 months of negotiations and just a week before a year-end deadline. Although the agreement still leaves critical details that need to be worked out, the latest events have eliminated most of the uncertainty surrounding the exit of UK from the European Union.

Meanwhile, the Greater Europe, in addition to its already existing quantitative easing program entailing 20 billion euros of net asset purchases per month, introduced its pandemic emergency purchase program (PEPP) to support the ailing economy.

While far from over, there was some respite, as towards the end of the year, the announcement of Coronavirus vaccines by Pfizer, Moderna, and AstraZeneca brought some hope and helped lift the market sentiment. All bets are now placed on the efficacy of the governments to administer the rollout of COVID-19 vaccines in 2021.

Domestic Economy:

Pakistan started the year on a positive note as the tough economic measures taken by the government since the commencement of its tenure in 2018 started bearing fruit. However, during the year 2020, just like the rest of the world, Pakistan's economy was hit hard by the COVID-19 pandemic which forced the government and the central bank to take drastic policy measures.

On the fiscal side, the government announced a relief package worth PKR 1.2 trillion, which led to an increase in the government expenditure. Consequently, the fiscal deficit which was initially projected to be 7.1% of GDP for FY20 clocked-in at 8.1%. However, the situation improved slightly during the second half of CY20 as the revenue growth picked up after the lifting of lockdown. During July-December 2020, Pakistan recorded a fiscal deficit of PKR 1,137 billion (2.5% of GDP).

Meanwhile, Headline Inflation (Base Year 2015-2016) eased after the first quarter of 2020 as the demand declined due to the introduction of COVID-19 lockdown by the government. Low base impact also came into play which further put downward pressure on the inflation reading. On average, NCPI clocked-in at 9.53% in 2020. To support the ailing economy and to stimulate growth, SBP decreased its policy rate from 13.25% to 7.00%, recording a cumulative decline of 625 basis points during the year.

On the external front, Pakistan was able to mitigate the pressure due to the inflows received from multilateral sources, including International Monetary Fund (IMF), World Bank (WB), Asian Development Bank (ADB) and Asian Infrastructure Investment Bank (AIIB), during the year. Another development that lent support to the economy under these unprecedented times was the debt relief initiative taken by the G-20 countries.

Moving on to the current account, Pakistan's exports took a larger hit as compared to the imports and declined by 11.57% during CY20 as compared to the last year. Imports declined by merely 6.48% despite a significant drop in international oil prices. Remittance during CY20 increased to USD 26 billion from USD 22 billion in CY19 (+17% on a YoY basis). Current account recorded a surplus during CY20 due to a significant jump in remittances.

As a result of improved current account balance, PKR depreciated moderately by 3.22% against the dollar during CY20 and closed the calendar year at 159.83 after touching a high of 168.44 in August, 2020. The KSE-100 index witnessed a V- shaped recovery, corrected itself from its low of 27,047 in March to end the year at 43,755.

Profit and Appropriation

The profit before and after taxation for the year ended December 31, 2020 together with appropriations is as under:

	Rs. in Million
**Profit before taxation**	**48,249**
Taxation	19,212
**Profit after taxation**	**29,037**
Un-appropriated profit brought forward	55,777
Other comprehensive income - net of tax	(342)
Surplus realized on disposal of revalued fixed assets - net of tax	23
Surplus realized on disposal of non-banking assets - net of tax	5
Transferred in respect of incremental depreciation from surplus on revaluation of fixed assets to un-appropriated profit - net of tax	89
	**55,552**
**Profit available for appropriation**	**84,589**
**Appropriations:**	
Statutory reserve	2,904
Final cash dividend at Rs. 5.0 per share - December 31, 2019	5,925
Interim cash dividend at Rs. 5.0 per share - March 31, 2020	5,925
**Total appropriations**	**14,754**
**Un-appropriated profit carried forward**	**69,835**
Earnings Per Share (Rs)	24.50

Dividends

The Board of Directors declared a final cash dividend of Rs. 15 per share for the year ended December 31, 2020, which is in addition to the Rs. 5 per share of interim dividend already paid to the shareholders for the quarter ended March 31, 2020, taking the dividend payout ratio to 81.63%. In compliance with SBP's instructions, Bank did not declared dividend for the second and third quarters. The effect of the recommendation is not reflected in the above appropriations.

COVID-19 Pandemic

Enhanced regulatory framework, increased competition from digital entrants and rising operating costs for the brick and mortar branch model had pushed the traditional banking architecture in Pakistan to look for avenues that harbor the potential for inducing wide-ranging resilience, efficiency and acceptability.

Emergence of COVID-19, during the year, further challenged the existing banking landscape and paved way for the industry to adopt emerging technologies in rendering digital financial services amidst responsibly enforced social distancing and containment measures. A swift and coordinated response from the monetary, fiscal and regulatory authorities diluted the impact of emanating downside risks and remained a key determinant in addressing the consequences of COVID-19 outbreak.

MCB undertook various timely measures, by invoking Business Continuity Plan, for the protection of its customers and employees; hence mitigating the risk of spreading COVID-19 outbreak and concurrently playing a pivotal role in supporting Governments' key pandemic responses for credit extension and provision of essential banking services to the general public.

Under the evolving operating and macro-economic scenario, Bank's sustained focus on developing a robust risk management platform, inculcating operational efficiencies, offering bespoke customer services and continuous enrichment of its technology based products laid the foundation for another year of significant financial growth.

Performance Review

With strong build up in core earnings, MCB's Profit After Tax (PAT) for the year ended December 31, 2020, posted a growth of 21% to reach Rs. 29.04 billion; translating into an Earning Per Share (EPS) of Rs. 24.5 against an EPS of Rs. 20.23 posted last year 2019.

Sizeable growth in current deposits, timely shift in maturity profiling of investments and favorable re-pricing lag enabled the Bank to increase its net interest income by 20% to Rs. 71.3 billion.

Fee income for the year 2020 was reported less by 3% over last year, primarily due to lower transaction volumes and business activities induced by the lockdowns. However, with their lifting, fee income is normalizing. Amidst evolving yield curve expectations, proactive duration management of the investments portfolio resulted in capital gains of Rs. 3.3 billion during the year under review. Hence, the total non-markup income posted growth of 9% to reach Rs. 18.14 billion.

On the operating expenses side (excluding pension fund reversal), despite sustained inflationary pressures, expansion in branch outreach and continued investment in technological infrastructure together with regular performance and merit adjustments for the Human Capital, the Bank's strategic focus of balancing short term tactical cost reductions with long term cost initiatives has assisted in containing growth in administrative expenses; in turn registering a decline of Rs. 129 million during the year 2020. Thus the cost to income ratio of the Bank significantly improved to 36.87% from 43.4% reported last year.

In anticipation that portfolios affected by the pandemic might require provisioning once SBP's relaxations and waivers expire in 2021, the management has exercised prudence and booked a general provision of Rs. 4.0 billion during the year under review, hence providing insulation and loss absorption capacity against any risks to asset quality.

On the financial position side, the total asset base of the Bank on an unconsolidated basis was reported at Rs. 1.76 trillion depicting an increase of 16% over December 2019. Analysis of the asset mix highlights that net investments increased by Rs. 267 billion (36%) whereas due to subdued domestic demand gross advances decreased by Rs. 26.5 billion (-5%) over December 2019. However, gross advances increased by Rs 19.9 billion in last quarter of 2020.

The Non-performing loan (NPLs) base of the Bank recorded an increase of Rs. 1.77 billion and was reported at Rs. 51.19 billion. The increase was primarily on account of subjective classification by the Bank on prudent basis. The Bank has not taken FSV benefit in calculation of specific provision and has increased its un-encumbered general provision reserve to Rs. 4.57 billion. The coverage and infection ratios of the Bank were reported at 98.87% and 9.97% respectively.

On the liabilities side, the deposit base of the Bank registered an unprecedented increase of Rs. 144.74 billion (+13%) over December 2019, with 15% growth contributed by current accounts, improving the current account mix to 38% and CASA ratio to 92.96%.

Return on Assets and Return on Equity was reported at 1.77% and 18.88% respectively, whereas book value per share was reported at Rs. 137.02.

While complying with the regulatory capital requirements, the Bank's total Capital Adequacy Ratio (CAR) is 20.98% against the requirement of 11.50% (including capital conservation buffer of 1.50% as reduced under the BPRD Circular Letter No. 12 of 2020). Quality of the capital is evident from Bank's Common Equity Tier-1 (CET1) to total risk weighted assets ratio which comes to 17.03% against the requirement of 6.00%. Bank's capitalization also resulted in a Leverage Ratio of 7.03% which is well above the regulatory limit of 3.0%. The Bank reported Liquidity Coverage Ratio (LCR) of 237.72% and Net Stable Funding Ratio (NSFR) of 174.86% against requirement of 100%.

MCB has been declared the Overall Most Outstanding Company in Pakistan - 2020 and Most Outstanding Company – Financials Sector in Pakistan - 2020 by Asiamoney, an associate of Euromoney. The Annual report of MCB Bank has also been adjudged 1st by ICAP/ ICMAP in the financial sector category. MCB has won this award 10 times in last 11 years with 8 consecutive wins.

Future Outlook

Despite the timely measures taken by the government to curtail the spread of the Novel Coronavirus and to support the economy, 2021 is expected to prove challenging for Pakistan. The main challenge that the government currently faces is to stimulate growth without significantly increasing the inflationary pressures in the economy.

Moreover, all eyes are currently focused on the re-initiation of the IMF Program which was stalled when the Covd-19 pandemic hit the country. The IMF Program bodes well for international confidence in Pakistan and adds to the government's capability to tap international markets. In addition, it also holds significance on account of the follow through impact it has on fiscal management, especially through the elimination of subsidies and inefficiencies. Hence, any positive outcome of the negotiations with IMF will lead to the betterment of the economy.

The banking sector outlook remains engulfed in multifarious challenges emanating from the current economic downturn amidst expectations of a modest 1.5% GDP growth in FY21, Fintech disruptions, escalating regulatory compliance, emerging cyber security threats, lowering margins and rising risks to asset quality given the challenging operating environment.

Amidst the challenges, MCB remains well poised to capitalize on its stable deposit based funding structure, ample liquidity buffers, wide branch outreach and a multi-pronged strategy governing continuous augmentation of Bank's robust risk management platform and adoption of cutting edge technologies for achieving broad based digitization and posting stable financial growth.

Risk Management Framework

The risk management framework has been separately disclosed in the Annual Report.

Credit Rating

The Bank enjoys highest local credit ratings of AAA / A1+ categories for long term and short term respectively, based on PACRA notification dated June 26, 2020.

Statement on Internal Control

The Board is pleased to endorse the statement made by management relating to Internal Control over Financial Reporting (ICFR) and overall internal controls. The Management's Statement on Internal Controls is included in the Annual Report.

Statement under Code of Corporate Governance and section 227 of Companies Act 2017:

The Board of Directors is committed to ensure that the requirements of Corporate Governance set by the Securities and Exchange Commission of Pakistan and requirements of Section 227 of Companies Act 2017 are fully met. The Bank has adopted good corporate governance practices and the Directors are pleased to report that:

- The financial statements, prepared by the management of the Bank, present a fair state of its affairs, result of its operations, cash flows and changes in equity.
- Proper books of account of the Bank have been maintained.
- Appropriate accounting policies have been consistently applied in preparation of financial statements. Accounting estimates are based on reasonable and prudent judgment.
- International Financial Reporting Standards, as applicable in Pakistan, have been followed in preparation of the financial statements and any departure there from has been adequately disclosed and explained in the Annual Accounts.
- There has been no material departure from the best practices of corporate governance.
- There are no significant doubts upon the Bank's ability to continue as a going concern.
- Profit amounting to Rs. 2.9 billion has been transferred to the Statutory Reserve for the year 2020.
- The System of Internal Control is sound in design and has been effectively implemented and monitored.
- Key operating and financial data of the last six years is presented in the relevant section of this Annual Report.
- Pattern of Shareholding, complying with the requirements prescribed by the code is annexed with this Annual Report.
- Statement of Compliance with Code of Corporate Governance is included in the Annual Report.
- Composition of the Board is given in the Statement of Compliance with the Code of Corporate Governance in the corporate governance section of this Annual Report.
- Names of the persons who, at any time during the year 2020, were directors of the Bank have been separately disclosed in the corporate governance section of this Annual Report.
- The Committees of Board of Directors along with their terms of reference/charter have been separately disclosed in the corporate governance section of this Annual Report.
- The number of Board and committees' meetings held during the year and attendance by each Director has been separately disclosed in the corporate governance section of this Annual Report.
- Details of directors' training programs are given in the statement of compliance with the code of corporate governance.
- The remuneration policy of non-executive directors, including independent directors, as approved by the Shareholders of the Bank is disclosed in the corporate governance section of this annual report.
- Detail of remuneration of Chairman, President/CEO and non-executive directors is disclosed in note 40 of the unconsolidated financial statements.
- The principal risks and uncertainties facing the Bank have been disclosed separately in this Annual Report.

Corporate Social Responsibility (CSR)

The Board acknowledges its rightful duty to operate as a highly socially responsible bank. The activities undertaken by the Bank with regard to CSR are disclosed in the Corporate Sustainability section of this annual report.

Auditors

The Auditors, M/s KPMG Taseer Hadi & Co. Chartered Accountants have completed their term of five years and are not eligible for reappointment as per the regulations. Therefore, upon recommendation of the Audit Committee, the Board recommends appointment of M/s A. F. Ferguson & Co., Chartered Accountants, as the statutory auditors of the Bank for the financial year 2021 in the forthcoming Annual General Meeting.

Appreciation and Acknowledgements

The Board of Directors of MCB Bank Limited would like to thank the Government of Pakistan, the State Bank of Pakistan, the Securities & Exchange Commission of Pakistan and other regulatory bodies for their continued support, all shareholders and customers of the Bank for their trust, and our employees for their continuous dedication and commitment.

For and on behalf of the Board of Directors,

Imran Maqbool

Imran Maqbool
President & CEO
MCB Bank Limited

Shahzad Hussain
Director
MCB Bank Limited

February 10, 2021

کریڈٹ ریٹنگ:

بینک، پاکستان کریڈٹ ریٹنگ ایجنسی (PACRA) کے نوٹیفیکیشن مورخہ 26 جون 2020ء کے مطابق بالترتیب ٹرپل اے (AAA) اور (+A1) اے ون پلس کی داخلی طور پر بلندترین طویل اور قلیل مدتی کریڈٹ ریٹنگ سے مستفید ہے۔

انٹرنل کنٹرول اسٹیٹمنٹ

مینجمنٹ کی جانب سے فنانشل رپورٹنگ پر اندرونی انضباط (ICFR) اور مجموعی اندرونی انضباط (انٹرنل کنٹرول) سے متعلقہ بیان کی، بورڈ بخوشی توثیق کرتا ہے۔ مینجمنٹ کا اندرونی انضباط سے متعلق بیان سالانہ رپورٹ میں شامل ہے۔

کوڈ آف کارپوریٹ گورننس اور کمپنیز ایکٹ 2017 کے سیکشن 227 کے تحت بیانیہ

بورڈ آف ڈائریکٹرز اس امر کو یقینی بنانے کے لئے پرعزم ہیں کہ سیکیورٹی اینڈ ایکسچینج کمیشن آف پاکستان کی جانب سے معین کردہ کارپوریٹ گورنس کے تمام معیار اور شرائط اور کمپنیز ایکٹ 2017 کے سیکشن 227 کے تمام مطلوبات کی مکمل پاسداری ہو۔ بینک نے کارپوریٹ گورنس کے بہترین معیار کو اختیار کیا ہے اور ڈائریکٹرز بمسرت رپورٹ کرتے ہیں کہ:

- بینک کی مینجمنٹ کی طرف سے تیار کردہ فنانشل اسٹیٹمنٹس اسکے معاملات اور کارکردگی، صورتحال، نقدی کے بہاؤ (کیش فلوز) اور ایکویٹی میں تبدیلیوں کا واضح اظہار کرتی ہیں۔
- بینک کے اکاؤنٹس کی درست کتابیں تیار کی گئی ہیں۔
- فنانشل اسٹیٹمنٹس کی تیاری میں اکاؤنٹنگ کی مخصوص پالیسیوں کا یکساں اطلاق کیا گیا ہے۔ اکاؤنٹنگ کے تخمینے موزوں اور محتاط اندازوں پر مبنی ہیں۔
- فنانشل اسٹیٹمنٹس کی تیاری میں پاکستان میں قابل اطلاق انٹرنیشنل اکاؤنٹنگ اسٹینڈرڈز کو مدنظر رکھا گیا ہے اور اس سے کسی بھی انحراف کو وضاحت کے ساتھ سالانہ اکاؤنٹس میں بیان کیا گیا ہے۔
- کارپوریٹ گورنس کے بہترین معیار اور ضابطہ عمل سے کوئی قابل ذکر روگردانی نہیں کی گئی ہے۔
- بطور ادارہ بینک کے کام جاری رکھنے کی اہلیت میں کسی شک وشبہ کی گنجائش نہیں ہے۔
- سال 2020ء کے دوران 2.9 بلین روپے کا منافع قانونی ریزرو میں منتقل کیا گیا ہے۔
- اندرونی انضباط کا نظام مضبوط خدوخال پر استوار کیا گیا ہے اور نہایت مؤثر انداز میں نافذ اور جانچا جاتا ہے۔
- چھ سال کا فنانشل مواد (ڈیٹا) اور اہم کارکردگی کے عوامل کو سالانہ رپورٹ کے متعلقہ سیکشن میں پیش کیا گیا ہے۔
- قانون میں مجوزہ شرائط کی تعمیل میں شیئر ہولڈنگ (حصص کی ملکیت) کی ترتیب / نمونہ کو سالانہ رپورٹ سے منسلک کیا گیا ہے۔
- کوڈ آف کارپوریٹ گورنس کی تعمیل (کمپلائینس) کا بیان سالانہ رپورٹ میں شامل ہے۔
- بورڈ کی تشکیل کو کوڈ آف کارپوریٹ گورنس کے تعمیلات کے بیانیہ میں سالانہ رپورٹ کے کارپوریٹ گورنس کے سیکشن میں بیان کیا گیا ہے۔
- سال 2020 کے دوران کسی بھی وقت بینک کے ڈائریکٹرز رہنے والے اشخاص کے نام سالانہ رپورٹ کے کارپوریٹ گورنس کے سیکشن میں الگ سے بیان کئے گئے ہیں۔
- بورڈ آف ڈائریکٹرز کی کمیٹیاں اور انکے ضابطہ کار / چارٹر کو سالانہ رپورٹ کے کارپوریٹ گورنس کے سیکشن میں الگ سے بیان کیا گیا ہے۔
- سال کے دوران بورڈ اور انکی کمیٹیوں کے اجلاسوں کی تعداد اور اس میں ہر ڈائریکٹر کی شرکت کی تفصیل اس سالانہ رپورٹ کے کارپوریٹ گورنس کے سیکشن میں الگ سے واضح کی گئی ہے۔
- ڈائریکٹرز کے تربیتی پروگرام کی تفصیل کوڈ آف کارپوریٹ گورنس کی تعمیل کے بیان میں دی گئی ہے۔
- غیر انتظامی ڈائیریکٹرز بشمول آزاد ڈائریکٹرز کے لیے، بینک کے حصص یافتگان کی جانب سے منظور شدہ، مشاہرے کی پالیسی اس سالانہ رپورٹ کے کارپوریٹ گورنس کے سیکشن میں درج کیا گیا ہے۔
- چیرمین، پریذیڈنٹ / سی ای او اور غیر انتظامی ڈائیریکٹرز کو ادا کیے گئے مشاہرہ کی تفصیل غیر مجموعی فنانشل اسٹیٹمنٹس کے نوٹ نمبر 40 میں درج کی گئی ہیں۔
- بینک کو درپیش بنیادی عوامل اور غیر یقینی کی صورتحال کو اس سالانہ رپورٹ میں الگ سے بیان کیا گیا ہے۔

کارپوریٹ معاشرتی ذمہ داریاں سی ایس آر (CSR):

بورڈ معاشرتی طور پر انتہائی ذمہ دار بینک کی حیثیت سے اپنے فرائض پر عملدرامد کا مکمل ادراک رکھتا ہے۔ سی ایس آر (CSR) کے حوالے سے بینک کے کیے گئے تمام اقدامات کو اس سالانہ رپورٹ میں موجود کارپوریٹ سسٹین ایبلٹی (برداشت کی قوت) رپورٹ میں واضح کیا گیا ہے۔

آڈیٹرز:

بینک کے آڈیٹرز میسرز کے پی ایم جی (M/s KPMG) تاثیر ہادی اینڈ کمپنی چارٹرڈ اکاؤنٹنٹس، نے اپنی مدت کے 5 سال مکمل کر لیے ہیں اور ضابطوں کے مطابق دوبارہ تقرری کی اہلیت نہیں رکھتے ہیں۔ لہذا، آڈٹ کمیٹی کی تجویز پر بورڈ آنے والے عام سالانہ اجلاس میں میسرز اے ایف فرگوسن اینڈ کمپنی چارٹرڈ اکاؤنٹنٹس کی مالی سال 2021 کے لئے بینک کے قانونی آڈیٹرز کی تقرری کی سفارش کرتا ہے۔

تسلیم وتحسین:

ایم سی بی بینک کے بورڈ آف ڈائریکٹرز حکومت پاکستان، اسٹیٹ بینک آف پاکستان، سکیورٹی اینڈ ایکسچینج کمیشن آف پاکستان اور دیگر انضباطی اداروں کی جاری معاونت، بینک کے شیئر ہولڈرز اور صارفین کا انکے اعتماد اور اپنے ملازمین کا انکے خلوص اور عزم پر شکریہ ادا کرتا ہے۔

منجانب وبرائے بورڈ آف ڈائریکٹرز

**عمران مقبول**	**شہزاد حسین**
پریزیڈنٹ اور سی ای او۔	ڈائریکٹر۔ ایم سی بی بینک لمیٹڈ
ایم سی بی بینک لمیٹڈ،	

10 فروری 2021ء

افرادی وسائل کی میرٹ اور کارکردگی کی بناء پر باقاعدہ مطابقت کے باوجود، بینک کی جانب سے قلیل مدت میں اخراجات میں تکنیکی کمی اور طویل مدت میں لاگت کو محدود رکھنے کے اقدامات میں توازن کے قیام پر کلیدی توجہ سے انتظامی اخراجات پر قابو رکھنے میں مدد ملی۔ جس کے باعث سال 2020ء کے دوران اس مد میں 129 ملین روپے کی کمی درج کی گئی۔ چنانچہ لاگت اور آمدنی کا تناسب گزشتہ سال کی 43.4 فیصد کی شرح کی نسبت نمایاں بہتری کے ساتھ 36.87 فیصد پر رپورٹ کیا گیا۔

اس توقع پر کہ سال 2021ء میں اسٹیٹ بینک آف پاکستان کی جانب سے دی گئی سہولتوں اور چھوٹ کی مدت ختم ہوتے ہی عالمگیر وباء سے متاثر پورٹ فولیو کے حوالے سے پروویژننگ (Provisioning) کی ضرورت پڑ سکتی ہے، بینک کی مینجمنٹ نے دانشمندی کا ثبوت دیتے ہوئے، اس زیر تجزیہ سال کے دوران، 4 بلین روپے کی جنرل پروویژن کا اندراج کیا ہے۔ چنانچہ، اپنے اثاثہ جات کے معیار کو محفوظ بنانے اور اس کو کسی بھی درپیش خدشات کو برداشت کرنے کی صلاحیت میں اضافہ کیا گیا ہے۔

مالیاتی پوزیشن کی مد میں، بینک کے غیر مجموعی بنیاد پر شمار کیئے گئے کل اثاثہ جات دسمبر 2019ء کے حجم سے 16 فیصد کے اضافے کے ساتھ 1.76 ٹریلین روپے پر درج کیئے گئے۔ اثاثہ جات کی ترکیب کا جائزہ خالص سرمایہ کاری میں 267 بلین روپے (36%) تک کا اضافہ ظاہر کرتا ہے جبکہ کمزور مقامی طلب کے باعث خالص قرضہ جات میں دسمبر 2019ء سے 26.5 بلین روپے (-5%) کی کمی دیکھی گئی تاہم سال 2020ء کی آخری سہ ماہی میں کل قرضہ جات 19.9 بلین روپے تک بڑھ گئے۔

بینک کے غیر فعال قرضوں کے حجم میں 1.77 بلین روپے کے اضافے کے ساتھ انکو 51.19 بلین روپے پر رپورٹ کیا۔ اس اضافے کی بنیادی وجہ بینک کی جانب سے دانشمندانہ بنیاد پر کی گئی موضوعی درجہ بندی (subjective classification) تھی۔ بینک نے مخصوص (سپیسفک) پروویژن کا اندراج کرتے وقت جبری فروخت کے فوائد کا شمار نہیں کیا اور اپنے غیر تصرف شدہ جنرل پروویژن کے ریزرو (ذخائر) میں 4.57 بلین روپے کا اضافہ کیا ہے۔ بینک کی کوریج اور انفکیشن کی شرحیں بالترتیب 98.97 فیصد اور 9.97 فیصد پر رپورٹ ہوئیں۔

واجبات کے حوالے سے، بینک کے ڈیپازٹس میں دسمبر 2019 کی نسبت 144.74 بلین روپے (13%) کی بے مثال ترقی دیکھنے میں آئی جس میں کرنٹ ڈیپازٹس میں 15 فیصد کی نمو شامل رہی اور یوں کرنٹ اکاؤنٹ کی ترکیب 38 فیصد اور کاسا (CASA) کی شرح 92.96 فیصد پر رہی۔

اثاثہ جات اور ایکویٹی کی آمدنی کی شرحیں بالترتیب 1.77 فیصد اور 18.88 فیصد پر رپورٹ ہوئیں جبکہ بک ویلیو فی شیئر 137.02 روپے پر درج کی گئی۔

سرمائے کے ضوابطی مطلوبات کی تعمیل کے ساتھ بینک کے کل سرمائے کی معقولیت کی شرح، 11.50 فیصد کی مطلوبہ سطح کی نسبت 20.98 فیصد رہی (بشمول بی پی آر ڈی کے سرکلر لیٹر نمبر 12 برائے سال 2020ء کے تحت محدود کیے گئے 1.50 فیصد کے کیپٹل کنزرویشن بفر کے)۔ بینک کے سرمائے کا معیار، بینک کی کامن ایکویٹی ٹیئر - I (CET-I) کی کل رسک ویٹڈ (Risk Weighted) اثاثہ جات کے تناسب سے بھی ظاہر ہوتا ہے جو کہ اپنی 6 فیصد کی مطلوبہ شرح کی نسبت 17.03 فیصد پر درج ہوئی۔ بینک کی کیپٹلائزیشن کے نتیجے میں لیوریج (LEVERAGE) کا تناسب بھی اپنی 3.0 فیصد کی ضوابطی حد سے خاصی بلندی کے ساتھ 7.03 فیصد پر درج ہوا۔ بینک نے لیکوڈیٹی کوریج کے تناسب کو 237.72 فیصد اور خالص سٹیبل (Net Stable) فنڈنگ کے تناسب کو 100 فیصد کی مطلوبہ شرح کے مقابلہ میں 174.86 فیصد پر رپورٹ کیا۔

ایشیامنی، جو کہ یورومنی کا ذیلی ادارہ ہے، کی جانب سے ایم سی بی کو پاکستان کی اوور آل موسٹ آؤٹ سٹینڈنگ کمپنی برائے 2020ء اور پاکستان کے مالیاتی شعبہ کی موسٹ آؤٹ سٹینڈنگ کمپنی برائے سال 2020ء کے ایوارڈ سے نوازا ہے۔ ایم سی بی بینک کی سالانہ رپورٹ کو آئی سی اے پی / آئی سی ایم اے پی (ICAP / ICMAP) نے پہلی پوزیشن پر تعینات کیا۔ ایم سی بی نے گزشتہ 11 سال میں یہ اعزاز 10 مرتبہ جیتا ہے جس میں 08 مسلسل سال شامل ہیں۔

مستقبل کی پیش بینی

ناول کرونا وائرس کے پھیلاؤ کو روکنے اور معیشت کو مدد فراہم کرنے کے لیے حکومت کی جانب سے اٹھائے گئے بروقت اقدامات کے باوجود سال 2021ء پاکستان کے لیے ایک مشکل سال ثابت ہونے کی توقع ہے۔ حکومت کو اس وقت جو بڑی مشکل درپیش ہے وہ معیشت پر افراط زر کے دباؤ میں نمایاں اضافے کے بغیر اپنی نمو کو متحرک کرنا ہے۔

مزید براں، فی الوقت تمام نگاہیں آئی ایم ایف کے پروگرام، جو کووڈ-19 کی عالمگیر وباء کے باعث جمود کا شکار ہے، کی دوبارہ بحالی کی طرف مبذول ہیں۔ آئی ایم ایف کا پروگرام، پاکستان کے لیے بین الاقوامی اعتماد کی یقین دہانی اور بین الاقوامی مارکیٹوں سے رجوع کرنے کی حکومتی صلاحیتوں کو بھی بڑھاتا ہے۔ اس کے علاوہ، یہ پروگرام سب سڈیز اور نااہلی کے خاتمے کے ذریعے مالیاتی انتظام پر کڑی نگاہ رکھنے کے اعتبار سے بھی اپنی نمایاں اہمیت کا حامل ہے۔ لہذا، آئی ایم ایف کے ساتھ مذاکرات میں کوئی بھی مثبت پیش رفت معیشت کے لیے بہتری کا باعث بن سکتی ہے۔

بینکنگ کا شعبہ موجودہ معاشی انحطاط، جو کہ مالی سال 2021ء میں جی ڈی پی کی محض 1.5 فیصد کی نمو کی توقع میں گھرا ہوا ہے، فن ٹیک (Fin Tech) رکاوٹیں، بڑھتی ہوئی ضوابطی تعمیلات، ابھرتے سائبر سیکیورٹی خطرات، کم ہوتے مارجنز اور اثاثہ جات کے معیار کو موجودہ کاروباری ماحول کے بڑھتے خدشات سے نبرد آزما ہے۔

ان خدشات کے درمیان، ایم سی بی اپنے ڈیپازٹ پر منحصر فنڈنگ کے مستحکم ڈھانچے، کشادہ لیکویڈیٹی بفرز، برانچوں کی وسیع رسائی اور کثیر الجہت حکمت عملی پر اپنی توجہ جاری رکھے ہوئے ہے جو کہ مضبوط رسک مینجمنٹ کے پلیٹ فارم اور جدید ترین ٹیکنالوجی کو اختیار کرنے کے ذریعے بینک کے ڈیجیٹلائزیشن اور ایک پائیدار مالیاتی نمو کے وسیع البنیاد ہدف کے حصول کے لیے جوشاں ہے۔

رسک مینجمنٹ فریم ورک (خدشات کے تدارک کا انتظام)

رسک مینجمنٹ فریم ورک کو سالانہ رپورٹ میں الگ واضح کیا گیا ہے۔

منافع اور تخصیص:

31 دسمبر 2020ء کو اختتام پذیر سال میں منافع قبل و بعد از ٹیکس اور تخصیص، مندرجہ ذیل ہے:

	ملین روپے
منافع قبل از ٹیکس	48,249
ٹیکس	19,212
منافع بعد از ٹیکس	29,037
افتتاحی غیر تخصیص شدہ منافع	55,777
دیگر جامع (کمپریہینسو) آمدنی خالص از ٹیکس	(342)
پائیدار اثاثوں کے سرپلس پر فروخت سے حاصل آمدن (خالص از ٹیکس)	23
غیر بینکاری اثاثوں کے سرپلس پر فروخت سے حاصل آمدن (خالص از ٹیکس)	5
پائیدار اثاثوں کی تخمینہ نو سے اضافی آمدنی کی غیر تخصیص شدہ منافع انکریمنٹل ڈیپریسی ایشن (قدر میں کمی) کی مد میں منتقلی	89
	**55,552**
**تخصیص کے لیے دستیاب منافع**	**84,589**
**تخصیص:**	
قانونی ریزرو	2,904
حتمی کیش ڈیویڈنڈ 5 روپے فی حصص 31 دسمبر 2019ء	5,925
عبوری کیش ڈیویڈنڈ 5 روپے فی حصص 31 مارچ 2020ء	5,925
کل تخصیص	**14,754**
اختتامی غیر تخصیص شدہ منافع	**69,835**
فی حصص آمدنی۔ (روپے)	24.50

ڈیویڈنڈز

بورڈ آف ڈائریکٹرز نے 31 دسمبر 2020ء کو اختتام پذیر سال کیلئے 15 روپے فی حصص کے حتمی کیش ڈیویڈنڈ کا اعلان کیا ہے جو کہ حصص یافتگان کو 31 مارچ 2020ء کو اختتام پذیر سہ ماہی کے لیے 5 روپے فی حصص، کے پہلے سے اداشدہ عبوری ڈیویڈنڈز کے علاوہ ہے اور اس طرح ڈیویڈنڈ ادا کرنے کی شرح 81.63 فیصد پر ریکارڈ ہوئی ہے۔ اس اعلان کے اثرات کو مندرجہ بالا تخصیص میں شمار نہیں کیا گیا ہے۔ اسٹیٹ بینک آف پاکستان کی ہدایات کی روشنی میں بینک نے دوسری اور تیسری سہ ماہی کے لیے ڈیویڈنڈ کا اعلان نہیں کیا ہے۔

کووڈ۔19 ایک عالمگیر وباء

بڑھتے انضباطی فریم ورک، ڈیجیٹل دور میں داخل ہوتے اداروں کے باعث مسابقتی ماحول میں اضافہ اور برانچوں کی کاروباری لاگت میں بڑھوتی نے پاکستان میں روایتی بینکاری کے اسلوب کو ایسے عوامل کی تلاش پر مائل کیا ہے جہاں وسیع البنیاد لچکداری، قابلیت اور پذیرائی کا موجب بنتے ہوئے اس میں موجود مواقع سے مستفید ہوا جاسکے۔

سال کے دوران، کووڈ - 19 کے ظہور نے موجودہ بینکنگ کی صورتحال میں مزید مشکلات پیدا کر دیں جس کے باعث اس صنعت کو سماجی فاصلوں اور دیگر حفاظتی انتظامات کے ذمہ دارانہ نافذ کرنے کے ساتھ ساتھ، اپنی ڈیجیٹل مالیاتی خدمات پیش کرتے ہوئے نئی ٹیکنالوجی اور جدت کو اختیار کرنا پڑا۔ مالیاتی، انضباطی اور اصلاحاتی اتھارٹیوں کی جانب سے بروقت اور ہم آہنگ ردعمل نے تنزلی کے خدشات سے ابھرنے والے اثرات کو کافی حد تک زائل کیا اور کووڈ - 19 کے پھیلاؤ سے پیدا ہونے والے مضمرات کو دور کرنے میں اپنے اہم کردار کو برقرار رکھا۔

ایم سی بی نے اپنے بزنس کونٹینیوٹی پلان (Bussiness Continuity Plan) کا اجراء کر کے بہت سے بروقت اقدامات کا آغاز کیا تا کہ اپنے صارفین اور ملازمین کے تحفظ کو یقینی بناتے ہوئے کووڈ - 19 کے پھیلاؤ سے جڑے خدشات کا تدارک کر سکے اور بعد میں عالمگیر وباء کے ردعمل کے طور پر، حکومت کی جانب سے قرضوں کی وسعت اور عوام الناس کو بینکنگ کی ضروری خدمات کی فراہمی کے نمایاں جوابی اقدامات میں تعاون کا اہم کردار بھی نبھایا۔

تبدیل ہوتی کاروباری اور کلاں معاشی صورتحال کے تناظر میں بینک نے اپنی توجہ ایک مضبوط رسک مینجمنٹ پلیٹ فارم، کاروباری قابلیتوں کی تاکید، صارفین کو انکی خواہش کے مطابق خدمات کی فراہمی اور ٹیکنالوجی سے مزین اپنی مصنوعات میں جاری افزودگی جیسے معاملات میں پیش رفت پر مرکوز رکھی جو مزید ایک سال کے لیے نمایاں معاشی نمو کی مضبوط بنیاد کا باعث بنی۔

کارکردگی کا جائزہ

اپنی خالص آمدنی میں مضبوط اضافے کے ساتھ ایم سی بی کا منافع بعداز ٹیکس، 31 دسمبر 2020 کو اختتام پذیر مدت کے لیے 21 فیصد کی نمو کے اندراج کے ساتھ 29.04 بلین روپے پر جا پہنچا۔ اور اس طرح گزشتہ سال کی 20.23 روپے فی شیئر کے مقابلہ میں 24.5 روپے فی شیئر کی آمدنی کو درج کیا گیا۔

کرنٹ ڈپازٹس میں نمایاں بڑھوتی، اپنی سرمایہ کاری کے میچورٹی پروفائلنگ کی بروقت منتقلی اور اسکی قیمتوں کے دوبارہ تخمینے کے فوائد کے باعث بینک کی خالص انٹرسٹ آمدنی 20 فیصد کے اضافے کے ساتھ 71.3 بلین روپے پر ریکارڈ کی گئی۔

سال 2020ء کے لیے بینک کی فیس کی آمدنی، لاک ڈاؤنز کی بدولت محدود کاروباری سرگرمیوں اور کم لین دین کی بنیادی وجہ سے گزشتہ سال کی نسبت 3 فیصد تک کم ہوئی۔ تاہم، اس لاک ڈاؤن کے خاتمہ کے بعد فیس آمدنی اپنے معمول کی طرف بڑھ رہی ہے۔ اس ارتقائی خط آمدنی کی توقعات کے درمیان، اپنی سرمایہ کاری کے پورٹ فولیو کی مدت کے پیش قدم انتظام کے نتیجے میں، اس زیر تجزیہ سال کے دوران بینک نے 3.3 بلین روپے کا کیپٹل گین حاصل کیا ہے۔ چنانچہ، کل غیر مارک اپ آمدنی 9 فیصد کی نمو درج کرتے ہوئے 18.14 بلین پر جا پہنچی۔

کاروباری اخراجات کے حوالے سے (ماسوائے پینشن فنڈ کی کٹوتی کے)، افراط زر کے دباؤ، برانچوں کی تعداد میں مزید اضافہ اور ٹیکنالوجی کے ڈھانچے میں مسلسل سرمایہ کاری اور اس کے ساتھ ساتھ اپنے

ڈائریکٹرز رپورٹ:

بورڈ آف ڈائریکٹرز کی جانب سے ہم ایم سی بی بینک لمیٹڈ کی سالانہ رپورٹ، برائے سال مختتمہ 31 دسمبر 2020ء کو پیش کرتے ہوئے نہایت مسرت محسوس کرتے ہیں۔

معاشی جائزہ:

عالمی معیشت

زیرِ تجزیہ سال میں عالمی صحت عامہ پر ایک فقید المثال بحران چھایا رہا۔ جس کے دوران، کووڈ - 19 کی عالمگیر وباء نے پوری دنیا کی معیشت کو جھنجھوڑ کے رکھ دیا اور دنیا بھر میں حکومتوں اور انکے متعلقہ مرکزی بینکوں کو مالیاتی اور انضباطی آسانیوں کے فوری اقدامات اٹھانے پر مجبور کر دیا۔ امریکہ میں فیڈرل ریزرو نے کمزور ہوتی معاشی تحریک اور افراط زر میں تعاون کے لیے، پالیسی ریٹ کو 0 تا 0.25 فیصد تک کم کر دیا اور یوں مجموعی طور پر 150 بی پی ایس کی کمی درج کی۔ امریکی فیڈرل ریزور کے زیر اثر تمام ابھرتی منڈیوں کے مرکزی بینکوں نے اپنی معیشتوں میں بہتری اور ٹھہراؤ کے لیے انٹرسٹ ریٹ میں کمی کی۔ امریکی حکومت نے عالمگیر وباء سے پیدا ہونے والی معاشی ابتری میں مدد فراہم کرنے کی غرض سے 2.3 ٹریلین امریکی ڈالرز کی وفاقی امداد، کے بڑے پیکج، پر دستخط کیئے ہیں۔

برطانیہ میں، بینک آف انگلینڈ نے، اسی مماثلت میں، انٹرسٹ ریٹ کو 0.75 فیصد سے کم کر کے 0.10 فیصد تک مقرر کر دیا اور معیشت پر کورونا وائرس کی عالمگیر وباء کے اثرات سے مقابلہ کرنے کے لیے اپنے بانڈز کی خریداری کے پروگرام کو وسیع کیا۔ بریگزٹ (Brexit) کے محاذ پر، برطانیہ اور یورپین یونین نے 11 ماہ کے طویل مذاکرات کے بعد، اس کی حتمی تاریخ سے محض ایک ہفتہ پہلے، ایک تاریخی معاہدہ کیا ہے۔ اگرچہ اس معاہدہ میں چند امور ابھی بھی حل طلب ہیں، تاہم، حالیہ واقعات نے برطانیہ کی یورپین یونین سے علیحدگی کے بارے میں غیر یقینی کی صورتحال کو کافی حد تک واضح کر دیا ہے۔ درین اثناء، یورپ نے 20 بلین یوروز کے خالص اثاثہ جات کی ماہانہ خریداری کے موجودہ مقداری آسانی کے پروگرام کے علاوہ اپنی بیمار معیشت کو توانا کرنے کے لیے پینڈامک ایمرجنسی پرچیز پروگرام (PEPP) (Pandemic Emergancy Purchase Program) کا آغاز بھی کیا ہے۔

گو ابھی یہ آزمائش خاتمہ سے دور ہے، تاہم سال کے اواخر میں فائزر (Pfizer) ، ماڈرینہ (Moderina) اور آسٹرازینکا (Astra Zeneca) کی جانب سے کرونا وائرس کی ویکسین کے اعلان سے کچھ امید اور سکھ کا سانس بھی ملا ہے جو مارکیٹ کے رجحان میں بہتری کا باعث بنا ہے۔ اب ساری نظریں سال 2021ء کے دوران کووڈ-19 کی ویکسین کی ترسیل اور فراہمی کے لیے حکومت کی انتظامی صلاحیتوں کی طرف لگی ہیں۔

مقامی معیشت

پاکستان نے سال کا آغاز مثبت رخ سے کیا جیسا کہ حکومت کی جانب سے، سال 2018ء سے اپنے دور کے آغاز سے ہی، اٹھائے گئے کڑے معاشی اقدامات کے ثمرات حاصل ہونا شروع ہو گئے۔ تاہم، سال 2020ء کے دوران، دیگر ممالک کی طرح، پاکستان کی معیشت بھی کووڈ-19 کی عالمگیر وباء سے شدید متاثر ہوئی اور حکومت اور مرکزی بینک کو فوری پالیسی اقدامات اٹھانے پر مجبور کر دیا۔

مالیاتی حوالے سے، حکومت نے 1.2 ٹریلین روپے کے ایک امدادی پیکیج کا اعلان کیا جس سے حکومت کے اخراجات میں اضافہ ہوا۔ چنانچہ مالیاتی خسارہ، جس کا ابتدائی تخمینہ جی ڈی پی کا 7.1 فیصد تھا، مزید بڑھ کر 8.1 فیصد پر درج ہوا۔ تاہم، سال 2020ء کی دوسری ششماہی کے دوران لاک ڈاؤن کے خاتمہ سے آمدنی میں ہونے والی نمو سے اس صورتحال میں قدرے بہتری پیدا ہوئی۔ جولائی تا دسمبر 2020ء کے دوران پاکستان کے مالی خسارے کا حجم 1,137 بلین روپے (جی ڈی پی کا 2.5 فیصد) رہا۔

دریں اثناء، افراط زر میں (بنیادی سال 2016 - 2015)، حکومت کی جانب سے کووڈ -19 کے لیے لگائے گئے لاک ڈاؤن کی وجہ سے طلب میں کمی کے نتیجے میں، سال 2020ء کی پہلی سہ ماہی میں کمی دیکھی گئی۔ کم بیس ایفیکٹ (Low Base Effect) بھی اپنا کردار ادا کرتے ہوئے افراط زر کی شرح پر کمی کے لیے دباؤ کا باعث بنا۔ اوسطاً، این سی پی آئی (NCPI) سال 2020ء میں 9.53 فیصد پر جا پہنچا۔ اپنی ناقص معیشت کی مدد اور شرح ترقی کو محرک رکھنے کے لیے اسٹیٹ بینک آف پاکستان نے پالیسی ریٹ کو 13.25 فیصد سے کم کرتے ہوئے 7.00 فیصد کی شرح پر مقرر کر دیا اور اس طرح سال کے دوران 625 بیسز پوائنٹس کی مجموعی کمی کا اندراج کیا۔

سال کے دوران، پاکستان نے مختلف ذرائع، جن میں آئی ایم ایف (IMF)، ورلڈ بینک (WB)، ایشین ڈیولپمنٹ بینک (ADB) اور ایشین انفراسٹرکچر انویسٹمنٹ بینک (AIB) شامل ہیں، سے وصول ہونے والی رقوم کی وجہ سے بیرونی محاذ پر دباؤ کو کامیابی سے برداشت کیا۔ ایک مزید پیش رفت جو کہ اس انوکھے اور صبر آزما دور میں مدد اور تعاون کا باعث بنی، وہ جی - 20 ممالک سے قرض کی ادائیگیوں میں سہولت بھی تھی۔

کرنٹ اکاؤنٹ کی طرف بڑھتے ہوئے، پاکستان کی برآمدات، درآمدات کے مقابلہ زیادہ متاثر ہوئیں جو کہ گزشتہ سال کے حجم کی نسبت سال 2020ء میں 11.57 فیصد تک کم ہو گئیں۔ تیل کی بین الاقوامی قیمتوں میں نمایاں کمی کے باوجود درآمدات میں محض 6.48 فیصد کی کمی واقع ہوئی۔ ترسیلات زر سال 2019ء کی 22 بلین امریکی ڈالرز سے بڑھ کر سال 2020ء کے دوران 26 بلین امریکی ڈالرز (سال بہ سال کی بنیاد پر۔ 17%) تک جا پہنچیں۔ کرنٹ اکاؤنٹ میں ترسیلات زر میں اس اضافے کی وجہ سے سال 2020ء میں سرپلس ریکارڈ کیا گیا۔

کرنٹ اکاؤنٹ بیلنس میں بہتری کے نتیجے میں سال 2020ء کے دوران، پاکستانی روپے میں امریکی ڈالر کے مقابلہ میں 3.22 فیصد کی معمولی فرسودگی ہوئی جو کہ اگست 2020ء میں 168.44 روپے فی ڈالر کی بلند سطح کو چھو کر سال کے اختتام تک 159.83 روپے فی ڈالر پر بند ہوا۔ کے ایس ای (KSE)۔100 انڈیکس میں عمودی شکل کی بحالی دیکھی گئی جو کہ مارچ میں 27,047 پوائنٹس کی اپنی کم ترین سطح کو خود سے درست کرتے ہوئے سال کے اختتام تک 43,755 پوائنٹس پر پہنچ گیا۔

Groups Review

Wholesale Banking Group

The Wholesale Banking Group (WBG) continued its legacy of providing a stellar performance by managing to achieve its goals despite a tumultuous year where systematic risks and changes abounded. The core businesses of the Group, including Corporate banking, Investment banking, and Transaction banking all contributed towards the Bank's bottom line by expanding the wallet share and bringing in New to Bank Customers.

During the year, several strategy papers were issued which included group limit settings for large corporates, limit review exercise, and Risk Asset Acceptance Criteria (RAAC). To manage the portfolio on a proactive basis, the Group continued to closely monitor the concentration of sectors and regions.

In the Bank’s Spirit, The Group managed to use its relationship and resources to actively Cross-sell various consumer products such as auto, home, personal loan to employees of Corporate Customers. The Group through actively exploring wallet share sizing, and being on the pulse of its relationships, secured cash management mandates for Corporate clients, and customer investment requirements in GOP securities.

The Group’s investment arm managed to close several transactions and as part SBP’s relief efforts helped facilitate the Bank’s customers to meet their financial obligation. MCB's Investment Banking received recognition from international institutions:

- **ABF Corporate & Investment Banking Awards 2020**
  - o Equity Deal of the Year - Pakistan for Maple Leaf Cement
  - o Syndicated Loan of the Year – Pakistan for Mobilink PKR 45 bln Syndication

- **The Asset Triple A Asia Infrastructure Awards 2020.**
  - o Telecom deal of the year in Pakistan (Mobilink PKR 45 bln Syndication)

- **The Asset Triple A Islamic Finance Awards 2020**
  - o Best Corporate Sukuk- Engro Polymer & Chemicals Limited 8.75 billion rupees OTC-listed Sukuk certificates (Lead Arranger)
  - o Best Acquisition Financing- AKT Sugar Mills 1.5 billion rupees syndicated Islamic term facility to acquire Gulf Sugar Mills (Lead arranger and adviser)
  - o Best Syndicated Loan- Pakistan Mobile Communications Limited 45 billion rupees syndicated term finance facility (Mandated lead adviser and arranger)

MCB Cash Management crossed the annual volume milestone of PKR 2.0 trillion in 2020 and remains one of the leading corporate cash management banks in Pakistan. A focus on the processing of Initial Public Offerings (IPO), Right issues, E-Dividends, and the digitization of domestic payments' modules contributed to the realization of this achievement. The combination of a vast branch network, state of the art technology platforms, efficient relationship management, came together to support growth in the Group, and meet the requirements of top Corporations across the country.

MCB Burqraftaar, the banks Home Remittance product remains amongst the top remittance payout banks in Pakistan. With the bank's extensive branch network and remittance partners scattered across the globe, we proudly contribute towards the national interest of Pakistan by promoting legal channels. Burqraftaar under its umbrella offers a suite of conventional and digital products for the ease and convenience of overseas Pakistanis and their loved ones back home.

In 2020, Strategic Acquisitions and Investments Division managed the divesture for MCB Financial Services Limited (MCBFSL), a wholly-owned subsidiary of MCB Bank Limited. The Division strives to explore internal and external options for the Bank’s strategic growth.

WBG team is geared up for further growth strategy in 2021, by continuing its focus on prudent book building.
In early 2021, WBG and International Banking have been merged into one group, Corporate Finance and International Banking (CFIBG) to create synergies and bring efficiency.

International Banking Group

MCB Bank has footprints in the International arena which includes Sri Lanka, Bahrain, and United Arab Emirates (UAE).

Sri Lanka Operations:

Bringing its regional experience & expertise, MCB Bank Limited, commenced its Sri Lanka operations in 1994. Starting off as a single branch, the bank focused on trade-related business. The bank has steadily grown over the years and currently holds the second-largest branch network among foreign banks in the country. With the development of a full suite of well-diversified product range, the bank now caters to the needs of segments of Retail, SME & Corporate clients. Today, deposits and advance portfolios are over LKR 20 Billion each with an overall asset base of over LKR 32 Billion.

Despite a much challenging operating environment and badly impacted economic performance of the country mainly due to the Covid-19 outbreak, MCB Sri Lanka

remained profitable for the year 2020. Also managed to secure its local rating [SL] A+ (Stable) by ICRA Lanka Limited, which indicates the better portfolio, risk, and liquidity management of the bank.

To ensure sustainability against the market risks, the bank continued to closely monitor its concentration of sectors and portfolios. During the year, several strategic decisions have been taken to effectively manage the lending portfolio while reducing the credit risk of the Balance Sheet. Further, The Bank has adhered to all necessary guidelines under the Covid-19 relief scheme announced by the Central Bank of Sri Lanka. Further, the bank has been able to improve its CASA and AD Ratio substantially during the year.

During the difficult market conditions for core banking business, the bank implemented several cost control initiatives which helped to restrict the total operating cost of the bank 29% below its annual budget for 2020.

UAE Operations:

MCB Bank commenced its operations in Dubai, UAE as a wholesale banking branch in 2015.

The franchise's strategy of diversifying its portfolio on both the liability and asset front has resulted in a dynamic portfolio with an ability to adjust as per market challenges. Focus on Financial Institutions and trade based short term liquid assets helped in achieving not only the bank's growth targets but also a portfolio with acceptable risk. The branch has been able to generate quality returns, by increasing its productivity.

Despite the economic backdrop, the Branch was able to maintain its deposits base in 2020, through diversification of the liability portfolio, while maintaining a CASA base of over 92%.

MCB UAE stood fast and firm in its commitment to expanding its outreach and in achieving its objectives while safeguarding the interest of our shareholders through prudent policy and by relying on the Bank’s core strengths.

Bahrain Operations:

MCB wholesale Bank Bahrain's key focus during the year was to align its books with current market demands. The branch worked on maintaining an adequate balance sheet size that was more responsive to the approved risk and return framework.

The focus of the franchise was to develop and implement several policies and procedures to cover ever-evolving regulations. MCB Bahrain also remained focused on AML/CFT and compliance areas. The branch's future strategy focuses on developing and mobilizing organic liquidity. Gap assessments with regards to internal policies and procedures were conducted and later approved for onward implementation.

Creating a safe environment for its customers and employees, MCB Bahrain maintained strong customer and employee safety protocols during the pandemic. Meetings through digital medium were implemented.

Retail Banking Group (RBG)

The year 2020 brought with it a unique set of opportunities and challenges. The environment became a test of our resolve, capabilities and commitment. Half of MCB Bank’s branches were closed for a better part of the first half of 2020 due to COVID-19. MCB Head Office, Circle and Regional teams worked at almost 50% capacity while Federal/Provincial Government and the regulators were issuing guidelines on a real time basis to better safeguard customers and to ensure continuity of operations in the banking sector. RBG branches and offices have made this possible during unprecedented times.

Team RBG remained focused on core revenue streams and enhancing cross selling volumes with efficient cost management. Through a smart mix of products, process initiatives, sales support programs, service management activities, robust governance & untiring efforts of our teams, “The Best Selling Machine” has enabled RBG to celebrate another victorious year. The addition of Relationship Managers in our branches & “Participation From All Initiative” to ensure focus on relationship management, core deposit mobilization & cross selling of bank’s products has improved overall customer experience.

RBG, being the biggest deposit and revenue contributor to the overall growth of MCB Bank, has always been instrumental in building “low-cost deposits” with CASA mix standing at 94%. The best part of this year has been the phenomenal and our highest ever Average Deposit Growth achieved in both Total Deposits as well as Current Deposits. Growing fee income from diverse products and branch operations has been instrumental in increasing profitability. Prudent month-on-month budget management, starting from a branch level, ensured revenue maximization and controls over expenses.

During 2020, in line with SBP’s guideline to facilitate customers during the COVID-19 pandemic, our credit team facilitated relaxations for existing lending clients and offered “Rozgaar Scheme” to offset the cash flow impact of the pandemic. Agri. Financing also witnessed a modest growth.

The year had been especially fruitful with the introduction of new products & initiatives. MCB Bank is proud to be one of the leading banks that offered the SBP mandated Roshan Digital Account, a digital banking solution for Non-Resident Pakistanis (NRPs) and Resident Pakistanis. The Roshan Digital proposition includes digital payments, savings and investments avenues. MCB Bank is the only bank offering 9 foreign currencies and has succeeded in attracting good volumes.

MCB One Current Account is another iconic product launched in 2020. The product caters to the needs of all customer segments and demographics.

Governance:

Strict implementation of policies and procedures has been embedded for a robust compliance and control culture to maximize operational efficiency and governance. Complete focus on Operation Excellence has helped us achieve higher sales, better quality service and enhanced levels of internal controls. In implementation of branch network optimization strategy, a number of low potential branches were merged, relocated or closed, while under the network expansion plan 21 new branches were opened during the year. MCB ATMs uptime remained 98.3% despite the challenges faced due to Covid-19 pandemic. Introduction of Pak Rupee term deposit rollover / renewal facility and FCY Term Deposits centralization has resulted in more business, efficient portfolio tracking and monitoring.

We are confident that our passion and dedication will continue to grow in the future. RBG intends to build further momentum with Roshan Digital to engage the maximum NRPs for account opening and investment. The Group also intends to focus on conversion of Home Remittances, soliciting NTBs, retention / deepening of existing deposits on-boarding Cash Management and Payroll & Collection Mandates in 2021. Dispersion of funds to the mass market in order to meet SBP's vision of providing subsidized low cost mortgages, construction finance and loans to youth (Kamyab Jawan) will also be done in a prudent manner.

Fight against COVID-19 & similar challenges:

COVID-19 was a major event of 2020 that impacted all aspects of Group operations. RBG lost a number of good colleagues and family members during the continuing pandemic. Their courage to resolutely perform their duties during these testing times and their contribution to our success shall always be remembered and cherished. Developments on the vaccine front and its gradual roll-out worldwide are likely to keep up the expectations of healthy recovery during 2021. However, the effectiveness of these measures is yet to be gauged.

Our continuing challenge remains maintaining and building upon the momentum we have created & maintained throughout the past few years. With over 1,400 branches and sub-branches spread across 56 regions and more than 8 million customers - the team, with its commitment, passion and robust execution plan, is geared to surpass expectations year-on-year through sustained performance to achieve even greater results.

Special Assets Management

The year 2020 has been an extremely challenging time for Pakistan's economy due to Covid-19 pandemic and variety of other reasons. However, despite all these challenges, SAM made continued progress on its strategic path and recorded cumulative recovery of Rs. 2.4 Billion by settling a large number of hardcore and protracted defaults. While achieving improved operational efficiency, SAM further strengthened its policy & procedural framework and built capabilities for future growth. Today, the diversity, creativity and expertise of SAM's team in management of NPLs are unique, which differentiate the Bank amongst its peers in this particular business segment.

While economy in the year 2021 is expecting growth viz. recital of the businesses, Non-Performing Loans (NPLs) of the banking sector have already flowed to Rs. 868 Billion as of September 30, 20 from Rs. 776 Billion at the end of the year 2019. Therefore, we believe that future still holds challenges for the banking industry in the context of remedial management of impaired advances. Nevertheless, SAM will continue to focus on its milestones to take full advantage of potential growth opportunities, arising out of revival of manufacturing & real estate sector and overall business environment, to mark 2021 another banner year to maintain its leadership position in the industry.

Consumer Banking Group

MCB Consumer Banking continues to win confidence and trust for its valued customers for financial solutions of their needs. Consumer products are designed in a way keeping in mind the convenience and hassle free financial solution to match their Lifestyle. MCB consumer banking showed a stable growth and also introduced new product variants to further enhance customer's confidence.

Wealth Management:

The Banca business has been on a steady upward growth trajectory over the past few years. Sales grew from Rs. 1.4 Billion to Rs. 3.15 Billion in the 5 year period from 2015-2019, a CAGR of 22%. Despite the impact of COVID in 2020, MCB still performed strongly and achieved Rs. 2.6 Billion in Sales, about 82% of the number achieved in 2019. This demonstrated the traction, strength and resilience of the business, as the overall industry numbers

were down by 30% relative to 2020. Relative profitability was even higher through a focus on cost management and timely renewal of policies, with profits from the business at 85% of the previous year.

With more favorable market conditions in the second half of the year, Investment Services posted impressive numbers in 2020 with total sales of Rs. 5.7 Billion in the year, a growth of 124% from 2019. In terms of revenue and profits, MCB's Investment Services business was the clear market leader with the highest numbers in the industry.

Consumer Lending:

The MCB consumer lending book size at the end of year 2020 was over PKR 28 Billion, serving over 134,000+ customers across all lending products.

During the year 2020, consumer lending underwent a series of challenges related to economic and operational fronts, mostly brought about by the Covid-19 pandemic. The business, however, got some boost in sales in the latter half of the year as KIBOR declined thereby reducing the cost of borrowing for the end-customers.

During this year, we launched an auto fleet loan product to cater to Small and Medium Enterprises. In line with SBP instructions we also started offering subsidized home loans under Government Markup Subsidy Scheme and also launched a home loan variant as well as a lien-based credit card solution for non-resident Pakistanis.

The business continued to form strategic alliances with OEMs to provide value-added service to our auto customers and also offered high end tactical and regular discounts campaigns to its esteemed credit card customers.

To assist our credit card customers better, we also introduced SMS banking and IVR self-service channel.

Capital Market

Beset by an unprecedented health crisis, 2020 was undeniably a challenging year for the market. Concerned about the devastating implications of this economic shock on an already struggling economy the market succumbed to panic selling. Market volatility increased beyond measure and at times stretched to levels triggering trade halts. A critical support was however found at 27,267 points where accommodative monetary stance and generous fiscal stimulus arrested the panic induced free fall. Efficacy of these measures was further amplified by a corresponding flattening of Covid-19 curve and subsequent lifting of lockdowns. This injected a fresh impulse in the market and set if off on the path to recovery. The rally was supported by improving high frequency indicators, stabilizing macro backdrop, announcement of construction amnesty, satisfactory vaccine trials, status quo in FATF review and visible progress towards circular debt resolution. Dampeners in the shape of stalled IMF program, exorbitant inflation outturns, political tensions and onslaught of second wave of Covid-19 kept the gains in check. Nonetheless, incredibly cheap valuations and relative attractiveness of equity as an asset class amid a low interest rate environment kept the market momentum up and running. Consequently, benchmark index closed the year in green posting a return of ~7.41% (4% in dollar terms). Following suit, Capital Markets Division took this adversity in stride and managed to outperform the benchmark by a staggering margin for the year while actively mitigating risk. Expecting the Bull Run to continue well into 2021, we express optimism on market outlook and Capital Market Division's future performance.

Treasury and FX Group

During the year 2020, Pakistan's economy was hit hard by the Covid-19 pandemic. MCB's Treasury & FX Group, however, efficiently navigated through these unprecedented circumstances. The Group surpassed its performance targets for the year and made a significant contribution to the Bank's markup and non-markup revenues.

The year under review was marked by high interest rate volatility with the policy rate coming down to 7.00% from 13.25% as the central bank tried to mitigate the impact of the coronavirus pandemic on the economy. MCB Treasury took advantage of this opportunity and booked capital gain on some portion of its bond portfolio. The yield curve, however, steepened as the year progressed, indicating the market's expectations of impending rate hikes. In such an environment, Treasury continued to decrease the Duration of its investment portfolio. Moreover, due to the outflow from SCRA accounts (commonly known as hot money), the FX market also witnessed significant USDPKR volatility during the first half of the calendar year. Once again, Treasury expertly managed its exposures. Hence, 2020 proved to be an outstanding year for MCB's FX Income. In addition, MCB Treasury & FX Group ensured that its clients were kept up-to-date with the quickly changing market dynamics. All in all, the interbank desks (both Money-Market and FX), despite the market volatility, maintained their status as top market-makers.

More than ever, the year 2020 was full of uncertainties that exposed businesses to various market risks. As an authorized Derivatives Dealer, our derivatives team educated suitable clients about the use of derivatives to manage their risks and offered a range of derivative products to suit their hedging needs.

During the year, MCB Treasury Research Desk ensured that the internal and the external stakeholders of the bank are catered for by assisting them in gauging the impact of the Covid-19 pandemic on the state of the economy. The Research Desk delivered timely projections of foreign exchange rates, interest rate, inflation and other macroeconomic variables to the Bank's clientele and kept the stakeholders informed about the latest developments on the Covid-19 front. Moreover, the Research Desk published Daily Economic Snapshot, and released bi-monthly MCB Purchasing Managers' Index (MCB PMI) to the Bank's clients to measure the strength of the manufacturing sector in the economy.

Information Technology Group

Information Technology has transformed the banking industry worldwide for the better and has provided us with the necessary tools to manage the challenges of an ever growing economy. The integration of technology has been the cornerstone of recent financial sector reforms across industry aimed at increasing the productivity and efficiency of financial operations, strengthening the banking sector to meet high consumer expectations and to secure a leadership position amongst peers. However, the most challenging part of the ever changing world of 'Technology' is to neutralize the impact of obsolescence and advancements at a strategic level without compromising availability, security and reliability of the 'Data and Information'.

In MCB Bank, the entire management and the board is committed to enrich Information Technology infrastructure at the enterprise level which is clearly evident from the investments the bank chose to make in the recent past. We are committed in our mission "To be a leading bank in the Information Technology sphere, with our focus on digital transformation to enhance customer experience". The multi-pronged approach comprises of but is not limited to one of the largest footprint of online branches with a centralized core and associated banking systems, strategic acceptance systems at both eCommerce and POS levels, robust Internet Banking and Mobile Banking platforms, revolutionary product and services offered through world class Digital Lounges, highly efficient & scalable Contact Centers, one of its kind state-of-the-art Enterprise Data network, established International footprint, proactive controls to combat money laundering and frauds, interoperability with other financial entities and Branchless Banking solutions to facilitate the customers irrespective of their location.

Information Technology Group (ITG) at MCB Bank is comprised of a team consisting of thorough professionals having a proven track record of project delivery and IT infrastructure management at an enterprise. The components of the organizational structure of ITG are included with IT Enterprise Infrastructure, IT Operations, IT Software Solutions, IT Support Services, IT Service Management, IT Information Security, IT Business Technology, IT Compliance and Internal Control, IT Business Continuity, IT Project Management, IT Procurement and IT Financial Services who are committed to servicing it's consumers both internally and externally 24/7. Recently ITG successfully streamlined existing operations across board and recorded the highest ever uptime of core banking along with affiliated applications throughout the year. Nevertheless, the availability of Data Centers and the back-end systems along with all necessary services were remarkably remained at five-nines. In addition to operational achievements by ITG, new benchmarks were achieved to comply with the highlighted recommendations by external and internal audit committees and the SBP.

ITG had also closely monitored Bank wide projects and had driven related technology functions with a motive to accomplish the assigned tasks within the agreed timelines. Close coordination and liaison with working groups and vendor relationship management are the key factors in above achievements.

Looking forward to 2021, IT Group is more committed towards:

- Dynamic Customer Experience with Cross-TouchPoints
- Revolutionizing payments & Driving Financial Inclusion
- Agile & Goal Oriented Product Service Road Map Delivery
- Payment Card Industry: Compliance: PCI DSS Certification
- Improving Information & Cyber Security ecosystem

Apart from above commitments, IT Group shall continue to empower the staff with the requisite trainings and job enrichment plans to impart motivation and job success factors.

Digital Banking

COVID-19 brought disruptions in the global economy revolutionizing the way businesses have been conducted for decades. The world saw a significant shift towards digital payments giving birth to a new era. Digitization is on course to render the traditional modes of payment redundant. At MCB Bank the core driving force has always been in curating a personalized customer experience that adds value to their life. MCB Banks Digital Banking business has been designed to cater to the needs of the customers to tackle the ongoing pandemic.

Debit Card

MCB Bank was the first bank to offer chip based debit cards and it has one of the largest debit cards portfolio. In 2020 MCB introduced Near Field Communication (NFC) enabled debit cards for its consumers. This new technological innovation is rapidly being adopted by the world as it allows individuals to “tap and go” limiting the interaction between individuals which has become a need for today’s society.

For MCB the Year 2020 was a year of mega transformational project, the bank introduced the latest secure chip and pin cards with contactless technology to their entire card base along with the migration to a new card management host.

MCB Debit Card offers a rich value proposition to its cardholders offering discounts on lifestyle products and services alongside with round the clock availability of ATMs and POS network. In 2020 the main focus was on introducing discounts on healthcare services and online payments to provide convenience to consumers during COVID 19. With an MCB Debit Card, one can forget the need to carry cash in the new era of a digitalized economy.

Mobile Banking & Internet Banking

With the proposition “banking anywhere, anytime” MCB Mobile has proven and positioned itself as one of the most successful and highly sought after mobile banking services within a short span of time. MCB provide customers an easy and secure way to transfer money, carry out balance inquiry, check mini-statements, buy top-ups, pay bills and much more with and comfort of their homes. The bank witnessed a substantial increase in Mobile Banking transactions during 2020. With a cumulative number of 500,000+ App downloads the business is geared up to explore new possibilities across different verticals.

MCB Internet Banking is a state-of-the-art online banking platform with a friendly user interface providing a host of functionalities/services. MCB facilitates its customers to manage and control their bank accounts; whenever and wherever they want. During 2020 with COVID-19 lockdown restrictions and initiatives by the regulator, MCB saw substantial growth in Internet banking registrations.

ATM

ATM network also witnessed steady progression in terms of reach, reliability and transactional volume. MCB’s network now stands at 1400+ ATMs one of the largest national ATM network spread across 500+ cities. MCB Bank has an ATM uptime rate of 98% one of the best across the industry, ensured by the presence of ATM monitoring teams working 24/7 with periodic system checks and timely hardware replacements.

Card Acquiring (POS)

MCB Bank has a strong Network of approximately 3,000+ POS terminals at key locations across Pakistan. Our Point of Sale (POS) network is fully compliant with EMV & UICC (international safety & quality standards) and accepts Visa, MasterCard, UPI, JCB and Paypak enabled cards from all over the world. The POS Acquiring business continues to grow due to deepening of merchant relationships, round the clock support, increased POS productivity and quality of deployments.

Internet Acquiring (MCB eGate)

MCB eGate is a world class 3D secure online payment gateway, formed as a result of our partnership with MasterCard International. MCB eGate equips online businesses to accept payments reliably and securely from both credit and debit cards and currently facilitates more than 600+ online merchants, directly and indirectly. The online card acquisition footprint has grown and continues to grow substantially worldwide and MCB Bank is playing an integral part in developing the e-commerce market in the country. Further, to improve online shopping and merchant experience for e-commerce, MCB eGate payment system has now been upgraded from Mastercard Internet Gateway Service (MiGS) to Mastercard Payment Gateway Services (MPGS) which is fast, reliable and supports multiple payment modes. It also comes with the range of other value added features including real time fraud prevention and supports Mobile Commerce.

MCB Lite

MCB’s branchless banking offers its Lite mobile wallet customers the ability handle their daily transactions and payments in an efficient and real time manner. In 2020 MCB Lite undertook substantial projects with the successful migration of Lite cards to the new Core Management System (CMS) offering chip and pin functionality. Additionally the bank also introduced Lite EMV compliant cards and instant account opening through biometric device. This year branchless banking also saw the addition of reputable national organizations on its funds/salary disbursement portfolio. MCB Lite is all set to explore new avenues while embracing the digital revolution in the banking arena.

Operations Group

In the backdrop of significant challenges arising from the rapid spread of COVID-19 pandemic, Operations Group has remained steadfast in preserving its objective of strengthening internal controls and improving operational processes through automation and centralization while ensuring strict regulatory compliance. Operations Group has been at the forefront to stand hand-in-hand with the Bank in adapting to the extraordinary circumstances through an unprecedented effort from each and every

member. The Group managed the impact of COVID-19 by making effective social distancing arrangements through splitting teams for ensuring minimum turbulence in service delivery both for internal and external customers.

Priority of the Group continued to be on revitalizing business growth and refining service efficiencies through process automation and digitization. Consequently, functional support was provided in various projects having bank-wide significance including Roshan Digital Accounts, Foreign Exchange Cases Approval Portal and automation & centralization of FCY Term Deposits. Similarly, Operations Group led projects for enhancing system capabilities for Treasury Derivative System and its Product Suite for our overseas outfits. Valuable enhancements were also made in RTGS STP System to support branches meet COVID-19 contingencies through Direct Cheque Deposit Facility, Bulk Payments processing, revamping of remote area clearing protocols and providing comprehensive exception handling mechanism for customer facilitation. In addition, various new Core Banking screens were developed through enhanced Third Party Module Utility for Cash Transactions and Export Advance Payments to allow digitization of manual transactions and automated generation of related reports. Data cleansing exercise also continues to move ahead in full swing and productive results have started to be seen.

Operations Group has provided critical support for successful launch of various Government of Pakistan led projects like Low Cost Housing Finance and Subsidized Housing Finance Products under the Naya Pakistan Housing Scheme, refinance scheme for salary & wages, temporary economic refinance facility and refinance scheme for renewable energy to cater to economic relief for corporate sector. Likewise, launch of other new products has also been facilitated including Auto Fleet Product variant and introduction of new enriched accounts related services at MCB Call Centre through deployment of upgraded Interactive Voice Response (IVR) self-service.

Operations Group has also remained watchful of cost expansion and strived to optimize cost management through the set-up of In-House MCB Print Shop for printing customer account statements and letters in a cost effective manner. There has also been automation in deduction of various service charges including that related to duplicate issuance of payment instruments by providing system based solution thereby minimizing leakage of income and reducing manual workload. Centralized Cheque Clearing protocols were also revisited to change the frequency of delivery for processed cheques wherein a huge amount has been saved.

Keeping its focus on promoting Green Banking, Operations Group has facilitated a number of in-house and external staff trainings virtually. The Business Continuity framework was upgraded for building capability of an effective response to disruptive events and ensuring continuity of critical functions in an event of disaster. Green Office certification was also achieved for an MCB's iconic building i.e. MCB Centre, Lahore and various awareness initiatives were launched for staff and customers on Green Banking. In addition, support was provided to back offices and branches in procurement of COVID-19 related protective gears and arranging fully functional alternate sites for pandemic affected areas.

Operations Group is optimistic about 2021 and believes that 2021 will be the year of transition and recovery. The Group will continue to prioritize its digitization and centralization drive and remain focused on its ongoing cost discipline and proactive engagements with other groups to ensure marked improvements in service delivery and enriched product offerings. Operations Group looks ahead to fully support the Bank in pursuing its business strategy and to contribute in every possible way in its vision of becoming the leading financial services provider.

Compliance and Controls Group

The Compliance landscape and environment is becoming increasingly complex and challenging owing to enhanced Global focus on risks associated with Money laundering (ML), Terrorist Financing (TF) and Proliferation Financing (PF). Consequently the Compliance function is continuously striving to keep up with these challenges through a well-integrated and robust risk mitigation framework in the aforesaid areas of our banking operations. As heightened focus continues on tackling these issues, CCG is committed towards investing more in resources, processes and technology to combat these and other risk areas.

In order to remain abreast with regulatory requirements, CCG has pursued implementation of various dimensions of Compliance Risk Management (CRM) Guidelines issued by the regulator. In this regard, CCG aims to inculcate a compliance culture in the bank wherein ongoing regulatory requirements and industry challenges can be managed effectively in all jurisdictions of its operations.

CCG aims to ensure the highest standards of AML/CFT/CPF compliance, which requires management and employees to adhere to these standards by preventing use of Bank's delivery channels, products and services for money laundering, terrorist financing and proliferation financing. The Group provides a structural base to enable all concerned stakeholders to monitor out of pattern transactions to detect possible Money Laundering/ Terrorism Financing / Proliferation Financing activities

through Transaction Monitoring Solution (FCCM). Two Management sub-committees namely, AML/CFT sub-committee and Compliance Committee of Management oversee compliance with respect to relevant regulations, policies and procedures and steering of various AML/CFT/CPF initiatives in the Bank, to mitigate the risk of such activities for both domestic and overseas operations. Furthermore, CCG is also challenging all internal stakeholders to strengthen Bank's monitors with regards to ML/TF/PF risk emanating from Trade related business activities.

Highlights of 2020 include successful up gradation of Transaction Monitoring Solution (FCCM), implementation of system for KYC and Risk Profiling of Trade customers and implementation of specially designed SOPs for compliance of SBP's Framework on Trade Based Money Laundering (TBML).

Further, CCG also ensured bank wide training / awareness programs on AML / CFT / CPF for bank's staff all over the country. Moreover, CCG developed a structured model to continuously assess its inherent and residual Terrorism Financing risk based on threats and vulnerabilities. This activity also enables the bank to explore opportunities and strengthen its systems and controls to mitigate the residual risk.

CCG also oversees adherence to the regulatory requirements through onsite reviews, with specific emphasis on AML / CFT. It also performs overall evaluation of bank's Risk Control Self-Assessment (RCSA) Function and provides recommendations to enhance its effectiveness.

CCG ensures a professionally cordial working relationship with State Bank of Pakistan (SBP), Law Enforcement Agencies and other regulatory bodies in addition to coordination of SBP's Inspections. CCG has also been actively involved in pursuing Bank-wide implementation of SBP-led projects to digitize various Banking services and channels to facilitate customers' viz. Roshan Digital Accounts and end-to-end digitization of Foreign Exchange transaction requests of the customers. CCG played a meaningful role in enforcing large number of regulatory instructions issued by the regulator to mitigate the effects of covid-19 in the economy during the year 2020.

The Fraud Risk Management Department (FRMD) within CCG is responsible for mitigating Fraud Risk on pan bank basis. In addition to the management of Branch related fraud cases, FRMD also investigates Consumer & Digital banking related frauds, hence adopting a more focused approach towards managing Fraud risk. Further, Implementation of 'Internal Eye', a tool for reviewing and monitoring of transactions associated with employee accounts, enabled FRMD to monitor funds trafficking in employees accounts more guardedly. As a result of this, the scope of 'Internal Eye' will also cover the overseas branch going forward. In addition, FRMD is leading Fraud Risk Assessment Exercise Bank wide with an objective to build new controls or reinforcement of existing controls in order to counter the potential frauds and safeguard Bank's interest. Moreover, FRMD also spearheads resolution of regulatory observations through a cross functional Management sub-committee. In compliance with the Regulatory requirements of Employee Due Diligence, FRMD has designed Know Your Employee (KYE) Program, whereby, it also conducts KYE review exercise on quarterly basis by reviewing credentials of newly hired regular employees.

Our Service Quality (SQ) function, which is also part of CCG, continues its enhanced focus and rigor around customer experience through senior management oversight and continuous internal stakeholder engagement. Bank follows a multi-pronged approach to assess the quality of service standards for its customers. These measures include ongoing evaluation of our branch look & feel, speed of our product and service delivery and efficiency of our digital channels. The bank also seeks customer feedback on the same through its in-house Voice of Customer team. Weak areas identified through these initiatives are then worked upon to improve customer experience.

Fair treatment of customers continued to remain a priority agenda item for the bank throughout 2020. In addition to the class room trainings on "Fair Treatment of Customers", an e-learning program was also introduced in 2020 for frontline staff in order to increase staff coverage for this all important training module.

Service Quality function is also the custodian of customers' grievance handling and works in collaboration with all businesses / functions of the bank to ensure that not only customer complaints are managed effectively and efficiently, but also root causes leading to these complaints are eliminated.

Audit & Risk and Review Group

Internal audit function plays a pivotal role in the overall risk and control environment of any organization. The function provides assurance that is critical to the Board and management in assessing overall strength of an organization's control environment. Furthermore this function also adds value to the aforesaid control environment by virtue of its consultative role.

Audit & Risk Assets Review (Audit & RAR) Group is responsible for the internal audit function within MCB Bank Limited. Chief Internal Auditor functionally reports to the Board's Audit Committee and administratively to

the President. The Group conducts audits/reviews of various areas of the Bank under the globally recognized Risk Based Auditing Methodology whilst complying with the requirements of the International Standards for the Professional Practice of Internal Auditing issued by the Institute of Internal Auditors (USA) {IIA}.

Audit & RAR Group continued to perform its role effectively on both the assurance and consultative fronts during 2020. The Group evaluated efficacy of Bank's control systems by enhancing visibility of the management and the Board Audit Committee on the risk management and control related matters of the Bank (for Bank's domestic and overseas operations). The Group also highlighted areas for improvement and worked closely with the management through regular engagement as well as consistent follow-up, monitoring and guidance towards resolution of significant issues. Staff training and development remained a focus area for the Group which helped in enabling the internal audit teams in performance of their duties objectively as well as with professional due care.

With the zeal and commitment to play an effective role in the Bank's endeavor for continuous improvement, Audit & RAR Group will persevere for further strengthening of its resources, processes and Framework in 2021.

Legal Affairs Group

The mission of the Legal Affairs Group is to further the strategic goals and to protect and preserve the legal, ethical and financial integrity and the reputation of MCB Bank. This is accomplished by providing strategic legal advice on contentious and non-contentious matters, thereby ensuring that businesses conduct their activities in accordance with applicable laws and bye laws consistent with the mission, vision, and values of the MCB Bank. Legal compliance is the process or procedure to ensure that an organization follows relevant laws, regulations and business rules.

Legal Affairs Group comprises of two departments.

- Legal Affairs Department - Advisory
- Litigation Department

Legal Affairs Department - Advisory

The object of Legal Affairs Department – Advisory is to oversee the identified legal issues in all segments of business and their interrelation with, including marketing, sales, distribution, credit, finance, human resources, as well as corporate governance and business policy. This includes but is not limited to consultancy issues and adherence of processes for collateralization of finances. In this context, the Advisory Department analyses and reviews credits' security documentation of all segments of the Bank, like Corporate, Commercial, Retail and Consumer Banking and provide advice on perfection of documentation to secure Bank's interest.

At the helms of the affairs, diversity of work is exhaustive and apart from the major chunk of advice on securitization of Collaterals, it also includes review of all sorts of Agreements (Finance Agreements, Service Agreements, IT Agreements, Distributions Agreements, Non-disclosure Agreements, Product Agreements, Lease Agreements, E-Commerce Agreements etc.), Bank Guarantees, Advance Payment Guarantees, Mobilization Advance Payment Guarantees, Foreign Bank Guarantees, Financial Guarantees, LCs / Standby LCs, review of Product Manuals and their processes; SLAs are framed and transformed as per vendor's services across the board.

Corporate opinions are drafted and customer's relationship segments are assisted by review on the status of Corporate bodies, Partnership, Trust, Companies, funds etc. and Foreign Currency matters.

Advisory Department is also supportive by its quick advice and crisis management for queries by law enforcement agencies and public sector financial organizations like FIA/NAB, Anti-Narcotics, Police, Anti-Corruption, NAB, Customs /Income Tax /Sales Tax Departments/FBR etc.

More recently Advisory has also been actively engaged with SBP & PBA with regards to development of various upcoming laws and their amendments. This includes pragmatically reviewing and providing feedback/opinion on upcoming laws and their amendment with a view to secure the future interest of our business.

By standardizing different banking documents, the Legal Affairs Group has helped to introduce symmetry of documentation at all levels. Standardized templates have been uploaded on MCB Portal. Further, newly updated templates are uploaded and shared /exchanged with relevant business for implementation as when there is any amendment in relevant law/regulations etc.

During 2020, around 17,947 Opinions/Vettings were issued on collateral, security documents and allied legal issues raised by Business/Field, therefore advisory department has contributed in cost saving worth Millions of Rupees.

Litigation Department

The Litigation Department in coordination with businesses, oversees and handles bank wide litigation of different groups working within Bank and other litigation related proceedings pertaining to its customer or employees; Enlists Lawyers on panel in consultation and on the recommendation of relevant business/Group after conducting a detailed scrutiny via market check etc.;

Assigns cases in consultation and on recommendation of relevant business/Group; Negotiates Fee with the assigned lawyers in the cases assigned to them; Evaluates lawyers and their performance in cases assigned to them on bi-annual basis through directly monitoring their performance and on the basis of feedback received from the businesses and presents the same to LRC; Follow ups with businesses and updates centralized data of court cases on the basis of feedback received through court coordinators of respective businesses/Groups; Maintains centralized MIS of Litigation data and disseminates information in advance regarding fixation of cases to relevant business groups on daily basis; Reviews drafts of plaints/Appeals/Applications/FIRs and Settlement/ Rescheduling Agreements etc.; Renders opinions on court orders, stay orders, Summons/Warrants etc. as well as notices received from NAB, FIA & other LEAs.

This broad role encompasses Crisis Management for all segments of business for contentious and non-contentious matters of litigation and ancillary matters that arise directly or indirectly due to the litigation.

Following initiatives were taken by the Litigation Department during 2020:

- Retrieval/Compilation of Pan Pakistan Litigation Data/Record & Digitizing/Scanning of the same.
- Since Centralization of litigation expenses in June 2014, complete record with respect to litigation expenses is being maintained & shared with respective businesses on periodical basis.
- Development of Shadow Filing System which is in data entry phase. Once the same will be fully functional, it would enable litigation department to provide real time updates of court cases. It will also help in generating different reports for review of senior management and onward sharing with regulator and other departments/institutions.
- As many as four meetings of Litigation Review Committee (LRC) were held during the year 2020 and in said meetings, twelve (12) lawyers were enlisted on Bank's Approved panel of lawyers.
- The performance of three hundred & ten (310) lawyers was evaluated and discussed during LRC meetings on the basis of feedback received from the relevant businesses.
- In such meetings, the critical cases as well as recovery suits pending for decree for two years or more were discussed & the way forward/strategy for their expeditious disposal was also devised.
- As many as 754 cases, including recovery suits were filed during 2020 whereas 689 cases were disposed of/decreed out of the entire pending Litigation Portfolio as a result of strong follow up with the assigned lawyers which in turn resulted in substantial recovery of Bank's NPLs.
- Moreover, with the dismissal of cases, where damages were claimed against the bank, the bank was able to secure not only a sizeable amount of money but reputation of the bank was also safeguarded.
- Similarly, through the criminal litigation, especially in fraud & forgery incidents, the culprits were brought to book & were compelled to return the embezzled/ misappropriated amount and make good the bank's loss.
- A Large number of opinions on different legal matters were rendered in addition to vetting of all the pleadings (plaints, written statements/PLAs, Replications, Criminal Complaints and misc. applications) filed during the year 2020. Besides this, Settlement Agreements to be executed with the Customers/ Defaulters were also reviewed/vetted.
- As a result of negotiations with the panel lawyers, a hefty amount was saved on account of professional fee.

To strengthen itself, MCB Legal Affairs Group has shown significant growth in 2020 and is committed to perform up the curve in future to help the Bank to achieve better results.

Human Resource

MCB Bank follows the philosophy of working as an equal opportunity employer, in true spirit. We believe in initiating steps to foster and maintain a work climate that is conducive to achieving equal employment opportunities while ensuring a workplace free of discrimination and harassment. The bank believes in hiring and retaining talent who can contribute towards the achievement of all defined targets.

The Bank adopts a transparent performance management system developed on a defined KPI based scorecard for various business positions. The scorecard helps management define prioritization vis a vis objectives and setting future goals of its employees. The bank has instituted a Talent Management program with a view to identify high potential individuals and to groom them as future leaders.

The Bank has a vision to provide career progression opportunities to its employees; for this purpose an initiative was taken by the bank to convert a large number of individuals from an outsourced arrangement to Bank's Permanent employment pool. The conversion was also a step towards the fulfilling of several regulatory requirements.

To enhance the knowledge and skillset of its work force, a number of trainings were held during the year. Participants from all over the country were trained through different programs including in-house, ex-house, mobile,

and E-learning training programs. A segment comprising senior management was engaged through a Management Development Program as part of the Talent Management Scheme of the Bank.

Security

Security Division effectively ensured the safety and security of the Bank's physical assets despite numerous challenges during 2020. Through their persistent coordinated efforts, our security team meticulously managed security arrangements, ensured vigilant monitoring and supervision of branches across Pakistan, which translated into smooth and unimpeded branch operations.

MCB Bank branches are equipped with modern security equipment and manned by adequately skilled security staff that ensures banking transactions by customers and bank staff at branches are conducted in a safe and secure environment.

This year again, the security response rate was commendable, especially during national and other public holidays; all robbery attempts were successfully foiled owing to the robust and efficient security systems deployed at bank branches.

As in previous years, Regional Security Officers conducted security awareness training sessions for branch staff and security guards in their respective regions. With the right combination of trained and motivated man power and technology, Security Division is geared towards ensuring the safety of Bank's customers, assets and staff in the future.

Marketing Division

The Marketing Division is geared towards enhancing the positive image of the Bank through its corporate Brand Building, promotion of Bank's Product & Services on visible optimized mediums, standardization of corporate & brand communications, branch merchandizing, CSR and strong public relations.

During 2020, Marketing Division launched new TVCs for Roshan Digital Account, MCB Pyara Ghar Scheme and Awareness of Bank Alternate Delivery Channels during COVID19 Pandemic. Significant efforts were also undertaken by team to effectively support key regulator initiatives. The Division also played a pivotal role in providing support to marginalized communities and health workers on the frontlines of the battle against the COVID-19 Pandemic through targeted CSR initiatives. The Marketing Team also successfully managed and executed several internal events, such as the Bank's flagship Annual Sales Conference, its 72nd Anniversary ceremony and the Independence Day Illumination of MCB House in Lahore.

The Team revitalized its efforts to strengthen the Bank's presence on Social Media and used the communication channel effectively to create visibility for strategic brand alliances. The Division also took the initiative of creating tutorial and educational videos to encourage and facilitate the use of its financial solutions and services.

Going forward, Marketing Division will continue to focus on enhancing Brand visibility through a combination of available mediums, to reinforcing brand presence and create greater awareness of the Bank's products and services.

MCB Islamic Bank Limited

MCB Islamic Bank Limited (the "Bank") is the wholly owned subsidiary of MCB Bank Limited and commenced its operations in 2015 with a nation-wide network of branches.

The aim of MCB Islamic Bank Limited is to be the first choice Shari'ah compliant financial services provider for the customers and to carry out business purely in accordance with Shari'ah principles with full conviction and devotion.

Alhamdulillah, the Bank currently operates to provide Shari'ah compliant value added and innovative banking solutions for customers under the supervision of a Shari'ah Board chaired by the renowned Islamic scholar Professor Mufti Munib-Ur-Rehman.

The Bank focuses on building strong and lasting relationships as well as delivering an experience that satisfies all types of customers across Corporate, Commercial, SME, Consumer, Agriculture and Micro sectors. The Bank offers wide range of Riba Free and Shari'ah compliant products for both personal and business needs.

The Bank is using world's renowned Core Banking Software solution. Different services and products offered by the bank are available to customers through a branch network of 187 branches backed by 188 (176 On-Site and 12 Off-Site) ATMs. The Bank offers EMV enabled Debit Cards, IOS and Android based native Mobile Apps and Internet Banking services. Moreover, the Bank has also developed its suite of Cash Management services, including Payment upon Identification (PUI), Cash & Instrument Collection, Cash in Transit Services (CIT), Corporate Internet Banking by acquiring Oracle Banking Digital Experience (OBDX) and also working on development of product suit for Employee Banking under Cash Management umbrella.

The Bank is committed to share in the mutual benefits with the customers, staff and shareholders who participate in our business success under the highly skilled and seasoned management with the prime objective of ensuring our customers' satisfaction.

Corporate Governance

Corporate Governance at MCB Bank Limited ("MCB" or the "Bank") refers to rights and responsibilities among different stakeholders of the Bank through a set of rules, policies and practices keeping focus on proper delegation, transparency and accountability in the organization as a whole. The success of the Bank relies on its proven track record in upholding high standards of corporate governance.

Board Composition:

The Bank encourages representation of independent directors, non-executive directors and directors representing minority interests on its board of directors. At present the Board includes:

Category	Name
Independent Directors	Mr. Yahya Saleem<br>Mr. Salman Khalid Butt<br>Mr. Shahzad Hussain<br>Mr. Masood Ahmed Puri
Non-Executive Directors	Mian Mohammad Mansha<br>Mr. S. M. Muneer<br>Mr. Muhammad Tariq Rafi<br>Mian Umer Mansha<br>Mr. Mohd Suhail Amar Suresh bin Abdullah<br>Mr. Muhammad Ali Zeb<br>Mr. Shariffuddin Bin Khalid
Executive Director (President & CEO)	Mr. Imran Maqbool
Female Director (Non-Executive)	Mrs. Iqraa Hassan Mansha

Independent Directors and their Independence:

The Board has four (04) Independent Directors who meet the criteria of independence under the Companies Act, 2017 and the directives issued by the State Bank of Pakistan ("SBP").

Representation of Female Directors on Board:

Mrs. Iqraa Hassan Mansha is representing female Director on the Board.

Non-Executive Directors:

All the directors on the Board are Non-Executive Directors except for the President & CEO of the Bank. The Non-Executive Directors provide an outside viewpoint to the Board of Directors and are not involved in managing the affairs of the Bank.

Casual Vacancies on to Board of Directors:

No casual vacancy occurred on the Board of Directors during the year 2020.

Executive director serving as non-executive directors in other companies:

President & CEO, the Executive Director is serving on the Board of three (03) companies as Non-Executive Director.

Detail of Board Meetings held outside Pakistan:

During the year 2020, all the Board of Directors meetings were held in Pakistan.

Process of Appointment and Nomination of directors:

As per the provisions of the law, the directors are elected by the shareholders of the Bank, whereas, the casual vacancies on the Board are filled by the directors. Every director has to qualify the prior assessment criteria of 'Fit and Proper Test' as framed by the State Bank of Pakistan. While appointing a director, it is ensured that the Board is comprised of directors who have diversified experience, suitable knowledge, appropriate skill set/expertise and competency considered relevant in the context of the Bank's operations and to make the Board an effective decision making body. It is also ensured that the Board has an appropriate mix of directors including female member(s), diversity, size and well-structured to add value and to provide objective advice and have no material conflicts of interest.

Independent Directors are elected through the process of election of directors and meet the criteria laid down by the State Bank of Pakistan as well as comply with the requirements of the relevant rules and regulations. An independent director means a director who is not connected or does not have any other relationship, whether pecuniary or otherwise, with the company, its associated companies, subsidiaries, holding company or directors; and he can be reasonably perceived as being able to exercise independent business judgment without being subservient to any form of conflict of interest. At present, Independent Directors are selected from the data bank maintained by the Pakistan Institute of Corporate Governance.

Connection of External Search Consultancy for Selection of Independent Directors

The selection of independent directors as members of the Bank's Board of Directors is carried out from a list maintained by the Pakistan Institute of Corporate Governance ("PICG") under the Companies (Manner and Selection of Independent Directors) Regulations, 2018.

PICG has no other connections with the Bank, except for providing access to the database on independent directors besides directors' training and evaluation of Board and / or individual directors' performance.

Directors' Participation/Attendance in Board and Committee Meetings held during 2020

Sr. No.	Name of Director	Board of Directors (BoD)		Board's Committees: AC		BS&DC		RM&PRC		CR&MC		ITC		HR&RC		PP&CA		WO&WC	
		Member	Attended	Member	Attended	Member	Attended	Member	Attended	Member	Attended	Member	Attended	Member	Attended	Member	Attended	Member	Attended
1	Mian Mohammad Mansha	◆ (Chairman)	5/5	–	–	◆	4/4	–	–	–	–	–	–	◆	4/4	◆	4/4	–	–
2	Mr. S. M. Muneer	◆	5/5	–	–	◆	1/4	–	–	◆ (Chairman)	1/4	–	–	–	–	–	–	–	–
3	Mr. Muhammad Tariq Rafi	◆	5/5	–	–	–	–	–	–	–	–	–	–	–	–	–	–	◆	2/3
4	Mian Umer Mansha	◆	5/5	◆	5/5	◆ (Chairman)	4/4	◆	5/5	–	–	◆ (Chairman)	5/5	–	–	◆ (Chairman)	4/4	◆ (Chairman)	3/3
5	Mrs. Iqraa Hassan Mansha	◆	3/5	–	–	–	–	–	–	–	–	–	–	◆	2/4	◆	1/4	–	–
6	Mr. Muhammad Ali Zeb	◆	5/5	◆	5/5	–	–	◆ (Chairman)	5/5	◆	4/4	–	–	◆	3/4	◆	3/4	◆	3/3
7	Mr. Mohd Suhail Amar Suresh	◆	5/5	–	–	◆	4/4	◆	5/5	–	–	◆	5/5	–	–	–	–	–	–
8	Mr. Yahya Saleem	◆	5/5	–	–	–	–	–	–	–	–	◆	2/5	◆ (Chairman)	3/4	–	–	–	–
9	Mr. Salman Khalid Butt	◆	5/5	–	–	◆	4/4	◆	5/5	◆	4/4	◆	5/5	–	–	–	–	–	–
10	Mr. Shahzad Hussain	◆	5/5	◆ (Chairman)	5/5	–	–	–	–	–	–	–	–	–	–	–	–	–	–
11	Mr. Masood Ahmed Puri	◆	5/5	–	–	◆	4/4	–	–	–	–	–	–	–	–	–	–	–	–
12	Mr. Shariffuddin Bin Khalid	◆	5/5	◆	5/5	–	–	–	–	–	–	–	–	–	–	–	–	–	–
13	Mr. Imran Maqbool (President & CEO)	◆	5/5	–	–	◆	4/4	◆	5/5	◆	4/4	◆	5/5	–	–	◆	4/4	–	–
Total Number of Meetings Held		5		5		4		5		4		5		4		4		3	

Chairman ◆	Member ◆

**AC:**	Audit Committee	**ITC:**	Information Technology Committee
**BS&DC:**	Business Strategy & Development Committee	**PP&CA:**	Committee on Physical Planning & Contingency Arrangements
**RM&PRC:**	Risk Management & Portfolio Review Committee	**CR&MC:**	Compliance Review & Monitoring Committee
**HR&RC:**	Human Resource & Remuneration Committee	**WO&WC:**	Write-Off & Waiver Committee

The names of the persons who, at any time during the financial year, were directors of the Bank:

• Mian Mohammad Mansha	• Mr. Mohd Suhail Amar Suresh bin Abdullah
• Mr. S. M. Muneer	• Mr. Yahya Saleem
• Mr. Muhammad Tariq Rafi	• Mr. Salman Khalid Butt
• Mian Umer Mansha	• Mr. Shahzad Hussain
• Mrs. Iqraa Hassan Mansha	• Mr. Masood Ahmed Puri
• Mr. Muhammad Ali Zeb	• Mr. Shariffuddin Bin Khalid

Disclosure on Board of Directors

Sr. No.	Name of Directors	Date of Joining/ Leaving the Board	Status of Director	Member of Board Committees*	Number	Name of Companies
1	Mian Mohammad Mansha	Apr-91	Non-Executive Director	- HR&RC<br>- BS&DC<br>- PP&CA	1	MCB Non-Bank Credit Organization, CJSC, Azerbaijan (Formerly: MCB Leasing CJSC, Azerbaijan)
2	Mr. S. M. Muneer	Apr-91	Non-Executive Director	- BS&DC<br>- CR&MC	5	Din Textile Mills Limited<br>Din Leather (Pvt.) Limited<br>Din Farm Products (Pvt.) Limited<br>Arabian Sea Country Club<br>Din Industries Limited
3	Mr. Muhammad Tariq Rafi	Apr-91	Non-Executive Director	- WO&WC	5	Siddiqsons Limited<br>Siddiqsons Tin Plate Limited<br>Central Depository Co. of Pakistan Limited<br>Triple Tree Associates<br>Siddiqsons Energy Limited
4	Mian Umer Mansha	11-Nov-97/ 08-Sep-2007 & 27-Mar-2009	Non-Executive Director	- AC<br>- BS&DC<br>- RM&PRC<br>- PP&CA<br>- ITC<br>- WO&WC	11	Nishat Mills Limited<br>Adamjee Insurance Company Limited<br>Adamjee Life Assurance Company Limited<br>Nishat Hotels & Properties Limited<br>Nishat Developers (Pvt.) Limited<br>Nishat (Raiwind) Hotels and Properties Limited<br>Nishat Dairy (Pvt.) Limited<br>Nishat Agriculture Farming (Pvt.) Limited<br>Hyundai Nishat Motor (Pvt.) Limited<br>Nishat Agrotech Farms (Pvt) Limited<br>Nishat Sutas Dariy Limited
5	Mrs. Iqraa Hassan Mansha	03-May-16	Non-Executive Director	- HR&RC<br>- PP&CA	4	Nishat Hotels & Properties Limited<br>Nishat (Raiwind) Hotels and Properties Limited<br>Nishat Real Estate Development Company (Private) Limited<br>Emporium Properties (Pvt) Limited
6	Mr. Muhammad Ali Zeb	27-Mar-09/07-Apr-2011 & 17-Jun-2013	Non-Executive Director	- AC<br>- RM&PRC<br>- HR&RC<br>- CR&MC<br>- PP&CA<br>- WO&WC	3	Adamjee Insurance Company Limited<br>Adamjee Life Assurance Company Limited<br>Nishat Sutas Dairy Limited
7	Mr. Mohd Suhail Amar Suresh	24-Feb-14	Non-Executive Director	- RM&PRC<br>- BS&DC<br>- ITC	3	Maybank Shared Services Sdn Bhd<br>MBB Labs Pvt Ltd<br>Technology Park Malaysia Corporation Sdn Bhd (TPM)

Sr. No.	Name of Directors	Date of Joining/ Leaving the Board	Status of Director	Member of Board Committees*	Number	Name of Companies
8	Mr. Yahya Saleem	27-Mar-18	Non-Executive Independent Director	- HR&RC<br>- ITC	6	Saleem Memorial Trust Hospital
						NC Entertainment Private Limited
						NC Trading USA
						YSG Trading Private Limited
						YSG Services Canada
						YSG Services Private Limited
9	Mr. Salman Khalid Butt	10-Nov-18	Non-Executive Independent Director	- BS&DC<br>- RM&PRC<br>- CR&MC<br>- ITC	4	Saleem Memorial Trust Hospital
						New Heights Management Ltd, a BVI Company
						New Heights Concepts Ltd, a BVI Company
						Next Commercial FZ LLC, a Ras El Khaimah (Rakez), U.A.E. Company
10	Mr. Shahzad Hussain	31-May-19	Non-Executive Independent Director	- AC	2	NAMAL Education Foundation
						Punjab Health Care Commission
11	Mr. Masood Ahmed Puri	31-May-19	Non-Executive Independent Director	- BS&DC	3	Transarab Trading Services (KSA)
						Jedex Logistics Pvt. Ltd (Pakistan)
						Jedex Transport Company LLC (UAE)
12	Mr. Shariffuddin Bin Khalid	23-Jul-19	Non-Executive Director	- AC	8	Malayan Banking Berhad
						Maybank (Cambodia) Plc
						Maybank Islamic Berhad
						Marine & General Berhad
						M&G Marine Logistics Holdings Sdn. Bhd.
						M&G Tankers Sdn. Bhd.
						Jasa Marin (Labuan) Plc
						M&G Ship Management (L) Pte Limited
13	Mr. Imran Maqbool President & CEO	21-Dec-12	Executive Director	- BS&DC<br>- RM&PRC<br>- CR&MC<br>- ITC<br>- PP&CA	3	Adamjee Insurance Company Limited
						Adamjee Life Assurance Company Limited
						MCB Employees Foundation

**AC:**	Audit Committee	**ITC:**	Information Technology Committee
**BS&DC:**	Business Strategy & Development Committee	**PP&CA:**	Committee on Physical Planning & Contingency Arrangements
**RM&PRC:**	Risk Management & Portfolio Review Committee	**CR&MC:**	Compliance Review & Monitoring Committee
**HR&RC:**	Human Resource & Remuneration Committee	**WO&WC:**	Write-Off & Waiver Committee

Board Committees

The Board has formed eight (8) sub-committees as given below:

1. Audit Committee;
2. Business Strategy & Development Committee;
3. Risk Management & Portfolio Review Committee;
4. Compliance Review & Monitoring Committee;
5. Information Technology Committee;
6. Human Resource & Remuneration Committee;
7. Committee on Physical Planning & Contingency Arrangements; and
8. Write-Off & Waiver Committee.

Audit Committee

Composition:

1. Mr. Shahzad Hussain - (Chairman, Independent Director)
2. Mian Umer Mansha - (Non-Executive Director)
3. Mr. Muhammad Ali Zeb - (Non-Executive Director)
4. Mr. Shariffuddin Bin Khalid - (Non-Executive Director)

Terms of Reference

The main Terms of Reference/ roles & responsibilities of the Committee are:

1. Determination of appropriate measures to safeguard the bank's assets;
2. Reviewing annual and interim financial statements of the bank, prior to their approval by the Board of Directors, focusing on:
   - Major judgmental areas;
   - Significant adjustments resulting from the audit;
   - The going concern assumption;
   - Any changes in accounting policies and practices;
   - Compliance with applicable accounting standards;
   - Compliance with listing regulations, other Statutory and regulatory requirements; and
   - All related party transactions.
3. Reviewing preliminary announcements of results prior to external communication and publication;
4. Facilitating the external audit and discussion with external auditors of major observations arising from interim and final audits and any matter that the auditors may wish to highlight (in the absence of management, where necessary);
5. Reviewing Management Letter issued by External Auditors and management's response thereto;
6. Ensuring coordination of internal auditors with external auditors of the Bank and SBP inspection team(s);
7. Making recommendations to the Board of Directors for the appointment of external auditors, their removal, audit fees, the provision of any service permissible to be rendered to the Bank by the external auditors in addition to audit of its financial statements, measures for redressal and rectification of non-compliances with the Regulations. The Board shall give due consideration to the recommendations of the audit committee and where it acts otherwise it shall record the reasons thereof;
8. Reviewing and recommending of the Internal Audit Framework i.e. internal audit function's policies and manuals (also covering extent and nature of assignments/ engagements that can be provided to the management by internal audit function in its 'Advisory / Consulting' role) to the Board for approval.
9. Reviewing, internal audit strategy, reporting framework and procedures developed by Chief Internal Auditor and ensuring its fullest support to the internal audit function and internal auditors enabling them in performing their mandated activities independently and in objective manner;
10. Reviewing and approving scope and extent of internal audit as well as risk based annual audit plan. Furthermore, reviewing the implementation status of the approved audit plan on a quarterly basis.
11. Ensuring that the internal audit function has adequate resources (financial, human, operational, physical and technological), its staff is adequately trained and the function is appropriately placed within the bank with access to Bank's people, information, processes, properties, records and systems;
12. Ensuring independence of internal audit function, independence & objectivity of internal auditors, optimal utilization of audit resources, effectiveness of the internal audit function in Bank's overall governance and internal control framework and constructive engagement of internal audit function with senior management and auditee units etc.;
13. Reviewing and recommending budget / resource requirement of Audit & RAR Group to the Board for approval. Further, reviewing, on a quarterly basis, the utilization of Audit & RAR Group's assigned budget and if required, recommending provision of additional resource(s) to enable the Group in performance of its activities;
14. Formulating 'Key Performance Indicators' (KPIs) for the Chief Internal Auditor and evaluate his/her performance against set KPIs on an annual basis;
15. Approving appointment/ re-hiring/ renewal of contract and removal of Chief Internal Auditor along with his/her compensation package/remuneration (including performance based bonus, increments, cash rewards etc.), allied benefits (both financial/ non-financial), promotion/demotion and other terms of employment to the Board through Board's Human

Resources & Remuneration Committee. However, recommendation of Board's Human Resource & Remuneration Committee may be sought by the Audit Committee/ Board regarding compensation package of Chief Internal Auditor, keeping in view the institution-wide remuneration policy, formulated in terms of BPRD Circular No. 01 of 2017;

16. Consideration of major findings of internal investigations of activities characterized by fraud, corruption and abuse of power and management's response thereto;
17. Reviewing summaries of quarterly report on frauds/ forgeries/ dacoities;
18. Reviewing, on quarterly basis, summary of significant violations/observations, internal control deficiencies, organizational and personal material conflicts of interest etc. to have deep insights into state of internal controls and to set specific, time bound action points/indicators to monitor improvements. Further reviewing the management action plan to ensure that audit observations/ recommendations receive proper and timely attention by senior management;
19. Analyzing and identifying (for necessary action(s)), on a regular basis, 'root cause(s)' of control breaches of critical nature that keep on occurring in at-least two audit periods despite implementation of audit recommendations;
20. Obtaining Chief Internal Auditor's independent annual assessment/opinion on the state of Bank's internal controls based on the audits conducted over the period;
21. Ascertaining that the internal control systems including financial and operational controls, accounting systems for timely and appropriate recording of purchases and sales, receipts and payments, assets and liabilities and the reporting structure are adequate and effective;
22. Reviewing the Bank's statement on internal control systems prior to endorsement by the Board of Directors and internal audit reports;
23. Instituting special projects, value for money studies or other investigations on any matter specified by the Board of Directors, in consultation with the Chief Executive Officer ("CEO") and to consider remittance of any matter to the external auditors or to any other external body;
24. Determination of compliance with relevant statutory requirements;
25. Establishing, maintaining and promoting regular communication with senior management regarding deficiencies in internal controls; review actions taken by management to address identified deficiencies and ascertaining new developments to achieve a uniform organization-wide commitment/ buy-in for implementation of strong and effective internal controls;
26. Reviewing effectiveness of Whistle Blowing procedures for receiving (through internal or external sources) complaints/concerns regarding business ethics/conduct practices, governance & risk management practices, controls over financial reporting, auditing practices etc. Ensuring that such complaints / concerns are treated confidentially and that the reporting employee(s) are protected and not penalized in any manner whatsoever. Ensuring that employees remain aware of existence of such procedures including mechanism for utilizing them and are encouraged to be a 'whistleblower';
27. Review of arrangement for staff and management to report to audit committee in confidence, concerns, if any, about actual or potential improprieties in financial and other matters and recommend instituting remedial and mitigating measures;
28. Reviewing, on a periodic basis, the Internal Controls over Financial Reporting (ICFR) system for its effective implementation and its continuous up gradation.
29. Monitoring compliance with the Listed Companies (Code of Corporate Governance) Regulations, 2019 and identification of significant violations thereof;
30. Consideration of any other issue or matter as may be assigned by the Board of Directors.

Business Strategy & Development Committee

Composition:

1. Mian Umer Mansha - (Chairman, Non-Executive Director)
2. Mian Mohammad Mansha - (Non-Executive Director)
3. Mr. S. M. Muneer -(Non-Executive Director)
4. Mr. Mohd Suhail Amar Suresh - (Non-Executive Director)
5. Mr. Salman Khalid Butt - (Independent Director)
6. Mr. Masood Ahmed Puri - (Independent Director)
7. President & CEO - (Executive Director)

Terms of Reference

The main Terms of Reference of the Committee are to:

1. Review and develop Vision & Mission statements and core values for MCB both from long and short term perspective.
2. Develop Bank's initiatives relating to business philosophy and acquisition, strategic investment and divestment, capital raising exercise, strategic alliances and brand management. In particular, review the following important matters:
   a. Policy initiatives;
   b. Business organization;
   c. Oversee expansion plans; and
   d. Contingency planning relating to business realignment.

3. Review and devise medium and long term business plans and policies based on strategy, future direction and milestones set by the Board.
4. Monitor the progress of the key strategy initiatives undertaken by the Bank.
5. Keep oversight on Bank's Overseas Operations.
6. Undertake such other tasks as may be delegated by the Board from time to time.

Risk Management & Portfolio Review Committee

Composition:

1. Mr. Muhammad Ali Zeb - (Chairman, Non-Executive Director)
2. Mian Umer Mansha - (Non-Executive Director)
3. Mr. Mohd Suhail Amar Suresh - (Non-Executive Director)
4. Mr. Salman Khalid Butt - (Independent Director)
5. President & CEO - (Executive Director)

Terms of Reference

The main Terms of Reference of the Committee are to:

1. Review the Bank's Risk Management Framework, in light of internal developments, guidelines issued by the regulators and international best practices, on as and when required basis and recommend to the Board for approval;
2. Ensure Bank's compliance towards Capital Adequacy and other related Basel/ regulatory requirements on an on-going basis. Review Capital Adequacy Ratio (CAR) in detail on half yearly basis;
3. Review and recommend to Board, Bank's Risk Appetite Statement on an annual basis;
4. Review various reports pertaining to the risk in the bank's portfolio prepared by the Risk Management Group. The Committee shall also consider comments of the relevant senior management official/ Committee while reviewing such reports and communicate the planned/executed corrective actions to the Board, if required;

Compliance Review & Monitoring Committee

Composition:

1. Mr. S. M. Muneer - (Chairman, Non-Executive Director)
2. Mr. Muhammad Ali Zeb - (Non-Executive Director)
3. Mr. Salman Khalid Butt - (Independent Director)
4. President & CEO - (Executive Director)

Terms of Reference

The main Terms of Reference of the Committee are:

1. To review six monthly reports, prepared by the Compliance & Controls Group and routed through the President, on overall compliance risk management in the Bank (local as well as overseas operations) including the actions taken on the recommendations and observations of SBP in its Annual/Thematic Inspection Reports;
2. To evaluate the effectiveness of the Bank's overall management of compliance risk, at least annually; keeping in view the regulatory observations in on-site examinations, regulatory enforcement actions, internal assessments/feedback from internal audit, compliance reviews, as well as interactions with the Chief Compliance Officer ("CCO");
3. To carry on liaison between the Board and the management and make recommendations to the Board, if necessary, for taking decisions on expedient and appropriate disposal of compliance issues raised through Compliance Committee of Management ("CCM") and SBP's inspection reports and other AML/CFT related identified issues;
4. To oversee the effectiveness of Service Quality function of the Bank and to review the Bank's performance against committed service deliverables.
5. To review/recommend Compliance Risk Strategy/ Policy, Compliance Program and allied policies and oversee its implementation across the Bank in letter and spirit;
6. To recommend appointment of CCO on the advice of the President and ensure that position of CCO does not remain vacant for more than 60 days. Furthermore, to approve any disciplinary action or termination of the CCO;
7. To ensure that CCO has appropriate stature, authority, resources (physical, financial and human), support to fulfill his duties, independence and capacity to offer his objective opinions and advise to senior management and Board on matters of compliance risks;
8. To ensure that Compliance Function ("CCG") has subject experts on various critical areas such as risk management, credit operations, product compliance, customer service, international trade, outsourcing, corporate governance, financial disclosures, business continuity, information technology, general banking operations, AML/CFT etc. to provide guidance to business areas as and when required.
9. To ensure that Compliance Function ("CCG"), being the second line of defence, assists line managers/ departments in designing and implementing adequate controls to manage risks of non-compliance and monitors and assesses bank-wide compliance risk and reports risk profile to Board and Compliance Committee of Management.
10. To engage with the CCO on half yearly basis, for his feedback on issues faced by the Compliance Function ("CCG") in the implementation of board approved compliance program;

11. To review the minutes of Compliance Committee of Management (CCM) meetings to ascertain its effectiveness in managing compliance risk;
12. To review the progress in implementing remedial actions taken with respect to instances of non-compliance or control weakness as identified by Compliance Function through its regular compliance reviews and / or various other sources;
13. To review the compliance risk relevant agenda items as required under SBP regulations/ instructions of overseas jurisdictions, AML/CFT perspective and SBP inspection reports' observations as per their respective timelines/ frequencies (quarterly, semi-annually or annually);
14. To satisfy itself of receiving the accurate as well as comprehensive information required to perform its compliance risk oversight responsibilities, including seeking assurances from Senior Management that the compliance risk controls have been implemented and are working effectively;
15. To ensure changes in the Rules/Regulations and Laws are reviewed on an ongoing basis in the existing policies or through introduction of new policies.
16. The Committee would recommend to the Management for updation of existing policies of the Bank's local and overseas operations, if required, and/or determine the need for introduction of new policies, in accordance with the changes in the following:
    a. Local laws, including but not limited to the SBP Act, Banking Companies Ordinance, Prudential Regulations, Code of Corporate Governance, Securities & Exchange Commission of Pakistan Instructions, Rules, Regulations & Circulars and Listing Regulations of Stock Exchanges;
    b. Existing and prospective business environment; and
    c. Operational requirements.
17. Any other issue that is deemed necessary and required by the regulations.

Information Technology Committee

Composition:

1. Mian Umer Mansha - (Chairman, Non-Executive Director)
2. Mr. Yahya Saleem - (Independent Director)
3. Mr. Mohd Suhail Amar Suresh - (Non-Executive Director)
4. Mr. Salman Khalid Butt - (Independent Director)
5. President & CEO - (Executive Director)

Terms of Reference

The main Terms of Reference of the Committee are:

1. To approve an overall plan for IT system for the Bank, prepared by the management;
2. To approve the organizational IT and Digital strategic plans to ensure an effective use of information technology and digital initiatives in the Bank by all Departments;
3. To approve and oversee the management's program to automate the organization's use of internal information to ensure that data is organized and shared in a manner that adds value and enhances productivity;
4. To approve and oversee a reliable and secure information security infrastructure with the capacity to address future threats;
5. To approve policies that promote development of information technology and Digital resources in an organized, deliberate, secured, and cost effective manner;
6. To review and approve management recommendations for IT standards for ensuring compliance with regulatory requirements and identifying and mitigating significant IT related risks;
7. To review and approve the technology procurements as per Bank's approved IT Strategy. The Committee may further delegate the same to the management as and when deemed fit;
8. To undertake any other IT or Digital Banking related work assigned to the Committee by the Board.

Human Resource & Remuneration Committee

Composition:

1. Mr. Yahya Saleem - (Chairman, Independent Director)
2. Mian Mohammad Mansha - (Non-Executive Director)
3. Mrs. Iqraa Hassan Mansha - (Non-Executive Director)
4. Mr. Muhammad Ali Zeb - (Non-Executive Director)

Terms of Reference

The main Terms of Reference of the Committee shall be to ensure that:

1. The existing policies are reviewed periodically and as necessary, revised and recommended to the Board, in order to attract and retain highly qualified employees. This may include review of remuneration policy and remuneration setting mechanism at least once every three years. Among other factors, the review of remuneration framework shall include, but not limited to:
    a. The effectiveness of remuneration policy and mechanism i.e., whether it is providing the expected outcomes;
    b. Any necessary changes required; and
    c. Any unintended consequences.

    The findings of review and rectification measures shall be presented to the Board for approval.

2. The latest entry-level procedures are put in place for recruitment of entrants.
3. The existing training facilities for the new entrants as well as for up-gradation of skill level of all employees are reviewed and revised, if required.
4. Proper classification & reclassification of employees' pay scales, job description and methods of their periodical review are put in place.
5. The Bank-wide remuneration policy takes into account all cadres of employees along with the pay-gap between the highest paid and the lowest paid employees, across various levels as well as across the Bank. The pay-gap shall be based on the total compensation awarded to the employees and should be maintained at reasonable levels without allowing concentration of the benefits of Bank's performance only to top level or certain class or group of employees. The pay structure for all employees should be fair, competitive and encourage performance and motivation.
6. That the remuneration policy of the Bank provides for reasonable levels of compensation for contractual employees of Bank and commensurate with their assignments.
7. That a separate structure of remuneration for Material Risk Controllers ("MRC") and Material Risk Takers ("MRTs") is developed as per remuneration policy. The MRTs should be identified as functions and designations rather than as individuals. The MRTs should have appropriate level of authority and control.
8. An objective criterion for work appraisal/performance is developed and linked with the annual merit increase.
9. A review is undertaken of the organizational structure to bring it in line with business strategy & development plan and approve an organizational set up or any revision in the existing set up taking into account the recommendations of the President & CEO.
10. An in-house human resource expertise is developed to undertake market analysis of above policies with a view to developing MCB policies.
11. If so required, the Committee can seek independent external advice/expert opinion for accomplishment of devising an effective and prudent remuneration framework.
12. Effective management information system is developed to monitor the implementation of policies as approved by the Board.
13. Recommendation to the Board for consideration and approval of a policy framework for determining remuneration of directors (both executive and non-executive directors) and members of Senior Management. The definition of senior management will be determined by the Board which shall normally include the first layer of management below the Chief Executive Officer.
14. Selection, evaluation, compensation (including retirement benefits) and succession planning of the CEO, COO, CFO, Company Secretary and Head of Internal Audit and recommendation to the Board.
15. The structure of compensation package of Executive Directors, CEO, Key Executives and any other employee or group of employees Bank-wide is recommended to the Board for its own approval or approval of the shareholders, according to legal and regulatory requirements.
16. The consideration and approval on recommendations of CEO on such matters for key management positions who report directly to CEO.
17. In devising the Remuneration setting policy and mechanism, the Committee can also seek internal inputs from any other Committee of the Board or directly from any department or official of the Bank.

Committee on Physical Planning & Contingency Arrangements

Composition:

1. Mian Umer Mansha - (Chairman, Non-Executive Director)
2. Mian Muhammad Mansha – (Non-Executive Director)
3. Mrs. Iqraa Hassan Mansha - (Non-Executive Director)
4. Mr. Muhammad Ali Zeb - (Non-Executive Director)
5. President & CEO - (Executive Director)

Terms of Reference

The main Terms of Reference of the Committee are:

1. To develop and devise an overall plan for physical infrastructure and contingency arrangements for the Bank;
2. To review and monitor all work in progress, including construction of premises and renovations, which shall, inter alia, be based on physical planning;
3. To review, monitor and recommend to the Board the building plans, master development agreements & contingency arrangements;
4. To review, from time to time, as the Committee deems appropriate, the administrative structures and plans in place to ensure the ongoing health and safety of utilities and physical assets, including land & buildings and recommend, as appropriate, changes in plans arising from this review;
5. To review updates on Bank's property purchases.

Write-Off & Waiver Committee

Composition:

1. Mian Umer Mansha - (Chairman, Non-Executive Director)
2. Mr. Muhammad Tariq Rafi - (Non-Executive Director)
3. Mr. Muhammad Ali Zeb - (Non-Executive Director)

Terms of Reference

The main Terms of Reference of the Committee are:

1. To review and approve write-off & waiver cases on behalf of the Board of Directors and;
2. To submit cases of write-off & waiver for post facto ratification by the Board.

Management Committees

Management committee

1. Mr. Imran Maqbool - Chairman
2. Mr. Malik Abdul Waheed
3. Mr. Hammad Khalid
4. Mr. Kamran Zaffar Muggo
5. Mr. Farid Ahmad
6. Mr. Usman Hassan
7. Mr. Muhammad Nauman Chughtai
8. Mr. Syed Mudassar Hussain Naqvi
9. Mr. Zargham Khan Durrani
10. Mr. Abrar Aleem
11. Mr. Hassan Nawaz Tarar
12. Mr. Shahzad Ishaq
13. Mr. Salman Y Zaidi
14. Mr. Shoaib Mumtaz

Purchase & Expense Committee

1. Mr. Kamran Zaffar Muggo
2. Mr. Hammad Khalid
3. Mr. Hassan Nawaz Tarar
4. Mr. Usman Hassan

Assets & Liabilities Committee

1. Mr. Imran Maqbool - Chairman
2. Mr. Hammad Khalid
3. Mr. Muhammad Nauman Chughtai
4. Mr. Salman Y Zaidi
5. Mr. Kamran Zaffar Muggo
6. Mr. Zargham Khan Durrani
7. Mr. Shahzad Ishaq
8. Mr. Shoaib Mumtaz

Write off Committee

1. Mr. Imran Maqbool - Chairman
2. Mr. Hammad Khalid
3. Mr. Muhammad Nauman Chughtai
4. Mr. Zargham Khan Durrani
5. Mr. Shoaib Mumtaz
6. Mr. Omair Safdar
7. Mr. M. Haris Hasan
8. Mr. Muhammad Ali

Investment Committee

1. Mr. Imran Maqbool - Chairman
2. Mr. Omair Safdar
3. Mr. Salman Y Zaidi
4. Mr. Hammad Khalid
5. Mr. Muhammad Nauman Chughtai
6. Mr. Shoaib Mumtaz

IT Steering Committee

1. Mr. Imran Maqbool - Chairman
2. Mr. Hammad Khalid
3. Mr. Muhammad Nauman Chughtai
4. Mr. Kamran Zaffar Muggo
5. Mr. Zargham Khan Durrani
6. Mr. Abrar Aleem
7. Mr. Shoaib Mumtaz

Disciplinary Action Committee

1. Mr. Usman Hassan - Chairman
2. Mr. Kamran Zaffar Muggo
3. Mr. Farid Ahmad
4. Mr. Muhammad Nauman Chughtai
5. Mr. Hassan Nawaz Tarar

Litigation Review Committee

1. Mr. Syed Mudassar Hussain Naqvi - Chairman
2. Mr. Malik Abdul Waheed
3. Mr. M. Haris Hasan
4. Mr. Muhammad Ali
5. Mr. Zargham Khan Durrani
6. Mr. Shahzad Ishaq
7. Mr. Usman Hassan
8. Mr. Mansoor Qadir
9. Mr. Amir Nawab

Management Credit & Risk Committee

1. Mr .Imran Maqbool - Chairman
2. Mr. Muhammad Nauman Chughtai
3. Mr. Rizwan Chughtai – Acting member

Overseas Monitoring Committee

1. Mr. Malik Abdul Waheed - Chairman
2. Mr. Hammad Khalid
3. Mr. Muhammad Nauman Chughtai
4. Mr. Farid Ahmad
5. Mr. Kamran Zaffar Muggo
6. Mr. Shoaib Mumtaz

Compliance Committee of Management

1. Mr. Imran Maqbool - Chairman
2. Mr. Farid Ahmad
3. Mr. Malik Abdul Waheed
4. Mr. Kamran Zaffar Muggo
5. Mr. Usman Hassan
6. Mr. Muhammad Nauman Chughtai
7. Mr. Syed Mudassar Naqvi
8. Mr. Zargham Khan Durrani
9. Mr. Abrar Aleem
10. Mr. Shoaib Mumtaz

11. Mr. Shahzad Ishaq

Cyber Security Committee

1. Mr. M. Nauman Chughtai - Chairman
2. Mr. Shahzad Ishaq
3. Mr. Farid Ahmad
4. Mr. Abrar Aleem
5. Mr. Kamran Zaffar Muggo
6. Mr. Shoaib Mumtaz
7. Mr. Zargham Khan Durrani

Outsourcing Relationship Review Committee

1. Mr. Kamran Zaffar Muggo
2. Mr. M. Nauman Chughtai
3. Mr. Farid Ahmad

Management Sub-Committee for Resolution of Long Outstanding Audit Issues

1. Mr. Malik Abdul Waheed - Chairman
2. Mr. Kamran Zaffar Muggo
3. Mr. Shahzad Ishaq
4. Mr. Hammad Khalid
5. Mr. Abrar Aleem
6. Mr. Zargham Khan Durrani

Management Sub-Committee on AML/CFT

1. Mr. Farid Ahmad - Chairman
2. Mr. Malik Abdul Waheed
3. Mr. Kamran Zaffar Muggo
4. Mr. Zargham Khan Durrani
5. Mr. Muhammad Nauman Chughtai
6. Mr. Shoaib Mumtaz

Management Sub-Committee for Monitoring of Central Banks' Inspection Reports

1. Mr. Malik Abdul Waheed - Chairman
2. Mr. Farid Ahmad
3. Mr. Kamran Zaffar Muggo
4. Mr. M. Nauman Chughtai
5. Mr. Abrar Aleem

Performance Evaluation of the Board of Directors, its Committee and Individual Directors:

During the year, MCB Bank Limited ("MCB" or the "Bank") performed an in-house performance evaluation of the Board, the Chairman, Individual Directors including Independent Directors, the President & CEO and Board's Committees in line with the 'Guidelines on Performance Evaluation of the Board of Directors' ("SBP Guidelines") issued by the State Bank of Pakistan ("SBP") and the Listed Companies (Code of Corporate Governance) Regulations, 2019 ("CCG Regulations").

MCB Board, since 2014, has put a formal and an effective mechanism for annual performance evaluation of the Board, its Committees and Individual Directors as per the requirements of CCG Regulations as amended and enacted from time to time. In 2016, the Bank amended its scope and methodology to align it in conformity with the SBP Guidelines and conducted the performance evaluation of overall Board, its Committees and Individual Directors for the ensuing years accordingly.

Performance Evaluation through External Independent Evaluator:

SBP under BPRD Circular No. 11 of 2016 dated August 22, 2016 required that Board evaluation should be undertaken by an external independent evaluator at least once in every three years. Last year, the Bank also engaged the Pakistan Institute of Corporate Governance ("PICG") as an Independent External Evaluator, who conducted the performance evaluation of the Board, its Committees, the Chairman, the President & CEO and Individual Board Members.

Criteria for Annual Performance Evaluation of the Overall Board of Directors:

While conducting the annual performance evaluation of the Board as a whole, the Bank in line with the SBP Guidelines adopted an in-house approach. The overall rating of the Board was found highly encouraging, particularly, in respect of its composition, expertise, effective risk management, adequate system of internal controls and audit function. The Board considers the long-term policy-related matters and sets the Bank's strategic aims to put them into effect and to uphold the vision, mission and core values of the Bank. It exhibits high standards of business and professional conduct in managing and supervising affairs of the Bank and reporting to shareholders on its stewardship. The Board acts on a fully informed basis in the best interest of the Bank and its stakeholders and set 'tone at the top' in order to promote a sound corporate culture.

During the year, the overall performance evaluation of the Board was conducted by the Evaluation Committee of the Bank on basis of questionnaires broadly covered under below mentioned criteria:

a) Is the composition of the board appropriate, having the right mix of knowledge, expertise and skills to maximize performance?
b) How well the board exercises its role ensuring that the organization supports and upholds the vision and mission, core values etc.?
c) Is the policy framework of bank/DFI developed appropriately?
d) What has been the board's contribution towards developing strategies?
e) Is the board able to make timely strategic decisions ensuring operations are in line with strategies?
f) Is the information provided to the board appropriate, accurate, timely and unbiased?
g) What has been the board's contribution in ensuring

robust and effective risk management?

h) Has the board ensured that internal control and the audit function are conducted in an effective manner?
i) Has the board ensured timely and accurate disclosure on all material information?
j) Is the board as a whole, up-to-date with latest developments in the regulatory environment?
k) Are the board procedures conducive to effective performance and flexible enough to deal with all eventualities?
l) Is the board effective in adherence to the code of conduct?

Criteria for Annual Performance Evaluation of the Board Committees:

The rationale for the formation of Board Committees is to assist it in the performance of its functions and enhance the efficiency by sharing the work load of the Board. MCB Board has formed eight (8) Committees assisting the Board in the area of audit, strategy setting, risk management, human resource management, physical planning & contingency arrangements, information technology, compliance reviews and write-offs & waivers. The size, structure and skill set of the Board Committees are in line with the applicable rules, regulations and laws as well as business needs of the Bank.

The annual performance evaluation of the Board Committees was also performed by the Evaluation Committee by using in-house-approach on the basis of specific questions/criteria mentioned in the SBP Guidelines. The consolidated final ratings of the evaluation of the Board Committees were in line with the rating given by the PICG as an independent evaluator during the year 2018. The overall results showed that the Board Committees have well-defined objectives and have been properly structured with appropriate Terms of Reference and fulfilling their respective roles effectively and efficiently.

During the year, the performance evaluation of the Board Committees was conducted by the Evaluation Committee of the Bank on basis of questionnaires broadly covered under below mentioned criteria:

a) Are the size, structure and skill set of committees appropriate?
b) Does each committee have adequate and appropriate written terms of reference?
c) Are the committees effectively discharging their functions and duties as per terms of reference?
d) Is the frequency of committee meetings adequate?
e) Are the committee meetings organized properly with appropriate procedures?
f) Are the committee meetings conducted in a manner that encourages open communication and meaningful participation of its members?
g) How effectively and proactively committees have followed up with their areas of concern?
h) Are the suggestions and recommendations of committees effective?

Annual Performance Evaluation of the Chairman, Individual and Independent Directors:

As per the requirements and criteria given by the SBP Guidelines, the performance evaluation of the Chairman of the Board was performed by the Independent Directors. Similarly, the performance evaluation of Individual Directors was conducted by the Chairman; however, Independent Directors are evaluated by all other Directors of the Bank excluding the Director being evaluated. Based on the results of the performance evaluation of the Chairman, Independent and other Non-Executive Directors, it was found that the individual Directors were making valuable contributions with proper commitment to their respective roles and responsibilities.

Criteria for Annual Performance Evaluation of the President & CEO of the Bank:

The President & Chief Executive Officer ("CEO") of the Bank is vested with the responsibility of managing overall affairs of the Bank. He through effective leadership and team building efforts achieved the maximum possible performance of the Bank and managed the affairs of the Bank in accordance with strategic and long term objectives of the Bank. He ensured that the Bank's resources and budgetary targets are aligned with the strategic plan of the Bank and the best industry practices. He promoted the highest standards of integrity, probity and good governance within the Bank. He showed his commitment to stakeholders of the Bank in defining strategic planning with great emphasis on service quality, investment in network infrastructure and human capital, fortifying compliance & risk standards and also remained steadfast in maintaining Bank's fortress balance sheet. The Senior Management under his leadership devised its goals in line with the strategic direction of the Bank.

During the year, the performance evaluation of the President & CEO was conducted by all the Directors of the Bank on basis of below mentioned criteria:

a) Were the financial/business targets set by the board achieved?
b) Does he possess leadership qualities i.e. correct anticipation of business trends, opportunities and priorities affecting the institution's prosperity and operations?
c) Has he developed clear mission statement, policies, and strategic plans that harmoniously balance the needs of all the stakeholders?
d) Does he ensure that company's resources and budgets are aligned with the implementation of the organization's strategic plan?
e) Does he establish an effective organization structure to ensure management's focus on key functions?
f) Does he timely and effectively execute strategies set by the board?
g) Has he served as an effective representative while communicating with all the stakeholders?

Directors' Remuneration:

Remuneration is considered to be very important for overall success of the Bank as it helps to attract high quality professionals to the Bank. The Board has determined such level of remuneration which is appropriate and commensurate with the level of responsibility and expertise offered by the members of the Board of Directors and aimed at attracting and retaining members needed to govern the Bank successfully, and creating value addition. The Board has determined the scale of remuneration including additional remuneration to be paid to the Non-Executive Directors including Independent Directors and the Chairman for attending Board Meetings and its Committees meetings of the Bank. The scale of remuneration including additional remuneration is within the prescribed limits of the State Bank of Pakistan and it has been duly approved by the Shareholders of the Bank.

Directors' Remuneration Policy:

The Board has framed and approved a comprehensive and transparent remuneration policy "the Policy" for the Chairman and other Directors. The Policy aims to set out the requirements and methodology for the determination of the scale of the remuneration to be paid, to the Chairman and other Directors for attending the Board and its Committees meetings during a financial year. The Policy ensures that the Board Members are fairly rewarded with regard to their respective responsibilities undertaken, and also to attract and retain high-caliber, experienced directors by offering appropriate remuneration levels commensurate with their expertise, skill and experience. It has been formulated with clear mandate and charter, keeping in view the ownership structure, governance mechanism, risk profile, scope of operations and performance of the Bank. The Policy is applicable to the Non-Executive/Independent Directors, Executive Director(s) and the Chairman of the Board of Directors. It has been prepared in accordance with the provisions of applicable laws, rules and regulations as amended from time to time.

The Bank being financial services provider always adheres to practicing good governance, enabling to enhance its efficiency and footprints in financial sector. MCB's Board is comprised of directors who have diversified experience, suitable knowledge, appropriate skill set/expertise and competencies considered relevant in the context of the Bank's operations and to make the Board an effective oversight and decision making body.

Payment of Directors' Remuneration:

The detail of remuneration paid to the Executive Director and the Non-Executive Directors during the year 2020 has been disclosed in the Note No. 40 of the Unconsolidated Financial Statements of the Bank for the year ended December 31, 2020.

Directors' Orientation:

Directors' orientation enables the directors to have better understanding of specific context under which directors operate and comprehend their duties and responsibilities and also to acquaint them with wider scope of the responsibilities, propagate due diligence and acting in good faith while effectively managing the interests of the Bank. It continues through the orientation stage and leads to ongoing directors' education as well as directors' and Board's assessment. An orientation program is aimed at increasing director's familiarity with the Bank, its industry as well as equipping the directors with sufficient information and resources that facilitate fully-informed decisions.

In this connection, the Board Members are regularly provided with update on new applicable laws, rules and regulations including amendments thereto to apprise them with their powers, duties and responsibilities. At the time of induction of new director(s), he/ she is given orientation about the Bank's corporate governance framework, its businesses, current issues, strategies and operations by the Management to acquaint them with the Bank's overall operations in order to enable them to effectively govern the affairs of the Bank on behalf of shareholders. The directors are also provided with the detailed written material in shape of extracts from relevant laws, rules & regulations on powers, duties & responsibilities of the Board of Directors.

Policy of retention of fee / remuneration from Nomination on the Board of other companies:

The Directors have approved the Nomination Policy whereby Bank's Executives are nominated on the Board of other Companies on behalf of the Bank. Nominee Directors who are employees of the Bank shall, however, have to surrender compensation such as meeting attendance fee to the Bank.

Directors' Training Program:

All the Board Members except one, either have minimum education and experience required for exemption from Directors Training Program ("DTP") or have already completed such training as narrated in the Listed Companies (Code of Corporate Governance) Regulations, 2019 (the "Regulations") and the Prudential Regulations issued by SBP.

MCB Board is fully adhered to directors training arrangements under the Regulations. Further, the Bank is also well ahead of the prescribed deadlines of DTP requirements as given by the regulator.

Board's Function and Decision Making:

MCB Board plays an effective role and provides entrepreneurial leadership and direction for the Management of the Bank within a framework of prudent and effective controls. It promotes collective vision

of the Bank purpose, its culture, its values and also demonstrates ethical leadership. MCB Board comprises of local as well as foreign directors who have diversified experience, suitable knowledge, appropriate skills/ expertise and competency considered relevant in the context of the Bank's operations and to make the Board an effective decision making body. The collective wisdom of the Board is translated into its decisions which form the basis for Management to achieve its targets.

The primary role of the Board of Directors of the Bank is to enhance shareholder value. The directions provided by the Board enable the Senior Management to deliver remarkable returns to stakeholders, sustainable performance and value added services. It also helps in building a corporate culture of equality, trust and team spirit. MCB Board is concerned with strategic matters and overseeing the business of the Bank in light of emerging risks and opportunities, on a regular basis and also involved in establishing and reviewing the strategies, yearly targets and financial objectives of the Bank.

Significant Issues/Matters discussed/approved by the Board of Directors:

During the year 2020, the Board of Directors deliberated/ approved the following Significant Issues/ Matters:

- Annual Budget for the year 2021;
- Strategic Business Plans for MCB Sri Lanka & MCB UAE – 2020-2022;
- Bank's Policies including periodic reviews and amendments thereto;
- Periodical review of Terms of Reference ("TORs") of Board's Committees;
- Financial Results of MCB Bank and consolidated Financial Results with its subsidiaries on quarterly, half-yearly and annual basis together with Directors' Report, Auditors' Report and Chairman's Review Report;
- Declaration of Interim and Final Cash Dividends;
- Related Party Transactions as recommended by Board's Audit Committee;
- Management Letter(s) issued by the External Auditors of the Bank and its compliance status;
- Update on Biometric Verification Project;
- Approval of Action Plan relating to Data Cleansing Exercise;
- Review of Institutional Risk Assessment Framework ("IRAF") Questionnaire;
- Significant activities and achievements of Board's Committees;
- Complaints Received under Whistle Blowing Program and Action taken thereon;
- Matters recommended by Board's Committees;
- Appointment/Engagement of External Auditors of the Bank as well as for Bank's Overseas Operations and also to Perform Agreed upon Procedures as recommended by the Audit Committee;
- Exception to Board's approved policies along with MIS;
- Exception from Current and Linkage Ratio Policy Framework;
- SBP Inspection Reports along with Time-Bound Action Plan thereon;
- Performance Evaluation of the Board as a whole, its Committees and Individual Directors;
- Matters pertaining to subsidiaries;
- Regulations and Standards by Central Bank of the UAE;
- Quarterly and Annual Reports on Fraud & Forgery Cases;
- Fraud Risk Assessment;
- Consumer Lending Business and Wealth Management Business;
- Write-offs/Waivers approved at Different Authority Levels and recoveries thereto;
- Status and implications of all material law suits filed by and against the Bank;
- Disposal of MCB Financial Services Limited, a wholly owned subsidiary of the Bank;
- Donations for Charitable Institutions under CSR activities of the Bank;
- Nominations of Members of the Board and Management on the Board of subsidiaries and other companies;
- Shares transferred of the Bank during the year 2020;
- Annual Branch Expansion Plan of the Bank; and
- Update on Laws, Rules and Regulations.

Matters Delegated to the Management:

The Board sets the strategic objectives and takes the overall responsibility of overseeing its implementation. The Board performs its duties by giving guidelines to the Management, setting performance targets and monitoring their achievements. The Management is primarily responsible for implementing the strategies as approved by the Board of Directors in conducting the operations of the Bank effectively. Tactical and operational matters are delegated to the Management. Further, under the direction and oversight of the President & CEO of the Bank, the Senior Management carries out and manages the Bank's activities in a manner consistent with the strategies, business goals, risk appetite, incentive compensation and other policies approved by the Board of Directors.

Governance Practices Exceeding Legal Requirements:

MCB Board as a whole respects the country laws and ensures meticulous compliance of applicable laws, rules & regulations and being a leading Bank always adheres to provide information and disclosures above the minimum regulatory requirements. The Board never gives the room for any sort of non-compliance and takes it as reputational risk for the Bank. The Management also regularly updates the Board with the latest development in the regulatory

environment and maintains stringent control over regulatory compliance through designated resources. Following are some of the practices of the Bank which exceed the minimum legal requirements:

- The Board has formed its eight sub-Committees as against legal requirement of four.
- The Bank has only one Executive Director (President & CEO) though permitted two executive directors by SBP and four (one third of the Board as executive directors) under the Listed Companies (Code of Corporate Governance) Regulations, 2019.
- Voluntary adoption of best reporting practices as prescribed by ICAP/ICMAP/SAFA with a view to making the Bank's financials more transparent.
- The Bank reports additional information in the Annual Report for stakeholders which is not required by any laws.

Roles and Responsibilities of the Chairman and the President & CEO of the Bank:

The Roles and Responsibilities of the Chairman and the President & CEO of the Bank are described below:

Roles and Responsibilities of the Chairman:

The Chairman of the Board of Directors ("the Board") shall be elected from amongst the non-executive directors of the Bank and shall not hold the office of Chief Executive Officer ("CEO"). He shall be responsible for leadership of the Board and shall ensure that the Board plays an effective role in fulfilling its responsibilities. The Chairman has responsibilities and powers as vested in him/her by law, Articles of Association of the Bank and/or assigned, from time to time, by the Board. In particular, the Chairman will coordinate the affairs of the Board and chair the meetings of the Bank; however, he shall not participate in day-to-day management affairs of the Bank.

The Chairman shall ensure that:

- The composition of the Board is in accordance with legal and regulatory requirements;
- The Board as a whole is functioning effectively in accordance with applicable laws, regulations and rules to inculcate sound business principles and prudent commercial practices;
- The Board receives appropriate, accurate, timely and unbiased information, in particular, about the Bank's affairs and performance to enable the Board to take sound and effective decisions;
- The meetings of the Board and the Shareholders of the Bank are convened in compliance with legal and regulatory requirements; and proceedings of such meetings are accurately and fairly recorded. The agenda of the meetings take full account of applicable laws & regulations and the requirements of Bank's business;
- All Board Members are encouraged to participate and raise issues and concerns in the Board discussions, whilst promoting highest standards of Corporate Governance;
- The Board is concentrating on relevant issues and conflicts (if any) are effectively resolved;
- The Board sets the tone and values of the Bank; promotes a culture of openness and constructive debate and effective decision making;
- The Board is periodically updated on its statutory and fiduciary duties, as required in relevant laws, regulations and rules, enabling the Directors to perform their roles & responsibilities properly and prudently in the best interest of the Bank;
- Good relationship is maintained with Board Members, the Management and the Shareholders; so that obligations to the Shareholders and other stakeholders are understood and met; and
- Shareholders' and other stakeholders' interest is promoted in the decisions taken by the Board.

The Chairman and the Vice-Chairman are elected by the Board in accordance with the provisions of the Articles of Association of the Bank and hold the office for a period of three years. In the absence of the Chairman, the above mentioned roles and responsibilities of the Chairman are performed by the Vice-Chairman of the Bank.

Roles and Responsibilities of the President & CEO:

The President & CEO of the Bank, subject to the control and directions of Board, are entrusted with the whole, or substantially the whole, of the power of the Management to direct, manage, administer and control the affairs of the Bank. He shall be responsible to the Board for the implementation of its strategies, policies and decisions. The terms and conditions of appointment of the President & CEO are determined by the Board of Directors. The President is to be deemed a director and also entitled to all the rights and privileges and be subject to all the liabilities of that office. He/she possess leadership qualities i.e. correct anticipation of business trends, opportunities and priorities affecting the institution's prosperity and operations.

The President & CEO of the Bank shall:

- Set the appropriate performance standards to achieve financial/business targets set by the Board;
- Ensure that Bank's resources and budgets are aligned with the implementation of its strategic plan;
- Ensure assessment, monitoring and effective management of the significant risks to the Bank;
- Ensure that Bank maintains high standards of corporate citizenship and social responsibility wherever it operates;
- Establish an effective organizational structure having appropriate resources/systems within the Bank, to ensure Management's focus on key functions;
- Timely and effectively execute strategies set by the Board;

- Manage the affairs of the Bank in accordance with strategies and long term objectives approved by the Board;
- Ensure effective communication with the Board, Shareholders, Employees, regulatory authorities and other stakeholders and serve as an effective representative of the Bank while communicating with all the stakeholders;
- Ensure through effective leadership, team building and motivation that the maximum possible performance is achieved by the Bank and ensure that the affairs of the Bank are being managed in accordance with highest ethical standards, sound business principles and prudent commercial practices;
- Exercise the overall control, discretion, administration and supervision for the sound and efficient management and conduct of the business of the Bank;
- Monitor short term goals and ensure that the operating groups/divisions develop their own plans for the future, which need to be quantified as far as possible with benchmarks established;
- Conduct a periodic performance review of the Senior Management team so that major initiatives such as expansion strategies, acquisitions and capital investments should be finalized and adopted through major marketing and development exercises;
- Provide the Board with the relevant information it needs to carry out its fiduciary responsibilities and to supervise the Senior Management;
- Liaise between the Board and the staff, and communicate on a regular basis with both to promote understanding, cohesiveness and coordination for development of policies and their implementation;
- Ensure the compliance of applicable laws, rules and regulations;
- Ensure establishment of an effective information mechanism whereby internal and/or external significant/material items affecting Bank's affairs are identified and shared with relevant stakeholders on timely basis; and
- Maintain follow up on regulators' observations and other lawful instructions and issues raised by external and/or internal auditors and to ensure their strict adherence/compliance in Bank's operations.

Code of Conduct & Ethical Standards for Directors:

The Bank has also developed "Code of Conduct & Ethical Standards for Directors" as per the requirements of Code of Corporate Governance which is signed annually by every Director of the Bank.

Directors' Profile:

Directors' profile has been incorporated in the "Board of Directors" section.

Accessibility of Annual Report-2020:

Annual Report-2020 and other information of the Bank are accessible on Bank's Website: www.mcb.com.pk

Security Clearance of Foreign Directors:

Foreign Directors elected on the Board of Bank requires security clearance from Ministry of Interior through SECP. All legal formalities and requirements have been met and fulfilled in this regard.

Board's Policy on Diversity:

The Board of Directors firmly believes that the diverse mix of gender, knowledge, expertise and skill sets of the members / employees enhances the effectiveness of the Bank.

MCB is committed in fostering, cultivating and preserving a culture of diversity and inclusion. Human capital is the most valuable asset the Bank has. The collective sum of the individual's life experiences, knowledge, inventiveness, innovation, self-expression, unique capabilities and talent that employees invest in their work, represents a significant part of Bank's culture, as well as reputation and achievement.

The Bank embraces and encourages employees' with a mix of age, physical disability, family ethnicity, language, political affiliation, religion, sexual orientation, socioeconomic status and other characteristics that make its employees unique.

Related Parties and approved Policy for Related Party Transactions

The Board of Directors has approved a Policy for Related Party Transactions. The Bank's policy is to conduct all the related party transactions on an arm's length basis in the in the normal course of business. If a transaction is not conducted on arm's length basis then specific approvals or ratifications is required by the Board on recommendation of the Audit Committee of the Bank in order to avoid any potential conflict of interest.

The policy specifies that all transactions entered into with related parties shall require Board's approval on the recommendation of the Board Audit Committee of the Bank, which is chaired by an independent director of the Bank.

Every director (including spouse, children, step children and parents) of the Bank who is in any way, whether directly or indirectly, concerned or interested in any contract or arrangement entered into, or to be entered into, by or on behalf of the Bank shall disclose the nature of his concern or interest at a meeting of the board. No director of the Bank shall, as a director, take any part in the discussion of, or vote on, any contract or arrangement entered into, or to be entered into, by or on behalf of the Bank, if he/she is in any way, whether directly or indirectly, concerned or interested in the contract or arrangement, nor shall his/

her presence count for the purpose of forming a quorum at the time of any such discussion or vote.

During the year, the Bank has entered into transactions and contracts with the related parties i.e. subsidiary and associate companies, post-employment benefit plans for the Bank's employees, Key Management Personnel (KMPs), Close Family Members (CFMs) of KMPs and other related entities. Those transactions include lending activities, acceptance and placements, off balance sheet transactions and provision of other banking and financial services that are carried out in the ordinary course of business on an arm's length basis at commercial rates, except for the transactions that KMPs have availed under HR policy of the Bank. Contributions to and accruals in respect of staff retirement benefits and other benefit plans are made in accordance with the actuarial valuations / terms of the contribution plan. Remuneration to the executives / officers is determined in accordance with the terms of their appointment.

The Bank has made detailed disclosures about related party transactions in its financial statements annexed with this annual report.

During the year, no contract or arrangement entered with related parties other than in the ordinary course of business on an arm's length basis.

Managing Conflict of Interest:

Overview:

A conflict of interest arises when a director, directly or indirectly, has an interest, pecuniary or otherwise, in performing his functions or duties and such interest could lead to impair his ability to consider and decide any matter impartially, without creating biasness in his or her own decision.

The Board Members owe certain fiduciary duties, including the duties of loyalty, due diligence, and confidentiality to the Bank which require that a director must act in good faith in order to promote the objectives of the Bank in the best interests of the Bank itself and its shareholders, employees and community as a whole. In this regard, the Bank had developed "Code of Conduct & Ethical Standards for Directors" as per requirements of Code of Corporate Governance which required that every director of the Bank has to disclose potential or actual conflicts of interest with respect to his/her duties as soon as they arise or he/she becomes aware of them. All the Board Members took reasonable steps to avoid being in an actual, apparent or potential conflict of interest. The Board recognizes the responsibility to adhere to the defined policies of the Bank and avoid perceived conflicts of interest that may arise during the course of business.

Disclosure of Interest by Director:

Every director (including spouse, children, step children and parents) of the Bank who is in any way, whether directly or indirectly, concerned or interested in any contract or arrangement entered into, or to be entered into, by or on behalf of the Bank shall disclose the nature of his concern or interest at a meeting of the board. No director of the Bank shall, as a director, take any part in the discussion of, or vote on, any contract or arrangement entered into, or to be entered into, by or on behalf of the Bank, if he/she is in any way, whether directly or indirectly, concerned or interested in the contract or arrangement, nor shall his/her presence count for the purpose of forming a quorum at the time of any such discussion or vote. As per the Code of Corporate Governance, the Bank has developed the mechanism to place the matter before the general meeting for its approval where majority of the directors are interested in any transaction or event.

Disclosure of Interest by Officers:

No other Officer of the Bank who is in any way, directly or indirectly, concerned or interested in any proposed contract or arrangement with the Bank shall, unless he discloses the nature and extent of his/her interest in the transaction and obtains the prior approval of the Board of Directors, enter into any such contract or arrangement.

Exposure in Companies where Directors are Interested:

The Bank may take exposure in the companies in which directors (including their spouses, parents, and children) hold key management positions, or are interested as partner, director or guarantor, or shareholders holding 5% or more of the share capital of that concern, with the approval of the majority of the directors excluding the director concerned. The financing facilities shall be extended at market terms and conditions and be dealt with on arm's length basis.

The Bank shall not enter into leasing, renting and sale/purchase of any kind with their directors, officers, employees or such persons who either individually or in concert with family members beneficially own 5% or more of the equity of the Bank.

The Bank shall not take unsecured exposure on, or take exposure against the guarantee of any of its directors, any of the family members of any of directors and any firm or private company in which our directors are interested as director, proprietor and partner or public company in which such persons are substantially interested.

Conflicts of Interest Register:

The Bank maintains a register of all contracts, arrangements or appointments in which directors are interested.

Insider Trading:

Directors being the insiders are prohibited by the law to indulge in insider trading. They shall not deal directly or indirectly in the securities of the Bank whether on their own account or their relative's account, if they are in possession of any unpublished price sensitive/inside

information, which if published or known, is likely to materially affect the price of Bank's securities. Directors are usually considered to have such information, shall not communicate directly or indirectly the said information to others who might exploit such information while trading in the securities of the Bank.

As per the regulatory requirements relating to Insiders' trading, the Bank is maintaining the register of Insiders who have access to unpublished price sensitive/inside information and the said Register regularly updated by the authorized personnel of the Senior Management of the Bank.

Whenever, the Bank or a person acting on its behalf, discloses any inside information to any third party in the normal exercise of employment, profession or duty; a complete and effective public disclosure of that information is made simultaneously unless such person owes a duty of confidentiality. Further, Bank has approved policy on Prohibition of Insider Trading which is effectively implemented throughout the Bank.

Investor Grievances

MCB Bank ensures safeguarding the interests of its stakeholders by effective communication at regular intervals through multiple mediums. However, the Bank acknowledges that there may be instances where the stakeholders may have unaddressed concerns which if unresolved may become a grievance. To timely address any untoward incident, the Bank has a well-functioning grievance resolution mechanism that provides a transparent and credible process resulting in outcomes that are seen as impartial, effective, and durable. Through this initiative the Bank is able to reduce investment risks, provide an effective avenue to express and resolve concerns, thereby substantiating positive relationship. The Bank ensures quality services with uncompromising focus on investors' concerns and transparency in execution thereby extending respect to the trust placed.

The Bank is rigorously following the complaints of investors received from any regulatory forum and designate exclusive resources to resolve the matter both effectively and efficiently. Further, a centralized function namely the Shares department in the Corporate Affairs Division striving hard to manage any such investor grievances in consultation with the share registrar. Investors can lodge complaints by contacting the shares registrar, or write a letter or send an email to the share department of the Bank. A designated e-mail address, (investor.relations@mcb.com.pk) has been created to timely address the same and is readily available through our website and annual reports. The Bank ensures resolution of any grievances within statutory timelines.

Human resource management policies and succession planning

Human Resource Policies" have been approved by the Board of Directors of the Bank on the recommendation of Human Resource and Remuneration Committee of Board in order to provide clear and definitive directions on human resource (HR) related matters.

MCB being an equal opportunity employer is committed to create a congenial and efficient work environment in which the employees are assured a non-discriminatory, transparent, harassment free and respectful atmosphere regardless of their cast, creed, religion and gender. The Bank talent-acquisition policies provide unbiased criteria for hiring people through lateral and batch hiring, from any background as long as they qualify for the professional criteria required by the Bank. The culture at MCB Bank also depicts a healthy, team based and cooperative environment. We value the unique talents and perspectives of our employees and strive to create a respectful workplace.

The Bank is committed towards employee development practices which enable all its employees to reach their optimum potential, thereby creating a high performance organization. This belief is supported by the Bank's comprehensive approach towards performance management, career development and management training. The Bank affirms its belief in motivating its work force through positive reinforcement and opportunities in each of these key areas regarding employee development. The Bank has a transparent KPI based performance assessment and reward mechanism that allows front office employees to track their performance with their goal achievement throughout the year.

Salient feature of Staff Compensation/Remuneration Policy

The Remuneration Policy of Bank is designed to promote a culture of sound compensation aligned with risk and responsibilities in a transparent manner for acquisition of talent, retention of employees and achievement of stakeholders expectations. MCB's remuneration policy applies to all staff. The policy covers identification of Material Risk Taker (MRT) and Material Risk Controllers (MRC), performance assessment through balanced scorecards, compensation structure and deferral mechanism.

The responsibility for approving the remuneration policy rests with the Board of Directors. The Board has constituted Human Resource & Remuneration Committee (HR&RC) for recommending to the Board, the structure of the remuneration policy, including the remuneration setting mechanisms, structures, composition of remuneration, and other related matters. HR & RC may take the support of Bank's functions (e.g. Finance, Risk, Audit, Compliance, and HR). At management level, HRMG leads the overall remuneration policy of MCB.

All compensation provided to MCB staff can be divided into fixed or variable remuneration. Fixed remuneration is that part of the compensation which remains unffected by the performance of the Bank or individual employee. Fixed component of remuneration consists of basic salary

and allowances that are part of the total compensation package of the employee. Variable remuneration is the part of total compensation package of an employee which is linked with some predetermined measures of performance. Variable compensation is linked with the individual's performance and comprises of performance bonus, commissions, incentives and allowances.

The Bank has identified functions and designations as MRTs/MRCs. These include, President/CEO, direct reportee to the President/CEO (members of management committee), Country heads of overseas branches, direct reportee to the members of senior management managing critical functions as determined by HR&RC and all other material Business units.

Balanced scorecards are defined for all MRTs & MRCs for carrying out an objective and transparent performance assessment. The variable compensation for the MRTs and MRCs is linked to the performance result derived from the scorecard. The performance assessment for MRTs and MRCs is performed via the structured balanced scorecard mechanism that is in place to ensure that objective risk and return measures are duly taken into account for determining the bonuses and awards for MRTs and MRCs. The bonuses and awards for MRTs are determined based on the performance of the individual, their respective department and the overall Bank.

MRCs in the Bank have suitable autonomy and authority to perform their tasks independently, without influence from the functions they are assigned to oversee and review. KPIs in the scorecards of MRCs are independent of the KPIs of the business functions that they oversee. This ensures that achievement of financial targets of the business functions are not considered for the performance assessment of the MRCs.

For MCB employees classified as MRTs and MRCs, at least 25% of their variable remuneration shall be deferred. Minimum deferral period is three years with no vesting prior to year 1. Deferred variable remuneration shall be paid.

Policy for Sustainability and Corporate Social Responsibility (CSR)

Policy Sustainability and Corporate Social Responsibility (CSR) is detailed in Sustainability and Corporate Social Responsibility Section of this report.

Responsibilities of Management and the Board of Directors toward the preparation and presentation of the financial statements

The Management is aware of its responsibility for the preparation and fair presentation of the financial statements in accordance with accounting and reporting standards as applicable in Pakistan, the requirements of Banking Companies Ordinance, 1962 and the Companies Act, 2017 (XIX of 2017) and for such internal control as management determines is necessary to enable the preparation of financial statements that are free from material misstatement, whether due to fraud or error.

The Board of directors is responsible for overseeing the Bank’s financial reporting process.

Policy for Safety of Records of the Bank:

Record management is a methodological approach to control the maintenance and disposition of organization’s record. Record management ensures that valuable record evidencing an organization’s activities that have legal, financial, administrative or historical value are protected and accessible while expired record is systematically destroyed. Thus MCB Bank has put in place comprehensive processes, controls and guidelines on handling, protection, retention, retrieval, and disposition of recorded business information generated daily which are of ongoing importance to MCB’s overall service capability and regulatory compliance. In its endeavor to comply these guidelines / processes, bank has already achieved major milestones.

Social and Environmental Responsibility

MCB Bank Limited undertakes its responsibility to be recognized as an organization that is aware of both its social and environmental obligation. The Bank continuously strives to inculcate the same by creating awareness amongst stakeholders, streamlining its operational processes and reinforcing the same through various policies. The key areas that the Bank focuses upon are to provide a safe and healthy workplace, protect the environment and conserve energy through use of appropriate technology and management practices.

Some of the salient features of the Bank policy are as follows:

- Compliance with local, national and international laws and regulations as well as the spirit thereof and conduct of business operations with honesty and integrity
- Promote and engage in social welfare activities that help strengthen communities and contribute to the enrichment of society
- Provide innovative, safe and outstanding high quality banking products and services exceeding the expectations of customers
- Significant investment to develop technological based ‘Alternative Delivery Channels’ for maximum ‘Financial inclusion’
- Sustainable development through building and maintaining sound relationships with our stakeholders through open and fair communication
- Communication and dialogue with employees, to build and share the value of “Mutual Trust and Mutual Responsibility” and work together for the success of our all stakeholders
- Respect for people by honoring the culture, customs,

history and laws of Pakistan. Constantly search for safer, cleaner and superior practices that satisfy the evolving needs of the society
- Minimize the environmental impact of business operations, by working to reduce the wastage of all resources
- Develop, establish and promote practices enabling the environment and economy to coexist harmoniously and build close and cooperative relationships with individuals and organizations involved in environmental preservation

Review by the Board of the Bank's Business Continuity Plan

Business Continuity Management (BCM) is a process that identifies and recognizes risks, threats and vulnerabilities that could impact Bank's operations internally or externally. BCM provides framework and creates ability for an organization to mitigate risk, withstand changes in the environment and still perform its critical processes and functions.

A robust Business Continuity Plan (BCP) outlines a range of disaster scenarios and the steps to be taken in order to minimize the potential harm to business during adverse situations. BCP's/system recovery procedures are written ahead of time therefore Bank endeavors to have sustainable, effective enterprise wide BCM program to provide seamless services and product reach to its customers/ stakeholders.

The Board of Directors periodically reviews and gauges Banks preparedness to deal with any untoward situation. Furthermore the senior management of the Bank ensures to maintain an effective Business Continuity Policy & Framework to ascertain that clear and concise plans are maintained for all critical areas which encompasses strong remedial actions to reduce the risk of downtime in any contingency scenario. In these difficult times when the world was taken aback by COVID-19 and every segment of life got affected the Bank adopted a proactive approach and immediately arranged for the Personal Protective Equipment's (PPEs) comprising of face masks, hand gloves, face shields, hand sanitizers etc. for its staff and visiting customers. Furthermore special screening arrangement, maintaining social distance, minimizing staff strength, periodic disinfection in branches and offices were the initiatives taken to ensure continuity of business and services, thus complying with Government and WHO precautionary instructions.

Further to nurture and enhance the confidence on Bank's system and processes; Business Continuity Plans are tested and updated on regular intervals. Since BCM is well knitted into the Banks structure and branch network, thus it gives confidence to its millions of customers, stakeholders and regulator that the Bank can live up-to the commitment and expectations by ensuring continued functionality of its critical businesses and functions in any circumstances.

Stakeholders' engagement

Stakeholders' engagement and the steps taken to solicit and understand the views of the shareholders is detailed in stakeholders' engagement section of this report.

Whistle Blowing Program

Overview

MCB Bank is committed to continually operate at the highest standards of conduct in our business. We are the trustees of public funds and it is our core value to serve our community with integrity. We endeavor to earn and uphold the trust of all our customers and stakeholders by serving and dealing with them lawfully, ethically and professionally.

Purpose

This program provides a channel to Bank's staff and outside parties such as shareholders, vendors, customers etc. for raising concerns/complaints about irregularities, impropriety, financial malpractices, frauds & forgeries, personnel harassment and improper conduct or wrongdoing without any fear of reprisal or adverse consequences. The objective of the program is to address/resolve these concerns/complaints to prevent and/or detect improper activities for safeguarding the interest and reputation of the Bank and its stakeholders.

Scope

The program covers deliberate, voluntary disclosure of individual or organizational impropriety by a person who has or had privileged access to data, information or event about an actual, suspected or anticipated wrongdoing within or by an organization that is within its ability to control.

Protection of Whistle Blowers

MCB Bank is committed to the protection of genuine complainants against action(s) taken in reprisal for the making of protected disclosures. Confidentiality of the complainant's identity, the nature of the report and the identity of the suspected person is strictly maintained.

The Bank does not tolerate harassment or victimization and takes action, which could involve disciplinary proceedings, to protect complainants when they raise a concern in good faith.

Incentives for Whistle Blowing

Complainant may be awarded monetary benefit/career advancement depending upon the nature and gravity of the concerns/complaints.

Whistle Blowing Mechanism

Employees or outside parties with concerns or complaints may report such concerns or complaints to Whistle Blowing Unit through any of the following means:

- Landline
- E-mail
- Fax
- Website
- Regular Mail

Concerns and complaints are investigated and findings are shared with the senior management for their necessary action. Information related to investigations is also shared with the Audit Committee and Board of Directors.

Number of instances reported to Audit Committee

Number of whistle blowing incidents (wrongdoings) along with update on investigations/resolution reported to the Audit Committee and Board of Directors during the year 2020 was 13.

Significant changes from prior years

- During the year three group heads (Key Management Personnel) retired/resigned from their positions and board approved their replacements accordingly.
- During the year, the Bank, in line with the shareholder approval, disposed of its ownership stake in the wholly owned subsidiary namely MCB Financial Services Limited.

Shares held by Directors, Chief Executive Officer, their Spouses and Minor Children

Shares held by Directors, Chief Executive Officer, their Spouses and Minor Children have been disclosed in "Categories of Shareholders" section of the Annual Report.

Presence of Chairman of Audit Committee at the AGM

The Chairman of the Board Audit Committee was present at the AGM to answer questions on the Audit Committee's activities and matters within the scope of the Audit Committee's responsibilities.

Chairman's significant commitments

Mian Mohammad Mansha has served as Chairman of MCB Bank, after its privatization, from 1991 to mid-1995 and then from 1997 till date. Presently, he is also a member of the Board's Human Resource & Remuneration Committee, Committee on Physical Planning & Contingency Arrangements and Business Strategy & Development Committee at MCB Bank Limited. He does not have any significant commitment other than the one mentioned in his profile under "Directors Profile" section of the Annual Report.

Pandemic Recovery Plan by the Management and Policy Statement

The emergence of COVID - 19 pandemic during the year inflicted an unprecedented global crisis; taking a large toll on human lives, inducing a synchronized economic downturn and exerting long term socio-economic repercussions. The emanating uncertainty garnered emergency response from Governments' across the globe in the form of multiple policy measures implemented on the economic, fiscal and monetary fronts. The unprecedented COVID-19 crisis has brought about many challenges for the financial sector and regulator.

Responding to the challenge, MCB leveraged its robust "BCM Policy and Framework" to implement a comprehensive pandemic recovery plan encompassing guidelines by International and National authorities and a risk based focus to protect the staff from impact of pandemic illness and ensure information system uptime, data integrity, service availability and overall business resilience.

Pandemic impacted several aspects of the bank's operations such as shortage of staff, increase in use of ATMs, increase in volume of digital and call center services, delay in payments and increase in delinquencies etc.

During pandemic, the Bank ensured that strategies and processes are in place to enable business continuity to provide critical services while ensuring the health and safety of employees, customers, and vendors. Technology played a major role in business continuity.

1. Ensured the health and safety of employees, customers and vendors

During pandemic, the Bank implemented below measures to allow the continuity of operations with minimal contact between staff, customers, or suppliers for prolonged periods of time.

1. Redirected customers from branch/office visits to online banking services. Encouraged customers to use bank's digital tools and other resources for self- service banking and 24/7 account access.
2. Reduce hours of operation and enabled employees to work from home or from alternative sites.
3. Used teleconference/videoconference for meetings (even for people in the same building).
4. Implemented restrictions on visitor access to the bank facilities and enforced quarantine for sick staff.
5. Installed sanitizing gel dispensers throughout all areas used by clients inside the branch and in internal areas for employees.
6. Rearranged furniture in order to allow more space between employees and between employees and clients or vendors.
7. Ensured the regular disinfection of sensitive areas and defined office hygiene procedures.
8. Regular temperature check was ensured at entry points of all locations and face masks made mandatory.
9. Strict protocols on social distancing were put in place.

2. Ensured continuity of the Bank's operations

With shortage of staff, decline in branch activities, and the reduction in human interaction in general, the use of technology played an extremely important role.

1. Identified which jobs and tasks can and cannot be done, even partially, without being physically present in the office.
2. Ensured employees know how to use the various hardware and software dedicated for remote working.
3. Ensured preparedness for a high absentee rate and make back-up arrangements for key staff and functions.
4. Cross-train employees and ensure that succession/replacement plans are in place.
5. Established procedures for safeguarding data, including backups.
6. Audited the technology used for remote access and check for the need of increasing capacity, bandwidth and authentication mechanisms.
7. Checked the capacity of networks to handle increase in traffic.
8. Ensured telecommunication structure can handle increase in call volume.

IT Governance

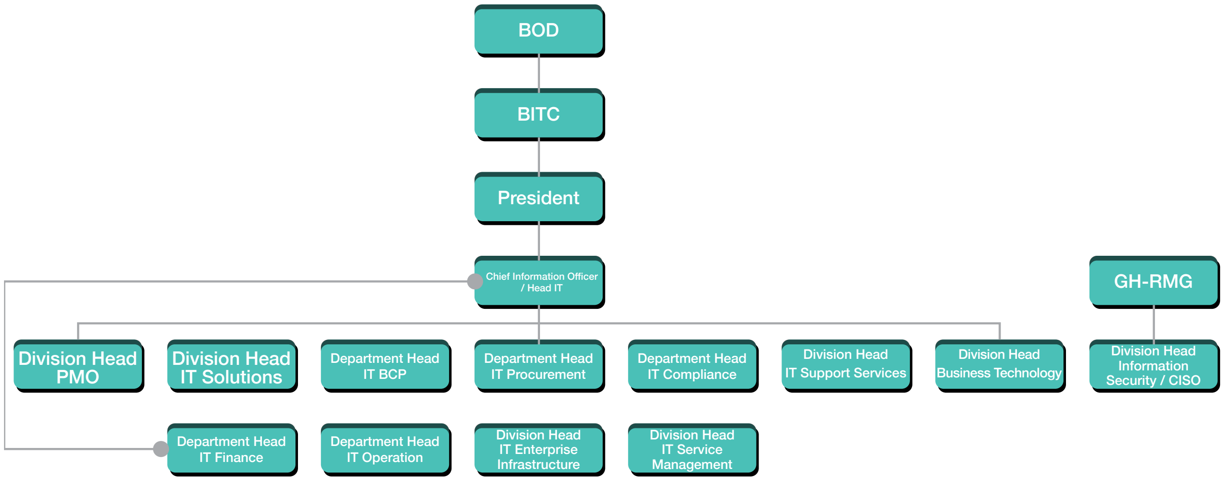

IT Governance is an integral part of Enterprise Governance and consists of the leadership and organizational structures and processes that ensure that the Bank's IT sustains and extends the Bank's strategies and objectives. IT Governance systematically engages the Board members, executive management and underlying staff. It establishes a discipline used by the organization to measure transparent accountability of decisions, and ensures the traceability of decisions to assigned responsibilities. Well-structured IT Governance would assist in creating efficiencies, enhance conformity to internationally accepted best practices, improve overall IT performance and also enable better control and security.

Information Technology Group is headed by CIO who in turn reports functionally and administratively to the President/CEO. The Office of the CIO provides the leadership for the development and delivery of world-class technology services.

The position is directly responsible for;

a) Managing the operations of Information and Technology Services for efficient and smooth delivery;
b) Recommending IT Strategy that is aligned with Bank's overall Strategy;
c) Encouraging technical innovation and the development of a robust and dependable technology infrastructure;
d) Strengthening the IT Governance;
e) Providing guidance, oversight, and strategic thinking on information technology;
f) Setting the overall direction for IT Group to introduce and implement innovative technology solutions;
g) Ensuring the availability of Bank's services as up & running and active DR invocation mechanism at the time of disaster.

Information Technology Group (ITG) has been taken care by teams of committed professionals, providing innovative and efficient solutions to achieve and nurture strategic objectives and goals of Business as well as other support groups under the guidance of Board IT Committee (BITC) and management IT Steering Committee (ITSC).

Group is further be strengthened by following functions:

a. IT Enterprise Infrastructure
b. IT Operations
c. IT Software Solutions
d. IT Service Management
e. IT Support Services
f. IT Business Technology
g. IT Project Management
h. IT Financial Services
i. IT Procurement
j. IT Compliances & Internal Control
k. IT Business Continuity

INTERNAL GOVERNANCE

The Bank's Management IT Steering Committee (ITSC) & Board IT Committee (BITC) are the governing bodies that review, monitor, prioritize and approve major IT projects. Key Objectives of these committees are:

- To provide a forum for discussions, review and advice on Technology needs, Investments, Issues & Progress;
- Prioritize, approve and monitor investments (projects & resource allocation), financial objectives and performance in order to review whether IT and Business strategies aligned with each other;
- Assessment of IT capability and adequacy of the IT infrastructure & Guidance on strategic goals and direction to see if enterprise achieving the optimum use of the IT resources;
- To review adoption of best practices, standardization and interoperability internally and externally;
- To provide resolution of cross-function or intercompany critical issues;
- Consideration of risk exposures and monitoring of risk management;
- To review the communication path between the board/executive and middle management.

Adoption and Statement of Adherence with the International Integrated Reporting Framework

In the course of its operations a commercial organization receives various inputs and converts them into value for itself and its stakeholders. This value creation can be over different time frames; short, medium and long. An integrated report describes this value creation process concisely including the business model, strategies, governance, processes, risks and opportunities.

Since MCB is one of the largest Commercial Bank and plays a vital role in the economy of the country. It also makes its presence felt at the grassroots level with its sprawling network of branches and its wide customer base. Therefore, it has an impact far beyond the bottom line. Being the Bank with one of the largest customer base it is imperative; therefore, that the Bank reassures its stakeholders that it is safeguarding the public interest.

Adoption of International Integrated Reporting Framework depends on the individual circumstances of an entity. The Bank has adopted the Integrated Reporting Framework to give an overview of Bank's philosophy to explain connection between its financial and non-financial information, which would enhance the user's understanding as to how the Bank is working to improve its performance keeping in view the stakeholder's interests. The business strategy information is linked directly to business activities and non-financial information, and provides explanations accordingly. Integrated framework is still considered to be a practice in its early stages. We will continue to improve the information produced to make it even easier to understand, while taking into account the opinion of stakeholders reading this report.

The Bank has included following content elements for the users of this report:

- Organizational overview and external environment
- Strategy and resource allocation
- Risks and opportunities
- Governance
- Performance and position
- Outlook
- Stakeholder's relationship and engagement
- Sustainability and corporate social responsibility
- Business Model
- Excellence in corporate reporting

The Bank's Annual Report 2020 covers the 12-month period from January 01, 2020 to December 31, 2020 and is consistent with our usual annual reporting cycle for financial and integrated reporting. The most recent previous report was dated December 31, 2019.

Profile of Shari'ah Advisor Board

Profile – MIB Shari'ah Board Members

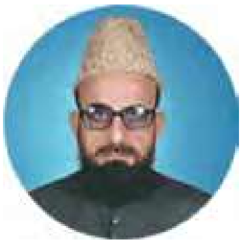

Prof. Mufti Munib-Ur-Rehman

Chairman Shari'ah Board

Prof. Mufti Munib-ur-Rehman, working with MCB Islamic Bank since September 2015, is a renowned Shari'ah scholar with a vast 48 years' teaching and 33 years' Fatawas issuance experience. He remained the member of Shari'ah Advisory Board of Securities and Exchange Commission of Pakistan (SECP) for three terms and the member of the Council of Islamic Ideology Pakistan (CIIP), he rendered voluntary services for the country since 2001 to 2020 as Chairman Central Moon Sighting Committee Pakistan. He served as Director of Islamic Studies, Hong Kong in 1985. He is the president of Tanzeem-ul-Madaris Ahle Sunnat Pakistan & Secretary General of Ittihad-e-Tanzeemat-e-Madaris Pakistan. He participated in international seminars in Saudi Arabia, UK, Norway, USA, Canada, Kazakhstan, Turkey and other Countries.

He remained member of the Board of Studies of University of Karachi, Federal Urdu University & Board of Intermediate Karachi. He remained member of the syndicate of University of Karachi & PMAS Arid University, Rawalpindi. He is the member of National Curriculum Pakistan & National Education Task Force. He was Shari'ah Adviser of Federal Shariat Court Pakistan. The 11 Volumes of his Fatwas has already published and has vast acceptability amongst Ulama. He is the Principle & Managing Trustee of Jamia Naeemia Karachi. He is Chairman Shari'ah Advisory Committee of Dawood Family Takaful Ltd and remained Chairman Shari'ah Board of Burj Bank Ltd for more than a decade.

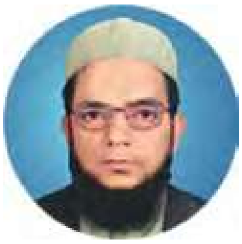

Mufti Syed Sabir Hussain

Resident Shari'ah Board Member/ Head-Shari'ah Compliance Department

Mufti Syed Sabir Hussain, working with MCB Islamic Bank Ltd. since September 2015, is a prominent Shari'ah Scholar and experienced Islamic Banker with 21 years of teaching, 14 years of Fatawa and Islamic banking experience. He holds Shahadat-Al-Alimiyah & Takhassus-Fil-Fiqh, M.A Islamic Studies, M.Phill in Islamic Banking & Finance and M.S (I.T) degrees. He is enrolled in Phd. on Islamic Banking and Finance from International Islamic University, Islamabad (IIUI).

He is ex-member of Shari'ah Advisory Board of SECP, Member of the Committee on Accounting and Auditing Standards of ICAP and invitee participant of Sahri'ah Advisory Committee of State Bank of Pakistan and Member of several committees constituted by SBP on AAOIFI Shari'ah standards. Further, he was member of review committee for Urdu translation of AAOIFI Shari'ah standards. He is author of 27 books on Islamic economics & banking and other social issues; He is also delivering lectures in different Dar-ul-Ulooms and Universities.

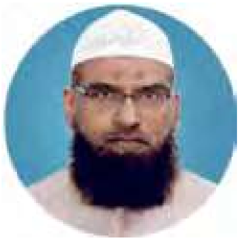

Mufti Nadeem Iqbal

Shari'ah Board Member

Mufti Nadeem Iqbal is the Shari'ah Board Member. He is senior teacher and Mufti at Dar-ul-Uloom Amjadia, Karachi and currently heading Dar-ul-Ifta. He has 29 years' experience of teaching Islamic Jurisprudence and 17 years' experience of issuing Fatawa (Shari'ah Opinions). He has 15 years' experience in Islamic Banking. He holds Master's Degree in Islamic Studies from University of Karachi, Takhusus-Fil-Fiqh from Dar-ul-Uloom Amjadia, Karachi, Fazil Dars-e-Nizami, and Fazil Shahada-tul-Almia. He gave his services as Resident Shari'ah Board Member/ Shari'ah Advisor at Soneri Bank's Islamic Banking Division for thirteen years. He is visiting faculty member at Sheikh Zayed Islamic Centre, University of Karachi and Hamdard University, Karachi. He is writer of several books including Islamic Jurisprudence.

Role of Shari’ah Board

i. The Shari’ah Board (SB) shall advise the BOD and the executive management of the MCB Islamic Bank Ltd. (MIB) on all Shari'ah related matters. All the SB’s Decisions/Rulings/Fatawas shall be binding on the MIB whereas the Shari'ah Board shall be responsible and accountable for all its Shari'ah decisions.

ii. The SB shall cause to develop a comprehensive Shari'ah compliance framework for all areas of operations of the MIB and shall approve all products/services to be offered and/or launched by the MIB.

iii. The SB shall ensure that all the MIB's products and services and related agreements/ contracts, structures, process flows, product manuals, marketing advertisements, sales illustrations and brochures etc. are in conformity with the rules and principles of Shari'ah. The executive management while seeking the SB’s decision on any proposal shall ensure provision of all the necessary information, details and documents enabling the SB to have adequate understanding of the product, its process flows, business and economic outcomes and Shari'ah permissibility or impermissibility.

iv. The Shari'ah Board shall have unhindered access to all records, documents and information from all sources including professional advisors and MIB's employees in discharge of its duties.

v. Considering the importance of the SB’s decisions and their binding nature, the SB shall rigorously deliberate on the proposals before giving any decision/fatwa; all such deliberations and rationale for allowing or disallowing a particular product/service etc. shall be duly recorded and documented.

vi. All the reports of internal/external Shari'ah audit and Shari'ah compliance reviews shall be submitted to the SB for prescribing appropriate enforcement action. SB shall take up the unresolved issues with the management and if warranted shall include the outstanding issues in their annual Shari'ah Board Report. Moreover, Head of SCD and RSBM shall discuss all the significant and unresolved issues with SBP inspection team during on-site inspection.

vii. The SB shall also specify the process/procedures for changing, modifying or revisiting Fatawas/Rulings/Guidelines etc. already issued by SB.

Meetings of Shari’ah Board held in 2020

S. No	Quarter	Date of Meeting	Meeting Attended (Yes / No)		
			Chairman	Member	RSBM
1	Q1	20-Mar-20	Yes	Yes	Yes
2	Q2	25-Jun-20	Yes	Yes	Yes
3	Q3	14-Sep-20	Yes	Yes	Yes
4	Q4	29-Dec-20	Yes	Yes	Yes

Meetings of Shari’ah Board-Board of Directors held in 2020

S. No	Half Year	Date of Meeting	Meeting Attended (Yes / No)		
			Chairman	Member	RSBM
1	1st	21-Apr-20	Yes	Yes	Yes
2	2nd	29-Dec-20	Yes	Yes	Yes

Membership on Shari’ah Board of other Companies

S. No	Name of Members	Date of Joining/ Leaving the Board	Status of Member-Chairman/ Resident member/ Non Resident Member	Number of Other Board Memberships along with name of companies
1	Mufti Munib-ur-Rehman	15-Sep-15	Chairman	1. Chairman Shari’ah Supervisory Board
2	Mufti Syed Sabir Hussain	16-Sep-15	Resident Member	1. Shari’ah Supervisory Board Member / Consultancy
3	Mufti Nadeem Iqbal	15-Oct-18	Non Resident Member	No other engagement

Report of Shari'ah Board

(For the Year ended December 31, 2020)

بِسْمِ اللهِ الرَّحْمٰنِ الرَّحِيْمِ

اَلْحَمْدُ لِلّٰهِ رَبِّ الْعَالَمِيْنَ وَالصَّلَاةُ وَالسَّلَامُ عَلٰى خَاتَمِ الْاَنْبِيَآءِ وَالْمُرْسَلِيْنَ وَعَلٰى اٰلِهٖ وَصَحْبِهٖ اَجْمَعِيْنَ اَمَّا بَعْدُ

The Shari'ah Board (SB) of MCB Islamic Bank Ltd. (MIB) was constituted in September, 2015. Currently Shari'ah Board comprises of respected Professor Mufti Munib-ur-Rehman as Chairman Shari'ah Board, Mufti Syed Sabir Hussain as Resident Shari'ah Board Member (RSBM) and Mufti Nadeem Iqbal as Member Shari'ah Board. Despite tough conditions of COVID – 19 all Shari'ah Board Members remain available throughout the year and many meetings were held through audio and video conferencing. In addition to that formal meeting of Shari'ah Board were held four times during the year 2020 on the following dates to review various matters which apart from other matters, include new products and services, product modifications, concepts, transactions, structures, processes and Shari'ah issues, referred to them:

First Shari'ah Board Meeting	–	March 20, 2020
Second Shari'ah Board Meeting	–	June 25, 2020
Third Shari'ah Board Meeting	–	September 14, 2020
Fourth Shari'ah Board Meeting	–	December 29, 2020

In order to assist the SB to supervise all these matters throughout the year, the Shari'ah Compliance Department ensured that, apart from the SB meetings, it closely coordinate with SB. This result in continuous involvement of SB on Shari'ah affairs of the Bank as well as enabled them to approve, by way of circulation, different matters in a timely manner.

1. While the Board of Directors and Executive Management are solely responsible to ensure that the operations of MIB are conducted in a manner that comply with Shari'ah principles at all times, we are required to submit a report on the overall Shari'ah compliance environment of MIB. During the year 2020 Shari'ah Board's held meetings with Board of Directors on the following dates:

First Shari'ah Board – Board of Directors' Meeting	–	April 21, 2020
Second Shari'ah Board – Board of Directors' Meeting	–	December 29, 2020

2. To form our opinion as expressed in this report, the Shari'ah Compliance Department (SCD) of MIB carried out reviews of each type of transactions, products, process flows/modus operandi and concepts under the supervision of RSBM/Head Shari'ah Compliance. SCD informed Shari'ah board regarding Shari'ah compliance review activities of Front and Back Offices of MIB during tough conditions of COVID-19 in the country. In 3rd Shari'ah Board meeting FTY – 2020 Shari'ah Board and CEO approved one-third (33%) waiver in assigned tasks of Shari'ah Compliance program – 2020 due to COVID-19 in the country, a list of such branch and non-branch entities were approved by Shari'ah Board. In this regard, 122 branches have been inspected for Shari'ah compliance with strict compliance of COVID-19 related SOPs and as far as Shari'ah compliance review of non-branch is concerned, it has been ensured to comply with approved list of Shari'ah Board with one-third (33%) waiver. In order to enhance the Islamic Banking and Finance knowledge and expertise of branch & non-branch entities' staff members; Shari'ah trainings were made mandatory for all staff of MIB with the coordination of Learning & Development Department (L&D – HRG).

3. Four (4) Instructions & Guidelines and Four (4) Fatawa by the Shari'ah Board of the MIB are in vogue without any changes. All Fatawas & Instructions and Guidelines issued by Shari'ah Board of the MIB are being implemented in the MIB in true letter and spirit.

4. SCD with the coordination of management and under the supervision of RSBM/Head Shari'ah Compliance has reviewed various Product documents and SB reviewed and approved 77 modus operandi out of which 28 for Corporate Banking, 29 for Commercial Banking, 04 for SME Banking and there were16 Standard process flows. As far as Products are concerned, SB issued 57 Shari'ah Vetting Certificates related to products, 19 for Consumer Car Finance, Housing Finance and Murabaha Microfinance which also includes Mera Pakistan-Mera Ghar Housing Finance Scheme & Review of Prime Minister's Kamyab Jawan Scheme has been completed and hopefully it will be launched in January, 2021, 29 for Corporate/Commercial/SME Banking, 5 for Liability Products and there were 2 each for Staff Financing and Banca Takaful products in the year 2020.

5. SCD has also facilitated Islamic Banking training sessions for the front and back offices staff of MIB. For compliance with regulatory instructions L&D not only arranged class room sessions but also uploaded Online Islamic Banking training modules at Learning Management Systems (LMS) for the easy access of Islamic Banking & Finance knowledge to staff. Despite the difficult conditions due to COVID–19, L&D was determined to adhere to cover the

Islamic Banking & Finance trainings of its staff. Therefore, this year L&D has started Virtual classroom session with facilitation of SCD for the safety of staff and it allows learning for all by overcoming geographical obstacles.

Moreover, in view of COVID–19 conditions L&D-HRG has taken initiatives to establish an effective and comprehensive Islamic Banking training mechanism in compliance with IBD Circular No. 02 of 2018, Dated: Jun 29, 2018, "Enhanced Training & Capacity Building Measures for Islamic Banking Institutions (IBIs)" issued by Islamic Banking Department, State Bank of Pakistan, for MIB front and back offices staff at all levels. SCD's staff facilitated as internal trainers on the Shari'ah related training initiatives during the year. Furthermore, alongside regulatory requirements of executive management Shari'ah trainings, L&D and SCD have jointly developed Islamic Banking & Finance Module and AAOIFI Shari'ah Standards Module for capacity building of Executive Management, which will be launched during 2021.

6. Shari'ah Board praises and encourages the continuous, comprehensive & profound efforts and commitment of MIB's Board of Directors and the Management regarding implementation of all instructions and guidelines issued by Shari'ah Board especially under the tough conditions of COVID–19.

Recommendations:

Based on the observations made through Shari'ah review reports and Shari'ah Compliance checks, it is recommended that:

i. In future, there shall be more Shari'ah Trainings in compliance with regulatory requirements. There should be a comprehensive mechanism to cater situations like COVID-19 to ensure continuity and compliance of Shari'ah Trainings.

ii. More focus is needed on Product & Shari'ah trainings of Corporate, Commercial & SME banking and it is strongly recommended to ensure mandatory Product & Shari'ah trainings of the staff members before launching new products. Further there must be additional focus on training of Products which require complex calculations particularly in Musharakah Running Finance, as some issues were found in its transactional calculation during the Shari'ah Compliance review.

iii. It is recommended to arrange general public awareness programs like, Seminars, Workshops and Question & Answer Sessions from MIB platform for building up the true image of Islamic Banking & Finance as well as creating awareness/removing misconception about Islamic banking.

iv. Shari'ah trainings of MIB higher management is strongly recommended.

v. It is recommended and emphasized to continue with Microfinance activities and MIB should encourage the Islamic Microfinance due to its requirement in the country. Through Islamic Microfinance, Islamic Banking Industry can penetrate at grass-root level to facilitate micro level traders.

vi. The newly deployed Pool Management System is currently facing challenges towards fulfillment of unique customers' requirements that results in some adjustments at the time of profit distribution. It is recommended to get these improvements in Pool Management System to avoid any regulatory non-compliance.

vii. Usage of Islamic Banking terminologies must be ensured during MIB's activities. As far as internal environment of the bank is concerned, all staff members of front and back offices are strongly recommended to follow the proper dress code strictly, which should be in line with MIB Dress code policy, cultural norms, and reflect due modesty as required by the dictates of Shari'ah.

Conclusion:

Shari'ah Board has reviewed & advised corrective measures on SBP Inspection Report, the External & Internal Shari'ah Audit and Shari'ah Compliance Inspection reports and is of the view that:

i. MIB has complied with Shari'ah rules and principles in the light of Fatawa, Instructions and Guidelines issued by Shari'ah Board.

ii. MIB has complied with SBP Inspection report in true letter and spirit.

iii. MIB has complied with directives, regulations, instructions and guidelines related to Shari'ah compliance issued by SBP in accordance with the rulings of SBP's Shari'ah Advisory Committee.

iv. MIB has a comprehensive mechanism in place to ensure Shari'ah Compliance in their overall operations.

v. As far as Charity fund is concerned, separate liability account (non-chequing) is opened in separate General Ledgers for each type of Charity Fund Collection

a. Charity due to late payment,

b. Charity due to void transactions and

c. Amount due to non-purification of dividend.

Funds cumulated in above mentioned each type of “Charity Collection Fund Account” is maintained in Shari’ah Compliant remunerative account at the discretion of Shari’ah Board/Resident Shari’ah Board Member.

Charity Fund is utilized for charitable, social welfare, religious, educational or any other purposes approved by Charity Committee /Shari’ah Board.

In the year 2020 the addition in the amount of Charity was PKR 45.333 million from different heads which was instructed to transfer to the Charity account.

**Additions in Charity account during the year**	Rupees in 000s
- Received from customers against late payment	42,477
- Dividend purification amount	771
- Profit on Charity saving account	2,085
**- Charity due to void transactions**	**Nil**
**Total additions in Charity account during the year**	**45,333**

The bank has disbursed the Charity amount to Shari’ah approved charitable organizations as per MIB’s charity policy and SBP’s guidelines. Details of Charity account are available in the note # 19.2. Further it is note able that during the year, there was no addition in Charity account due to any non-Shari’ah compliant transaction of the bank. It shows the strict compliance and control of SB’s instructions and guideline, SBP’s regulations and internal policies of the Bank. Shari’ah Board would like to praise Shari’ah Compliance Department/RSBM for such efforts made by SCD during tough times of COVID-19.

vi. MIB has complied with the SBP instructions on profit and loss distribution and pool management.

vii. While the Bank is actively pursuing training of its human resources about various aspects of Islamic Banking & Finance through training sessions/seminars, however further improvement is required to enhance the level of awareness of Islamic Banking & Finance of the staff, management and the BOD through enhanced training mechanism for each level. The management and the BOD have made sincere efforts and appreciate the importance of Shari’ah compliance in overall operations of MIB.

viii. The Shari’ah Board has been provided adequate resources enabling it to discharge its duties effectively.

Shari’ah Board praises and acknowledged the efforts of Shari’ah Compliance Department/RSBM of MCB Islamic Bank Ltd. that besides Shari’ah Compliance environment of the bank in regulatory inspection there was no instance regarding Non-compliance of regulatory requirements as far as Shari’ah Governance Framework is concerned.

Shari’ah Board would like to take this opportunity to offer praise to Almighty ALLAH and seek his guidance and Tauwfeeq, and to express its wishes for further progress, development and prosperity of Islamic Banking, Alhamdulillah under the sincere efforts of senior management, and Islamic Banking industry in Pakistan as a whole.

وَاللهُ سُبْحَانَهُ وَتَعَالىٰ اَعْلَمُ

Professor Mufti Munib-ur-Rehman
Chairman Shari’ah Board

Mufti Syed Sabir Hussain
Resident Shari’ah Board Member

Mufti Nadeem Iqbal
Member Shari’ah Board

Date of Report: January 21, 2021.

MCB
Bank for Life

بینک نے چیریٹی کی رقم شریعہ سے منظور شدہ خیراتی تنظیموں کو ایم آئی بی کی چیریٹی پالیسی اور اسٹیٹ بینک کی ہدایات والائحۂ عمل کے مطابق فراہم کی ہے۔ چیریٹی اکاؤنٹ کی تفصیلات نوٹ #۲-۱۹ پر دستیاب ہیں۔
یہاں یہ اَمر بھی قابلِ ذکر ہے کہ امسال بینک کے چیریٹی اکاؤنٹ میں کوئی اضافہ باطل و ناجائز معاملات کی وجہ سے نہیں ہوا، جو اِسٹیٹ بینک کی ہدایات، قواعد و ضوابط اور بینک کی داخلی پالیسیوں کی تعمیل اور کنٹرول کو ظاہر کرتا ہے۔ شریعہ بورڈ، شریعہ کمپلائنس ڈیپارٹمنٹ کی کووڈ-19 کے مشکل ترین حالات میں کی جانے والی کوششوں کی تعریف کرتا ہے۔

و۔ ایم آئی بی کے نفع و نقصان کی تقسیم اور پول کی اِنتظام کاری اِسٹیٹ بینک آف پاکستان کی ہدایات کے عین مطابق ہے۔

ز۔ اگرچہ بینک فعّال طریقے سے اپنے افرادِکار کو اِسلامی بینکاری اور مالیاتی نظام کے بارے میں تربیت دے رہا ہے، تاہم اَفرادِکار، اِنتظامیہ اور بورڈ آف ڈائریکٹرز میں آگہی پیدا کرنے کی غرض سے اسلامی بینکاری کی تربیت میں مزید اضافے کی ضرورت ہے۔ اِنتظامیہ اور بورڈ آف ڈائریکٹرز نے اخلاص پر مبنی کوششیں کی ہیں اور اُنہوں نے ایم آئی بی میں شریعہ کمپلائنس کی اہمیت کو تسلیم کیا ہے۔

ح۔ شریعہ بورڈ کو اپنی ذمہ داریوں سے عہدہ برآں ہونے کے لئے خاطر خواہ وسائل فراہم کیے گئے ہیں۔

شریعہ بورڈ، ایم سی بی اسلامی بینک لمیٹڈ کے شریعہ کمپلائنس ڈیپارٹمنٹ / ریزیڈنٹ شریعہ بورڈ ممبر کی کاوشوں کا اِعتراف کرتے ہوئے اس قابلِ ذکر امر کی تعریف کرتا ہے کہ شریعہ کمپلائنس ڈیپارٹمنٹ / ریزیڈنٹ شریعہ بورڈ ممبر نے بینک کے شریعہ کمپلائنس ماحول کو برقرار رکھتے ہوئے شریعہ گورننس فریم ورک کی تعمیل کو یقینی بنایا، جس کی تصدیق ریگولیٹری معائنہ نے بھی کی اور شریعہ گورننس فریم ورک کی کوئی خلاف ورزی رپورٹ نہیں کی گئی۔

شریعہ بورڈ اِس موقع کا فائدہ اُٹھاتے ہوئے اللہ تعالیٰ کی بارگاہ میں حمد و ثناء پیش کرتا ہے اور اُس کے ہدایات و توفیق کا خواہاں ہے اور سینئر منتظمین اور اسلامی بینکاری انڈسٹری کی مخلصانہ کوششوں سے اسلامی بینکاری نظام کی مزید پیشرفت، ترقی اور خوشحالی کا متمنّی ہے۔

شریعہ بورڈ اللہ تعالیٰ کی بارگاہ میں اُس کی تعریف پیش کرنے اور اُس سے ہدایت اور توفیق اور سینئر منتظمین اور اسلامی بینکاری انڈسٹری کی مخلصانہ کوششوں سے ان شاء اللہ اسلامی بینکاری نظام کی مزید پیشرفت، ترقی اور خوشحالی کا متمنّی ہے۔

وَاللّٰهُ سُبْحَانَهُ وَتَعَالٰى اَعْلَمُ

پروفیسر مفتی منیب الرحمٰن
چیئرمین شریعہ بورڈ

مفتی مدیم اقبال	مفتی سیّد صابر حسین
شریعہ بورڈ ممبر	ریزیڈینٹ شریعہ بورڈ ممبر

تاریخِ اِجراء: ۲۱ جنوری ۲۰۲۱ء

مزید یہ کہ کووڈ-19 کے حالات کو پیش نظر رکھتے ہوئے اسٹیٹ بینک آف پاکستان کے اسلامی بینکاری ڈیپارٹمنٹ کی جانب سے جاری کردہ سرکلر (نمبر ۲/۲۰۱۸ بتاریخ ۲۹ جون ۲۰۱۸ء) پر عملدرآمد کو یقینی بنانے کے لئے مؤثر اور جامع اقدامات کئے گئے۔ لہٰذا ایک پر اثر اور جامع پروگرام L&D-HRG کی طرف سے ترتیب دیا گیا، جس کے تحت بینک کے ہر درجہ کے فرنٹ اور بیک آفس اسٹاف کو اسلامی بینکاری کی ٹریننگ دی گئی۔ شریعہ کمپلائنس ڈیپارٹمنٹ کے اسٹاف ممبرز نے شریعہ ٹریننگز کے لئے پورا سال اپنی خدمات دیں۔ مزید برآں، ایگزیکٹو مینجمنٹ کی شریعہ ٹریننگ کی ریگولیٹری ضروریات کے ساتھ ساتھ L&D اور شریعہ کمپلائنس ڈیپارٹمنٹ نے مل کر ایگزیکٹو مینجمنٹ کی مہارت کو مزید بڑھانے کی غرض سے اسلامی بینکاری اور AAOIFI کے جاری کردہ شرعی معیارات کے کورسز تیار کئے ہیں، جو ۲۰۲۱ء کے دوران شروع کردیئے جائیں گے۔

۲ شریعہ بورڈ اپنی جاری کردہ ہدایات ولائحہ عمل پر عملدرآمد کے اعتبار سے خاص طور پر کووڈ 19 کے مشکل حالات میں بینک کے منتظمین کی مسلسل اور جامع کوششوں کی تعریف اور ہمت افزائی کرتا ہے۔

سفارشات:

شریعہ بورڈ شرعی اعتبار سے کئے گئے جائزوں کی جانچ پڑتال کے بعد درج ذیل سفارشات پیش کرتا ہے:

الف۔ مستقبل میں، ریگولیٹری تقاضوں کی تعمیل میں مزید شریعہ ٹریننگز ہونی چاہئیں۔ شریعہ ٹریننگز کے تسلسل اور تعمیل کو یقینی بنانے کے لئے کووڈ-19 جیسے حالات کے لئے ایک جامع طریقہ کار ہونا چاہئے۔

ب۔ کارپوریٹ، کمرشل اور ایس ایم ای بینکاری کی پروڈکٹس اور شریعہ کی ٹریننگز پر زیادہ توجہ دینے کی ضرورت ہے اور نئی پروڈکٹس کو شروع کرنے سے قبل اسٹاف کی لازمی پروڈکٹ اور شریعہ کی ٹریننگز کو یقینی بنانے کی بھرپور سفارش کی جاتی ہے۔ اس کے علاوہ ایسی پروڈکٹس کی ٹریننگ پر بھی زیادہ توجہ دینی ہوگی، جن کے لئے خاص طور پر پیچیدہ حساب وکتاب کی ضرورت ہوتی ہے جیسا کہ مشارکہ رننگ فائنانس، کیونکہ شرعی تعمیل کے جائزے کے دوران مشارکہ رننگ فائنانس کے حساب وکتاب کے معاملات میں کچھ مسائل پائے گئے۔

ج۔ اسلامی بینکاری اور مالیات کی اصل روح کی تشہیر اور عام لوگوں کو آگاہی دینے کے ساتھ ساتھ اسلامی بینکاری کے بارے میں شعور پیدا کرنے اور غلط فہمیوں کو دور کرنے کے لئے، ایم آئی بی کے پلیٹ فارم سے سیمینارز، ورکشاپس اور سوال وجواب کے سیشنز کا اہتمام کرنے کی سفارش بھی کی جاتی ہے۔

د۔ ایم آئی بی کے اعلیٰ منتظمین کی شریعہ ٹریننگز کی بھرپور سفارش کی جاتی ہے۔

ہ۔ مائیکروفنانس سرگرمیوں کو جاری رکھنے کی پرزور سفارش کی جاتی ہے۔ ایم آئی بی کو ملک میں مائیکروفنانس کی ضرورت کے سبب اسلامی مائیکروفنانس کی حوصلہ افزائی کرنی چاہئے۔ اسلامی مائیکروفنانس کے ذریعے، اسلامی بینکاری انڈسٹری کم سرمایہ کے ساتھ کام کرنے والے تاجروں کی سہولت کے لئے ابتدائی درجہ پر کام کرسکتی ہے۔

و۔ موجودہ نئے پول مینجمنٹ سسٹم کے ذریعے فی الحال منفرد صارفین کی ضروریات کی تکمیل مشکلات کا سامنا کررہی ہے، جس کے نتیجے میں منافع کی تقسیم کے وقت کچھ مسائل کا سامنا کرنا پڑتا ہے۔ کسی بھی ریگولیٹری عدم تعمیل سے بچنے کے لئے نئے پول مینجمنٹ سسٹم میں بہتری لانے کی سفارش کی جاتی ہے۔

ز۔ ایم آئی بی کی سرگرمیوں کے دوران اسلامی بینکاری اصطلاحات کے استعمال کو یقینی بنانا ہوگا۔ جہاں تک بینک کے داخلی ماحول کا تعلق ہے تمام عملے کے فرنٹ اور بیک آفسز کے اسٹاف کو لباس ووضع قطع کے حوالے سے منظور شدہ قواعد وضوابط کی سختی سے پیروی کرنے کی تاکید کی جاتی ہے تاکہ اسٹاف کا پیرہن ایم آئی بی ڈریس کوڈ پالیسی، ثقافتی اُصولوں اور شرعی تقاضوں کے مطابق شائستہ مزاج کی عکاسی کرتا ہو۔

خلاصہ کلام:

شریعہ بورڈ نے ایس بی پی معائنہ رپورٹ، بیرونی اور داخلی شریعہ آڈٹ اور شریعہ کمپلائنس کی رپورٹس کو دیکھنے کے بعد اصلاحی اقدامات کے لئے ہدایات دیں، لہٰذا شریعہ بورڈ کے مطابق:

الف۔ ایم آئی بی نے شریعہ بورڈ کے جاری کردہ فتاویٰ جات اور ہدایات ولائحہ عمل کے مطابق شرعی اُصول وضوابط کو پورا کیا ہے۔

ب۔ ایم آئی بی نے حقیقی معنوں میں اصل روح کو مدِ نظر رکھتے ہوئے ایس بی پی انسپکشن رپورٹ پر عملدرآمد کیا ہے۔

ج۔ ایم آئی بی، اسٹیٹ بینک آف پاکستان کے اَحکامات، قوانین وہدایات اور لائحہ عمل (جو اسٹیٹ بینک آف پاکستان کے شریعہ بورڈ کے احکام کے مطابق ہیں) سے ہم آہنگ ہے۔

د۔ شریعہ کمپلائنس کو یقینی بنانے کے لئے ایم آئی بی کے پاس ایک جامع طریقہ کار موجود ہے۔

ہ۔ جہاں تک چیریٹی فنڈ کا تعلق ہے، ہر طرح کے چیریٹی فنڈ جمع کرنے کے لئے علیحدہ نان چیکنگ، انکمبٹی اکاؤنٹ کھولے جاتے ہیں۔

i. ادائیگی میں تاخیر کی وجہ سے چیریٹی،

ii. باطل معاملات کی وجہ سے چیریٹی، اور

iii. منافع سے غیر شرعی منافع منہا کرنے کی وجہ سے چیریٹی۔

عام طور پر مذکورہ بالا تمام مدّات میں جمع کی گئی رقوم اپنے اپنے "شریعہ کمپلائنٹ نفع بخش چیریٹی کلیکشن فنڈ اکاؤنٹ" میں شریعہ بورڈ/ ریزیڈنٹ شریعہ بورڈ ممبر کی صوابدید پر جمع کی جاتی ہیں، چیریٹی فنڈ، چیریٹی کمیٹی/ شریعہ بورڈ کے منظور شدہ کسی خیراتی، معاشرتی بہبود، مذہبی یا تعلیمی مقاصد کے لئے استعمال ہوتا ہے۔

سال ۲۰۲۰ء میں چیریٹی فنڈ کی مختلف مدّات میں جمع کی گئی رقوم میں کل اضافہ ۴۵.۳۳۳ ملین رہا، جس کو چیریٹی اکاؤنٹ میں منتقل کرنے کی ہدایت کی گئی۔

امسال چیریٹی فنڈ میں اضافہ	روپے (000) میں
صارف کی طرف سے ادائیگی میں تاخیر کی وجہ سے	۴۲،۴۷۷
منافع میں سے غیر شرعی منافع منہا کرنے کی وجہ سے	۷۷۱
چیریٹی سیونگ اکاؤنٹ پر منافع	۲،۰۸۵
باطل معاملات کی وجہ سے	کوئی نہیں
امسال چیریٹی فنڈ میں کل اضافہ	۴۵،۳۳۳

شریعہ بورڈ رپورٹ

(مالی سال اختتام پذیر ۳۱ دسمبر ۲۰۲۰ء)

بِسْمِ اللّٰہِ الرَّحْمٰنِ الرَّحِیْمِ

اَلْحَمْدُ لِلّٰہِ رَبِّ الْعَالَمِیْنَ وَالصَّلوٰۃُ وَالسَّلَامُ عَلٰی خَاتَمِ الْاَنْبِیَآءِ وَالْمُرْسَلِیْنَ وَعَلٰی اٰلِہٖ وَصَحْبِہٖ اَجْمَعِیْنَ اَمَّا بَعْدُ

ایم سی بی اسلامک بینک (ایم آئی بی) کا شریعہ بورڈ ستمبر ۲۰۱۵ء کو تشکیل پایا، موجودہ شریعہ بورڈ پروفیسر مفتی منیب الرحمٰن چیئرمین شریعہ بورڈ، مفتی سیّد صابر حسین ریزیڈنٹ شریعہ بورڈ ممبر اور مفتی ندیم اقبال ممبر شریعہ بورڈ پر مشتمل ہے۔ ۲۰۲۰ء میں پورا سال کووڈ-19 کی وجہ سے پیش آنے والے سخت ترین حالات کے باوجود شریعہ بورڈ کی خدمات ادارے کو حاصل رہیں، بینک کے معاملات کے حوالے سے شریعہ بورڈ نے ٹیلی فون اور ویڈیو کانفرنس کالز کے ذریعے بھی کئی غیر رسمی ملاقاتیں کیں۔ علاوہ ازیں ۲۰۲۰ء میں شریعہ بورڈ کے ۴ رسمی اجلاس درج ذیل تاریخوں میں منعقد ہوئے، جس میں دیگر اُمور کے علاوہ نئی پراڈکٹس اور خدمات کے ساتھ ساتھ موجودہ پراڈکٹس اور خدمات میں ترامیم کا شرعی جائزہ بھی لیا گیا۔

- ☆ شریعہ بورڈ کا پہلا اجلاس، ۲۰ مارچ ۲۰۲۰ء
- ☆ شریعہ بورڈ کا دوسرا اجلاس، ۲۵ جون ۲۰۲۰ء
- ☆ شریعہ بورڈ کا تیسرا اجلاس، ۱۴ ستمبر ۲۰۲۰ء
- ☆ شریعہ بورڈ کا چوتھا اجلاس، ۲۹ دسمبر ۲۰۲۰ء

مندرجہ بالا شریعہ بورڈ کی مجالس کے علاوہ بھی سال بھر تمام اُمور کی شریعہ بورڈ کی جانب سے نگرانی میں شریعہ کمپلائنس ڈیپارٹمنٹ نے شریعہ بورڈ کے ساتھ ہم آہنگی کو یقینی بنایا، جس کے نتیجے میں شریعہ بورڈ کی بینک کے شرعی اُمور میں مستقل شمولیت اور بروقت منظوری کو یقینی بنایا گیا۔

۱ بورڈ آف ڈائریکٹرز اور ایگزیکٹیو منتظمین اس اَمر کے ذمہ دار ہیں کہ ایم آئی بی کے جملہ معاملات شریعتِ مطہرہ کے اُصولوں کے عین مطابق ہوں، لہٰذا ہم (شریعہ بورڈ) ایم آئی بی کے شریعہ کمپلائنس کی مجموعی صورتِ حال کے بارے میں ایک رپورٹ جمع کرانے کے ذمہ دار ہیں۔ ۲۰۲۰ء میں شریعہ بورڈ کے بورڈ آف ڈائریکٹرز کے ساتھ ۲ رسمی اجلاس درج ذیل تاریخوں میں منعقد ہوئے:

- ☆ شریعہ بورڈ – بورڈ آف ڈائریکٹرز کا پہلا اجلاس، ۲۱ اپریل ۲۰۲۰ء
- ☆ شریعہ بورڈ – بورڈ آف ڈائریکٹرز کا دوسرا اجلاس، ۲۹ دسمبر ۲۰۲۰ء

۲ رپورٹ میں ظاہر کردہ اُمور کے مطابق اپنی رائے دیتے ہوئے عرض یہ ہے کہ ایم آئی بی کے شریعہ کمپلائنس ڈیپارٹمنٹ نے ریزیڈنٹ شریعہ بورڈ ممبر / ہیڈ شریعہ کمپلائنس ڈیپارٹمنٹ کی زیرِ نگرانی تمام قسم کے مالی معاملات، پروڈکٹس، معاملات کے مراحل اور نظریات کا جائزہ لیا ہے۔ شریعہ کمپلائنس ڈیپارٹمنٹ نے شریعہ بورڈ کو فرنٹ اور بیک آفسز کے شریعہ کمپلائنس جائزے کی سرگرمیوں کے حوالے سے کووڈ-19 میں پیش آنے والے سخت ترین حالات سے آگاہ کیا۔ ۲۰۲۰ء میں شریعہ بورڈ کے تیسرے اجلاس میں شریعہ بورڈ اور بینک کے پریزیڈنٹ نے شریعہ کمپلائنس پروگرام کے مختص کردہ کاموں میں ایک تہائی (۳۳%) کمی کی منظوری ملک میں موجود کووڈ-19 کے پیشِ نظر دی، شریعہ بورڈ نے باقی رہ جانے والی برانچوں اور گروپس / ڈویژنز / ڈیپارٹمنٹس کے شریعہ کمپلائنس جائزے کی فہرست کی باقاعدہ منظوری دی۔ اس حوالے سے کووڈ-19 سے متعلقہ ایس او پیز کی سخت تعمیل کے ساتھ ایم آئی بی کی ۱۲۲ برانچوں کا شرعی اعتبار سے جائزہ لیا گیا اور گروپس / ڈویژنز / ڈیپارٹمنٹس کے شریعہ کمپلائنس جائزے کو شریعہ بورڈ کی منظور کردہ فہرست کے مطابق ایک تہائی (۳۳%) کمی کے ساتھ یقینی بنایا گیا۔ اسلامی بینکاری کے بارے میں تمام برانچوں / گروپس / ڈویژنز / ڈیپارٹمنٹس کی ان کے کام کی نوعیت کے اعتبار سے معلومات اور مہارت کو بڑھانے کی غرض سے شریعہ کی تربیت ہیومن ریسورس ڈیپارٹمنٹ کے تعاون سے تمام اسٹاف کے لئے لازم قرار دی گئی۔

۳ ایم آئی بی کے شریعہ بورڈ کی جاری کردہ ۴ ہدایات و لائحہ عمل اور ۴ فتاوی جات بغیر کسی تبدیلی کے موجود ہیں۔ ایم آئی بی میں شریعہ بورڈ کی جاری کردہ تمام ہدایات و لائحہ عمل اور فتاوی جات پر عملدرآمد کو ان کی اصل روح کے مطابق یقینی بنایا گیا۔

۴ بینک کے منتظمین کے تعاون سے شریعہ کمپلائنس ڈیپارٹمنٹ نے ریزیڈنٹ شریعہ بورڈ ممبر / ہیڈ شریعہ کمپلائنس ڈیپارٹمنٹ کی زیرِ نگرانی ۲۰۲۰ء میں ان تمام پراڈکٹس کی دستاویزات کا جائزہ لیا اور شریعہ بورڈ نے ۷۷ فائنانسنگ کے لائحہ عمل کی دستاویزات جن میں سے ۲۸ کارپوریٹ بینکاری، ۲۹ کمرشل بینکاری، ۴ ایس ایم ای بینکاری اور ۱۶ تمام صارفین کے لئے استعمال کئے جاسکنے والے فائنانسنگ کے لائحہ عمل کا جائزہ لیا اور ان کی منظوری بھی دی۔ جہاں تک پراڈکٹس کا تعلق ہے، شریعہ بورڈ نے ۷۵ شرعی منظوری کے سرٹیفکیٹ جاری کئے، جن میں سے ۱۹ کنزیومر کار فائنانسنگ، ہاؤس فائنانسنگ، مرابحہ مائیکرو فائنانس شامل ہیں، اس میں میرا پاکستان میرا گھر ہاؤسنگ فائنانس اسکیم بھی شامل ہے اور پرائم منسٹر کامیاب جوان اسکیم کا شرعی جائزہ بھی مکمل کیا جا چکا ہے، جو کہ جنوری ۲۰۲۱ء میں جاری کردی جائے گی۔ ۲۹ شرعی معائنہ سرٹیفکیٹ کا تعلق کارپوریٹ، کمرشل اور ایس ایم ای بینکاری سے ہے، جبکہ ۵ لائبلیٹی پراڈکٹس، ۲ اسٹاف فائنانسنگ اور ۲ کا تعلق بینکا تکافل سے ہے۔

۵ شریعہ کمپلائنس ڈیپارٹمنٹ نے بینک کے فرنٹ اور بیک آفس اسٹاف کے لئے اسلامی بینکاری کی ٹریننگ کے کئی پروگرامز منعقد کئے ہیں۔ مزید برآں اسٹیٹ بینک آف پاکستان کے اسلامی بینکاری ڈیپارٹمنٹ کی جانب سے جاری کردہ ہدایات پر عملدرآمد کو یقینی بنانے کے لئے کلاس روم سیشنز کا انعقاد کیا گیا، اس کے علاوہ لرننگ مینجمنٹ سسٹم کے ذریعے آن لائن کورسز کرائے گئے، جس نے اسٹاف کے لئے اسلامی بینکاری کی معلوت تک رسائی کو آسان بنایا۔ کووڈ-19 کی وجہ سے مشکل حالات کے باوجود، L&D نے اپنے اسٹاف کی اسلامی بینکاری اور مالیات کی تربیت کا عزم کیا۔ لہٰذا اس سال L&D نے عملے کی کووڈ-19 سے حفاظت کو مدِ نظر رکھتے ہوئے شریعہ کمپلائنس ڈیپارٹمنٹ کے تعاون سے ورچوئل کلاس روم سیشنز کا آغاز کیا، جس کی وجہ سے جغرافیائی رکاوٹوں سے نکل کر اسٹاف کے لئے سیکھنے کے مواقع میسّر آئے۔

Sustainability & Corporate Social Responsibility

As a leader in the Pakistani banking sector, MCB Bank has a great legacy in looking after the preservation of the interests of all our stakeholders. Our employees fully embody our values of service and quality.

Through careful evaluation and selection, the Bank selects and promotes projects and services that benefit the Pakistani economy and society in the most effective manner. Particular emphasis is placed upon the need to promote environmental sustainability and social welfare.

Our policy

We ensure that CSR initiatives embody a vision of harmonious and sustainable development in Pakistani communities. Broadly speaking, the pillars of CSR activities undertaken by the Bank ensure:

1. Compliance with relevant laws and regulations both in letter and spirit
2. Business operations with honesty and integrity
3. Engagement in social welfare activities that help strengthen communities and contribute towards the uplift of society
4. Support and promote financial inclusion and literacy
5. To build and maintain sound relationships with customers and other stakeholders through open and fair communication in order to contribute towards sustainable image-building
6. Respect for culture, customs, history and laws as the Bank constantly searches for safer, cleaner and better practices that meet the growing needs of society
7. Minimize environmental footprint to coexist harmoniously whilst encouraging minimum wastage of resources

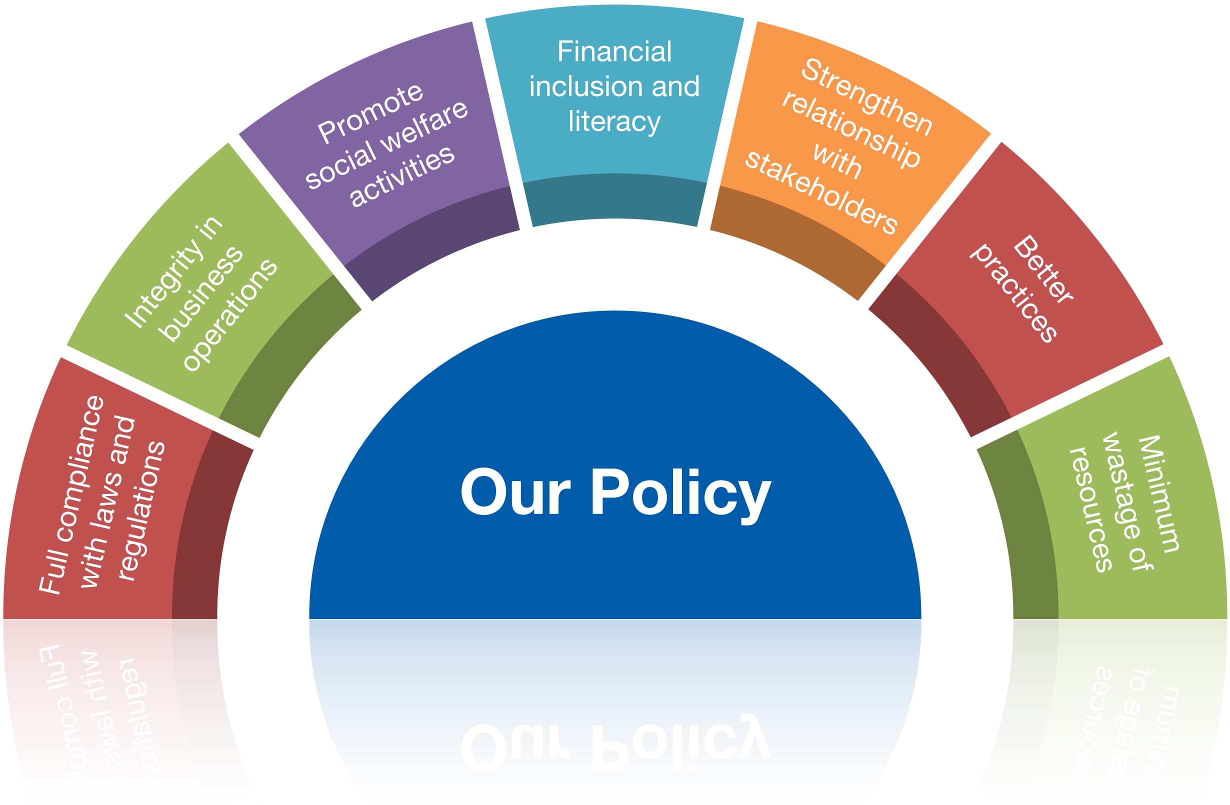

Our approach to sustainability

The Bank has focused on several key principles as an institution. It is committed towards fostering a better work place and cleaner environment through its varied initiatives. By committing to a culture of excellence, good governance, transparency and integrity, it ensures that all activities are conducted in a manner that is ethically responsible and beneficial for all stakeholders. MCB Bank has a well-defined Code of Ethics and Conduct policy that serves as a guideline for the behavior and ethics of employees.

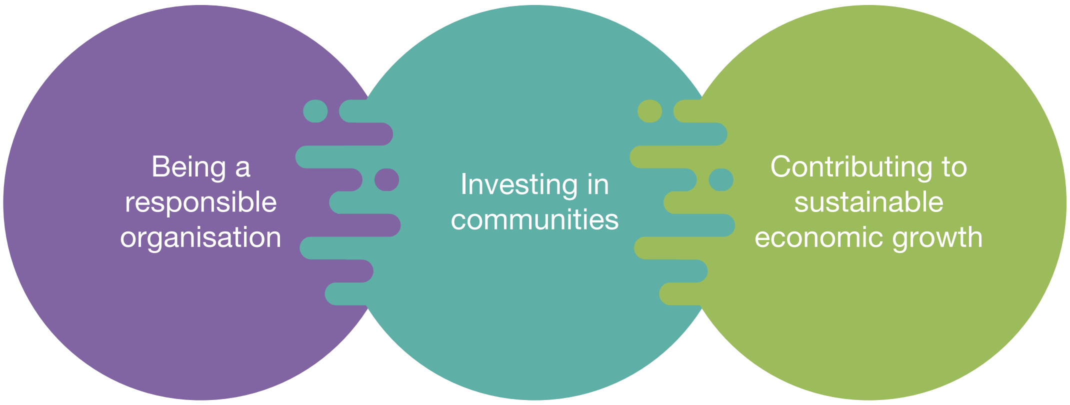

Contributing to sustainable economic growth

MCB Bank uses its core business of banking to promote sustainable development in all the markets it operates in.

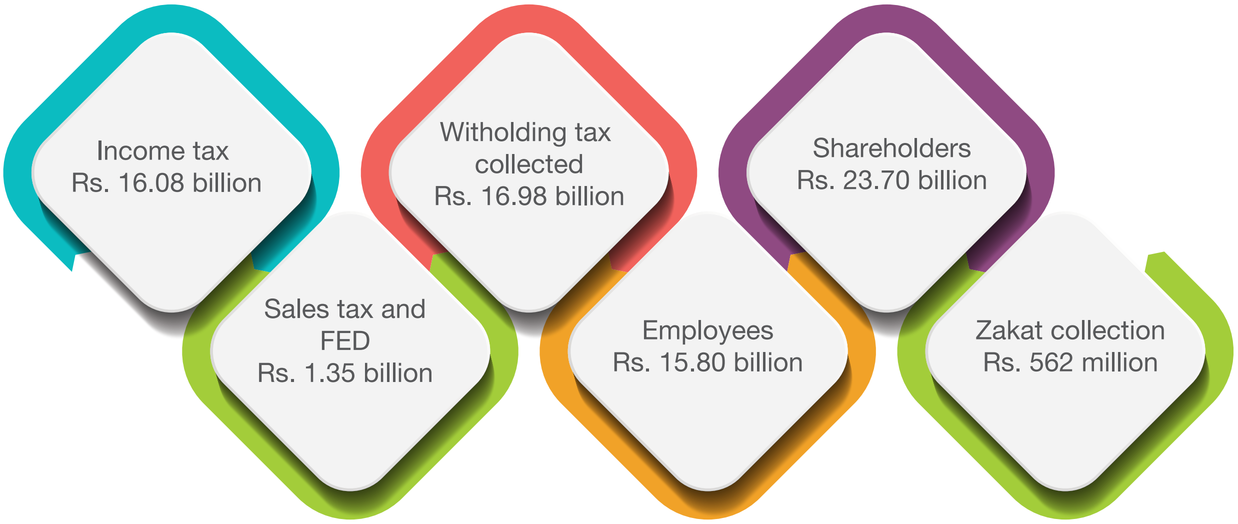

Contribution to Economy & National Exchequer

MCB Bank has the highest market capitalization in the banking industry. In 2020, the Bank paid approximately PKR 16.08 billion on account of income taxes to Government Treasury and collected over PKR 16.98 billion for the National Exchequer as withholding tax agent under different provisions of Income Tax Ordinance 2001. In addition, the Bank has also paid PKR 1.35 billion in respect of sales tax and FED.

The contribution by the Bank to the national economy by way of value addition was PKR 68.48 billion, out of which around PKR 15.80 Billion were distributed to employees and PKR 23.70 billion to shareholders.
Zakat is an essential component in delivering assistance to those most in need. The Bank bolstered the zakat collection efforts of prominent public welfare organisations such as Shaukat Khanum and Edhi Welfare Organisation through its communication mediums such as MCB Mobile Banking, MCB Internet Banking and ATM Screens. MCB Bank also contributed to the national exchequer in Zakat Deductions to the sum of PKR 562 million.

The Bank is making significant contribution to the development and growth of the country.
An analysis of the Bank's value creation and allocation of value among key stakeholder groups is represented in Statement of Value Added.

Key financial highlights

Key financial figures and related ratios are discussed in financial performance section.

Being a responsible organization

Our foremost duty is to create a platform which reflects our values. We do this by making the right investments in developing our human resources, protecting the interests of our customers, weighing environmental impact in our decision making and combating the scourge of corruption with our best practices.

The Bank’s policies therefore address these key aspects:

- Measures to combat COVID-19 pandemic
- Occupational health and safety
- Business continuity management
- Business ethics and anti-corruption measures
- Quality checks and mystery shopping
- Service council
- Customer experience management
- Turnaround Time (TAT) monitoring
- Consumer protection measures

Measures to Combat COVID-19 Pandemic

With the onset of the COVID-19 pandemic during Q1-2020, the Bank took immediate measures to offset the risks associated with the rapidly spreading pandemic. While ensuring that day to day business was not adversely impacted, the safety and security of all employees and valued customers was a key priority. The following steps were implemented with immediate effect while applying out of the box strategic planning and execution within stringent timelines:

- Immediate reduction of staff at branches and back offices via adaptation and improvisation of workplace dynamics
- Daily attendance reporting detailing staff strength at Head Office/key buildings and branches
- Effective implementation and review of regulation directed SOPs at all back office buildings and branches (including the deployment of sanitizers, thermal guns, masks)
- Arrangement of staff testing at branches, buildings, alongside reimbursement for individual staff testing
- Utilization of alternate building spaces to relocate critical staff while ensuring social distancing measures
- Creation of an alternate fully functional working environment for Bank's senior management in the eventuality of an emergency evacuation required at the Head Office

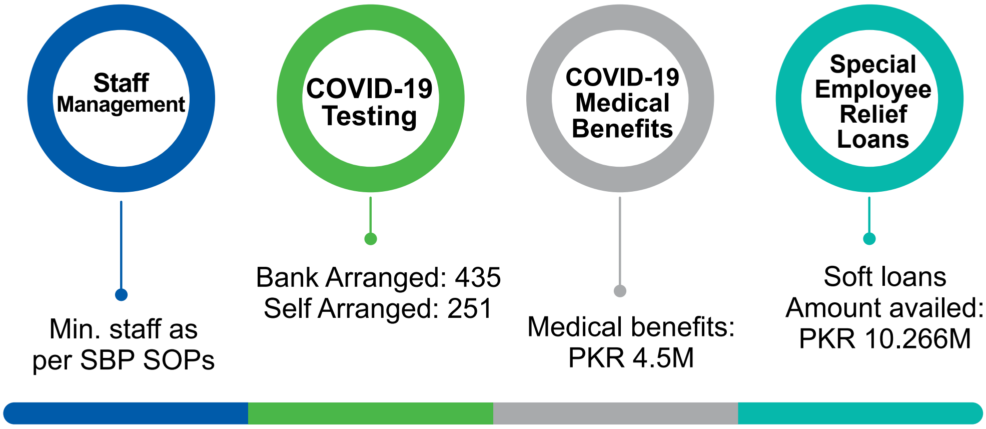

Collaborative measures implemented by the Bank to ensure staff and customer safety during the Covid-19 pandemic:

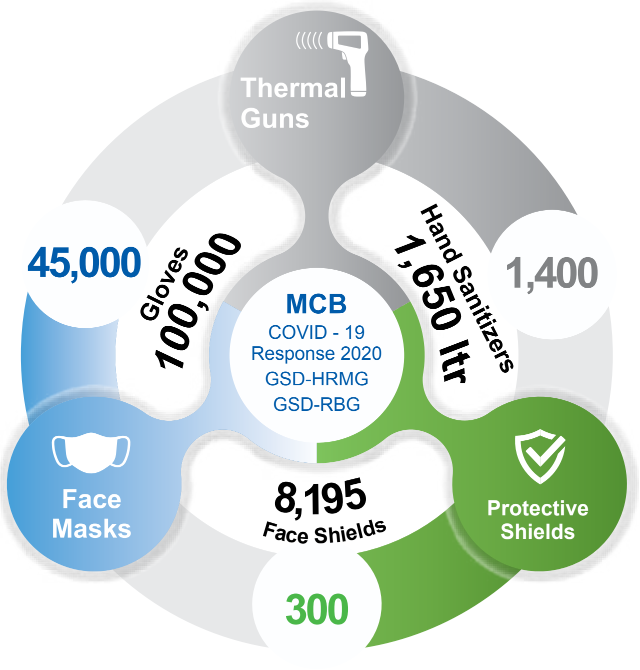

While addressing the unprecedented challenges posed by the pandemic, the bank maintained its usual support for staff with ongoing CSR measures including Umrah allowance, staff welfare fund, and allowances for marriages/funerals.

In the case of operations in Sri Lanka, the bank took all relevant health & safety measures to ensure the well-being of our staff, customers and all stakeholders. All branches were provided necessary safety equipment and materials to monitor and safeguard the working environment. Strict monitoring process was implemented and practiced across the Country Office and all branches in line with Health Ministry Guidelines to mitigate the risks. During the pandemic lockdown period, given the importance of essential banking services to our valued customers, selected MCB Bank branches remained open adhering to regulatory and local authority guidelines.

Occupational Health and Safety

For any progressive and productive organization, sound health & safety alongside a congenial work environment serve as key elements to gauge organizational impact; MCB Bank takes pride in providing a productive environment to its employees.

Since there is a strong focus on the safety and wellbeing of Bank employees and its customers, all iconic buildings and branches of the Bank are equipped with modern fire safety, surveillance and security equipment (as applicable). Furthermore, trained security personnel ensure the physical safety and security of all employees, customers, buildings and equipment.

The Bank has developed a comprehensive “Health, Safety & Environment Policy” which is periodically reviewed and updated by the Bank Management and implemented across the board.

MCB Bank is cognizant of the fact that to achieve any objective, staff is the prime enabler. Therefore building administrators, floor coordinators and additional support staff in multistoried/multipurpose buildings ensure the provision of a safe, healthy and conducive work environment for Bank staff.

Additionally, staff evacuation drills are periodically conducted under the supervision of the security department which helps staff practice how to act and respond during different emergency situations. First aid kits are maintained at all major buildings and branches of the Bank.

The dangers of smoking are well-known. Among adults, second-hand smoke can cause serious ailments including

cardiovascular and respiratory diseases, coronary heart disease and lung cancer. Keeping in mind the dangers posed by smoking to both smokers and non-smokers alike, the senior management of MCB Bank has declared all Bank buildings No Smoking Zones. Staff members are allowed to smoke at a safe distance away from bank premises. For the safety of bank staff and their family, pictorial safety messages on topics such as Dengue prevention measures, safe driving/riding in the rain tips, precautionary measures in case of smog/fog, heat precautions, earthquake safety tips, fire safety actions, etc. are also frequently disseminated through the Bank's internal communications.

All permanent employees of the Bank are provided with medical coverage under a comprehensive staff group life and medical insurance policy. MCB Bank has always been keen in taking initiatives such as on-site health checkups, arranging seminars on health & safety and emergency preparedness within its staff to safeguard life and assets of the Bank.

Business Continuity Management

Business Continuity Management (BCM) is a multifaceted process that identifies and recognizes risks, threats and vulnerabilities that potentially impact the Bank's operations both in internal and external contexts. BCM provides framework and creates ability for an organization to mitigate risk in a dynamic, ever-changing environment and still perform its critical processes and functions.

A robust Business Continuity Plan (BCP) outlines a range of disaster scenarios and the steps to be taken in order to minimize the potential harm to business during adverse situations. System recovery procedures are structurally planned for the future, therefore the Bank endeavors to sustain and deploy an effective enterprise-wide BCM program to provide seamless services and products for customers/stakeholders.

The Board of Directors periodically reviews and gauges our preparedness to deal with any untoward situation. The senior management of the Bank ensures an effective Business Continuity Policy & Framework exists. This ensures clear and concise plans are maintained for all critical areas to reduce the risk of downtime in any contingency scenario.

In these difficult times, when the global COVID-19 pandemic has impacted every segment of life, the Bank adopted a proactive approach and immediately arranged for Personal Protective Equipment’s (PPEs) comprising of face masks, hand gloves, face shields, hand sanitizers etc. for its staff and visiting customers. Furthermore, special screening arrangements, maintenance of social distance, reducing the deployment of staff, periodic disinfection in branches and offices were key initiatives taken to ensure continuity of business and services, thus complying with Government and WHO precautionary instructions.

Furthermore, to nurture and enhance the confidence in Bank’s system and processes, Business Continuity Plans are periodically reviewed and updated. Since BCM runs smoothly across the Bank’s corporate structure and branch network, the confidence and expectations of millions of customers, stakeholders and regulator is upheld. The BCM is part of our commitment to ensuring continued functionality of critical businesses and functions under any circumstances.

Business Ethics and Anti-Corruption Measures

The Bank actively identifies and addresses possible risk factors through the implementation of policies and procedures designed to reduce the possibility of such incidents. In this regard, it has fielded, alongside its Human Resource Policies and Procedures, a comprehensive “Code of Conduct and Business Ethics” which is disseminated to staff for information and sign off. This document is also readily available to all staff on Intranet Portal.

The Bank continues to maintain a strong compliance culture across the board. Employees are expected to perform all tasks with diligence and honesty at all times. The Code of Conduct of the Bank comprehensively defines the values and minimum standards for ethical business conduct.

Employees ensure that all interactions with clients, competitors, business partners, government and regulatory authorities, shareholders, or with one another, follow a vigorous ethical standard. The Bank's foremost effort is to ensure that the conduct of the employees is impeccable.

This is done with the help of guidelines that ensure compliance with all applicable laws and regulations. MCB Bank strives to ensure a friendly and harassment-free environment for all employees. The policy for protection of women harassment has been revisited and is circulated Bank-wide every year. Zero tolerance for any form of harassment or discrimination is further covered in the Bank's existing Code of Conduct.

The Disciplinary Action Committee (DAC) takes vigorous action to address any violation of policies, procedures, acts of fraud and forgery, breach of discipline and code of conduct, ethics and business practices, law of land and statutory regulations by an employee.

To maintain a harmonious and efficient work environment in which the employees are assured a non-discriminatory, transparent, harassment free and respectful atmosphere regardless of their caste, religion and gender, HRMG has issued a clear and non-discriminatory Code of Conduct, violation of which may lead to disciplinary action.

Service Council

Service Council is a monthly forum, chaired by the President, which brings together key stakeholders from across the bank with a view to place service on the forefront through thought leadership, collaborative discussions and creation of a clear service roadmap.

Customer Experience Management

Feedback is solicited from customers for all contact points via surveys and remedial actions are taken for identified areas. The end goal of these measures is to be the most preferred bank in Pakistan.

Turnaround Time (TAT) Monitoring

Monitoring and evaluation of service indicators is part of the belief in increasing and retaining one's customer base. In order to maintain a strong hold on processes within the Bank, the Service Quality Division has devised several controllable measures at par with prevailing market practices. Against each measure, a tolerance level along with a timeline is set. Similar to Branch Banking, indicators for Consumer Assets, Credit Cards, Bancassurance, Call Centre, Mobile Banking Internet Banking and ATM Uptime are monitored on a monthly basis.

Quality Checks and Mystery Shopping:

During 2020, 99% of total branches were monitored with respect to service parameters and protocols. The remaining branches were not visited owing to security concerns/remote locations and few were under renovation. Moreover, 929 branches were 'Mystery Shopped' by independent external agencies and results of this activity were shared with management for further improvement.

Consumer Protection Measures

The Bank is committed to providing quality products and services to its customers. It maintains a privacy statement for the usage of its products i.e. Credit Cards, ATM PINs etc.

To ensure a culture of 'Quality Customer Service' the Bank has a dedicated Service Quality Division with the objective of strengthening the Bank's service culture. Regular training sessions are conducted in all Circles, Call Centers and other front-end staff offices regarding 'Service Excellence' and 'Customer Satisfaction'.

Customer Grievance Handling

Bank considers complaints as opportunities for improvement and understands the link between complaint resolution and customer loyalty. We believe that complaints are a primary measure of customer dissatisfaction; thus, they should be taken seriously and staff should be encouraged to bring complaints to the forefront so that gaps can be identified and fixed.

Service Quality (SQ) function is the custodian of customers' grievance handling and works in collaboration with all businesses / functions of the bank responsible for acknowledging, investigating, tracking, escalating and resolving customer complaints within specified turnaround times. A centralized complaint resolution team manages all customer complaints through a Complaint Management System. Currently, all our customer touch points have access to this system so as to ensure that all complaints, whether verbal or written, are immediately captured in the system.

Following are the channels through which complaints are received:

- MCB Call Center
- MCB Branches
- MCB E-mail

- Social Media
- Letter/Fax
- Customer Service Centers
- Banking Mohtasib Secretariat
- State Bank of Pakistan
- Management Committee (MANCOM)

The Bank makes its best effort to ensure that resolution of complaints is comprehensive, appropriate and quick. The customer is kept informed on the status of their complaint, starting from complaint acknowledgement till its resolution. The escalation matrix for complaint resolution observed and designed in the system is such that a complaint, if not resolved within the specified turnaround time, gets escalated to the next senior level of management and keeps on escalating further till resolved.

SQ also performs in-depth qualitative and quantitative complaints analysis followed by suggestions and recommendations in order to eliminate root causes of customer issues and drive continuous improvement.

During 2020, a total of 152,234 complaints were logged in the system out of which 150,788 complaints were resolved during the year (resolution rate 99.05%).

There was a 35% increase in total logged complaints in 2020 as compared to the previous year. Total complaints logged during 2019 were 112,359.

Statement of Complaints	Numbers	Percentage
**Total Complaints Received**	152,234	–
Closed	150,788	99.05%
Open	1446	0.95%
Average time taken for resolution		8 Working Days

Total Login Details:	Total	Contribution
Complaints	152,234	88%
Request/Queries/Reversals	20,320	12%
Total	172,554	100%

Investing in communities

MCB Bank is committed to creating sustainable economic and social development for our stakeholders.

Following areas were addressed by the Bank in 2020:

- Outreach to the healthcare sector
- Education
- Sports
- Contribution to the public good
- Equal opportunity for all employees
- Energy conservation
- Environmental protection
- Women empowerment
- Green banking activities and initiatives

Outreach to the Healthcare Sector

The Bank seeks to support key initiatives that bolster the healthcare sector of Pakistan.

During 2020, the coronavirus pandemic was a key area for focus. To help ensure the health and safety of those fighting at the frontline of the crisis, the Bank donated Personal Protective Equipment (PPE) such as masks, protective clothing and goggles for personnel of Health Department- Government of Punjab and Makran Division.

MCB Bank deepened its commitment to major health initiatives throughout the year. Comprehensive marketing collateral was also deployed to raise awareness of breast cancer in collaboration with Pink Ribbon. The awareness campaign also supported Pink Ribbon in the NGO's efforts to raise funds for Pakistan's first ever Breast Cancer Hospital. The Bank also supported the construction of hospital and completion of pathology lab at Saleem Memorial Trust Hospital.

The Bank also helped generate awareness for organizations like Saylani Welfare Trust, Sindh Institute of Urology and Transplantation (SIUT), Edhi Welfare Organization and Shaukat Khanum Memorial Cancer Hospital with its internal and external communication platforms.

As part of its effort, the Bank helped to promote donations through platforms such as MCB Mobile Banking and MCB Internet Banking. MCB Bank also supported:

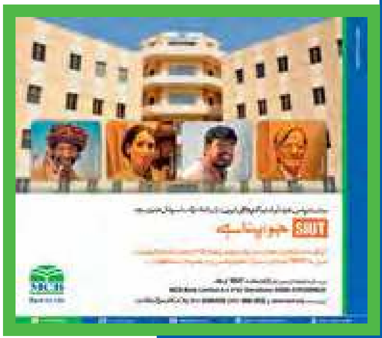

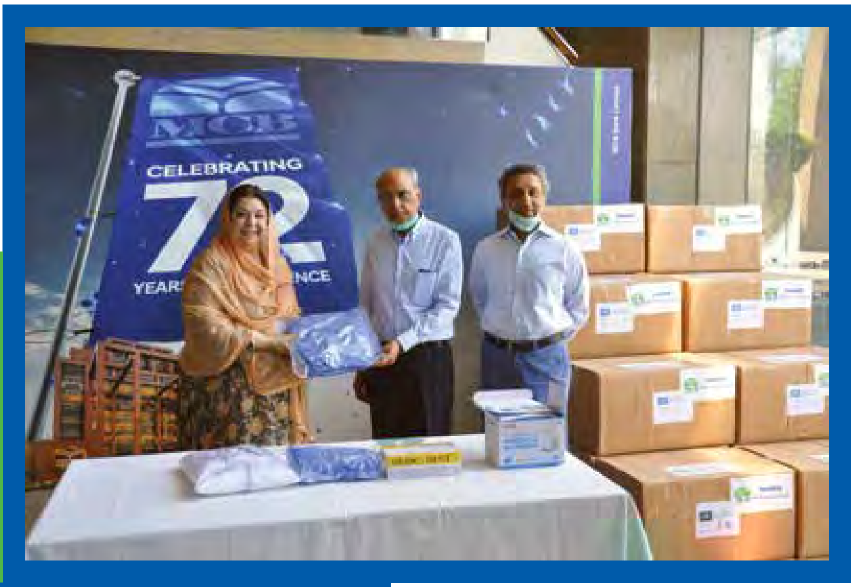

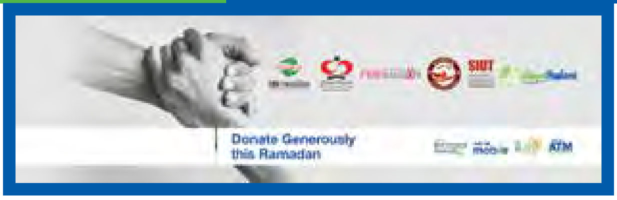

Education

Given the importance of the educational sector, MCB Bank fully supports its uplift. Furthermore, the Thardeep Rural Development Programme (TRDP) was provided support with the purchase of books and furniture for a public library established in the rural area of Mithi, Sindh. The initiative focuses on raising the literacy level in this location. MCB Bank also contributed to the 1st Convocation held by Indus Medical College.

Sports

The Bank recognizes the important role played by sports in the well-being and health of the Nation. In this regard, it focused on supporting the POF All Pakistan Floodlit Football Tournament and International Men's & Women's Squash Tournament.

Contribution to the Public Good

MCB Bank will always support any endeavor that promotes the greater good. This was done through different initiatives throughout the year. The aim of these projects was to have a positive social impact on the many communities the Bank works with in different capacities.

As part of its civic responsibility to support communities affected by the COVID-19 pandemic, the Bank provided critical support by donation of ration packs to needy families in Lahore in collaboration with the Jahandad Society for Community

Development. In battling the scourge of corruption, the Bank was poised to support the efforts of the government by sponsoring branding material for the Anti-Corruption Day Event.

Measures were taken to minimize the impact of COVID-19 and maximize output during the crisis for the Bank's customers. In line with SBP directives, the Bank provided financial relief with restructuring/rescheduling for 1,800+ accounts. In the case of credit cards, in addition to restructuring credit card dues in some cases, the Bank offered low markup installment and deferment plans to assist customers.

The Bank also contributed to society during these trying times in consultation with its insurance providers by providing complimentary benefit of PKR 150,000 to our conventional and Takaful policy holders in addition to the existing policy benefits and provided relaxation in premium / contribution payments to all its policy and certificate holders till June 2020.

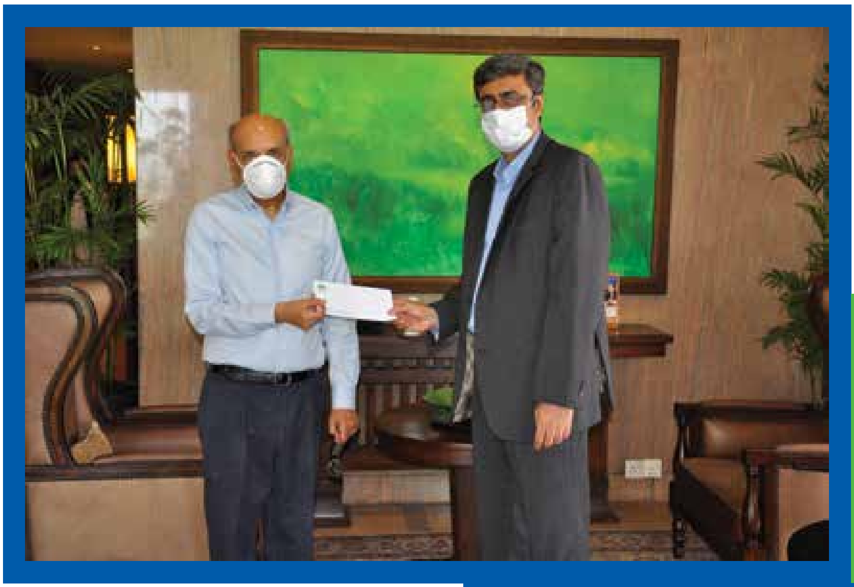

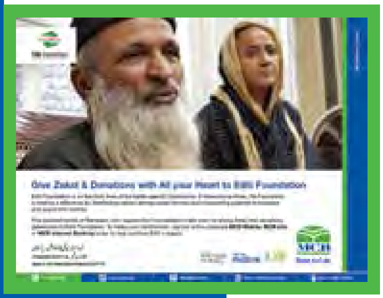

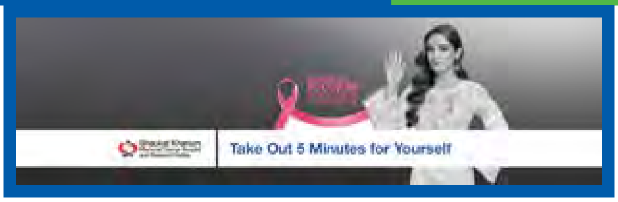

Equal Opportunity for All Employees

The Bank prides itself on providing equal employment opportunities, free of discrimination, and implemented on a methodical merit based non-discriminatory selection process. The Bank capped off the year 2020 with permanent staff strength of 13,272 employees and 103 contractual employees. The ratio of female staff members stood at 16.3% compared to 15.8% from the previous year.

Energy Conservation

MCB Bank policies are geared towards reducing our environmental footprint and promoting the use of energy smartly and economically to cut down on operational expenses. This policy is enforced countrywide to conserve energy, by exercising strict control over electricity lights discipline whether in the Bank Branches or Principal offices.

Recent WWF certification of Green Banking to MCB Centre building is a great achievement in-line with energy conservation by implementing policies which encompass solar energy, LED Lights, paperless work and water conservation. WWF-Green Office is a practical environmental program designed for office/workplaces, which helps to reduce the carbon footprint, achieve resource conservation and contribute towards slowing down climate change. Under the guidelines of WWF-Green Office, training programs were run by WWF –Green Office Team at MCB Centre which followed by audit for "logo" and "diploma" and WWF Certification is awarded to MCB Centre for fulfilling their minimum Green Office criteria. The Bank also engages employees through its internal communication forum to follow the best practices and initiatives to inculcate consciousness to save energy.

Energy Saving Measures

MCB Bank is already following the policy to exercise strict controls over excessive lighting and restricting switching-on of lights to required areas only as well as switching-off all lighting immediately on office closure timing except those for building security requirement.

Almost all the bank buildings have been switched over to the LED Lights instead of conventional bulbs/tube lights. Post office/late hour sitting is discouraged to exercise saving of energy. To exercise maximum control over building energy resources, a Building Management System (BMS) is installed at MCB Bank Principal buildings (MCB House Lahore, MCB Center Lahore and MCB Tower Karachi). The facility enables centralized control from a single point/place.

The first of its class waste heat from cogeneration plant is installed at one of MCB Bank's principal buildings i.e. MCB Centre, Lahore. A waste heat of gas engine (1555 KW) is also installed at MCB Principal building, MCB Tower Karachi, to produce hot water to be used in chiller with boiler. Almost 100 to 150 tons of extra cooling is generated through this process.

Environmental Cleanliness & Protective Measures

MCB Bank is working on Environmental Protection as a top strategic priority. The Bank believes in a safe environment for the welfare of the staff.

MCB Bank is moving towards paperless banking and resource efficiency in our operations. Some of our notable initiatives include reduction of environmental footprint in transportation and mobility of staff, energy efficiency and greening of office premises and self-segregation of waste into dry and wet waste streams at source. We ensure that all our dry waste including paper and plastic streams are reused in our corporate offices and bank branches through ethical recycling measures.

We ensure that our wet waste including kitchen and organic materials are responsibly disposed at landfills with minimum impact to the environment. For this purpose, we use technology to monitor and track our sustainability drive to achieve zero waste objectives. Partnership building is essential for environmental sustainability, therefore we are keen to cultivate impactful collaboration with civil society organizations such as AMAL which facilitates the Green Office Program to achieve net zero objectives for the second consecutive year. With this collaboration, we intend to set new trends for sustainable banking in Pakistan.

The COVID-19 pandemic did not hold back efforts towards greater sustainability. Some of our efforts resulted in following impacts:

1. Collection of dry waste for ethical recycling = 9.2 tons
2. Reduction in CO2 emissions = 450 tons

MCB Bank has taken these initiatives not only to meet legal requirements but as its corporate responsibility to address environmental concerns of our clients and stakeholders. We are proud to remain ahead of our competitors as one of the first banks in Pakistan to adopt Green Banking Guidelines of the State Bank in letter and spirit. The MCB Bank management appreciates the cooperation it received from its employees and staff and the interest shown by stakeholders in adopting responsible and ethical business practices.

High quality janitorial services in the cities are provided for three principal buildings (MCB House Lahore, MCB Centre Lahore and MCB Tower Karachi). For each building, administrators and building floor coordinators conduct periodical checks to maintain the highest cleanliness standards.

Green Banking Activities and Initiatives

Activities and initiatives taken by the Bank under SBP IH&SMEFD Circular # 08 dated October 09, 2017 are discussed in Green Banking Initiative section.

Certifications Acquired and International Standards Adopted

World Wide Fund for Nature (WWF) - Green Office Certification

MCB has successfully attained 'Green Office Certification' from WWF for one of its iconic buildings; MCB Centre, Lahore. This certification represents a landmark achievement in MCB's pursuit of reducing the ecological footprint at its workplace and has consequently placed the Bank in an exclusive club of domestic Banks that have met the requirements of this rigorous assessment and certification program.

International Standards of Sustainability adopted as per UN SDGs and UN Global Compact

	UN SDG Indicator	UN Global Impact	MCB Banks' Alignment
	3: Good Health and Well Being		In addition to supporting employees' health and wellbeing, the Bank also actively engages in community services within the health sector under its CSR Plan.<br><br>The detailed initiatives have been disclosed in the Sustainability & Corporate Social Responsibility section of the Annual Report.
	5: Gender Equality	6: Elimination of discrimination with respect to employment.	The Bank prides itself on providing equal employment opportunities that are free of discrimination and are being implemented on a methodical and merit based selection process:<br>• There has been a consistent growth in the number of female staff at the Bank (15.8% in 2019 to 16.3% in 2020).<br>• The representation of women in the senior management positions stood at 16%; with 20 female staff members reporting directly to the Head of Departments (HOD's) who in turn report to the CEO.<br><br>The Bank's product portfolio has been deployed to foster the captioned goal by actively promoting the MCB Ladies Account portfolio, wherein a total of 2,520 Accounts were successfully added to the portfolio during 2020.
	7: Affordable and Clean Energy	8: Initiatives to promote greater environmental responsibility<br><br>9: Fostering environment friendly practices	• Bank has converted 10 branches to solar power operations.<br>• Buildings have been switched over to energy efficient LED Lights instead of conventional bulbs/tube lights.<br>• A Building Management System (BMS) has been installed at the principal buildings to enable centralized control over management of energy resources.<br>• Contributing to the Central Bank's cause for inculcating environmental considerations in banking products, the Bank has extended financing facilities to customers against renewable energy products; the total outstanding balance as at December 31, 2020 stood at Rs. 75 million.
	8. Decent Work and Economic Growth	3: Upholding freedom of association<br><br>10: Working against corruption.	Despite constraints created by the COVID-19 outbreak, the Bank continued to effectively play its role as a key service provider and intermediary in the financial market; hence, making a significant contribution to the country's economic growth:<br>• Created employment opportunities which led to the hiring of 1,638 employees.<br>• Contributed Rs. 16.08 billion to the national exchequer on account of income taxes to the Government Treasury.<br>• Extended finance to all key economic segments including the SME and Agriculture sector which cumulatively contribute above 40% to the domestic GDP.<br>• Played a central role in supporting Governments' and Central Banks key pandemic responses for credit extension and un-interrupted provision of essential banking services to the general public.
	9: Industry Innovation and Infrastructure		The Bank continues to actively contribute to the Central Banks cause of improving financial inclusion in the country by extending branch outreach and customer digital touchpoints.
	12: Responsible Consumption and Production		MCB's Green Banking Policy lays the foundation for safeguarding the Bank against environmental vulnerabilities and playing its due role in transforming the country towards a low carbon and climate resilient economy.<br><br>The detailed policy has been disclosed in the Green Banking section of the Annual Report.

Green **Banking**

"Green Banking" is a term that refers to promotion of environmentally friendly practices that aid Banks and their clients in identifying and managing environmental risks as well as reducing their carbon footprint and related socially adverse actions.

MCB Bank undertakes its responsibility to be recognized as an organization that is aware of its environmental obligations. MCB Green Banking Office (GBO) was established in 2018 which is effectively implementing and monitoring the Green Banking Guidelines issued by State Bank of Pakistan. MCB GBO aims to make Banks processes, use of IT and physical infrastructure more sustainable and efficient to have minimal impact on the environment.

The Bank is continuously striving to inculcate the environmental consciousness among stakeholders, transforming operational processes to safeguard against environmental vulnerabilities, and play its due role in conversion of country's economy into a low carbon and climate resilient economy.

Few Green initiatives undertaken by MCB Bank are as mentioned below:

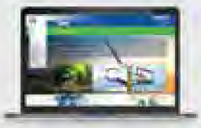

Green Banking Web-Page

A dedicated web-page has been developed on the MCB Bank Corporate website for external stakeholders' awareness regarding MCB's initiatives on Green Banking segment.

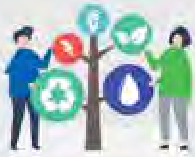

Green Products/Services

MCB Bank endeavors to integrate sustainability into products and services provided by the Bank. Such products involve environment-friendly features including renewable energy, modern drip/sprinkler irrigation techniques etc. All MCB Fun Club account holders have the option of planting a tree in their name and watch it grow on Google Earth. In addition, clients are provided with advisory services on availing green finance for environmentally friendly/renewable energy projects.

An array of Green initiatives has been augmented into the Digital Banking services which help to reduce dependence on paper. These services include eco-friendly digital initiatives like Internet Banking, Mobile Banking, SMSs etc. Recently, MCB Bank has introduced the Roshan Digital Account (RDA), a digital solution for on-boarding Non-Resident Pakistanis (NRPs). The Bank is offering e-Statement and E-Alerts, and the Direct Fund Transfer facility has also been enabled for Credit Card customers.

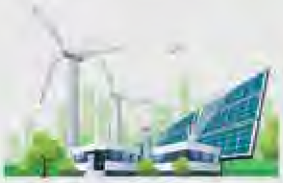

Renewable Energy Initiative

MCB Bank has installed solar power systems in some Branches and ATMs to avoid Greenhouse Gas (GHG) emissions. These solar installations augmented clean energy in energy mix and ultimately avoided approximately 13 tons of carbon dioxide (CO_2) emissions as of December, 2020 to the environment.

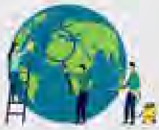

Environmental Risk Management System

Environmental Risk Assessment of customers who approach the Bank for a credit facility is now part of the credit approval process, and this assessment is carried out on an ongoing basis at the time of annual renewal of a customer's credit package.

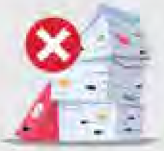

Paperless Operations

MCB Bank is opting for ways and mechanisms to reduce the consumption of paper and other associated resources through automation of paper-based workflows and processes. Few initiatives undertaken for reducing paper consumption include but are not limited to suspension of paper-based internal circulars dissemination, introduction of web-based dispute claim filing utility for all Alternate Delivery Channels and deployment of application-based workflows system for gradual migration towards paperless environment. Furthermore, the Bank is also implementing a value-added feature to its ATMs which will provide an option to the customers for making financial transactions without printing receipts.

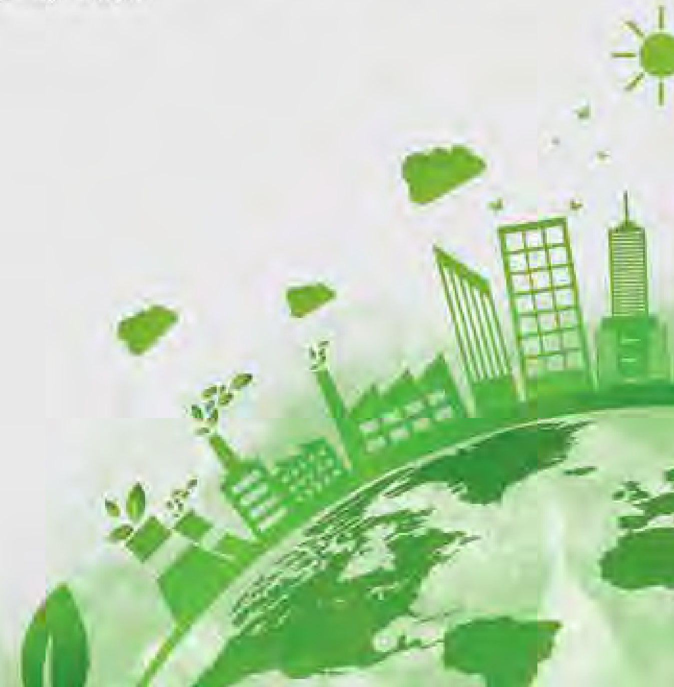

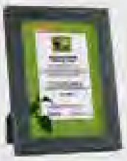

World Wide Fund for Nature (WWF) - Green Office Certification

MCB Bank has successfully obtained WWF - Green Office Certification for one of its iconic buildings - MCB Centre, Lahore. This is a landmark achievement and makes MCB Bank one of the few Banks who have successfully met the requirements of this rigorous assessment and certification program.

A WWF certified Green Office is a workplace that reduces its burden on the environment, achieves savings and slows down climate change by reducing its carbon dioxide (CO_2) emissions. A building-specific Environmental Management Plan (EMP) had been implemented to achieve the Green Office Certification.

The implementation of EMP helps to cut down on energy/electricity, fuel, paper and recycling of dry waste generated from the building. The engineering and administrative controls enabled to reduce approximately 90 tons of CO_2 emissions during 2020 as compared to the previous year.

As part of the certification process, WWF undertook a detailed certification audit to check the resource conservation measures implemented in the building. MCB Bank satisfactorily met all the set requirements for MCB Center to be declared as Green Office.

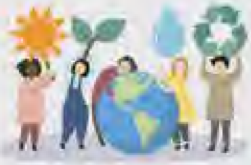

Green Awareness Campaign

A Green Awareness Campaign regarding environment-related annual days such as World Water Day, Mother Earth Day and World Environment Day has been commemorated to alert employees and customers about the significance of the environment. The commemoration includes different dissemination mediums such as artwork-based emails, standees displayed at different buildings of the Bank and web-banners displayed on MCB Bank's corporate website. Furthermore, employee's perception has been developed on "Own Impact Reduction Measures" through electronic dissemination of specially designed artworks regarding usage of electricity, water, paper, fuel etc. at a regular frequency to instill the practice of resource conservation in their daily lives.

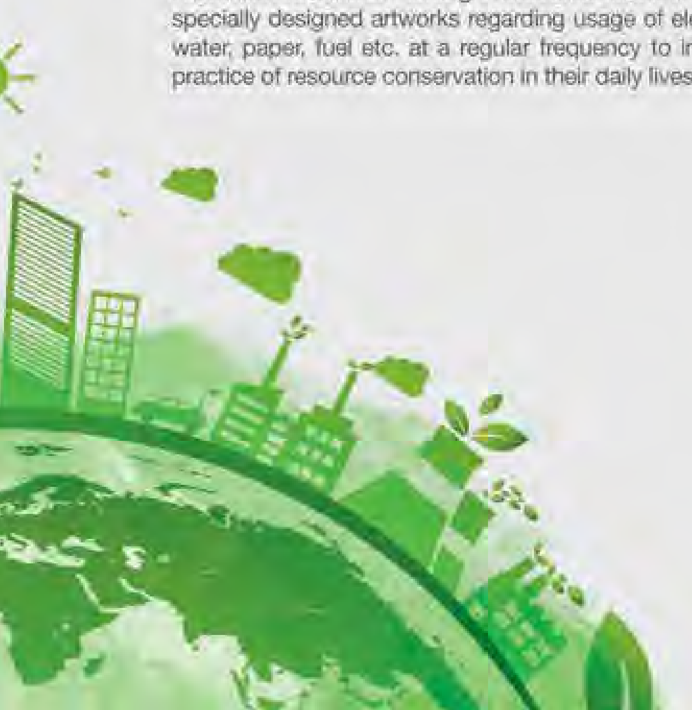

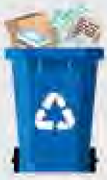

Dry Waste Management

MCB Bank has partnered with a civil service organization which provides services of waste collection and recycling through its green partner network. As a pilot project, the dry waste generated from two iconic buildings in Lahore which weighs approximately 8.3 tons is collected and recycled in an environment-friendly manner. The waste is being reused as raw material for other industries which ultimately reduces the burden on landfill sites and the environment.

Green points were allocated against each KG of recycled waste, which were redeemed by procuring environmentally-friendly products. The allocated green point for each product includes a significant social contribution for training and empowering of youth and women.

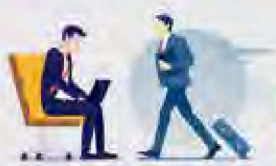

Reduction of Business Travel

MCB Bank has significantly reduced business-related travel, especially during the COVID-19 situation, moving towards effective use of video conferencing/telepresence technology. These virtual meetings save time, money and other associated resources, resulting in lesser carbon footprint as compared to travel-based meetings.

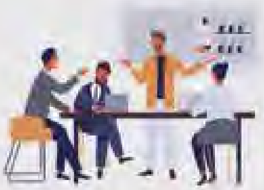

Employees Capacity Building

COVID-19 pandemic has provided us with an opportunity to innovate and adopt sustainable means to learn and impart knowledge to employees by keeping them abreast of COVID-19 advisories/circulars digitally during lockdown situations. The Green Banking and other trainings are provided through e-learning portal which conserves substantial resources including paper, printing, electricity, fuel etc. and ultimately reduces carbon footprint to environment as compared to room-based trainings.

Green Banking trainings have become the major driver to sensitize the staff regarding environmental implications and adoption of the Green culture across the Bank. 2,189 personnel have successfully received training on Green Banking during 2020.

MCB
Bank for Life

Stakeholder Relationship & Engagement

The development of sustained stakeholder relationships is paramount to the performance of any institution. From short term assessments to long-term strategic relationship building, 'Stakeholders' Engagement' lies at the core of our business practices to promote improved risk management, compliance with regulatory and lender requirements in addition to overall growth of the Bank.

In achieving and entrenching its integrated approach to sustainability, MCB Bank takes a highly collaborative approach towards ensuring maximum interaction with, and input by all its stakeholders.

At MCB, stakeholder engagement involves far more than merely communicating with its various stakeholder groups. The Bank regards its stakeholders as partners and makes every effort to use all possible mediums to ensure that they are abreast with disclosures, aware of forums to provide valuable input and feedback that can help the Bank to grow, strengthen relations and meet expectations to serve better.

Stakeholder Engagement Process and Frequency

The following tables provide an overview of stakeholder engagements at MCB Bank

Stakeholders	Stakeholder worthiness and reasons of engagement	Engagement frequency	Mode of consultation / interaction
Institutional Investors / Shareholders / Analysts	• To deliver relevant and timeous information to existing and potential shareholders<br>• To keep shareholders posted to ensure that our shares are traded at a fair value<br>• To ensure that the image of the bank and the trust placed in by our valuable shareholders, continues to improve, thereby minimizing the potential for reputational risks	Quarterly, semi-annually and annually<br><br>When the need arises	• Quarter, semi-annual and annual financial statements<br>• Annual General Meeting<br>• Participation in local and international road shows<br>• Press releases / Media announcements<br>• Communications and answering investor / analyst questions
Employees	• Integral to deliver strategic objectives<br>• Our most important and valued ambassador<br>• To ensure that we remain an employer of choice by providing a safe, positive and inspiring working environment<br>• To understand and respond the needs and concerns of our staff members<br>• To educate our staff regarding strategic direction and to communicate the pertinent information relating to bank activities<br>• Continuously encouraging employees and working towards creating a healthy, ethical and supportive work environment	Annual<br><br>When the need arises	Investing in a wide range of training programs for every member of the human capital to ensure personal and professional development.<br><br>In addition to the regular communication that takes place with direct managers and teams through a range of interactive channels, specific employees engagement include:<br>• Regular electronic and printed newsletters<br>• Compliance letters<br>• Annual conference<br>• Strategy sessions<br>• Grievance reporting procedure<br>• One Bank, One Team sessions with senior management

Stakeholders	Stakeholder worthiness and reasons of engagement	Engagement frequency	Mode of consultation / interaction
Customers	• To win and maintain customers by developing and providing products and services to improve the brand.<br>• To understand the growing financial services needs of our customers.<br>• To provide better solution and advice to our customers' financial requirements<br>• To ensure accuracy of our customers respective information.<br>• Integral to achieve strategic objectives	Regular interaction of customers through branch staff<br><br>Dependent on customers' specific requirements	• Spreading the geographical boundaries through opening more branches across the country.<br><br>• Continuously innovating in product suite and operational process to meet customer requirements in the most efficient manner.<br>• Interaction through our branches, relationship managers, call centers, social media, surveys and various advertising activities
Suppliers/Service Providers	• Adhere to proper procurement regulations while maintaining a good business relationships with the service providers	Routine basis/ When the need arises	• Rigorously following internal procurement policy and upgrading the policy regularly to ensure strong control and fair treatment of suppliers.
Regulator	• To maintain open, honest and transparent relationships with regulator<br>• To ensure meticulous compliance with legal and regulatory requirements<br>• Develop legislation and policies that impact the environment in which we operate	Daily, weekly, quarterly<br><br>When the need arises	• Active engagement with regulator improves level of compliance these engagements include meetings with representative of regulator and written communications on need basis<br>• One-on-one Meetings<br>• Submission of applicable statutory returns Responding / enquiring various queries / information

Stakeholders	Stakeholder worthiness and reasons of engagement	Engagement frequency	Mode of consultation / interaction
Communities	• To have best collaboration with our community for delivering our social responsibilities<br>• To obtain input from communities regarding key focus areas<br>• To create awareness of our integrated sustainability commitments and initiatives<br>• Conducting business without causing disruptions in the society	When the need arises	The Bank actively participates in various social work initiatives as part of its corporate social responsibility. Being a conscientious member of the corporate community, the bank contributes to various social and charitable causes including towards health, education and social sectors.<br><br>The bank is consistent in support for community development projects and interaction with a wide range of non-profit organizations.<br><br>Enhancing financial access to marginalized population and most importantly, adding value to the society by being a good and transparent corporate citizen.
Government	• To build strong and constructive relationship with government, both as a partner in the development of our country and as a current / potential client<br>• To contribute in legislative development for evolution in our activities and operations<br>• To endorse our commitments for public sector business development	When the need arises or on request by either side	• Understanding and ensuring all legal and regulatory requirements are complied with<br>• Engaging with the government to address matters impacting business
Media	• To acknowledge the role of media as a channel to communicate with relevant stakeholders and public at large	When the need arises	• Advertisements through print, electronic, social media, website, interviews and capacity building seminars

Investor Relation Section on Corporate Website

The management of the Bank provides equal and fair treatment to all investors/shareholders through transparent investor relations, increased awareness, effective communication, and prompt resolution of investors'/ shareholders' complaints.

The Bank disseminates information to its investors and shareholders through a mix of information exchange platforms, including its corporate website, maintained in both English and Urdu Languages under the applicable regulatory framework. The website is updated regularly to provide detailed and latest Bank information including but not limited to financial highlights, investor information, dividend and other requisite information besides the link to SECP's investor education portal, the 'Jamapunji'. The "Investor Relations" section is also maintained on MCB website www.mcb.com.pk to promote investor relations and facilitate access to the Bank for grievance / other query registration.

Steps to Encourage Minority Shareholders Participation in AGM

Apart from being an event for decision making on important matters, General Meeting also provides a forum for two-way engagement with the shareholders, particularly the minority shareholders. Therefore, the Bank takes the following measures to ensure meaningful participation of minority shareholders in General meetings:

- Notice of General meetings is sent to every member of the Bank at least 21 days before the meeting. The notice is also published in newspapers (both English & Urdu) having nationwide circulation. Moreover, the notice is also circulated from the forum of Pakistan Stock Exchange.
- The Bank timely updates its website with respect to all notices of general meetings.
- Annual Report of the Bank is sent to each member of the Bank before Annual General Meeting (AGM)
- All members are entitled to appoint proxy to attend, speak and vote at the meeting on his/her behalf.
- Members can attend and participate in the AGM through video-link.
- During AGM, a detailed briefing on the Bank's performance and future plans is given to the shareholders in both Urdu and English
- The shareholders are encouraged to raise queries and give suggestions relating to the Bank's operations.

Stakeholder Engagement Policy & Summary of the Analyst Briefings

The Bank believes in frequent and transparent interaction with the stakeholder to share financial performance, future outlook, regulatory and economic environment. Such interactions facilitate in building the stakeholder's confidence in the institution.

Analyst briefings are interactive sessions between the management of the Bank and the investor community whereby the Bank takes the opportunity to apprise the local and foreign investors about the business environment and economic indicators of the country, explain Bank's financial performance, competitive environment investment decisions, challenges faced as well as business outlook.

The idea behind the Bank's investor engagement through these briefings is to give the right perspective of the business affairs of the Bank to the investors (both existing and potential) which help them in making their investment decisions.

The Bank conducts quarterly analyst briefings in order to share details pertaining to results announced and to respond to any queries of analysts relating to results and future prospects.

Other than the quarterly analysts briefing, business analysts are provided with information and briefings as and when they require without compromising the confidentiality. Face to face discussions have also been arranged with foreign analysts as and when required. The briefing further envisages our transparent and continuously evolving stakeholders' engagement approach.

Briefing is being held as teleconferencing and during the year four analysts briefing were held on following dates;

Results	Date
Annual Results 2019	February 17, 2020
1st Quarter Results 2020	May 4, 2020
2nd Quarter Results 2020	August 25, 2020
3rd Quarter Results 2020	November 11, 2020

In addition to the above mentioned regular teleconferencing sessions, during the year Bank also held Corporate Analyst Briefing Session on November 11, 2020 using the online meeting platform. Session was attended by senior management of MCB Bank Limited and various analysts and investors. CFO of the bank presented a detailed analysis of Bank's performance along with future outlook; session was followed by Q & A session.

Issues Raised in the Last Annual General Meeting (AGM)

The proceedings of the 72nd Annual General Meeting of the shareholders of MCB Bank Limited was held on Thursday, March 19, 2020 at 11:00 am, Nishat Hotel, Emporium Mall, Lahore. Amidst the threat posed by the ongoing COVID-19 outbreak, the proceedings of the meeting were also covered through a video link in order to avoid crowd gathering in accordance with the directives of the Government.

Mian Mohammad Mansha, the Chairman of the Board of Directors, presided over the meeting. The meeting was attended by Board members of the Bank including the Chief Executive Officer along with the Chief Financial Officer and the Company Secretary; either in person or through a video link.

The company secretary invited the CFO to present key highlights of the audited financial statements for the year 2019 and elucidate on the salient features of the Banks' performance. There were no significant issues raised in the last AGM that are pending implementation, however, the shareholders while appreciating the overall growth in Bank's financial performance sought some general clarifications and comments on the progress of the Bank.

One of the shareholders enquired about the reasons for increase in tax charge for the year 2019. Responding to the shareholders query, the CFO apprised the forum that in addition to increased profitability, Super Tax charge, which was initially levied vide Finance Act, 2015 had continued and was extended vide Finance Act 2019 with retrospective effect thereby causing recognition of an additional charge for the tax year 2018 in 2019.

Another shareholder enquired about the expected impact of COVID-19 outbreak on the Bank's financial performance and its operating environment in general. The CFO replied that while the management was proactively monitoring the evolving scenario and the Bank remained well poised to respond to the emanating risks, the ultimate impact from the outbreak still remained extremely uncertain and largely dependent on the pandemics' pathway and ramifications of the policy measures adopted by regulatory, monetary and fiscal authorities for combating the outbreak.

The CEO also addressed the forum and informed the shareholders that the Bank remained a well-capitalized institution with a capital base well above the regulatory limits and Basel capital requirements. He mentioned that the Bank had the highest cash dividend per share in the industry and also remained one of the prime stocks traded in the Pakistani equity markets reflected by its highest market capitalization in the financial institution category as at December 31, 2019.

The shareholders appreciated the financial performance of the Bank and congratulated the Board of Directors and the management for the same.

Following businesses were also discussed during the AGM:

- Consideration and approval of Annual Accounts of 2019
- Approval for alteration in the Articles of Association of the Bank
- Approval for scale of remuneration including additional remuneration for Board of Directors
- Approval of final cash dividend – 2019
- Appointment of External Auditors

Statement of Value Added

	2020 PKR (mln)	%	2019 PKR (mln)	%
**Value Added**				
Net interest income	71,334		59,616	
Non interest income	18,136		16,679	
Operating expenses excluding staff costs, depreciation, amortization and WWF	(13,455)		(14,897)	
Provision against advances, investments & others	(7,313)		(2,484)	
Value added available for distribution	68,702		58,914	
**Distribution of value added:**				
**To employees**				
Remuneration, provident fund and other benefits	15,806	23.01%	14,585	24.76%
**To government**				
Income tax	19,212	27.96%	16,125	27.37%
**To shareholders**				
Cash dividends to shareholders	23,701	34.50%	20,146	34.20%
**To Society**				
Donations	113	0.16%	–	0.00%
**To expansion and growth**				
Depreciation, Amortization, Retained earnings and reserves	9,871	14.37%	8,057	13.68%
	68,702	100%	58,914	100%

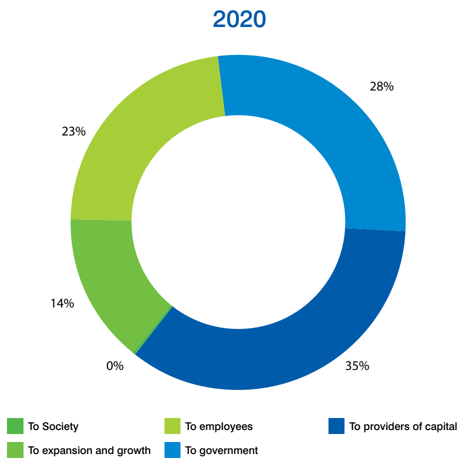

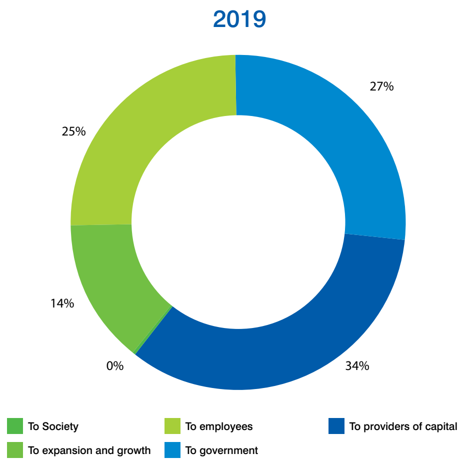

Code of Conduct

- The Code of Conduct spells out the behaviour expected from employees of MCB Bank Limited (MCB), reflecting fairness, transparency and accountability. The Code of Conduct gives a quick reference check for acceptable business practices.

- However, the Code of Conduct does not replace defined and comprehensive HR Policies of MCB Bank Limited.

- MCB Bank is committed to conduct its business in accordance with the applicable laws, rules and regulations as defined by the State Bank of Pakistan by adhering to high standards of business ethics which reflect our corporate values.

- Adherence to the Code of conduct is mandatory for all employees of MCB Bank Ltd-Pakistan.

In line with code of conduct the employees of the bank shall

Abidance of Laws / Rules

- Conform to and abide by the Bank rules and policies, wherever we operate and obey all lawful orders and directives which may from time to time be given by any person or persons under whose jurisdiction, superintendence or control, the persona will, for the time being, be placed. To undertake at all times compliance with and observation of all applicable laws, regulations and Bank policies, wherever the Bank operates.

Integrity

- Conduct the highest standards of ethics, professional integrity and dignity in all dealings with the public, customers, investors, employees, and government officials, State Bank of Pakistan and fellow Bankers and non-engagement in acts discreditable to the Bank, profession and nation

- In case of awareness of any breaches of laws and regulations, frauds and other criminal activities or other similar serious incidents that might affect the interests of the Bank, the same shall be informed to the senior management immediately, including any issue, which may pose a reputational risk.

- Not use this policy to raise grievances or act in bad faith against colleagues.

Professionalism

- Serve the Bank honestly and faithfully and strictly serve the Bank affairs and the affairs of its constituents, use utmost endeavor to promote the interest and goodwill of the Bank and show courtesy and attention in all transactions/ correspondence with officers of Government, State Bank of Pakistan, other Banks & Financial Institutions, other establishments dealing with the Bank, the Bank constituents and the public.

- In case the employment is terminated for any reason, all rights to property and information generated or obtained as part of employment relationship will remain the exclusive property of MCB.

- Comply with the laws and regulations on money laundering and fraud prevention and immediate reporting of all suspicions of money laundering as per the guidelines provided in CDD & AML/ CFT Handbook and Anti-fraud Framework Policy for the Management and the staff.

- Not to engage in any act of violation of CDD & AML / CFT Handbook's guidelines given by the State Bank of Pakistan and be extremely vigilant in protecting MCB Bank from being misused by anyone to launder money by violating these guidelines.

- Ensure that all customer complaints are resolved quickly, fairly and recorded appropriately.

Conflict of Interest

- Avoid all such circumstances in which there is personal conflict of interest, or may appear to be in conflict, with the interest of the Bank or its customers.

- In case of potential conflict of interest, the same should be declared immediately to senior management, action is taken to resolve and manage it in open manner and resolving the conflict of interest on their own would be avoided.

- Report to the Company Secretary within three (3) days about any sale and purchase of MCB shares (own or spouse) in case the annual basic salary exceeds Rs. 500,000/-.

- Not buy, sell or take position in any manner regarding MCB Bank shares during Closed Period, as announced by Company Secretary.

Relatives and close friends

- Avoid conflict of interest arising, where an employee makes or participates in a decision which affects another person with whom one has a personal relationship (such as a relative, parent, spouse, cousin, close friend or personal associate). In cases where a conflict may arise, employees must advise their immediate line manager. Wherever possible, employees should disqualify themselves from dealing with those persons in such situations.

Code of Conduct

Political Participation

- Not obtain membership of any political party, or take part in, subscribing in aid of, or assist in any way, any political movement in or outside of Pakistan or relating to the affairs of Pakistan.

- Not express views detrimental to the ideology, sovereignty or integrity of Pakistan.

- Not canvass or otherwise interfere or use influence in connection with or take part in any election as a candidate to a legislative/local body or issue an address to the electorate whether in Pakistan or elsewhere. However, the right to vote can be exercised.

- Not bring or attempt to bring political or other pressure/ influence directly or indirectly to bear on the authorities/ superior officers or indulge in derogatory pamphleteering, contribute, or write letters to the newspapers, anonymously or in own name contribute or appear in media, with an intent to induce the authority/ superior officers to act in a manner inconsistent with rules, in respect of any matter relating to appointment, promotion, transfer, punishment, retirement or for any other conditions of service of employment.

Financial Interest

- Not indulge in any of the following activities without prior permission of competent authority (GH - HRM for VP & below and President for SVP & Above):

- Borrow money from or in any way place myself under pecuniary obligation to a broker or moneylender or anyone, including but not limited to any firm, company or person having dealings with the Bank.

- Buy or sell stock, shares or securities of any description without funds to meet the full cost in the case of purchase or scripts for delivery in the case of sale. However, a bona-fide investment of own funds in such stocks, shares and securities as wished can be made.

- Lend money in private capacity to a constituent of the Bank or have personal dealings with a constituent in the purchase or sale of bills of exchange, Government paper or any other securities.

- Act as agent for an insurance company otherwise than as agent for or on behalf of the Bank.

- Be connected with the formation or management of a joint stock company or hold office of a director.

- Engage in any other commercial business or pursuit, either on own account or as agent for another or others.

- Engage in any outside employment or office whether stipendiary or honorary during my employment with MCB Bank.

- Undertake part-time work for a private or public body or private person, or accept fee thereof.

- Any kind of trading advice concerning the securities of MCB Bank or to third parties even when such director, officer or employee does not possess material nonpublic information about MCB Bank.

- In reviewing or approving a loan application from a corporation wherein holding office as director, partner or guarantor.

Gift, Favors Etc.

- Not use the employment status to seek personal gain from those doing business or seeking to do business with MCB, nor accept such gain if offered.

- Not accept any gift, favors, entertainment or other benefit the size or frequency of which exceeds normal business contacts from clients, stakeholders, colleagues of the Bank or from persons likely to have dealings with the Bank including candidates for employment in the Bank.

- Reporting in writing to immediate supervisor within three working days in case any sizeable gift / favor is received from any third parties.

Confidentiality

- Maintain the privacy and confidentiality (during the course of employment and after its termination for whatever reason), of all the information acquired during the course of professional activities and refrain from disclosing the same unless otherwise required by statutory authorities / law. Inside information about Bank's customers/affairs including customer data, product manuals, confidential financial and business information of the Bank etc., shall not be used for own gain or for that of others either directly or indirectly.

- Not trade in relevant investments or indulge in giving tips to another person or dealing on behalf of relatives, friends or any other third parties, whilst in possession of non-public price sensitive information.

- Not disclose to a customer or customers or to any irrelevant quarter(s) that a suspicious transaction or related information is being reported for investigation unless any law enforcement agency requires any lawful information. (Only authorized representatives can pass on information to Law enforcing agencies after obtaining clearance on information content from relevant GH / BH(for RBG) and LAG representative).

Code of Conduct

Data Security

- Only access or update the system and data according to the authority given by the Bank. Any unauthorized access or updation will hold the person liable for a penal action by the Bank in accordance with HR policies.

- Not compromise access to system by communicating identification and /or passwords to others.

- Ensure that material non-public information is secure. Not discuss such information in public places where it can be overheard, such as elevators, restaurants, taxis and airplanes.

Communication / Contact with Media

- Be truthful in all advertisings and promotional efforts and to publish only accurate information about the Bank operations under valid authority as prescribed in the Bank policy.

- Not give any kind of confidential information or interview on behalf of the Bank or in my official capacity in the print/electronic media or road / talk shows or participate or act in television/stage plays or in any media or cinema without having permission from the Head of Corporate Communication & GH - HRM.

Speak Up

- To inform line management & HR of any perceived wrong doing / malpractice at any level, as an obligation to report it under the Bank whistle blowing program / policy.

Business / Work Ethics

- Respect fellow colleagues and work as a team. To be, at all times, courteous and not to let any personal differences affect work.

Customer Centricity

- Treat every customer of the Bank with respect and courtesy.

- Be responsive to customer complaints, and to feedback on products and services.

- Provide relevant, complete and clear information to customers to the best of one's knowledge.

- Sell products or services to customers that are within the legitimate scope of one's job.

- Remain update with the latest products of the Bank, and provide all relevant information to the customers.

Personal Responsibility

- Safeguard as a personal responsibility, both the tangible and intangible assets of MCB and its customer(s) that are under personal control and not to use Bank assets for personal benefits except where permitted by MCB.

- Not use any Bank facilities including a car or telephone to promote trade union activities, or carry weapons into Bank premises unless so authorized by the management, or to carry on trade union activities during office hours, or in banking premises, or subject Bank officials to physical harassment or abuse.

Punctuality

- Ensure attendance and punctuality as per HR policies, departmental requirements & job standards and for any absence during working hours obtain written permission of the immediate supervisor.

Dress Code

- Maintain a standard of personal hygiene / neatness and follow MCB Bank dress code policy in true spirit to promote a professional work environment during office hours.

International Travel

- Be culturally sensitive to the socio-cultural norms of the host country.

- Represent Country and organization by conforming to high standards of personal and professional ethics at all times.

Work Environment

- Cooperate in maintaining a healthy and productive work environment and not get engaged in the selling, manufacturing and distributing using any illegal substance or being under the influence of illegal drugs or alcohol while on the job.

- Ensure strict adherence to all policies of the Bank, as announced by the management from time to time and contribute utmost effort in maintaining a conductive work environment.

Usage of Communication Tools

- Ensure strict adherence to the use of internet, emails and telephone provided by the Bank for official use only.

- Never use the Bank system to transmit or receive electronic images or text containing ethnic slurs, social epithets or anything that might be construed as harassing, offensive or insulting to others.

Code of Conduct

- Never utilize Bank system to disseminate any material detrimental to the ideology, sovereignty or integrity of Pakistan.

- Never indulge and /or utilize the Bank system for supporting any terrorist activity within and / or outside Pakistan.

Reporting and Accountability

- Maintain all books, data, information and records with scrupulous integrity, reflecting in an accurate and timely manner and to ensure that all business transactions are reported and documented correctly according to the business practices. Ensure facts are not misinterpreted / misused /tampered pertaining to:

- Issuing an incorrect account statement / any other information for any customer or fellow employees / management.

- Placing a fake claim for reimbursement of any expenses (including medical insurance).

- Unrecorded or recorded funds / assets or any other Bank's documents.

- Posting of false, artificial or misleading entries in the books or record of the Bank.

- Intimate line management and HRM of any changes in the personal circumstances relating to service tenure and other related benefits, provided by the Bank.

Ethics for working with Female Employees

Gender Discrimination In Employment Training / Promotion

- Ensure adherence to the guidelines of MCB Bank's non-discrimination on the basis of gender which limits the individual's right of recruitment, future training, promotion and any other related benefits.

Zero Tolerance for Favoritism or Discrimination

- Not be a part of any undue favor / discriminatory advantage to any colleague / subordinate staff.

Personal Space

- No right to intrude on the personal space / close proximity of any staff particularly females.

Female Staff/Employee Privacy

- Recognize that female staff have more privacy and sensitivity needs in keeping with our cultural norms. Therefore, behaviour towards them must reflect that sensitivity, respect and consideration.

Harassment Against Women

- Any type of harassment is not acceptable at MCB Bank. Harassment occurs when someone's actions or words, based on gender, race, sexuality, caste, creed, and color are unwelcome, violate another person's dignity and creates a hostile environment. (Reference to HR Policy Manual section 6, 6.2.5, Protection against harassment of women at the workplace Act, 2010)

- Accordingly, not engage in harassment in any form. It may include objectionable epithets, threatened or actual physical harm and intimidating conduct directed against the individual that negatively affects the performance and well-being of an individual.

Sexual Harassment

- Keeping in mind the Bank's policy with reference to the Protection against harassment of Women at the Workplace Act, 2010, adherence to all guidelines given by the Bank.

Communication

- Not send sexually explicit or offensive communications and respect the privacy of fellow employee especially female employees. Following factors should be adhered to in order to maintain effective communication and ethical standards:

  1. Not send any electronic mail that is abusive or threatens the safety of an Individual(s).
  2. Always use a professional tone in all official communications.
  3. Be careful when using sarcasm and humor

Workplace bullying

Refrain from any form of Workplace bullying:

- Shouting or swearing at an employee or otherwise verbally abusing him / her.

- Singling out an employee for excessive criticism/public humiliation.

- Excluding an employee from company activities and undermining his / her work contributions.

- Language or actions that embarrass or humiliate an employee.

- Inappropriate practical jokes, especially if they are targeted.

Insider Trading

Comply with insider trading policy and to abide by all guidelines provided in the policy.

Statement on Internal Controls

The internal control structure of MCB Bank Limited (Bank) comprises the Board of Directors, Senior Management, Risk Management Group, Financial Control Group, Operations Group, Compliance & Controls Group, Audit & Risk Assets Review (Audit & RAR) Group, Internal Control Units (ICUs) within all Groups and the controls & self-assessment procedures implemented at other functions within the Bank. The Bank's management is responsible to establish and maintain an adequate and effective system of internal controls and procedures under the policies approved by the Board. The management is also responsible for evaluating effectiveness of the Bank's internal control system that covers material matters through identification of control objectives as well as review of significant policies and procedures.

Bank's internal control system has been designed to identify and mitigate the risk of failure to achieve overall business objectives of the Bank. Internal controls and policies are designed to provide reasonable assurance regarding the effectiveness and efficiency of the Bank's operations, reliability of financial information and compliance with applicable laws & regulations. However, it needs to be stated that systems are designed to manage, rather than eliminate the risk of failure to achieve the business objectives and can only provide reasonable and not absolute assurance against material misstatement or loss.

The management of the Bank has adopted the Integrated Framework on Internal Controls issued by the Committee of Sponsoring Organizations of the Treadway Commission (COSO) and has completed all the stages as set out in the roadmap provided by the State Bank of Pakistan (SBP) through the Guidelines on Internal Controls. Bank's assessment included documenting, evaluating and testing of the design and operating effectiveness of its Internal Controls over Financial Reporting (ICFR). Bank has developed a management testing and reporting framework for monitoring ongoing operating effectiveness of key controls.

Concerted efforts are made by every Group to improve the control environment at grass root level by regularly reviewing and streamlining procedures to prevent and rectify control lapses as well as imparting training for improvement at various levels. Compliance & Controls Group (CCG), through its specialized teams and centralized automated solutions, also oversees adherence to the regulatory requirements, with specific emphasis on Anti-Money Laundering (AML) / Combatting the Financing of Terrorism (CFT) / Countering Proliferation Financing (CPF). In addition, CCG also leads the Management's Committee on AML/CFT/CPF for oversight of AML/CFT/CPF compliance with respect to relevant laws, regulations, policies and procedures.

The scope of Audit & RAR Group, independent from the management, inter alia includes, review and assessment of the adequacy and effectiveness of the control activities across the Bank as well as evaluation of compliance with the Bank's prescribed policies and procedures. All significant / material findings of the internal audit activities are reported to the Board's Audit Committee. The Audit Committee actively monitors implementation of internal controls to ensure that identified risks are mitigated to safeguard interest of the Bank.

All significant and material findings of the internal and external auditors as well as observations of the regulators are addressed on priority by the management and their status is reported periodically to the Board's Audit Committee and the Board's Compliance Review & Monitoring Committee respectively, which ensures that the management takes appropriate corrective actions and put in place a system to minimize repetition for strengthening of the control environment.

Senior management team, through different Management Sub-Committees, monitor resolution / compliance of issues identified by the Regulators, Statutory Auditors as well as Audit & RAR Group. Periodic meetings of these Management Sub-committees are held to ensure expeditious resolution / compliance of aforementioned issues. The Management Committee chaired by the Bank's President / Chief Executive Officer monitors the performance of sub-committees on annual basis.

In accordance with SBP's directives and as stated earlier, the Bank has completed all stages of ICFR roadmap and a Long Form Report (LFR) on the assessment of Bank's ICFR for the year 2019 issued by the statutory auditors has been submitted to SBP in compliance with its directives stated in OSED Circular No. 1 of 2014 dated February 07, 2014. None of the deficiencies identified had a material impact on Financial Reporting.

Based upon the results derived through ongoing testing of financial reporting controls and internal audits carried out during the year, the management considers that the Bank's existing internal control system is adequate and has been effectively implemented and monitored. The management will continue enhancing its coverage and compliance with the SBP Guidelines on Internal Controls and further strengthening its control environment on an ongoing basis.

Based on the above, the Board of Directors has duly endorsed management's evaluation of internal controls including ICFR in the Directors' report.

Farid Ahmad
Chief Compliance Officer

Kamran Zaffar Muggo
Group Head Operations

Hammad Khalid
Chief Financial Officer

Muhammad Farooq Wasi
Chief Internal Auditor

Statement of Compliance with Listed Companies
(Code of Corporate Governance) Regulations, 2019

MCB Bank Limited
For the year ended December 31, 2020

MCB Bank Limited ("MCB" or the "Bank") has complied with the requirements of the Listed Companies (Code of Corporate Governance) Regulations, 2019 (the "Regulations") in the following manner:

1. The total number of Directors including President & CEO are 13 as per the following:

   a. Male: 12
   b. Female: 01

2. The composition of the Board is as follows:

Category	Name
**Independent Directors**	Mr. Yahya Saleem<br>Mr. Salman Khalid Butt<br>Mr. Shahzad Hussain<br>Mr. Masood Ahmed Puri
**Non-Executive Directors**	Mian Mohammad Mansha<br>Mr. S. M. Muneer<br>Mr. Muhammad Tariq Rafi<br>Mian Umer Mansha<br>Mr. Mohd Suhail Amar Suresh bin Abdullah<br>Mr. Muhammad Ali Zeb<br>Mr. Shariffuddin Bin Khalid
**Executive Director (President & CEO)**	Mr. Imran Maqbool
**Female Director (Non-Executive)**	Mrs. Iqraa Hassan Mansha

   The Bank has twelve (12) elected directors and one CEO who is a deemed director. The fraction of 0.33 is below half (0.50) and accordingly, the same has not been rounded up as one.

3. The directors have confirmed that none of them is serving as a director on more than seven listed companies, including the Bank;

4. The Bank has prepared a 'Code of Conduct' and has ensured that appropriate steps have been taken to disseminate it throughout the Bank along with its supporting policies and procedures;

5. The Board has developed a vision/mission statement, overall corporate strategy and significant policies of the Bank. The Board has ensured that complete record of particulars of significant policies along with their date of approval or updating is maintained by the Bank;

6. All the powers of the Board have been duly exercised and decisions on relevant matters have been taken by the Board/ Shareholders as empowered by the relevant provisions of the Companies Act, 2017 (the "Act") and the Regulations;

7. The meetings of the Board were presided over by the Chairman and, in his absence, by the Vice Chairman or a director elected by the Board for this purpose. The Board has complied with the requirements of Act and the Regulations with respect to frequency, recording and circulating minutes of meeting of the Board;

8. The Board has a formal policy and transparent procedures for remuneration of directors in accordance with the Act and the Regulations;

9. All the Board Members except one either have minimum education and experience as required under Regulation 19 for exemption from Directors Training Program, or have already completed Directors Training Program pursuant to the requirements of the Regulations;

10. During the year, there was no any such appointment of the Chief Financial Officer ("CFO"), the Company Secretary and the Head of Internal Audit;

11. The CFO and the President & Chief Executive Officer ("CEO") duly endorsed the financial statements before approval of the Board;

12. The Board has formed its eight (8) Committees. The names and composition of Committees along with the details of Committee(s) Members have been given in the Section on Corporate Governance;

13. The Terms of Reference ("TORs") of the aforesaid Committees have been formed, documented and advised to the respective Committee for compliance;

14. The frequency of the Committee meetings has been given in the Section on Corporate Governance;

15. The Board has set up an effective internal audit function which is considered suitably qualified and experienced for the purpose and conversant with the policies and procedures of the Bank;

16. The Statutory Auditors of the Bank have confirmed that they have been given a satisfactory rating under the Quality Control Review program of the Institute of Chartered Accountants of Pakistan ("ICAP") and registered with Audit Oversight Board of Pakistan, that they and all their partners are in compliance with International Federation of Accountants ("IFAC") guidelines on code of ethics as adopted by the ICAP and that they and the partners of the firm involved in the audit are not a close relative (spouse, parent, dependent and non-dependent children) of the President & CEO, CFO, head of Internal Audit, Company Secretary or director of the Bank;

17. The Statutory Auditors or the persons associated with them have not been appointed to provide other services except in accordance with the Act, these regulations or any other regulatory requirement and the auditors have confirmed that they have observed IFAC guidelines in this regard; and

18. We confirm that all requirements of regulations 3, 6, 7, 8, 27, 32, 33 and 36 of the Regulations have been complied with.

For and on behalf of the Board of Directors

Imran Maqbool

Imran Maqbool
President & CEO

Mansha

Mian Mohammad Mansha
Chairman

MCB Bank Limited

February 10, 2021
Lahore

Independent Auditor's Review Report

To the members of MCB Bank Limited

Review Report on the Statement of Compliance contained in Listed Companies (Code of Corporate Governance) Regulations, 2019

We have reviewed the enclosed Statement of Compliance with the Listed Companies (Code of Corporate Governance) Regulations, 2019 (the Regulations) prepared by the Board of Directors of MCB Bank Limited ("the Bank") for the year ended 31 December 2020, in accordance with the requirement of regulation 36 of the Regulations.

The responsibility for compliance with the Regulations is that of the Board of Directors of the Bank. Our responsibility is to review whether the Statement of Compliance reflects the status of the Bank's compliance with the provisions of the Regulations and report if it does not and to highlight any non-compliance with the requirements of the Regulations. A review is limited primarily to inquiries of the Bank's personnel and review of various documents prepared by the Bank to comply with the Regulations.

As a part of our audit of the financial statements we are required to obtain an understanding of the accounting and internal control systems sufficient to plan the audit and develop an effective audit approach. We are not required to consider whether the Board of Directors' statement on internal control covers all risks and controls or to form an opinion on the effectiveness of such internal controls, the Bank's corporate governance procedures and risks.

The Regulations require the Bank to place before the Audit Committee, and upon recommendation of the Audit Committee, place before the Board of Directors for their review and approval, its related party transactions. We are only required and have ensured compliance of this requirement to the extent of the approval of the related party transactions by the Board of Directors upon recommendation of the Audit Committee.

Based on our review, nothing has come to our attention which causes us to believe that the Statement of Compliance does not appropriately reflect the Bank's compliance, in all material respects, with the requirements contained in the Regulations as applicable to the Bank for the year ended 31 December 2020.

KPMG Taseer Hadi & Co.
Chartered Accountants

Date: February 26, 2021
Lahore

MCB
Bank for Life

Report of the Audit Committee

The Audit Committee comprises four (4) non-executive directors including one Independent Director, one being Chairman of the Audit Committee. Further, two qualified chartered accountants with diversified professional experience in various sectors are members of the Audit Committee. Moreover, one of the members is fellow member of Chartered Institute of Management Accountants, (United Kingdom). The members of the Audit Committee are qualified professionals and possess enriched experience of working at the Boards & Senior Management levels of entities operating in both banking and non-banking sectors.

Role of audit committee to discharge its responsibilities towards financial statements and committee overall approach to risk management:

- In line with the requirements of Listed Companies (Code of Corporate Governance) Regulations – 2019 and Guidelines for Internal Audit Function issued by the State Bank of Pakistan, the Chief Internal Auditor functionally reports to the Board's Audit Committee and administratively to Bank's President / CEO. The Committee ensures staffing of the internal audit function with personnel of sufficient internal audit knowledge and experience, as well as equipping of the function with necessary resources and authority to execute their responsibilities independently and objectively.
- The committee approves and overseas the risk assessment, annual audit plan and related enablers/budget along with resource requirements of Audit & Risk Assets Review (Audit & RAR) Group.
- All significant and material findings of the internal audit activities are reported to the Audit Committee. The Audit Committee actively monitors implementation of internal controls to ensure that identified risks are mitigated to safeguard the interest of the Bank.
- Audit Committee actively engages in the review of the Bank's quarterly, half yearly and annual financial statements as well as oversight of internal audit activities in accordance with the requirements of Listed Companies (Code of Corporate Governance) Regulations – 2019 and the Charter of the Board Audit Committee, duly approved by the Board of Directors.
- The Committee understands its responsibility to ensure that the significant issues in relation to financial statements are addressed properly by debating and challenging the critical judgments and estimates made by the management. Furthermore, Audit & RAR group also reviews the Bank's quarterly, half yearly and annual financial statements and discusses the significant matters with management.
- The Committee ensures the independence of external auditor, effectiveness of external audit process and appointment/ reappointment of external auditor by performing the followings:
  - Review the terms of engagement and ensure that external auditor is independent to the Bank in terms of local regulatory requirements.
  - Ensure that external auditors have resources and professional qualification to conduct the audit.
  - The Auditors have been allowed direct access to the Audit Committee.
  - Discuss external auditors' feedback on the Bank's critical accounting estimates and judgments.
  - Discuss the significant control issues and significant audit matters identified by external auditors.

Audit Committee held five (5) meetings, during the year 2020, and following matters (including significant matters) were discussed:

- Review of the Bank's periodic financial statements, including disclosure of related party transactions prior to their approval by the Board of Directors (BOD).
- Review of status of compliance against observations highlighted by internal and external auditors, including regular updates on the rectification actions taken by the management in response to the audit findings.
- Review of status of implementation of decisions of BOD and its Sub-Committees.
- Review of significant issues (including critical repeated observation) highlighted by internal auditors during audits/ reviews of branches and other functions of the Bank along with management actions thereon.
- Review of analysis related to significant frauds and forgery incidents in the Bank, with specific focus on nature and reasons along with Management action(s) thereof. Review of annual fraud risk assessment along with action plan for strengthening of internal controls.
- Review, approval and oversight of Risk Assessment, Annual Audit Plan and related enablers/budget along with resource requirements of Audit & Risk Assets Review (Audit & RAR) Group.
- Review of status of trainings imparted to internal audit staff, along with status of activities under Quality Assurance & Improvement Program of Internal Audit.
- Review of resolution status of complaints lodged under the Bank's Whistle Blowing Program.
- Review of performance of Chief Internal Auditor against Key Performance Indicators (KPIs) for Audit & RAR Group. Review and approval of KPIs of Chief Internal Auditor for 2021.
- Review & approval of Audit Group's increments, bonuses, promotions and performance appraisal of Chief Internal Auditor.
- Review of progress on Audit Group's Strategic Initiatives and Milestones (2020 – 2022).

- Recommendation of scope and appointment of external auditors, including audit and consultancy fee. Audit Committee further ensured coordination between internal and external auditors.
- The Committee reviewed Annual Assessment by Audit & RAR Group on adequacy & effectiveness of Bank's processes for controlling and managing its risks in all core areas of the Bank's (including subsidiaries audited in year 2020) operations.
- The Committee reviewed annual confirmation regarding organizational independence of Audit & RAR Group.
- In addition to the above, the Committee also reviewed and recommended the following to the Board for approval:
  - Global Internal Audit Policy Version 3.0
  - Policy for Engagement of External Auditor on Providing Assurance and Non-Assurance Services of MCB Sri Lanka
  - IFRS-9 ECL Provisioning Policy MCB – Bahrain, Version 2.0
  - IFRS-9 ECL Provisioning Policy MCB – UAE Operations, Version 2.0
  - Investor Relations Policy - Ver. 3.0
  - Internal Audit Manual Version 8.0
  - Internal Audit Manual Sri Lanka Operations Version 4.0

Committee performance

Performance of the Audit Committee is annually reviewed by the Board of Directors and Board appreciated the Committee's role in thoroughly reviewing the financial statements and Bank's internal audit function and other financial matters of critical importance.

Internal Control Framework and role of Internal audit

The Bank's internal control structure comprises the Board of Directors, Senior Management, Risk Management Group, Compliance & Controls Group, Financial Control Group, the controls and self-assessment procedures implemented at other functions within the Bank; and Audit & Risk Assets Review (RAR) Group. The Management is responsible for establishing and maintaining a system of adequate and effective internal controls and procedures for implementing strategy and policies, as approved by the Board of Directors. The Bank has adopted integrated framework on Internal Controls issued by the Committee of Sponsoring Organizations of the Treadway Commission (COSO) and has completed all the stages, as set out in the roadmap provided by the State Bank of Pakistan (SBP) through the Guidelines on Internal Controls.

Audit & RAR Group has performed its role effectively on both assurance and consultative fronts. The Group played pivotal role in evaluating the efficacy of Bank's control systems and contributing towards their ongoing effectiveness by enhancing visibility of the Board and the management on the risk management and internal control matters of the Bank. All significant and material findings of the internal audit activities are reported to the Audit Committee of the Board of Directors. The Audit Committee actively monitors implementation of internal controls to ensure that identified risks are mitigated to safeguard the interest of the Bank.

The Audit Committee will continue to provide guidance to the Audit & RAR Group and the Management for further strengthening of Bank's risk management practices and internal control environment.

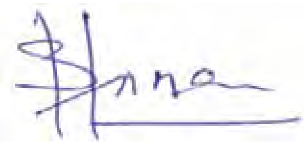

Shahzad Hussain
Chairman Audit Committee
MCB Bank Limited
Lahore

Unconsolidated
Financial Statements
MCB Bank Limited

Independent Auditor's Report

To the members of MCB Bank Limited

Report on the Audit of the Unconsolidated Financial Statements

Opinion

We have audited the annexed unconsolidated financial statements of MCB Bank Limited ("the Bank"), which comprise the unconsolidated statement of financial position as at December 31, 2020 and the unconsolidated profit and loss account, the unconsolidated statement of comprehensive income, the unconsolidated statement of changes in equity and the unconsolidated statement of cash flows for the year then ended, along with unaudited certified returns received from the branches except for 50 branches which have been audited by us and notes to the unconsolidated financial statements, including a summary of significant accounting policies and other explanatory information and we state that we have obtained all the information and explanations which, to the best of our knowledge and belief, were necessary for the purposes of the audit.

In our opinion and to the best of our information and according to the explanations given to us, the unconsolidated statement of financial position, the unconsolidated profit and loss account, the unconsolidated statement of comprehensive income, the unconsolidated statement of changes in equity and the unconsolidated statement of cash flows together with the notes forming part thereof conform with the accounting and reporting standards as applicable in Pakistan, and, give the information required by the Banking Companies Ordinance, 1962 and the Companies Act, 2017 (XIX of 2017), in the manner so required and respectively give a true and fair view of the state of the Bank's affairs as at December 31, 2020 and of the profit and other comprehensive income, the changes in equity and its cash flows for the year then ended.

Basis for Opinion

We conducted our audit in accordance with International Standards on Auditing (ISAs) as applicable in Pakistan. Our responsibilities under those standards are further described in the Auditor's Responsibilities for the Audit of the Unconsolidated Financial Statements section of our report. We are independent of the Bank in accordance with the International Ethics Standards Board for Accountants' Code of Ethics for Professional Accountants as adopted by the Institute of Chartered Accountants of Pakistan (the Code) and we have fulfilled our other ethical responsibilities in accordance with the Code. We believe that the audit evidence we have obtained is sufficient and appropriate to provide a basis for our opinion.

Key Audit Matters

Key audit matters are those matters that, in our professional judgment, were of most significance in our audit of the unconsolidated financial statements of the current year. These matters were addressed in the context of our audit of the unconsolidated financial statements as a whole, and in forming our opinion thereon, and we do not provide a separate opinion on these matters.

Following are the Key Audit Matters:

S. No.	Key Audit Matters	How the matter was addressed in our audit
1	**Provision against advances**	
	Refer to note 11 and the accounting policies in notes 4.2 (b) and 6.4 to the unconsolidated financial statements.<br><br>The Bank's advances to the customers represent 26.34% of its total assets as at 31 December 2020 and are stated at Rs. 462.94 billion which is net of provision of Rs. 50.61 billion at the year end.<br><br>The provision against advances was identified as a key audit matter in our audit as it involves a considerable degree of management judgment and compliance with the Prudential Regulations (PRs) issued by the State Bank of Pakistan.	Our audit procedures in respect of provision against loans and advances included the following:<br><br>• Assessing the design and operating effectiveness of manual and automated controls over classification and provisioning of advances including:<br>- The accuracy of data input into the system used for disbursement and recovery of credit facilities;<br>- Controls over correct classification of non-performing advances on time based criteria;<br>- Controls over accurate computation and recording of provisions; and<br>- Controls over the governance and approval process related to provision.

S. No.	Key Audit Matters	How the matter was addressed in our audit
		• Testing, on a sample basis, credit exposures identified by the management as displaying indicators of impairment, assessed the number of days overdue and assessed appropriateness of amount reported for provision in accordance with the PRs;<br>• Testing, on a sample basis, credit exposure where the management has not identified as displaying indicators of impairment challenged the management's assessment by reviewing the historical performances, account movement, financial ratios and reports on security maintained and formed our own view whether any impairment indicators are present;<br>• For consumer advances, analyzed the days past due report for calculation of provision required in accordance with PRs; and<br>• Checking, on a sample basis, accuracy of specific provision against non-performing advances and of general provision against consumer and SME advances by recomputing the provision made in accordance with the criteria prescribed under the PRs.
2	**Valuation of Investments**	
	Refer to note 10 and the accounting policies in notes 4.2 (c) and 6.2 to the unconsolidated financial statements.<br><br>As at December 31, 2020 the Bank has investments classified as "Available-for-sale", "Held for trading" and "Held to maturity", amounting to Rs. 1,002.85 billion which in aggregate represent 57.06 % of the total assets of the Bank.<br><br>Investments are carried at cost or fair value in the accordance with the Bank's accounting policy relating to their recognition. Provision against investments is based on impairment policy of the Bank which includes both objective and subjective factors.<br><br>We identified the valuation of investments including determination of impairment allowance on investments classified as 'Available-for-sale' and "Held to maturity" as a key audit matter because of their significance in relation to the total assets of the Bank and judgment involved in assessing impairment allowance.	Our procedures in respect of valuation of investments included the following:<br><br>• Assessing the design and tested the operating effectiveness of the relevant controls in place relating to valuation of investments;<br><br>• Checking, on a sample basis, the valuation of investments to supporting documents, externally quoted market prices and break-up values; and<br><br>• Evaluating the management's assessment of available for sale and held to maturity investments for any additional impairment in accordance with the Bank's accounting policies and performed an independent assessment of the assumptions.

Information Other than the Unconsolidated Financial Statements and Auditor's Report Thereon

Management is responsible for the other information. The other information comprises the information included in the Bank's Annual Report but does not include the unconsolidated financial statements and our auditors' report thereon.

Our opinion on the unconsolidated financial statements does not cover the other information and we do not express any form of assurance conclusion thereon.

In connection with our audit of the unconsolidated financial statements, our responsibility is to read the other information and, in doing so, consider whether the other information is materially inconsistent with the unconsolidated financial statements or our knowledge obtained in the audit, or otherwise appears to be materially misstated. If, based on the work we have performed, we conclude that there is a material misstatement of this other information, we are required to report that fact. We have nothing to report in this regard.

Responsibilities of Management and the Board of Directors for the Unconsolidated Financial Statements

Management is responsible for the preparation and fair presentation of the unconsolidated financial statements in accordance with accounting and reporting standards as applicable in Pakistan, the requirements of Banking Companies Ordinance, 1962 and the Companies Act, 2017 (XIX of 2017) and for such internal control as management determines is necessary to enable the preparation of unconsolidated financial statements that are free from material misstatement, whether due to fraud or error.

In preparing the unconsolidated financial statements, management is responsible for assessing the Bank's ability to continue as a going concern, disclosing, as applicable, matters related to going concern and using the going concern basis of accounting unless management either intends to liquidate the Bank or to cease operations, or has no realistic alternative but to do so.

The Board of directors is responsible for overseeing the Bank's financial reporting process.

Auditor's Responsibilities for the Audit of the Unconsolidated Financial Statements

Our objectives are to obtain reasonable assurance about whether the unconsolidated financial statements as a whole are free from material misstatement, whether due to fraud or error, and to issue an auditor's report that includes our opinion. Reasonable assurance is a high level of assurance, but is not a guarantee that an audit conducted in accordance with ISAs as applicable in Pakistan will always detect a material misstatement when it exists. Misstatements can arise from fraud or error and are considered material if, individually or in the aggregate, they could reasonably be expected to influence the economic decisions of users taken on the basis of these unconsolidated financial statements.

As part of an audit in accordance with ISAs as applicable in Pakistan, we exercise professional judgment and maintain professional skepticism throughout the audit. We also:

- Identify and assess the risks of material misstatement of the unconsolidated financial statements, whether due to fraud or error, design and perform audit procedures responsive to those risks, and obtain audit evidence that is sufficient and appropriate to provide a basis for our opinion. The risk of not detecting a material misstatement resulting from fraud is higher than for one resulting from error, as fraud may involve collusion, forgery, intentional omissions, misrepresentations, or the override of internal control.

- Obtain an understanding of internal control relevant to the audit in order to design audit procedures that are appropriate in the circumstances, but not for the purpose of expressing an opinion on the effectiveness of the Bank's internal control.

- Evaluate the appropriateness of accounting policies used and the reasonableness of accounting estimates and related disclosures made by management.

- Conclude on the appropriateness of management's use of the going concern basis of accounting and, based on the audit evidence obtained, whether a material uncertainty exists related to events or conditions that may cast significant doubt on the Bank's ability to continue as a going concern. If we conclude that a material uncertainty exists, we are required to draw attention in our auditor's report to the related disclosures in the unconsolidated financial statements or, if such disclosures are inadequate, to modify our opinion. Our conclusions are based on the

audit evidence obtained up to the date of our auditor's report. However, future events or conditions may cause the Bank to cease to continue as a going concern.

- Evaluate the overall presentation, structure and content of the unconsolidated financial statements, including the disclosures, and whether the unconsolidated financial statements represent the underlying transactions and events in a manner that achieves fair presentation.

We communicate with the Board of Directors regarding, among other matters, the planned scope and timing of the audit and significant audit findings, including any significant deficiencies in internal control that we identify during our audit.

We also provide to the Board of Directors with a statement that we have complied with relevant ethical requirements regarding independence, and to communicate with them all relationships and other matters that may reasonably be thought to bear on our independence, and where applicable, related safeguards.

From the matters communicated with the Board of Directors, we determine those matters that were of most significance in the audit of the unconsolidated financial statements of the current year and are therefore the key audit matters. We describe these matters in our auditor's report unless law or regulation precludes public disclosure about the matter or when, in extremely rare circumstances, we determine that a matter should not be communicated in our report because the adverse consequences of doing so would reasonably be expected to outweigh the public interest benefits of such communication.

Report on Other Legal and Regulatory Requirements:

Based on our audit, we further report that in our opinion:

a) proper books of account have been kept by the Bank as required by the Companies Act, 2017 (XIX of 2017) and the returns referred above from the branches have been found adequate for the purpose of our audit;

b) the unconsolidated statement of financial position, the unconsolidated profit and loss account, the unconsolidated statement of comprehensive income, the unconsolidated statement of changes in equity and the unconsolidated statement of cash flows together with the notes thereon have been drawn up in conformity with the Banking Companies Ordinance, 1962 and the Companies Act, 2017(XIX of 2017) and are in agreement with the books of account and returns;

c) investments made, expenditure incurred and guarantees extended during the year were in accordance with the objects and powers of the Bank and the transactions of the Bank which have come to our notice have been within the powers of the Bank; and

d) zakat deductible at source under the Zakat and Ushr Ordinance, 1980 (XVIII of 1980), was deducted by the Bank and deposited in the Central Zakat Fund established under section 7 of that Ordinance.

We confirm that for the purpose of our audit we have covered more than sixty per cent of the total loans and advances of the Bank.

The engagement partner on the audit resulting in this independent auditor's report is M. Rehan Chughtai.

Lahore
Date: February 26, 2021

KPMG Taseer Hadi & Co.
Chartered Accountants

MCB
Bank for Life

Unconsolidated Statement of Financial Position

As at December 31, 2020

	Note	2020	2019
		(Rupees in '000)	
**ASSETS**			
Cash and balances with treasury banks	7	122,180,839	132,704,797
Balances with other banks	8	24,030,328	12,542,239
Lendings to financial institutions	9	17,139,453	1,090,058
Investments	10	1,015,869,448	748,764,502
Advances	11	462,941,787	496,678,874
Fixed assets	12	58,027,904	58,271,245
Intangible assets	13	938,458	957,552
Deferred tax assets		–	–
Other assets	14	56,334,253	64,142,748
		1,757,462,470	1,515,152,015
**LIABILITIES**			
Bills payable	16	23,980,692	11,821,698
Borrowings	17	164,001,533	89,505,892
Deposits and other accounts	18	1,289,502,304	1,144,763,259
Liabilities against assets subject to finance lease		–	–
Subordinated debt		–	–
Deferred tax liabilities	19	6,975,158	5,850,645
Other liabilities	20	82,900,828	94,295,738
		1,567,360,515	1,346,237,232
**NET ASSETS**		190,101,955	168,914,783
**REPRESENTED BY**			
Share capital	21	11,850,600	11,850,600
Reserves	22	80,696,335	77,591,253
Surplus on revaluation of assets	23	27,720,418	23,695,441
Unappropriated profit		69,834,602	55,777,489
		190,101,955	168,914,783
**CONTINGENCIES AND COMMITMENTS**	24		

The annexed notes 1 to 48 and annexures I to II form an integral part of these unconsolidated financial statements.

Imran Maqbool	Hammad Khalid	S.M. Muneer	Mian Umer Mansha	Shahzad Hussain
President/Chief Executive	Chief Financial Officer	Director	Director	Director

Unconsolidated Profit and Loss Account

For the year ended December 31, 2020

	Note	2020	2019
		(Rupees in '000)	
Mark-up / return / interest earned	26	136,075,705	138,291,896
Mark-up / return / interest expensed	27	64,741,214	78,675,682
Net mark-up / interest income		71,334,491	59,616,214
**NON MARK-UP / INTEREST INCOME**			
Fee and commission income	28	10,936,325	11,288,050
Dividend income		1,209,753	1,377,173
Foreign exchange income		2,525,340	2,894,735
Income from derivatives		4,087	14,616
Gain on securities	29	3,332,032	832,846
Other income	30	128,250	271,533
Total non-markup / interest Income		18,135,787	16,678,953
Total Income		89,470,278	76,295,167
**NON MARK-UP / INTEREST EXPENSES**			
Operating expenses	31	32,645,782	32,670,918
Workers welfare fund		964,978	802,046
Other charges	32	297,397	236,357
Total non-markup / interest expenses		33,908,157	33,709,321
Profit before provisions		55,562,121	42,585,846
Provisions / (reversals) and write offs - net	33	7,313,166	2,483,534
Extra ordinary / unusual items		–	–
**PROFIT BEFORE TAXATION**		48,248,955	40,102,312
Taxation	34	19,211,654	16,125,473
**PROFIT AFTER TAXATION**		29,037,301	23,976,839
		Rupees	
**Basic and diluted earnings per share**	35	24.50	20.23

The annexed notes 1 to 48 and annexures I to II form an integral part of these unconsolidated financial statements.

Imran Maqbool	Hammad Khalid	S.M. Muneer	Mian Umer Mansha	Shahzad Hussain
President/Chief Executive	Chief Financial Officer	Director	Director	Director

Unconsolidated Statement of Comprehensive Income

For the year ended December 31, 2020

	2020	2019
	(Rupees in '000)	
**Profit after taxation for the year**	29,037,301	23,976,839
**Other comprehensive income**		
**Items that may be reclassified to profit and loss account in subsequent periods:**		
Effect of translation of net investment in foreign branches	201,352	1,045,588
Movement in surplus on revaluation of investments - net of tax	4,021,886	6,975,891
	4,223,238	8,021,479
**Items that will not be reclassified to profit and loss account in subsequent periods:**		
Remeasurement loss on defined benefit obligations - net of tax	(342,311)	(474,421)
Movement in surplus on revaluation of operating fixed assets - net of tax	–	6,908,341
Movement in surplus on revaluation of non-banking assets - net of tax	119,544	165,776
	(222,767)	6,599,696
**Total comprehensive income**	33,037,772	38,598,014

The annexed notes 1 to 48 and annexures I to II form an integral part of these unconsolidated financial statements.

Imran Maqbool	Hammad Khalid	S.M. Muneer	Mian Umer Mansha	Shahzad Hussain
President/Chief Executive	Chief Financial Officer	Director	Director	Director

Unconsolidated Statement of Changes in Equity

For the year ended December 31, 2020

	Share capital	Capital reserve			Statutory reserve	Revenue reserve	Suplus/(deficit) on revaluation of		Unappropriated profit	Total
		Share premium	Non-distributable capital reserve	Exchange translation reserve		General reserve	Investments	Fixed / non - banking assets		
	(Rupees in '000)									
Balance as at December 31, 2018	11,850,600	23,751,114	908,317	1,629,543	29,259,007	18,600,000	(2,758,144)	12,505,248	53,532,044	149,277,729
**Total comprehensive income for the year ended December 31, 2019**										
Profit after taxation for the year ended December 31, 2019	–	–	–	–	–	–	–	–	23,976,839	23,976,839
Other comprehensive income - net of tax	–	–	–	1,045,588	–	–	6,975,891	7,074,117	(474,421)	14,621,175
	–	–	–	1,045,588	–	–	6,975,891	7,074,117	23,502,418	38,598,014
Transfer to statutory reserve	–	–	–	–	2,397,684	–	–	–	(2,397,684)	–
Transfer in respect of incremental depreciation from surplus on revaluation of fixed assets to unappropriated profit - net of tax	–	–	–	–	–	–	–	(53,248)	53,248	–
Surplus realized on disposal of revalued fixed assets - net of tax	–	–	–	–	–	–	–	(10,560)	10,560	–
Surplus realized on disposal of revalued non-banking assets - net of tax	–	–	–	–	–	–	–	(37,863)	37,863	–
**Transactions with owners, recorded directly in equity**										
Final cash dividend at Rs. 4.0 per share - December 31, 2018	–	–	–	–	–	–	–	–	(4,740,240)	(4,740,240)
Interim cash dividend at Rs. 4.0 per share - March 31, 2019	–	–	–	–	–	–	–	–	(4,740,240)	(4,740,240)
Interim cash dividend at Rs. 4.0 per share - June 30, 2019	–	–	–	–	–	–	–	–	(4,740,240)	(4,740,240)
Interim cash dividend at Rs. 4.0 per share - September 30, 2019	–	–	–	–	–	–	–	–	(4,740,240)	(4,740,240)
	–	–	–	–	–	–	–	–	(18,960,960)	(18,960,960)
Balance as at December 31, 2019	11,850,600	23,751,114	908,317	2,675,131	31,656,691	18,600,000	4,217,747	19,477,694	55,777,489	168,914,783
**Total comprehensive income for the year ended December 31, 2020**										
Profit after taxation for the year ended December 31, 2020	–	–	–	–	–	–	–	–	29,037,301	29,037,301
Other comprehensive income - net of tax	–	–	–	201,352	–	–	4,021,886	119,544	(342,311)	4,000,471
	–	–	–	201,352	–	–	4,021,886	119,544	28,694,990	33,037,772
Transfer to statutory reserve	–	–	–	–	2,903,730	–	–	–	(2,903,730)	–
Transfer in respect of incremental depreciation from surplus on revaluation of fixed assets to unappropriated profit - net of tax	–	–	–	–	–	–	–	(89,135)	89,135	–
Surplus realized on disposal of revalued fixed assets - net of tax	–	–	–	–	–	–	–	(22,544)	22,544	–
Surplus realized on disposal of revalued non-banking assets - net of tax	–	–	–	–	–	–	–	(4,774)	4,774	–
**Transactions with owners, recorded directly in equity**										
Final cash dividend at Rs. 5.0 per share - December 31, 2019	–	–	–	–	–	–	–	–	(5,925,300)	(5,925,300)
Interim cash dividend at Rs. 5.0 per share - March 31, 2020	–	–	–	–	–	–	–	–	(5,925,300)	(5,925,300)
	–	–	–	–	–	–	–	–	(11,850,600)	(11,850,600)
**Balance as at December 31, 2020**	11,850,600	23,751,114	908,317	2,876,483	34,560,421	18,600,000	8,239,633	19,480,785	69,834,602	190,101,955

For details of dividend declaration and appropriations, please refer note 46 to these unconsolidated financial statements.
For details of reserves, please refer note 22 to these unconsolidated financial statements.
The annexed notes 1 to 48 and annexures I to II form an integral part of these unconsolidated financial statements.

Imran Maqbool	Hammad Khalid	S.M. Muneer	Mian Umer Mansha	Shahzad Hussain
President/Chief Executive	Chief Financial Officer	Director	Director	Director

Unconsolidated Cash Flow Statement

For the year ended December 31, 2020

	Note	2020	2019
		(Rupees in '000)	
**CASH FLOW FROM OPERATING ACTIVITIES**			
Profit before taxation		48,248,955	40,102,312
Less: Dividend income		(1,209,753)	(1,377,173)
		47,039,202	38,725,139
Adjustments:			
Depreciation on fixed assets	12.2	2,005,061	1,916,646
Depreciation on right of use assets	31	1,216,784	1,161,795
Depreciation on non-banking assets acquired in satisfaction of claims	31	30,049	45,456
Amortization	13	318,024	301,022
Provisions / (reversals) and write offs - net	33	7,313,166	2,483,534
Workers welfare fund		964,978	802,046
Gain on sale of non-banking assets acquired in satisfaction of claims	30	(3,976)	(111,948)
Charge / (reversal) for defined benefit plan	31.1	74,827	(101,509)
Gain on sale of fixed assets	30	(72,601)	(90,158)
Loss on termination of lease liability against right of use assets	30	15,637	–
Unrealized loss on revaluation of investments classified as held for trading	29	224	4,176
Interest expensed on lease liability against right-of-use assets	27	1,106,824	1,132,099
Loss on amalgamation of a subsidiary		–	7,512
Gain on sale of shares in a subsidiary	29.1	(72,194)	–
		12,896,803	7,550,671
		59,936,005	46,275,810
Decrease / (increase) in operating assets			
Lendings to financial institutions		(16,049,395)	34,016,183
Held-for-trading securities		8,179,715	(128,778)
Advances		26,262,334	7,224,925
Others assets (excluding advance taxation)		9,710,281	(16,297,896)
		28,102,935	24,814,434
(Decrease) / increase in operating liabilities			
Bills Payable		12,158,994	(3,877,582)
Borrowings from financial institutions		75,447,775	(125,990,277)
Deposits		144,739,045	95,725,644
Other liabilities (excluding current taxation)		(18,195,415)	13,859,980
		214,150,399	(20,282,235)
Defined benefits paid		(302,940)	(309,074)
Income tax paid		(16,078,289)	(2,306,594)
Net cash flow from operating activities		285,808,110	48,192,341
**CASH FLOW FROM INVESTING ACTIVITIES**			
Net investments in available-for-sale securities		(286,438,369)	8,627,647
Net investments in held-to-maturity securities		17,363,419	309,822
Investment in a subsidiary		–	(350,000)
Dividends received		1,179,851	1,394,813
Investments in fixed assets		(2,791,537)	(4,395,890)
Investments in Intangible assets		(298,880)	(625,800)
Proceeds from sale of fixed assets		186,909	158,186
Proceeds from sale of non-banking assets acquired in satisfaction of claims		39,000	540,000
Investments in non-banking assets acquired in satisfaction of claims		–	(64,445)
Net cash inflow from amalgamation of a subsidiary		–	40,968
Proceeds from divestment in a subsidiary		99,694	–
Effect of translation of net investment in foreign branches		201,352	1,045,588
Net cash flow (used in) / generated from investing activities		(270,458,561)	6,680,889
**CASH FLOW FROM FINANCING ACTIVITIES**			
Payments of Subordinated debt		–	(3,891,019)
Dividend paid		(11,750,637)	(18,838,387)
Payment of lease liability against right-of-use-assets		(1,682,647)	(1,427,643)
Net cash flow used in financing activities		(13,433,284)	(24,157,049)
Effects of exchange rate changes on cash and cash equivalents		1,595,436	3,553,077
**Increase in cash and cash equivalents**		3,511,701	34,269,258
Cash and cash equivalents at beginning of the year		142,302,153	109,628,331
**Cash and cash equivalents at end of the year**	36	145,813,854	143,897,589

The annexed notes 1 to 48 and annexures I to II form an integral part of these unconsolidated financial statements.

Imran Maqbool	Hammad Khalid	S.M. Muneer	Mian Umer Mansha	Shahzad Hussain
President/Chief Executive	Chief Financial Officer	Director	Director	Director

Notes To The Unconsolidated Financial Statements

For the year ended December 31, 2020

1. STATUS AND NATURE OF BUSINESS

MCB Bank Limited (the 'Bank') is a banking company incorporated in Pakistan and is engaged in commercial banking and related services. The Bank's ordinary shares are listed on the Pakistan stock exchange. The Bank's Registered Office and Principal Office are situated at MCB -15 Main Gulberg, Lahore. The Bank operates 1,418 branches (2019: 1,399 branches) within Pakistan and 11 branches (2019: 11 branches) outside Pakistan (including the Karachi Export Processing Zone branch).

2. BASIS OF PRESENTATION

2.1 These unconsolidated financial statements represent separate financial statements of MCB Bank Limited. The consolidated financial statements of the Group are being issued separately.

2.2 In accordance with the directives of the Federal Government regarding the shifting of the banking system to Islamic modes, the State Bank of Pakistan has issued various circulars from time to time. Permissible forms of trade-related modes of financing include purchase of goods by banks from their customers and immediate resale to them at appropriate profit in price on deferred payment basis. The purchases and sales arising under these arrangements are not reflected in these unconsolidated financial statements as such but are restricted to the amount of facility actually utilized and the appropriate portion of profit thereon.

2.3 The unconsolidated financial statements are presented in Pak Rupees, which is the Bank's functional and presentation currency. The amounts are rounded off to the nearest thousand.

3. STATEMENT OF COMPLIANCE

3.1 These unconsolidated financial statements have been prepared in accordance with accounting and reporting standards as applicable in Pakistan. The accounting and reporting standards comprise of:

- International Financial Reporting Standards (IFRS) issued by the International Accounting Standards Board (IASB) as are notified under the Companies Act, 2017;
- Islamic Financial Accounting Standards (IFAS) issued by the Institute of Chartered Accountants of Pakistan as are notified under the Companies Act, 2017;
- Provisions of and directives issued under the Banking Companies Ordinance, 1962, the Companies Act, 2017; and
- Directives issued by the SBP and the Securities and Exchange Commission of Pakistan (SECP).

Wherever the requirements of the Banking Companies Ordinance, 1962, Companies Act, 2017 or the directives issued by the SBP and the SECP differ with the requirements of IFRS or IFAS, requirements of the Banking Companies Ordinance, 1962, the Companies Act, 2017 and the said directives shall prevail.

The State Bank of Pakistan has deferred the applicability of International Financial Reporting Standard 9, 'Financial Instruments' through BPRD Circular No. 04 of 2019 dated October 23, 2019 and International Accounting Standards 40, 'Investment Property' for Banking Companies through BSD Circular No. 10 dated August 26, 2002. The Securities and Exchange Commission of Pakistan (SECP) has deferred applicability of IFRS-7 "Financial Instruments: Disclosures" on banks through S.R.O 411(1) /2008 dated April 28, 2008. Accordingly, the requirements of these standards have not been considered in the preparation of these unconsolidated financial statements. However, investments have been classified and valued in accordance with the requirements prescribed by the State Bank of Pakistan through various circulars.

IFRS10 Consolidated Financial Statements was made applicable from period beginning on or after January 01, 2015 vide S.R.O 633(I)/2014 dated July 10, 2014 by SECP. However, SECP has directed through S.R.O 56(I) /2016 dated January 28, 2016, that the requirements of consolidation under section 237 of the repealed Companies Ordinance 1984 (Section 228 of Companies Act 2017) and

IFRS-10 “Consolidated Financial Statements” is not applicable in case of investment by companies in mutual funds established under Trust structure. Accordingly, the requirements of these standards have not been considered in the preparation of these unconsolidated financial statements.

3.2 Standards, interpretations and amendments to published approved accounting standards that are effective in the current year

There are certain other new standards and interpretations of and amendments to existing accounting standards that have become applicable to the Bank for accounting periods beginning on or after January 1, 2020. These are considered either to not be relevant or not to have any significant impact on the Bank’s unconsolidated financial statements.

3.3 Standards, interpretations and amendments to published approved accounting standards that are not yet effective

The following other standards, amendments and interpretations of approved accounting standards are effective for accounting periods beginning on or after January 1, 2021:

	Effective date (annual periods beginning on or after)
IAS 1, Presentation of Financial Statements (Amendments)	January 1, 2022
IAS 16, Property, plant and equipment (Amendments)	January 1, 2022
IAS 37, Provisions, Contingent Liabilities and Contingent Assets (Amendments)	January 1, 2022

The SECP, through SRO 229(I)/2019 dated February 14, 2019, has notified that IFRS 9, Financial Instruments, is applicable for accounting periods ending on or after June 30, 2019. However, as per BPRD Circular No. 04 of 2019 dated October 23, 2019 of SBP, effective date of IFRS 9 implementation is January 01, 2021.The requirements of this standard are incorporated in these unconsolidated financial statements for the jurisdictions where IFRS 9 has been adopted.

IFRS 9, Financial Instruments: Classification and Measurement, addresses recognition, classification, measurement and derecognition of financial assets and financial liabilities. The standard has also introduced a new impairment model for financial assets which requires recognition of impairment charge based on an ‘expected credit losses’ (ECL) approach rather than the ‘incurred credit losses’ approach as currently followed. The ECL approach has an impact on all assets of the Bank which are exposed to credit risk. The impact of the application of IFRS 9 in Pakistan on the Bank's financial statements is being assessed and SBP's implementation guidelines are awaited.

There are other new and amended standards and interpretations that are mandatory for the Bank’s accounting periods beginning on or after January 1, 2021 but are considered not to be relevant or do not have any significant effect on the Bank’s operations and are therefore not detailed in these unconsolidated financial statements.

4. BASIS OF MEASUREMENT

4.1 These unconsolidated financial statements have been prepared under the historical cost convention except that certain classes of fixed assets and non-banking assets acquired in satisfaction of claims are stated at revalued amounts and certain investments and derivative financial instruments have been marked to market and are carried at fair value. In addition, obligations in respect of staff retirement benefits are carried at present value.

4.2 Critical accounting estimates and judgments

The preparation of unconsolidated financial statements in conformity with the approved accounting standards requires the use of certain critical accounting estimates. It also requires the management

Notes To The Unconsolidated Financial Statements

For the year ended December 31, 2020

to exercise its judgment in the process of applying the Bank's accounting policies. Estimates and judgments are continually evaluated and are based on historical experiences, including expectations of future events that are believed to be reasonable under the circumstances. Revisions to accounting estimates are recognized in the period in which the estimates are revised if the revision affects only that period, or in the period of revision and future periods if the revision affects both current and future periods. The areas where various assumptions and estimates are significant to the Bank's financial statements or where judgment was exercised in the application of accounting policies are as follows:

a) Classification of investments

In classifying investments, the Bank follows the guidance provided in SBP circulars:

- Investments classified as 'held for trading', are securities which are acquired with an intention to trade by taking advantage of short term market / interest rate movements and are to be sold within 90 days of acquisition.
- Investments classified as 'held to maturity' are non-derivative financial assets with fixed or determinable payments and fixed maturity. In making this judgment, the Bank evaluates its intention and ability to hold such investment to maturity.
- The investments other than those in subsidiaries and associates which are not classified as 'held for trading' or 'held to maturity' are classified as 'available for sale'.

b) Provision against advances

The Bank reviews its loan portfolio to assess the amount of non-performing advances and provision required there against on regular basis. While assessing this requirement, various factors including the delinquency in the account, financial position of the borrowers and the requirements of the Prudential Regulations are considered.

The amount of general provision is determined in accordance with the relevant regulations and management's judgment as explained in note 11.4.4.

c) Impairment of 'available for sale' equity investments

The Bank determines that 'available for sale' equity investments are impaired when there has been a significant or prolonged decline in the fair value below its cost. The determination of what is significant or prolonged requires judgment. In making this judgment, the Bank evaluates among other factors, the normal volatility in share price. In addition, the impairment may be appropriate when there is an evidence of deterioration in the financial health of the investee and sector performance, changes in technology and operational/financial cash flows.

d) Taxation

In making the estimates for income taxes currently payable by the Bank, the management considers the current income tax laws and the decisions of appellate authorities on certain issues in the past.

e) Fair value of derivatives

The fair values of derivatives which are not quoted in active markets are determined by using valuation techniques. The valuation techniques take into account the relevant underlying parameters including foreign currency involved, interest rates, yield curves, volatilities, contracts duration etc.

f) Depreciation, amortization and revaluation of operating fixed assets

The management reviews the useful lives and residual values of assets annually by considering expected pattern of economic benefit that the management expects to drive from the item and the maximum period up to which such benefits are expected to be available. Any change in estimates in future years might affect the carrying amounts of the respective items of assets with a corresponding effect on the depreciation charge and impairment.Such change is accounted for as change in accounting estimates in accordance with International Accounting Standard (IAS) 8 "Accounting

Policies, Changes in Accounting Estimates and Errors". Further, the Bank estimates the revalued amount of land and buildings on a regular basis. The estimates are based on valuations carried out by independent professional valuers under the market conditions.

g) Staff retirement benefits

Certain actuarial assumptions have been adopted as disclosed in note 38 of these unconsolidated financial statements for the actuarial valuation of staff retirement benefit plans. Actuarial assumptions are entity's best estimates of the variables that will determine the ultimate cost of providing post employment benefits. Changes in these assumptions in future years may affect the liability / asset under these plans in those years.

h) Lease term

The Bank applies judgement to determine the lease term for some lease contracts in which it is a lessee that include renewal options. The assessment of whether the Bank is reasonably certain to exercise such options impacts the lease term, which significantly affects the amount of lease liabilities and right-of-use assets recognized.

5. FINANCIAL RISK MANAGEMENT

These risk management policies continue to remain robust and the Bank is reviewing its portfolio regularly and conducts rapid portfolio reviews in line with emerging risks.

Impact of COVID-19 on the Financial Statements

The COVID - 19 pandemic has taken a toll on all economies and emerged as a contagion risk around the globe, including Pakistan. To reduce the impact on businesses and economies in general, regulators / governments across the globe have introduced a host of measures on both the fiscal and economic fronts.

The State Bank of Pakistan (SBP) has also responded to the crisis by cutting the Policy Rate by 625 basis points during the period to 7% by June 2020. Other regulatory measures to provide an impetus to economic activity include the following:

– Reduction in the capital conservation buffer by 100 basis points to 1.5%;
– Increasing the regulatory limit on extension of credit to SMEs to Rs 180 million;
– Relaxing the debt burden ratio for consumer loans from 50% to 60%;
– Allowing banks to defer borrowers' principal loan payments by one year and / or restructure / reschedule loans for borrowers who require relief of principal repayment exceeding one year and / or mark-up with no reflection on credit history; and
– Introduction of refinancing schemes for payment of wages and salaries.

COVID 19 has impacted the banks in Pakistan from various facets which include muted credit risk increase, reduced fee income due to slowdown in economic activity, branch closures and cyber security threat management.

The potential impact of the economic stress posed by the COVID-19 outbreak is difficult to predict, as many of Bank's borrowers have availed the SBP enabled deferment/restructuring & rescheduling relief. Hence, the management has exercised prudence and booked General Provision of Rs. 4.0 billion during the year ended December 31, 2020.

Detailed disclosure on financial risk management has been reported in Note 45 to the financial statements.

6. SUMMARY OF SIGNIFICANT ACCOUNTING POLICIES

The accounting policies adopted in the preparation of these unconsolidated financial statements are consistent with those of the previous financial year:

Notes To The Unconsolidated Financial Statements

For the year ended December 31, 2020

6.1 IFRS 16 - Lease Liability & Right of Use Assets

The lease liability is initially measured at the present value of lease payments to be made over the term of the lease, discounted using the Banks's incremental borrowing rate. The lease liability is subsequently measured at amortized cost using the effective interest rate method. The carrying amount is remeasured/adjusted if there are changes in the future cash flows or the lease term.

The right-of-use asset is initially measured at cost, which comprises the initial amount of the lease liability, adjusted for, as applicable, any lease payments made at or before the commencement date. On subsequent measurement, right-of-use assets are stated at cost less any accumulated depreciation / accumulated impairment losses and are adjusted for any remeasurement of the lease liability.

Right-of-use assets are depreciated on a straight line basis over the lease term as this method closely reflects the expected pattern of consumption of future economic benefits. Carrying amount of the lease liability is derecognised upon termination of the lease contract with corresponding adjustment to right-of-use asset. Gain or loss on termination of lease contract is recognised in the profit and loss account.

The Bank has elected not to recognize a right-of-use asset and the corresponding lease liability for short-term leases with terms of 12 months or less and leases of low-value assets. Payments associated with these leases are recognized as an expense in the profit or loss account on a straight-line basis.

6.2 Investments

The Bank classifies its investments as follows:

a) Held for trading

These are securities, which are either acquired for generating profit from short-term fluctuations in market prices, interest rate movements, dealers margin or are securities included in a portfolio in which a pattern of short-term profit taking exists.

b) Held to maturity

These are securities with fixed or determinable payments and fixed maturity in respect of which the Bank has the positive intent and ability to hold to maturity.

c) Available for sale

These are investments, other than those in subsidiaries and associates, that do not fall under the 'held for trading' or 'held to maturity' categories.

Investments are initially recognized at cost which in case of investments other than 'held for trading' include transaction costs associated with the investment. Transaction costs on investments held for trading are expensed in the profit and loss account.

All purchases and sales of investments that require delivery within the time frame established by regulation or market convention are recognized at the trade date. Trade date is the date on which the Bank commits to purchase or sell the investment.

In accordance with the requirements of the State Bank of Pakistan, quoted securities, other than those classified as 'held to maturity', investments in subsidiaries and investments in associates are subsequently re-measured to market value. Surplus / deficit arising on revaluation of quoted securities which are classified as 'available for sale', is taken to surplus / deficit on revaluation of investments through statement of comprehensive income in equity till disposal at which time it is recorded in profit and loss account. Surplus / deficit arising on revaluation of quoted securities which are classified as 'held for trading', is taken to the profit and loss account, currently.

Unquoted equity securities (excluding investments in subsidiaries and associates) are valued at the lower of cost and break-up value. Break-up value of equity securities is calculated with reference to the net assets of the investee company as per the latest available audited financial statements. Investments classified as 'held to maturity' are carried at amortized cost less accumulated impairment losses, if any.

Associates are all entities over which the Bank has significant influence but not control. Subsidiaries are all entities over which the Bank has the power to govern the financial and operating policies accompanying a shareholding of more than one half of the voting rights. Investments in subsidiaries and investments in associates are carried at cost less accumulated impairment losses, if any.

Provision for impairment in the values of securities (except debentures, participation term certificates and term finance certificates) is made currently. Impairment of 'available for sale' equity investments is discussed in 4.2(c). Provisions for impairment in value of debentures, participation term certificates and term finance certificates are made as per the requirements of the Prudential Regulations issued by the State Bank of Pakistan.

Impairment against investment in subsidiaries and associates is assessed as per the requirements of IAS 36.

6.3 Sale and repurchase agreements

Securities sold subject to a repurchase agreement (repo) are retained in these unconsolidated financial statements as investments and the counter party liability is included in borrowings. Securities purchased under an agreement to resell (reverse repo) are not recognized in these unconsolidated financial statements as investments and the amount extended to the counter party is included in lendings to financial institutions. The difference between the purchase / sale and re-sale / re-purchase price is recognized as mark-up income / expense on a time proportion basis, as the case may be.

6.4 Advances

Advances are stated net of specific and general provisions. Specific provision is determined on the basis of the Prudential Regulations and other directives issued by the State Bank of Pakistan (SBP) and charged to the profit and loss account. Provisions are held against identified as well as unidentified losses. Provisions against unidentified losses include general provision against Consumer and Small Enterprise (SEs) loans made in accordance with the requirements of the Prudential Regulations issued by SBP and provision based on historical loss experience on advances. General provisions pertaining to overseas advances are made in accordance with the requirements of the regulatory authorities of the respective countries. Advances are written off when there is no realistic prospect of recovery.

Leases where the Bank transfers substantially all the risks and rewards incidental to ownership of an asset to the lessee are classified as finance leases. A receivable is recognized at an amount equal to the present value of the lease payments including any guaranteed residual value. Finance lease receivables are included in advances to the customers.

6.5 Fixed assets and depreciation

Fixed assets other than land and buildings are stated at cost less accumulated depreciation and accumulated impairment losses, if any. Buildings are carried at revalued amount less any accumulated depreciation and subsequent impairment losses, if any. Land is carried at revalued amount less any subsequent impairment losses, if any. Cost of property and equipment of foreign operations includes exchange differences arising on currency translation at year-end rates.

Capital work-in-progress is stated at cost less accumulated impairment losses, if any. These are transferred to specific assets as and when assets become available for use.

Depreciation on all fixed assets (excluding land) is charged using the straight line method in accordance with the rates specified in note 12.2 to these unconsolidated financial statements and after taking into account residual value, if any. The residual values, useful lives and depreciation methods are reviewed and adjusted, if appropriate, at each balance sheet date.

Notes To The Unconsolidated Financial Statements

For the year ended December 31, 2020

Depreciation on additions is charged from the month the assets are available for use while no depreciation is charged in the month in which the assets are disposed off.

Land and buildings are revalued by independent, professionally qualified valuers with sufficient regularity to ensure that their net carrying amount does not differ materially from their fair value. An increase arising on revaluation is credited to the surplus on revaluation of fixed assets account. A decrease arising on revaluation of fixed assets is adjusted against the surplus of that asset or, if no surplus exists, is charged to the profit and loss account as an impairment of the asset. A surplus arising subsequently on an impaired asset is reversed through the profit and loss account up to the extent of the original impairment.

Surplus on revaluation of fixed assets (net of associated deferred tax) to the extent of the incremental depreciation charged on the related assets is transferred to unappropriated profit.

Gains / losses on sale of property and equipment are credited / charged to the profit and loss account currently, except that the related surplus on revaluation of land and buildings (net of deferred taxation) is transferred directly to unappropriated profit.

Subsequent costs are included in the asset's carrying amount or are recognized as a separate asset, as appropriate, only when it is probable that future economic benefits associated with the item will flow to the Bank and the cost of the item can be measured reliably. All other repairs and maintenance are charged to the profit and loss account.

6.5.1 Intangible assets

Intangible assets are stated at cost less accumulated amortization and accumulated impairment losses, if any. Intangible assets are amortized from the month when these assets are available for use, using the straight line method, whereby the cost of the intangible assets are amortized over its estimated useful lives over which economic benefits are expected to flow to the Bank. The useful lives are reviewed and adjusted, if appropriate, at each balance sheet date.

6.6 Impairment

The carrying amount of assets are reviewed at each balance sheet date for impairment whenever events or changes in circumstances indicate that the carrying amounts of the assets may not be recoverable. If such indication exists and where the carrying value exceeds the estimated recoverable amount, assets are written down to their recoverable amounts. The resulting impairment loss is taken to the profit and loss account except for impairment loss on revalued assets, which is adjusted against the related revaluation surplus to the extent that the impairment loss does not exceed the surplus on revaluation of that asset.

6.7 Staff retirement benefits

The Bank operates the following staff retirement benefits for its employees:

a) For clerical / non-clerical staff who did not opt for the new scheme, the Bank operates the following:

- an approved contributory provident fund;
- an approved gratuity scheme; and
- a contributory benevolent scheme

b) For clerical / non-clerical staff who joined the Bank after the introduction of the new scheme and for others who opted for the new scheme introduced in 1975, the Bank operates the following:

- an approved non-contributory provident fund introduced in lieu of the contributory provident fund;
- an approved pension fund; and
- contributory benevolent scheme

c) For officers who joined the Bank after the introduction of the new scheme and for others who opted for the new scheme introduced in 1977, the Bank operates the following:

- an approved non-contributory provident fund introduced in lieu of the contributory provident fund;
- an approved pension fund, and
- contributory benevolent fund.

However, the management has replaced the pension benefits for employees in the officer category with a contributory provident fund for services rendered after December 31, 2003.

d) For executives and officers who joined the Bank on or after January 01, 2000, the Bank operates an approved contributory provident fund.

e) Post retirement medical benefits to entitled employees.

Annual contributions towards the defined benefit plans and schemes are made on the basis of actuarial advice using the Projected Unit Credit Method. The above benefits are payable to staff at the time of separation from the Bank's services subject to the completion of qualifying period of service. Actuarial gains / losses arising from experience adjustments and changes in actuarial assumptions are recognized in other Comprehensive Income in the period of occurrence.

Past service cost is the change in the present value of the defined benefit obligation resulting from a plan amendment or curtailment. The Bank recognizes past service cost as an expense at the earlier of the following dates:

(i) when the plan amendment or curtailment occurs; and

(ii) and when the Bank recognizes related restructuring costs or termination benefits.

Employees' compensated absences

Liability in respect of employees' compensated absences is accounted for in the year in which these are earned on the basis of actuarial valuation carried out using the Projected Unit Credit Method. Actuarial gains / losses arising from experience adjustments and changes in actuarial assumptions are recognized in Profit and Loss account in the period of occurrence.

6.8 Taxation

Current

Provision for current taxation is based on taxable income at the current rates of taxation after taking into consideration available tax credits and rebates. The charge for current tax also includes adjustments where considered necessary, relating to prior years which arise from assessments framed / finalized during the year.

Deferred

Deferred tax is recognized using the balance sheet liability method on all temporary differences between the amounts attributed to assets and liabilities for financial reporting purposes and amounts used for taxation purposes. The Bank records deferred tax assets / liabilities using the tax rates, enacted or substantively enacted by the balance sheet date expected to be applicable at the time of its reversal. Deferred tax asset is recognized only to the extent that it is probable that future taxable profits will be available against which the asset can be utilized. Deferred tax assets are reduced to the extent that it is no longer probable that the related tax benefit will be realized. The Bank also recognizes deferred tax asset / liability on deficit / surplus on revaluation of securities and deferred tax liability on surplus on revaluation of fixed assets which is adjusted against the related deficit / surplus in accordance with the requirements of International Accounting Standard (IAS) 12, 'Income Taxes'.

Notes To The Unconsolidated Financial Statements
For the year ended December 31, 2020

Deferred tax liability is not recognized in respect of taxable temporary differences associated with exchange translation reserves of foreign operations, where the timing of the reversal of the temporary difference can be controlled and it is probable that the temporary differences will not reverse in the foreseeable future.

6.9 Provisions

Provisions are recognized when the Bank has a legal or constructive obligation as a result of past events and it is probable that an outflow of resources will be required to settle the obligation and a reliable estimate of the amount can be made. Provisions are reviewed at each balance sheet date and are adjusted to reflect the current best estimates.

6.10 Foreign currencies

6.10.1 Foreign currency transactions

Transactions in foreign currencies other than the results of foreign operations discussed in note 6.10.2 are translated to Rupees at the foreign exchange rates prevailing on the transaction date. Monetary assets and liabilities in foreign currencies are expressed in Rupee terms at the rates of exchange prevailing at the balance sheet date. Forward foreign exchange contracts are valued at the rates applicable to their respective maturities.

6.10.2 Foreign operations

The assets and liabilities of foreign branches are translated to Rupees at exchange rates prevailing at the statement of financial position date. The results of foreign operations are translated to Rupees at the average rate of exchange for the year.

6.10.3 Translation gains and losses

Translation gains and losses are included in the profit and loss account, except those arising on the translation of the Bank's net investment in foreign branches, which are taken to the capital reserve (exchange translation reserve) until the disposal of the net investment, at which time these are recognized in the profit and loss account.

6.10.4 Commitments

Commitments for outstanding forward foreign exchange contracts are disclosed in these unconsolidated financial statements at committed amounts. Contingent liabilities / commitments for letters of credit and letters of guarantee denominated in foreign currencies are expressed in Rupee terms at the rates of exchange prevailing at the statement of financial position date.

6.11 Acceptances

Acceptances comprise undertakings by the Bank to pay bills of exchange drawn on customers. The Bank expects most acceptances to be simultaneously settled with the reimbursement from the customers.

6.12 Revenue recognition

- Mark-up / interest on advances and returns on investments are recognized on a time proportion basis using the effective interest method except that mark-up / interest on non-performing advances and investments is recognized on a receipt basis, in accordance with the requirements of the Prudential Regulations issued by the State Bank of Pakistan (SBP) or as permitted by the regulations of the overseas regulatory authorities of countries where the branches operate. Where debt securities are purchased at premium or discount, such premium / discount is amortized through the profit and loss account over the remaining period of maturity.

- Financing method is used in accounting for income from lease financing. Under this method, the unearned lease income (excess of the sum of total lease rentals and estimated residual value over the cost of leased assets) is deferred and taken to income over the term of the lease period so as to produce a constant periodic rate of return on the outstanding net investment in lease. Gains / losses on termination of lease contracts are recognized as income when these are realized.

– Commission income is recognized on a time proportion basis.

– Dividend income is recognized when the Bank's right to receive dividend is established.

– Gain / loss on sale of investments is credited / charged to profit and loss account currently.

6.13 Assets acquired in satisfaction of claims

Non-banking assets (NBA) acquired in satisfaction of claims are carried at revalued amounts less accumulated depreciation and impairment loss. These assets are revalued by professionally qualified valuers with sufficient regularity to ensure that their net carrying value does not differ materially from their fair value. A surplus arising on revaluation of property is credited to the 'surplus on revaluation of non banking assets' account through statement of comprehensive income in equity and any deficit arising on revaluation is taken to profit and loss account directly. Legal fees, transfer costs and direct costs of acquiring title to property are charged to profit and loss account and not capitalized.

6.14 Cash and cash equivalents

Cash and cash equivalents include cash and balances with treasury banks and balances with other banks (net of overdrawn Nostro balances) in current and deposit accounts.

6.15 Financial instruments

6.15.1 Financial assets and financial liabilities

Financial instruments carried on the statement of financial position include cash and balances with treasury banks, balances with other banks, lendings to financial institutions, investments, advances, other assets, bills payable, borrowings, deposits and other liabilities. The particular recognition methods adopted for significant financial assets and financial liabilities are disclosed in the individual policy statements associated with these assets and liabilities.

6.15.2 Derivative financial instruments

Derivative financial instruments are initially recognized at fair value on the date on which a derivative contract is entered into and are subsequently remeasured at their fair value using valuation techniques. All the derivative financial instruments are carried as an asset when the fair value is positive and as a liability when the fair value is negative. Any change in the fair value of derivative financial instruments is taken to the profit and loss account currently.

6.15.3 Off setting

Financial assets and financial liabilities are off set and the net amount is reported in these unconsolidated financial statements when there is a legally enforceable right to set off and the Bank intends either to settle on a net basis, or to realize the assets and settle the liabilities, simultaneously.

6.16 Borrowings / deposits

Borrowings / deposits are recorded at the proceeds received. The cost of borrowings / deposits is recognized as an expense in the period in which this is incurred.

6.17 Segment reporting

A segment is a distinguishable component of the Bank that is engaged in providing products or services (business segment) or in providing products or services within a particular economic environment (geographical segment), which is subject to risks and rewards that are different from those of other segments. The Bank's primary format of reporting is based on business segments.

6.17.1 Business segments

Retail banking

This includes retail lending and deposits, banking services, cards and branchless banking.

Notes To The Unconsolidated Financial Statements

For the year ended December 31, 2020

Corporate banking

This comprises of loans, deposits, project financing, trade financing, investment banking and other banking activities / with Bank's corporate and public sector customers.

Consumer Banking

This segment primarily constitutes consumer financing activities with individual customers of the Bank. Product suite offered to these customers include credit cards, auto loans, housing finance and personal loans.

Treasury

This includes fixed income, equity, foreign exchange, credit, funding, own position securities, lendings and borrowings and derivatives for hedging and market making.

International Banking

This comprises of loans, deposits, project financing, trade financing, investment banking and other banking activities by Bank's overseas operations.

Others

This includes the head office related activities and other functions which cannot be classified in any of the above segments.

6.17.2 Geographical segments

The Bank operates in three geographic regions being:

- Pakistan
- South Asia
- Middle East

6.18 Dividend distribution and appropriation

Dividends (including bonus dividend) and other appropriations (except appropriations which are required by law) are recognized in the period in which these are approved.

6.19 Business combination

Business combinations other than under common control transaction are accounted for by applying the acquisition method. The cost of acquisition is measured as the fair value of assets given, equity instruments issued and the liabilities incurred or assumed at the date of acquisition. The consideration transferred includes the fair value of any asset or liability resulting from a contingent consideration arrangement, if any. Acquisition-related costs are expensed as incurred. Identifiable assets acquired and liabilities and contingent liabilities assumed in a business combination are measured initially at their fair values at the acquisition date. The excess of the consideration transferred over the fair value of the identifiable net assets acquired is recorded as goodwill. If this is less than the fair value of the net assets acquired in the case of a bargain purchase, the difference is recognized directly in the profit and loss account or as directed by the SBP.

6.20 Earnings per share

The Bank presents basic and diluted earnings per share (EPS). Basic EPS is calculated by dividing the profit or loss attributable to ordinary shareholders of the Bank by the weighted average number of ordinary shares outstanding during the year.

Notes To The Unconsolidated Financial Statements
For the year ended December 31, 2020

	Note	2020	2019
		(Rupees in '000)	
**7. CASH AND BALANCES WITH TREASURY BANKS**			
In hand			
Local currency		22,094,317	18,738,087
Foreign currencies		6,183,785	2,201,941
		28,278,102	20,940,028
With State Bank of Pakistan in			
Local currency current account	7.1	47,257,342	56,533,231
Foreign currency current account	7.2	1,966,635	277,126
Foreign currency deposit account	7.3	10,215,984	14,023,401
		59,439,961	70,833,758
With other central banks in			
Foreign currency current account	7.4	11,851,311	16,220,148
With National Bank of Pakistan in			
Local currency current account		21,673,576	24,390,028
Prize bonds		937,889	320,835
		122,180,839	132,704,797

7.1 This represents current accounts maintained with the SBP under the Cash Reserve Requirement of section 22 of the Banking Companies Ordinance, 1962.

7.2 This represents US Dollar settlement account maintained with SBP.

7.3 This represents account maintained with the SBP to comply with the Special Cash Reserve requirement. This includes balance of Rs. 6,810.656 million (2019: Rs. 10,517.551 million) which carries interest rate of 0% (2019: 0.70%) per annum as declared by SBP.

7.4 Foreign currency current account with other central banks are maintained to meet their minimum cash reserves and capital requirements pertaining to the foreign branches of the Bank.

	Note	2020	2019
		(Rupees in '000)	
**8. BALANCES WITH OTHER BANKS**			
Outside Pakistan			
In current account		21,798,363	8,396,527
In deposit account	8.1	2,231,965	4,145,712
		24,030,328	12,542,239
		24,030,328	12,542,239

8.1 Balances with other banks outside Pakistan in deposit accounts carry interest rate of 1.5% (2019: 2.60% to 8.30%) per annum.

	Note	2020	2019
		(Rupees in '000)	
**9. LENDINGS TO FINANCIAL INSTITUTIONS**			
Call / clean money lendings	9.1	11,002,195	1,090,058
Repurchase agreement lendings (Reverse Repo)	9.2	6,137,258	–
		17,139,453	1,090,058

Notes To The Unconsolidated Financial Statements

For the year ended December 31, 2020

9.1 Call money lending carries mark-up rate ranging from 0.1% to 4.55% (2019: 7.0% to 9.0%) per annum and is due to mature in January 2021.

9.2 Repurchase agreement lendings carry mark-up rate ranging from 6.4% to 7.1% per annum and is due to mature in January 2021.

	2020	2019
	(Rupees in '000)	
**9.3 Particulars of lending**		
In local currency	6,137,258	880,853
In foreign currencies	11,002,195	209,205
	17,139,453	1,090,058

	2020			2019		
	Held by Bank	Further given as collateral	Total	Held by Bank	Further given as collateral	Total
	(Rupees in '000)					
**9.4 Securities held as collateral against lendings to financial institutions**						
Market Treasury Bills	6,137,258	–	6,137,258	–	–	–
Total	6,137,258	–	6,137,258	–	–	–

10. INVESTMENTS

10.1 Investments by type:

	Note	2020				2019			
		Cost/ amortised cost	Provision for diminution	Surplus/ (Deficit)	Carrying value	Cost/ amortised cost	Provision for diminution	Surplus (Deficit)	Carrying value
		(Rupees in '000)							
**Held-for-trading securities**									
Federal Government Securities		1,309,116	–	(224)	1,308,892	9,479,849	–	(4,128)	9,475,721
Shares		–	–	–	–	13,158	–	(48)	13,110
		1,309,116	–	(224)	1,308,892	9,493,007	–	(4,176)	9,488,831
**Available-for-sale securities**									
Federal Government Securities		946,641,148	(4,719)	9,537,433	956,173,862	666,128,289	–	4,981,349	671,109,638
Shares and units		26,582,088	(10,116,283)	3,119,160	19,584,965	24,938,090	(10,148,760)	1,497,556	16,286,886
Non Government Debt Securities		1,797,840	–	2,252	1,800,092	1,309,773	–	(3,359)	1,306,414
Foreign Securities		7,463,939	(1,714)	17,509	7,479,734	3,657,020	–	13,295	3,670,315
		982,485,015	(10,122,716)	12,676,354	985,038,653	696,033,172	(10,148,760)	6,488,841	692,373,253
**Held-to-maturity securities**									
Federal Government Securities		4,612,390	(11,542)	–	4,600,848	16,207,249	(2,211)	–	16,205,038
Provincial Government Securities		118	(118)	–	–	118	(118)	–	–
Non Government Debt Securities		9,270,317	(490,341)	–	8,779,976	9,867,925	(533,788)	–	9,334,137
Foreign Securities		3,149,647	(27,281)	–	3,122,366	8,320,599	(3,569)	–	8,317,030
		17,032,472	(529,282)	–	16,503,190	34,395,891	(539,686)	–	33,856,205
**Associates**	10.12	700,401	–	–	700,401	700,401	–	–	700,401
**Subsidiaries**	10.12	12,319,037	(725)	–	12,318,312	12,346,537	(725)	–	12,345,812
**Total Investments**		1,013,846,041	(10,652,723)	12,676,130	1,015,869,448	752,969,008	(10,689,171)	6,484,665	748,764,502

Notes To The Unconsolidated Financial Statements
For the year ended December 31, 2020

	Note	2020 Cost/ amortised cost	2020 Provision for diminution	2020 Surplus/ (Deficit)	2020 Carrying value	2019 Cost/ amortised cost	2019 Provision for diminution	2019 Surplus (Deficit)	2019 Carrying value
		(Rupees in '000)							
**10.2 Investments by segments:**									
Federal Government Securities:									
Market Treasury Bills		598,470,191	–	256,237	598,726,428	397,866,023	–	(413,415)	397,452,608
Pakistan Investment Bonds		349,738,865	–	9,180,611	358,919,476	292,723,081	–	5,376,303	298,099,384
Sukuks bonds		1,791,122	(2,872)	35,195	1,823,445	195,929	–	14,333	210,262
Naya Pakistan Certificates		465,779	–	–	465,779	–	–	–	–
Euro Bonds		2,096,697	(13,389)	65,166	2,148,474	1,030,354	(2,211)	–	1,028,143
		952,562,654	(16,261)	9,537,209	962,083,602	691,815,387	(2,211)	4,977,221	696,790,397
Provincial Government Securities		118	(118)	–	–	118	(118)	–	–
Shares:									
Listed Companies		24,999,638	(9,946,958)	3,119,160	18,171,840	23,398,083	(9,982,844)	1,497,508	14,912,747
Unlisted Companies		1,582,450	(169,325)	–	1,413,125	1,553,165	(165,916)	–	1,387,249
		26,582,088	(10,116,283)	3,119,160	19,584,965	24,951,248	(10,148,760)	1,497,508	16,299,996
Non Government Debt Securities									
Listed		5,064,730	(16,269)	7,918	5,056,379	4,946,646	(16,269)	(3,359)	4,927,018
Unlisted		6,003,427	(474,072)	(5,666)	5,523,689	6,231,052	(517,519)	–	5,713,533
		11,068,157	(490,341)	2,252	10,580,068	11,177,698	(533,788)	(3,359)	10,640,551
Foreign Securities									
Government securities		10,606,326	(27,281)	17,509	10,596,554	11,517,802	(2,828)	13,295	11,528,269
Non Government Debt securities		–	–	–	–	452,567	(741)	–	451,826
Unlisted equity securities		7,260	(1,714)	–	5,546	7,250	–	–	7,250
		10,613,586	(28,995)	17,509	10,602,100	11,977,619	(3,569)	13,295	11,987,345
Associates									
- Adamjee Insurance Company Limited	10.8	647,880	–	–	647,880	647,880	–	–	647,880
- Euronet Pakistan (Private) Limited		52,521	–	–	52,521	52,521	–	–	52,521
		700,401	–	–	700,401	700,401	–	–	700,401
Subsidiaries									
MCB Islamic Bank Limited		11,550,000	–	–	11,550,000	11,550,000	–	–	11,550,000
MCB Arif Habib Savings & Investments Limited		320,123	–	–	320,123	320,123	–	–	320,123
Financial Management Services (Pvt) Limited	10.9	725	(725)	–	–	725	(725)	–	–
MCB Non-Bank Credit Organization Closed Joint Stock Company		448,189	–	–	448,189	448,189	–	–	448,189
MCB Financial Services Limited	10.10	–	–	–	–	27,500	–	–	27,500
		12,319,037	(725)	–	12,318,312	12,346,537	(725)	–	12,345,812
Total Investments		1,013,846,041	(10,652,723)	12,676,130	1,015,869,448	752,969,008	(10,689,171)	6,484,665	748,764,502

Notes To The Unconsolidated Financial Statements

For the year ended December 31, 2020

		Note	2020	2019
			(Rupees in '000)	
**10.2.1**	**Investments given as collateral**			
	- Market Treasury Bills		91,279,273	22,820,226
	- Pakistan Investment Bonds		1,000,283	5,316,208
			92,279,556	28,136,434
**10.3**	**Provision for diminution in value of investments**			
**10.3.1**	Opening balance		10,689,171	7,865,388
	Exchange and other adjustments		13,474	251
	Charge / (reversals)			
	Charge for the year		1,956,360	3,275,539
	Reversals for the year		(1,529)	(29,964)
	Reversal on disposals		(2,004,753)	(422,043)
			(49,922)	2,823,532
	Amounts written off		–	–
	Closing balance	10.3.3	10,652,723	10,689,171

10.3.2 Particulars of provision against debt securities

Category of classification

	2020		2019	
	NPI	Provision	NPI	Provision
	(Rupees in '000)			
**Domestic**				
Doubtfull	–	–	145,656	72,828
Loss	490,459	490,459	461,078	461,078
	490,459	490,459	606,734	533,906

10.3.3 This includes a general provision of Rs 43.542 million (December 31, 2019: Rs 5.780 million) held by overseas branches in accordance with the requirements of IFRS 9.

10.4 Quality of Available for Sale Securities

Details regarding quality of Available for Sale (AFS) securities are as follows;

	2020	2019
	(Rupees in '000)	
**Federal Government Securities - Government guaranteed**		
Market Treasury Bills	597,161,075	386,882,153
Pakistan Investment Bonds	346,405,148	278,739,813
Euro Bonds	1,341,706	506,323
Sukuk Bonds	1,267,440	–
Naya Pakistan Certificates	465,779	–
	946,641,148	666,128,289

Notes To The Unconsolidated Financial Statements

For the year ended December 31, 2020

	2020	2019
	(Rupees in '000)	
**Listed Companies and mutual funds**		
Automobile Assembler	1,418,843	1,230,989
Automobile Part and Accessories	413,930	413,930
Cable and Electrical Goods	523,036	523,036
Cement	2,050,172	2,100,969
Chemical	285,638	75,680
Close end Mutual Fund	1,186,851	1,186,851
Commercial Banks	3,002,215	2,014,437
Engineering	1,587,837	1,818,843
Fertilizer	3,298,245	2,876,608
Food and Personal Care Products	1,229,398	1,199,266
Insurance	599,364	599,364
Investment Banks / Companies	41,784	41,784
NIT Units	5,253	5,253
Oil & Gas Exploration Companies	2,747,824	2,533,285
Oil & Gas Marketing Companies	452,460	434,633
Open End Mutual Fund	190,083	312,349
Paper and Board	340,473	391,951
Pharmaceutical	941,959	1,068,443
Power Generation and Distribution	2,516,198	2,516,198
Refinery	1,013,306	887,318
Technology and Communication	707,827	706,796
Textile	446,942	446,942
	24,999,638	23,384,925

	2020		2019	
	Cost	Breakup value	Cost	Breakup value
	(Rupees in '000)			
**Unlisted Companies**				
Central Depository Company Limited	184,426	756,153	184,426	687,011
First Capital Investment (Pvt) Limited	2,500	2,667	2,500	3,334
First Women Bank Limited	63,300	215,838	63,300	215,838
ISE Towers Reit Management Company Limited	30,346	93,780	30,346	87,958
National Investment Trust Limited	1,027,651	1,661,565	1,027,651	1,665,882
National Institutional Facilitation Technologies	1,527	51,998	1,527	51,641
Pak Agro Storage And Service Corporation	2,500	1,239,050	2,500	605,006
1 Link (Pvt) Limited	50,000	202,032	50,000	147,332
Naymat Collateral Management Company	29,285	25,876	–	–
Arabian Sea Country Club	5,000	–	5,000	–
SME Bank Limited	10,106	–	10,106	–
Al-Ameen Textile Mills Limited	197	–	197	–
Custodian Management Services	1,000	–	1,000	–
Galaxy Textile Mills Limited	30,178	–	30,178	–
Pakistan Textile City (Pvt) Limited	50,000	–	50,000	–
Ayaz Textile Mills Limited	2,253	–	2,253	–
Musarrat Textile Mills Limited	36,045	–	36,045	–
Sadiqabad Textile Mills Limited	26,361	–	26,361	–
Al-Arabia Sugar Mills Limited - Preference shares	4,775	–	4,775	–
Pak Elektron Limited - Preference shares	25,000	25,000	25,000	25,000
	1,582,450	4,273,959	1,553,165	3,489,002

Notes To The Unconsolidated Financial Statements

For the year ended December 31, 2020

	2020	2019
	Cost	
	(Rupees in '000)	
**Non Government Debt Securities**		
**Listed**		
- AA+, AA, AA-	1,547,840	957,840
**Unlisted**		
- AA+, AA, AA-	250,000	250,000
- A+, A, A-	–	101,933
	250,000	351,933

	2020		2019	
	Cost	Rating	Cost	Rating
	(Rupees in '000)			
**Foreign Securities**				
**Government Securities**				
- Bahrain	–	–	–	–
- Sri Lanka	7,456,679	Caa1	3,649,770	B2
	7,456,679		3,649,770	

	2020	2019
	Cost	
	(Rupees in '000)	
**Unlisted**		
Lanka Clear (Private) Limited	857	854
Credit Information Bureau of Sri Lanka	26	26
Lanka Financial Services Bureau Limited	1,714	1,707
Society for Worldwide Inter Fund Transfer (SWIFT)	4,663	4,663
	7,260	7,250

10.5 Particulars relating to Held to Maturity securities are as follows:

	2020	2019
**Federal Government Securities - Government guaranteed**		
Pakistan Investment Bonds	3,333,719	13,842,253
Market Treasury Bills	–	1,645,036
Sukuk bonds	523,680	195,928
Euro Bonds	754,991	524,032
	4,612,390	16,207,249
**Provincial Government Securities**	118	118

Notes To The Unconsolidated Financial Statements

For the year ended December 31, 2020

	2020	2019
	Cost	
	(Rupees in '000)	
**Non Government Debt Securities**		
**Listed**		
- AAA	1,796,250	1,796,970
- AA+, AA, AA-	1,644,520	2,125,716
- Unrated	66,120	66,120
	3,506,890	3,988,806
**Unlisted**		
- AA+, AA, AA-	4,849,483	4,848,863
- A+, A, A-	439,874	439,910
- Unrated	474,070	590,346
	5,763,427	5,879,119

	2020		2019	
	Cost	Rating	Cost	Rating
	(Rupees in '000)			
**Foreign Securities**				
**Government Securities**				
- Sri Lanka	3,149,647	Caa1	1,544,542	B2
- United Arad Emirates	–		6,323,490	Aa2
	3,149,647		7,868,032	

	2020	2019
	Cost	
	(Rupees in '000)	
**NonGovernment Debt Securities**		
**Listed**		
- A+, A, A-	–	153,702
**Unlisted**		
- AAA		
	–	298,865
	–	298,865

10.5.1 The market value of securities classified as held-to-maturity as at December 31, 2020 amounted to Rs. 17,002.908 million (December 31, 2019: Rs. 33,464.168 million).

10.6 Available for sale" Market Treasury Bills and Pakistan Investment Bonds are eligible for rediscounting with the State Bank of Pakistan.

Notes To The Unconsolidated Financial Statements

For the year ended December 31, 2020

10.7 Investments include Pakistan Investment Bonds amounting to Rs. 67.9 million (2019: Rs. 67.9 million) earmarked by the SBP against TT discounting facilities sanctioned to the Bank. In addition, Pakistan Investment Bonds amounting to Rs. 5 million (2019: Rs. 5 million) have been pledged with the Controller of Military Accounts on account of Regimental Fund account and Pakistan Investment Bonds amounting to Rs.100 million (2019: Rs. 100 million) have been pledged with the National Clearing Company of Pakistan Limited (NCCPL) on account of removal of irrevocable undertaking as alternate option for collateral against participant's exposure in stock market.

10.8 Investment of the Bank in Adamjee Insurance Company Limited is carried at cost amounting to Rs. 647.880 million (2019: Rs. 647.880 million) as at December 31, 2020. The market value of the investment in Adamjee Insurance Company Limited as at December 31, 2020 amounted to Rs. 2,752.400 million (2019: Rs. 2,946.300 million).

10.9 This investment is fully provided for. The company is dormant and has no asset and liability.

10.10 During the year, the Bank has disposed off its wholly owned subsidiary "MCB Financial Services Limited".

10.11 Certain approved / Government securities are kept with the SBP to meet statutory liquidity requirements calculated on the basis of domestic demand and time liabilities.

10.12 Summarized financial information of associates and subsidiaries

	Country of incorporation	% of interest held	Revenue	Profit / (loss) after tax	Assets	Liabilities
	(Rupees in '000)					
**2020**						
**Associates**						
Euronet Pakistan (Private) Limited (unaudited based on December 31, 2020)	Pakistan	30%	718,076	(59,342)	870,136	722,516
Adamjee Insurance Company Limited (unaudited based on September 30, 2020)	Pakistan	20%	20,596,203	1,319,951	95,997,472	74,918,209
**Subsidiaries**						
MCB Islamic Bank Limited (audited based on December 31, 2020)	Pakistan	100.00%	9,676,435	208,316	141,170,886	130,443,114
MCB Arif Habib Savings & Investments Limited (audited based on June 30, 2020)	Pakistan	51.33%	835,520	257,669	2,265,572	714,877
MCB Non-Bank Credit Organization Closed Joint Stock Company (audited based on December 31, 2020)	Azerbaijan	99.94%	163,849	46,863	825,024	400,034

Notes To The Unconsolidated Financial Statements

For the year ended December 31, 2020

	Country of incorporation	% of interest held	Revenue	Profit / (loss) after tax	Assets	Liabilities
			(Rupees in '000)			
**2019**						
**Associates**						
Euronet Pakistan (Private) Limited (unaudited based on December 31, 2019)	Pakistan	30%	557,579	25,769	648,477	435,306
Adamjee Insurance Company Limited (unaudited based on September 30, 2019)	Pakistan	20%	19,551,861	1,108,984	82,967,479	63,892,816
**Subsidiaries**						
MCB Islamic Bank Limited (audited based on December 31, 2019)	Pakistan	100.00%	9,848,819	(243,612)	105,017,261	94,550,460
MCB Arif Habib Savings & Investments Limited (audited based on June 30, 2019)	Pakistan	51.33%	715,079	24,235	2,044,323	582,097
MCB Non-Bank Credit Organization Closed Joint Stock Company (audited based on December 31, 2019)	Azerbaijan	99.94%	155,085	55,014	943,878	576,914
MCB Financial Services Limited (audited based on December 31, 2019)	Pakistan	100.00%	41,537	11,442	96,910	3,956

11. ADVANCES

	Note	Performing		Non Performing		Total	
		2020	2019	2020	2019	2020	2019
				(Rupees in '000)			
Loans, cash credits, running finances, etc.	11.1	444,168,998	471,126,199	50,524,753	48,759,157	494,693,751	519,885,356
Bills discounted and purchased		18,192,157	19,486,758	664,294	665,102	18,856,451	20,151,860
Advances - gross		462,361,155	490,612,957	51,189,047	49,424,259	513,550,202	540,037,216
Provision against advances							
- Specific		–	–	(45,142,956)	(41,934,421)	(45,142,956)	(41,934,421)
- General	11.4.4	(5,465,459)	(1,423,921)	–	–	(5,465,459)	(1,423,921)
		(5,465,459)	(1,423,921)	(45,142,956)	(41,934,421)	(50,608,415)	(43,358,342)
Advances - net of provision		456,895,696	489,189,036	6,046,091	7,489,838	462,941,787	496,678,874

Notes To The Unconsolidated Financial Statements

For the year ended December 31, 2020

11.1 Includes net investment in finance lease as disclosed below:

	2020				2019			
	Not later than one year	Late than one and less than five years	Over five year	Total	Not later than one year	Late than one and less than five years	Over five year	Total
	(Rupees in '000)							
Lease rentals receivable	1,521,860	1,436,815	1,133,794	4,092,469	56,237	1,972,680	2,365,197	4,394,114
Residual value	20,428	103,490	23,039	146,957	14,769	35,544	10,368	60,681
Minimum lease payments	1,542,288	1,540,305	1,156,833	4,239,426	71,006	2,008,224	2,375,565	4,454,795
Financial charges for future periods	(3,770)	(255,311)	(418,859)	(677,940)	(3,376)	(247,521)	(517,870)	(768,767)
Present value of minimum lease payments	1,538,518	1,284,994	737,974	3,561,486	67,630	1,760,703	1,857,695	3,686,028

	2020	2019
	(Rupees in '000)	
**11.2 Particulars of advances (Gross)**		
In local currency	469,211,685	481,408,603
In foreign currencies	44,338,517	58,628,613
	513,550,202	540,037,216

11.3 Advances include Rs. 51,189.047 million (2019: Rs.49,424.259 million) which have been placed under the non-performing status as detailed below:

	Note	2020 Non performing loans	2020 Provision	2019 Non performing loans	2019 Provision
		(Rupees in '000)			
**Category of Classification**					
**Domestic**					
Other Assets Especially Mentioned	11.3.1	43,508	1,983	123,678	3,529
Substandard		211,900	52,156	584,129	145,075
Doubtful		264,759	132,380	2,690,841	1,345,421
Loss		42,224,438	41,485,949	37,835,619	37,087,269
		42,744,605	41,672,468	41,234,267	38,581,294
**Overseas**					
Not past due but impaired		–	–	–	–
Overdue by:					
Upto 90 days		5,321	4,913	10,688	7,400
91 to 180 days		2,020	505	118,182	95,452
181 to 365 days		19,961	9,981	141,231	137,601
> 365 days		8,417,140	3,455,089	7,919,891	3,112,674
		8,444,442	3,470,488	8,189,992	3,353,127
Total		51,189,047	45,142,956	49,424,259	41,934,421

11.3.1 This represents non-performing portfolio of agricultural and small enterprise financing classified as OAEM as per the requirements of the Prudential Regulation for Agricultural and Small Enterprise Financing issued by the State Bank of Pakistan.

Notes To The Unconsolidated Financial Statements
For the year ended December 31, 2020

11.4 Particulars of provision against advances

	Note	2020 Specific	2020 General	2020 Total	2019 Specific	2019 General	2019 Total
		(Rupees in '000)					
Opening balance		41,934,421	1,423,921	43,358,342	41,943,509	1,266,717	43,210,226
Exchange adjustments		50,555	7,065	57,620	302,297	21,237	323,534
Charge for the year		5,703,057	4,097,524	9,800,581	3,356,159	155,449	3,511,608
Reversals	11.4.3	(2,215,829)	(63,051)	(2,278,880)	(3,649,742)	(19,482)	(3,669,224)
		3,487,228	4,034,473	7,521,701	(293,583)	135,967	(157,616)
Amounts written off	11.5	(329,248)	–	(329,248)	(17,802)	–	(17,802)
Closing balance		45,142,956	5,465,459	50,608,415	41,934,421	1,423,921	43,358,342

11.4.1 Particulars of provision against advances

	2020 Specific	2020 General	2020 Total	2019 Specific	2019 General	2019 Total
	(Rupees in '000)					
In local currency	41,672,468	5,148,833	46,821,301	38,581,294	1,087,464	39,668,758
In foreign currencies	3,470,488	316,626	3,787,114	3,353,127	336,457	3,689,584
	45,142,956	5,465,459	50,608,415	41,934,421	1,423,921	43,358,342

11.4.2 State Bank of Pakistan vide BSD Circular No. 2 dated January 27, 2009, BSD Circular No. 10 dated October 20, 2009, BSD Circular No. 02 of 2010 dated June 03, 2010 and BSD Circular No.1 of 2011 dated October 21, 2011 has allowed benefit of forced sale value (FSV) of Plant & Machinery under charge, pledged stock and mortgaged residential, commercial & industrial properties (land and building only) held as collateral against NPLs for five years from the date of classification. However, management has not taken the FSV benefit in calculation of specific provision.

11.4.3 This includes reversal of provisions and reduction of non-performing loans amounting to Rs. 84 million (2019: Rs. NIL) as a result of settlement on debt asset swap arrangement with customers.

11.4.4 General provision against consumer loans represents provision maintained against fully secured performing portfolio and unsecured performing portfolio as required by the Prudential Regulations issued by the SBP. General provision against Small Enterprise Finance represents provision maintained at an amount equal to 1% of unsecured performing portfolio as required by the Prudential Regulations issued by the SBP. General provisions pertaining to overseas advances are made in accordance with the requirements of the regulatory authorities of the respective countries in which the overseas branches operate.

In addition, the Bank has also maintained a general provision of Rs 4,564.689 million (December 31, 2019: Rs 527 million) against financing made on prudent basis. This general provision is in addition to the requirements of Prudential Regulations. Also refer note 5 to these unconsolidated financial statements.

		Note	2020	2019
			(Rupees in '000)	
**11.5**	**PARTICULARS OF WRITE OFFs:**			
**11.5.1**	Against Provisions	11.4	329,248	17,802
	Directly charged to Profit & Loss account		–	–
			329,248	17,802
**11.5.2**	Write Offs of Rs. 500,000 and above			
	- Domestic	11.6	299,535	14,613
	- Overseas		–	2,725
	Write Offs of below Rs. 500,000		29,713	464
			329,248	17,802

Notes To The Unconsolidated Financial Statements

For the year ended December 31, 2020

11.6 DETAILS OF LOAN WRITE OFF OF Rs. 500,000/- AND ABOVE

In terms of sub-section (3) of Section 33A of the Banking Companies Ordinance, 1962, the statement in respect of written-off loans or any other financial relief of Rupees five hundred thousand or above allowed to a person(s) during the year ended December 31, 2020 is given at Annexure I of the unconsolidated financial statements. However, this write off does not affect the Bank's right to recover the debts from these customers.

	Note	2020	2019
		(Rupees in '000)	
**12. FIXED ASSETS**			
Capital work-in-progress	12.1	802,966	975,566
Property and equipment	12.2	50,467,607	49,620,934
Right-of-use assets	12.3	6,757,331	7,674,745
		58,027,904	58,271,245
**12.1 Capital work-in-progress**			
Civil works		418,187	451,189
Equipment		98,383	90,946
Advances to suppliers		283,029	428,617
Others		3,367	4,814
		802,966	975,566

12.2 Property and Equipment

	2020								
	Freehold land	Leasehold land	Building on Freehold land	Building on Leasehold land	Furniture and fixtures	Electrical, office and computer equipment	Vehicles	Leasehold improvement	Total
	(Rupees in '000)								
**At January 1, 2020**									
Cost / Revalued amount	26,123,665	2,893,079	13,898,388	637,102	1,860,523	14,508,613	1,070,928	854,594	61,846,892
Accumulated depreciation	–	–	–	–	(1,107,450)	(10,205,701)	(603,975)	(308,832)	(12,225,958)
Net book value	26,123,665	2,893,079	13,898,388	637,102	753,073	4,302,912	466,953	545,762	49,620,934
**Year ended December 31, 2020**									
Opening net book value	26,123,665	2,893,079	13,898,388	637,102	753,073	4,302,912	466,953	545,762	49,620,934
Additions	189,607	6,999	975,707	168,601	209,610	985,975	56,916	370,722	2,964,137
Disposals	–	–	(85,546)	–	(488)	(4,336)	(23,938)	–	(114,308)
Depreciation charge	–	–	(487,380)	(28,597)	(141,646)	(1,112,379)	(109,472)	(125,587)	(2,005,061)
Exchange rate adjustments	–	–	413	691	256	582	335	(372)	1,905
Transfers	–	–	(17,242)	–	61	(61)	–	17,242	-
Closing net book value	26,313,272	2,900,078	14,284,340	777,797	820,866	4,172,693	390,794	807,767	50,467,607
**At December 31, 2020**									
Cost / Revalued amount	26,313,272	2,900,078	14,770,261	806,387	2,052,270	15,340,049	1,012,162	1,217,035	64,411,514
Accumulated depreciation	–	–	(485,921)	(28,590)	(1,231,404)	(11,167,356)	(621,368)	(409,268)	(13,943,907)
Net book value	26,313,272	2,900,078	14,284,340	777,797	820,866	4,172,693	390,794	807,767	50,467,607
Rate of depreciation / estimated useful life	–	–	Upto 60 years	Upto 50 years	10%	10%-25%	20%	Lease term	–

Notes To The Unconsolidated Financial Statements

For the year ended December 31, 2020

	2019								
	Freehold land	Leasehold land	Building on Freehold land	Building on Leasehold land	Furniture and fixtures	Electrical, office and computer equipment	Vehicles	Leasehold improvement	Total
	(Rupees in '000)								
**At January 1, 2019**									
Cost / Revalued amount	19,556,136	2,440,151	12,138,906	563,273	1,689,662	13,954,280	1,149,293	521,985	52,013,686
Accumulated depreciation	-	-	(865,869)	(116,820)	(996,689)	(9,595,619)	(613,480)	(252,675)	(12,441,152)
Net book value	19,556,136	2,440,151	11,273,037	446,453	692,973	4,358,661	535,813	269,310	39,572,534
**Year ended December 31, 2019**									
Opening net book value	19,556,136	2,440,151	11,273,037	446,453	692,973	4,358,661	535,813	269,310	39,572,534
Additions	805,354	-	1,978,039	142,646	192,157	1,088,501	100,175	352,874	4,659,746
Movement in surplus on assets revalued during the year	5,744,825	452,928	1,019,986	73,227	-	-	-	-	7,290,966
Transfer from Non-Banking assets	34,000	-	17,520	-	-	-	-	-	51,520
Disposal	(21,000)	-	-	-	(1,306)	(4,777)	(40,683)	(262)	(68,028)
Depreciation charge	-	-	(406,881)	(27,778)	(132,669)	(1,147,355)	(132,259)	(69,704)	(1,916,646)
Exchange rate adjustments	-	-	9,566	2,554	3,167	6,633	3,907	5,015	30,842
Transfers	4,350	-	7,121	-	(1,249)	1,249	-	(11,471)	-
Closing net book value	26,123,665	2,893,079	13,898,388	637,102	753,073	4,302,912	466,953	545,762	49,620,934
**At December 31, 2019**									
Cost / Revalued amount	26,123,665	2,893,079	13,898,388	637,102	1,860,523	14,508,613	1,070,928	854,594	61,846,892
Accumulated depreciation	-	-	-	-	(1,107,450)	(10,205,701)	(603,975)	(308,832)	(12,225,958)
Net book value	26,123,665	2,893,079	13,898,388	637,102	753,073	4,302,912	466,953	545,762	49,620,934
Rate of depreciation / estimated useful life	-	-	Upto 60 years	Upto 50 years	10%	10%-25%	20%	Lease term	-

12.2.1 Leasehold land include a plot of land measuring 3,120.46 square yards having book value of Rs. 1,419.809 million situated at Railway Quarters, I.I. Chundrigar Road, Karachi, (the "Plot"), where a tenant is claiming for the possession as tenant of an insignificant area of only 18 square feet of the plot, however there is no issue over the title of the subject property. Both the Constitutional Petitions filed by the Bank have been dismissed by the Sindh High Court on 28 January 2016 against the Bank. The Bank has filed an appeal before the Supreme Court of Pakistan.

12.2.2 The land and buildings of the Bank were revalued as at December 31, 2019 by independent valuers (K.G. Traders (Pvt) Limited, Tristar International Consultant (Pvt) Limited & Sardar Enterprises), valuation and engineering consultants, on the basis of market value. The total surplus against revaluation of fixed assets as at December 31, 2020 amounts to Rs. 20,211.952 million.

12.2.3 Had the land and buildings not been revalued, the total carrying amounts of revalued properties as at December 31, 2020 would have been as follows:

	(Rupees in '000)
Land	12,676,870
Buildings	11,386,665

Notes To The Unconsolidated Financial Statements
For the year ended December 31, 2020

12.2.4 The gross carrying amount (cost) of fully depreciated assets that are still in use are as follows:

	(Rupees in '000)
Furniture and fixtures	604,447
Electrical, computers and office equipment	7,251,702
Vehicles	474,229

12.2.5 Carrying amount of temporarily idle property of the Bank is Rs. 436.136 million (2019: Rs. 161.886 million).

12.2.6 The information relating to disposal of operating fixed assets to related parties is given in Annexure II of these unconsolidated financial statements.

	Note	2020	2019
		(Rupees in '000)	
**12.3 Movement in right-of-use assets is as follows:**			
Opening balance		7,674,745	8,426,460
Additions / Adjustments		734,068	410,080
Derecognition		(434,698)	–
Depreciation charge	31	(1,216,784)	(1,161,795)
Closing net book value		6,757,331	7,674,745
**13. INTANGIBLE ASSETS**			
Capital work-in-progress		394,643	316,742
Computer software	13.1	543,815	640,810
		938,458	957,552

	2020	2019
	(Rupees in '000)	
	Computer software	
**13.1 At January 1**		
Cost	3,963,740	3,380,565
Accumulated amortisation	(3,322,930)	(3,008,229)
Net book value	640,810	372,336
**Year ended December 31**		
Opening net book value	640,810	372,336
Additions	220,979	566,863
Amortisation charge	(318,024)	(301,022)
Exchange rate adjustments	50	2,633
Closing net book value	543,815	640,810
**At December 31**		
Cost	4,185,598	3,963,740
Accumulated amortisation and impairment	(3,641,783)	(3,322,930)
Net book value	543,815	640,810
Rate of amortisation (percentage)	14% to 33.33%	14% to 33.33%
Useful life	3 - 7 years	3 - 7 years

13.2 The gross carrying amount (cost) of fully depreciated intangible assets that are still in use is Rs. 3,085.368 million.

Notes To The Unconsolidated Financial Statements

For the year ended December 31, 2020

	Note	2020	2019
		(Rupees in '000)	
**14. OTHER ASSETS**			
Income/ mark-up accrued in local currency		17,085,615	22,099,766
Income/ mark-up accrued in foreign currencies		305,759	558,407
Advances, deposits, advance rent and other prepayments		2,249,497	3,720,012
Advance taxation (payments less provisions)		–	–
Compensation for delayed income tax refunds		133,809	133,809
Non-banking assets acquired in satisfaction of claims	14.1	3,277,778	3,251,508
Branch adjustment account		276,102	37,075
Mark to market gain on forward foreign exchange contracts		4,854,527	3,875,681
Unrealized gain on derivative financial instruments	25	517,033	1,236,517
Acceptances	20	20,030,754	18,152,032
Receivable from the pension fund	38.4	3,370,179	3,605,121
Others		5,961,655	9,399,297
		58,062,708	66,069,225
Less: Provision held against other assets	14.2	2,582,686	2,604,137
Other Assets (net of provision)		55,480,022	63,465,088
Surplus on revaluation of non-banking assets acquired in satisfaction of claims		854,231	677,660
Other Assets - total		56,334,253	64,142,748
**14.1** Market value of Non-banking assets acquired in satisfaction of claims		4,036,914	3,838,230

Non-banking assets acquired in satisfaction of claims of the Bank are revalued as at December 31, 2020 by independent valuers (Tristar International Consultant, Design Crafts, Material Design Services, Pee Dee & Associates and J&M Associates) on the basis of market value.

	Note	2020	2019
		(Rupees in '000)	
**14.1.1 Non-banking assets acquired in satisfaction of claims**			
Opening balance		3,838,230	4,035,114
Additions		84,000	64,445
Revaluation		183,915	255,040
Disposals		(35,024)	(428,052)
Depreciation		(30,049)	(45,456)
(Charge) / reversal of impairment		(4,158)	8,659
Transfer to fixed assets		–	(51,520)
Closing balance		4,036,914	3,838,230
**14.1.2 Gain on disposal of non-banking assets acquired in satisfaction of claims**			
Disposal proceeds		39,000	540,000
Less			
- Cost		35,820	433,813
- Depreciation		(796)	(5,761)
		35,024	428,052
Gain	14.1.2.1	3,976	111,948

Notes To The Unconsolidated Financial Statements

For the year ended December 31, 2020

	Note	2020	2019
		(Rupees in '000)	
**14.1.2.1 Breakup of gain/loss realized on sale of non-banking assets:**			
Commercial property Rawalpindi		3,976	–
Industrial property Lahore		–	111,948
		3,976	111,948
**14.2 Provision held against other assets**			
Non banking assets acquired in satisfaction of claims		95,095	90,938
Others		2,487,591	2,513,199
		2,582,686	2,604,137
**14.2.1 Movement in provision held against other assets**			
Opening balance		2,604,137	2,550,584
Charge for the year		54,269	12,587
Reversals		(77,917)	(36,023)
	33	(23,648)	(23,436)
Amount written off		(16,591)	(3,638)
Exchange and other adjustments		18,788	80,627
Closing balance		2,582,686	2,604,137

15. CONTINGENT ASSETS

There were no contingent assets of the Bank as at December 31, 2020 (2019: NIL).

	Note	2020	2019
		(Rupees in '000)	
**16. BILLS PAYABLE**			
In Pakistan		23,912,803	11,786,207
Outside Pakistan		67,889	35,491
		23,980,692	11,821,698
**17. BORROWINGS**			
**Secured**			
Borrowings from State Bank of Pakistan			
Under export refinance scheme	17.1	34,998,802	33,862,262
Under long term financing facility	17.2	22,150,335	18,138,200
Under renewable energy performance platform	17.3	74,760	85,062
Refinance Scheme for wages and salaries	17.4	10,074,011	–
Temporary economic refinance facility	17.5	1,694,659	–
Under financing facility for storage of agricultural produce	17.6	191,254	188,809
		69,183,821	52,274,333
Repurchase agreement borrowings	17.7	92,225,530	28,099,229
**Total secured**		161,409,351	80,373,562
**Unsecured**			
Borrowings from other financial institution	17.8	1,712,914	774,914
Call borrowings	17.9	319,669	6,845,683
Overdrawn nostro accounts		397,313	1,349,447
Others		162,286	162,286
**Total unsecured**		2,592,182	9,132,330
	17.10	164,001,533	89,505,892

Notes To The Unconsolidated Financial Statements

For the year ended December 31, 2020

17.1 The Bank has entered into agreements for financing with the State Bank of Pakistan (SBP) for extending export finance to customers. As per the agreements, the Bank has granted SBP the right to recover the outstanding amount from the Bank at the date of maturity of the finance by directly debiting the current account maintained by the Bank with SBP. These borrowings are repayable within six months. These carry mark up rates ranging from 1.0% to 2.0% per annum.

17.2 These borrowings have been obtained from the SBP for providing financing facilities to exporters for adoption of new technologies and modernization of their plant and machinery. These borrowings are repayable within a period ranging from 3 years to 10 years. These carry mark up rates ranging from 2.0% to 3.50% per annum.

17.3 These borrowings have been obtained from the SBP for providing financing facilities to customers against renewable energy projects. These borrowings are repayable within a period of twelve years with two years grace period from date of disbursement. These carry mark up rate of 2% per annum.

17.4 These borrowings have been obtained from the SBP for providing financing facilities to help businesses in payment of wages and salaries to their workers and employees for supporting continued employment. These borrowing are repayable in 8 equal quarterly instalments begining from January 2021. These carry mark up rates ranging from 0% to 2% per annum.

17.5 These borrowings have been obtained from the SBP for providing concessionary refinancing facility to the industry for purchase of new imported and locally manufactured plant & machinery to set up new projects. These borrowings are repayable within a period of ten years including a grace period of upto 2 years. These carry mark up rate of 1% per annum.

17.6 These borrowings have been obtained from SBP under “Financing Facility for Storage of Agricultural Produce (FFSAP)” to encourage Private Sector to establish Silos, Warehouses and Cold Storages. These borrowings are repayable within a period ranging from 3 years to 10 years. These carry mark up rates ranging from 2.50% to 3.50% per annum.

17.7 These carry mark-up rates ranging from 6.15% to 7.25% per annum (2019:8.0% to 13.20% per annum) and are secured against government securities of carrying value of Rs. 92,279.556 million (2019: Rs. 28,136.434 million). These are repayable latest by February 2021.

17.8 These carry mark-up rates ranging from 1.90% to 4.00% per annum (2019: 2.75% to 3.00% per annum). These are repayable latest by August 2022.

17.9 These carry mark-up at the rate of 1.15% per annum (2019: 2.30% to 13.10% per annum). These are repayable by January 2021.

	2020	2019
	(Rupees in '000)	
**17.10 Particulars of borrowings with respect to currencies**		
In local currency	161,494,499	85,452,572
In foreign currencies	2,507,034	4,053,320
	164,001,533	89,505,892

Notes To The Unconsolidated Financial Statements

For the year ended December 31, 2020

18. DEPOSITS AND OTHER ACCOUNTS

	2020			2019		
	In Local currency	In Foreign currencies	Total	In Local currency	In Foreign currencies	Total
	(Rupees in '000)					
**Customers**						
Current deposits	397,258,854	53,823,514	451,082,368	336,833,189	51,619,020	388,452,209
Savings deposits	649,410,009	45,805,328	695,215,337	552,121,108	45,357,701	597,478,809
Term deposits	71,492,234	14,103,734	85,595,968	85,296,905	15,518,802	100,815,707
Others	23,319,545	3,218,232	26,537,777	21,537,429	2,854,010	24,391,439
	1,141,480,642	116,950,808	1,258,431,450	995,788,631	115,349,533	1,111,138,164
**Financial Institutions**						
Current deposits	10,885,621	659,092	11,544,713	9,421,664	2,093,098	11,514,762
Savings deposits	14,068,212	115,935	14,184,147	13,005,530	26,432	13,031,962
Term deposits	1,030,968	4,090,613	5,121,581	1,143,468	7,741,444	8,884,912
Others	–	220,413	220,413	–	193,459	193,459
	25,984,801	5,086,053	31,070,854	23,570,662	10,054,433	33,625,095
	1,167,465,443	122,036,861	1,289,502,304	1,019,359,293	125,403,966	1,144,763,259

		2020	2019
		(Rupees in '000)	
18.1	Composition of deposits		
	- Individuals	829,982,651	716,900,889
	- Government (Federal and Provincial)	55,860,439	65,583,218
	- Public Sector Entities	82,241,309	52,680,247
	- Banking Companies	4,909,064	9,205,676
	- Non-Banking Financial Institutions	26,161,790	24,419,419
	- Private Sector	290,347,051	275,973,810
		1,289,502,304	1,144,763,259

18.2 Deposits and other accounts include deposits eligible to be covered under the Deposits Protection insurance arrangements amounting to Rs 895,879.416 million (2019: Rs 751,072.744 million).

Notes To The Unconsolidated Financial Statements

For the year ended December 31, 2020

19. DEFERRED TAX LIABILITIES

	Note	2020			
		At January 01, 2020	Recognised in P&L A/C	Recognised in OCI	At December 01, 2020
		(Rupees in '000)			
**Taxable Temporary Differences on**					
- Surplus on revaluation of fixed assets	23.1	1,346,550	(60,134)	–	1,286,416
- Surplus on revaluation of Non-banking assets	23.2	237,181	(2,570)	64,371	298,982
- Accelerated tax depreciation		1,754,097	96,692	–	1,850,789
- Receivable from pension fund		1,261,793	119,428	(201,659)	1,179,562
- Business combination		705,218	–	–	705,218
- Surplus on revaluation of investments	23	2,271,094	–	2,165,627	4,436,721
		7,575,933	153,416	2,028,339	9,757,688
**Deductible Temporary Differences on**					
- Provision against advances		(1,725,288)	(1,057,242)	–	(2,782,530)
- Deficit on revaluation of investments	23	–	–	–	–
		(1,725,288)	(1,057,242)	–	(2,782,530)
		5,850,645	(903,826)	2,028,339	6,975,158

	Note	2019			
		At January 01, 2019	Recognised in P&L A/C	Recognised in OCI	At December 01, 2019
		(Rupees in '000)			
**Taxable Temporary Differences on**					
- Surplus on revaluation of fixed assets	23.1	992,684	(28,759)	382,625	1,346,550
- Surplus on revaluation of non-banking assets	23.2	168,305	(20,388)	89,264	237,181
- Accelerated tax depreciation		1,607,561	146,536	–	1,754,097
- Receivable from pension fund		1,335,309	155,621	(229,137)	1,261,793
- Business combination		705,218	–	–	705,218
- Surplus on revaluation of investments	23	–	–	2,271,094	2,271,094
		4,809,077	253,010	2,513,846	7,575,933
**Deductible Temporary Differences on**					
- Provision against advances		(1,791,747)	66,459	–	(1,725,288)
- Deficit on revaluation of investments		(1,485,153)	–	1,485,153	–
		(3,276,900)	66,459	1,485,153	(1,725,288)
		1,532,177	319,469	3,998,999	5,850,645

Notes To The Unconsolidated Financial Statements

For the year ended December 31, 2020

		Note	2020	2019
			(Rupees in '000)	
**20.**	**OTHER LIABILITIES**			
	Mark-up/ return/ interest payable in local currency		2,272,650	22,831,727
	Mark-up/ return/ interest payable in foreign currencies		247,926	649,536
	Unearned commission and income on bills discounted		212,337	181,751
	Accrued expenses		5,898,224	5,397,614
	Provision for taxation (provisions less payments)		10,185,375	6,130,846
	Workers' welfare fund	20.1	8,838,684	7,873,706
	Acceptances	14	20,030,754	18,152,032
	Unclaimed/dividend payable		1,692,942	1,592,979
	Mark to market loss on forward foreign exchange contracts		4,485,302	4,642,692
	Unrealized loss on derivative financial instruments	25	513,343	1,232,806
	Staff welfare fund		5,598	5,727
	Provision for employees' compensated absences	38.4	919,407	939,495
	Provision for post retirement medical benefits	38.4	2,004,122	1,921,348
	Provision for employees' contributory benevolent scheme	38.4	222,084	221,193
	Retention money		20,657	20,657
	Insurance payable against consumer assets		698,949	655,146
	Unclaimed balances		877,552	993,105
	Duties and taxes payable		1,846,580	753,674
	Provision against off-balance sheet obligations		46,189	46,581
	Security deposits against lease		499,089	491,366
	Lease liability against right of use assets		8,035,048	8,295,864
	Others		13,348,016	11,265,893
			82,900,828	94,295,738

20.1 Supreme Court of Pakistan vide its order dated November 10, 2016 has held that the amendments made in the law introduced by the Federal Government for the levy of Workers Welfare Fund were not lawful. The Federal Board of Revenue has filed review petitions against this order which are currently pending.

Legal advice obtained on the matter indicates that consequent to filing of these review petitions the judgment may not currently be treated as conclusive. Accordingly, the Bank maintained its provision in respect of WWF.

21. SHARE CAPITAL

21.1 Authorized Capital

2020	2019		2020	2019
(Number of shares)			(Rupees in '000)	
1,500,000,000	1,500,000,000	Ordinary shares of Rs. 10 each	15,000,000	15,000,000

21.2 Issued, subscribed and paid up

2020	2019		2020	2019
(Number of shares)			(Rupees in '000)	
		<u>Ordinary shares</u>		
197,253,795	197,253,795	Fully paid in cash	1,972,538	1,972,538
915,776,953	915,776,953	Issued as bonus shares	9,157,769	9,157,769
72,029,258	72,029,258	Issued for consideration other than cash	720,293	720,293
1,185,060,006	1,185,060,006		11,850,600	11,850,600

21.3 The movement in the issued, subscribed and paid-up capital during the year is as follows:

2020	2019		2020	2019
(Number of shares)			(Rupees in '000)	
1,185,060,006	1,185,060,006	Opening balance at January 1	11,850,600	11,850,600
1,185,060,006	1,185,060,006	Closing balance at December 31	11,850,600	11,850,600

21.4 Number of shares held by the associated undertakings as at December 31, are as follows:

	2020	2019
	(Number of shares)	
Adamjee Insurance Company Limited	47,827,287	47,827,287
Nishat Mills Limited	88,015,291	88,015,291
Siddiqsons Limited	14,276,462	14,276,462
Nishat (Aziz Avenue) Hotels and Properties Limited	141,950	29,000
Nishat Real Estates Development Company (Private) Limited	54,500	–
	150,315,490	150,148,040

	Note	2020	2019
		(Rupees in '000)	
**22. RESERVES**			
Share premium		23,751,114	23,751,114
Non- distributable capital reserve - gain on bargain purchase option	22.1	908,317	908,317
Exchange translation reserve		2,876,483	2,675,131
Statutory reserve	22.2	34,560,421	31,656,691
General reserve		18,600,000	18,600,000
		80,696,335	77,591,253

22.1 Under IFRS-3 a bargain purchase represents an economic gain which should be immediately recognized by the acquirer as income. However, the amount of bargain purchase gain was not taken to the profit and loss account as the SBP, through its letter BPRD(R&PD)/2017/14330 dated June 13, 2017 recommended that the amount of gain may be routed directly into equity as a Non-distributable Capital Reserve (NCR). The NCR may become available for distribution through a stock dividend only with prior approval of the SBP. The Bank, before distribution of the gain as a stock dividend, may adjust any subsequent provisions/deficit, assessed by the Bank or recommended by the Banking Inspection Department of SBP, in the acquired assets and liabilities of NIB Bank Limited against the NCR.

22.2 Statutory reserve represents amount set aside as per the requirements of section 21 of the Banking Companies Ordinance, 1962.

Notes To The Unconsolidated Financial Statements

For the year ended December 31, 2020

	Note	2020	2019
		(Rupees in '000)	
**23. SURPLUS ON REVALUATION OF ASSETS**			
Surplus on revaluation of			
- Available for sale securities	10.1	12,676,354	6,488,841
- Fixed Assets	23.1	20,211,952	20,383,765
- Non-banking assets acquired in satisfaction of claims	23.2	854,231	677,660
		33,742,537	27,550,266
Deferred tax on surplus on revaluation of:			
- Available for sale securities	19	4,436,721	2,271,094
- Fixed Assets	23.1	1,286,416	1,346,550
- Non-banking assets acquired in satisfaction of claims	23.2	298,982	237,181
		6,022,119	3,854,825
		27,720,418	23,695,441

23.1 Surplus on revaluation of fixed assets

	2020	2019
Surplus on revaluation of fixed assets as at January 1	20,383,765	13,185,366
Recognised during the year	–	7,290,966
Realised on disposal during the year - net of deferred tax	(22,544)	(10,560)
Transferred to unappropriated profit in respect of incremental depreciation charged during the year - net of deferred tax	(89,135)	(53,248)
Related deferred tax liability on incremental depreciation charged during the year	(47,995)	(28,672)
Related deferred tax liability on surplus realised on disposal	(12,139)	(87)
Surplus on revaluation of fixed assets as at December 31	20,211,952	20,383,765
Less: Related deferred tax liability on:		
- revaluation as at January 1	1,346,550	992,684
- recognised during the year	–	382,625
- surplus realised on disposal during the year	(12,139)	(87)
- incremental depreciation charged during the year	(47,995)	(28,672)
	1,286,416	1,346,550
	18,925,536	19,037,215

23.2 Surplus on revaluation of non-banking assets acquired in satisfaction of claims

	2020	2019
Surplus on revaluation as at January 1	677,660	480,871
Recognised during the year	183,915	255,040
Realised on disposal during the year - net of deferred tax	(4,774)	(37,863)
Related deferred tax liability on surplus realised on disposal	(2,570)	(20,388)
Surplus on revaluation as at December 31	854,231	677,660
Less: Related deferred tax liability on:		
- revaluation as at January 1	237,181	168,305
- revaluation recognised during the year	64,371	89,264
- surplus realised on disposal during the year	(2,570)	(20,388)
	298,982	237,181
	555,249	440,479

Notes To The Unconsolidated Financial Statements

For the year ended December 31, 2020

		Note	2020	2019
			(Rupees in '000)	
**24.**	**CONTINGENCIES AND COMMITMENTS**			
	- Guarantees	24.1	178,571,960	173,535,128
	- Commitments	24.2	507,506,107	649,690,990
	- Other contingent liabilities	24.3	27,960,316	27,920,652
			714,038,383	851,146,770
**24.1**	**Guarantees:**			
	Financial guarantees		149,925,920	141,181,839
	Performance guarantees		25,900,273	30,401,373
	Other guarantees		2,745,767	1,951,916
			178,571,960	173,535,128
**24.2**	**Commitments:**			
	Documentary credits and short-term trade-related transactions			
	- letters of credit		172,617,563	145,217,983
	Commitments in respect of:			
	- forward foreign exchange contracts	24.2.1	318,420,575	405,615,318
	- forward government securities transactions	24.2.2	11,089,775	87,696,638
	- derivatives	24.2.3	4,471,383	10,244,806
	Commitments for acquisition of:			
	- operating fixed assets		710,570	859,953
	- intangible assets		196,241	56,292
			507,506,107	649,690,990
**24.2.1**	**Commitments in respect of forward foreign exchange contracts**			
	Purchase		168,432,858	217,809,539
	Sale		149,987,717	187,805,779
			318,420,575	405,615,318
**24.2.2**	**Commitments in respect of government securities transactions**			
	Purchase		11,089,775	82,284,304
	Sale		–	5,412,334
			11,089,775	87,696,638

Notes To The Unconsolidated Financial Statements
For the year ended December 31, 2020

		2020	2019
		(Rupees in '000)	
**24.2.3**	**Commitments in respect of derivatives**		
	FX options (notional)		
	Purchase	182,800	431,449
	Sale	182,800	431,449
		365,600	862,898
	Cross Currency Swaps (notional)		
	Purchase	1,975,311	4,428,663
	Sale	2,130,472	4,636,745
		4,105,783	9,065,408
	Interest Rate Swaps (notional)		
	Purchase	–	316,500
	Sale	–	–
		–	316,500
		4,471,383	10,244,806

24.2.4 The Bank makes commitments to extend credit in the normal course of its business but these being revocable commitments do not attract any significant penalty or expense if the facility is unilaterally withdrawn.

		Note	2020	2019
			(Rupees in '000)	
**24.3**	**Other contingent liabilities**			
	Claims against the Bank not acknowledged as debts	24.3.1	27,960,316	27,920,652

24.3.1 These represent certain claims by third parties against the Bank, which are being contested in the Courts of law. The management is of the view that these relate to the normal course of business and the possibility of an outflow of economic resources is remote.

24.4 For assessment year 1988-89 through tax year 2019, the tax department disputed Bank's treatment on certain issues, where the Bank's appeals are pending at various appellate forums, entailing an additional tax liability of Rs. 6,033 million (2019: Rs. 1,487 million). Such issues inter alia principally include disallowance of expenses for non deduction of withholding tax and non availability of underlying records, provision for non performing loans, attribution of expenses to heads of income other than income from business and disallowance of credit for taxes paid in advance / deducted at source.

The Bank has filed appeals which are pending at various appellate forums. In addition, certain decisions made in favour of the Bank are being contested by the department at higher forums. No provision has been made in the financial statements regarding the aforesaid additional tax demand and already issued favourable decisions where the department is in appeal, as the management is of the view that the issues will be decided in the Bank's favour as and when these are taken up by the Appellate Authorities.

24.5 Amortisation of goodwill and other intangibles amounting to Rs 28.08 billion of Ex. NIB

Issue of goodwill and other related assets amortization for few years has been assessed in Bank's favour at appellate forums during the year, however, the tax department has filed appeal against these decisions. The management has not recorded any tax benefit because the issue has not attained finality.

25. DERIVATIVE INSTRUMENTS

25.1 Product Analysis

	2020					
	Cross Currency Swaps		Interest Rate Swaps		Fx Option	
	Notional principal	Mark to market gain/loss	Notional principal	Mark to market gain/loss	Notional principal	Mark to market gain/loss
	(Rupees in '000)					
**Counterparties**						
**With Banks for**						
Hedging	1,975,311	512,508	–	–	182,800	4,525
Market Making	–	–	–	–	–	–
**With other entities for**						
Hedging	–	–	–	–	–	–
Market Making	2,130,472	(508,818)	–	–	182,800	(4,525)
**Total**						
Hedging	1,975,311	512,508	–	–	182,800	4,525
Market Making	2,130,472	(508,818)	–	–	182,800	(4,525)

	2019					
	Cross Currency Swaps		Interest Rate Swaps		Fx Option	
	Notional principal	Mark to market gain/loss	Notional principal	Mark to market gain/loss	Notional principal	Mark to market gain/loss
	(Rupees in '000)					
**Counterparties**						
**With Banks for**						
Hedging	4,428,663	1,218,634	316,500	11,510	431,449	2,683
Market Making	–	–	–	–	–	–
**With other entities for**						
Hedging	–	–	–	–	–	–
Market Making	4,636,745	(1,226,433)	–	–	431,449	(2,683)
**Total**						
Hedging	4,428,663	1,218,634	316,500	11,510	431,449	2,683
Market Making	4,636,745	(1,226,433)	–	–	431,449	(2,683)

Notes To The Unconsolidated Financial Statements

For the year ended December 31, 2020

25.2 Maturity Analysis

	2020				
	No. of contracts	Notional principal	Mark to Market		
			Negative	Positive	Net
	(Rupees in '000)				
**Remaining Maturity**					
Upto 1 month	8	924,787	(54,638)	54,840	202
1 to 3 months	4	120,859	(1,545)	1,545	–
3 to 6 months	4	421,010	(71,467)	71,915	448
6 month to 1 Year	3	329,626	(23,640)	18,177	(5,463)
1 to 2 Year	4	1,094,545	(220,328)	226,567	6,239
2 to 3 Years	0	–	–	–	–
3 to 5 Years	2	1,580,556	(141,725)	143,989	2,264
Total	25	4,471,383	(513,343)	517,033	3,690

	2019				
	No. of contracts	Notional principal	Mark to Market		
			Negative	Positive	Net
	(Rupees in '000)				
**Remaining Maturity**					
Upto 1 month	14	409,597	(518)	518	–
1 to 3 months	32	1,076,111	(154,368)	154,707	339
3 to 6 months	1	52,745	(25,888)	–	(25,888)
6 month to 1 Year	7	1,847,480	(298,299)	299,689	1,390
1 to 2 Year	9	3,199,475	(312,482)	323,222	10,740
2 to 3 Years	4	1,714,098	(340,100)	353,553	13,453
3 to 5 Years	2	1,945,300	(101,151)	104,828	3,677
Total	69	10,244,806	(1,232,806)	1,236,517	3,711

25.3 Disclosure relating to qualitative and quantitative information on exchange traded derivatives are disclosed in note 45.5.

25.4 Risk management related to derivatives is discussed in note 45.5.

	2020	2019
	(Rupees in '000)	
**26. MARK-UP/RETURN/INTEREST EARNED**		
Loans and advances	42,879,345	57,329,597
Investments	91,983,471	75,481,025
Lendings to financial institutions	998,236	4,982,470
Balances with banks	214,653	498,804
	136,075,705	138,291,896

Notes To The Unconsolidated Financial Statements
For the year ended December 31, 2020

	Note	2020	2019
		(Rupees in '000)	
**27. MARK-UP/RETURN/INTEREST EXPENSED**			
Deposits		55,095,363	65,343,677
Borrowings		6,152,383	8,977,118
Subordinated debt		–	213,604
Cost of foreign currency swaps against foreign currency deposits / borrowings		2,386,644	3,009,184
Unwinding cost of liability against right-of-use assets		1,106,824	1,132,099
		64,741,214	78,675,682
**28. FEE & COMMISSION INCOME**			
Branch banking customer fees		1,914,132	1,810,293
Consumer finance related fees		420,289	331,007
Card related fees (debit and credit cards)		3,130,966	3,065,534
Credit related fees		47,282	183,688
Investment banking fee		144,137	168,748
Commission on trade		1,277,171	1,406,051
Commission on guarantees		562,447	584,369
Commission on cash management		614,297	602,605
Commission on remittances including home remittances		1,010,735	1,172,359
Commission on utility bills		79,791	106,320
Commission income - Bancassurance		1,329,986	1,432,878
Rent on lockers		216,420	236,616
Commission on investments services		30,131	56,360
Other Commission		158,541	131,222
		10,936,325	11,288,050
**29. GAIN ON SECURITIES, NET**			
Realised	29.1	3,332,256	837,022
Unrealised - held for trading	10.1	(224)	(4,176)
		3,332,032	832,846
**29.1** Realised gain on:			
Federal Government Securities		3,438,217	95,699
Subsidiary		72,194	(7,512)
Non Government Debt Securities		(76,169)	–
Shares & units		(101,986)	748,835
		3,332,256	837,022
**30. OTHER INCOME**			
Rent on property		67,310	69,427
Gain on sale of fixed assets-net		72,601	90,158
Loss on termination of lease liability against right of use assets		(15,637)	–
Gain on sale of non banking assets - net	14.1.2	3,976	111,948
		128,250	271,533

Notes To The Unconsolidated Financial Statements

For the year ended December 31, 2020

	Note	2020	2019
		(Rupees in '000)	
**31. OPERATING EXPENSES**			
**Total compensation expense**	31.1	15,805,510	14,585,179
**Property expense**			
Rent and taxes		160,642	226,910
Insurance		23,197	21,200
Utilities cost		1,218,168	1,203,064
Fuel expense generators		334,634	534,428
Security (including guards)		1,240,298	1,382,080
Repair and maintenance (including janitorial charges)		653,082	811,653
Depreciation on right-of-use assets	12.3	1,216,784	1,161,795
Depreciation	12.2	641,564	504,153
		5,488,369	5,845,283
**Information technology expenses**			
Software maintenance		1,148,436	1,188,463
Hardware maintenance		212,381	298,898
Depreciation	12.2	547,930	615,598
Amortisation	13	318,024	301,022
Network charges		598,380	601,405
Insurance		3,850	3,129
		2,829,001	3,008,515
**Other operating expenses**			
Directors' fees and allowances		50,060	57,479
Legal and professional charges		268,451	350,295
Outsourced services costs	37.1	691,787	690,391
Travelling and conveyance		260,676	321,448
NIFT clearing charges		166,175	152,009
Depreciation	12.2	815,567	796,895
Depreciation on non-banking assets acquired in satisfaction of claims	14.1.1	30,049	45,456
Training and development		36,791	57,230
Postage and courier charges		233,891	303,262
Communication		326,441	373,368
Stationery and printing		563,090	639,343
Marketing, advertisement & publicity		695,291	625,463
Donations	31.2	112,596	100
Auditors' remuneration	31.3	29,720	29,944
Cash transportation charges		709,262	799,226
Repair and maintenance		401,826	415,645
Subscription		20,637	20,184
Entertainment		183,703	231,549
Remittance charges		211,631	197,223
Brokerage expenses		36,124	32,561
Card related expenses		829,055	1,182,346
CNIC verification charges		128,614	206,509
Insurance		1,483,323	1,440,525
Others		238,142	263,490
		8,522,902	9,231,941
		32,645,782	32,670,918

Total cost for the year included in other operating expenses relating to outsourced activities is Rs 275.517 million (2019: Rs 307.993 million) which pertains to payments made to companies incorporated in Pakistan. This includes payments other than outsourced services costs, which are disclosed above. Total Cost of outsourced activities for the year given to related parties is Rs 275.517 million (2019: Rs 258.190 million). Outsourcing shall have the same meaning as specified in BPRD Circular No. 06 of 2019.

Notes To The Unconsolidated Financial Statements

For the year ended December 31, 2020

The Bank has material outsourcing arrangement with “Euronet Pakistan Private Limited” (a related party) for hosting of debit cards, prepaid cards, POS terminals and ATMs switch system. The annual cost of outsourcing is Rs 275.517 million (2019: 258.190 million).

		Note	2020	2019
			(Rupees in '000)	
31.1	**Total compensation expense**			
	Fees and allowances		470,085	497,837
	Managerial remuneration			
	i) Fixed		11,514,132	10,928,504
	ii) Variable - cash bonus / awards		2,430,988	2,030,589
	Charge / (reversal) for defined benefit plan		74,827	(101,509)
	Contribution to defined contribution plan		394,598	366,744
	Commission		348,633	330,812
	Staff group insurance		386,388	312,868
	Rent and house maintenance		58,094	56,205
	Medical		35,135	35,516
	Conveyance		88,130	89,913
	**Sub-total**		15,801,010	14,547,479
	Sign-on bonus	31.1.1	3,700	100
	Severance allowance	31.1.2	800	37,600
	**Grand Total**		15,805,510	14,585,179

31.1.1 During the year sign on bonus was paid to 4 employees (2019: 1).

31.1.2 Severance allowance pertains to 1 employee (2019: 6).

31.2 Detail of donations made during the year is as follows:

	2020	2019
	(Rupees in '000)	
Murshid Hospital & Health Care Centre	–	100
Saleem Memorial Trust Hospital	95,000	–
Specialized Healthcare and Medical Education		
Department, Government of Punjab - (COVID 19 relief)	9,996	–
Jahandad Society For Community Development	5,000	–
District Administration Lahore - (COVID 19 relief)	2,600	–
	112,596	100

31.2.1 None of the directors, executives or their spouses had any interest in the donees, except for Mr. Yahya Saleem who is a director in the Saleem Memorial Trust Hospital.

		2020	2019
		(Rupees in '000)	
31.3	**Auditors' remuneration**		
	Audit fee	16,500	15,628
	Fee for audit of foreign branches	10,272	9,905
	Special certifications and sundry advisory services	473	348
	Sales tax	825	2,500
	Out-of-pocket expenses	1,650	1,563
		29,720	29,944

Notes To The Unconsolidated Financial Statements

For the year ended December 31, 2020

		Note	2020	2019
			(Rupees in '000)	
**32.**	**OTHER CHARGES**			
	Penalties of State Bank of Pakistan		191,766	46,069
	VAT & National Building tax & Crop Insurance Levy		92,502	179,643
	Education cess		13,129	10,645
			297,397	236,357
**33.**	**PROVISIONS / (REVERSALS) & WRITE OFFS - NET**			
	(Reversals) / provisions against balance with Banks		(3,569)	(3,111)
	(Reversals) / provisions for diminution in value of investments	10.3.1	(49,922)	2,823,532
	(Reversals) / provisions against loans & advances	11.4	7,521,701	(157,616)
	(Reversals) / provisions against off balance sheet items	20	(448)	9,025
	Reversals against other assets	14.2.1	(23,648)	(23,436)
	Recovery of written off / charged off bad debts		(130,948)	(164,860)
			7,313,166	2,483,534
**34.**	**TAXATION**			
	Current		20,115,480	15,355,566
	Prior years		–	450,438
	Deferred	19	(903,826)	319,469
			19,211,654	16,125,473

34.1 Relationship between tax expense and accounting profit

	2020	2019
Accounting profit for the year	48,248,955	40,102,312
Tax rate	35%	35%
Tax on income	16,887,134	14,035,809
Tax effect of permanent differences	67,118	16,124
Tax effect of prior years reversals	–	450,438
Others	2,257,402	1,623,102
Tax charge for the year	19,211,654	16,125,473

		2020	2019
		(Rupees in '000)	
**35.**	**BASIC AND DILUTED EARNINGS PER SHARE**		
	Profit for the year after tax	29,037,301	23,976,839
		(Number)	
	Weighted average number of ordinary shares	1,185,060,006	1,185,060,006
		(Rupees)	
	Basic and diluted earnings per share	24.50	20.23

	Note	2020	2019
		(Rupees in '000)	
**36. CASH AND CASH EQUIVALENTS**			
Cash and balances with treasury banks	7	122,180,839	132,704,797
Balances with other banks	8	24,030,328	12,542,239
Overdrawn nostro accounts	17	(397,313)	(1,349,447)
		145,813,854	143,897,589

36.1 Reconciliation of movement of liabilities to cash flows arising from financing activities

	2020						2019					
	Liabilities		Equity				Liabilities		Equity			
	Sub-ordinated loan	Other liabilities	Share capital	Reserves	Unappropriated profit	Total	Sub-ordinated loan	Other liabilities	Share capital	Reserves	Unappropriated profit	Total
	(Rupees in '000)											
**Balance as at January 01,**	–	94,295,738	11,850,600	77,591,253	55,777,489	239,515,080	3,891,019	62,673,355	11,850,600	74,147,981	53,532,044	206,094,999
Changes from Financing cash flows												
Redemption of Subordinated loan	–	–	–	–	–	–	(3,891,019)	–	–	–	–	(3,891,019)
Payment of lease liability against right-of-use-assets	–	(1,682,647)	–	–	–	(1,682,647)	–	(1,427,643)	–	–	–	(1,427,643)
Dividend paid	–	–	–	–	(11,750,637)	(11,750,637)	–	–	–	–	(18,838,387)	(18,838,387)
Total changes from financing cash flows	–	(1,682,647)	–	–	(11,750,637)	(13,433,284)	(3,891,019)	(1,427,643)	–	–	(18,838,387)	(24,157,049)
**Liability related**												
Changes in Other liabilities												
- Cash based	–	(18,498,355)	–	–	–	(18,498,355)	–	13,550,906	–	–	–	13,550,906
- Dividend payable	–	99,963	–	–	(99,963)	–	–	122,573	–	–	(122,573)	–
- Non cash based	–	8,686,129	–	–	–	8,686,129	–	19,376,547	–	–	–	19,376,547
Total liability related other changes	–	(9,712,263)	–	–	(99,963)	(9,812,226)	–	33,050,026	–	–	(122,573)	32,927,453
Total equity related other changes	–	–	–	3,105,082	25,907,713	29,012,795	–	–	–	3,443,272	21,206,405	24,649,677
Balance as at December 31	–	82,900,828	11,850,600	80,696,335	69,834,602	245,282,365	–	94,295,738	11,850,600	77,591,253	55,777,489	239,515,080

Notes To The Unconsolidated Financial Statements

For the year ended December 31, 2020

	2020	2019
	(Number)	
**37. STAFF STRENGTH**		
Permanent	13,536	13,480
On Bank contract	107	116
Bank's own staff strength at end of the year	13,643	13,596

37.1 In addition to the above,118 (2019: 157) employees of outsourcing services companies were assigned to the Bank as at the end of the year to perform services other than guarding, tea and janitorial services. Outsourced staff includes 111 (2019: 147) working domestically and 7 (2019: 10) working abroad.

38. DEFINED BENEFIT PLAN

38.1 General description

The Bank operates the following retirement benefits for its employees:

- Pension fund - funded
- Benevolent scheme - unfunded
- Post retirement medical benefits - unfunded
- Employees compensated absence - unfunded

The plan assets and defined benefit obligations are based in Pakistan.

38.2 Number of Employees under the scheme

The number of employees covered under the following defined benefit schemes are:

	2020	2019
	(Number)	
- Pension fund - funded	5,410	6,294
- Benevolent scheme - unfunded	1,108	1,692
- Post retirement medical benefits - unfunded	13,386	13,332
- Employees compensated absence - unfunded	13,386	13,332

38.3 Principal actuarial assumptions

The latest actuarial valuations of the pension fund, employees' contributory benevolent scheme, post retirement medical benefits and employee's compensated absences were carried out at December 31, 2020. The principal actuarial assumptions used are as follows:

	Approved Pension fund		Employees' contributory benevolent scheme		Post retirement medical benefits		Employees compensated absences	
	2020	2019	2020	2019	2020	2019	2020	2019
	(%)							
Discount rate	9.75	11.25	9.75	11.25	9.75	11.25	9.75	11.25
Expected rate of return on plan assets	9.75	11.25	–	–	–	–	–	–
Expected rate of salary increase	7.75	9.25	7.75	9.25	–	–	7.75	9.25
Expected rate of increase in pension	0-5	0-5	–	–	–	–	–	–
Expected rate of increase in medical benefit	–	–	–	–	7.75	9.25	–	–

Notes To The Unconsolidated Financial Statements

For the year ended December 31, 2020

38.4 Reconciliation of (receivable from) / payable to defined benefit plans

	Approved Pension fund		Employees' contributory benevolent scheme		Post retirement medical benefits		Employees compensated absences	
	2020	2019	2020	2019	2020	2019	2020	2019
	(Rupees in '000)							
Present value of obligations	5,097,744	5,182,991	222,084	221,193	2,004,122	1,921,348	919,407	939,495
Fair value of plan assets	(8,467,923)	(8,788,112)	–	–	–			
(Receivable) / payable	(3,370,179)	(3,605,121)	222,084	221,193	2,004,122	1,921,348	919,407	939,495

38.5 Movement in defined benefit obligations

	Note	Approved Pension fund		Employees' contributory benevolent scheme		Post retirement medical benefits		Employees compensated absences	
		2020	2019	2020	2019	2020	2019	2020	2019
		(Rupees in '000)							
Obligations at the beginning of the year		5,182,991	4,690,587	221,193	214,252	1,921,348	1,730,409	939,495	1,028,129
Current service cost	38.8.1	64,350	60,879	21,742	22,341	53,018	43,653	24,428	24,740
Interest cost		560,303	592,985	22,931	26,035	208,381	219,145	98,224	128,046
Benefits paid		(405,032)	(430,453)	(34,721)	(35,528)	(138,149)	(152,964)	(132,782)	(123,486)
Re-measurement loss / (gain)	38.8.2	(304,868)	268,993	(9,061)	(5,907)	(40,476)	81,105	(9,958)	(117,934)
Obligations at end of the year		5,097,744	5,182,991	222,084	221,193	2,004,122	1,921,348	919,407	939,495

38.6 Movement in fair value of plan assets

	Note	Approved Pension fund		Employees' contributory benevolent scheme		Post retirement medical benefits		Employees compensated absences	
		2020	2019	2020	2019	2020	2019	2020	2019
		(Rupees in '000)							
Fair value at the beginning of the year		8,788,112	8,505,757	–	–	–	–	–	–
Interest income on plan assets		965,880	1,098,495	–	–	–	–	–	–
Benefits paid		(405,032)	(430,453)	–	–	–	–	–	–
Re-measurement loss	38.8.2	(881,037)	(385,687)	–	–	–	–	–	–
Fair value at end of the year		8,467,923	8,788,112	–	–	–	–	–	–

38.7 Movement in (receivable) / payable under defined benefit schemes

	Note	Approved Pension fund		Employees' contributory benevolent scheme		Post retirement medical benefits		Employees compensated absences	
		2020	2019	2020	2019	2020	2019	2020	2019
		(Rupees in '000)							
Opening balance		(3,605,121)	(3,815,170)	221,193	214,252	1,921,348	1,730,409	939,495	1,028,129
Charge / (reversal) for the year	38.8.1	(341,227)	(444,631)	41,961	45,472	261,399	262,798	112,694	34,852
Employees' contribution		–	–	2,712	2,904	–	–	–	–
Re-measurement loss / (gain) recognised in OCI during the year	38.8.2	576,169	654,680	(9,061)	(5,907)	(40,476)	81,105	–	–
Benefits paid by the Bank		–	–	(34,721)	(35,528)	(138,149)	(152,964)	(132,782)	(123,486)
Closing balance		(3,370,179)	(3,605,121)	222,084	221,193	2,004,122	1,921,348	919,407	939,495

Notes To The Unconsolidated Financial Statements

For the year ended December 31, 2020

38.8 Charge for defined benefit plans

38.8.1 Cost recognised in profit and loss

	Approved Pension fund		Employees' contributory benevolent scheme		Post retirement medical benefits		Employees compensated absences	
	2020	2019	2020	2019	2020	2019	2020	2019
	(Rupees in '000)							
Current service cost	64,350	60,879	21,742	22,341	53,018	43,653	24,428	24,740
Net interest on defined benefit asset / liability	(405,577)	(505,510)	22,931	26,035	208,381	219,145	98,224	128,046
Employees' contribution	–	–	(2,712)	(2,904)	–	–	–	–
Actuarial (gain)	–	–	–	–	–	–	(9,958)	(117,934)
	(341,227)	(444,631)	41,961	45,472	261,399	262,798	112,694	34,852

38.8.2 Re-measurements recognised in OCI during the year

	Approved Pension fund		Employees' contributory benevolent scheme		Post retirement medical benefits		Employees compensated absences	
	2020	2019	2020	2019	2020	2019	2020	2019
	(Rupees in '000)							
Loss / (gain) on obligation - financial assumptions	(304,868)	268,993	(9,061)	(5,907)	(40,476)	81,105	–	–
Actual return on plan assets over expected interest income	881,037	385,687	–	–	–	–	–	–
Re-measurement loss / (gain) recognised in OCI	576,169	654,680	(9,061)	(5,907)	(40,476)	81,105	–	–

38.9 Components of plan assets

	Approved Pension fund		Employees' contributory benevolent scheme		Post retirement medical benefits		Employees compensated absences	
	2020	2019	2020	2019	2020	2019	2020	2019
	(Rupees in '000)							
Cash and cash equivalents - net	24,451	107,344	–	–	–	–	–	–
Shares	8,129,575	8,387,473	–	–	–	–	–	–
Open ended mutual funds units	313,897	293,295	–	–	–	–	–	–
	8,467,923	8,788,112	–	–	–	–	–	–

38.9.1 Significant risk associated with the plan assets

The Fund's investments in equity securities and units of mutual funds are subject to price risk. These risks are regularly monitored by Trustees of the employee funds.

Notes To The Unconsolidated Financial Statements

For the year ended December 31, 2020

38.10 Sensitivity analysis

Sensitivity analysis has been performed by varying one assumption keeping all other assumptions constant and calculating the impact on the present value of the defined benefit obligations under the various employee benefit schemes. The increase / (decrease) in the present value of defined benefit obligations as a result of change in each assumption is summarized below:

	Approved Pension fund	Employees' contributory benevolent scheme	Post retirement medical benefits	Employees compensated absences
	(Rupees in '000)			
1% increase in discount rate	(337,220)	(16,983)	(222,612)	(56,543)
1% decrease in discount rate	384,510	19,474	275,965	62,843
1 % increase in expected rate of salary increase	78,500	–	–	63,453
1 % decrease in expected rate of salary increase	(73,370)	–	–	(58,050)
1% increase in expected rate of pension increase	282,140	–	–	–
1% decrease in expected rate of pension increase	(252,260)	–	–	–
1% increase in expected rate of medical benefit increase	–	–	144,805	–
1% decrease in expected rate of medical benefit increase	–	–	(123,190)	–

38.11 Expected contributions to be paid to the funds in the next financial year

No contributions are being made to pension fund due to surplus of fair value of plan's assets over present value of defined obligation. No contribution to the pension fund is expected in the next year.

38.12 Expected charge / (reversal) for the next financial year

Based on actuarial advice, management estimates that the charge / (reversal) in respect of defined benefit plans for the year ending December 31, 2021 would be as follows:

	Approved Pension fund	Employees' contributory benevolent scheme	Post retirement medical benefits	Employees compensated absences
	(Rupees in '000)			
Expected charge / (reversal) for the next financial year	(265,940)	38,232	257,380	114,262

38.13 Maturity profile

The weighted average duration of the obligation (in years)	8.34	8.34	8.34	8.34

38.14 Funding Policy

The Bank endeavours to ensure that liabilities under the various employee benefit schemes are covered by the Fund on any valuation date having regards to the various actuarial assumptions such as projected future salary increase, expected future contributions to the fund, projected increase in liability associated with future service and the projected investment income of the Fund.

Notes To The Unconsolidated Financial Statements
For the year ended December 31, 2020

38.15 The defined benefit plans may expose the bank to actuarial risks such as longevity risk, investment risk, salary increase risk and withdrawal rate risk as described below;

**Investment risk**	The risk arises when the actual performance of the investments is lower than expectation and thus creating a shortfall in the funding objectives.
**Longevity risk**	The risk arises when the actual lifetime of retirees is longer than expectation. This risk is measured at the plan level over the entire retiree population.
**Salary increase risk**	The most common type of retirement benefit is one where the benefit is linked with final salary. The risk arises when the actual increases are higher than expectation and impacts the liability accordingly.
**Withdrawal rate**	The risk of actual withdrawals varying with the actuarial assumptions can impose a risk to the benefit obligation. The movement of the liability can go either way.

39. DEFINED CONTRIBUTION PLAN

The Bank operates an approved contributory provident fund for 11,458 (2019: 11,056) employees where contributions are made by the Bank at 8.33% (2019: 8.33%) and employees ranging from 8.33% to 15% per annum (2019: 8.33% to 15% per annum) of the basic salary.

The Bank also operates an approved non-contributory provident fund for 687 (2019: 745) employees who have opted for the new scheme, where contributions are made by the employees ranging from 8.33% to 15% per annum (2019: 8.33% to 15% per annum) of the basic salary.

40. COMPENSATION OF DIRECTORS AND KEY MANAGEMENT PERSONNEL

40.1. Total compensation expense

		2020					
		Directors				Key	Other material
	Note	Chairman	Executive (other than CEO	Non executive	President/ CEO	management personnel	risk takers/ controllers
		(Rupees in '000)					
Fee and allowances	40.2	7,210	–	42,850	–	112	10,111
Managerial Remuneration							
i) Fixed		–	–	–	72,362	301,222	684,109
ii) Cash Bonus / Awards	40.1.1	–	–	–	50,000	144,349	218,954
Contribution to defined contribution plan		–	–	–	–	8,317	25,756
Rent & house maintenance		–	–	–	240	15,736	10,948
Medical		–	–	–	1,828	1,281	5,694
Severance allowance		–	–	–	–	800	–
Overseas allowance		–	–	–	–	37,559	–
Security		–	–	–	804	–	–
Commission					–	300	43,407
Club membership		–	–	–	1,872	–	300
Total		7,210	–	42,850	127,106	509,676	999,279
Number of Persons		1	–	11	1	23	124

Notes To The Unconsolidated Financial Statements

For the year ended December 31, 2020

	2019					
	Directors				Key	Other material
	Chairman	Executive (other than CEO	Non executive	President/ CEO	management personnel	risk takers/ controllers
	(Rupees in '000)					
Fee and allowances	25,000	–	32,479	–	672	4,458
Managerial Remuneration						
i) Fixed	–	–	–	63,046	293,751	443,330
ii) Cash Bonus / Awards	–	–	–	40,000	137,134	135,427
Contribution to defined contribution plan	–	–	–	2,484	8,495	16,232
Rent & house maintenance	–	–	–	240	15,350	7,894
Medical	–	–	–	1,071	1,262	4,319
Severance allowance	–	–	–	–	18,500	8,800
Overseas allowance	–	–	–	–	38,947	7,416
Security	–	–	–	1,189	–	–
Commission	–	–	–	–	57	14,471
Club membership	–	–	–	70	–	2,160
Total	25,000	–	32,479	108,100	514,168	644,507
Number of Persons	1	–	12	1	23	75

40.1.1 During the year 2020, Rs 20.6 million bonus has been deferred.

40.2 Remuneration paid to Directors for participation in Board and Committee meetings

	2020										
		For Board Committee									
	Board meeting	Board's Audit Committee	BS & DC	RM & PRC	HR & RC	ITC	PP & CA	CR & MC	Wo & WC	*As Board Chairman	Total
	(Rupees in '000)										
Mian Mohammad Mansha	3,840	–	300	–	300	–	400	–	–	2,370	7,210
Mr. S. M. Muneer	1,700	–	100	–	–	–	–	100	–	–	1,900
Mr. Tariq Rafi	1,700	–	–	–	–	–	–	–	200	–	1,900
Mian Umer Mansha	1,700	500	400	500	–	500	400	–	300	–	4,300
Mrs. Iqraa Hassan Mansha	1,200	–	–	–	200	–	100	–	–	–	1,500
Mr. Muhammad Ali Zeb	1,700	500	–	500	300	–	300	400	300	–	4,000
Mr. Mohd Suhail Amar Suresh	4,460	–	400	500	–	500	–	–	–	–	5,860
Mr. Yahya Saleem	4,466	–	–	–	300	200	–	–	–	–	4,966
Mr. Salman Khalid Butt	4,586	–	400	500	–	500	–	400	–	–	6,386
Mr. Masood Ahmed Puri	4,466	–	400	–	–	–	–	–	–	–	4,866
Mr. Shahzad Hussain	1,700	500	–	–	–	–	–	–	–	–	2,200
Mr. Shariffuddin Bin Khalid	4,472	500	–	–	–	–	–	–	–	–	4,972
	35,990	2,000	2,000	2,000	1,100	1,700	1,200	900	800	2,370	50,060

Notes To The Unconsolidated Financial Statements

For the year ended December 31, 2020

	2019										
		For Board Committee									
	Board meeting	Board's Audit Committee	BS & DC	RM & PRC	HR & RC	ITC	PP & CA	CR & MC	Wo & WC	*As Board Chairman	Total
	(Rupees in '000)										
Mian Mohammad Mansha	–	–	–	–	–	–	–	–	–	25,000	25,000
Mr. S. M. Muneer	130	–	30	–	–	–	–	30	–	–	190
Mr. Tariq Rafi	430	–	–	–	–	–	–	–	–	–	430
Mian Umer Mansha	300	330	330	300	–	330	330	–	–	–	1,920
Mrs. Iqraa Hassan Mansha	230	–	–	–	–	–	–	–	–	–	230
Mr. Muhammad Ali Zeb	330	330	–	230	330	–	300	330	–	–	1,850
Mr. Mohd Suhail Amar Suresh	4,620	–	230	230	–	230	–	–	–	–	5,310
Mr. Yahya Saleem	4,710	–	–	–	130	200	–	–	–	–	5,040
Mr. Salman Khalid Butt	5,814	–	330	230	–	330	–	300	–	–	7,004
Mr. Masood Ahmed Puri	3,503	–	200	–	–	–	–	–	–	–	3,703
Mr. Shahzad Hussain	300	300	–	–	–	–	–	–	–	–	600
Mr. Shariffuddin Bin Khalid	3,504	300	–	–	–	–	–	–	–	–	3,804
Mr. Nor Hizam Bin Hashim	2,268	130	–	–	–	–	–	–	–	–	2,398
	26,139	1,390	1,120	990	460	1,090	630	660	–	25,000	57,479

*During the year, the Board Chairman was paid proportionate amount of Rs 2.370 million in lieu of fixed annual remuneration (2019: Rs. 25 million) approved by the shareholders of the Bank in its 62nd Annual General Meeting held on March 26, 2010. Effective from February 05, 2020 in accordance with BPRD Circular No. 03 of 2019 dated August 19, 2019, the remuneration to the Chairman for attending the Board and committee meetings was paid inline with the remuneration scale approved by the shareholders of the Bank in its 72nd Annual General Meeting held on March 19, 2020.

40.3 The Chairman has been provided with free use of the Bank maintained car. In addition to the above, the Chief Executive and certain executives are provided with free use of the Bank's maintained cars and household equipment in accordance with the terms of their employment.

41. FAIR VALUE MEASUREMENTS

The fair value of traded investments is based on quoted market prices, except for tradable securities classified by the Bank as ‘held to maturity’. Quoted securities classified as held to maturity are carried at amortised cost. Fair value of unquoted equity investments other than investments in associates and subsidiaries is determined on the basis of break up value of these investments as per the latest available financial statements.

Fair value of fixed term loans, other assets, other liabilities, fixed term deposits and borrowings cannot be calculated with sufficient reliability due to absence of current and active market for such assets and liabilities and reliable data regarding market rates for similar instruments. The provision for impairment of loans and advances has been calculated in accordance with the Bank’s accounting policy as stated in note 6.4 to these unconsolidated financial statements.

The maturity and repricing profile and effective rates are stated in note 45.

In the opinion of the management, the fair value of the financial assets and financial liabilities other than those carried at fair value and disclosed in note 41.1 are not significantly different from their carrying values since assets and liabilities are either short-term in nature or re-priced over short term.

Notes To The Unconsolidated Financial Statements
For the year ended December 31, 2020

41.1 Fair value of financial assets

The Bank measures fair values using the following fair value hierarchy that reflects the significance of the inputs used in making the measurements:

Level 1: Fair value measurements using quoted prices (unadjusted) in active markets for identical assets or liabilities.

Level 2: Fair value measurements using inputs other than quoted prices included within Level 1 that are observable for the assets or liability, either directly (i.e. as prices) or indirectly (i.e. derived from prices).

Level 3: Fair value measurements using input for the asset or liability that are not based on observable market data (i.e. unobservable inputs).

Valuation techniques used in determination of fair valuation of financial instruments within level 2

Item	Valuation approach and input used
Federal Government securities	The fair values of Federal Government securities are determined using the PKRV rates.
Term Finance and Bonds	Investments in debt securities (comprising term finance certificates, bonds and any other security issued by a company or a body corporate for the purpose of raising funds in the form of redeemable capital) are valued on the basis of the rates announced by the Mutual Funds Association of Pakistan (MUFAP) in accordance with the methodology prescribed by the Securities and Exchange Commission of Pakistan.
Foreign exchange contracts	The valuation has been determined by interpolating the mid rates announced by the State Bank of Pakistan.
Derivatives	The fair values of derivatives which are not quoted in active markets are determined by using valuation techniques. The valuation techniques take into account the relevant underlying parameters including foreign currency involved, interest rates, yield curves, volatilities, contracts duration etc.
Operating fixed assets (land and building) & NBA	Land and buildings are revalued every three years using professional valuers on the panel of Pakistan Banker's Association. The valuation is based on their assessment of market value of the properties.

The table below analyses the financial and non-financial assets carried at fair values, by valuation methods. For financial assets, the Bank essentially carries its investments in debt and equity securities at fair values. Valuation of investments is carried out as per guidelines specified by the SBP. In case of non-financial assets, the Bank has adopted revaluation model (as per IAS 16) in respect of land and building.

Notes To The Unconsolidated Financial Statements
For the year ended December 31, 2020

	2020				
	Carrying value	Level 1	Level 2	Level 3	Total
	(Rupees in '000)				
**On balance sheet financial instruments**					
**Financial assets - measured at fair value**					
Investments					
Federal Government Securities	957,482,754	–	957,482,754	–	957,482,754
Shares	18,171,840	18,171,840	–	–	18,171,840
Non-Government Debt Securities	1,800,092	–	1,800,092	–	1,800,092
Foreign Securities	7,474,188	–	7,474,188	–	7,474,188
**Financial assets - disclosed but not measured at fair value**					
Investments (HTM, unlisted ordinary shares, subsidiaries and associates)	30,940,574	–	–	–	–
Cash and balances with treasury banks	122,180,839	–	–	–	–
Balances with other banks	24,030,328	–	–	–	–
Lendings to financial institutions	17,139,453	–	–	–	–
Advances	462,941,787	–	–	–	–
Other assets	46,267,752	–	–	–	–
**Non - Financial Assets measured at fair value**					
Operating fixed assets (land and buildings)	44,275,487	–	44,275,487	–	44,275,487
Non-banking assets	4,036,914	–	4,036,914	–	4,036,914
**Off-balance sheet financial instruments - measured at fair value**					
Forward purchase of foreign exchange	168,432,858	–	3,902,198	–	3,902,198
Forward sale of foreign exchange	149,987,717	–	4,271,423	–	4,271,423
Derivatives purchase	2,158,111	–	517,033	–	517,033
Derivatives sale	2,313,272	–	513,343	–	513,343

Notes To The Unconsolidated Financial Statements
For the year ended December 31, 2020

	2019				
	Carrying value	Level 1	Level 2	Level 3	Total
	(Rupees in '000)				
**On balance sheet financial instruments**					
**Financial assets - measured at fair value**					
Investments					
Federal Government Securities	680,585,359	–	680,585,359	–	680,585,359
Shares	14,912,747	14,912,747	–	–	14,912,747
Non-Government Debt Securities	1,306,414	–	1,306,414	–	1,306,414
Foreign Securities	3,663,065	–	3,663,065	–	3,663,065
**Financial assets - disclosed but not measured at fair value**					
Investments (HTM, unlisted ordinary shares, subsidiaries and associates)	48,296,917	–	–	–	–
Cash and balances with treasury banks	132,704,797	–	–	–	–
Balances with other banks	12,542,239	–	–	–	–
Lendings to financial institutions	1,090,058	–	–	–	–
Advances	496,678,874	–	–	–	–
Other assets	52,808,501	–	–	–	–
**Non - Financial Assets measured at fair value**					
Operating fixed assets (land and buildings)	43,552,234	–	43,552,234	–	43,552,234
Non-banking assets	3,838,230	–	3,838,230	–	3,838,230
**Off-balance sheet financial instruments - measured at fair value**					
Forward purchase of foreign exchange	217,809,539	–	4,146,908	–	4,146,908
Forward sale of foreign exchange	187,805,779	–	3,379,897	–	3,379,897
Derivatives purchase	5,176,612	–	1,232,827	–	1,232,827
Derivatives sale	5,068,194	–	1,229,116	–	1,229,116

The Bank's policy is to recognise transfers into and out of the different fair value hierarchy levels at the date the event or change in circumstances that caused the transfer occurred. There were no transfers between levels 1 and 2 during the year.

(a) **Financial instruments in level 1**

Financial instruments included in level 1 comprise of investments in listed ordinary shares and units of mutual funds.

(b) **Financial instruments in level 2**

Financial instruments included in level 2 comprise of Sukuk Bonds, Pakistan Investment Bonds, Market Treasury Bills, Term Finance certificates, FX options, Cross Currency Swaps, Interest Rate Swaps and Forward Exchange Contracts.

(c) **Financial instruments in level 3**

Currently, no financial instruments are classified in level 3.

Notes To The Unconsolidated Financial Statements

For the year ended December 31, 2020

42 SEGMENT INFORMATION

42.1 Segment details with respect to business activities

The segment analysis with respect to business activity is as follows:

	2020								
	Retail Banking	Consumer Banking	Corporate Banking	Treasury	International Banking	Others	Sub-total	Eliminations	Total
	(Rupees in '000)								
**Profit & Loss**									
Net mark-up/return/profit	(44,807,213)	2,788,656	28,085,051	83,819,922	1,448,075	–	71,334,491	–	71,334,491
Inter segment revenue - net	86,028,798	(486,242)	(22,360,468)	(69,939,999)	(171,994)	6,929,905	–	–	–
Non mark-up / return / interest income	6,306,650	2,118,001	3,282,442	5,618,895	813,588	(3,789)	18,135,787	–	18,135,787
Total Income	47,528,235	4,420,415	9,007,025	19,498,818	2,089,669	6,926,116	89,470,278	–	89,470,278
Segment direct expenses	20,460,118	1,462,911	586,415	368,025	1,309,898	9,720,790	33,908,157	–	33,908,157
Inter segment expense allocation	–	–	–	–	–	–	–	–	–
Total expenses	20,460,118	1,462,911	586,415	368,025	1,309,898	9,720,790	33,908,157	–	33,908,157
Provisions	2,306,248	113,643	2,763,155	(31,111)	129,368	2,031,863	7,313,166	–	7,313,166
Profit before tax	24,761,869	2,843,861	5,657,455	19,161,904	650,403	(4,826,537)	48,248,955	–	48,248,955
**Balance Sheet**									
Cash and Bank balances	58,362,119	317,242	394,030	64,577,425	21,166,578	1,393,773	146,211,167	–	146,211,167
Investments	–	–	10,578,310	990,720,067	14,571,071	–	1,015,869,448	–	1,015,869,448
Net inter segment lending	1,050,376,236	–	–	–	–	201,834,399	1,252,210,635	(1,252,210,635)	–
Lendings to financial institutions	–	–	–	6,137,258	11,002,195	–	17,139,453	–	17,139,453
Advances - performing	94,735,206	26,845,772	313,633,477	–	21,681,241	–	456,895,696	–	456,895,696
-non performing - net	171,804	177,613	2,382	–	4,973,954	720,338	6,046,091	–	6,046,091
Others	35,621,546	2,370,726	22,789,948	12,206,991	4,580,374	37,731,030	115,300,615	–	115,300,615
**Total Assets**	1,239,266,911	29,711,353	347,398,147	1,073,641,741	77,975,413	241,679,540	3,009,673,105	(1,252,210,635)	1,757,462,470
Borrowings	58,910,004	–	10,372,566	91,069,170	3,649,793	–	164,001,533		164,001,533
Deposits and other accounts	1,147,268,725	21,263,015	65,961,390	–	55,009,174	–	1,289,502,304		1,289,502,304
Net inter segment borrowing	–	4,816,853	252,358,835	981,733,802	13,301,145	–	1,252,210,635	(1,252,210,635)	–
Others	33,088,182	3,631,485	18,705,356	838,769	6,015,301	51,577,585	113,856,678		113,856,678
**Total liabilities**	1,239,266,911	29,711,353	347,398,147	1,073,641,741	77,975,413	51,577,585	2,819,571,150	(1,252,210,635)	1,567,360,515
Equity	–	–	–	–	–	190,101,955	190,101,955	–	190,101,955
**Total Equity & liabilities**	1,239,266,911	29,711,353	347,398,147	1,073,641,741	77,975,413	241,679,540	3,009,673,105	(1,252,210,635)	1,757,462,470
**Contingencies & Commitments**	55,974,597	–	288,001,956	320,068,131	20,930,195	29,063,504	714,038,383	–	714,038,383

	2019								
	Retail Banking	Consumer Banking	Corporate Banking	Treasury	International Banking	Others	Sub-total	Eliminations	Total
	(Rupees in '000)								
**Profit & Loss**									
Net mark-up/return/profit	(50,496,018)	3,938,893	36,263,272	68,147,338	1,762,729	–	59,616,214	–	59,616,214
Inter segment revenue - net	99,832,569	(2,583,003)	(31,413,118)	(73,135,260)	(180,860)	7,479,672	–	–	–
Non mark-up / return / interest income	6,318,956	2,111,319	3,414,776	3,519,577	782,895	531,430	16,678,953	–	16,678,953
Total Income	55,655,507	3,467,209	8,264,930	(1,468,345)	2,364,764	8,011,102	76,295,167	–	76,295,167
Segment direct expenses	21,762,153	1,330,299	525,336	311,978	1,284,815	8,494,740	33,709,321	–	33,709,321
Inter segment expense allocation	–	–	–	–	–	–	–	–	–
Total expenses	21,762,153	1,330,299	525,336	311,978	1,284,815	8,494,740	33,709,321	–	33,709,321
Provisions	345,610	(34,217)	947,291	2,772,041	75,179	(1,622,370)	2,483,534	–	2,483,534
Profit before tax	33,547,744	2,171,127	6,792,303	(4,552,364)	1,004,770	1,138,732	40,102,312	–	40,102,312
**Balance Sheet**									
Cash and Bank balances	50,154,942	21,483	676,139	65,572,277	28,149,362	672,833	145,247,036	–	145,247,036
Investments	–	–	10,638,292	723,114,519	15,011,691	–	748,764,502	–	748,764,502
Net inter segment lending	940,015,024	–	–	–	–	164,292,627	1,104,307,651	(1,104,307,651)	–
Lendings to financial institutions	–	–	–	880,853	209,205	–	1,090,058	–	1,090,058
Advances - performing	98,464,054	24,342,756	341,191,287	–	25,190,939	–	489,189,036	–	489,189,036
-non performing - net	1,074,787	113,148	1,237,494	–	4,836,865	227,544	7,489,838	–	7,489,838
Others	34,759,963	1,882,259	25,393,004	13,804,786	2,368,790	45,162,743	123,371,545	–	123,371,545
**Total Assets**	1,124,468,770	26,359,646	379,136,216	803,372,435	75,766,852	210,355,747	2,619,459,666	(1,104,307,651)	1,515,152,015
Borrowings	45,355,965	–	7,057,504	33,572,200	3,520,223	–	89,505,892	–	89,505,892
Deposits and other accounts	1,033,804,761	–	55,425,048	–	55,523,627	9,823	1,144,763,259	–	1,144,763,259
Net inter segment borrowing	–	24,124,908	299,650,838	768,067,183	12,464,722	–	1,104,307,651	(1,104,307,651)	–
Others	45,308,044	2,234,738	17,002,826	1,733,052	4,258,280	41,431,141	111,968,081	–	111,968,081
**Total liabilities**	1,124,468,770	26,359,646	379,136,216	803,372,435	75,766,852	41,440,964	2,450,544,883	(1,104,307,651)	1,346,237,232
Equity	–	–	–	–	–	168,914,783	168,914,783	–	168,914,783
**Total Equity & liabilities**	1,124,468,770	26,359,646	379,136,216	803,372,435	75,766,852	210,355,747	2,619,459,666	(1,104,307,651)	1,515,152,015
**Contingencies & Commitments**	48,973,361	–	262,455,692	496,556,621	14,015,586	29,145,510	851,146,770	–	851,146,770

42.2 Segment details with respect to geographical locations

GEOGRAPHICAL SEGMENT ANALYSIS

	2020					
	Pakistan	South Asia	Middle East	Sub-total	Eliminations	Total
	(Rupees in '000)					
**Profit & Loss**						
Net mark-up/return/profit	69,887,495	862,868	584,128	71,334,491	–	71,334,491
Inter segment revenue - net	111,160	(95,930)	(15,230)	–	–	–
Non mark-up / return / interest income	17,325,905	265,900	543,982	18,135,787	–	18,135,787
Total Income	87,324,560	1,032,838	1,112,880	89,470,278	–	89,470,278
Segment direct expenses	32,601,367	650,469	656,321	33,908,157	–	33,908,157
Inter segment expense allocation	–	–	–	–	–	–
Total expenses	32,601,367	650,469	656,321	33,908,157	–	33,908,157
Provisions	7,183,798	78,422	50,946	7,313,166	–	7,313,166
Profit before tax	47,539,395	303,947	405,613	48,248,955	–	48,248,955
**Balance Sheet**						
Cash and Bank balances	125,365,815	1,556,335	19,289,017	146,211,167	–	146,211,167
Investments	1,001,298,378	10,519,130	4,051,940	1,015,869,448	–	1,015,869,448
Net inter segment lendings	12,319,043	–	–	12,319,043	(12,319,043)	–
Lendings to financial institutions	6,137,258	1,864,193	9,138,002	17,139,453	–	17,139,453
Advances - performing	435,310,355	12,722,090	8,863,251	456,895,696	–	456,895,696
- non performing - net	6,034,187	11,904	–	6,046,091	–	6,046,091
Others	110,746,462	4,057,318	496,835	115,300,615	–	115,300,615
**Total Assets**	1,697,211,498	30,730,970	41,839,045	1,769,781,513	(12,319,043)	1,757,462,470
Borrowings	160,351,740	1,790,053	1,859,740	164,001,533	–	164,001,533
Deposits and other accounts	1,237,359,663	18,298,522	33,844,119	1,289,502,304	–	1,289,502,304
Net inter segment borrowing	–	6,525,907	5,793,136	12,319,043	(12,319,043)	–
Others	109,398,140	4,116,488	342,050	113,856,678	–	113,856,678
**Total liabilities**	1,507,109,543	30,730,970	41,839,045	1,579,679,558	(12,319,043)	1,567,360,515
Equity	190,101,955	–	–	190,101,955	–	190,101,955
**Total Equity & liabilities**	1,697,211,498	30,730,970	41,839,045	1,769,781,513	(12,319,043)	1,757,462,470
Contingencies & Commitments	693,108,189	17,479,001	3,451,193	714,038,383	–	714,038,383

	2019					
	Pakistan	South Asia	Middle East	Sub-total	Eliminations	Total
	(Rupees in '000)					
**Profit & Loss**						
Net mark-up/return/profit	57,855,267	1,085,296	675,651	59,616,214	–	59,616,214
Inter segment revenue - net	107,447	(119,613)	12,166	–	–	–
Non mark-up / return / interest income	15,915,192	251,485	512,276	16,678,953	–	16,678,953
Total Income	73,877,906	1,217,168	1,200,093	76,295,167	–	76,295,167
Segment direct expenses	32,419,640	737,142	552,539	33,709,321	–	33,709,321
Inter segment expense allocation	–	–	–	–	–	–
Total expenses	32,419,640	737,142	552,539	33,709,321	–	33,709,321
Provisions	2,388,895	29,232	65,407	2,483,534	–	2,483,534
Profit before tax	39,069,371	450,794	582,147	40,102,312	–	40,102,312
**Balance Sheet**						
Cash and Bank balances	117,308,448	2,169,276	25,769,312	145,247,036	–	145,247,036
Investments	733,752,811	5,571,042	9,440,649	748,764,502	–	748,764,502
Net inter segment lendings	11,539,343	–	–	11,539,343	(11,539,343)	–
Lendings to financial institutions	880,852	209,206	–	1,090,058	–	1,090,058
Advances - performing	464,016,151	17,771,048	7,401,837	489,189,036	–	489,189,036
- non performing - net	7,460,190	29,648	–	7,489,838	–	7,489,838
Others	121,004,686	2,193,220	173,639	123,371,545	–	123,371,545
**Total Assets**	1,455,962,481	27,943,440	42,785,437	1,526,691,358	(11,539,343)	1,515,152,015
Borrowings	85,985,670	845,703	2,674,519	89,505,892	–	89,505,892
Deposits and other accounts	1,091,755,442	18,431,297	34,576,520	1,144,763,259	–	1,144,763,259
Net inter segment borrowing	–	6,151,041	5,388,302	11,539,343	(11,539,343)	–
Others	109,306,586	2,515,399	146,096	111,968,081	–	111,968,081
**Total liabilities**	1,287,047,698	27,943,440	42,785,437	1,357,776,575	(11,539,343)	1,346,237,232
Equity	168,914,783	–	–	168,914,783	–	168,914,783
**Total Equity & liabilities**	1,455,962,481	27,943,440	42,785,437	1,526,691,358	(11,539,343)	1,515,152,015
**Contingencies & Commitments**	837,131,184	9,436,548	4,579,038	851,146,770	–	851,146,770

42.3 Transactions between reportable segments are based on an appropriate transfer pricing mechanism using agreed rates. Furthermore, segment assets and liabilities include inter segment balances. Costs which are not allocated to segments are included in the Head office. Income taxes are managed at bank level and are not allocated to operating segments.

42.4 No revenue from transactions with a single external customer or counterparty amounted to 10% or more of the Bank's total revenue in 2020 or 2019.

Notes To The Unconsolidated Financial Statements
For the year ended December 31, 2020

43 RELATED PARTY TRANSACTIONS

The Bank has related party relationship with its subsidiaries, associates, employee benefit plans, its directors and key management personnel and their close family members.

The Banks enters into transactions with related parties in the ordinary course of business and on substantially the same terms as for comparable transactions with person of similar standing. Contributions to and accruals in respect of staff retirement benefits and other benefit plans are made in accordance with the actuarial valuations / terms of the contribution plan. Remuneration to the executives / officers is determined in accordance with the terms of their appointment.

Details of transactions with related parties during the year, other than those which have been disclosed elsewhere in these unconsolidated financial statements are as follows:

	2020					2019				
	Directors	Key management personnel	Subsidiaries	Associates	Other related Parties	Directors	Key management personnel	Subsidiaries	Associates	Other related parties
	(Rupees in '000)									
**Balances with other banks**										
In current accounts	–	–	–	–	–	–	–	–	–	14,389
	–	–	–	–	–	–	–	–	–	14,389
**Lendings to Financial Institutions**										
Opening balance	–	–	880,853	–	–	–	–	631,351	–	–
Addition during the year	–	–	23,703,928	–	–	–	–	22,355,349	–	–
Repaid during the year	–	–	(24,584,781)	–	–	–	–	(22,105,847)	–	–
Closing balance	–	–	–	–	–	–	–	880,853	–	–
**Investments**										
Opening balance	–	–	12,346,537	700,401	254,253	–	–	12,046,512	700,401	254,253
Investment made during the year	–	–	–	–	–	–	–	350,000	–	–
Investment disposed off during the year	–	–	(27,500)	–	–	–	–	–	–	–
Adjustment under amalgamation scheme	–	–	–	–	–	–	–	(49,975)	–	-
Closing balance	–	–	12,319,037	700,401	254,253	–	–	12,346,537	700,401	254,253
Provision for diminution in value of investments	–	–	725	–	5,000	–	–	725	–	5,000
**Advances**										
Opening balance	1,722	129,048	889,811	–	339,520	658	141,390	366,872	–	1,050,277
Addition / exchange adjustment during the year	18,202	60,257	–	356,898	336,695	29,148	62,216	522,939	–	1,642,434
Repaid during the year	(18,882)	(21,947)	(33,107)	–	(303,183)	(28,084)	(55,470)	–	–	(1,558,481)
Transfer in / (out)	–	(601)	–	–	(220,885)	–	(19,087)	–	–	(794,710)
Closing balance	1,042	166,757	856,704	356,898	152,147	1,722	129,048	889,811	–	339,520
Provision held against advances	–	–	–	–	–	–	–	–	–	–

	2020					2019				
	Directors	Key management personnel	Subsidiaries	Associates	Other related Parties	Directors	Key management personnel	Subsidiaries	Associates	Other related parties
	(Rupees in '000)									
**Other Assets**										
Markup receivable	–	3,149	2,338	895	2,382	–	3,202	31,552	–	5,191
Advances, deposits, advance rent and other prepayments	–	–	12,715	310,504	27,835	–	–	5,322	246,720	27,080
Acceptances	–	–	–	–	–	–	–	–	–	–
Receivable from Pension Fund	–	–	–	–	3,370,179	–	–	–	–	3,605,121
Unrealized gain on forward foreign exchange contracts - outstanding	–	–	39,415	–	–	–	–	27,493	–	–
Provision held against other assets	–	–	–	–	–	–	–	–	–	–
**Borrowings**										
Opening balance	–	–	3,902	–	69,166	–	–	19,146	–	–
Borrowings / exchange adjustment during the year	–	–	482,356	–	7,973	–	–	386,228	–	144,166
Settled during the year	–	–	(460,902)	–	–	–	–	(401,471)	–	(75,000)
Closing balance	–	–	25,356	–	77,139	–	–	3,902	–	69,166
**Deposits and other accounts**										
Opening balance	602,381	140,761	54,482	3,657,552	4,179,849	3,745,457	168,528	65,933	3,339,847	3,212,620
Received during the year	669,282	1,189,437	2,349,111	44,628,206	76,375,987	9,889,584	866,766	4,513,372	22,234,251	62,263,273
Withdrawn during the year	(969,533)	(1,163,832)	(2,346,830)	(43,469,978)	(75,154,115)	(13,025,336)	(884,532)	(4,515,594)	(21,916,546)	(61,423,281)
Transfer in / (out) - net	–	(27,800)	(2,885)	–	(531,780)	(7,324)	(10,001)	(9,229)	–	127,237
Closing balance	302,130	138,566	53,878	4,815,780	4,869,941	602,381	140,761	54,482	3,657,552	4,179,849
**Other Liabilities**										
Markup payable	50	100	–	42,549	10,654	7,263	556	158	50,535	66,906
Accrued expenses and other payable	–	–	22,850	62,624	32	15,326	–	23,134	62,402	50
Payable to MCB Employee Security Services	–	–	–	–	27,031	–	–	–	–	24,565
Advance received against sale of property	–	–	20,000	–	–	–	–	20,000	–	–
**Contingencies and Commitments**										
Commitments and contingent liabilities - outstanding	–	–	–	10,512	1,756,270	–	–	–	10,444	746,868
Forward foreign exchange contracts (Notional) - outstanding	–	–	1,342,106	–	–	–	–	2,044,093	–	–
Bank guarantee	–	–	102,038	–	–	–	–	26,711	–	–

Notes To The Unconsolidated Financial Statements

For the year ended December 31, 2020

	2020					2019				
	Directors	Key management personnel	Subsidiaries	Associates	Other related Parties	Directors	Key management personnel	Subsidiaries	Associates	Other related parties
	(Rupees in '000)									
**RELATED PARTY TRANSACTIONS**										
Income										
Markup / return / interest earned	–	13,098	143,011	1,809	13,189	–	12,357	208,168	–	20,846
Fee and commission income	–	–	30,253	1,177,371	1,638	–	–	56,047	1,301,954	4,631
Dividend income	–	–	120,109	192,500	36,213	–	–	49,892	175,000	41,162
Gain on forward foreign exchange contracts matured during the year	–	–	–	–	53,120	–	–	–	–	12,850
Net gain on sale of securities	72	40	–	3,836	6,485	–	–	–	281	10,990
Gain on sale of assets	–	98	–	–	–	–	186	–	–	–
Rent income and reimbursement of other expenses	–	–	42,514	8,808	2,280	–	–	40,100	8,485	3,105
**Expense**										
Markup / return / interest expensed	30,257	2,626	2,008	223,767	233,677	145,454	1,999	2,771	218,029	364,984
**Other Operating expenses**										
Clearing expenses paid to NIFT	–	–	–	–	166,175	–	–	–	–	152,009
Contribution to provident fund	–	–	–	-	394,598	–	–	–	–	366,744
Rent expenses	–	–	10,715	42,264	37,947	–	–	9,741	21,347	38,680
Cash sorting expenses	–	–	–	–	114,845	–	–	–	–	160,766
Stationery expenses	–	–	–	–	244,697	–	–	–	–	258,411
Security guards expenses	–	–	–	–	329,116	–	–	–	–	359,119
Remuneration to key executives and non-executive directors fee	177,166	509,676	–	–	–	165,579	514,168	–	–	–
Outsourcing service expenses	–	–	–	275,517	–	–	–	–	258,190	–
Donation Expense	–	–	–	–	95,000	–	–	–	-	–
E-dividend processing fee and CDC charges	–	–	–	–	4,757	–	–	–	–	5,968
Travelling expenses	–	–	–	–	38,507	–	–	–	–	37,644
Hotel stay expenses	–	–	–	–	3,410	–	–	–	–	2,573
Repair and maintenance charges	–	–	–	–	1,989	–	–	–	–	2,125
Advertisement expenses	–	–	–	–	7,308	–	–	–	–	12,934
Miscellaneous expenses and payments	–	–	–	–	3,225	–	–	–	–	2,376
Insurance premium-net of refund	–	–	–	646,676	–	–	–	–	719,873	–
Insurance claim settled	–	–	–	46,067	–	–	–	–	57,566	–
**Other Transactions**										
Proceeds from sale of assets	–	381	–	–	–	–	347	–	–	–
Purchase of fixed assets	–	–	1,550	3,277	5,712	–	–	–	46,339	20,003
Sale of foreign currency	–	–	45,457,828	–	–	–	–	35,675,520	–	–
Purchase of foreign currency	–	–	35,244,343	–	–	–	–	25,874,008	–	–
Payments against home remittances	–	–	3,898,627	–	–	–	–	2,980,860	–	–
Sale of government securities	268,847	124,304	–	5,740,348	8,592,672	203,735	94,105	–	6,043,338	22,634,697
Purchase of government securities	19,827	999	–	1,232,917	653,148	–	58,956	–	6,410,544	17,302,184
Forward exchange contracts matured during the year	–	–	–	–	11,446,226	–	–	–	–	24,426,964

The Chairman has been provided with free use of the Bank maintained car. The Chief Executive and certain executives are provided with free use of the Bank's maintained cars and household equipment in accordance with the terms of their employment.

Notes To The Unconsolidated Financial Statements

For the year ended December 31, 2020

44 CAPITAL ADEQUACY, LEVERAGE RATIO & LIQUIDITY REQUIREMENTS

44.1 Capital Adequacy

	2020	2019
	(Rupees in '000)	
**Minimum Capital Requirement (MCR):**		
Paid-up capital (net of losses)	11,850,600	11,850,600
**Capital Adequacy Ratio (CAR):**		
Eligible Common Equity Tier 1 (CET 1) Capital	152,901,428	136,256,771
Eligible Additional Tier 1 (ADT 1) Capital	–	–
Total Eligible Tier 1 Capital	152,901,428	136,256,771
Eligible Tier 2 Capital	35,507,111	27,354,014
**Total Eligible Capital (Tier 1 + Tier 2)**	188,408,539	163,610,785
**Risk Weighted Assets (RWAs):**		
Credit Risk	635,599,185	638,492,645
Market Risk	122,603,850	108,276,486
Operational Risk	139,735,092	120,887,137
**Total**	897,938,127	867,656,268
**Common Equity Tier 1 Capital Adequacy ratio**	17.03%	15.70%
**Tier 1 Capital Adequacy Ratio**	17.03%	15.70%
**Total Capital Adequacy Ratio**	20.98%	18.86%

The SBP through its BSD Circular No. 07 dated April 15, 2009 has prescribed the minimum paid up capital (net of losses) for all locally incorporated banks of Rs. 10 billion. The paid up capital of the Bank for the year ended December 31, 2020 stood at Rs. 11.851 billion and is in compliance with the SBP requirements. Further, under Basel III instructions, banks are required to maintain minimum Capital Adequacy Ratio (CAR) of 11.50% (including a capital conservation buffer of 1.5% which has been revised downwards from 2.5% as per BPRD Circular No. 12 dated March 26, 2020), Common Equity Tier 1 (CET 1) ratio of 6.0% and Tier 1 ratio of 7.50% as at December 31, 2020. The Bank is fully compliant with prescribed ratios.

Under the current capital adequacy regulations, credit risk and market risk exposures are measured using the Standardized Approach and operational risk is measured using the Basic Indicator Approach. Credit risk mitigants are also applied against the Bank's exposures based on eligible collateral under simple approach.

44.2 Leverage Ratio (LR):

	2020	2019
	(Rupees in '000)	
Eligible Tier-1 Capital	152,901,428	136,256,771
Total Exposures	2,174,932,446	1,928,383,315
Leverage Ratio	7.03%	7.07%

Notes To The Unconsolidated Financial Statements
For the year ended December 31, 2020

	2020	2019
	(Rupees in '000)	
**44.3 Liquidity Requirements**		
**Liquidity Coverage Ratio (LCR):**		
Total High Quality Liquid Assets	934,508,535	713,965,089
Total Net Cash Outflow	393,109,786	362,188,259
Liquidity Coverage Ratio	237.72%	197.13%
**Net Stable Funding Ratio (NSFR):**		
Total Available Stable Funding	1,130,301,361	983,364,374
Total Required Stable Funding	646,417,507	699,043,391
Net Stable Funding Ratio	174.86%	140.67%

The full disclosures on the Capital Adequacy, Leverage Ratio & Liquidity Requirements as per SBP instructions issued from time to time are available at https://www.mcb.com.pk/investor-relations/capital-adequacy-statements.

45 RISK MANAGEMENT

Risk is an inherent part of banking business activities. The risk management framework and governance structure at MCB helps to mitigate and counter any foreseeable risk in its various lines of business. Risk awareness forms an integral part of strategic and operational activities of risk management. Through its Global Risk Management Policy, Bank sets the best course of action under uncertainty by identifying, prioritizing, mitigating and monitoring risk issues, with the goal of enhancing shareholders' value. Bank's risk management structure is based on the following five guiding principles:

- Optimizing risk/return in a controlled manner
- Establishing clear responsibility and accountability
- Establishing independent and properly resourced risk management function.
- Promoting open risk culture
- Adopting international best practices in risk management

Keeping in view dynamics of internal and external environment, the bank regularly reviews and updates policy manuals / frameworks and procedures in accordance with domestic regulatory environment and international standards.

The Bank executes its risk strategy and undertakes controlled risk-taking activities within its risk management framework. The Board of Directors and its relevant committee, i.e. the Risk Management & Portfolio Review Committee (RM&PRC), the senior management and its relevant committees, i.e. the Management Credit and Risk Committee (MC&RC), Asset Liability Committee (ALCO), etc., are responsible to ensure formulation and implementation of comprehensive Risk Management Framework. This framework is based on prudent risk identification, measurement, management and monitoring processes which are closely aligned with the activities of the bank. The framework combines core policies, procedures and process designs with broad oversight and is supported by an efficient monitoring mechanism across the bank to ensure that risks are kept within an acceptable level.

The Bank ensures that not only the relevant risks are identified but their implications are also considered and basis provided for managing and measuring the risks. Through Internal Control units, the Bank ensures that effective controls are in place to mitigate each of the identified risk.

Independent from business groups, Head of Risk Management reports functionally to the Risk Management & Portfolio Review Committee (RM&PRC) and administratively to the President; the RM&PRC convenes regular meetings to evaluate bank's risk and portfolio concentrations. The Risk Management Group performs the following critical functions:

- Risk Management Policy Formulation
- Credit Risk Management
- Credit Review

- Credit Risk Control
- Market Risk Management
- Liquidity Risk Management
- Operational Risk Management
- IT Risk Management

Keeping in view the international best practices and SBP requirements, Board of Directors of the Bank has approved a Risk Appetite Statement, which takes into account quantitative and qualitative risk indicators, covering target ratios, credit, market, operational, liquidity and business risks.

45.1 Credit Risk

Credit risk arises from bank's dealings with individuals, corporate borrowers, financial institutions, sovereigns etc. The Bank is exposed to credit risk through its lending and investment activities. Credit risk makes up the largest part of the Bank's exposure and it stems from Bank's both on and off-balance sheet activities. Purpose of Credit Risk Management function is to identify, measure, manage, monitor and mitigate credit risk. To manage adverse outcomes in terms of unfavorable scenarios, multiple control factors in the lending structure of the Bank provide additional comfort and support. Such controls range from quality of eligible collateral, pre-disbursement safety measures to post disbursement monitoring.

The Bank has adopted Standardized Approach to measure Credit risk regulatory capital charge in compliance with Basel requirements. The approach mainly takes into account the assessment of external credit rating agencies. In line with SBP guidelines on Internal Credit Risk Rating Systems, the Bank has developed rating systems and all its borrowers are internally rated. In order to further enhance the credit risk analysis and the processes, Probability Default based internal credit risk rating (ICRR) system based on the statistical modeling and validation in line with Basel principles. The revamped ICRR is currently focused on Corporate Commercial and Corporate Large customer categories. The Internal Credit Risk Rating Model for rating of SME Customers has also been revamped to achieve more accurate results and to improve the quality of credit decisions.

In order to manage bank's credit risk, following policies and procedures are in place:

- Individuals who take or manage risks clearly understand them in order to protect the Bank from avoidable risks;
- The approval of credit limits to counter parties are subject to pre-fact review;
- Extension in credit facility or material change to the credit facility is subject to credit review;
- Approval and review process is reviewed by RM&PRC and internal audit;
- Management periodically reviews the powers of credit approving and credit reviewing authorities.

Ongoing administration of the credit portfolio is an essential part of the credit process that supports and controls extension and maintenance of credit. The Bank's Credit Risk Control is responsible for performing following activities:

- Credit disbursement authorization
- Collateral coverage and monitoring
- Compliance of loan covenants/ terms of approval
- Maintenance/ custody of collateral and security documentation
- Credit Risk Limit Controls

Credit Risk Monitoring is based on a comprehensive reporting framework. Continuous monitoring of the credit portfolio and the risks attached thereto are carried out at different levels including businesses, Audit & Risk Assets Review, Credit Risk Control, Credit Risk Management Division, etc.

To ensure a prudent distribution of asset portfolio, the Bank manages its lending and investment activities within an appropriate limits framework. Per party exposure limit is maintained in accordance with SBP Prudential Regulations.

The Bank creates specific provision against Non-Performing Loans (NPLs) in accordance with the Prudential Regulations and other directives issued by the State Bank of Pakistan (SBP) and charged to the profit and loss account. Provisions are held against identified as well as unidentified losses. Provisions against unidentified losses include general provision against consumer loans and Small

Notes To The Unconsolidated Financial Statements
For the year ended December 31, 2020

enterprise (SEs) made in accordance with the requirements of the Prudential Regulations issued by SBP and provision based on historical loss experience on advances. General provisions pertaining to overseas advances are made in accordance with the requirements of the regulatory authorities of the respective countries. Please refer note No. 11.4 for reconciliation of changes in specific and general provisions.

The Risk Management function of the Bank has further strengthened its credit review procedures in the light of COVID-19 and is regularly conducting assessments of the credit portfolio to identify borrowers most likely to be affected due to changes in the business and economic environment.

Management of Non Performing Loans

The Bank has a Special Assets Management (SAM) function, which is responsible for management of non performing loans. SAM undertakes restructuring / rescheduling of problem loans, as well as litigation of both civil and criminal cases for collection of debt.

Stress Testing

Credit Risk stress testing is a regular exercise. Bank's credit exposures including funded and non-funded facilities are subjected to stress tests. This exercise is conducted on a quarterly basis through assigning shocks to all assets of the Bank and assessing its resulting affect on capital adequacy inline with SBP requirements.

45.1.1 Lendings to financial institutions

Credit risk by public / private sector

	Gross lendings		Non - performing lendings		Provision hold	
	2020	2019	2020	2019	2020	2019
	(Rupees in '000)					
Public/ Government	15,975,261	209,206	–	–	–	–
Private	1,164,192	880,852	–	–	–	–
	17,139,453	1,090,058	–	–	–	–

45.1.2 Investment in debt securities

Credit risk by industry sector

	Gross Investments		Non - performing Investments		Provision hold	
	2020	2019	2020	2019	2020	2019
	(Rupees in '000)					
Chemical and pharmaceuticals	1,750,000	1,750,000	–	–	–	–
Electricity, gas, steam and air conditioning supply	–	101,933	–	–	–	–
Financials including government securities	971,996,914	712,505,023	118	118	118	118
Manufacture of cement	285,000	285,000	285,000	285,000	285,000	285,000
Manufacture of sugar	145,656	145,656	145,656	145,656	145,656	72,828
Manufacture of textiles	53,531	53,531	53,531	53,531	53,531	53,531
Telecommunications	–	114,747	–	114,747	–	114,747
Others	6,154	7,682	6,154	7,682	6,154	7,682
	974,237,255	714,963,572	490,459	606,734	490,459	533,906
**Credit risk by public / private sector**						
Public/ Government	963,820,326	703,333,310	–	–	–	–
Private	10,416,929	11,630,262	490,459	606,734	490,459	533,906
	974,237,255	714,963,572	490,459	606,734	490,459	533,906

MCB
Bank for Life

Notes To The Unconsolidated Financial Statements
For the year ended December 31, 2020

45.1.3 Advances

Credit risk by industry sector

	Gross Advances		Non - performing Advances		Provision hold	
	2020	2019	2020	2019	2020	2019
	(Rupees in '000)					
Agriculture, forestry and fishing	5,122,317	6,209,291	477,421	390,080	437,355	362,867
Construction	18,081,634	18,950,532	289,791	289,435	278,464	287,840
Electricity, gas, steam and air conditioning supply	37,521,494	42,221,235	376,717	379,846	374,996	378,125
Electronics and electrical appliances	5,842,215	4,322,348	102,262	113,496	101,825	107,776
Financials	17,534,127	20,897,873	462,665	532,286	462,665	532,286
Footwear and Leather garments	3,778,108	4,357,262	170,131	173,985	169,736	173,590
Human health and social work activities	922,333	2,760,760	45,596	50,211	34,973	34,981
Individuals	43,906,423	39,528,041	4,316,081	4,377,113	3,911,755	3,999,488
Manufacture of basic metals and metal products	14,913,709	16,420,515	3,028,467	3,104,026	3,018,387	2,892,814
Manufacture of cement	9,742,405	10,344,007	392,862	392,862	392,862	392,862
Manufacture of chemicals and pharmaceutical products	42,384,893	45,734,806	275,980	334,485	273,047	331,551
Manufacture of coke and refined petroleum products	4,982,529	14,789,736	412,061	399,317	411,445	398,701
Manufacture of food & beverages products	39,631,918	51,582,644	3,274,437	3,014,962	3,058,910	2,786,691
Manufacture of machinery, equipment and transport Equipment	2,182,220	4,100,112	433,943	435,301	396,551	434,527
Manufacture of rubber and plastics products	4,034,047	5,020,189	665,778	661,498	662,506	658,226
Manufacture of sugar	33,283,221	23,116,287	4,658,087	4,497,643	4,655,219	3,434,211
Manufacture of textiles	76,794,166	78,774,991	13,475,285	13,537,492	13,322,828	13,088,287
Mining and quarrying	5,033,270	4,252,835	5,019	7,286	5,019	7,286
Manufacturing of Pulp, Paper, Paperboard	2,492,010	3,778,008	179,539	242,331	150,661	213,453
Ship Breaking	5,520,548	4,916,424	4,348,014	2,436,614	4,348,014	2,436,614
Services	10,353,488	10,694,258	464,150	596,857	460,207	588,408
Telecommunications	18,287,167	15,883,537	42,798	42,798	42,798	42,798
Transportation and storage	53,061,768	57,884,271	75,901	93,955	66,253	84,307
Wholesale and retail traders	53,691,912	47,103,072	12,521,768	12,562,163	7,471,824	7,573,707
Others	4,452,280	6,394,182	694,294	758,217	634,656	693,025
	513,550,202	540,037,216	51,189,047	49,424,259	45,142,956	41,934,421
**Credit risk by public / private sector**						
Public/ Government	74,377,320	85,944,207	639,825	639,825	639,825	639,825
Private	439,172,882	454,093,009	50,549,222	48,784,434	44,503,131	41,294,596
	513,550,202	540,037,216	51,189,047	49,424,259	45,142,956	41,934,421

Notes To The Unconsolidated Financial Statements
For the year ended December 31, 2020

		2020	2019
		(Rupees in '000)	
45.1.4	**Contingencies and Commitments**		
	**Credit risk by industry sector**		
	Agriculture, forestry and fishing	2,134,487	541,768
	Construction	20,639,681	27,382,939
	Electricity, gas, steam and air conditioning supply	24,907,422	16,080,103
	Electronics and electrical appliances	4,968,549	3,145,057
	Financials	390,150,887	579,135,125
	Footwear and Leather garments	517,178	80,901
	Human health and social work activities	1,612,922	409,816
	Individuals	2,618,245	6,205,880
	Manufacture of basic metals and metal products	8,207,595	6,414,150
	Manufacture of cement	4,686,576	1,294,594
	Manufacture of chemicals and pharmaceutical products	25,507,582	19,042,481
	Manufacture of coke and refined petroleum products	8,129,298	15,218,033
	Manufacture of food & beverages products	19,214,176	18,244,851
	Manufacture of machinery, equipment and transport Equipment	8,946,430	6,755,084
	Manufacture of rubber and plastics products	4,883,983	2,041,121
	Manufacture of sugar	3,939,151	3,640,983
	Manufacture of textiles	33,696,487	31,827,288
	Mining and quarrying	144,328	460,490
	Manufacturing of Pulp, Paper, Paperboard	1,362,984	1,163,879
	Ship Breaking	412,969	498,050
	Services	57,348,336	54,955,804
	Telecommunications	17,006,854	14,669,698
	Transportation and storage	10,643,212	3,394,704
	Wholesale and retail traders	25,016,888	16,723,095
	Others	37,342,163	21,820,876
		714,038,383	851,146,770
	**Credit risk by public / private sector**		
	Public/ Government	216,856,696	321,606,927
	Private	497,181,687	529,539,843
		714,038,383	851,146,770

45.1.5 Concentration of Advances

The bank top 10 exposures on the basis of total (funded and non-funded exposures) aggregated to Rs 235,823.529 million (2019: Rs. 236,769.236 million) are as following:

	2020	2019
	(Rupees in '000)	
Funded	75,373,723	80,717,454
Non Funded	160,449,536	156,051,782
Total Exposure	235,823,259	236,769,236

The sanctioned limits against these top 10 exposures aggregated to Rs 311,201.617 million (2019: 291,923.228 million)

There is no provision against these top 10 exposures.

Notes To The Unconsolidated Financial Statements
For the year ended December 31, 2020

45.1.6 Advances - Province/Region-wise Disbursement & Utilization

	2020						
	Disbursements	Utilization					
		Punjab	Sindh	KPK including FATA	Balochistan	Islamabad	AJK including gilgit Balochistan
	(Rupees in '000)						
**Province/Region**							
Punjab	419,237,388	374,143,233	37,128,846	6,191,473	337,087	1,436,749	-
Sindh	411,850,220	12,962,814	351,555,947	6,776,970	40,554,489	-	-
KPK including FATA	4,741,216	171,325	-	4,569,891	-	-	-
Balochistan	1,676,862	-	-	-	1,676,862	-	-
Islamabad	15,574,363	1,180,640	-	2,287,606	-	12,106,117	-
AJK including Gilgit-Baltistan	281,068	62,557	-	-	-	-	218,511
Total	853,361,117	388,520,569	388,684,793	19,825,940	42,568,438	13,542,866	218,511

	2019						
	Disbursements	Utilization					
		Punjab	Sindh	KPK including FATA	Balochistan	Islamabad	AJK including gilgit Balochistan
	(Rupees in '000)						
**Province/Region**							
Punjab	572,121,989	500,803,912	57,758,820	10,024,921	322,571	3,210,918	847
Sindh	577,715,877	12,573,575	494,016,936	3,370,558	67,526,981	227,827	-
KPK including FATA	5,441,928	67,761	-	5,374,167	-	-	-
Balochistan	1,216,050	-	-	-	1,216,050	-	-
Islamabad	32,691,486	3,470,284	227,242	1,782,828	-	27,135,929	75,203
AJK including Gilgit-Baltistan	316,940	425	-	-	-	-	316,515
Total	1,189,504,270	516,915,957	552,002,998	20,552,474	69,065,602	30,574,674	392,565

45.2 Market Risk

Market Risk arises from changes in market rates such as Interest Rates, Foreign Exchange Rates, Equity Prices, credit spreads and/or commodity prices as well as their correlations and volatilities resulting in a loss to earnings and capital. MCB is exposed to market risk primarily through its trading activities, which are centered in the Treasury and Foreign Exchange Group and the Capital Market Division. Market risk also arises from market-making, facilitation of client business and proprietary positions in equities, fixed income and interest rate products and foreign exchange, which exposes bank to interest rate risk, foreign exchange risk and equity price risk.

The Bank's Market Risk Management structure consists of Risk Management & Portfolio Review Committee (RM&PRC) of the Board, Management Credit and Risk Committee, ALCO and independent Market Risk Management Division reporting directly to Group Head Risk Management. Market Risk function works in close partnership with the business segments to identify and monitor market risks throughout the Bank and to define market risk policies and procedures. Market Risk seeks to facilitate efficient risk/return management decisions, reduce volatility in operating performance and provide transparency into the Bank's market risk profile for senior management, the Board of Directors and regulators. Market risk authority, including both approval of market risk limits and approval of market risks is vested in the ALCO.

In line with regulatory requirements, MCB has clearly defined, in its Global Risk Management policy, the positions which shall be subject to market risk. The definition covers the accounting classifications as well as positions booked by different business groups under "Available for Sale" category. The assets subject to trading book treatment are frequently, mostly on daily basis, valued and actively managed. The positions which does not fulfill the criteria of Trading book falls under the Banking Book and are treated as per SBP requirements.

Notes To The Unconsolidated Financial Statements

For the year ended December 31, 2020

The Bank measures and manages Market Risk by using different risk parameters with combinations of various limits. Board approved Global Risk Management Policy provides guidelines for assuming controlled market risk, its monitoring and management. The approved limits are compared with the numbers generated by the market risk management systems based on the trading activity and the outstanding positions.

Besides conventional methods, the Bank also uses VaR (Value at Risk) technique for market risk assessment of positions assumed by its treasury and capital market groups. In-house based solutions are used for calculating mark to market value of positions and generating VaR (value at risk) and sensitivity numbers. Thresholds for different positions are established to compare the expected losses at a given confidence level and over a specified time horizon.

A framework of stress testing, scenario analysis and reverse stress tests covering both banking and trading books as per SBP guidelines is also in place. The results of the stress tests are reviewed by senior management and also reported to the SBP.

The Bank is also exposed to interest rate risk both in trading and banking books. Risk parameters along with the marked to market values of government securities held by the Bank's treasury are generated on daily basis. The risk parameters include duration, PVBP, and VaR on individual security basis as well as on portfolio basis. These reports are presented to the senior management for review on a daily basis.

45.2.1 Balance sheet split by trading and banking books

	2020			2019		
	Banking book	Trading book	Total	Banking book	Trading book	Total
	(Rupees in '000)					
Cash and balances with treasury banks	122,180,839	–	122,180,839	132,704,797	–	132,704,797
Balances with other banks	24,030,328	–	24,030,328	12,542,239	–	12,542,239
Lendings to financial institutions	17,139,453	–	17,139,453	1,090,058	–	1,090,058
Investments	29,521,903	986,347,545	1,015,869,448	46,902,418	701,862,084	748,764,502
Advances	462,941,787	–	462,941,787	496,678,874	–	496,678,874
Fixed assets	58,027,904	–	58,027,904	58,271,245	–	58,271,245
Intangible assets	938,458	–	938,458	957,552	–	957,552
Other assets	56,334,253	–	56,334,253	64,142,748	–	64,142,748
	771,114,925	986,347,545	1,757,462,470	813,289,931	701,862,084	1,515,152,015

45.2.2 Foreign Exchange Risk

Foreign exchange risk exposes the bank to changes in the values of current holdings and future cash flows denominated in currencies other than home currency due to the exchange rate fluctuation and volatility. The types of instruments exposed to this risk include investments in foreign branches, foreign currency-denominated loans, foreign currency-denominated deposits, future cash flows in foreign currencies arising from foreign exchange transactions, etc.

The core objective of foreign exchange risk management is to ensure the foreign exchange exposure of the Bank remain within defined risk appetite and insulate bank against undue losses that may arise due to volatile movements in foreign exchange rates or interest rates.

Limit structure to manage Foreign exchange risk including gap limits on different tenors in major currencies are in place to control risk. Bank's net open position and foreign exchange exposure limit (FEEL) is monitored and reported on intra-day and day end basis. Foreign exchange risk parameters including VaR is generated and monitored on daily basis. Stress testing of foreign exchange portfolio and its reporting to senior management and RM&PRC of the Board is a regular feature.

Notes To The Unconsolidated Financial Statements

For the year ended December 31, 2020

	2020				2019			
	Foreign currency Assets	Foreign currency liabilities	Off--balance sheet items	Net foreign currency exposure	Foreign currency Assets	Foreign currency liabilities	Off--balance sheet items	Net foreign currency exposure
	(Rupees in '000)							
United States Dollar	44,103,967	54,985,985	10,749,737	(132,281)	38,440,687	59,119,303	21,719,452	1,040,836
Sri Lankan Rupees	144,228	–	–	144,228	–	164,290	148,959	(15,331)
Arad Emirates Dirham	–	248,645	39,598	(209,047)	194,163	949,404	590,192	(165,049)
Euro	2,433,944	6,576,870	4,143,526	600	3,187	4,574,963	4,564,076	(7,700)
Great Britain Pound Sterling	2,474,888	6,151,953	3,637,777	(39,288)	35,160	4,928,555	4,822,148	(71,247)
Japanese Yen	22,226	20	22,007	44,214	–	4,498	17,723	13,225
Other currencies	323,737	–	47,086	370,823	100,234	–	31,769	132,003
	49,502,990	67,963,473	18,639,731	179,249	38,773,431	69,741,013	31,894,319	926,737

	2020		2019	
	Banking book	Trading book	Banking book	Trading book
	(Rupees in '000)			
Impact of 1% change in foreign exchange rates on				
- Profit and loss account	–	1,792	–	9,267

45.2.3 Equity position Risk

Bank's proprietary positions in the equity instruments exposes it to the equity price risk in its trading and banking books. Equity price risk is managed by applying trading limit, scrip-wise and portfolio wise nominal limits. VaR analysis and stress testing of the equity portfolio are also performed and reported to senior management on a daily basis. The stress test for equity price risk assesses the impact of the fall in the stock market index using certain assumptions. In addition to this stress testing, historical scenario analysis on equities is also performed periodically as advised by the State Bank of Pakistan through Guideline on Stress Testing.

	2020		2019	
	AFS	HFT	AFS	HFT
	(Rupees in '000)			
Impact of 5% change in equity prices on				
- Profit and loss account	–	–	–	656
- Other comprehensive income	908,592	–	744,981	–

45.2.4 Yield / Interest Rate Risk in the Banking Book (IRRBB)-Basel II Specific

Yield/ Interest rate sensitivity position for on-balance sheet instruments is based on the earlier of contractual re-pricing or maturity date and for off-balance sheet instruments is based on settlement date.

The increase / (decrease) in earnings due to change in the interest rate is as follows:

	2020		2019	
	Banking Book	Trading Book	Banking Book	Trading Book
	(Rupees in '000)			
Impact of 1% increase in interest rates on				
- Profit and loss account	(3,987,679)	5,250,521	(2,621,559)	2,609,306
- Other comprehensive income	–	(7,497,582)	–	(6,716,523)

The Bank has classified Available for Sale investments as Trading in Basel-II.

Notes To The Unconsolidated Financial Statements

For the year ended December 31, 2020

45.2.5 Mismatch of Interest Rate Sensitive Assets and Liabilities

	2020											
	Effective yield / interest rate	Total	Exposed to Yield/ Interest risk									Non-interest bearing financial instruments
			Upto 1 month	Over 1 to 3 months	Over 3 to 6 months	Over 6 months to 1 year	Over 1 to 2 years	Over 2 to 3 years	Over 3 to 5 years	Over 5 to 10 years	Above 10 years	
		(Rupees in '000)										
**On-balance sheet financial instruments**												
**Assets**												
Cash and balances with treasury banks	-	122,180,839	6,810,656	-	-	-	-	-	-	-	-	115,370,183
Balances with other banks	0.21%	24,030,328	2,556,166	799,172	-	-	-	-	-	-	-	20,674,990
Lending to financial institutions	7.27%	17,139,453	17,139,453	-	-	-	-	-	-	-	-	-
Investments	10.77%	1,015,869,448	179,084,505	415,139,293	160,598,685	61,659,011	22,916,296	41,949,392	30,794,310	71,118,956	-	32,609,000
Advances	9.34%	462,941,787	432,461,756	6,982,362	3,401,653	1,931,754	3,619,904	1,742,088	4,803,725	1,859,527	6,139,018	-
Other assets		46,267,752	-	-	-	-	-	-	-	-	-	46,267,752
		1,688,429,607	638,052,536	422,920,827	164,000,338	63,590,765	26,536,200	43,691,480	35,598,035	72,978,483	6,139,018	214,921,925
**Liabilities**												
Bills payable		23,980,692	-	-	-	-	-	-	-	-	-	23,980,692
Borrowings	5.30%	164,001,533	121,266,747	8,214,674	5,044,522	1,813,871	8,382,537	2,571,133	4,831,690	11,876,359	-	-
Deposits and other accounts	4.50%	1,289,502,304	730,683,033	21,243,238	13,630,588	30,543,903	1,998,833	115,810	1,689,628	212,000	-	489,385,271
Other liabilities		60,731,156	-	-	-	-	-	-	-	-	-	60,731,156
		1,538,215,685	851,949,780	29,457,912	18,675,110	32,357,774	10,381,370	2,686,943	6,521,318	12,088,359	-	574,097,119
**On-balance sheet gap**		150,213,922	(213,897,244)	393,462,915	145,325,228	31,232,991	16,154,830	41,004,537	29,076,717	60,890,124	6,139,018	(359,175,194)
**Off-balance sheet financial instruments**												
FX options purchase		182,800	122,370	60,430	-	-	-	-	-	-	-	-
Forward purchase of Government securities		11,089,775	11,089,775	-	-	-	-	-	-	-	-	-
Cross Currency Swaps purchase		1,975,311	339,938	-	210,505	87,318	547,272	-	790,278	-	-	-
Interest Rate Swaps purchase		-	-	-	-	-	-	-	-	-	-	-
Foreign exchange contracts purchase		168,432,858	69,994,741	64,922,148	24,944,134	8,571,835	-	-	-	-	-	-
		181,680,744	81,546,824	64,982,578	25,154,639	8,659,153	547,272	-	790,278	-	-	-
FX options sale		182,800	122,370	60,430	-	-	-	-	-	-	-	-
Forward sale of Government securities		-	-	-	-	-	-	-	-	-	-	-
Cross Currency Swaps sale		2,130,472	340,109	-	210,505	242,308	547,272	-	790,278	-	-	-
Foreign exchange contracts sale		149,987,717	52,355,290	60,513,836	23,089,616	14,028,975	-	-	-	-	-	-
		152,300,989	52,817,769	60,574,266	23,300,121	14,271,283	547,272	-	790,278	-	-	-
**Off-balance sheet gap**		29,379,755	28,729,055	4,408,312	1,854,518	(5,612,130)	-	-	-	-	-	-
**Total Yield/Interest Risk Sensitivity Gap**			(185,168,189)	397,871,227	147,179,746	25,620,861	16,154,830	41,004,537	29,076,717	60,890,124	6,139,018	
**Cumulative Yield/Interest Risk Sensitivity Gap**			(185,168,189)	212,703,038	359,882,784	385,503,645	401,658,475	442,663,012	471,739,729	532,629,853	538,768,871	

Notes To The Unconsolidated Financial Statements
For the year ended December 31, 2020

	Effective yield / interest rate	Total	2019									
			Exposed to Yield/ Interest risk									Non-interest bearing financial instruments
			Upto 1 month	Over 1 to 3 months	Over 3 to 6 months	Over 6 months to 1 year	Over 1 to 2 years	Over 2 to 3 years	Over 3 to 5 years	Over 5 to 10 years	Above 10 years	
			(Rupees in '000)									
**On-balance sheet financial instruments**												
**Assets**												
Cash and balances with treasury banks	0.70%	132,704,797	10,517,551	-	-	-	-	-	-	-	-	122,187,246
Balances with other banks	0.55%	12,542,239	2,805,338	1,340,374	-	-	-	-	-	-	-	8,396,527
Lending to financial institutions	10.91%	1,090,058	1,090,058	-	-	-	-	-	-	-	-	-
Investments	11.47%	748,764,502	180,617,919	47,610,581	43,392,286	186,216,758	104,446,954	13,359,230	60,307,907	83,531,045	-	29,281,822
Advances	11.70%	496,678,874	465,897,240	13,865,557	2,430,422	980,072	1,816,256	3,009,684	1,481,043	1,754,233	5,444,367	-
Other assets		52,808,501	-	-	-	-	-	-	-	-	-	52,808,501
		1,444,588,971	660,928,106	62,816,512	45,822,708	187,196,830	106,263,210	16,368,914	61,788,950	85,285,278	5,444,367	212,674,096
**Liabilities**												
Bills payable		11,821,698	-	-	-	-	-	-	-	-	-	11,821,698
Borrowings	7.84%	89,505,892	54,865,233	9,993,161	7,185,921	1,467,529	2,097,344	2,217,657	3,572,934	8,106,113	-	-
Deposits and other accounts	5.96%	1,144,763,259	631,437,026	35,105,817	22,527,727	24,408,630	3,459,232	2,002,175	1,270,783	-	-	424,551,869
Other liabilities		76,717,784	-	-	-	-	-	-	-	-	-	76,717,784
		1,322,808,633	686,302,259	45,098,978	29,713,648	25,876,159	5,556,576	4,219,832	4,843,717	8,106,113	-	513,091,351
**On-balance sheet gap**		121,780,338	(25,374,153)	17,717,534	16,109,060	161,320,671	100,706,634	12,149,082	56,945,233	77,179,165	5,444,367	(300,417,255)
**Off-balance sheet financial instruments**												
FX options purchase		431,449	204,798	226,651	-	-	-	-	-	-	-	-
Outright purchase of Government Securities		82,284,304	82,284,304	-	-	-	-	-	-	-	-	-
Cross currency swaps - purchase		4,428,663	-	311,405	-	765,490	1,522,069	857,049	972,650	-	-	-
Interest Rate Swaps - purchase		316,500	-	-	-	316,500	-	-	-	-	-	-
Foreign exchange contracts purchase		217,809,539	82,724,358	85,102,271	45,299,680	4,683,230	-	-	-	-	-	-
		305,270,455	165,213,460	85,640,327	45,299,680	5,765,220	1,522,069	857,049	972,650	-	-	-
FX options sale		431,449	204,798	226,651	-	-	-	-	-	-	-	-
Forward sale of Government securities		5,412,334	5,412,334	-	-	-	-	-	-	-	-	-
Cross Currency Swaps - sale		4,636,745	-	311,405	52,745	765,490	1,677,406	857,049	972,650	-	-	-
Foreign exchange contracts sale		187,805,779	68,703,212	67,523,912	50,136,199	1,442,456	-	-	-	-	-	-
		198,286,307	74,320,344	68,061,968	50,188,944	2,207,946	1,677,406	857,049	972,650	-	-	-
**Off-balance sheet gap**		106,984,148	90,893,116	17,578,359	(4,889,264)	3,557,274	(155,337)	-				
**Total Yield/Interest Risk Sensitivity Gap**			65,518,963	35,295,893	11,219,796	164,877,945	100,551,297	12,149,082	56,945,233	77,179,165	5,444,367	
**Cumulative Yield/Interest Risk Sensitivity Gap**			65,518,963	100,814,856	112,034,652	276,912,597	377,463,894	389,612,976	446,558,209	523,737,374	529,181,741	

Notes To The Unconsolidated Financial Statements

For the year ended December 31, 2020

	2020	2019
	(Rupees in '000)	
**Reconciliation to total assets**		
**Balance as per balance sheet**	1,757,462,470	1,515,152,015
Less: Non financial assets		
Fixed assets	58,027,904	58,271,245
Intangible assets	938,458	957,552
Other assets	10,066,501	11,334,247
	69,032,863	70,563,044
**Total financial assets**	1,688,429,607	1,444,588,971

	2020	2019
	(Rupees in '000)	
**Reconciliation to total assets**		
**Balance as per balance sheet**	1,567,360,515	1,346,237,232
Less: Non financial liabilities		
Other liabilities	22,169,672	17,577,954
Deferred tax liability	6,975,158	5,850,645
	29,144,830	23,428,599
**Total financial liabilities**	1,538,215,685	1,322,808,633

Yield risk is the risk of decline in earnings due to adverse movement of the yield curve.

Interest rate risk is the risk that the value of the financial instrument will fluctuate due to changes in the market interest rates.

45.3 Operational Risk

Operational risk is the risk of loss resulting from inadequate or failed internal processes, people, and systems or from external events. This definition includes legal risks but excludes strategic and reputational risks.

The Bank's operational risk management framework, as laid down in the Global Risk Management Policy, duly approved by BOD, is flexible enough to implement in stages and permits the overall risk management approach to evolve in the light of organizational learning and the future needs of the Bank. Operational loss events are reviewed and appropriate corrective actions taken on an ongoing basis, including measures to improve control procedures with respect to design and operative effectiveness.

Operational Risk Management helps the Bank understand risks and improve mitigating controls so as to minimize operational risks that are inherent in almost all areas of the Bank. Going forward, the Bank will further strengthen its risk function, policies and procedures to facilitate its operations and improve quality of assets to safeguard interest of depositors.

45.3.1 Operational Risk-Disclosures Basel II Specific

Currently, the bank is reporting operational risk capital charge under Basic Indicator Approach (BIA). The Bank took a number of initiative with respect to operational risk management like using Key Risk Indicators (Kris), Loss events database and Risk & Control Self Assessments (ROSA) to manage its operational risk effectively.

In accordance with the Operational Risk Management (OR) regulations, policy and framework, a database covering operational risk events is being maintained using a state of the art software solution, which has enhanced features and a better workflow management. This new software has further augmented bank's capacity to capture and report operational risk events and Kris. The software is also capable of generating periodical regulatory and management reports. Periodical updates on Operational Risk events are presented to senior management and the Risk Management and Portfolio Review Committee of the Board.

Notes To The Unconsolidated Financial Statements

For the year ended December 31, 2020

45.4 Liquidity Risk

Liquidity represents the ability to fund assets and meet obligations as they become due. The Bank understands that liquidity does not come for free, and surplus liquidity has an opportunity cost which needs to be recognized. Liquidity risk is a risk of not being able to obtain funds at a reasonable price within a reasonable time period to meet obligations as they become due. Liquidity is essential to the ability to operate financial services businesses and, therefore, the ability to maintain surplus levels of liquidity through economic cycles is crucial. Particularly during periods of adverse conditions, liquidity management is among the most important activities that the MCB conducts during both normal and stress periods. MCB recognizes that liquidity risk can arise from the Bank's activities and can be grouped into three categories:

- Inflows/Outflows from on-balance sheet items (other than marketable securities and wholesale borrowings) and off-balance sheet items;
- Marketability of trading securities; and
- Capacity to borrow from the wholesale markets for funding as well as trading activities.

Liquidity Management

The Asset Liability Management Committee of the bank has the responsibility for the formulation of overall strategy and oversight of the Asset Liability Management function. Board has approved a comprehensive Liquidity Risk Policy (part of Risk Management Policy), which stipulates policies regarding maintenance of various ratios, funding preferences, and evaluation of Banks' liquidity under normal and stress scenarios. A framework to assess the maturity profile of non-contractual assets and liabilities is in place to supplement the liquidity management. Bank's comprehensive liquidity management framework assists it to closely watch the liquidity position through monitoring of early warning indicators and stress testing, to ensure effective and timely decision making.

MCB's liquidity risk management framework is designed to identify measure and manage in a timely manner the liquidity risk position of the Bank. The underlying policies and procedures include: Global Risk Management policy, Global Treasury Policy, Investment policy, Contingency Funding Plan, Liquidity Strategy and Limit Structure which are reviewed and approved regularly by the senior management / Board members. Moreover; the Bank also prepares a 'Contingency Funding Plan' (CFP) to address liquidity issues in time of stress/crises situation containing early warning indicators to pre-empt unforeseen liquidity crises. MCB Bank conducts Liquidity Risk Analysis on regular basis as well as Maturity of gaps are also reviewed in order to ensure diversification in terms of tenors. MCB liquidity risk framework envisages to project the Bank's funding position during temporary and long-term liquidity changes, including those caused by liability erosion and explicitly identifying quantifying and ranking all sources of funding preferences, such as reducing assets, modifying or increasing liability structure; and using other alternatives for controlling statement of financial position changes. MCB performs regular liquidity stress tests as part of its liquidity monitoring activities. The purpose of the liquidity stress tests is intended to ensure sufficient liquidity for the Bank under both idiosyncratic and systemic market stress conditions. MCB's liquidity risk management approach involves intraday liquidity management, managing funding sources and evaluation of structural imbalances in balance sheet structure.

In view of the relaxation granted by SBP for deferral of principal and markup and for rescheduling / restructuring of loans there will be an impact on the maturity profile of the Bank. The Asset and Liability Committee (ALCO) of the Bank is monitoring the liquidity position and the Bank is confident that the liquidity buffer currently maintained is sufficient to cater to any adverse movement in the cash flow maturity profile.

Liquidity Management:

In view of the relaxation granted by SBP for deferral of principal and markup and for rescheduling / restructuring of loans there will be an impact on the maturity profile of the Bank. The Asset and Liability Committee (ALCO) of the Bank is monitoring the liquidity position and the Bank is confident that the liquidity buffer currently maintained is sufficient to cater to any adverse movement in the cash flow maturity profile

Intraday Liquidity Management

Intraday liquidity management is about managing the daily payments and cash flows. Bank has policies to ensure that sufficient cash is maintained during the day to make payments through local payment system. The policy of the Bank is to maintain adequate liquidity at all times, in all geographical locations and for all currencies and hence to be in a position, in the normal course of business, to meet obligations, repay depositors and fulfill commitments.

Managing Funding Sources

Managing funding sources, as per policy MCB maintain a portfolio of marketable securities that can either be sold outright or sold through a repurchase agreement to generate cash flows for meeting unexpected liquidity requirement. As a part of liquidity management MCB maintains borrowing relationships to ensure the continued access to diverse market of funding sources. MCB's sound credit rating together with excellent market reputation has enabled MCB to secure ample call lines with local and foreign banks. The level of liquidity reserves as per regulatory requirements also mitigates risks. MCB's investment in marketable securities is much higher than the Statutory Liquidity requirements.

45.4.1 Maturities of Assets and Liabilities - based on contractual maturity of the assets and liabilities of the Bank

	2020													
	Total	Upto 1 day	Over 1 to 7 days	Over 7 to 14 days	Over 14 days to 1 months	Over 1 or 2 months	Over 2 to 3 months	Over 3 to 6 months	Over 6 to 9 months	Over 9 months to 1 years	Over 1 to 2 years	Over 2 to 3 years	Over 3 to 5 years	Over 5 years
	(Rupees in '000)													
**Assets**														
Cash and balances with treasury banks	122,180,839	122,180,839	-	-	-	-	-	-	-	-	-	-	-	-
Balances with other banks	24,030,328	21,793,041	639,225	-	799,031	-	799,031	-	-	-	-	-	-	-
Lending to financial institutions	17,139,453	1,864,192	15,275,261	-	-	-	-	-	-	-	-	-	-	-
Investments	1,015,869,448	1,032,330	660,200	91,757,770	72,188,252	159,114,738	179,247,663	114,811,198	60,862,579	5,059,417	28,051,162	75,507,721	36,666,731	190,909,687
Advances	462,941,787	69,434,527	16,280,888	5,567,948	21,964,456	20,382,678	31,484,973	53,402,604	34,512,637	28,348,910	51,224,268	36,344,592	56,301,425	37,691,881
Fixed assets	58,027,904	7,799	46,800	54,601	218,707	327,908	327,908	983,725	983,725	983,725	3,528,643	3,562,702	4,097,577	42,904,084
Intangible assets	938,458	1,979	11,878	13,857	31,674	59,389	59,389	178,167	178,167	178,167	225,791	-	-	-
Deferred tax assets	2,782,530	7,623	45,740	53,364	129,597	221,078	236,324	693,727	701,350	693,727	-	-	-	-
Other assets	56,334,253	115,353	1,680,408	7,413,870	6,023,597	9,571,753	9,356,338	1,992,478	579,658	83,001	2,260,624	6,419,673	10,837,500	-
	1,760,245,000	216,437,683	34,640,400	104,861,410	101,355,314	189,677,544	221,511,626	172,061,899	97,818,116	35,346,947	85,290,488	121,834,688	107,903,233	271,505,652
**Liabilities**														
Bills payable	23,980,692	799,357	4,796,138	5,595,495	12,789,702	-	-	-	-	-	-	-	-	-
Borrowings	164,001,533	27,145,780	91,245,233	1,537,110	1,338,623	6,239,388	1,975,286	5,044,522	924,633	889,239	8,382,537	2,571,133	4,831,690	11,876,359
Deposits and other accounts	1,289,502,304	1,199,768,203	3,244,480	5,172,564	11,883,056	11,137,355	10,105,884	13,630,588	17,258,524	13,285,379	1,998,833	115,810	1,689,628	212,000
Deferred tax liabilities	9,757,688	33,174	35,167	30,030	3,668	127,642	20,372	85,781	726,634	196,315	655,206	1,911,154	2,455,143	3,477,402
Other liabilities	82,900,828	11,993,186	1,961,994	3,496,367	5,147,109	5,533,265	5,961,832	13,433,608	2,147,942	2,087,801	3,880,245	7,956,617	15,387,832	3,913,030
	1,570,143,045	1,239,739,700	101,283,012	15,831,566	31,162,158	23,037,650	18,063,374	32,194,499	21,057,733	16,458,734	14,916,821	12,554,714	24,364,293	19,478,791
**Net assets**	190,101,955	(1,023,302,017)	(66,642,612)	89,029,844	70,193,156	166,639,894	203,448,252	139,867,400	76,760,383	18,888,213	70,373,667	109,279,974	83,538,940	252,026,861
Share capital	11,850,600													
Reserves	80,696,335													
Surplus on revaluation of assets	27,720,418													
Unappropriated profit	69,834,602													
	190,101,955													

Notes To The Unconsolidated Financial Statements

For the year ended December 31, 2020

	2019													
	Total	Upto 1 day	Over 1 to 7 days	Over 7 to 14 days	Over 14 days to 1 months	Over 1 or 2 months	Over 2 to 3 months	Over 3 to 6 months	Over 6 to 9 months	Over 9 months to 1 years	Over 1 to 2 years	Over 2 to 3 years	Over 3 to 5 years	Over 5 years
	(Rupees in '000)													
**Assets**														
Cash and balances with treasury banks	132,704,797	132,704,797	-	-	-	-	-	-	-	-	-	-	-	-
Balances with other banks	12,542,239	8,879,536	541,582	1,083,933	696,814	1,340,374	-	-	-	-	-	-	-	-
Lending to financial institutions	1,090,058	209,206	-	-	880,852	-	-	-	-	-	-	-	-	-
Investments	748,764,502	523,435	12,011,605	1,395,543	165,746,557	4,146,532	38,619,156	37,874,254	101,658,052	85,835,490	109,566,556	17,164,625	64,933,046	109,289,651
Advances	496,678,874	112,864,577	10,898,866	9,633,175	33,566,678	22,988,288	27,545,204	55,225,422	25,931,037	35,055,991	42,508,022	34,030,023	46,800,437	39,631,154
Fixed assets	58,271,245	8,032	48,204	56,237	224,491	336,966	336,966	1,010,898	1,010,898	1,010,898	3,274,809	3,404,675	4,271,900	43,276,271
Intangible assets	957,552	1,717	10,296	12,012	27,456	51,480	51,480	154,441	154,441	154,441	301,022	38,766	-	-
Deferred tax assets	1,725,288	4,727	28,361	33,088	80,356	137,078	146,531	430,140	434,867	430,140	-	-	-	-
Other assets	64,142,748	189,805	3,239,882	7,594,076	7,376,794	12,172,177	13,452,420	3,216,119	3,261,953	172,314	1,214,143	2,141,963	10,111,102	-
	1,516,877,303	255,385,832	26,778,796	19,808,064	208,599,998	41,172,895	80,151,757	97,911,274	132,451,248	122,659,274	156,864,552	56,780,052	126,116,485	192,197,076
**Liabilities**														
Bills payable	11,821,698	394,056	2,364,340	2,758,396	6,304,906	-	-	-	-	-	-	-	-	-
Borrowings	89,505,892	19,597,099	23,761,935	5,906,627	5,599,571	7,526,054	2,467,108	7,185,921	664,824	802,705	2,097,344	2,217,657	3,572,934	8,106,113
Deposits and other accounts	1,144,763,259	1,036,080,008	2,388,779	7,366,606	10,153,502	12,241,878	22,863,939	22,527,727	15,917,007	8,491,623	3,459,232	2,002,175	1,270,783	-
Deferred tax liabilities	7,575,933	10,848	16,036	12,021	(35,127)	14,092	(101,995)	29,162	66,342	(28,156)	568,296	296,041	2,832,251	3,896,122
Other liabilities	94,295,738	9,808,328	7,271,888	6,967,559	16,492,957	5,507,468	5,832,693	10,851,954	5,079,603	2,003,742	2,425,495	3,420,466	13,756,886	4,876,699
	1,347,962,520	1,065,890,339	35,802,978	23,011,209	38,515,809	25,289,492	31,061,745	40,594,764	21,727,776	11,269,914	8,550,367	7,936,339	21,432,854	16,878,934
**Net assets**	168,914,783	(810,504,507)	(9,024,182)	(3,203,145)	170,084,189	15,883,403	49,090,012	57,316,510	110,723,472	111,389,360	148,314,185	48,843,713	104,683,631	175,318,142
Share capital	11,850,600													
Reserves	77,591,253													
Surplus on revaluation of assets	23,695,441													
Unappropriated profit	55,777,489													
	168,914,783													

Notes To The Unconsolidated Financial Statements

For the year ended December 31, 2020

45.4.2 Maturities of assets and liabilities - based on expected maturities of the assets and liabilities of the Bank

	2020									
	Total	Upto 1 month	Over 1 to 3 months	Over 3 to 6 months	Over 6 months to 1 year	Over 1 to 2 years	Over 2 to 3 years	Over 3 to 5 years	Over 5 to 10 years	Above 10 years
	(Rupees in '000)									
**Assets**										
Cash and balances with treasury banks	122,180,839	122,180,839	–	–	–	–	–	–	–	–
Balances with other banks	24,030,328	23,231,297	799,031	–	–	–	–	–	–	–
Lending to financial institutions	17,139,453	17,139,453	–	–	–	–	–	–	–	–
Investments	1,015,869,448	165,626,376	338,357,141	114,805,937	65,897,443	28,013,808	75,470,367	36,564,210	175,676,689	15,457,477
Advances	462,941,787	69,597,045	32,217,300	27,668,757	49,069,203	79,509,554	66,966,890	82,943,687	46,065,324	8,904,027
Fixed assets	58,027,904	327,909	655,816	983,725	1,967,449	3,528,643	3,562,702	4,046,673	6,266,060	36,688,927
Intangible assets	938,458	59,388	118,778	178,167	356,334	225,791	–	–	–	–
Deferred tax assets	2,782,530	236,324	457,402	693,727	1,395,077	–	–	–	–	–
Other assets	56,334,253	15,233,227	18,917,932	2,002,637	662,660	2,260,624	6,419,673	10,837,500	–	–
	1,760,245,000	413,631,858	391,523,400	146,332,950	119,348,166	113,538,420	152,419,632	134,392,070	228,008,073	61,050,431
**Liabilities**										
Bills payable	23,980,692	23,980,692	–	–	–	–	–	–	–	–
Borrowings	164,001,533	121,266,747	8,214,674	5,044,522	1,813,871	8,382,537	2,571,133	4,831,690	11,876,359	–
Deposits and other accounts	1,289,502,304	72,039,661	48,536,715	57,946,390	56,649,804	317,092,872	315,209,849	316,783,667	105,243,346	–
Deferred tax liabilities	9,757,688	103,420	147,968	85,470	921,877	654,883	1,910,831	2,432,012	1,711,329	1,789,898
Other liabilities	82,900,828	22,598,657	11,491,122	13,437,582	4,235,743	3,880,245	7,956,617	15,387,832	3,451,697	461,333
	1,570,143,045	239,989,177	68,390,479	76,513,964	63,621,295	330,010,537	327,648,430	339,435,201	122,282,731	2,251,231
**Net assets**	190,101,955	173,642,681	323,132,921	69,818,986	55,726,871	(216,472,117)	(175,228,798)	(205,043,131)	105,725,342	58,799,200
Share capital	11,850,600									
Reserves	80,696,335									
Surplus on revaluation of assets	27,720,418									
Unappropriated profit	69,834,602									
	190,101,955									

	2019									
	Total	Upto 1 month	Over 1 to 3 months	Over 3 to 6 months	Over 6 months to 1 year	Over 1 to 2 years	Over 2 to 3 years	Over 3 to 5 years	Over 5 to 10 years	Above 10 years
	(Rupees in '000)									
**Assets**										
Cash and balances with treasury banks	132,704,797	132,704,797	-	-	-	-	-	-	-	-
Balances with other banks	12,542,239	11,201,865	1,340,374	-	-	-	-	-	-	-
Lending to financial institutions	1,090,058	1,090,058	-	-	-	-	-	-	-	-
Investments	748,764,502	179,665,525	42,764,030	37,872,595	187,483,587	109,524,248	17,120,045	64,890,737	94,837,469	14,606,266
Advances	496,678,874	94,021,656	52,033,469	46,410,125	51,464,951	69,033,399	60,132,349	74,935,777	41,348,513	7,298,635
Fixed assets	58,271,245	336,965	577,984	1,106,846	2,021,795	3,274,809	2,124,651	5,551,924	6,047,543	37,228,728
Intangible assets	957,552	51,480	102,961	154,441	308,882	301,022	38,766	-	-	-
Deferred tax assets	1,725,288	146,532	283,609	430,140	865,007	-	-	-	-	-
Other assets	64,142,748	18,400,556	25,599,507	3,241,210	3,434,267	1,214,143	2,141,963	10,111,102	-	-
	1,516,877,303	437,619,434	122,701,934	89,215,357	245,578,489	183,347,621	81,557,774	155,489,540	142,233,525	59,133,629
**Liabilities**										
Bills payable	11,821,698	11,821,698	-	-	-	-	-	-	-	-
Borrowings	89,505,892	54,865,233	9,993,161	7,185,921	1,467,529	2,097,344	2,217,657	3,572,934	8,106,113	-
Deposits and other accounts	1,144,763,259	67,516,013	54,469,232	50,398,665	60,795,072	274,914,858	273,457,801	272,726,409	90,485,209	-
Deferred tax liabilities	7,575,933	3,780	(88,384)	28,760	37,225	568,296	295,246	2,832,251	2,577,062	1,321,697
Other liabilities	94,295,738	40,540,731	11,340,161	10,855,453	7,079,846	2,425,495	3,420,466	13,756,886	4,338,448	538,252
	1,347,962,520	174,747,455	75,714,170	68,468,799	69,379,672	280,005,993	279,391,170	292,888,480	105,506,832	1,859,949
**Net assets**	168,914,783	262,871,979	46,987,764	20,746,558	176,198,817	(96,658,372)	(197,833,396)	(137,398,940)	36,726,693	57,273,680
Share capital	11,850,600									
Reserves	77,591,253									
Surplus on revaluation of assets	23,695,441									
Unappropriated profit	55,777,489									
	168,914,783									

Liquidity Gap Reporting

When an asset or liability does not have any contractual maturity date, the period in which these are assumed to mature has been taken as the expected date of maturity. Bank regularly conducts an objective and systematic behavioral study using regression analysis technique to ascertain the maturity of its non-contractual assets and liabilities. Core and non-core parts of the non-contractual assets and liabilities are segregated through the behavioral study. Non Core part is placed among the short term maturity buckets i.e. up to 1 Year based on the model results, whereas core part is distributed among the longer terms buckets based on the discussion and decision by the ALCO. Following percentages are used to distribute the core assets and liabilities among longer term buckets:

Over 1 to 2 Years	Over 2 to 3 Years	Over 3 to 5 Years	Over 5 to 10 Years
30%	30%	30%	10%

Notes To The Unconsolidated Financial Statements

For the year ended December 31, 2020

45.5 Derivative Risk

Most business clients have either interest rate exposures arising from debt financing or currency exposures arising out of commercial transactions from import and export of goods. Businesses face the risk of sudden movements in interest rates or foreign exchange rates that may adversely affect their profitability. MCB Bank provides solutions to this problem through its derivatives desk in major types of derivative instruments i.e.; forwards, futures, swaps and options. As an Authorized Derivative Dealer (ADD), MCB is an active participant in Derivative market and has flexibility in providing a broad range of derivatives products covering both hedging and market making to satisfy customers' needs. As an ADD, the bank offers derivative products which are permitted under the Financial Derivative Business Regulations (FDBR) or as permitted by the State Bank of Pakistan. Before executing Derivative transactions, the bank ensures that the clients understand the risk and reward associated with the derivative being offered. Derivative transactions are executed with appropriate clients only.

Risk management activities take place at the following different levels.

Strategic Level:

By senior management Assets and Liabilities Management Committee (ALCO), Management Credit and Risk Committee (MC&RC) and the Board of Directors to institute a risk management framework and to ensure provision of all resources and support required for effective risk management on Bank-wide basis. The Board provides the overall limits/thresholds for derivatives business.

Macro Level

By Treasury and FX Group and Risk Management Group, responsible for policy formulation, procedure development and implementation, monitoring and reporting.

Micro Level:

Treasury Derivatives and Structured Product Desk where risks are actually created and Treasury Operations for settlements of the transactions.

Derivative Risk Management caters the following risks:-

Market Risk arises from changes in market rates such as Interest Rates, Foreign Exchange Rates, Equity Prices, credit spreads and/or commodity prices as well as their correlations and volatilities resulting in a loss to earnings and capital. In line with SBP's regulatory guidelines, Bank hedges back-to-back all option transactions with other financial institutions. Bank minimizes the exchange rate risk on its Cross Currency Swap Portfolio by hedging the exposure in interbank market. Bank also manages interest rate risk on its Interest Rate Derivatives and Cross Currency Swaps through various sensitivity limits approved by ALCO. Marked to market positions and sensitivity of the derivatives transactions are monitored on regular basis. All individual deals are approved at the appropriate level of authority after analyzing the risk and benefits associated with the deals.

Credit risk is a probable risk of loss resulting from customer's inability to meet contractual obligation that may have adverse impact on Bank's profitability. Bank manages the risk by setting policies and limits for counterparty based on a pre-defined criteria linked with financial health of the customer. The exposure of each counterparty is monitored by Risk Management Function of the Bank on daily basis.

Considering small Derivative portfolio, bank is not exposed to any liquidity risk. However; Bank manages its liquidity risk through Bank's liquidity risk framework which is defined in relevant Liquidity Risk Section.

Notes To The Unconsolidated Financial Statements
For the year ended December 31, 2020

Bank has adequate system and controls for smooth execution of derivative transactions. Transactions are executed in line with well defined accounting and operational aspects to mitigate the operational risk. Policies and control functions are regularly reviewed on periodic basis. The Bank uses a third party's Super Derivative System which provides front end sales and structuring capabilities, end to end valuation solutions, risk management systems, back end processing and provides analytical tools to measure various risk exposures and carry out sensitivity analysis.

The Bank uses a third party's Super Derivative System which provides front end sales and structuring capabilities, end to end valuation solutions, risk management systems, back end processing and provides analytical tools to measure various risk exposures and carry out sensitivity analysis.

The goal of asset/liability management (ALM) is to properly manage the risk related to changes in interest rates, the mix of balance sheet assets and liabilities, the holding of foreign currencies, and the use of derivatives. Due to thin liquidity in the derivative market, interest rate derivatives are not actively used to manage/alter the interest rate risk profile of the bank."

46 EVENTS AFTER THE REPORTING DATE

46.1 The Board of Directors in its meeting held on February 10, 2021 has announced a final cash dividend in respect of the year ended December 31, 2020 of Rs.15.00 per share (2019: Rs. 5.00 per share). These unconsolidated financial statements for the year ended December 31, 2020 do not include the effect of these appropriations which will be accounted for subsequent to the year end.

47 GENERAL

Figures have been rounded off to the nearest thousand of rupees unless otherwise stated.

48 DATE OF AUTHORIZATION FOR ISSUE

These unconsolidated financial statements were authorized for issue by the Board of Directors of the Bank in their meeting held on February 10, 2021.

**Imran Maqbool**	**Hammad Khalid**	**S.M. Muneer**	**Mian Umer Mansha**	**Shahzad Hussain**
President/Chief Executive	Chief Financial Officer	Director	Director	Director

Annexure I

Statement showing written-off loans or any other financial relief of five hundred thousand or above provided during the year 2020

Sr. No.	Name and address of the borrower	Name of individuals/ partners/ directors		Father's /Husband's name	Outstanding liabilities at beginning of year				Principal Written off	Interest/ Markup Written off/ waived	Other Fin. Reliefs Provided	Total
		Name	NIC No.		Principal	Interest / mark up	Others	Total				
**Domestic**												
1	**Saddique Rafique & Sons**<br>74-B Ghallah Mandi Vehari	Abdul Sattar	36603-2585282-7	Muhammad Saddique	–	467	56	523	–	467	56	523
2	**Shell Burewala Express**<br>Shop # 13-14, Gulshan E Rehman Multan Road Burewala.	Abdul Rasheed	36601-1589087-1	Abdul Hameed	–	1,175	46	1,221	–	1,175	46	1,221
3	**Amir & Co**<br>Islamia Colony Road, Noor e Wali Street, Rahim Yar Khan.	Muhammad Nadeem Aslam	31303-4864137-1	Haji Muhammad Aslam	–	888	50	938	–	888	50	938
4	**Azeem Exports (Pvt.) Ltd.**<br>202/RB Tanki Stop Ghona Road Faisalabad.	Mr. Ibrar Ahmed<br>Mrs. Yasmeen Ibrar<br>Mr. Naeem Akhtar	33100-0310806-9<br>33100-0336460-2<br>33100-6007043-8	Mushtaq Ahmed<br>Ibrar Ahmed<br>Ijaz Ahmed	–	29,234	80	29,314	–	29,234	80	29,314
5	**Mushtaq Ahmed Khan**<br>Chandni Chowk, Nawan Jandanwala, District Bhakkar	Mushtaq Ahmed Khan	38103-2239766-3	Ghulam Qadir	–	531	44	575	–	531	44	575
6	**Shahid Iqbal & Haji** Ghulam Rasool<br>1. 668-A S/Town Sargodha<br>2. 662-A S/Town Sargodha	Shahid Iqbal<br>Haji Ghulam Rasool	38403-6114219-7	Ch. Muhammad Iqbal<br>Haji Makhdoom<br>Muhammad Hussain	–	665	32	697	–	665	32	697
7	**SNS Textiles (Pvt) Ltd**<br>Head Office at 17-D-II, Green Town, Factory Area, Lahore	Muhammad Nadeem<br>Shahid Maqsood<br>Rizwana Nadeem<br>Sara Shahid	35202-9903037-1<br>35202-3442308-5<br>35202-6481919-2<br>35202-6509332-8	Inayat Ullah<br>M. Maqsood<br>M. Nadeem<br>Shahid Maqsood	–	29,619	3,371	32,990	–	29,619	3,371	32,990
8	**Muhammad Zulfiqar Arshad**<br>House no. 163 Block-M, DHA, Lahore	Muhammad Zulfiqar Arshad	35201-9336715-9	Arsahd Ali Rashid	–	4,203	–	4,203	–	4,203	–	4,203
9	**Amir Iftikhar**<br>House no. 364 Block-W, Phase-III,Defence Housing Authority, Lahore	Amir Iftikhar	35201-5066143-9	Sh. Iftikhar ud Din	–	3,742	19	3,761	–	3,742	19	3,761
10	**Gulzar Begum**<br>House no. bearing no. 78 Block C-2, PGEC Housing Society, Sattukatla, Lahore	Gulzar Begum	35202-1791054-6	Malik Muhammad Akram	–	971	–	971	–	971	–	971
11	**Muhammad Asif & Muhammad Yaseen**<br>House No 279 Muhallah / Block 13-B-1 Township Lahore	Muhammad Asif<br>Muhammad Yaseen	35202-4417428-9<br>35202-0937710-9	Muhammad Yaseen<br>Bashir Ahmed	–	1,570	75	1,645	–	1,570	75	1,645

Statement showing written-off loans or any other financial relief of five hundred thousand or above provided during the year 2020

Sr. No.	Name and address of the borrower	Name of individuals/ partners/ directors: Name	NIC No.	Father's /Husband's name	Outstanding liabilities at beginning of year: Principal	Interest / mark up	Others	Total	Principal Written off	Interest/ Markup Written off/ waived	Other Fin. Reliefs Provided	Total
**Domestic**												
12	**Ch. Aziz Ur Rehman & Mst. Rubina Aziz**<br>237 - D/II, Johar Town, Lahore	Ch. Aziz Ur Rehman<br>Mst. Rubina Aziz	35202-5462193-7<br>35202-1754926-2	Ch. Abdul Rehman<br>Ch. Aziz Ur Rehman	–	4,149	–	4,149	–	4,149	–	4,149
13	**Abdul Jawad Shami**<br>House No. 2, Block H, Gulshan Ravi, Lahore	Abdul Jawad Shami	35202-6499439-7	Abdul Saleem Shami	–	2,164	–	2,164	–	2,164	–	2,164
14	**Mazhar Hussain and Nasira Mazhar**<br>House No 5, Shershah Block, Garden Town, Lahore	Mazhar Hussain<br>Nasira Mazhar	35202-2997013-3 35202-2692430-0	Sheikh Muhammad<br>Hussain Mazhar Hussain	–	1,173	50	1,223	–	1,173	50	1,223
15	**Waseem Ahmad**<br>House # 7, Street # 8, Mohallah Qureshi, Mazang, Lahore	Waseem Ahmad	35202-8395241-9	Mushtaq Ahmad	–	1,016	–	1,016	–	1,016	–	1,016
16	**Madina Rice Mill**<br>G.T Road, Gakhar Mandi, Tehsil Wazirabad, District Gujranwala	Muhammad Aslam	34104-4289360-9	Muhammad Sagheer	–	5,450	–	5,450	–	5,450	–	5,450
17	**Muhammad Shakir**<br>House No. 140/2, Sector 5-F,<br>New Karachi, Karachi	Muhammad Shakir	42101-4630058-7	Liaquat Shaikh Qureshi	–	815	35	850	–	815	35	850
18	**Muhammad Tanvir Mirza Rehmani**<br>House No. 140, 1st Floor, Block 18, Gulshan -E Iqbal, Karachi	Muhammad Tanvir Mirza Rehmani	42201-2527934-5	Muhammad Iftikhar Mirza Rehmani	–	855	–	855	–	855	–	855
19	**Muhammad Naeem Umar**<br>House No. 2-A,4/27, Nazimabad # 02, Karachi.	Muhammad Naeem Umar	42101-2173377-3	Muhammad Umar	–	1,843	–	1,843	–	1,843	–	1,843
20	**Abdul Waheed**<br>Flat No. 308, 3rd Floor, Block-A, Mariam Palace, Kharadar Karachi.	Abdul Waheed	42301-8963441-5	Muhammad Siddique	–	600	–	600	–	600	–	600
21	**Suroor Maira Apparel (Pvt) Ltd.**<br>House # 33/2, Street 29, Phase V, DHA, Karachi	Zahir Rahimtoola<br>Assad Ullah Khan<br>Sherezad Rahimtoola	42000-0477988-9<br>42301-8897950-1<br>42301-0936325-2	Mehmoob Rahimtoola<br>Mansoor Hussain<br>Zahir Rahimtoola	–	2,419	–	2,419	–	2,419	–	2,419
22	**Lable Garments & Access. (Pvt) Ltd.**<br>House # 33/2, Street 29, Phase V, DHA, Karachi	Zahir Rahimtoola<br>Assad Ullah Khan<br>Sherezad Rahimtoola<br>Daulat Rahimtoola	42000-0477988-9<br>42301-8897950-1<br>42301-0936325-2<br>42000-2438892-4	Mehmoob Rahimtoola<br>Mansoor Hussain<br>Zahir Rahimtoola	–	734	–	734	–	734	–	734
23	**Qureshi Netwear Industries**<br>788 Murad Pure New Marala Road Sialkot	Muhammad Jamil Ahmed Qureshi	37403-0669367-1	Muhammad Ramzan	–	3,544	17	3,561	–	3,544	17	3,561

Annexure I

Statement showing written-off loans or any other financial relief of five hundred thousand or above provided during the year 2020

Sr. No.	Name and address of the borrower	Name of individuals/ partners/ directors		Father's /Husband's name	Outstanding liabilities at beginning of year				Principal Written off	Interest/ Markup Written off/ waived	Other Fin. Reliefs Provided	Total
		Name	NIC No.		Principal	Interest / mark up	Others	Total				
**Domestic**												
24	**To To Sweet & Food Corporation** Mohallah Farooq Nagar, Kachi abadi, Near girls Primary School, Sheikhupura.	Shahid Parvaiz	35404-4242532-9	Asghar Ali	–	1,069	–	1,069	–	1,069	–	1,069
25	**Rai Shadat And Co** Ghalla Mandi, Jalalpur Bhattian	Rai Shadat Khan	34302-1215157-3	Rai Sultan Mehmood	–	1,003	–	1,003	–	1,003	–	1,003
26	**Zohaib Rice Mills** Ikhlas pur Road, Shakar Garh, Narowal	Malik Niaz Ahmad	34502-6305208-3	Islam Din	–	1,685	–	1,685	–	1,685	–	1,685
27	**Hassan Rice Mills** Gujranwala Road Kot Rehar Dist Gujranwala Tehsil Wazirabad	Hassan Asghar	34101-2676637-5	Muhammad Asghar	–	599	–	599	–	599	–	599
28	**Mumtaz Traders** Shopping Center Nangi Mirpur	Muhammad Mumtaz Ch.	81302-1707251-7	Muhammad Zaman	–	1,063	–	1,063	–	1,063	–	1,063
29	**Mughal Traders** House No.03, Satiana Banglow Jaranwala Road, Faisalabad	Muhmmad Rafi Ul Zaman	33104-2126873-5	Abdul Ghafoor	–	453	52	505	–	453	52	505
30	**Muhammad Awias** Sadiq Plaza 3rd floor Room No.12, near Akkas Photo Studio Katchery Bazar Faislabad	Muhammad Awais	33100-8174477-3	Muhammad Younas	–	8,960	102	9,062	–	8,960	102	9,062
31	**J.W Gases** B-89 Commercial Centre Gulistan Colony, Faisalabad.	Muhammad Waseem	33100-4224474-0	Malik Wazir Ali	–	1,605	53	1,658	–	1,605	53	1,658
32	**Ahmed Brothers** Shop No. 01, Trust Plaza, Fatima Jinnah Road, Sargodha	Khalid Khursheed Piracha	38403-3107227-9	Khursheed Ahmed Piracha	–	945	83	1,028	–	945	83	1,028
33	**Umer Hayat** Village & P.O Rasool Pur Tarar, District Hafizabad.	Umer Hayat	34302-9618143-5	Imtiaz Ahmed	–	1,996	77	2,073	–	1,996	77	2,073
34	**Zaib Traders Rice Dealer** Railway Road Baddo Malhi, Tehsil & District Narowal.	Muhammad Aslam	34501-4606789-1	Akbar Ali	–	1,958	61	2,019	–	1,958	61	2,019
35	**Millenium Securities & Investments (Pvt) Ltd.** Office No. 01, 92-East, 3rd Floor, Razia Sharif Plaza, Fazl-ul-Haq Road, Blue Area, Islamabad	Ahsan Imtiaz Bhatti<br>Azhar Imtiaz Bhatti<br>Athar Imtiaz Bhatti<br>Tahira Imtiaz Bhatti<br>Ayesa Ehsan	61101-0105948-7<br>61101-8043568-9<br>61101-1964243-1<br>61101-1186037-2<br>35202-2557738-6	Imtiaz Ahmed Bhatti<br>Imtiaz Ahmed Bhatti<br>Imtiaz Ahmed Bhatti<br>Imtiaz Ahmed Bhatti<br>Ahsan Anwar	47,260	74,713	3	121,976	–	74,093	–	74,093

Statement showing written-off loans or any other financial relief of five hundred thousand or above provided during the year 2020

Sr. No.	Name and address of the borrower	Name of individuals/ partners/ directors		Father's /Husband's name	Outstanding liabilities at beginning of year				Principal Written off	Interest/ Markup Written off/ waived	Other Fin. Reliefs Provided	Total
		Name	NIC No.		Principal	Interest / mark up	Others	Total				
Domestic												
36	**D.G Flour Mills**<br>Mouza Jhoke Yar Shah Taunsa Road D.G Khan.	Muhammad Zaffar Ullah Khan Ghalzai<br>Khan Dur Muhammad Khan Ghalzai	32102-1795936-9<br>36302-0324205-3	Muhammad Aslam Khan Ghalzai<br>Khan Noor Muhammad Khan	–	2,532	66	2,598	–	2,532	66	2,598
37	**Khawaja Brothers Brokers**<br>House No.549 Block G Shah Rukne Alam Colony Multan.	Shahid Latif (Deceased)<br>Mst. Rukhsana Zaka (Deceased)<br>Mst.Kiran Shahid<br>Arskan Shahid<br>Mst. Sadaf Shahid<br>Mst. Kanwal Shahid<br>Waqas Shahid	36302-1757105-1<br>36302-9437571-2<br>36302-0590964-6<br>36302-2059711-9<br>36302-9533426-2<br>36302-9522976-2<br>36302-5108178-7	Khawaja Abdul Latif<br>W/o Khawaja Shahid Latif<br>D/o Khawaja Shahid Latif<br>S/o Khawaja Shahid Latif<br>D/o Khawaja Shahid Latif<br>D/o Khawaja Shahid Latif<br>S/o Khawaja Shahid Latif	–	2,742	17	2,759	–	2,742	17	2,759
38	**Changwani Cotton Ginners**<br>Jampur Road,Basti Mari ,Mauza Paiga Tehsil & District D.G Khan.	Malik Rashid Ahmad<br>Maqbool Ahmad<br>Zaffar Iqbal<br>Mst. Parveen Bibi<br>Saeed Ahmad<br>Malik Allah Baksh<br>Malik Bashir Ahmad<br>Ghulam Fared	32102-5968852-5<br>32102-0934213-3<br>32102-0934215-5<br>32102-0899191-2<br>30962-396138-0<br>30999-2834110-0<br>32102-7360864-7<br>32102-3933414-1	Qadir Baksh<br>Malik Baksh<br>Maqbool Ahmad<br>W/o Zaffar Iqbal<br>Haji Sher Muhammad<br>Haji Sher Muhammad<br>Haji Sher Muhammad<br>Haji Sher Muhammad	–	621	100	721	–	621	100	721
39	**Gold Star Oil Mills**<br>Sher Shah Road Bye Pass Multan.	Khawaja Muhammad Ehsan Ul Haq<br>Khawaja Muhammad Imdad Ul Haq<br>Khawaja Muhammad Ikram Ul Haq<br>Khawaja Muhammad Inam Ul Haq<br>Mst. Musarat Bibi	36302-2475854-5<br>36302-7301653-3<br>36302-0364961-7<br>36302-6265395-5<br>36302-0330915-4	Khawaja Muhammad Afzal Haq<br>Khawaja Muhammad Afzal Haq<br>Khawaja Muhammad Afzal Haq<br>Khawaja Muhammad Afzal Haq<br>Khawaja Muhammad Afzal Haq	–	18,840	16	18,856	–	18,840	16	18,856
40	**Mian Brothers**<br>Jalla Mor, Tehsil Dunyapur, District Lodhran	Mian Hamid Raza Shakir	36201-1503143-1	Mian Allah Ditta	118	441	75	634	–	440	75	515
41	**Ghulam Mohyuddin & Sons**<br>65 G.T. Road, Hide Market, Lahore	Sheikh Ghulam Mohyuddin Sethi<br>Sheikh Moeen Mohyuddin Sethi	35202-3577613-1<br>35202-6249148-5	Muhammad Lateef Sethi<br>Sheikh Ghulam Mohyuddin Sethi	–	2,931	–	2,931	–	2,931	–	2,931
42	**Sohail Sarfraz Etc**<br>House # 26-L, Gulberg-II, Lahore	Sohail Sarfraz<br>Sunil Sarfraz<br>Naghmana Sarfraz	35202-2518098-3<br>35202-2400089-4<br>35202-2518088-7	Sarfraz Ahmed	–	1,950	17	1,967	–	1,950	17	1,967
43	**Saqib Jamshaid Dar & Sarwar Saeeda**<br>House No. 5, Race Course Road, Near Post Office Shadman Lahore.	Saqib Jamshaid Dar<br>Sarwar Saeeda	35202-1774555-3<br>35202-1604635-4	Muhammad Jamshed Dar	–	2,336	75	2,411	–	2,411	–	2,411

Annexure I

Statement showing written-off loans or any other financial relief of five hundred thousand or above provided during the year 2020

Sr. No.	Name and address of the borrower	Name of individuals/ partners/ directors		Father's /Husband's name	Outstanding liabilities at beginning of year				Principal Written off	Interest/ Markup Written off/ waived	Other Fin. Reliefs Provided	Total
		Name	NIC No.		Principal	Interest / mark up	Others	Total				
**Domestic**												
44	**Ali Adnan Munir & Munir Ahmad** House No.817 Block-A Sabzazar Colony, Multan Road, Lahore	Ali Adnan Munir Munir Ahmad Ch"	35202-2496025-1 35202-2776934-9	Munir Ahmed Ch Ch. Muhammad Ibrahim	–	876	70	946	–	876	70	946
45	**Muhammad Ashfaq** House No. P-511, St No. 12, Mohallah Sarfraz Colony, Raja Wala, Faisalabad	Muhammad Ashfaq	33100-7847086-5	Abdul Sattar	–	2,912	67	2,979	–	2,912	67	2,979
46	**IMTIAZ ALI RAJPER** House No. 12-C/9, KDA Scheme No. 7, Chandni Chowk, Main University Road, Karachi	Imtiaz Ali Rajper	42201-4050361-5	Shah Muhammad	–	711	–	711	–	711	–	711
47	**Muhammad Hanif Liaquat** Hosue# 29, Ibrahim Allah Rakhan Building	Muhammad Hanif Liaquat	-	-	140	425	–	565	140	425	–	565
48	**Ahmed Bilal Awan** H.No. 2/536 Street N O.07 Gurunanak Puran wala Lahore	Ahmed Bilal Awan	35200-1452001-5	Ghulam Rasul Awan	324	713	–	1,037	324	713	–	1,037
49	**Khurram Rafi** Hosue 5 Muslim Street, Baghbanpura, Lahore	Khurram Rafi	35201-9921896-1	Mian Muhammad Rafi	250	385	–	635	250	385	–	635
50	**Aatif Mushtaq** House No.21,St No.4 Quaid-E-Azam Colony Karachi	Aatif Mushtaq	37405-2174893-5	Mushtaq Ahmed Anjum	284	364	–	648	284	364	–	648
51	**Sohail Shahabuddin Meghani** 7 Star Lodges, Near Agha Khan Jamatkhana, Garden E St, Karachi"	Sohail Shahabuddin Meghani	42201-6868522-3	Shahabuddin Meghani	275	318	–	593	275	318	–	593
52	**Changaz Ali Khan** Flat No. A 25 , Rofi App 13D 2, Gulshan E Iqbal, Karachi	Changaz Ali Khan	41304-0248214-9	Muhammad Ali Khan	244	281	–	525	244	281	–	525
53	**Muhammad Atif Riaz** Scheme#2. Shalimar Link House No 1 Ali Park, Lahore."	"Muhammad Atif Raiz Muhammad Raiz Parveen Raiz"	35202-3192609-7 35202-8211659-9 35202-9858708-8	Haji Muhammad Riaz Noor Muhammad Muhammad Raiz	–	793	17	810	–	793	17	810
54	**Badar Ali** House # 2, Street # 7, Flemming Road, Lahore	Badar Ali	35202-9994444-1	Muhammad Asif	–	1,120	60	1,180	–	1,120	60	1,180
55	**Shakib Pasha** 56 Sikandar Block, Allama Iqbal Town, Lahore.	Shakib Pasha	35202-3006115-1	Zulifqar Haider	–	1,071	17	1,088	–	1,071	17	1,088

Annexure I

Statement showing written-off loans or any other financial relief of five hundred thousand or above provided during the year 2020

Sr. No.	Name and address of the borrower	Name of individuals/ partners/ directors		Father's /Husband's name	Outstanding liabilities at beginning of year				Principal Written off	Interest/ Markup Written off/ waived	Other Fin. Reliefs Provided	Total
		Name	NIC No.		Principal	Interest / mark up	Others	Total				
**Domestic**												
56	**Muhammad Ilyas**<br>Old Sabzi Mandi<br>Rehmania Road Street<br>No.1 Faisalabad	Muhammad Ilyas	33100-2993303-3	Ghulam Muhammad	–	479	39	518	–	479	39	518
57	**Abdul Sattar**<br>House no. 5, Street 6,<br>Block 25, Sargodha	Abdul Sattar	38403-9161790-1	Ali Muhammad	110	815	94	1,019	–	815	94	909
58	**National Filling Point**<br>Petrol Pump at Babar<br>Road, District Council<br>Sargodha	Azhar Ul Haque Sheikh	38403-2070079-5	Fazal Haq	–	685	69	754	–	685	69	754
59	**Zeenat Tea Company**<br>Room No. 13, Suleman<br>Plaza, Ashraf Road,<br>Peshawar City"	Shahzad Arif	17301-5095424-1	Abdul Ghani	1,000	843	–	1,843	–	749	–	749
60	**Ghousia Traders**<br>Adda Machiwal, Burewala<br>Road, Vehari."	Rana Abdul Aziz Khan	36603-1391142-1	Rana Taj Muhammad	–	1,045	50	1,095	–	1,045	50	1,095
61	**Muhammad Aslam Khan**<br>(Staff Finance), Flat<br>N.18/8-E,Sector I-8/I<br>Islamabad.	Muhammad Aslam Khan	61101-2205616-9	Muhammad Ameen Khan	–	704	16	720	–	704	16	720
62	**Ahmad Traders**<br>Ward No.9 street Masjid<br>Embli Wali Jatoi District<br>Muzaffargarh.	Rao Shafqat Ali	32302-5901986-7	Liaquat Ali	–	1,122	59	1,181	–	1,122	59	1,181
63	**Indus Ginners**<br>Indus Cotton Factory<br>JamPur Road, Rajanpur	Amna Tabbasum	32403-9050100-6	Sheikh Abdul Sattar	–	1,666	17	1,683	–	1,666	17	1,683
64	**Sajid Textile Industries Limited**<br>Plot # 6 Sector 12-A<br>North karachi Ind Area<br>Karachi	Sajid Maqbool<br>Zahid Maqbool<br>Farrukh Maqbool<br>Khurram Maqbool	42101-8642463-9<br>42101-3632846-3<br>42101-3546491-3<br>42101-1766790-5	Maqbool Ahmed<br>Maqbool Ahmed<br>Maqbool Ahmed<br>Maqbool Ahmed	80,599	121,140	–	201,739	80,599	121,140	–	201,739
65	**Benzer Apparels**<br>Plot 8/1-C, Street<br>5,Sector 12-C,North<br>Karachi Township Karachi	Muhammad Iqbal Jarullah<br>Muhammad Irfan Jaraullah<br>Ahtesham Ahmed	42000-0562364-9<br>42000-0562342-3<br>42201-0754459-9	Sheikh Muhammad Jarullah<br>Muhammad Iqbal Jarullah<br>Muhammad Iqbal Jarullah	–	42,323	350	42,673	–	42,323	350	42,673
66	**Four Star Packages**<br>Cl-38, Sector 6-B, North<br>Karachi Industrial Area,<br>Karachi	Jamil Ahmed	42101-1832155-9	Wali Mohammad	–	1,520	–	1,520	–	1,520	–	1,520
67	**Alpha Tex**<br>House No.C-39, Block-6,<br>Gulshan-e-Iqbal<br>Karachi"	Tasneem Javed	42201-9464461-2	Javed Akhtar	–	12,869	143	13,012	–	12,869	143	13,012

Annexure I

Statement showing written-off loans or any other financial relief of five hundred thousand or above provided during the year 2020

Sr. No.	Name and address of the borrower	Name of individuals/ partners/ directors		Father's /Husband's name	Outstanding liabilities at beginning of year				Principal Written off	Interest/ Markup Written off/ waived	Other Fin. Reliefs Provided	Total
		Name	NIC No.		Principal	Interest / mark up	Others	Total				
**Domestic**												
68	**Tharparkar Sugar Mills Limited**<br>14-C, 5th Commercial Lane Zamzama Boulevard, Phase V, Defence Housing Authority Karachi	Imtiaz Ali Shah<br>Irfan Ali Shah<br>Irshad Ali Shah<br>Syed Shabahat Khan<br>Waheed Murad<br>Mohammad Saleem	44103-2975179-7<br>44103-4745570-5<br>42501-5421949-9<br>42201-8154350-3<br>44103-3462014-1<br>44103-9761187-1	Ghulam Haider Shah<br>Ghulam Haider Shah<br>Ghulam Haider Shah<br>Syed Wajahat Hussain<br>Mitho Khan<br>Jam	–	314,215	35	314,250	–	314,215	35	314,250
69	**Asia Motor Company**<br>G-32, Ruby Plaza,Preedy Street, Karachi	Muhammad Saleem Varind	42101-5401777-7	Dawood Varind	–	7,135	130	7,265	–	7,135	130	7,265
70	**CNC Electronics**<br>Shop no. 2 SB. 5m, Kehkashan Complex, B1, 13-C Gulshan-e-iqbal Karachi	Shahab Rahseed Khan	42201-2852589-3	Abdul Rasheed Khan	–	7,135	–	7,135	–	7,135	–	7,135
71	**Taha International Trading**<br>Shop no. 2 SB. 5m, Kehkashan Complex, B1, 13-C Gulshan-e-iqbal Karachi	Shahab Rahseed Khan	42201-2852589-3	Abdul Rasheed Khan	–	17,301	–	17,301	–	17,301	–	17,301
72	**Imdad Commission Shop**<br>Kassisey Road, Ghallah Mandi Jalalpur Bhattian	Imdad Ullah	34302-1211858-3	Muhammad Hayat	204	598	17	819	–	553	17	570
73	**Ch. Ammad Hassan Darogha**<br>Mohallad Darogha wala, Khyali Shahpur, Gujranwala.	Ch. Ammad Hassan Darogha	34101-6747667-1	Ch. Hassan Ali	–	715	25	740	–	715	25	740
74	**Allah -o-Akbar Petroleum**<br>Chak No.420/GB Jhok Burhan Tehsil Tandlianwala District Faisalabad.	Chaudhry Ghulam Nabi Shahid<br>Faisal Rasheed	33106-2013848-7<br>244-81-472816	Ch. Muhammad Deen<br>Ahmed Din	2,941	1,521	68	4,530	–	1,542	68	1,610
75	**Muhammad Saleem**<br>House No. NW06 (NW 2 Old) Govt Degree College Road, Mohalla Raja Sultan, Asghar Mall Rawalpindi	Muhammad Saleem	37405-9435622-7	Ghulam Farooq	8,141	3,776	15	11,932	–	3,841	15	3,856
76	**Hakimullah & Co.**<br>Khungi, Tehsil Timergara. Distt Dir (Lower)"	Attiullah<br>Muhammad Ali Johar (Late)	15302-8069659-5<br>15302-4054631-7	Haji Karimullah<br>Hakimullah	419	2,663	994	4,076	–	2,664	994	3,658
77	**Qamar Ul Islam**<br>House No. 169, ST. Mohalla Saidpur Scheme-2, Rawalpindi	Qamar ul Islam	37405-0502007-1	Jamil Ahmed	–	5,192	–	5,192	–	5,192	–	5,192

Annexure I

Statement showing written-off loans or any other financial relief of five hundred thousand or above provided during the year 2020

Sr. No.	Name and address of the borrower	Name of individuals/ partners/ directors		Father's /Husband's name	Outstanding liabilities at beginning of year				Principal Written off	Interest/ Markup Written off/ waived	Other Fin. Reliefs Provided	Total
		Name	NIC No.		Principal	Interest / mark up	Others	Total				
**Domestic**												
78	**Mian Mehmood Ahmad & Sons**<br>Shop # 159 New Ghallah Mandi Multan."	Mehmood Ahmad	35102-0624116-7	Abdul Majeed	800	990	15	1,805	–	993	15	1,008
79	**Muhammad Azam Muhammad Naeem**<br>Shop No. 38-C Ghallah Mandi Haroonabad District Bahawalnagar.	Muhammad Azam<br>Muhammad Naeem	31104-9134472-9	Muhammad Ismail	1,944	514	60	2,518	–	520	60	580
80	**Mazhar Hussain**<br>Village Nanowali, Tehsil & Distt. Sialkot.	Mazhar Hussain	34603-2287791-7	Muhammad Shafi	–	709	57	766	–	709	57	766
81	**Zahid Rice Traders R/o Manzoorabad PO Khas,**<br>Wazirabad, District Gujranwala "	Zahid Iqbal Cheema	34104-2219080-9	Muhammad Malik Cheema	290	1,380	119	1,789	–	1,383	119	1,502
82	**Al-Madinah Plastic Pvt. Ltd.**<br>F-4 Main Maripur Road, Site Area Karchi"	Fida Hussain Nayani<br>Ghulam Abbass Nayani<br>Mushtaq Hussain Nayani<br>Zahid Hussain Nayani	42301-1813517-1<br>42201-4107366-3<br>42201-9775332-1<br>42301-7297089-7	Wazir Ali Nayani<br>Wazir Ali Nayani<br>Wazir Ali Nayani<br>Wazir Ali Nayani	–	16,749	–	16,749	–	16,749	–	16,749
83	**Silver Textile Factory**<br>Plot No.D-28, Sector 12-A, North Karachi Industrial Area, Karachi	Syed Ahmed Ali<br>Syed Owais Ali<br>Syed Usman Ali<br>Syed Yaqoob Ali	42101-3971892-7<br>42101-4627666-3<br>42101-7642737-7<br>42101-8095918-5	Syed Raza Ali<br>Syed Raza Ali<br>Syed Raza Ali<br>Syed Raza Ali	–	1,637	–	1,637	–	1,637	–	1,637
84	**Muhammad Zia ul Haq**<br>House No. 589, Block F/2, M.A. Johar Town, Lahore	Muhammad Zia ul Haq	35202-2363794-3	Muhammad Ikram ul Haq Chaudhry	5,863	4,744	19	10,626	–	4,447	19	4,466
85	**Yawar Hanif**<br>House # I-A/C-I Block G, Gulberg III Lahore	Yawar Hanif Mian<br>Riffat Munawar<br>Nuzhat Hanif	35202-7634724-1<br>35202-7544977-2<br>35202-7554572-2	Muhammad Hanif Mian<br>Mian Munwar Ghafoor<br>Muhammad Hanif Mian	–	4,847	19	4,866	–	4,847	19	4,866
86	**Zohaib Traders**<br>House no. 65-A, Dilkusha Colony, Kot Lakhpat, Lahore.	Muhammad Zohaib	35202-3037668-9	Dilshad Ahmad	–	1,292	117	1,409	–	1,292	117	1,409
87	**Khalid Ishaq Re-Rolling Mills**<br>Lakhodair Road, Muhalla Iqbal Nagar, Shalamar Town, Lahore	"Imran Hussain<br>Irfan Hussain"	35202-2544005-9	Sh. Muhammad Ali<br>Sh. Muhammad Ali	–	543	35	578	–	543	35	578
88	**Saeed Ahmad**<br>F-215, 2nd floor, Block C, Sea World Clifton, Karachi	Saeed Ahmad	42301-6875199-3	Muhammad Annayat Ullah	334	897	–	1,231	–	899	–	899
89	**Muhammad Nazim**<br>House # 159B, Street 8-C, Feroz Colony Out Fall Road, Sanat Nagar, Lahore	Muhammad Nazim	35202-0236163-3	Haji Muhammad Yaqoob	538	1,180	–	1,718	–	1,155	–	1,155

Annexure I

Statement showing written-off loans or any other financial relief of five hundred thousand or above provided during the year 2020

Sr. No.	Name and address of the borrower	Name of individuals/ partners/ directors		Father's /Husband's name	Outstanding liabilities at beginning of year				Principal Written off	Interest/ Markup Written off/ waived	Other Fin. Reliefs Provided	Total
		Name	NIC No.		Principal	Interest / mark up	Others	Total				
**Domestic**												
90	**Al-Rehmat Enterprises** Jinnah Road, Back side, Islamia College, Gujranwala	Mian Waheed Ud din	34101-7252734-3	Rehmat Ali	2,000	2,632	–	4,632	–	2,654	–	2,654
91	**Ali Sabir** 38-A, Canal View Housing Society, Lahore	Ali Sabir	35202-2497389-3	Muhammad Rashid Khan	–	2,824	–	2,824	–	2,824	–	2,824
92	**Nuzhat iftikhar** 06-Zikria Town, Bosan Road, Multan	Nuzhat Iftikhar	37405-6871157-4	Sardar Iftikhar Ahmad	262	927	–	1,189	–	928	–	928
93	**Al Syed Enterprises** Pindi Bhattian Road Sadwan Tarar Dist. Hafizabad	Syed Sajjad Ali (Late)	34302-8603269-3	Syed Anwar Shah	–	871	74	945	–	871	74	945
94	**Ikram Ullah** Village Jagganwala, P.O Jharianwala, District Hafizabad.	Ikram Ullah	34301-1699085-7	Muhammad Idrees	558	842	47	1,447	–	808	47	855
95	**Shan Fabrics** House no. P-212 Street No.06 Mohalla Garden Colony Faisalabad.	Muhammad Iqbal	33100-6783237-9	Sheikh Allah Ditta	1,529	1,613	95	3,237	–	1,601	95	1,696
96	**Muhammad Zafar Ullah** House No. P-140 Street No.10, Ahmedabad Faisalabad	Muhammad Zafar Ullah	33100-7318037-7	Sher Muhammad	2,951	1,978	67	4,996	–	1,974	67	2,041
97	**Tariq Irfan Khan** House No. P-48, New Civil Lines Faisalabad.	Tariq Irfan Khan	33100-7220740-7	Muhammad Khan	–	6,022	67	6,089	–	6,022	67	6,089
98	**Al Rehman Kinno** Grading Factory Chak No. 06-B Tehsil Bhalwal, Distt Sargodha	Mehar Muhammad Zaman	38401-8528298-9	Mehar Ghulam Hussain	–	9,261	67	9,328	–	9,261	67	9,328
99	**Pak Rice Mills** Rana Tanveer Road, Nangle Kaswala, Muridkey.	Muhammad Arshad	35401-6606289-1	Muhammad Nazir	–	711	17	728	–	711	17	728
100	**Ch. Asif Mahmood** Ghalla Mandi Warburton, Nankana Sahib	Ch. Asif Mahmood	35501-0106412-9	Abdul Ghani	–	538	–	538	–	538	–	538
101	**M. Ahmad Garments** & Dildar Ahmed Near Darbar Allah Hoo & MashaAllah Furniture Jiya Moosa, Shahdra, Lahore	Dildar Ahmad	35202-9691226-3	Gulzar Ahmed	–	772	17	789	–	772	17	789
102	**Good Life Shoes** 347-GT Road, Near Moman Pura Lahore	Muhammad Akram Anjum	35201-6313040-9	Muhammad Ali	–	564	102	666	–	564	102	666

Annexure I

Statement showing written-off loans or any other financial relief of five hundred thousand or above provided during the year 2020

Sr. No.	Name and address of the borrower	Name of individuals/ partners/ directors		Father's /Husband's name	Outstanding liabilities at beginning of year				Principal Written off	Interest/ Markup Written off/ waived	Other Fin. Reliefs Provided	Total
		Name	NIC No.		Principal	Interest / mark up	Others	Total				
**Domestic**												
103	**Kashif Construction Company**<br>18-Infantry Road,<br>Mustafabad, Lahore.	Manzoor Kashif	35201-1651678-5	Muhammad Hussain	–	1,205	17	1,222	–	1,205	17	1,222
104	**Aslam Tractor House Rehman Pura G.T,**<br>Road Muridke District Sheikhpura	"Muhammad Aslam<br>Mirza Akbar Ali"	35401-7733223-9<br>35401-7738223-9	Muhammad Hussain<br>Muhammad Hussain	–	822	–	822	–	822	–	822
105	**Saleem Ahmed Riaz**<br>House No. 7, Block-C,<br>Model Town Society,<br>Lahore	Saleem Ahmed Riaz	35202-4482042-5	H. M Ahmed Riaz	–	19,716	66	19,782	–	19,716	66	19,782
106	**Sajid & Co**<br>G Block, Sabzazar<br>Scheme, Lahore.	Sajid Mahmood	35202-8752556-9	Mahmood ahmad	–	898	–	898	–	898	–	898
107	**Sardar Zari Corporation**<br>P/o Khas Thingi Colony,<br>Chak No. 76 W/B, Tehsil<br>& District Vehari.	"Mahmood Nasir<br>Roshan Din"	"36603-8632447-5<br>36603-3724902-7"	"Sardar Muhammad<br>Chiragh Din"	200	610	17	827	–	561	17	578
108	**The Farmers Group**<br>Shop # 12 Masjid Ghallah<br>Mandi Vehari.	Husnain Ahmad	36603-5763044-7	Ahmad Yar	–	1,195	41	1,236	–	1,195	41	1,236
109	**Kiyani Business Channel**<br>Shop # 13, Ghallah<br>Mandi Vehari Market.	Muhammad Nadeem<br>Kiyani	36603-1457627-3	Raja Meer Aslam	–	607	42	649	–	607	42	649
110	**Al-Hafiz Flour Mills**<br>Qadirpur Rawan Multan.	Imtiaz Ahmad	36302-2446316-5	Hafiz Iftikhtiar Ahmad	2,019	13,048	–	15,067	–	13,056	–	13,056
111	**First Capital Equities Ltd**<br>4th Floor, Lakson Square,<br>Building No.1, Sarwar<br>Saheed Road, Karachi.	Mian Ehsan Ul Haq<br>Ahsan Zia Azhar<br>Ahmed Batla<br>Abdul Samad Raja<br>Suhail Qurban<br>Malik Safeer Raza	42301-7045034-9<br>61101-2024487-5<br>42101-1950059-7<br>42000-7573045-7<br>35202-3028538-3	Mian Munawar<br>Mian Zia Ud Din<br>Sheikh Noor<br>Abdul Wahab<br>QurbanHussain<br>Nawazish Hussain	–	98,956	–	98,956	–	98,956	–	98,956
112	**First Capital Equities Ltd**<br>4th Floor, Lakson Square,<br>Building No.1, Sarwar<br>Saheed Road, Karachi."	Mian Ehsan Ul Haq<br>Ahsan Zia Azhar<br>Ahmed Batla<br>Abdul Samad Raja<br>Suhail Qurban<br>Malik Safeer Raza	42301-7045034-9<br>61101-2024487-5<br>42101-1950059-7<br>42000-7573045-7<br>35202-3028538-3	Mian Munawar<br>Mian Zia Ud Din<br>Sheikh Noor<br>Abdul Wahab<br>QurbanHussain<br>Nawazish Hussain	–	121,092	–	121,092	–	121,092	–	121,092
113	**Millat Zarai Services**<br>Tehsil Ahmed Pur Sial<br>District Jhang	Muhammad Aslam<br>Chaudhry Muhammad<br>Akbar	36302-9840417-7<br>33203-1382478-1	Hurmat Khan<br>Hurmat Khan	4,896	454	95	5,445	–	498	95	593

Annexure I

Statement showing written-off loans or any other financial relief of five hundred thousand or above provided during the year 2020

Sr. No.	Name and address of the borrower	Name of individuals/ partners/ directors: Name	NIC No.	Father's /Husband's name	Outstanding liabilities at beginning of year: Principal	Interest / mark up	Others	Total	Principal Written off	Interest/ Markup Written off/ waived	Other Fin. Reliefs Provided	Total
**Domestic**												
114	**Medicon Pharmaceutical Industries (Pvt) Ltd.**<br>B-1/11, Industrial Estate Hayatabad, Jamrud Road Peshawar.	Maqbool Khan<br>Shakeela Bukhari<br>Farjad Maqbool	17301-2252149-9<br>17301-4904731-0<br>17301-9112974-7	Lal Mir Khan<br>W/O Maqbool Khan<br>Maqbool Khan	–	11,236	–	11,236	–	11,236	–	11,236
115	**Sheikho Corporation**<br>Adda Ghulam Hussain Tehsil & District Vehari.	Muhammad Iqbal	36602-0983305-5	Muhammad Tufail	299	1,144	51	1,494	–	1,148	51	1,199
116	**Muhammad Saleem & Co**<br>Adda Jhal Sial Tehsil & District Vehari	Muhammad Saleem	36601-4343571-1	Chaudhry Ranjha Khan	350	669	–	1,019	–	670	–	670
117	**Ali Enterprises**<br>Inside Ahmed Puri Gate Bahawalpur.	Muhammad Iqbal Akhtar	31202-6889172-9	Muhammad Saeed	–	1,767	92	1,859	–	1,767	92	1,859
118	**Ahmad Commission Shop**<br>Shop # 01 Masjid Market Ghallah Mandi Vehari.	Rao Abdul Jabbar Khan	36603-6564830-9	Rao Liloo Khan	–	1,151	46	1,197	–	1,151	46	1,197
119	**Malik Brothers**<br>General Bus Stand Bahawalnagar.	Malik Kashif Zaheer<br>Malik Muhammad Qasim Zaheer<br>Rahat Zaheer	31101-1655553-1<br>31101-1646859-7<br>31101-1580445-4	Zaheer Ahmed<br>Zaheer Ahmed<br>Zaheer Ahmed	–	1,294	50	1,344	–	1,294	50	1,344
120	**Sana Ullah Chatha**<br>Qadirabad Road, Madina Chowk Ali Pur Chatha, Tehsil Wazirabad District Gujranwala.	Sana Ullah Chatha	34104-3085440-7	Ashiq Ali	749	2,323	17	3,089	–	1,770	17	1,787
121	**Sagheer Hussain**<br>17-A, ALAF Town, Behind Khan Colony, near Bypass, Sheikhupura.	Muhammad Sagheer Hussain Shah	35402-1956884-7	Syed Nadir Hussain Shah	150	520	–	670	–	521	–	521
122	**Arif Khalid & Najma Arif**<br>House No. 72, Survey No. 26, 11th Lane, Khayaban-e-Badar, DHA, Phase-VI, Karachi	Arif Khalid<br>Najma Arif	42301-0994647-3<br>42301-0900980-4	Khalid Mehboob<br>W/o. Arif Khalid	2,160	7,344	33	9,537	–	7,354	33	7,387
123	**Shafqat Ali**<br>30-I Block Gulshan-E-Ravi, Lahore	Shafqat Ali	35202-2998805-1	Muhammad Din	–	714	87	801	–	714	87	801
124	**Shaukat Khan**<br>St# 7, Itifaq Colony, Lajpat Road, Shahdara, Lahore	Shaukat Khan	13302-0398861-1	Abdul Raheem Khan	–	481	21	502	–	481	21	502
125	**Pervaiz Akhtar Ch**<br>Dipalpur Distt: Norowal	Pervaiz Akhtar Ch	-	-	216	375	–	591	216	375	–	591
126	**Rehmat Ali**	Rehmat Ali	402-85-084745	-	359	494	–	853	359	494	–	853

MCB Bank for Life

Statement showing written-off loans or any other financial relief of five hundred thousand or above provided during the year 2020

Sr. No.	Name and address of the borrower	Name of individuals/ partners/ directors		Father's /Husband's name	Outstanding liabilities at beginning of year				Principal Written off	Interest/ Markup Written off/ waived	Other Fin. Reliefs Provided	Total
		Name	NIC No.		Principal	Interest / mark up	Others	Total				
**Domestic**												
127	**Habib Ullahn**<br>Vill & Po Fazalabad The. Takhat Bhai Distt. Mardan	Habib Ullahn	-	-	311	265	–	576	311	265	–	576
128	**Ghulam Hussain**	Ghulam Hussain	41103-9298542-7	Mohammad Hussain	249	311	–	560	249	311	–	560
129	**Umed Ali**	Umed Ali	446-87-063810	-	237	285	–	522	237	285	–	522
130	**Muhammad Sachal Khapri**	Muhammad Sachal Khapri	473-45-562892	Mubarik Ali	286	264	–	550	286	264	–	550
131	**Khadim Hussain**	Khadim Hussain	432-85-024995	-	346	280	–	626	346	280	–	626
132	**Rab Nawaz**<br>Rind Mohallah Ghulamullah Distt: Thatta	Rab Nawaz	41406-6205121-7	Haji Allah Obayo Rind	236	510	–	746	236	510	–	746
133	**Ahmed Ali Memon**<br>Hno.35/3/B, Near Imam Bargah Lahori Mohallah Larkana	Ahmed Ali Memon	-	Fateh Muhammad Memon	417	171	–	588	417	171	–	588
134	**Abdul Sattar**<br>Allah Bukhish Arbab The. Tando Allah Yar Distt: Hyderabad	Abdul Sattar	-	Gul Muhammad	384	321	–	705	384	321	–	705
135	**Mujeeb-Ur-Rehman**<br>Hno.158-A, Bhtai Town Jamohoro Hyderabad	Mujeeb-Ur-Rehman	-	Haji Khan	343	326	–	669	343	326	–	669
136	**Muhammad Afzal**<br>Umer Bodla Taluka Sakrand Distt: Nawabshah	Muhammad Afzal	-	Abdul Latif Unner	315	241	–	556	315	241	–	556
137	**Syed Iftikhar Shah**	Syed Iftikhar Shah	432-85-024684	Bahawal shah	345	250	–	595	345	250	–	595
138	**Syed Sabir Hussain**	Syed Sabir Hussain	432-90-240362	-	339	290	–	629	339	290	–	629
139	**Syed Abdul Wahab Shah**	Syed Abdul Wahab Shah	488-90-154338	Syed Haji Khan	306	198	–	504	306	198	–	504
140	**Muhammad Khan Liskani**	Muhammad Khan Liskani	44203-1253564-1	Mataro Khan Liskani	408	286	–	694	408	286	–	694
141	**Mubarak Shah Rice Mills**<br>Main Chiniot Road, Bhowana"	Syed Muhammad Ali Raza<br>Saima Raza	33100-9856916-7<br>33100-0896288-0	Syed Jawad Shah<br>W/O Muhammad Ali Raza	489	1,424	–	1,913	–	868	–	868
142	**Muhammad Usman Sheikh**<br>House No. P-343/3, Main Road Al Masoom Town Faisalabad	Muhammad Usman Sheikh	33100-4555973-7	Sheikh Neik Muhammad	–	3,510	68	3,578	–	3,510	68	3,578
143	**Ch. Abdul Hakeem**<br>Ghullah Mandi, Gujrat	Ch. Abdul Hakeem	52365-4542133-5	Nazir Ahmad	699	1,507	16	2,222	–	1,519	16	1,535

Annexure I

Statement showing written-off loans or any other financial relief of five hundred thousand or above provided during the year 2020

Sr. No.	Name and address of the borrower	Name of individuals/ partners/ directors		Father's /Husband's name	Outstanding liabilities at beginning of year				Principal Written off	Interest/ Markup Written off/ waived	Other Fin. Reliefs Provided	Total
		Name	NIC No.		Principal	Interest / mark up	Others	Total				
**Domestic**												
144	**Lahore Rice Mills** Nankana road, warburton, Thesil Nankana Sahib district Sheikhupura.	Romana Ibrahim (Deceased)<br>Muhammad Ibrahim (Deceased) Abid Latif<br>Zahida Mumtaz	35402-5582769-4<br>35402-1831219-1<br>35501-0322352-3	Muhammad Ibrahim<br>Muhammad Ashiq<br>Sofi Ghulam Yasin<br>Mumtaz Ahmed	–	654	19	673	–	654	19	673
145	**Zahid & CO.** G.T Road Narowal Road, Muridkey.	Zahid Javed	35201-3768775-5	Amjad Hussain	–	851	18	869	–	851	18	869
146	**RN Garments** Habib Park Multan Chungi Lahore."	Rana Nadeem Siddique<br>Muhammad Naeem<br>Siddique Zahida Parveen	35202-2767725-9<br>35202-5476820-3<br>35202-9106400-2	Muhammad Siddique Khan<br>Muhammad Siddique Khan<br>H/O Rana Muhammad Siddique Khan	498	3,922	19	4,439	–	3,924	19	3,943
147	**N.S Surgical** H # 236, Durrani Town, Sialkot	Syed Amjad Ali Shah<br>Syed Tanveer Abbas<br>Muhammad Arshad<br>Muntazir Abbas	34603-5431551-5<br>34603-7573856-7<br>34603-5049096-6<br>34603-7212107-3	Syed Karamat Ali Shah<br>Syed Karamat Ali Shah<br>Saif ud din<br>Karamat Ali	–	5,463	–	5,463	–	5,463	–	5,463
148	**Sheikh Zahir Masud.** House No. 309-B, Valley Road, Westridge -I, Opposite Valley Clinic, Peshawar Road, Rawalpindi	Sheikh Zahir Masud	37405-0658251-7	Sheikh Nisar Ahmad	30	6,163	–	6,193	–	4,939	–	4,939
149	**Zafar Iqbal** House No. G-18, Mohallla Gulnar Colony, Club Line Pehawar Road Rawalpinidi	Zafar Iqbal	37405-3552776-5	Muhammad Iqbal	–	617	–	617	–	617	–	617
150	**Nadeem Khalid** House No. NW-661-A, Saidpur Scheme No. 1, Rawalpindi	Nadeem Khalid	37405-6647588-1	Sheikh Muhammad Khalid	–	3,151	–	3,151	–	3,151	–	3,151
151	**Naseer Ud Din (Naseer** Flour Mills) House No.B-737 , B Block , Tariq Road, St Town Rawalpindi	Naseer ud Din	37405-6004057-7	Rafiq ud Din	–	1,045	–	1,045	–	1,045	–	1,045
152	**Imran Corporation Thatta Garha, Tehsil** Pindi Bhattian, District Hafizabad	Shahid Irfan	34302-3213089-1	Sarfraz Khan	1,450	1,254	70	2,774	–	1,290	70	1,360
153	**Al-Kashif Enterprises** Paris Road, Sialkot"	Azhar Munir	34603-1090308-5	Munir Ahmad	2,456	6,887	18	9,361	–	7,010	18	7,028
154	**Good Shoes** P-358 ,10 Diglus pura,Faisalabad	Muhammad Mukarram	33102-1809505-7	Muhammad Akram	1,197	2,453	93	3,743	–	2,454	93	2,547

Statement showing written-off loans or any other financial relief of five hundred thousand or above provided during the year 2020

Sr. No.	Name and address of the borrower	Name of individuals/ partners/ directors		Father's /Husband's name	Outstanding liabilities at beginning of year				Principal Written off	Interest/ Markup Written off/ waived	Other Fin. Reliefs Provided	Total
		Name	NIC No.		Principal	Interest / mark up	Others	Total				
**Domestic**												
155	**Shaheen Kiran**<br>150 Sarfraz Rafiq Road, Cantt Lahore.	Shaheen Kiran	35201-9415544-2	Jehanzab Qureshi	556	3,623	54	4,233	–	3,483	54	3,537
156	**Javaid Iqbal**<br>House#30, Block K-2 Wapda Town Lahore	Javaid Iqbal<br>Asim Iqbal	35202-2701922-3<br>35202-2701918-9	Sarfraz Ahmed	–	1,109	67	1,176	–	1,109	67	1,176
157	**Muhammad Amir Attique**<br>Data Nagar Badami Bagh 9 B 6 Lahore.	Muhammad Amir Attique	35202-2539918-3	Muhammad Siddique	–	534	19	553	–	534	19	553
158	**Amir Shahzad**<br>Shah Kamal Road, Mughalpura, Lahore.	Amir Shahzad	35201-4665788-3	Muhammad Riaz Akhtar	–	769	15	784	–	769	15	784
159	**Sh. Rasheed & Sons**<br>Narang Mandi, Mohallah SheikhanThana Abadi, Narang Mandi.	Abdul Hadi	35401-7933430-9	Muhammd Rashid	–	681	68	749	–	681	68	749
160	**Sohaib Munir**<br>House No: F-18, Al-Makkah Society, Butt Chowk, Lahore.	Sohaib Munir	35202-8093810-9	Munir Ahmed Tariq	–	677	18	695	–	677	18	695
161	**Ice Berg**<br>House#20, Block B, GECHS, Model Town Link Road, Lahore.	Syed Gul Abid	35200-1423120-5	Syed Ali Abid	–	979	–	979	–	979	–	979
162	**Abdul Samad/Salam/ Sami**<br>134-A-1 Gulberg III Ghalib Market Lahore	Abdul Salam<br>Abdul Samad<br>Abdul Sami	35200-1488952-3<br>35202-2231223-1<br>35202-2017018-7	Abdul Ghani Ch.<br>Abdul Ghani Ch.<br>Abdul Ghani Ch.	–	4,091	19	4,110	–	4,091	19	4,110
163	**Rana Liaqat Ali**<br>House #1, Block 29, Sodiwal Colony, Multan Road, Lahore.	Rana Liaqat Ali	35202-4973490-7	Chaudhary Muhammad Hussain	–	1,398	17	1,415	–	1,398	17	1,415
164	**Afzal Agro Industries**<br>Afzal Chambers Mcload Road, Lahore.	Mr. Muhammad Shahid Afzal<br>Mr. Muhammad Amjad Afzal<br>Mr. Muhammad Wasil Amjad	35200-1548339-7<br>35200-1548348-7<br>35200-1548339-1	Haji Muhammad Afzal<br>Hajid Muhammad Afzal<br>Muhammad Amjad Afzal	–	2,148	17	2,165	–	2,148	17	2,165
165	**Amir & Co.**<br>4-K.M, Narowal Road, Muridke.	Amir Shahzad<br>Adeel Ashraf<br>Muhammad Ashraf	35201-3559789-7<br>35202-2559795-7<br>35202-1017148-7	Muhammad Ashraf<br>Muhammad Ashraf<br>Mian Din Muhammad	–	1,071	47	1,118	–	1,071	47	1,118
166	**Khalid Mehmood Chaudhry**<br>House No. 414, Ravi Block, Allama Iqbal Town, Lahore	Khalid Mehmood Chaudhry	35200-1529618-3	Chaudhry Abdul Rehman	485	2,208	72	2,765	–	2,088	72	2,160

Annexure I

Statement showing written-off loans or any other financial relief of five hundred thousand or above provided during the year 2020

Sr. No.	Name and address of the borrower	Name of individuals/ partners/ directors		Father's /Husband's name	Outstanding liabilities at beginning of year				Principal Written off	Interest/ Markup Written off/ waived	Other Fin. Reliefs Provided	Total
		Name	NIC No.		Principal	Interest / mark up	Others	Total				
**Domestic**												
167	**Ghulam Shabbir** House No: 2, Mohalla, Kachha Rahim Road, Misri Shah, Lahore.	Ghulam Shabbir	35202-3453276-9	Muhammad Ramzan	–	1,542	47	1,589	–	1,542	47	1,589
168	**Imran Jaffery** House No: 122-A, Askari Flats, Gulbeg III, Lahore.	Imran Jaffery	35200-4768906-7	Nawazish Ali Jaffery	–	1,838	80	1,918	–	1,838	80	1,918
169	**"Green Tech International** 4-K.M, Narowal Road, Muridke.	Naeem Kibria Bhatti<br>Ejaz Ahmed	35202-2961155-3<br>35202-4739328-7	Ghulam Kibria Bhatti<br>Niaz Ahmed	–	2,561	17	2,578	–	2,561	17	2,578
170	**Al Madina Medical Store** 544/Huma Block Allama Iqbal Town Lahore.	Mateen Ahmed	35202-8243712-5	Mukhtar Ahmed	–	1,914	47	1,961	–	1,914	47	1,961
171	**Khurram Shahzad** St.#5-Fkotli Per Abdul Rehman Rasheed H#4-A Lahore	Khurram Shahzad<br>Safia BiBi<br>Aslam Zia	35201-3741496-7<br>35201-3273263-2<br>35201-5521292-5	Aslam Zia<br>Aslam Zia<br>Muhamamd Hussain	–	584	62	646	–	584	62	646
172	**Aslam Filling Station** Vehari Road Multan	Malik Muhammad Aslam	36302-0270481-7	Haji Allah Wasaya	78	1,000	17	1,095	–	980	17	997
173	**Rahi Filling Station** Vehari Road Multan	Malik Muhammad Aslam	36302-0270481-7	Haji Allah Wasaya	99	1,417	17	1,533	–	1,418	17	1,435
174	**Pakistan Traders** Ghallah Mandi Bahawalpur	Maqsood Alam	35200-1562539-1	Abdul Majeed	–	959	17	976	–	959	17	976
175	**Khawaja Abid Hussain** House # 24 New Timber Market, Multan	Khawaja Abid Hussain	36302-6159197-9	Aashiq Hussain	–	6,242	66	6,308	–	6,242	66	6,308
176	**Ittefaq Corporation** Chowk B.C.G, Vehari Road Multan.	Muhammad Nisar<br>Dildar Ahmad<br>Faqeer Muhammad	36303-2763026-5<br>36303-7417443-1<br>36303-0975636-7	Hakeem<br>Hakeem<br>Abdul Aziz	–	14,968	–	14,968	–	14,968	–	14,968
177	**Pak Goods Transport Company** 593-Bahawalpur By Pass Chowk Multan.	Muhammad Nisar<br>Muhammad Ajmal Niaz	36303-2763026-5<br>36302-1413702-5	Hakeem<br>Chaudhry Niaz Ahmad	–	640	17	657	–	640	17	657
178	**Allah Wali Medicine Company** Zahid Ali Ldge Aziz street H # 1499/3-E Khawaja Colony Railway Road Multan	Muhammad Zahid Saeed Sheikh	36302-1139784-1	Shekh Muhammad Khalid Saeed	–	1,093	66	1,159	–	1,093	66	1,159
179	**Amar Shaheed Traders** House # D-255, T.C. # 398, Situated at Hindu Muhalla, Near Krishan Nagar, Deh Daharki, Deharki, District Ghotki	Gurdas Mal	45101-9196949-9	Wapari Mal	–	1,219	100	1,319	–	1,219	100	1,319

Statement showing written-off loans or any other financial relief of five hundred thousand or above provided during the year 2020

Sr. No.	Name and address of the borrower	Name of individuals/ partners/ directors		Father's /Husband's name	Outstanding liabilities at beginning of year				Principal Written off	Interest/ Markup Written off/ waived	Other Fin. Reliefs Provided	Total
		Name	NIC No.		Principal	Interest / mark up	Others	Total				
**Domestic**												
180	**Noor Muhammad Shah** Village Wasi Malook Shah, Taluka & District Tando Muhammad Khan	Noor Muhammad Shah	41308-0483317-5	S/o. Syed Shahzado Shah	50	1,006	25	1,081	–	755	25	780
181	**Muhammad Sheraz Qureshi** (House No. B-102, Malir Halt, Shahfaisal Town, Karachi)	Muhammad Sheraz Qureshi	42301-3321612-9	Muhammad Shoaib Qureshi	1,260	1,104	–	2,364	–	1,085	–	1,085
182	**Samee Ullah Traders Through Muhammad Nawaz -Proprietor** Narowal Zafarwal Road, Village Saddowala Uncha, Tehsil & Distt Narowal	Muhammad Nawaz	34501-5145318-7	Babu Khan	599	1,571	52	2,222	–	1,587	52	1,639
183	**Umer Khalid & Company,** Street # 2 Mohallah Hassan Colony Rahim Yar Khan Tehsil & District Rahim Yar Khan.	Mr. Mian Khalid Mehmood	31303-9029704-3	S/o Mr. Mian Ghulam Muhammad	3,606	2,065	92	5,763	–	2,166	92	2,258
184	**Paaris Communication:-** House No. P-375, Street No.06, Redex Road Nisar Colony Faisalabad	Haji Tahir Razzaq	33100-5720478-9	Muhammad Razzaq Ahmed	1,450	1,129	47	2,626	–	1,162	47	1,209
185	**Sheikh Ashfaq Hussain & Sons:-** Main Bazar Gojra District toba Tek Sing	Sheikh Ashfaq Hussain<br>Bilal Shahzad<br>Muhammad Usman Shshzad	33301-7129999-5<br>33301-7224999-5<br>33301-5464298-7	Sheikh Bagh Hussain<br>Sheikh Ashfaq Hussain<br>Sheikh Ashfaq Hussain	780	1,046	69	1,895	–	1,053	69	1,122
186	**Nina Industries Ltd.** A-29/A, Manghopir Road, Karachi	Waqar A. Sattar<br>Saeed A. Sattar<br>Urooj Saeed<br>Kashif Saeed Sattar<br>Yasir Waqar<br>Muzaffar S. Khan<br>Ansar Hussain	42201-6071516-3<br>42201-0937336-9<br>42301-5809641-9<br>42000-5177233-5<br>42201-3174193-5<br>42101-8184503-7<br>42301-0799773-1	Sheikh Abdul Sattar<br>Sheikh Abdul Sattar<br>Saeed A. Sattar<br>Saeed A. Sattar<br>Waqar A. Sattar<br>A S Khan<br>(Late) Jaffer Ali	90,916	130,207	1,942	223,065	90,916	130,207	1,942	223,065
187	**M.Asif Butt** House # 16-A Mehmood Road, New Samnabad, Lahore.	M Asiff Butt	35202-7383230-7	M. Afzal Butt	1,652	1,566	–	3,218	–	1,602	–	1,602
188	**Anser Khan** Latko Chak, Tehsil Shakar Ghar, District Narowal	Mr. Ansar Khan	34502-1599685-2	Mola Dad Khan	–	1,601	67	1,668	–	1,601	67	1,668
189	**Spinzer Enterprises (Pvt) Ltd** Plot No. 101, 102 , Industrial Estate, Jamrud Road, Peshawar	Shehryar Lodhi<br>Nighat Sheryar Lodhi	17301-2071286-3<br>17301-2817365-0	Muhammad Yousaf Lodhi<br>W/o Shehryar Lodhi	6,067	4,725	–	10,792	–	4,880	–	4,880

Annexure I

Statement showing written-off loans or any other financial relief of five hundred thousand or above provided during the year 2020

Sr. No.	Name and address of the borrower	Name of individuals/ partners/ directors		Father's /Husband's name	Outstanding liabilities at beginning of year				Principal Written off	Interest/ Markup Written off/ waived	Other Fin. Reliefs Provided	Total
		Name	NIC No.		Principal	Interest / mark up	Others	Total				
Domestic												
190	**Syed Hassan Sami**<br>House No. 249/B, Sector G-10/4, Islamabad & House No. 10, Street No, 02, Kehkashan Colony Rawalpindi	Syed Hassan Sami	61101-6109414-5	Syed Abdul Sami	1,899	1,279	–	3,178	–	1,322	–	1,322
191	**Malik Rasheed Ahmad**<br>Khanqah Shareef Bahawalpur"	Mr. Malik Rasheed Ahmad	31202-9092109-3	S/o Mr. Noor Muhammad	995	568	37	1,600	–	591	37	628
192	**Manzoor Ahmad Paracha**<br>Ghallah Mandi Dera Ghazi Khan	Mr. Manzoor Ahmad Paracha	32102-0988509-7	S/o Mr. Amir Ahmad	2,199	1,336	43	3,578	–	1,397	43	1,440
193	**Al-Karam Cotton Ginners**<br>Chak # 25-A, Allahabad Road Liaquatpur	Mr. Nisar Mehmood<br>Mr. Atta Muhammad	31302-2354964-9<br>31302-9640717-5	S/o Mr. Jan Muhammad<br>S/o Mr. Jan Muhammad	5,685	10,072	67	15,824	–	10,124	67	10,191
194	**Mehr's Enterprises**<br>Wakeel Khan Road, Kamoke, Gujranwala	Waseem Ejaz Chudhary	34101-2660328-1	Ejaz Hussain Mehr	31,343	17,161	–	48,504	–	17,927	–	17,927
195	**Mian Ahmed Buksh**<br>House No.145, Block-B, Mohafiz town, Sargodha	Mian Ahmed Buksh	38401-7476787-9	Muhammad Nazir	699	609	–	1,308	–	629	–	629
196	**Babar Hayat**<br>House # 251, Block A, New Muslim Town Lahore	Babar Hayat<br>Ghulam Zohra	35202-4841286-5.<br>35202-9757619-2	Ch. Khalid Hayat	–	8,992	–	8,992	–	8,992	–	8,992
197	**Ikram Nabi**<br>30/1, 23rd Street, Khayaban-e-Tanzeem, Phase-V, D.H.A., Karachi	Ikram Nabi	42301-5015587-5	Sajjad Nabi (Late)	3,900	1,681	–	5,581	–	1,808	–	1,808
198	**Syed Amjad Hussain Shah**<br>Mohalla Maqam Sharif, Post Office, Olahi, Khushab	Syed Amjad Hussain Shah	38201-1919058-9	Syed Akbar Shah	969	1,185	32	2,186	–	1,112	32	1,144
199	**Zeeshan Ahmad Siddiqi**<br>Sattlite Town, Gujranwala	Zeeshan Ahmad Siddiqi	34101-2616755-1	Imtiaz Ahmad Sidique	76	502	41	619	–	501	41	542
200	**Muhammad Kashif**<br>St 04 Bazar 02 Razaabad Faisalabad	Muhammad Kashif	33100-8223336-5	Mukhtar Ahmed	474	596	82	1,152	–	515	82	597
201	**Javed Hayat & Khurram Hayat**<br>both residents of House No. 57, Block R – 1, Johar Town, Lahore	Javed Hayat<br>Khurram Hayat	35202-7319007-3<br>35202-3323873-9	Muhammad Hayat<br>Muhammad Hayat	9,681	5,571	73	15,325	–	5,855	73	5,928

Annexure I

Statement showing written-off loans or any other financial relief of five hundred thousand or above provided during the year 2020

Sr. No.	Name and address of the borrower	Name of individuals/ partners/ directors		Father's /Husband's name	Outstanding liabilities at beginning of year				Principal Written off	Interest/ Markup Written off/ waived	Other Fin. Reliefs Provided	Total
		Name	NIC No.		Principal	Interest / mark up	Others	Total				
**Domestic**												
202	**Saifullah Bhatti & Company**<br>Bhatti Town, Sharakpur Sharif, Distt: Sheikhupura.	Saif Ullah Bhatti	35401-2039836-5	Muhammad Sarwar	375	765	–	1,140	–	692	–	692
203	**Universal Tyre Traders**<br>Multan Road, Lahore.	Muhammad Nadeem	35202-3005236-5	Muhammad Shafi	1,572	1,222	77	2,871	–	1,215	77	1,292
204	**Naeem Traders**<br>House 48, Survey # NE-III-48, Hammad Colony, Mouza Baghanwala, Lahore"	Muhammad Naeem<br>Muhammad Umer<br>Muhammad Usman	35202-3348039-7<br>35202-9510325-5<br>35202-3442089-7	Haji Ismail	9,000	11,430	17	20,447	–	11,568	17	11,585
205	**Shahid Hussain Chaudhry**<br>H#131, Umar Block, Allama Iqbal Town, Lahore.	Shahid Hussain Chaudhry	35202-2958356-5	Ch. Ghulam Hussain	6,117	4,107	17	10,241	–	4,291	17	4,308
206	**Usman Enterprises**<br>Noor Rice Mill, Ghallah Mandi, Alipur Chattha.	Muhammad Usman Chattha	35202-1167746-9	Riaz Ahmad Chattha	493	2,365	17	2,875	–	1,871	17	1,888
207	**Haseeb Traders**<br>Mohllah, Normal School, Ghakhar Mandi, Gujranwala	Basharat Ali	34104-2304870-9	Rehmat Ali	218	552	47	817	–	557	47	604
208	**Hi Knit Pvt Limited**<br>Plot # 3,Sector 12-D North Karachi Ind Area Karachi	Syed Jawed Mustafa<br>Mahad Mustafa	42301-6661443-1<br>42301-7559437-7	Syed Muhammad Mustafa<br>Syed Jawed Mustafa	9,318	21,468	–	30,786	9,318	21,468	–	30,786
209	**Sultan Flour Mills**<br>Jampur Road D.G Khan.	Mr. Khaliq Mehmood<br>Mr.Javed Iqbal	32102-0950584-5<br>32102-8697318-1	S/o Mr. Muhammad Azeem<br>S/o Mr. Muhammad Azeem	8,000	6,425	16	14,441	–	6,645	16	6,661
210	**Al-Khaliq Flour Mills**<br>Jampur Road D.G Khan.	Mr. Khaliq Mehmood	32102-0950584-5	S/o Mr. Muhammad Azeem	3,548	4,434	86	8,068	–	4,531	86	4,617
211	**Muhammad Nawaz-**<br>Lubna Akram<br>H#443-A, Nazim Abad, Jhang Road, Faisalabad.	Muhammad Nawaz<br>Lubna Akram	33100-7580792-5<br>33100-0807282-4	Abdul Hameed<br>Akram	812	493	–	1,305	–	512	–	512
212	**Akram & Brothers**<br>63-B-Grain Market Vehari	Mr. Muhammad Akram	36603-8604946-3	S/o Mr. Jamal Din	564	566	17	1,147	–	577	17	594
213	**Move (Pvt.) Ltd**<br>Shah Rukne Alam Colony Multan	Mr. Manzoor Ul Haq<br>Mr. Muhammad Zia Ul Haq<br>Mst.Kaneez Fatima	36302-9989167-1<br>36302-1345146-3<br>36302-5813085-2	S/o Mr. Chaudhry Bag Muhammad<br>S/o Mr. Manzoor Ul Haq<br>W/o Mr. Manzoor Ul Haq	1,164	914	100	2,178	–	933	100	1,033
214	**Rafique Steel**<br>S.S. Centre, Ireland Road, Off Nishtar Road, Lawrence Quarters, Karachi-74200	Muhammad Rafiq	42101-5981988-5	Muhammad Shafi	27,679	19,003	70	46,752	–	19,635	70	19,705

Annexure I

Statement showing written-off loans or any other financial relief of five hundred thousand or above provided during the year 2020

Sr. No.	Name and address of the borrower	Name of individuals/ partners/ directors		Father's /Husband's name	Outstanding liabilities at beginning of year				Principal Written off	Interest/ Markup Written off/ waived	Other Fin. Reliefs Provided	Total
		Name	NIC No.		Principal	Interest / mark up	Others	Total				
**Domestic**												
215	**Al Noor Fertilizer** Industries Limited 96-A, Sindhi Muslim Society Karachi"	Zia I Zakaria<br>Ismail H Zakaria<br>Ellias M Zakaria<br>Yusuf Ayoob<br>Suleman Ayoob<br>Aziz Ayoob	42301-1110269-9<br>514-35-072090<br>514-26-006142<br>517-38-000413<br>514-90-149300<br>514-86-012334	Ismail h Zakaria<br>Haji Zakaria Osman (Late)<br>Haji Zakaria Osman (Late)<br>Muhammad Ayoob (Late)<br>Muhammad Ayoob (Late))<br>Muhammad Ayoob (Late)	–	117,125	–	117,125	–	80,625	–	80,625
216	**International Manufacturing Company** House # 1 Railway Golf Avenue Dharam Pura Canal Bridge Lahore	Khalil Ahmed Khan	35201-1245373-5	Nazir Ahmed Khan	1,178	4,605	–	5,783	469	4,605	–	5,074
217	**Bilal Textile (Pvt) Ltd** P-34, Muslim Town, Officers Block, Near Dar-ul-Quran Behind Crescent Mills, Faisalabad	Muhammad Saleem Umer	33100-0902344-5	Mian Muhammad Umer	29,222	216,983	15	246,220	19,854	217,792	15	237,661
218	**Rehmat Wazir Textile Mills Ltd** 2Km Joyanwala More, Lahore Road, Sheikhupura.	MUHAMMAD NASEER SHEIKH	35202-5720954-7	SHEIKH RAHMAT ALI	–	170,315	–	170,315	–	170,315	–	170,315
219	**Rehmat Wazir Textile Mills Ltd** 2Km Joyanwala More, Lahore Road, Sheikhupura.	MUHAMMAD NAVEED REHMAT	35202-6016395-1	SHEIKH RAHMAT ALI	–	8,154	–	8,154	–	8,154	–	8,154
220	**Rehmat Wazir Textile Mills Ltd** 2Km Joyanwala More, Lahore Road, Sheikhupura.	WAZIR BEGUM	35202-8070430-4	W/O SHEIKH RAHMAT ALI	119,226	159,504	19	278,749	88,626	161,743	19	250,388
221	**Popolar Industries** Toor Abad, Daska Road, Sialkot.	"SHAHEEN BAIG<br>SALEEM MIRZA BAIG"	"34603-3547566-3<br>34603-5328312-1"	"MUHAMMAD BAIG<br>MUHAMMAD BAIG"	–	15,744	–	15,744	–	12,744	–	12,744
222	**Azmat Ali Khan** Mohalla Imamkot, Jalalpur Bhattian, Tehsil Pindi Bhattian, District Hafizabad	Azmat Ali Kham	34302-5609048-1	Muhammad Khan	957	543	67	1,567	–	498	67	565
223	**Iqbal Hussain** 563-24/A, Model Town Gujranwala	Iqbal Hussain	34101-2593426-1	Shah Muhammad	1,953	1,380	–	3,333	–	1,438	–	1,438
224	**Minhas Industries** Ferozwala, S # 5, Shazada Shaheed Colony Gujranwala	Munawar Hussain Minhas	37301-2358190-7	Muhammad Anwar Minhas	–	4,647	87	4,734	–	4,647	87	4,734

Annexure I

Statement showing written-off loans or any other financial relief of five hundred thousand or above provided during the year 2020

Sr. No.	Name and address of the borrower	Name of individuals/ partners/ directors		Father's /Husband's name	Outstanding liabilities at beginning of year				Principal Written off	Interest/ Markup Written off/ waived	Other Fin. Reliefs Provided	Total
		Name	NIC No.		Principal	Interest / mark up	Others	Total				
**Domestic**												
225	**Ranjha Trading Co.** Bhabra Road, Mouza Wan Miana, Tehsil Kotmoman District Sargodha	Muhammad Iqbal	38401-0252726-5	Bahoo Khan	4,999	1,621	67	6,687	–	1,620	67	1,687
226	**Babar Khan Lodhi & Abdul Rasheed** House # 197, Block B, Gulshan-e-Ravi, Lahore	Babar Khan Lodhi<br>Abdul Rasheed	35202-7894730-7<br>35202-2143094-7	Abdul Rasheed<br>Ata Muhammad	2,129	1,755	110	3,994	–	1,548	110	1,658
227	**K-2 Hosiery (Pvt) Limited** 2 – N, Gulberg II, Industrial Area, Lahore	Hassan Mumtaz<br>Mehryar Saigol<br>Saeed Khalid Uppal	35202-2957290-3<br>42301-2403429-5<br>35202-1475288-3	Hussain Mumtaz<br>M. Shakeel Saigol<br>Khalid Javed Uppal	8,139	8,963	77	17,179	3,139	9,096	77	12,312
228	**Al-Mehnaz Traders** Street Kambohan, Farooqabad, Distt. Sheikhopura	Muhammad Zafar Ullah<br>Mst. Musarat Nasrullah	35404-6987159-3	Hafiz Meraj Diin<br>Muhammad Zafar Ullah	469	2,395	28	2,892	–	2,334	28	2,362
229	**M. Arif Sheikh** 2B Commercial Phase, DHA Lahore	M. Arif Sheikh	37406-9390218-5	Sheikh Niaz Ahmed	12,006	1,529	–	13,535	–	1,905	–	1,905
230	**Sahaba International** Islamia Park, Nasir Road, Street No. 10, Sialkot	Asif Ali	34603-4213491-3	Umar Hayat	1,046	3,424	–	4,470	–	3,432	–	3,432
231	**Benish Khan** House No. 52-C-2, Gulberg – III, Lahore	Benish Khan	35202-5120669-8	Naseer Khan	9,243	9,004	166	18,413	–	9,329	166	9,495
					**607,062**	**2,230,023**	**13,749**	**2,850,834**	**299,535**	**2,192,855**	**13,671**	**2,506,061**

Annexure I I

Disposal of fixed assets (refer note 11.2.6)

Description	Cost/ revalued amount	Accumu-lated depreciation	Book value	Sales pro-ceeds	Mode of disposal	Particulars of buyers	Location
		(Rupees in '000)					
**Computers**							
Laptop	88	88	–	9	As per Bank's policy	Muhammad Ali	Lahore
Laptop	116	116	-	12	As per Bank's policy	Usman Hassan	Lahore
Laptop	334	100	244	258	As per Bank's policy	Nabeela Waheed	Lahore
Laptop	236	197	39	44	As per Bank's policy	Nadeem Afzal Khan	Lahore
	**784**	**501**	**283**	**323**			

Consolidated
Financial Statements
MCB Bank Limited

Directors' Report

On Consolidated Financial Statements

The Board of Directors present the report on the consolidated financial statements of MCB Bank Limited and its subsidiaries namely MCB – Arif Habib Savings & Investments Limited, MCB Islamic Bank Limited, Financial & Management Services (Private) Limited and MCB Non-Banking Credit Organization Closed Joint Stock Company for the year ended December 31, 2020.

Profit and Appropriation

The profit before and after taxation for the year ended December 31, 2020 together with appropriations is as under:

	Rs. in Million
**Profit before taxation**	**49,318**
Taxation	19,756
**Profit after taxation**	**29,562**
Profit attributable to non-controlling interest	(152)
**Profit attributable to ordinary shareholders**	**29,410**
Un-appropriated profit brought forward	56,109
Re-measurement of defined benefit plans - net of tax	(342)
Surplus realized on disposal of revalued fixed assets - net of tax	22
Surplus realized on disposal of non-banking assets - net of tax	4
Transferred in respect of incremental depreciation from surplus on revaluation of fixed assets to un-appropriated profit - net of tax	91
	**55,884**
**Profit available for appropriation**	**85,294**
Appropriations:	
Statutory reserve	2,945
Final cash dividend at Rs. 5.0 per share - December 31, 2019	5,925
Interim cash dividend at Rs. 5.0 per share - March 31, 2020	5,925
**Total appropriations**	**14,795**
**Un-appropriated profit carried forward**	**70,499**

Earnings per Share

The consolidated financial statements reflect Rs. 24.82 earnings per share for year under review.

For and on behalf of the Board of Directors,

Imran Maqbool
President & CEO
MCB Bank Limited

Shahzad Hussain
Director
MCB Bank Limited

February 10, 2021

ڈائریکٹرز رپورٹ

بورڈ آف ڈائریکٹرز، ایم سی بی بینک لمیٹڈ اور اس کے ذیلی اداروں جن میں عارف حبیب سیونگز اینڈ انویسٹمنٹ لمیٹڈ، ایم سی بی اسلامک بینک لمیٹڈ، فنانشل اینڈ مینجمنٹ سروسز (پرائیویٹ) لمیٹڈ اور ایم سی بی نان بینک کریڈٹ آرگنائزیشن کلوزڈ جوائنٹ اسٹاک کمپنی شامل ہیں، کی مجموعی مالیاتی اسٹیٹمنٹس برائے سال مختتمہ 31 دسمبر 2020ء پر رپورٹ پیش کرتے ہیں۔

منافع اور تخصیص:

31 دسمبر 2020 کو اختتام پذیر سال میں منافع قبل و بعد از ٹیکس اور تخصیص، مندرجہ ذیل ہے:

	ملین روپے
**منافع قبل از ٹیکس**	49,318
ٹیکس	19,756
**منافع بعد از ٹیکس**	29,562
نان کنٹرولنگ انٹرسٹ کے لیے تفویض شدہ منافع	(152)
**عام حصص یافتگان کے لیے تفویض شدہ منافع**	29,410
افتتاحی غیر تخصیص شدہ منافع	56,109
ڈیفائنڈ بینیفٹ پلان کی تخمینہ نو۔ خالص از ٹیکس	(342)
پائیدار اثاثوں کے سرپلس پر فروخت سے حاصل آمدن (خالص از ٹیکس)	22
غیر بینکاری اثاثوں کے سرپلس پر فروخت سے حاصل آمدن (خالص از ٹیکس)	4
پائیدار اثاثوں کی تخمینہ نو سے اضافی آمدنی سے منتقلی	91
	55,884
**تخصیص کے لیے دستیاب منافع**	85,294
**تخصیص:**	
قانونی ریزرو	2,945
حتمی کیش ڈیوڈینڈ 5 روپے فی حصص 31 دسمبر 2019	5,925
عبوری کیش ڈیوڈینڈ 5 روپے فی حصص 31 مارچ 2020	5,925
**کل تخصیص**	14,795
**افتتاحی غیر تخصیص شدہ منافع**	70,499

آمدنی فی حصص:

مجموعی مالیاتی اسٹیٹمنٹس، اس زیرِ تجزیہ سال کے لیے، 24.82 روپے فی حصص کی آمدنی کو ظاہر کرتے ہیں۔

منجانب و برائے بورڈ آف ڈائریکٹرز

عمران مقبول
پریزیڈنٹ اور سی ای او۔
ایم سی بی بینک لمیٹڈ،

شہزاد حسین
ڈائریکٹر۔ ایم سی بی بینک لمیٹڈ

10 فروری 2021ء

Independent Auditor's Report

To the members of MCB Bank Limited

Report on the Audit of the Consolidated Financial Statements

Opinion

We have audited the annexed consolidated financial statements of MCB Bank Limited and its subsidiaries ("the Group"), which comprise the consolidated statement of financial position as at December 31, 2020 and the consolidated profit and loss account, the consolidated statement of comprehensive income, the consolidated statement of changes in equity and the consolidated statement of cash flows for the year then ended, and notes to the consolidated financial statements, including a summary of significant accounting policies and other explanatory information.

In our opinion, consolidated financial statements give a true and fair view of the consolidated financial position of the Group as at December 31, 2020 and of its consolidated financial performance and its consolidated cash flows for the year then ended in accordance with the accounting and reporting standards as applicable in Pakistan.

Basis for Opinion

We conducted our audit in accordance with International Standards on Auditing (ISAs) as applicable in Pakistan. Our responsibilities under those standards are further described in the Auditor's Responsibilities for the Audit of the Consolidated Financial Statements section of our report. We are independent of the Group in accordance with the International Ethics Standards Board for Accountants' Code of Ethics for Professional Accountants as adopted by the Institute of Chartered Accountants of Pakistan (the Code) and we have fulfilled our other ethical responsibilities in accordance with the Code. We believe that the audit evidence we have obtained is sufficient and appropriate to provide a basis for our opinion.

Key Audit Matters

Key audit matters are those matters that, in our professional judgment, were of most significance in our audit of the consolidated financial statements of the current year. These matters were addressed in the context of our audit of the consolidated financial statements as a whole, and in forming our opinion thereon, and we do not provide a separate opinion on these matters.

Following are the Key Audit Matters:

S. No.	Key Audit Matters	How the matter was addressed in our audit
1	**Provision against advances**	
	Refer to note 11 and the accounting policies in notes 4.2 (b) and 6.5 to the consolidated financial statements.<br><br>The Group's advances to the customers represent 28.96% of its total assets as at 31 December 2020 and are stated at Rs. 547.69 billion which is net of provision of Rs. 50.68 billion at the year end.<br><br>The provision against advances was identified as a key audit matter in our audit as it involves a considerable degree of management judgment and compliance with the Prudential Regulations (PRs) issued by the State Bank of Pakistan.	Our audit procedures in respect of provision against loans and advances included the following:<br><br>• Assessing the design and operating effectiveness of manual and automated controls over classification and provisioning of advances including:<br>- The accuracy of data input into the system used for disbursement and recovery of credit facilities;<br>- Controls over correct classification of non-performing advances on time based criteria;<br>- Controls over accurate computation and recording of provisions; and<br>- Controls over the governance and approval process related to provision.<br>• Testing, on a sample basis, credit exposures identified by the management as displaying indicators of impairment, assessed the number of days overdue and assessed appropriateness of amount reported for provision in accordance with the PRs;

S. No.	Key Audit Matters	How the matter was addressed in our audit
		• Testing, on a sample basis, credit exposure where the management has not identified as displaying indicators of impairment challenged the management's assessment by reviewing the historical performances, account movement, financial ratios and reports on security maintained and formed our own view whether any impairment indicators are present;<br>• For consumer advances, analyzed the days past due report for calculation of provision required in accordance with PRs; and<br>• Checking, on a sample basis, accuracy of specific provision against non-performing advances and of general provision against consumer and SME advances by recomputing the provision made in accordance with the criteria prescribed under the PRs.
2	**Valuation of Investments**	
	Refer to note 10 and the accounting policies in notes 4.2 (c) and 6.3 to the consolidated financial statements.<br><br>As at December 31, 2020, the Group has investments classified as "Available-for-sale", "Held for trading" and "Held to maturity", amounting to Rs. 1,031.74 billion which in aggregate represent 54.55 % of the total assets of the Group.<br><br>Investments are carried at cost or fair value in the accordance with the Group's accounting policy relating to their recognition. Provision against investments is based on impairment policy of the Group which includes both objective and subjective factors.<br><br>We identified the valuation of investments including determination of impairment allowance on investments classified as 'Available-for-sale' and "Held to maturity" as a key audit matter because of their significance in relation to the total assets of the Group and judgment involved in assessing impairment allowance.	Our procedures in respect of valuation of investments included the following:<br>• Assessing the design and tested the operating effectiveness of the relevant controls in place relating to valuation of investments;<br>• Checking, on a sample basis, the valuation of investments to supporting documents, externally quoted market prices and break-up values; and<br>• Evaluating the management's assessment of available for sale and held to maturity investments for any additional impairment in accordance with the Group's accounting policies and performed an independent assessment of the assumptions.

Information Other than the Consolidated Financial Statements and Auditor’s Report Thereon

Management is responsible for the other information. The other information comprises the information included in the Group's Annual Report but does not include the consolidated financial statements and our auditors' report thereon.

Our opinion on the consolidated financial statements does not cover the other information and we do not express any form of assurance conclusion thereon.

In connection with our audit of the consolidated financial statements, our responsibility is to read the other information and, in doing so, consider whether the other information is materially inconsistent with the consolidated financial statements or our knowledge obtained in the audit, or otherwise appears to be materially misstated. If, based on the work we have performed, we conclude that there is a material misstatement of this other information, we are required to report that fact. We have nothing to report in this regard.

Responsibilities of Management and the Board of Directors for the Consolidated Financial Statements

Management is responsible for the preparation and fair presentation of the consolidated financial statements in accordance with accounting and reporting standards as applicable in Pakistan, and Companies Act, 2017 and for such internal control as management determines is necessary to enable the preparation of consolidated financial statements that are free from material misstatement, whether due to fraud or error.

In preparing the consolidated financial statements, management is responsible for assessing the Group’s ability to continue as a going concern, disclosing, as applicable, matters related to going concern and using the going concern basis of accounting unless management either intends to liquidate the Group or to cease operations, or has no realistic alternative but to do so.

The Board of directors is responsible for overseeing the Group’s financial reporting process.

Auditor’s Responsibilities for the Audit of the Consolidated Financial Statements

Our objectives are to obtain reasonable assurance about whether the consolidated financial statements as a whole are free from material misstatement, whether due to fraud or error, and to issue an auditor’s report that includes our opinion. Reasonable assurance is a high level of assurance, but is not a guarantee that an audit conducted in accordance with ISAs as applicable in Pakistan will always detect a material misstatement when it exists. Misstatements can arise from fraud or error and are considered material if, individually or in the aggregate, they could reasonably be expected to influence the economic decisions of users taken on the basis of these consolidated financial statements.

As part of an audit in accordance with ISAs as applicable in Pakistan, we exercise professional judgment and maintain professional skepticism throughout the audit. We also:

- Identify and assess the risks of material misstatement of the consolidated financial statements, whether due to fraud or error, design and perform audit procedures responsive to those risks, and obtain audit evidence that is sufficient and appropriate to provide a basis for our opinion. The risk of not detecting a material misstatement resulting from fraud is higher than for one resulting from error, as fraud may involve collusion, forgery, intentional omissions, misrepresentations, or the override of internal control.

- Obtain an understanding of internal control relevant to the audit in order to design audit procedures that are appropriate in the circumstances, but not for the purpose of expressing an opinion on the effectiveness of the Group’s internal control.

- Evaluate the appropriateness of accounting policies used and the reasonableness of accounting estimates and related disclosures made by management.

- Conclude on the appropriateness of management’s use of the going concern basis of accounting and, based on the audit evidence obtained, whether a material uncertainty exists related to events or conditions that may cast significant doubt on the Group’s ability to continue as a going concern. If we conclude that a material uncertainty exists, we are required to draw attention in our auditor’s report to the related disclosures in the consolidated financial

statements or, if such disclosures are inadequate, to modify our opinion. Our conclusions are based on the audit evidence obtained up to the date of our auditor's report. However, future events or conditions may cause the Group to cease to continue as a going concern.

- Evaluate the overall presentation, structure and content of the consolidated financial statements, including the disclosures, and whether the consolidated financial statements represent the underlying transactions and events in a manner that achieves fair presentation.

- Obtain sufficient appropriate audit evidence regarding the financial information of the entities or business activities within the Group to express an opinion on the consolidated financial statements. We are responsible for the direction, supervision and performance of the group audit. We remain solely responsible for our audit opinion.

We communicate with the Board of Directors regarding, among other matters, the planned scope and timing of the audit and significant audit findings, including any significant deficiencies in internal control that we identify during our audit.

We also provide to the Board of Directors with a statement that we have complied with relevant ethical requirements regarding independence, and to communicate with them all relationships and other matters that may reasonably be thought to bear on our independence, and where applicable, related safeguards.

From the matters communicated with the Board of Directors, we determine those matters that were of most significance in the audit of the consolidated financial statements of the current year and are therefore the key audit matters. We describe these matters in our auditor's report unless law or regulation precludes public disclosure about the matter or when, in extremely rare circumstances, we determine that a matter should not be communicated in our report because the adverse consequences of doing so would reasonably be expected to outweigh the public interest benefits of such communication.

The engagement partner on the audit resulting in this independent auditor's report is M. Rehan Chughtai.

KPMG Taseer Hadi & Co.
Chartered Accountants

Lahore
Date: February 26, 2021

MCB
Bank for Life

Consolidated Statement of Financial Position

As at December 31, 2020

	Note	2020	2019
		(Rupees in '000)	
**ASSETS**			
Cash and balances with treasury banks	7	132,053,041	142,957,358
Balances with other banks	8	29,011,521	21,371,753
Lendings to financial institutions	9	17,968,243	6,060,869
Investments	10	1,036,217,535	757,441,590
Advances	11	547,685,708	548,472,860
Fixed assets	12	63,679,312	64,201,807
Intangible assets	13	1,867,244	1,978,975
Deferred tax assets		–	–
Other assets	14	62,793,791	69,729,659
		1,891,276,395	1,612,214,871
**LIABILITIES**			
Bills payable	16	26,451,513	12,795,325
Borrowings	17	184,577,340	92,859,968
Deposits and other accounts	18	1,388,737,961	1,226,593,025
Liabilities against assets subject to finance lease		–	–
Subordinated debt		–	–
Deferred tax liabilities	19	7,491,040	6,214,223
Other liabilities	20	91,027,158	102,405,513
		1,698,285,012	1,440,868,054
**NET ASSETS**		192,991,383	171,346,817
**REPRESENTED BY**			
Share capital	21	11,850,600	11,850,600
Reserves	22	81,060,051	77,894,829
Surplus on revaluation of assets	23	28,803,351	24,752,206
Unappropriated profit		70,498,820	56,108,779
		192,212,822	170,606,414
Non-controlling interest		778,561	740,403
		192,991,383	171,346,817
**CONTINGENCIES AND COMMITMENTS**	24		

The annexed notes 1 to 48 and annexures I to II form an integral part of these consolidated financial statements.

Imran Maqbool	Hammad Khalid	S.M. Muneer	Mian Umer Mansha	Shahzad Hussain
President/Chief Executive	Chief Financial Officer	Director	Director	Director

Consolidated Profit and Loss Account

For the year ended December 31, 2020

	Note	2020	2019
		(Rupees in '000)	
Mark-up / return / interest earned	26	145,772,451	148,088,700
Mark-up / return / interest expensed	27	69,929,012	84,370,609
Net mark-up / interest income		75,843,439	63,718,091
**NON MARK-UP / INTEREST INCOME**			
Fee and commission income	28	11,971,491	12,230,871
Dividend income		969,322	1,240,771
Foreign exchange income		2,735,228	2,957,999
Income from derivatives		4,087	14,616
Gain on securities	29	3,396,296	828,873
Other income	30	192,103	244,551
Total non-markup / interest Income		19,268,527	17,517,681
Total Income		95,111,966	81,235,772
**NON MARK-UP / INTEREST EXPENSES**			
Operating expenses	31	37,763,917	37,685,431
Workers welfare fund		974,808	802,046
Other charges	32	297,987	260,743
Total non-markup / interest expenses		39,036,712	38,748,220
Share of profit of associates		573,078	339,633
Profit before provisions		56,648,332	42,827,185
Provisions / (reversals) and write offs - net	33	7,330,044	2,673,592
Extra ordinary / unusual items		–	–
**PROFIT BEFORE TAXATION**		49,318,288	40,153,593
Taxation	34	19,756,019	16,206,382
**PROFIT AFTER TAXATION**		29,562,269	23,947,211
Profit attributable to non-controlling interest		(152,042)	(78,962)
**PROFIT ATTRIBUTABLE TO EQUITY SHAREHOLDERS OF THE BANK**		29,410,227	23,868,249
		Rupees	
**Basic and diluted earnings per share**	35	24.82	20.14

The annexed notes 1 to 48 and annexures I to II form an integral part of these consolidated financial statements.

Imran Maqbool	Hammad Khalid	S.M. Muneer	Mian Umer Mansha	Shahzad Hussain
President/Chief Executive	Chief Financial Officer	Director	Director	Director

Consolidated Statement of Comprehensive Income

For the year ended December 31, 2020

	2020	2019
	(Rupees in '000)	
**Profit after taxation for the year**	29,562,269	23,947,211
**Other comprehensive income**		
**Items that may be reclassified to profit and loss account in subsequent periods:**		
Effect of translation of net investment in foreign branches and subsidiaries		
- Equity shareholders of the bank	212,508	1,079,203
- Non-controlling interest	7	20
	212,515	1,079,223
Share of exchange translation reserve of associate	7,321	43,369
Movement in surplus/ (deficit) on revaluation of investments - net of tax		
- Equity shareholders of the bank	4,074,542	7,138,866
Movement in surplus on associated undertaking - net of tax	(24,823)	93,516
	4,049,719	7,232,382
	4,269,555	8,354,974
**Items that will not be reclassified to profit and loss account in subsequent periods:**		
Remeasurement loss on defined benefit obligations - net of tax	(342,311)	(474,421)
Surplus on revaluation of operating fixed assets - net of tax	–	7,038,689
Surplus on revaluation of non-banking assets - net of tax	119,544	165,776
	(222,767)	6,730,044
**Total comprehensive income**	33,609,057	39,032,229
**Attributable to:**		
- Equity shareholders of the bank	33,457,008	38,953,247
- Non-controlling interest	152,049	78,982
	33,609,057	39,032,229

The annexed notes 1 to 48 and annexures I to II form an integral part of these consolidated financial statements.

**Imran Maqbool**	**Hammad Khalid**	**S.M. Muneer**	**Mian Umer Mansha**	**Shahzad Hussain**
President/Chief Executive	Chief Financial Officer	Director	Director	Director

Consolidated Statement of Changes in Equity

For the year ended December 31, 2020

	Share capital	Capital reserve			Statutory reserve	Revenue reserve	Suplus/(deficit) on revaluation of			Unappropriated profit	Total	Non controlling interest	Grand total
		Share premium	Non-distributable capital reserve	Exchange translation reserve		General reserve	Investments	Associate	Fixed / non-banking assets				
	(Rupees in '000)												
Balance as at December 31, 2018	11,850,600	23,973,024	908,317	1,607,782	29,285,450	18,600,000	(2,812,615)	251,246	12,979,244	53,971,079	150,614,127	708,752	151,322,879
**Total comprehensive income for the year ended December 31, 2019**													
Profit after taxation for the year ended December 31, 2019	-	-	-	-	-	-	-	-	-	23,868,249	23,868,249	78,962	23,947,211
Other comprehensive income - net of tax	-	-	-	1,122,572	-	-	7,138,866	93,516	7,204,465	(474,421)	15,084,998	20	15,085,018
	-	-	-	1,122,572	-	-	7,138,866	93,516	7,204,465	23,393,828	38,953,247	78,982	39,032,229
Transfer to statutory reserve	-	-	-	-	2,397,684	-	-	-	-	(2,397,684)	-	-	-
Transfer in respect of incremental depreciation from surplus on revaluation of fixed assets to unappropriated profit - net of tax	-	-	-	-	-	-	-	-	(54,093)	54,093	-	-	-
Surplus realized on disposal of revalued fixed assets - net of tax	-	-	-	-	-	-	-	-	(10,560)	10,560	-	-	-
Surplus realized on disposal of revalued non-banking assets - net of tax	-	-	-	-	-	-	-	-	(37,863)	37,863	-	-	-
**Transactions with owners, recorded directly in equity**													
Final cash dividend at Rs. 4.0 per share - December 31, 2018	-	-	-	-	-	-	-	-	-	(4,740,240)	(4,740,240)	-	(4,740,240)
Interim cash dividend at Rs. 4.0 per share - March 31, 2019	-	-	-	-	-	-	-	-	-	(4,740,240)	(4,740,240)	-	(4,740,240)
Interim cash dividend at Rs. 4.0 per share - June 30, 2019	-	-	-	-	-	-	-	-	-	(4,740,240)	(4,740,240)	-	(4,740,240)
Interim cash dividend at Rs. 4.0 per share - September 30, 2019	-	-	-	-	-	-	-	-	-	(4,740,240)	(4,740,240)	-	(4,740,240)
	-	-	-	-	-	-	-	-	-	(18,960,960)	(18,960,960)	-	(18,960,960)
Share of dividend attributable to Non-controlling interest	-	-	-	-	-	-	-	-	-	-	-	(47,331)	(47,331)
Balance as at December 31, 2019	11,850,600	23,973,024	908,317	2,730,354	31,683,134	18,600,000	4,326,251	344,762	20,081,193	56,108,779	170,606,414	740,403	171,346,817
**Total comprehensive income for the year ended December 31, 2020**													
Profit after taxation for the year ended December 31, 2020	-	-	-	-	-	-	-	-	-	29,410,227	29,410,227	152,042	29,562,269
Other comprehensive income - net of tax	-	-	-	219,829	-	-	4,074,542	(24,823)	119,544	(342,311)	4,046,781	7	4,046,788
	-	-	-	219,829	-	-	4,074,542	(24,823)	119,544	29,067,916	33,457,008	152,049	33,609,057
Transfer to statutory reserve	-	-	-	-	2,945,393	-	-	-	-	(2,945,393)	-	-	-
Transfer in respect of incremental depreciation from surplus on revaluation of fixed assets to unappropriated profit - net of tax	-	-	-	-	-	-	-	-	(90,800)	90,800	-	-	-
Surplus realized on disposal of revalued fixed assets - net of tax	-	-	-	-	-	-	-	-	(22,544)	22,544	-	-	-
Surplus realized on disposal of revalued non-banking assets - net of tax	-	-	-	-	-	-	-	-	(4,774)	4,774	-	-	-
**Transactions with owners, recorded directly in equity**													
Final cash dividend at Rs. 5.0 per share - December 31, 2019	-	-	-	-	-	-	-	-	-	(5,925,300)	(5,925,300)	-	(5,925,300)
Interim cash dividend at Rs. 5.0 per share - March 31, 2020	-	-	-	-	-	-	-	-	-	(5,925,300)	(5,925,300)	-	(5,925,300)
	-	-	-	-	-	-	-	-	-	(11,850,600)	(11,850,600)	-	(11,850,600)
Share of dividend attributable to Non-controlling interest	-	-	-	-	-	-	-	-	-	-	-	(113,891)	(113,891)
**Balance as at December 31, 2020**	11,850,600	23,973,024	908,317	2,950,183	34,628,527	18,600,000	8,400,793	319,939	20,082,619	70,498,820	192,212,822	778,561	192,991,383

For details of dividend declaration and appropriations, please refer note 46 to these consolidated financial statements.

For details of reserves, please refer note 22 to these consolidated financial statements.

The annexed notes 1 to 48 and annexures I to II form an integral part of these consolidated financial statements.

**Imran Maqbool**	**Hammad Khalid**	**S.M. Muneer**	**Mian Umer Mansha**	**Shahzad Hussain**
President/Chief Executive	Chief Financial Officer	Director	Director	Director

Consolidated Cash Flow Statement

For the year ended December 31, 2020

	Note	2020	2019
		(Rupees in '000)	
**CASH FLOW FROM OPERATING ACTIVITIES**			
Profit before taxation		49,318,288	40,153,593
Less: Dividend income and share of profit of associates		(1,542,400)	(1,580,404)
		47,775,888	38,573,189
Adjustments:			
Depreciation on fixed assets	12.2	2,403,220	2,233,008
Depreciation on right of use assets	31	1,697,826	1,610,893
Impairment on fixed assets		4,269	–
Depreciation on non-banking assets acquired in satisfaction of claims	31	30,049	45,456
Amortization	13	469,860	425,585
Provisions / (reversals) and write offs - net	33	7,330,044	2,673,592
Workers welfare fund		974,808	802,046
Gain on sale of non-banking assets acquired in satisfaction of claims	30	(3,976)	(111,948)
Gain on conversion of Ijarah agreements	30	(63,290)	–
Loss on termination of lease liability against right of use assets	30	15,637	–
Charge / (reversal) for defined benefit plan	31.1	74,827	(101,509)
Gain on sale of fixed assets	30	(73,164)	(63,176)
Unrealized loss on revaluation of investments classified as held for trading	29	(110,269)	(83,986)
Interest expensed on lease liability against right-of-use assets	27	1,495,614	1,506,301
		14,245,455	8,936,262
		62,021,343	47,509,451
**Decrease / (increase) in operating assets**			
Lendings to financial institutions		(11,907,374)	33,089,021
Held-for-trading securities		8,244,331	(148,713)
Advances		(6,724,193)	18,629,092
Others assets (excluding advance taxation)		9,319,674	(17,511,282)
		(1,067,562)	34,058,118
**(Decrease) / increase in operating liabilities**			
Bills Payable		13,656,188	(4,207,947)
Borrowings from financial institutions		92,691,055	(129,752,644)
Deposits		162,144,936	104,286,490
Other liabilities (excluding current taxation)		(18,845,300)	14,307,719
		249,646,879	(15,366,382)
Defined benefits paid		(302,940)	(309,074)
Income tax paid		(16,309,576)	(2,506,095)
Net cash flow from operating activities		293,988,144	63,386,018
**CASH FLOW FROM INVESTING ACTIVITIES**			
Net investments in available-for-sale securities		(297,967,382)	6,412,423
Net investments in held-to-maturity securities		17,593,419	(1,001,069)
Dividends received		1,161,822	1,433,411
Investments in fixed assets		(3,051,399)	(4,817,498)
Investments in Intangible assets		(358,181)	(949,464)
Proceeds from sale of fixed assets		191,342	167,374
Proceeds from sale of non-banking assets acquired in satisfaction of claims		39,000	540,000
Investments in non-banking assets acquired in satisfaction of claims		–	(64,445)
Proceeds from divestment in a subsidiary		99,694	–
Effect of translation of net investment in foreign branches & subsidiaries		212,515	1,079,223
Net cash flow (used in) / generated from investing activities		(282,079,170)	2,799,955
**CASH FLOW FROM FINANCING ACTIVITIES**			
Payments of subordinated debt		–	(3,891,019)
Dividend paid		(11,872,206)	(18,877,984)
Payment of lease liability against right-of-use-assets		(2,327,634)	(1,987,479)
**Net cash flow used in financing activities**		(14,199,840)	(24,756,482)
Effects of exchange rate changes on cash and cash equivalents		1,595,436	3,553,077
**Increase in cash and cash equivalents**		(695,430)	44,982,568
Cash and cash equivalents at beginning of the year		161,391,072	118,003,940
**Cash and cash equivalents at end of the year**	36	160,695,642	162,986,508

The annexed notes 1 to 48 and annexures I to II form an integral part of these consolidated financial statements.

Imran Maqbool	Hammad Khalid	S.M. Muneer	Mian Umer Mansha	Shahzad Hussain
President/Chief Executive	Chief Financial Officer	Director	Director	Director

Notes To The Consolidated Financial Statements

For the year ended December 31, 2020

1. STATUS AND NATURE OF BUSINESS

The "Group" consists of

Holding company

MCB Bank Limited (the 'Bank') is a banking company incorporated in Pakistan and is engaged in commercial banking and related services. The Bank's ordinary shares are listed on the Pakistan stock exchange. The Bank's Registered Office and Principal Office are situated at MCB -15 Main Gulberg, Lahore. The Bank operates 1,418 branches (2019: 1,399 branches) within Pakistan and 11 branches (2019: 11 branches) outside Pakistan (including the Karachi Export Processing Zone branch).

1.1 Subsidiary companies

MCB - Arif Habib Savings and Investments Limited

MCB - Arif Habib Savings and Investments Limited was incorporated on August 30, 2000, as an unquoted public limited company. During 2008, the company was listed on the Pakistan Stock Exchange by way of offer for sale of shares by a few of the existing shareholders of the company to the general public. The registered office of the company is situated at 2nd Floor, Adamjee House, I.I Chundrigar Road, Karachi, Pakistan.

The company is registered as an Asset Management Company and Investment Advisor under the Non-Banking Finance Companies (Establishment and Regulation) Rules, 2003 and Pension Fund Manager under Voluntary Pension System Rules 2005. The company also manages discretionary portfolio accounts. The Bank owns 51.33% shares of the company.

MCB Non-Bank Credit Organization Closed Joint Stock Company

MCB Non-banking Credit Organization Closed Joint Stock Company was incorporated on 16 October 2009 and domiciled in the Republic of Azerbaijan. The company is a closed joint stock company limited by shares and was set up in accordance with Azerbaijani regulations. The registered office of the company is located at 49B Tbilisi Ave. Baku AZ1065, Republic of Azerbaijan. The Bank owns 99.94% shares of the company.

The Company's principal business activity is provision of finance leases within the Republic of Azerbaijan. The company leases out various types of industrial equipment, equipment used in medicine, health care, and for other business needs. In addition, the company leases out cars and trucks. Further the company is also involved in real estate finance leases.

MCB Islamic Bank Limited

MCB Islamic Bank Limited (MCBIBL) was incorporated in Pakistan as an unlisted public limited company on May 15, 2014 to carry out the business of an Islamic commercial Bank in accordance and in conformity with the principles of Islamic Shari'ah and in accordance with regulations and guidelines of the State Bank of Pakistan. The Securities and Exchange Commission of Pakistan granted "Certificate of Commencement of Business" to MCBIBL on January 30, 2015. MCBIBL is a wholly owned subsidiary of MCB Bank Limited (MCB).

The State Bank of Pakistan (SBP) granted a "Certificate of Commencement of Banking Business" to MCBIBL on September 14, 2015 under Section 27 of the Banking Companies Ordinance, 1962. MCBIBL formally commenced operations as a Scheduled Islamic Commercial Bank with effect from October 15, 2015 upon receiving notification in this regard from SBP under section 37 of the State Bank of Pakistan Act, 1956. Currently, the Bank is engaged in corporate, commercial, consumer, micro finance, investment and retail banking activities.

MCBIBL is operating through 187 branches in Pakistan (December 31, 2019: 185 branches). The Registered Office of MCBIBL is situated at 59 Block T, Phase II, DHA, Lahore Cantt and Principal Office is situated at 339 Block Z, Phase III, DHA Lahore Cantt.

Financial & Management Services (Private) Limited

Financial & Management Services (Private) Limited is fully provided subsidiary and the company is dormant and has no asset and liability. The Board of Directors of the Bank has approved winding up of the Company. The Bank holds 95.90% shareholding in this subsidiary.

MCB Financial Services Limited

During the year, the Group disposed off its ownership stake in MCB Financial Services Limited, a wholly owned subsidiary, with effect from October 31, 2020.

2. BASIS OF PRESENTATION

2.1 These consolidated financial statements include the financial statements of MCB Bank Limited and its subsidiary companies and share of the profit / reserves of associates (the "Group").

2.2 In accordance with the directives of the Federal Government regarding the shifting of the banking system to Islamic modes, the State Bank of Pakistan has issued various circulars from time to time. Permissible forms of trade-related modes of financing include purchase of goods by banks from their customers and immediate resale to them at appropriate profit in price on deferred payment basis. The purchases and sales arising under these arrangements are not reflected in these consolidated financial statements as such but are restricted to the amount of facility actually utilized and the appropriate portion of profit thereon. The Islamic Banking operations of the Group have complied with the requirements set out under the Islamic Financial Accounting Standards issued by the Institute of Chartered Accountants of Pakistan and notified under the provisions of the Companies Act, 2017.

2.3 Key financial figures of the MCBIBL are disclosed in Annexure II to these consolidated financial statements.

2.4 The consolidated financial statements are presented in Pak Rupees, which is the Group's functional and presentation currency. The amounts are rounded off to the nearest thousand.

3. STATEMENT OF COMPLIANCE

3.1 These consolidated financial statements have been prepared in accordance with accounting and reporting standards as applicable in Pakistan. The accounting and reporting standards comprise of:

- International Financial Reporting Standards IFRS issued by the International Accounting Standards Board (IASB) as are notified under the Companies Act, 2017;
- Islamic Financial Accounting Standards (IFAS) issued by the Institute of Chartered Accountants of Pakistan as are notified under the Companies Act, 2017;
- Provisions of and directives issued under the Banking Companies Ordinance, 1962, the Companies Act, 2017; and
- Directives issued by the SBP and the Securities and Exchange Commission of Pakistan (SECP).

Wherever the requirements of the Banking Companies Ordinance, 1962, Companies Act, 2017 or the directives issued by the SBP and the SECP differ with the requirements of IFRS or IFAS, requirements of the Banking Companies Ordinance, 1962, the Companies Act, 2017 and the said directives shall prevail.

The State Bank of Pakistan has deferred the applicability of International Financial Reporting Standard 9, 'Financial Instruments' through BPRD Circular No. 04 of 2019 dated October 23, 2019 and International Accounting Standards 40, 'Investment Property' for Banking Companies through BSD Circular No. 10 dated August 26, 2002. The Securities and Exchange Commission of Pakistan (SECP) has deferred applicability of IFRS-7 "Financial Instruments: Disclosures" on banks through S.R.O 411(1) /2008 dated April 28, 2008. Accordingly, the requirements of these standards have not been considered in the preparation of these consolidated financial statements. However, investments have been classified and valued in accordance with the requirements prescribed by the State Bank of Pakistan through various circulars.

Notes To The Consolidated Financial Statements
For the year ended December 31, 2020

IFRS10 Consolidated Financial Statements was made applicable from period beginning on or after January 01, 2015 vide S.R.O 633(I)/2014 dated July 10, 2014 by SECP. However, SECP has directed through S.R.O 56(I) /2016 dated January 28, 2016, that the requirements of consolidation under section 237 of the repealed Companies Ordinance 1984 (Section 228 of Companies Act 2017) and IFRS-10 “Consolidated Financial Statements” is not applicable in case of investment by companies in mutual funds established under Trust structure. Accordingly, the requirements of these standards have not been considered in the preparation of these consolidated financial statements.

The State Bank of Pakistan has deferred the applicability of IFAS 3 ‘Profit and Loss Sharing on Deposits’, through BPRD Circular No.04 dated February 25, 2015.

3.2 Standards, interpretations and amendments to published approved accounting standards that are effective in the current year

There are certain new standards and interpretations of and amendments to existing accounting standards that have become applicable to the Group for accounting periods beginning on or after January 1, 2020. These are considered either to not be relevant or not to have any significant impact on the Group’s consolidated financial statements.

3.3 Standards, interpretations and amendments to published approved accounting standards that are not yet effective

The following other standards, amendments and interpretations of approved accounting standards are effective for accounting periods beginning on or after January 1, 2021:

	Effective date (annual periods beginning on or after)
IAS 1, Presentation of Financial Statements (Amendments)	January 1, 2022
IAS 16, Property, plant and equipment (Amendments)	January 1, 2022
IAS 37, Provisions, Contingent Liabilities and Contingent Assets (Amendments)	January 1, 2022

The SECP, through SRO 229(I)/2019 dated February 14, 2019, has notified that IFRS 9, Financial Instruments, is applicable for accounting periods ending on or after June 30, 2019. However, as per BPRD Circular No. 04 of 2019 dated October 23, 2019 of SBP, effective date of IFRS 9 implementation is January 01, 2021. The requirements of this standard have been incorporated in these consolidated financial statements for the jurisdictions/entities where IFRS 9 has been adopted.

IFRS 9, Financial Instruments: Classification and Measurement, addresses recognition, classification, measurement and derecognition of financial assets and financial liabilities. The standard has also introduced a new impairment model for financial assets which requires recognition of impairment charge based on an ‘expected credit losses’ (ECL) approach rather than the ‘incurred credit losses’ approach as currently followed. The ECL approach has an impact on all assets of the Group which are exposed to credit risk. The impact of the application of IFRS 9 in Pakistan on the Group's financial statements is being assessed and SBP's implementation guidelines are awaited.

There are other new and amended standards and interpretations that are mandatory for the Group accounting periods beginning on or after January 1, 2021 but are considered not to be relevant or do not have any significant effect on the Group’s operations and are therefore not detailed in these consolidated financial statements.

4. BASIS OF MEASUREMENT

4.1 These consolidated financial statements have been prepared under the historical cost convention except that certain classes of fixed assets and non-banking assets acquired in satisfaction of claims are stated at revalued amounts and certain investments and derivative financial instruments have been marked to market and are carried at fair value. In addition, obligations in respect of staff retirement benefits are carried at present value.

4.2 Critical accounting estimates and judgments

The preparation of consolidated financial statements in conformity with the approved accounting standards requires the use of certain critical accounting estimates. It also requires the management to exercise its judgment in the process of applying the Group accounting policies. Estimates and judgments are continually evaluated and are based on historical experiences, including expectations of future events that are believed to be reasonable under the circumstances. Revisions to accounting estimates are recognized in the period in which the estimates are revised if the revision affects only that period, or in the period of revision and future periods if the revision affects both current and future periods. The areas where various assumptions and estimates are significant to the Group consolidated financial statements or where judgment was exercised in the application of accounting policies are as follows:

a) Classification of investments

In classifying investments, the Group follows the guidance provided in SBP circulars:

- Investments classified as ‘held for trading’, are securities which are acquired with an intention to trade by taking advantage of short term market / interest rate movements and are to be sold within 90 days of acquisition.
- Investments classified as ‘held to maturity’ are non-derivative financial assets with fixed or determinable payments and fixed maturity. In making this judgment, the Group evaluates its intention and ability to hold such investment to maturity.
- The investments other than those in associates which are not classified as ‘held for trading’ or ‘held to maturity’ are classified as ‘available for sale’.

b) Provision against advances

The Group reviews its loan portfolio including Islamic financing and related assets to assess the amount of non-performing advances and provision required on regular basis. While assessing this requirement various factors including the delinquency in the account, financial position of the borrowers and the requirements of the Prudential Regulations are considered.

The amount of general provision is determined in accordance with the relevant regulations and management’s judgment as explained in note 11.4.4.

c) Impairment of ‘available for sale’ equity investments

The Group determines that ‘available for sale’ equity investments are impaired when there has been a significant or prolonged decline in the fair value below its cost. The determination of what is significant or prolonged requires judgment. In making this judgment, the Group evaluates among other factors, the normal volatility in share price. In addition, the impairment may be appropriate when there is an evidence of deterioration in the financial health of the investee and sector performance, changes in technology and operational/financial cash flows.

d) Taxation

In making the estimates for income taxes currently payable by the Group, the management considers the current income tax laws and the decisions of appellate authorities on certain issues in the past.

e) Fair value of derivatives

The fair values of derivatives which are not quoted in active markets are determined by using valuation techniques. The valuation techniques take into account the relevant underlying parameters including foreign currency involved, interest rates, yield curves, volatilities, contracts duration etc.

f) Depreciation, amortization and revaluation of operating fixed assets

The Group’s management reviews the useful lives and residual values of assets annually by considering expected pattern of economic benefit that the management expects to drive from the

Notes To The Consolidated Financial Statements

For the year ended December 31, 2020

item and the maximum period up to which such benefits are expected to be available. Any change in estimates in future years might affect the carrying amounts of the respective items of assets with a corresponding effect on the depreciation charge and impairment.Such change is accounted for as change in accounting estimates in accordance with International Accounting Standard (IAS) 8 “Accounting Policies, Changes in Accounting Estimates and Errors”. Further, the Group estimates the revalued amount of land and buildings on a regular basis. The estimates are based on valuations carried out by independent professional valuers under the market conditions.

g) Staff retirement benefits

Certain actuarial assumptions have been adopted as disclosed in note 38 of these consolidated financial statements for the actuarial valuation of staff retirement benefit plans. Actuarial assumptions are entity’s best estimates of the variables that will determine the ultimate cost of providing post employment benefits. Changes in these assumptions in future years may affect the liability / asset under these plans in those years.

h) Lease term

The Group applies judgement to determine the lease term for some lease contracts in which it is a lessee that include renewal options. The assessment of whether the Group is reasonably certain to exercise such options impacts the lease term, which significantly affects the amount of lease liabilities and right-of-use assets recognized.

5. FINANCIAL RISK MANAGEMENT

These risk management policies continue to remain robust and the Group is reviewing its portfolio regularly and conducts rapid portfolio reviews in line with emerging risks.

Impact of COVID-19 on the Financial Statements

The COVID - 19 pandemic has taken a toll on all economies and emerged as a contagion risk around the globe, including Pakistan. To reduce the impact on businesses and economies in general, regulators / governments across the globe have introduced a host of measures on both the fiscal and economic fronts.

The State Bank of Pakistan (SBP) has also responded to the crisis by cutting the Policy Rate by 625 basis points during the period to 7% by June 2020. Other regulatory measures to provide an impetus to economic activity include the following:

– Reduction in the capital conservation buffer by 100 basis points to 1.5%;
– Increasing the regulatory limit on extension of credit to SMEs to Rs 180 million;
– Relaxing the debt burden ratio for consumer loans from 50% to 60%;
– Allowing banks to defer borrowers’ principal loan payments by one year and or restructure / reschedule loans for borrowers who require relief of principal repayment exceeding one year and / or mark-up with no reflection on credit history; and
– Introduction of refinancing schemes for payment of wages and salaries.

COVID 19 has impacted the banks in Pakistan from various facets which include muted credit risk increase, reduced fee income due to slowdown in economic activity, branch closures and cyber security threat management.

The potential impact of the economic stress posed by the COVID-19 outbreak is difficult to predict, as many of Group's borrowers have availed the SBP enabled deferment/restructuring & rescheduling relief. Hence, the management has exercised prudence and booked General Provision of Rs. 4.0 billion during the year ended December 31, 2020.

Detailed disclosure on financial risk management has been reported in note 45 to these consolidated financial statements.

Notes To The Consolidated Financial Statements

For the year ended December 31, 2020

6. SUMMARY OF SIGNIFICANT ACCOUNTING POLICIES

The accounting policies adopted in the preparation of these consolidated financial statements are consistent with those of the previous financial year:

6.1 Basis of consolidation

a) These consolidated financial statements include the financial statements of MCB Bank Limited and its subsidiary companies and share of the profit / reserves of associates are accounted for under the equity basis of accounting.

b) Subsidiaries are all entities over which the Group has the power to govern the financial and operating policies accompanying a shareholding of more than one half of the voting rights. The existence and effect of potential voting rights that are currently exercisable are considered when assessing whether the Group controls another entity. Subsidiaries are fully consolidated from the date on which control is transferred to the Group. They are de-consolidated from the date when control ceases / the subsidiaries are disposed off. The assets and liabilities of subsidiary companies have been consolidated on a line by line basis based on the financial statements as at December 31, 2020 and the carrying value of investments held by the Bank is eliminated against the subsidiaries' shareholders' equity in these consolidated financial statement. Material intra-group balances and transactions have been eliminated.

c) Associates are all entities over which the Group has significant influence but not control or joint control. Investments in associates are accounted for by the equity method of accounting and are initially recognised at cost, thereafter for the post-acquisition change in the Group's share of net assets of the associate, the cumulative post-acquisition movements are adjusted in the carrying amount of the investment. Accounting policies of the associates have been changed where necessary to ensure consistency with the policies adopted by the Group.

d) Non-controlling interest is that part of the net results of operations and of net assets of subsidiary companies attributable to interests which are not owned by the Group.

6.2 IFRS 16 - Lease Liability & Right of Use Assets

The lease liability is initially measured at the present value of lease payments to be made over the term of the lease, discounted using the Group's incremental borrowing rate. The lease liability is subsequently measured at amortized cost using the effective interest rate method. The carrying amount is remeasured/adjusted if there are changes in the future cash flows or the lease term.

The right-of-use asset is initially measured at cost, which comprises the initial amount of the lease liability, adjusted for, as applicable, any lease payments made at or before the commencement date. On subsequent measurement, right-of-use assets are stated at cost less any accumulated depreciation / accumulated impairment losses and are adjusted for any remeasurement of the lease liability.

Right-of-use assets are depreciated on a straight line basis over the lease term as this method closely reflects the expected pattern of consumption of future economic benefits. Carrying amount of the lease liability is derecognised upon termination of the lease contract with corresponding adjustment to right-of-use asset. Gain or loss on termination of lease contract is recognised in the profit and loss account.

The Group has elected not to recognize a right-of-use asset and the corresponding lease liability for short-term leases with terms of 12 months or less and leases of low-value assets. Payments associated with these leases are recognized as an expense in the consolidated profit or loss account on a straight-line basis .

Notes To The Consolidated Financial Statements

For the year ended December 31, 2020

6.3 Investments

The Group classifies its investments as follows:

a) Held for trading

These are securities, which are either acquired for generating profit from short-term fluctuations in market prices, interest rate movements, dealers margin or are securities included in a portfolio in which a pattern of short-term profit taking exists.

b) Held to maturity

These are securities with fixed or determinable payments and fixed maturity in respect of which the Group has the positive intent and ability to hold to maturity.

c) Available for sale

These are investments, other than those in subsidiaries and associates, that do not fall under the 'held for trading' or 'held to maturity' categories.

Investments are initially recognized at cost which in case of investments other than 'held for trading' include transaction costs associated with the investment. Transaction costs on investments held for trading are expensed in the profit and loss account.

All purchases and sales of investments that require delivery within the time frame established by regulation or market convention are recognized at the trade date. Trade date is the date on which the Group commits to purchase or sell the investment.

In accordance with the requirements of the State Bank of Pakistan, quoted securities, other than those classified as 'held to maturity' and investments in associates are subsequently re-measured to market value. Surplus / deficit arising on revaluation of quoted securities which are classified as 'available for sale', is taken to surplus / deficit on revaluation of investments through statement of comprehensive income in equity till disposal at which time it is recorded in profit and loss account. Surplus / deficit arising on revaluation of quoted securities which are classified as 'held for trading', is taken to the profit and loss account, currently.

Unquoted equity securities (excluding investments in associates) are valued at the lower of cost and break-up value. Break-up value of equity securities is calculated with reference to the net assets of the investee company as per the latest available financial statements. Investments classified as 'held to maturity' are carried at amortized cost less accumulated impairment losses, if any.

Provision for impairment in the values of securities (except debentures, participation term certificates and term finance certificates) is made currently. Impairment of 'available for sale' equity investments is discussed in 4.2(c). Provisions for impairment in value of debentures, participation term certificates and term finance certificates are made as per the requirements of the Prudential Regulations issued by the State Bank of Pakistan.

Impairment against investment in associates is assessed as per the requirements of IAS 36.

6.4 Sale and repurchase agreements

Securities sold subject to a repurchase agreement (repo) are retained in these consolidated financial statements as investments and the counter party liability is included in borrowings. Securities purchased under an agreement to resell (reverse repo) are not recognized in these consolidated financial statements as investments and the amount extended to the counter party is included in lendings to financial institutions. The difference between the purchase / sale and re-sale / re-purchase price is recognized as mark-up income / expense on a time proportion basis, as the case may be.

6.5 Advances

Advances are stated net of specific and general provisions. Specific provision is determined on the

basis of the Prudential Regulations and other directives issued by the State Bank of Pakistan (SBP) and charged to the profit and loss account. Provisions are held against identified as well as unidentified losses. Provisions against unidentified losses include general provision against Consumer and Small Enterprise (SEs) loans made in accordance with the requirements of the Prudential Regulations issued by SBP and provision based on historical loss experience on advances. General provisions pertaining to overseas advances are made in accordance with the requirements of the regulatory authorities of the respective countries. Advances are written off when there is no realistic prospect of recovery.

Leases where the Group transfers substantially all the risks and rewards incidental to ownership of an asset to the lessee are classified as finance leases. A receivable is recognized at an amount equal to the present value of the lease payments including any guaranteed residual value. Finance lease receivables are included in advances to the customers.

In Murabaha transactions, the Group purchases the goods through its agent or client and after taking the possession, sells them to the customer on cost plus profit basis either in a spot or credit transaction. Under Murabaha financing, funds disbursed for purchase of goods are recorded as 'Advance against Murabaha finance'. On culmination of Murabaha i.e. sale of goods to customers, Murabaha financing are recorded at the deferred sale price. Goods purchased but remaining unsold at the statement of financial position date are recorded as inventories.

The Group values its inventories at the lower of cost and net realizable value. The net realizable value is the estimated selling price in the ordinary course of business less the estimated cost necessary to make the sale. Cost of inventories represents actual purchases made by the Group / customers as the agent of the Group for subsequent sale.

In Ijarah financing, the Group provides the asset on pre-agreed rentals for specific tenors to the customers. Ijarah assets are stated at cost less depreciation and are disclosed as part of 'Islamic financing and related assets'. The rental on Ijarah under Islamic Financial Accounting Standard - 2 Ijarah (IFAS 2) are recorded as income / revenue. The Group charges depreciation from the date of recognition of Ijarah of respective assets to Mustajir. Ijarah assets are depreciated over the period of Ijarah using the straight line method. Impairment of Ijarah assets is determined in accordance with the Prudential Regulations issued by the SBP.

In Diminishing Musharaka based financing, the Group enters into Musharaka based on Shirkat-ul-Milk for financing and agreed share of fixed assets (example: house, land, plant, machinery or vehicle) with its customers and enters into period profit payment agreement for the utilization of the Group's Musharaka share by the customer.

In Istisna financing, the Group acquires the described goods to be manufactured by the customer from raw material of its own and deliver to the Group within an agreed time. The goods are then sold and the amount financed is received back by the Group alongwith profit.

In Salam financing, the Group pays full in advance to its customer for buying specified goods / commodities to be delivered to the Group within an agreed time. The goods are then sold and the amount financed is received back by the Group alongwith profit.

In Running Musharaka based financing, the Group enters into financing with the customer based on Shirkat-ul-Aqd or Business Partnership in the customer's operating business where the funds can be withdrawn or refunded during the Musharakah period.

6.6 Fixed assets and depreciation

Fixed assets other than land and buildings are stated at cost less accumulated depreciation and accumulated impairment losses, if any. Buildings are carried at revalued amount less any accumulated depreciation and subsequent impairment losses, if any. Land is carried at revalued amount less any subsequent impairment losses, if any. Cost of property and equipment of foreign operations includes exchange differences arising on currency translation at year-end rates.

Notes To The Consolidated Financial Statements
For the year ended December 31, 2020

Capital work-in-progress is stated at cost less accumulated impairment losses, if any. These are transferred to specific assets as and when assets become available for use.

Depreciation on all fixed assets (excluding land) is charged using the straight line method in accordance with the rates specified in note 12.2 to these consolidated financial statements and after taking into account residual value, if any. The residual values, useful lives and depreciation methods are reviewed and adjusted, if appropriate, at each balance sheet date.

Depreciation on additions is charged from the month the assets are available for use while no depreciation is charged in the month in which the assets are disposed off.

Land and buildings are revalued by independent, professionally qualified valuers with sufficient regularity to ensure that their net carrying amount does not differ materially from their fair value. An increase arising on revaluation is credited to the surplus on revaluation of fixed assets account. A decrease arising on revaluation of fixed assets is adjusted against the surplus of that asset or, if no surplus exists, is charged to the profit and loss account as an impairment of the asset. A surplus arising subsequently on an impaired asset is reversed through the consolidated profit and loss account up to the extent of the original impairment.

Surplus on revaluation of fixed assets (net of associated deferred tax) to the extent of the incremental depreciation charged on the related assets is transferred to unappropriated profit.

Gains / losses on sale of property and equipment are credited / charged to the consolidated profit and loss account currently, except that the related surplus on revaluation of land and buildings (net of deferred taxation) is transferred directly to unappropriated profit.

Subsequent costs are included in the asset's carrying amount or are recognized as a separate asset, as appropriate, only when it is probable that future economic benefits associated with the item will flow to the Group and the cost of the item can be measured reliably. All other repairs and maintenance are charged to the consolidated profit and loss account.

6.6.1 Intangible assets

Intangible assets are stated at cost less accumulated amortization and accumulated impairment losses, if any. Intangible assets are amortized from the month when these assets are available for use, using the straight line method, whereby the cost of the intangible assets are amortized over its estimated useful lives over which economic benefits are expected to flow to the Group. The useful lives are reviewed and adjusted, if appropriate, at each balance sheet date.

6.7 Impairment

The carrying amount of assets are reviewed at each balance sheet date for impairment whenever events or changes in circumstances indicate that the carrying amounts of the assets may not be recoverable. If such indication exists and where the carrying value exceeds the estimated recoverable amount, assets are written down to their recoverable amounts. The resulting impairment loss is taken to the consolidated profit and loss account except for impairment loss on revalued assets, which is adjusted against the related revaluation surplus to the extent that the impairment loss does not exceed the surplus on revaluation of that asset.

6.8 Staff retirement benefits

MCB Bank Limited (Holding Company)

The Bank operates the following staff retirement benefits for its employees:

a) For clerical / non-clerical staff who did not opt for the new scheme, the Bank operates the following:

- an approved contributory provident fund;
- an approved gratuity scheme; and
- a contributory benevolent scheme

b) For clerical / non-clerical staff who joined the Bank after the introduction of the new scheme and for others who opted for the new scheme introduced in 1975, the Bank operates the following:

- an approved non-contributory provident fund introduced in lieu of the contributory provident fund;
- an approved pension fund; and
- contributory benevolent scheme

c) For officers who joined the Bank after the introduction of the new scheme and for others who opted for the new scheme introduced in 1977, the Bank operates the following:

- an approved non-contributory provident fund introduced in lieu of the contributory provident fund;
- an approved pension fund, and
- contributory benevolent fund.

However, the management has replaced the pension benefits for employees in the officer category with a contributory provident fund for services rendered after December 31, 2003.

d) For executives and officers who joined the Bank on or after January 01, 2000, the Bank operates an approved contributory provident fund.

e) Post retirement medical benefits to entitled employees.

Annual contributions towards the defined benefit plans and schemes are made on the basis of actuarial advice using the Projected Unit Credit Method. The above benefits are payable to staff at the time of separation from the Bank's services subject to the completion of qualifying period of service. Actuarial gains / losses arising from experience adjustments and changes in actuarial assumptions are recognized in other Comprehensive Income in the period of occurrence.

Past service cost is the change in the present value of the defined benefit obligation resulting from a plan amendment or curtailment. The Bank recognizes past service cost as an expense at the earlier of the following dates:

(i) when the plan amendment or curtailment occurs; and

(ii) when the Bank recognizes related restructuring costs or termination benefits.

Employees' compensated absences

Liability in respect of employees' compensated absences is accounted for in the year in which these are earned on the basis of actuarial valuation carried out using the Projected Unit Credit Method. Actuarial gains / losses arising from experience adjustments and changes in actuarial assumptions are recognized in consolidated profit and loss account in the period of occurrence.

MCB Islamic Bank Limited

MCBIBL operates a recognised contributory provident fund scheme for all its permanent employees. Equal monthly contributions are made both by the Bank and its permanent employees, to the Fund at the rate of 8.33% of the basic salaries of employees. However, an employee has an option to increase his/her contribution upto 15% but the Bank will still contribute 8.33% of the employee's basic salary. The Bank has no further payment obligation once the contributions have been paid. The contributions are recognised as employee benefit expense when they are due.

Notes To The Consolidated Financial Statements

For the year ended December 31, 2020

6.9 Taxation

Current

Provision for current taxation is based on taxable income at the current rates of taxation after taking into consideration available tax credits and rebates. The charge for current tax also includes adjustments where considered necessary, relating to prior years which arise from assessments framed / finalized during the year.

Deferred

Deferred tax is recognized using the balance sheet liability method on all temporary differences between the amounts attributed to assets and liabilities for financial reporting purposes and amounts used for taxation purposes. The Group records deferred tax assets / liabilities using the tax rates, enacted or substantively enacted by the balance sheet date expected to be applicable at the time of its reversal. Deferred tax asset is recognized only to the extent that it is probable that future taxable profits will be available against which the asset can be utilized. Deferred tax assets are reduced to the extent that it is no longer probable that the related tax benefit will be realized. The Group also recognizes deferred tax asset / liability on deficit / surplus on revaluation of securities and deferred tax liability on surplus on revaluation of fixed assets which is adjusted against the related deficit / surplus in accordance with the requirements of International Accounting Standard (IAS) 12, 'Income Taxes'.

Deferred tax liability is not recognized in respect of taxable temporary differences associated with exchange translation reserves of foreign operations and subsidiary, where the timing of the reversal of the temporary difference can be controlled and it is probable that the temporary differences will not reverse in the foreseeable future.

6.10 Provisions

Provisions are recognized when the Group has a legal or constructive obligation as a result of past events and it is probable that an outflow of resources will be required to settle the obligation and a reliable estimate of the amount can be made. Provisions are reviewed at each balance sheet date and are adjusted to reflect the current best estimates.

6.11 Foreign currencies

6.11.1 Foreign currency transactions

Transactions in foreign currencies other than the results of foreign operations discussed in note 6.11.2 are translated to Rupees at the foreign exchange rates prevailing on the transaction date. Monetary assets and liabilities in foreign currencies are expressed in Rupee terms at the rates of exchange prevailing at the balance sheet date. Forward foreign exchange contracts are valued at the rates applicable to their respective maturities.

6.11.2 Foreign operations

The assets and liabilities of foreign branches and subsidiary are translated to Rupees at exchange rates prevailing at the statement of financial position date. The results of foreign operations and subsidiary are translated to Rupees at the average rate of exchange for the year.

6.11.3 Translation gains and losses

Translation gains and losses are included in the consolidated profit and loss account, except those arising on the translation of the Group's net investment in foreign branches and subsidiary, which are taken to the capital reserve (exchange translation reserve) until the disposal of the net investment, at which time these are recognized in the consolidated profit and loss account.

6.11.4 Commitments

Commitments for outstanding forward foreign exchange contracts are disclosed in these consolidated financial statements at committed amounts. Contingent liabilities / commitments for letters of credit and letters of guarantee denominated in foreign currencies are expressed in Rupee terms at the rates of exchange prevailing at the statement of financial position date.

Notes To The Consolidated Financial Statements

For the year ended December 31, 2020

6.12 Acceptances

Acceptances comprise undertakings by the Group to pay bills of exchange drawn on customers. The Group expects most acceptances to be simultaneously settled with the reimbursement from the customers.

6.13 Revenue recognition

- Mark-up / interest on advances and returns on investments are recognized on a time proportion basis using the effective interest method except that mark-up / interest on non-performing advances and investments is recognized on a receipt basis, in accordance with the requirements of the Prudential Regulations issued by the State Bank of Pakistan (SBP) or as permitted by the regulations of the overseas regulatory authorities of countries where the branches operate. Where debt securities are purchased at premium or discount, such premium / discount is amortized through the consolidated profit and loss account over the remaining period of maturity.
- Financing method is used in accounting for income from lease financing. Under this method, the unearned lease income (excess of the sum of total lease rentals and estimated residual value over the cost of leased assets) is deferred and taken to income over the term of the lease period so as to produce a constant periodic rate of return on the outstanding net investment in lease. Gains / losses on termination of lease contracts are recognized as income when these are realized.
- Profit on investments in Sukuks is recognised on time proportion basis. Where Sukuks (excluding those classified as held for trading) are purchased at a premium or discount, such premiums / discounts are amortised through the profit or loss account using the effective yield method.
- Profit on Salam financing is recognised on time proportion basis.
- Profit on Running Musharaka financing is booked on an accrual basis and is subject to adjustment (if any) upon declaration of profit by Musharakah partners.
- Commission income is recognized on a time proportion basis.
- Dividend income is recognized when the Group's right to receive dividend is established.
- Gain / loss on sale of investments is credited / charged to the consolidated profit and loss account currently.
- Rental income from Ijarah financing is recognised on an accrual basis. Depreciation on Ijarah asset is charged to income (net of with rental income) over the period of Ijarah using the straight line method.
- Profit from Bai-Mua'jjal is recognised on a time proportionate basis.
- Profit on Diminishing Musharaka is recognised on a time proportionate basis.
- Profit on Istisna financing is recognised on a time proportionate basis.
- Profit from Musharaka placements with financial institutions is recognised on a time proportionate basis.
- Profit from Murabaha financing is accounted for on culmination of Murabaha transaction. Profit on Murabaha is recognised on an accrual basis. Profit on Murabaha transactions for the period from the date of disbursement to the date of culmination of Murabaha is recognised immediately on the later date.
- Revenue for acting as trustee is recognized on Net Assets Value (NAV) of respective funds.
- Management / advisory fee is calculated on a daily / monthly basis by charging specified rates to the net assets value / income of the Collective Investment Schemes. Advisory fee from the discretionary portfolio is calculated in accordance with the respective agreements with the clients. Management fee from the pension funds is calculated by charging the specified rates to the average net assets value.
- Revenue from trusteeship and custodian is recognised when the group satisfies a performance obligation by rendering promised services as per respective agreements.

Notes To The Consolidated Financial Statements
For the year ended December 31, 2020

The income on Islamic financing is recognised in accordance with the principles of Islamic Shari'ah and in accordance with regulations and guidelines of the State Bank of Pakistan. However, income, if any, received which does not comply with the principles of Islamic Shari'ah is recognised as charity payable if so directed by the Shari'ah Board of the Group.

6.14 Assets acquired in satisfaction of claims

Non-banking assets (NBA) acquired in satisfaction of claims are carried at revalued amounts less accumulated depreciation and impairment loss. These assets are revalued by professionally qualified valuers with sufficient regularity to ensure that their net carrying value does not differ materially from their fair value. A surplus arising on revaluation of property is credited to the 'surplus on revaluation of non banking assets' account through consolidated statement of comprehensive income in equity and any deficit arising on revaluation is taken to consolidated profit and loss account directly. Legal fees, transfer costs and direct costs of acquiring title to property are charged to the consolidated profit and loss account and not capitalized.

6.15 Cash and cash equivalents

Cash and cash equivalents include cash and balances with treasury banks and balances with other banks (net of overdrawn Nostro balances) in current and deposit accounts.

6.16 Financial instruments

6.16.1 Financial assets and financial liabilities

Financial instruments carried on the statement of financial position include cash and balances with treasury banks, balances with other banks, lendings to financial institutions, investments, advances, other assets, bills payable, borrowings, deposits and other liabilities. The particular recognition methods adopted for significant financial assets and financial liabilities are disclosed in the individual policy statements associated with these assets and liabilities.

6.16.2 Derivative financial instruments

Derivative financial instruments are initially recognized at fair value on the date on which a derivative contract is entered into and are subsequently remeasured at their fair value using valuation techniques. All the derivative financial instruments are carried as an asset when the fair value is positive and as a liability when the fair value is negative. Any change in the fair value of derivative financial instruments is taken to the consolidated profit and loss account currently.

6.16.3 Off setting

Financial assets and financial liabilities are off set and the net amount is reported in these consolidated financial statements when there is a legally enforceable right to set off and the Group intends either to settle on a net basis, or to realize the assets and settle the liabilities, simultaneously.

6.17 Borrowings / deposits

Borrowings / deposits are recorded at the proceeds received. The cost of borrowings / deposits is recognized as an expense in the period in which this is incurred.

Deposits, with respect to Islamic Banking operations, are generated on the basis of two modes i.e. Qard and Mudaraba.

Deposits taken on Qard basis are classified as 'Current accounts' and Deposits generated on Mudaraba basis are classified as 'Savings deposits' or 'Fixed deposits'. No profit or loss is passed to current account depositors.

Profits realized in investment pools are distributed in pre-agreed profit sharing ratio. Rab-ul-Maal share is distributed among depositors according to weightages assigned at the inception of profit calculation period.

Profits are distributed from the pool such that the depositors (remunerative) only bear the risk of assets in the pool during the profit calculation period. In case of loss in a pool during the profit calculation period, the loss is distributed among the depositors (remunerative) according to their ratio of Investments.

Asset pools may be created at the Group's discretion and the Group may add, amend, and transfer an asset to any other pool in the interests of the deposit holders.

6.18 Segment reporting

A segment is a distinguishable component of the Group that is engaged in providing products or services (business segment) or in providing products or services within a particular economic environment (geographical segment), which is subject to risks and rewards that are different from those of other segments. The Group's primary format of reporting is based on business segments.

6.18.1 Business segments

Retail banking

This includes retail lending and deposits, banking services, cards and branchless banking.

Corporate banking

This comprises of loans, deposits, project financing, trade financing, investment banking and other banking activities / with Group's corporate and public sector customers.

Consumer Banking

This segment primarily constitutes consumer financing activities with individual customers of the Group. Product suite offered to these customers include credit cards, auto loans, housing finance and personal loans.

Islamic Banking

This segment includes Islamic Banking operations of the Group.

Treasury

This includes fixed income, equity, foreign exchange, credit, funding, own position securities, lendings and borrowings and derivatives for hedging and market making.

Assets Management

It includes asset management, investment advisory, portfolio management, equity research and underwriting.

International Banking

This comprises of loans, deposits, project financing, trade financing, investment banking and other banking activities by Group's overseas operations.

Others

This includes the head office related activities and other functions which cannot be classified in any of the above segments.

6.18.2 Geographical segments

The Group operates in four geographic regions being:

- Pakistan
- South Asia
- Middle East
- Eurasia

Notes To The Consolidated Financial Statements

For the year ended December 31, 2020

6.19 Dividend distribution and appropriation

Dividends (including bonus dividend) and other appropriations (except appropriations which are required by law) are recognized in the period in which these are approved.

6.20 Business combination

Business combinations other than under common control transaction are accounted for by applying the acquisition method. The cost of acquisition is measured as the fair value of assets given, equity instruments issued and the liabilities incurred or assumed at the date of acquisition. The consideration transferred includes the fair value of any asset or liability resulting from a contingent consideration arrangement, if any. Acquisition-related costs are expensed as incurred. Identifiable assets acquired and liabilities and contingent liabilities assumed in a business combination are measured initially at their fair values at the acquisition date. The excess of the consideration transferred over the fair value of the identifiable net assets acquired is recorded as goodwill. If this is less than the fair value of the net assets acquired in the case of a bargain purchase, the difference is recognized directly in the consolidated profit and loss account or as directed by the SBP.

6.21 Earnings per share

The Group presents basic and diluted earnings per share (EPS). Basic EPS is calculated by dividing the profit or loss attributable to ordinary shareholders of the Group by the weighted average number of ordinary shares outstanding during the year.

6.22 Pool Management

The Group operates general and specific pools for deposits and inter-Group funds accepted / acquired under Mudaraba, Musharaka and Wakala modes.

Under the general deposits pool, the Group accepts funds on Mudaraba basis from depositors (Rabb-ul-Maal) where the Group acts as Manager (Mudarib) and invests the funds in the Shari'ah Compliant modes of financings, investments and placements. When utilising investing funds, the Group prioritizes the funds received from depositors over the funds generated from own sources.

Specific pools may be operated for funds acquired / accepted from the State Bank of Pakistan and other Groups for Islamic Export Refinance to Group's customers and liquidity management respectively under the Musharaka / Mudaraba modes. The Group also maintains an Equity Pool which consists of Group's equity and funds accepted on Qard (non-remunerative current deposit account) basis.

The profit of each deposit pool is calculated on all the remunerative assets booked by utilizing the funds from the pool after deduction of expenses directly incurred in earning the income of such pool, if any. The directly related costs comprise of depreciation on Ijarah assets, takaful premium, documentation charges etc. No general or administrative nature of expense is charged to pool. No provision against any non-performing asset of the pool is passed on to the pool except on the actual loss / write-off of such non-performing asset. The profit of the pool is shared between equity and other members of the pool on the basis of Musharaka at gross level (before charging of Mudarib fee) as per the investment ratio of the equity. The profit of the pool is shared among the members of the pool on pre-defined mechanism based on the weightages announced before the profit calculation period after charging of Mudarib fee.

The deposits and funds accepted under the above mentioned pools are provided to diversified sectors and avenues of the economy / business as mentioned in the notes and are also invested in Government of Pakistan backed Ijarah Sukuks. Staff financings are exclusively financed from the equity pool.

The risk characteristic of each pool mainly depends on the assets and liability profile of each pool.

6.23 Funds due to / from financial institutions

Bai Mu'ajjal

In Bai Mu'ajjal, the Group sells sukuk on credit to other financial institutions. The credit price is agreed at the time of sale and such proceeds are received at the end of the credit period.

Musharaka / Mudaraba / Wakala

In Musharaka / Mudaraba / Wakala, the Group invests in the Shariah compliant business pools of the financial institutions at the agreed profit and loss sharing ratio / fees.

	Note	2020	2019
		(Rupees in '000)	
**7. CASH AND BALANCES WITH TREASURY BANKS**			
In hand			
Local currency		25,039,386	20,833,520
Foreign currencies		7,088,257	2,802,190
		32,127,643	23,635,710
With State Bank of Pakistan in			
Local currency current account	7.1	51,920,851	62,180,493
Foreign currency current account	7.2	2,271,659	599,384
Foreign currency deposit account	7.3	10,532,776	14,320,863
		64,725,286	77,100,740
With other central banks in			
Foreign currency current account	7.4	11,851,311	16,220,148
With National Bank of Pakistan in			
Local currency current account		22,308,442	25,666,525
Prize bonds		1,040,359	334,235
		132,053,041	142,957,358

7.1 This represents current accounts maintained with the SBP under the Cash Reserve Requirement of section 22 of the Banking Companies Ordinance, 1962.

7.2 This represents US Dollar settlement account maintained with SBP.

7.3 This represents account maintained with the SBP to comply with the Special Cash Reserve requirement. This includes balance of Rs. 6,810.656 million (2019: Rs. 10,517.551 million) which carries interest rate of 0% (2019: 0.70%) per annum as declared by SBP.

7.4 Foreign currency current account with other central banks are maintained to meet their minimum cash reserves and capital requirements pertaining to the foreign branches of the Bank.

Notes To The Consolidated Financial Statements

For the year ended December 31, 2020

	Note	2020	2019
		(Rupees in '000)	
**8. BALANCES WITH OTHER BANKS**			
In Pakistan			
In current account		9,190	9,183
In deposit account	8.1	1,554	4,378,142
		10,744	4,387,325
Outside Pakistan			
In current account		26,768,812	12,838,712
In deposit account	8.2	2,231,965	4,145,716
		29,000,777	16,984,428
		29,011,521	21,371,753

8.1 This represents saving accounts carrying profit at expected rates ranging from 2.13% to 6.00% per annum (2019: 3% to 12.54% per annum).

8.2 Balances with other banks outside Pakistan in deposit accounts carry interest rate of 1.5% (2019: 2.60% to 8.30%) per annum.

	Note	2020	2019
		(Rupees in '000)	
**9. LENDINGS TO FINANCIAL INSTITUTIONS**			
Call / clean money lendings	9.1	11,002,195	209,205
Repurchase agreement lendings (Reverse Repo)	9.2	6,137,258	–
Musharaka arrangements		–	2,480,000
Bai Muajjal receivable - with State Bank of Pakistan	9.3	828,790	3,371,664
		17,968,243	6,060,869

9.1 Call money lending carries mark-up rate of 0.1% to 4.55% (2019: 7 %) per annum and is due to mature in January 2021.

9.2 Repurchase agreement lendings carry mark-up rates of 6.4% to 7.1% per annum and is due to mature in January 2021.

9.3 This represents Bai Muajjal receivable arrangements with State Bank of Pakistan carries rate of 9.14% per annum (2019: average rate of 10.34%) and having maturity till January 2021.

	2020	2019
	(Rupees in '000)	
**9.4 Particulars of lending**		
In local currency	6,966,048	5,851,664
In foreign currencies	11,002,195	209,205
	17,968,243	6,060,869

Notes To The Consolidated Financial Statements
For the year ended December 31, 2020

	2020			2019		
	Held by Bank	Further given as collateral	Total	Held by Bank	Further given as collateral	Total
	(Rupees in '000)					
**9.5 Securities held as collateral against lendings to financial institutions**						
Market Treasury Bills	6,137,258	–	6,137,258	–	–	–
Total	6,137,258	–	6,137,258	–	–	–

10. INVESTMENTS

10.1 Investments by type:

	Note	2020				2019			
		Cost/ amortised cost	Provision for diminution	Surplus/ (Deficit)	Carrying value	Cost/ amortised cost	Provision for diminution	Surplus (Deficit)	Carrying value
		(Rupees in '000)							
**Held-for-trading securities**									
Federal Government Securities		1,309,116	–	(224)	1,308,892	9,479,849	–	(4,128)	9,475,721
Shares and units		1,158,952	–	110,493	1,269,445	1,062,839	–	88,114	1,150,953
		2,468,068	–	110,269	2,578,337	10,542,688	–	83,986	10,626,674
**Available-for-sale securities**									
Federal Government Securities		969,144,840	(4,719)	9,477,853	978,617,974	677,027,359	–	4,973,965	682,001,324
Shares and units		28,398,065	(11,155,719)	3,412,147	20,654,493	26,846,236	(11,207,932)	1,673,181	17,311,485
Non Government Debt Securities		2,841,840	–	16,780	2,858,620	2,428,773	–	(4,671)	2,424,102
Foreign Securities		7,463,939	(1,714)	17,509	7,479,734	3,657,020	–	13,295	3,670,315
		1,007,848,684	(11,162,152)	12,924,289	1,009,610,821	709,959,388	(11,207,932)	6,655,770	705,407,226
**Held-to-maturity securities**									
Federal Government Securities		7,313,217	(11,542)	–	7,301,675	18,908,076	(2,211)	–	18,905,865
Provincial Government Securities		118	(118)	–	–	118	(118)	–	–
Non Government Debt Securities		9,615,317	(490,341)	–	9,124,976	10,442,925	(533,788)	–	9,909,137
Foreign Securities		3,149,647	(27,281)	–	3,122,366	8,320,599	(3,569)	–	8,317,030
		20,078,299	(529,282)	–	19,549,017	37,671,718	(539,686)	–	37,132,032
**Associates**	10.11	4,479,360	–	–	4,479,360	4,275,658	–	–	4,275,658
**Total Investments**		1,034,874,411	(11,691,434)	13,034,558	1,036,217,535	762,449,452	(11,747,618)	6,739,756	757,441,590

Notes To The Consolidated Financial Statements
For the year ended December 31, 2020

10.2 Investments by segments:

	Note	2020 Cost/ amortised cost	2020 Provision for diminution	2020 Surplus/ (Deficit)	2020 Carrying value	2019 Cost/ amortised cost	2019 Provision for diminution	2019 Surplus (Deficit)	2019 Carrying value
		(Rupees in '000)							
**Federal Government Securities:**									
Market Treasury Bills		598,470,191	–	256,237	598,726,428	397,866,023	–	(413,415)	397,452,608
Pakistan Investment Bonds		349,738,865	–	9,180,611	358,919,476	292,723,081	–	5,376,303	298,099,384
Sukuks bonds		24,294,814	(2,872)	(24,383)	24,267,559	11,094,999	–	6,949	11,101,948
Naya Pakistan Certificates		465,779	–	–	465,779	–	–	–	–
Euro Bonds		2,096,697	(13,389)	65,166	2,148,474	1,030,354	(2,211)	–	1,028,143
Bai Mu'ajjal		2,700,827	–	–	2,700,827	2,700,827	–	–	2,700,827
		977,767,173	(16,261)	9,477,631	987,228,543	705,415,284	(2,211)	4,969,837	710,382,910
**Provincial Government Securities**		118	(118)	–	–	118	(118)	–	–
**Shares:**									
Listed Companies		27,974,567	(10,986,392)	3,522,638	20,510,813	26,355,910	(11,042,016)	1,761,295	17,075,189
Unlisted Companies		1,582,450	(169,325)	–	1,413,125	1,553,165	(165,916)	–	1,387,249
		29,557,017	(11,155,717)	3,522,638	21,923,938	27,909,075	(11,207,932)	1,761,295	18,462,438
**Non Government Debt Securities**									
Listed		5,613,730	(16,269)	12,037	5,609,498	5,800,646	(16,269)	(2,050)	5,782,327
Unlisted		6,843,427	(474,072)	4,743	6,374,098	7,071,052	(517,519)	(2,621)	6,550,912
		12,457,157	(490,341)	16,780	11,983,596	12,871,698	(533,788)	(4,671)	12,333,239
**Foreign Securities**									
Government securities		10,606,326	(27,281)	17,509	10,596,554	11,517,802	(2,828)	13,295	11,528,269
Non Government Debt securities		–	–	–	–	452,567	(741)	–	451,826
Unlisted equity securities		7,260	(1,716)	–	5,544	7,250	–	–	7,250
		10,613,586	(28,997)	17,509	10,602,098	11,977,619	(3,569)	13,295	11,987,345
**Associates**									
- Adamjee Insurance Company Limited	10.9	4,435,075	–	–	4,435,075	4,211,707	–	–	4,211,707
- Euronet Pakistan (Private) Limited	10.10	44,285	–	–	44,285	63,951	–	–	63,951
		4,479,360	–	–	4,479,360	4,275,658	–	–	4,275,658
**Total Investments**		1,034,874,411	(11,691,434)	13,034,558	1,036,217,535	762,449,452	(11,747,618)	6,739,756	757,441,590

10.2.1 Investments given as collateral

	2020	2019
	(Rupees in '000)	
- Market Treasury Bills	91,279,273	22,820,226
- Pakistan Investment Bonds	1,000,283	5,316,208
	92,279,556	28,136,434

Notes To The Consolidated Financial Statements

For the year ended December 31, 2020

10.3 Provision for diminution in value of investments

		Note	2020	2019
			(Rupees in '000)	
10.3.1	**Opening balance**		11,747,618	8,746,566
	Exchange and other adjustments		13,474	251
	Charge / (reversals)			
	Charge for the year		1,993,335	3,452,808
	Reversals for the year		(1,529)	(29,964)
	Reversal on disposals		(2,061,464)	(422,043)
			(69,658)	3,000,801
	Amounts written off		–	–
	Closing balance	10.3.3	11,691,434	11,747,618

10.3.2 Particulars of provision against debt securities

Category of classification

	2020		2019	
	NPI	Provision	NPI	Provision
	(Rupees in '000)			
**Domestic**				
Doubtful	–	–	145,656	72,828
Loss	490,459	490,459	461,078	461,078
	490,459	490,459	606,734	533,906

10.3.3 This includes a general provision of Rs 43.542 million (December 31, 2019: Rs 5.780 million) held by overseas branches in accordance with the requirements of IFRS 9.

10.4 Quality of Available for Sale Securities

Details regarding quality of Available for Sale (AFS) securities are as follows;

	2020	2019
	Cost	
	(Rupees in '000)	
**Federal Government Securities - Government guaranteed**		
Market Treasury Bills	597,161,075	386,882,153
Pakistan Investment Bonds	346,405,146	278,739,813
Euro Bonds	1,341,706	506,323
Sukuk Bonds	23,771,134	10,899,070
Naya Pakistan Certificates	465,779	–
	969,144,840	677,027,359

Notes To The Consolidated Financial Statements

For the year ended December 31, 2020

	2020	2019
	Cost	
	(Rupees in '000)	
**Listed Companies and mutual funds**		
Automobile Assembler	1,573,156	1,385,302
Automobile Part and Accessories	413,930	413,930
Cable and Electrical Goods	659,439	659,439
Cement	2,050,172	2,100,969
Chemical	285,638	76,050
Close end Mutual Fund	1,186,851	1,186,851
Commercial Banks	3,002,215	2,014,437
Engineering	1,854,002	2,085,008
Fertilizer	3,469,300	3,047,663
Food and Personal Care Products	1,229,398	1,199,266
Insurance	599,364	599,364
Investment Banks / Companies	41,784	41,784
NIT Units	5,253	5,253
Oil & Gas Exploration Companies	2,864,529	2,649,990
Oil & Gas Marketing Companies	760,532	742,705
Open End Mutual Fund	190,083	312,349
Paper and Board	648,581	700,059
Pharmaceutical	1,103,230	1,230,160
Power Generation and Distribution	2,616,001	2,616,001
Real Estate Investment Trust	–	
Refinery	1,107,388	981,399
Technology and Communication	707,827	706,796
Textile	446,942	538,296
	26,815,615	25,293,071

	2020		2019	
	Cost	Breakup value	Cost	Breakup value
	(Rupees in '000)			
**Unlisted Companies**				
Central Depository Company Limited	184,426	756,153	184,426	687,011
First Capital Investment (Pvt) Limited	2,500	2,667	2,500	3,334
First Women Bank Limited	63,300	215,838	63,300	215,838
ISE Towers Reit Management Company Limited	30,346	93,780	30,346	87,958
National Investment Trust Limited	1,027,651	1,661,565	1,027,651	1,665,882
National Institutional Facilitation Technologies	1,527	51,998	1,527	51,641
Pak Agro Storage And Service Corporation	2,500	1,239,050	2,500	605,006
1 Link (Pvt) Limited	50,000	202,032	50,000	147,332
Naymat Collateral Management Company	29,285	25,876	–	–
Arabian Sea Country Club	5,000	–	5,000	–
SME Bank Limited	10,106	–	10,106	–
Al-Ameen Textile Mills Limited	197	–	197	–
Custodian Management Services	1,000	–	1,000	–
Galaxy Textile Mills Limited	30,178	–	30,178	–
Pakistan Textile City (Pvt) Limited	50,000	–	50,000	–
Ayaz Textile Mills Limited	2,253	–	2,253	–
Musarrat Textile Mills Limited	36,045	–	36,045	–
Sadiqabad Textile Mills Limited	26,361	–	26,361	–
Al-Arabia Sugar Mills Limited - Preference shares	4,775	–	4,775	–
Pak Elektron Limited - Preference shares	25,000	25,000	25,000	25,000
	1,582,450	4,273,959	1,553,165	3,489,002

Notes To The Consolidated Financial Statements

For the year ended December 31, 2020

	2020	2019
	Cost	
	(Rupees in '000)	
**Non Government Debt Securities**		
**Listed**		
- AA+, AA, AA-	1,676,840	1,086,840
- A+, A, A-	75,000	150,000
	1,751,840	1,236,840
**Unlisted**		
- AA+, AA, AA-	990,000	990,000
- A+, A, A-	100,000	201,933
	1,090,000	1,191,933

	2020		2019	
	Cost	Rating	Cost	Rating
	(Rupees in '000)			
**Foreign Securities**				
**Government Securities**				
- Bahrain	–	–	–	–
- Sri Lanka	7,456,679	Caa1	3,649,770	B2
	7,456,679		3,649,770	

	2020	2019
	Cost	
	(Rupees in '000)	
**Unlisted**		
Lanka Clear (Private) Limited	857	854
Credit Information Bureau of Sri Lanka	26	26
Lanka Financial Services Bureau Limited	1,714	1,707
Society for Worldwide Inter Fund Transfer (SWIFT)	4,663	4,663
	7,260	7,250

10.5 Particulars relating to Held to Maturity securities are as follows:

	2020	2019
**Federal Government Securities - Government guaranteed**		
Pakistan Investment Bonds	3,333,719	13,842,253
Market Treasury Bills	–	1,645,036
Sukuk bonds	523,680	195,928
Euro Bonds	754,991	524,032
Bai Mu'ajjal	2,700,827	2,700,827
	7,313,217	18,908,076
**Provincial Government Securities**	118	118

Notes To The Consolidated Financial Statements

For the year ended December 31, 2020

	2020	2019
	Cost	
	(Rupees in '000)	
**Non Government Debt Securities**		
**Listed**		
- AAA	1,796,250	1,796,970
- AA+, AA, AA-	1,989,520	2,700,716
- Unrated	66,120	66,120
	3,851,890	4,563,806
**Unlisted**		
- AA+, AA, AA-	4,849,483	4,848,863
- A+, A, A-	439,874	439,910
- Unrated	474,070	590,346
	5,763,427	5,879,119

	2020		2019	
	Cost	Rating	Cost	Rating
	(Rupees in '000)			
**Foreign Securities**				
**Government Securities**				
- Sri Lanka	3,149,647	Caa1	1,544,542	B2
- United Arad Emirates	–		6,323,490	Aa2
	3,149,647		7,868,032	

	2020	2019
	Cost	
	(Rupees in '000)	
**Non Government Debt Securities**		
**Listed**		
- A+, A, A-	–	153,702
**Unlisted**		
- AAA	–	298,865
	–	298,865

10.5.1 The market value of securities classified as held-to-maturity as at December 31, 2020 amounted to Rs. 17,351.392 million (December 31, 2019: Rs. 34,043.148 million).

10.6 "Available for sale" Market Treasury Bills and Pakistan Investment Bonds are eligible for rediscounting with the State Bank of Pakistan.

10.7 Investments include Pakistan Investment Bonds amounting to Rs. 67.9 million (2019: Rs. 67.9 million) earmarked by the SBP against TT discounting facilities sanctioned to the Bank. In addition, Pakistan Investment Bonds amounting to Rs. 5 million (2019: Rs. 5 million) have been pledged with the Controller of Military Accounts on account of Regimental Fund account and Pakistan Investment Bonds amounting to Rs.100 million (2019: Rs. Rs. 100 million) have been pledged with the National Clearing Company of Pakistan Limited (NCCPL) on account of removal of irrevocable undertaking as alternate option for collateral against participant's exposure in stock market.

Notes To The Consolidated Financial Statements

For the year ended December 31, 2020

10.8 Certain approved / Government securities are kept with the SBP to meet statutory liquidity requirements calculated on the basis of domestic demand and time liabilities.

10.9 Investment of the Group in Adamjee Insurance Company Limited has been accounted for under the equity method of accounting in accordance with the treatment specified in International Accounting Standard 28, (IAS 28) 'Accounting for Investments in Associates'. The market value of the investment in Adamjee Insurance Company Limited as at December 31, 2020 amounted to Rs. 2,752.400 million. (2019: Rs. 2,946.300 million).

Investment in Adamjee Insurance Company Limited under equity method - holding 20.00% (2019: 20%)

	2020	2019
	(Rupees in '000)	
**Opening balance**	4,211,707	3,959,039
Share of profit for the year before tax	585,968	326,755
Dividend from associate	(192,500)	(175,000)
Share of tax	(143,175)	(109,675)
	250,293	42,080
Share of other comprehensive income	(26,925)	210,588
Closing balance	4,435,075	4,211,707
**Share of other comprehensive income**		
Share of unrealized (deficit) / surplus on assets -net of tax	(34,246)	167,219
Share of exchange translation reserve of associate	7,321	43,369
	(26,925)	210,588

10.10 Investment of the Group in Euronet Pakistan Private Limited has been accounted for under the equity method of accounting in accordance with the treatment specified in International Accounting Standard 28, (IAS 28) 'Accounting for Investments in Associates'.

Investment in Euronet Pakistan Private Limited under equity method - holding 30% (2019: 30.00%)

	2020	2019
	(Rupees in '000)	
Opening balance	63,951	60,487
Share of (loss) / profit for the year before tax	(12,890)	12,878
Share of tax	(6,776)	(9,414)
Closing balance	(19,666)	3,464
	44,285	63,951

Notes To The Consolidated Financial Statements

For the year ended December 31, 2020

10.11 Summarized financial information of associates

	Country of incorporation	% of interest held	Revenue	Profit/(loss) after tax	Assets	Liabilities
			(Rupees in '000)			
**2020**						
**Associates**						
Euronet Pakistan (Private) Limited (unaudited based on December 31, 2020)	Pakistan	30%	718,076	(59,342)	870,136	722,516
Adamjee Insurance Company Limited (unaudited based on September 30, 2020)	Pakistan	20%	20,596,203	1,319,951	95,997,472	74,918,209
**2019**						
**Associates**						
Euronet Pakistan (Private) Limited (unaudited based on December 31, 2019)	Pakistan	30%	557,579	25,769	648,477	435,306
Adamjee Insurance Company Limited (unaudited based on September 30, 2019)	Pakistan	20%	19,551,861	1,108,984	82,967,479	63,892,816

11. ADVANCES

	Note	Performing		Non Performing		Total	
		2020	2019	2020	2019	2020	2019
		(Rupees in '000)					
Loans, cash credits, running finances, etc.	11.1	444,022,138	471,613,945	50,524,753	48,759,157	494,546,891	520,373,102
Islamic financing and related assets		84,205,962	50,965,243	756,471	381,427	84,962,433	51,346,670
Bills discounted and purchased		18,192,157	19,486,758	664,294	665,102	18,856,451	20,151,860
Advances - gross		546,420,257	542,065,946	51,945,518	49,805,686	598,365,775	591,871,632
Provision against advances							
- Specific		–	–	(45,168,351)	(41,937,761)	(45,168,351)	(41,937,761)
- General	11.4.4	(5,511,716)	(1,461,011)	–	–	(5,511,716)	(1,461,011)
		(5,511,716)	(1,461,011)	(45,168,351)	(41,937,761)	(50,680,067)	(43,398,772)
Advances - net of provision		540,908,541	540,604,935	6,777,167	7,867,925	547,685,708	548,472,860

11.1 Includes net investment in finance lease as disclosed below:

	2020				2019			
	Not later than one year	Late than one and less than five years	Over five year	Total	Not later than one year	Late than one and less than five years	Over five year	Total
	(Rupees in '000)							
Lease rentals receivable	2,022,558	1,835,337	1,133,794	4,991,689	623,319	2,500,339	2,365,197	5,488,855
Residual value	20,428	103,490	23,039	146,957	14,769	35,544	10,368	60,681
Minimum lease payments	2,042,986	1,938,827	1,156,833	5,138,646	638,088	2,535,883	2,375,565	5,549,536
Financial charges for future periods	(99,749)	(309,852)	(418,859)	(828,460)	(136,416)	(319,293)	(517,870)	(973,579)
Present value of minimum lease payments	1,943,237	1,628,975	737,974	4,310,186	501,672	2,216,590	1,857,695	4,575,957

Notes To The Consolidated Financial Statements

For the year ended December 31, 2020

	2020	2019
	(Rupees in '000)	
**11.2 Particulars of advances (Gross)**		
In local currency	554,179,378	532,760,442
In foreign currencies	44,186,397	59,111,190
	598,365,775	591,871,632

11.3 Advances include Rs. 51,945.518 million (2019: Rs. 49,805.686 million) which have been placed under the non-performing status as detailed below:

	Note	2020 Non performing loans	2020 Provision	2019 Non performing loans	2019 Provision
		(Rupees in '000)			
**Category of Classification**					
**Domestic**					
Other Assets Especially Mentioned	11.3.1	61,612	1,983	138,432	3,529
Substandard		309,191	60,382	591,832	146,872
Doubtful		459,053	137,341	3,048,946	1,346,099
Loss		42,671,220	41,498,157	37,836,484	37,088,134
		43,501,076	41,697,863	41,615,694	38,584,634
**Overseas**					
Not past due but impaired		–	–	–	–
Overdue by:		–	–	–	–
Upto 90 days		5,321	4,913	10,688	7,400
91 to 180 days		2,020	505	118,182	95,452
181 to 365 days		19,961	9,981	141,231	137,601
65 days		8,417,140	3,455,089	7,919,891	3,112,674
		8,444,442	3,470,488	8,189,992	3,353,127
Total		51,945,518	45,168,351	49,805,686	41,937,761

11.3.1 This represents non-performing portfolio of agricultural and small enterprise financing classified as OAEM as per the requirements of the Prudential Regulation for Agricultural and Small Enterprise Financing issued by the State Bank of Pakistan.

11.4 Particulars of provision against advances

	Note	2020 Specific	2020 General	2020 Total	2019 Specific	2019 General	2019 Total
		(Rupees in '000)					
Opening balance		41,937,761	1,461,011	43,398,772	41,944,267	1,292,113	43,236,380
Exchange adjustments		50,555	7,162	57,717	302,297	23,426	325,723
Charge for the year		5,730,579	4,106,594	9,837,173	3,359,542	174,710	3,534,252
Reversals	11.4.3	(2,215,829)	(63,051)	(2,278,880)	(3,649,841)	(29,238)	(3,679,079)
		3,514,750	4,043,543	7,558,293	(290,299)	145,472	(144,827)
Amounts written off	11.5	(334,715)	–	(334,715)	(18,504)	–	(18,504)
Closing balance		45,168,351	5,511,716	50,680,067	41,937,761	1,461,011	43,398,772

Notes To The Consolidated Financial Statements

For the year ended December 31, 2020

11.4.1 Particulars of provision against advances

	Note	2020 Specific	2020 General	2020 Total	2019 Specific	2019 General	2019 Total
		(Rupees in '000)					
In local currency		41,697,863	5,189,680	46,887,543	38,244,450	1,120,827	39,365,277
In foreign currencies		3,470,488	322,036	3,792,524	3,693,311	340,184	4,033,495
		45,168,351	5,511,716	50,680,067	41,937,761	1,461,011	43,398,772

11.4.2 State Bank of Pakistan vide BSD Circular No. 2 dated January 27, 2009, BSD Circular No. 10 dated October 20, 2009, BSD Circular No. 02 of 2010 dated June 03, 2010 and BSD Circular No.1 of 2011 dated October 21, 2011 has allowed benefit of forced sale value (FSV) of Plant and Machinery under charge, pledged stock and mortgaged residential, commercial and industrial properties (land and building only) held as collateral against NPLs for five years from the date of classification. The Bank (holding company) has not taken the FSV benefit in calculation of specific provision. However, one of the subsidiaries of the Bank has availed benefit of forced sale values amounting to Rs. 543.151 million (December 31, 2019: Rs. 178.374 million) in determining the provisioning against non-performing Islamic financing and related assets as at December 31, 2020. The additional benefit on the Bank's statement of profit and loss arising from availing the FSV benefit - net of tax amounts to Rs. 353.048 million as at December 31, 2020 (December 31, 2019: Rs. 115.943 million). However, the additional impact on profitability arising from availing the benefit of forced sales value is not available for payment of cash or stock dividends to shareholders.

11.4.3 This includes reversal of provisions and reduction of non-performing loans amounting to Rs. 84 million (2019: Rs. NIL) as a result of settlement on debt asset swap arrangement with customers.

11.4.4 General provision against consumer loans represents provision maintained against fully secured performing portfolio and unsecured performing portfolio as required by the Prudential Regulations issued by the SBP. General provision against Small Enterprise Finance represents provision maintained at an amount equal to 1% of unsecured performing portfolio as required by the Prudential Regulations issued by the SBP. General provisions pertaining to overseas advances are made in accordance with the requirements of the regulatory authorities of the respective countries in which the overseas branches operate.

In addition, the Group has also maintained a general provision of Rs. 4,564.689 million (December 31, 2019: Rs. 527 million) against financing made on prudent basis. This general provision is in addition to the requirements of Prudential Regulations. Refer note 5 to these consolidated financial statements.

		Note	2020	2019
			(Rupees in '000)	
**11.5**	**PARTICULARS OF WRITE OFFs:**			
**11.5.1**	Against Provisions	11.4	334,715	18,504
	Directly charged to Profit & Loss account		–	–
			334,715	18,504
**11.5.2**	Write Offs of Rs. 500,000 and above			
	- Domestic	11.6	299,532	14,613
	- Overseas		–	2,725
	Write Offs of below Rs. 500,000		35,183	1,166
			334,715	18,504

Notes To The Consolidated Financial Statements
For the year ended December 31, 2020

11.6 DETAILS OF LOAN WRITE OFF OF Rs. 500,000/- AND ABOVE

In terms of sub-section (3) of Section 33A of the Banking Companies Ordinance, 1962, the statement in respect of written-off loans or any other financial relief of Rupees five hundred thousand or above allowed to a person(s) during the year ended December 31, 2020 is given at Annexure- I of unconsolidated financial statements. However, this write off does not affect the Bank's right to recover the debts from these customers.

	Note	2020	2019
		(Rupees in '000)	
**12. FIXED ASSETS**			
Capital work-in-progress	12.1	871,274	1,068,429
Property and equipment	12.2	53,190,768	52,466,540
Right-of-use assets	12.3	9,617,270	10,666,838
		63,679,312	64,201,807

12.1 Capital work-in-progress

	2020	2019
Civil works	472,314	476,799
Equipment	98,383	90,946
Advances to suppliers	296,456	491,968
Others	4,121	8,716
	871,274	1,068,429

12.2 Property and Equipment

	2020								
	Freehold land	Leasehold land	Building on freehold land	Building on leasehold land	Furniture and fixtures	Electrical, office and computer equipment	Vehicles	Leasehold improvements	Total
	(Rupees in '000)								
**At January 1, 2020**									
Cost / Revalued amount	26,951,665	2,893,079	14,400,594	637,102	2,047,789	15,989,634	1,176,389	1,352,983	65,449,235
Accumulated depreciation	–	–	(236)	–	(1,159,800)	(10,690,712)	(643,336)	(488,611)	(12,982,695)
Net book value	26,951,665	2,893,079	14,400,358	637,102	887,989	5,298,922	533,053	864,372	52,466,540
**Year ended December 31, 2020**									
Opening net book value	26,951,665	2,893,079	14,400,358	637,102	887,989	5,298,922	533,053	864,372	52,466,540
Additions	189,607	6,999	976,836	168,601	228,078	1,174,108	60,767	443,560	3,248,556
Disposals	–	–	(85,546)		(2,579)	(6,910)	(23,938)	(1,300)	(120,273)
Depreciation charge	–	–	(498,884)	(28,597)	(172,075)	(1,345,944)	(125,219)	(232,501)	(2,403,220)
Impairment	–	–	–	–	(870)	(561)	–	(2,838)	(4,269)
Exchange rate adjustments	–	–	412	691	773	1,294	1,987	(1,723)	3,434
Transfers	–	–	(17,242)	–	61	(61)	–	17,242	-
Closing net book value	27,141,272	2,900,078	14,775,934	777,797	941,377	5,120,848	446,650	1,086,812	53,190,768
**At December 31, 2020**									
Cost / Revalued amount	27,141,272	2,900,078	15,273,596	806,387	2,239,957	16,995,598	1,120,324	1,775,260	68,252,472
Accumulated depreciation	–	–	(497,662)	(28,590)	(1,297,854)	(11,874,750)	(673,674)	(681,889)	(15,054,419)
Accumulated Impairment	–	–	–	–	(726)	–	–	(6,559)	(7,285)
Net book value	27,141,272	2,900,078	14,775,934	777,797	941,377	5,120,848	446,650	1,086,812	53,190,768
Rate of depreciation / estimated useful life	–	–	Upto 60 years	Upto 50 years	10%	10%-25%	20%	Lease term	-

Notes To The Consolidated Financial Statements

For the year ended December 31, 2020

	2019								
	Freehold land	Leasehold land	Building on freehold land	Building on leasehold land	Furniture and fixtures	Electrical, office and computer equipment	Vehicles	Leasehold improvements	Total
	(Rupees in '000)								
**At January 1, 2019**									
Cost / Revalued amount	20,287,003	2,440,151	12,602,260	563,273	1,831,107	15,115,272	1,231,531	870,429	54,941,026
Accumulated depreciation	–	–	(881,516)	(116,820)	(1,025,554)	(9,887,641)	(638,772)	(361,128)	(12,911,431)
Net book value	20,287,003	2,440,151	11,720,744	446,453	805,553	5,227,631	592,759	509,301	42,029,595
**Year ended December 31, 2019**									
Opening net book value	20,287,003	2,440,151	11,720,744	446,453	805,553	5,227,631	592,759	509,301	42,029,595
Additions	806,735	–	1,989,250	142,646	237,979	1,414,512	123,397	536,351	5,250,870
Movement in surplus on assets revalued during the year	5,840,577	452,928	1,073,211	73,227	–		–	–	7,439,943
Transfer from Non-Banking assets	34,000	–	17,520	–	–	–	–	–	51,520
Disposal	(21,000)	–	–	–	(6,202)	(5,101)	(40,683)	(31,212)	(104,198)
Depreciation charge	–	–	(417,054)	(27,778)	(151,530)	(1,346,139)	(146,347)	(144,160)	(2,233,008)
Exchange rate adjustments	–	–	9,566	2,554	3,438	6,770	3,927	5,563	31,818
Transfers	4,350	–	7,121	–	(1,249)	1,249	–	(11,471)	–
Closing net book value	26,951,665	2,893,079	14,400,358	637,102	887,989	5,298,922	533,053	864,372	52,466,540
**At December 31, 2019**									
Cost / Revalued amount	26,951,665	2,893,079	14,400,594	637,102	2,047,789	15,989,634	1,176,389	1,352,983	65,449,235
Accumulated depreciation	–	–	(236)	–	(1,159,800)	(10,690,712)	(643,336)	(488,611)	(12,982,695)
Net book value	26,951,665	2,893,079	14,400,358	637,102	887,989	5,298,922	533,053	864,372	52,466,540
Rate of depreciation / estimated useful life	–	–	Upto 60 years	Upto 50 years	10%	10%-25%	20%	Lease term	–

12.2.1 Leasehold land include a plot of land measuring 3,120.46 square yards having book value of Rs. 1,419.809 million situated at Railway Quarters, I.I. Chundrigar Road, Karachi, (the “Plot”), where a tenant is claiming for the possession as tenant of an insignificant area of only 18 square feet of the plot, however there is no issue over the title of the subject property. Both the Constitutional Petitions filed by the Group have been dismissed by the Sindh High Court on 28 January 2016 against the Group. The Group has filed an appeal before the Supreme Court of Pakistan.

12.2.2 The land and buildings of the Group were revalued as at December 31, 2019 by independent valuers (K.G. Traders (Pvt) Limited, Tristar International Consultant (Pvt) Limited & Sardar Enterprises), valuation and engineering consultants, on the basis of market value. The total surplus against revaluation of fixed assets as at December 31, 2020 amounts to Rs. 20,856.881 million.

12.2.3 Had the land and buildings not been revalued, the total carrying amounts of revalued properties as at December 31, 2020 would have been as follows:

	(Rupees in '000)
Land	13,007,065
Buildings	11,731,135

12.2.4 The gross carrying amount (cost) of fully depreciated assets that are still in use are as follows:

	(Rupees in '000)
Furniture and fixtures	608,625
Electrical, computers and office equipment	7,347,691
Vehicles	488,175
Lease Hold Improvements	35,697

Notes To The Consolidated Financial Statements
For the year ended December 31, 2020

12.2.5 Carrying amount of temporarily idle property of the Group is Rs. 436.136 million (2019: Rs. 161.886 million)

12.2.6 The information relating to disposal of operating fixed assets to related parties is given in Annexure I of these consolidated financial statements.

12.3 Movement in right-of-use assets is as follows:

	Note	2020	2019
		(Rupees in '000)	
Opening balance		10,666,838	11,392,795
Additions / Adjustments		1,263,307	884,936
Derecognition		(615,049)	–
Depreciation charge	31	(1,697,826)	(1,610,893)
Closing net book value		9,617,270	10,666,838

13. INTANGIBLE ASSETS

	Note	2020	2019
Capital work-in-progress		435,330	472,336
Goodwill		82,127	82,127
Management rights		192,000	192,000
Computer software	13.1	1,157,787	1,232,512
		1,867,244	1,978,975

	Note	2020	2019
		(Rupees in '000)	
		Computer software	
**13.1 At January 1**			
Cost		4,865,584	4,114,222
Accumulated amortisation		(3,633,072)	(3,193,692)
Net book value		1,232,512	920,530
**Year ended December 31**			
Opening net book value		1,232,512	920,530
Additions		395,186	734,933
Amortisation charge	31	(469,860)	(425,585)
Exchange rate adjustments		(51)	2,634
Closing net book value		1,157,787	1,232,512
**At December 31**			
Cost		5,262,979	4,865,584
Accumulated amortisation and impairment		(4,105,192)	(3,633,072)
Net book value		1,157,787	1,232,512
Rate of amortisation (percentage)		14% to 33.33%	14% to 33.33%
Useful life		3 - 7 years	3 - 7 years

13.2 The gross carrying amount (cost) of fully depreciated intangible assets that are still in use is Rs. 3,085.368 million.

Notes To The Consolidated Financial Statements
For the year ended December 31, 2020

		Note	2020	2019
			(Rupees in '000)	
**14.**	**OTHER ASSETS**			
	Income/ mark-up accrued in local currency		19,451,779	24,533,964
	Income/ mark-up accrued in foreign currencies		311,024	564,667
	Advances, deposits, advance rent and other prepayments		2,524,851	3,940,446
	Compensation for delayed income tax refunds		133,809	133,809
	Non-banking assets acquired in satisfaction of claims	14.1	3,277,778	3,251,508
	Branch adjustment account		421,204	77,281
	Mark to market gain on forward foreign exchange contracts		4,847,284	3,848,188
	Unrealized gain on derivative financial instruments	25	517,033	1,236,517
	Acceptances	20	22,747,369	20,346,205
	Receivable from the pension fund	38.4	3,370,179	3,605,121
	Others		6,919,936	10,118,430
			64,522,246	71,656,136
	Less: Provision held against other assets	14.2	2,582,686	2,604,137
	Other Assets (net of provision)		61,939,560	69,051,999
	Surplus on revaluation of non-banking assets acquired in satisfaction of claims		854,231	677,660
	Other Assets - total		62,793,791	69,729,659
**14.1**	Market value of Non-banking assets acquired in satisfaction of claims		4,036,914	3,838,230

Non-banking assets acquired in satisfaction of claims of the Bank are revalued as at December 31, 2020 by independent valuers (Tristar International Consultant, Design Crafts, Material Design Services, Pee Dee & Associates and J&M Associates) on the basis of market value.

		Note	2020	2019
			(Rupees in '000)	
**14.1.1**	**Non-banking assets acquired in satisfaction of claims**			
	Opening balance		3,838,230	4,035,114
	Additions		84,000	64,445
	Revaluation		183,915	255,040
	Disposals		(35,024)	(428,052)
	Depreciation		(30,049)	(45,456)
	(Charge) / reversal of impairment		(4,158)	8,659
	Transfer to fixed assets		–	(51,520)
	Closing balance		4,036,914	3,838,230
**14.1.2**	**Gain on disposal of non-banking assets acquired in satisfaction of claims**			
	Disposal proceeds		39,000	540,000
	Less			
	- Cost		35,820	433,813
	- Depreciation		(796)	(5,761)
			35,024	428,052
	Gain	14.1.2.1	3,976	111,948

	Note	2020	2019
		(Rupees in '000)	
**14.1.2.1 Breakup of gain/loss realized on sale of non-banking assets:**			
Commercial property Rawalpindi		3,976	–
Industrial property Lahore		–	111,948
		3,976	111,948
**14.2 Provision held against other assets**			
Non banking assets acquired in satisfaction of claims		95,095	90,938
Others		2,487,591	2,513,199
		2,582,686	2,604,137
**14.2.1 Movement in provision held against other assets**			
Opening balance		2,604,137	2,550,585
Charge for the year		54,269	12,587
Reversals		(77,917)	(36,023)
	33	(23,648)	(23,436)
Amount written off		(16,591)	(3,638)
Exchange and other adjustments		18,788	80,626
Closing balance		2,582,686	2,604,137

15. CONTINGENT ASSETS

There were no contingent assets of the Group as at December 31, 2020 (2019: NIL).

	Note	2020	2019
		(Rupees in '000)	
**16. BILLS PAYABLE**			
In Pakistan		26,383,624	12,759,834
Outside Pakistan		67,889	35,491
		26,451,513	12,795,325
**17. BORROWINGS**			
**Secured**			
Borrowings from State Bank of Pakistan			
Under export refinance scheme	17.1	37,844,720	36,513,083
Under long term financing facility	17.2	22,596,183	18,138,200
Under renewable energy performance platform	17.3	74,760	85,062
Refinance Scheme for wages and salaries	17.4	11,789,824	–
Temporary economic refinance facility	17.5	2,878,487	–
Under financing facility for storage of agricultural produce	17.6	191,254	188,809
		75,375,228	54,925,154
Repurchase agreement borrowings	17.7	92,225,530	28,099,229
**Total secured**		167,600,758	83,024,383
**Unsecured**			
Borrowings from other financial institution	17.8	1,720,341	889,161
Call borrowings	17.9	319,669	5,964,830
Overdrawn nostro accounts		368,920	1,342,603
Musharaka arrangements	17.10	14,405,366	1,476,705
Others		162,286	162,286
**Total unsecured**		16,976,582	9,835,585
	17.11	184,577,340	92,859,968

Notes To The Consolidated Financial Statements
For the year ended December 31, 2020

17.1 The Group has entered into agreements for financing with the State Bank of Pakistan (SBP) for extending export finance to customers. As per the agreements, the Group has granted SBP the right to recover the outstanding amount from the Group at the date of maturity of the finance by directly debiting the current account maintained by the Bank with SBP. These borrowings are repayable within six months. These carry mark up rates ranging from 1.0% to 2.0% per annum.

17.2 These borrowings have been obtained from the SBP for providing financing facilities to exporters for adoption of new technologies and modernization of their plant and machinery. These borrowings are repayable within a period ranging from 3 years to 10 years. These carry mark up rates ranging from 2.0% to 3.50% per annum.

17.3 These borrowings have been obtained from the SBP for providing financing facilities to customers against renewable energy projects. These borrowings are repayable within a period of twelve years with two years grace period from date of disbursement. These carry mark up rate of 2% per annum.

17.4 These borrowings have been obtained from the SBP for providing financing facilities to help businesses in payment of wages and salaries to their workers and employees for supporting continued employment. These borrowing are repayable in 8 equal quarterly instalments begining from January 2021. These carry mark up rates ranging from 0% to 2% per annum.

17.5 These borrowings have been obtained from the SBP for providing concessionary refinancing facility to the industry for purchase of new imported and locally manufactured plant & machinery to set up new projects. These borrowings are repayable within a period of ten years including a grace period of upto 2 years. These carry mark up rate of 1% per annum.

17.6 These borrowings have been obtained from SBP under “Financing Facility for Storage of Agricultural Produce (FFSAP)” to encourage Private Sector to establish Silos, Warehouses and Cold Storages. These borrowings are repayable within a period ranging from 3 years to 10 years. These carry mark up rates ranging from 2.50% to 3.50% per annum.

17.7 These carry mark-up rates ranging from 6.15% to 7.25% per annum (2019:8.0% to 13.20% per annum) and are secured against government securities of carrying value of Rs. 92,279.556 million (2019: Rs. 28,136.434 million). These are repayable latest by February 2021.

17.8 These carry mark-up rates ranging from 1.90% to 4.00% per annum (2019: 2.75% to 3.00% per annum). These are repayable latest by August 2022.

17.9 These carry mark-up at the rate of 1.15% per annum (2019: 2.30% to 13.10%per annum). These are repayable by January 2021.

17.10 These carry mark-up ranging from 6.7% to 7.2% per annum (2019: 8.5% to 9.0% per annum). These are repayable by March 2021.

	2020	2019
	(Rupees in '000)	
**17.11 Particulars of borrowings with respect to currencies**		
In local currency	182,091,272	88,699,245
In foreign currencies	2,486,068	4,160,723
	184,577,340	92,859,968

Notes To The Consolidated Financial Statements

For the year ended December 31, 2020

18. DEPOSITS AND OTHER ACCOUNTS

	2020			2019		
	In local currency	In foreign currencies	Total	In local currency	In foreign currencies	Total
	(Rupees in '000)					
**Customers**						
Current deposits	425,760,845	55,999,023	481,759,868	357,303,848	53,340,189	410,644,037
Savings deposits	689,241,146	47,759,213	737,000,359	583,751,817	47,420,170	631,171,987
Term deposits	89,353,999	14,182,837	103,536,836	100,346,882	15,590,901	115,937,783
Others	26,013,457	3,218,232	29,231,689	26,456,179	2,854,010	29,310,189
	1,230,369,447	121,159,305	1,351,528,752	1,067,858,726	119,205,270	1,187,063,996
**Financial Institutions**						
Current deposits	11,033,694	659,230	11,692,924	9,520,075	2,094,703	11,614,778
Savings deposits	16,530,222	117,569	16,647,791	14,992,263	27,986	15,020,249
Term deposits	4,557,468	4,090,613	8,648,081	4,959,099	7,741,444	12,700,543
Others	–	220,413	220,413	–	193,459	193,459
	32,121,384	5,087,825	37,209,209	29,471,437	10,057,592	39,529,029
	1,262,490,831	126,247,130	1,388,737,961	1,097,330,163	129,262,862	1,226,593,025

	2020	2019
	(Rupees in '000)	
**18.1** Composition of deposits		
- Individuals	873,722,061	751,908,455
- Government (Federal and Provincial)	60,113,810	73,275,909
- Public Sector Entities	87,102,803	52,949,106
- Banking Companies	4,893,635	10,219,893
- Non-Banking Financial Institutions	32,298,388	29,285,707
- Private Sector	330,607,264	308,953,955
	1,388,737,961	1,226,593,025

18.2 Deposits and other accounts include deposits eligible to be covered under the Deposits Protection insurance arrangements amounting to Rs 957,118.596 million (2019: Rs 802,238.351 million).

Notes To The Consolidated Financial Statements

For the year ended December 31, 2020

19. DEFERRED TAX LIABILITIES

	Note	2020			
		At January 01, 2020	Recognised in P&L A/C	Recognised in OCI	At December 01, 2020
		(Rupees in '000)			
**Taxable Temporary Differences on**					
- Surplus on revaluation of fixed assets	23.1	1,390,542	(61,031)	–	1,329,511
- Surplus on revaluation of Non-banking assets	23.2	237,181	(2,570)	64,371	298,982
- Accelerated tax depreciation		1,961,113	93,396	–	2,054,509
- Receivable from pension fund		1,261,793	119,428	(201,659)	1,179,562
- Business combination		705,218	–	–	705,218
- Investments in associated undertaking		1,247,340	87,603	(9,423)	1,325,520
- Surplus/deficit on revaluation of investments	23	2,329,519	–	2,193,979	4,523,498
		9,132,706	236,826	2,047,268	11,416,800
**Deductible Temporary Differences on**					
- Tax losses carried forward	19.1	(949,800)	177,443	–	(772,357)
- Provision against advances		(1,726,151)	(1,060,705)	–	(2,786,856)
- Others		(242,532)	(124,015)		(366,547)
		(2,918,483)	(1,007,277)	–	(3,925,760)
		6,214,223	(770,451)	2,047,268	7,491,040

	Note	2019			
		At January 01, 2019	Recognised in P&L A/C	Recognised in OCI	At December 01, 2019
		(Rupees in '000)			
**Taxable Temporary Differences on**					
- Surplus on revaluation of fixed assets	23.1	1,018,502	(29,214)	401,254	1,390,542
- Surplus on revaluation of non-banking assets	23.2	168,305	(20,388)	89,264	237,181
- Accelerated tax depreciation		1,810,834	150,279	–	1,961,113
- Receivable from pension fund		1,335,309	155,621	(229,137)	1,261,793
- Business combination		705,218	–	–	705,218
- Investments in associated undertaking		1,158,906	14,728	73,706	1,247,340
- Surplus/deficit on revaluation of investments	23	–	–	2,329,519	2,329,519
		6,197,074	271,026	2,664,606	9,132,706
**Deductible Temporary Differences on**					
- Tax losses carried forward	19.1	(818,652)	(131,148)	–	(949,800)
- Provision against advances		(1,791,768)	65,617	–	(1,726,151)
- Others		(92,755)	(149,777)	–	(242,532)
- Surplus/deficit on revaluation of investments		(1,510,754)	–	1,510,754	–
		(4,213,929)	(215,308)	1,510,754	(2,918,483)
		1,983,145	55,718	4,175,360	6,214,223

Notes To The Consolidated Financial Statements

For the year ended December 31, 2020

19.1 This includes deferred tax asset recorded on unused tax losses and minimum tax of one of the subsidiaries of the Group. The subsidiary's management, based on their internal projections / assessment of future taxable profits, believes that the subsidiary will be able to achieve sufficient taxable profits and tax liability in future to adjust unused tax losses and minimum tax before their expiry and accordingly has recorded the deferred tax asset on such basis. Tax losses and minimum tax have expiry dates between 2023 to 2025.

	Note	2020	2019
		(Rupees in '000)	
**20. OTHER LIABILITIES**			
Mark-up/ return/ interest payable in local currency		2,884,255	23,511,019
Mark-up/ return/ interest payable in foreign currencies		254,433	664,130
Unearned commission and income on bills discounted		252,312	252,842
Accrued expenses		6,888,435	6,310,392
Provision for taxation (provisions less payments)		10,130,229	6,045,948
Workers' welfare fund	20.1	8,838,684	7,873,706
Acceptances	14	22,747,369	20,346,205
Unclaimed/dividend payable		1,697,408	1,605,123
Mark to market loss on forward foreign exchange contracts		4,618,138	4,638,011
Unrealized loss on derivative financial instruments	25	513,343	1,232,806
Staff welfare fund		5,598	5,727
Provision for employees' compensated absences	38.4	919,407	939,495
Provision for post retirement medical benefits	38.4	2,004,122	1,921,348
Provision for employees' contributory benevolent scheme	38.4	222,084	221,193
Retention money		20,657	20,657
Insurance payable against consumer assets		698,949	655,146
Unclaimed balances		877,552	993,105
Duties and taxes payable		1,860,730	775,694
Charity fund balance		46,615	54,782
Provision against off-balance sheet obligations		46,188	46,581
Security deposits against lease		1,283,999	1,497,296
Lease liability against right of use assets		11,268,508	11,436,633
Others		12,948,143	11,357,674
		91,027,158	102,405,513

20.1 Supreme Court of Pakistan vide its order dated November 10, 2016 has held that the amendments made in the law introduced by the Federal Government for the levy of Workers Welfare Fund were not lawful. The Federal Board of Revenue has filed review petitions against this order which are currently pending.

Legal advice obtained on the matter indicates that consequent to filing of these review petitions the judgment may not currently be treated as conclusive. Accordingly, the Bank maintained its provision in respect of WWF.

21. SHARE CAPITAL

21.1 Authorized Capital

2020	2019		2020	2019
(Number of shares)			(Rupees in '000)	
1,500,000,000	1,500,000,000	Ordinary shares of Rs. 10 each	15,000,000	15,000,000

Notes To The Consolidated Financial Statements

For the year ended December 31, 2020

21.2 Issued, subscribed and paid up

2020	2019		2020	2019
(Number of shares)			(Rupees in '000)	
		**Ordinary shares**		
197,253,795	197,253,795	Fully paid in cash	1,972,538	1,972,538
915,776,953	915,776,953	Issued as bonus shares	9,157,769	9,157,769
72,029,258	72,029,258	Issued for consideration other than cash	720,293	720,293
1,185,060,006	1,185,060,006		11,850,600	11,850,600

21.3 The movement in the issued, subscribed and paid-up capital during the year is as follows:

2020	2019		2020	2019
(Number of shares)			(Rupees in '000)	
1,185,060,006	1,185,060,006	Opening balance at January 1	11,850,600	11,850,600
1,185,060,006	1,185,060,006	Closing balance at December 31	11,850,600	11,850,600

21.4 Number of shares held by the associated undertakings as at December 31, are as follows:

	2020	2019
	Number of shares	
Adamjee Insurance Company Limited	47,827,287	47,827,287
Nishat Mills Limited	88,015,291	88,015,291
Siddiqsons Limited	14,276,462	14,276,462
Nishat (Aziz Avenue) Hotels and Properties Limited	141,950	29,000
Nishat Real Estates Development Company (Private) Limited	54,500	–
	150,315,490	150,148,040

	Note	2020	2019
		(Rupees in '000)	
**22. RESERVES**			
Share premium		23,973,024	23,973,024
Non- distributable capital reserve - gain on bargain purchase option	22.1	908,317	908,317
Exchange translation reserve		2,950,183	2,730,354
Statutory reserve	22.2	34,628,527	31,683,134
General reserve		18,600,000	18,600,000
		81,060,051	77,894,829

22.1 Under IFRS-3 a bargain purchase represents an economic gain which should be immediately recognized by the acquirer as income. However, the amount of bargain purchase gain was not taken to the profit and loss account as the SBP, through its letter BPRD(R&PD)/2017/14330 dated June 13, 2017 recommended that the amount of gain may be routed directly into equity as a Non-distributable Capital Reserve (NCR). The NCR may become available for distribution through a stock dividend only with prior approval of the SBP. The Group, before distribution of the gain as a stock dividend, may adjust any subsequent provisions/deficit, assessed by the Bank or recommended by the Banking Inspection Department of SBP, in the acquired assets and liabilities of NIB Bank Limited against the NCR.

22.2 Statutory reserve represents amount set aside as per the requirements of section 21 of the Banking Companies Ordinance, 1962.

Notes To The Consolidated Financial Statements

For the year ended December 31, 2020

	Note	2020	2019
		(Rupees in '000)	
**23. SURPLUS ON REVALUATION OF ASSETS**			
Surplus on revaluation of			
- Available for sale securities	10.1	12,924,291	6,655,770
- Fixed Assets	23.1	20,856,881	21,031,256
- Non-banking assets acquired in satisfaction of claims	23.2	854,231	677,660
- Associated undertaking		577,001	611,247
		35,212,404	28,975,933
Deferred tax on surplus on revaluation of:			
- Available for sale securities	19	4,523,498	2,329,519
- Fixed Assets	23.1	1,329,511	1,390,542
- Non-banking assets acquired in satisfaction of claims	23.2	298,982	237,181
- Associated undertaking		257,062	266,485
		6,409,053	4,223,727
		28,803,351	24,752,206

23.1 Surplus on revaluation of fixed assets

	2020	2019
Surplus on revaluation of fixed assets as at January 1	21,031,256	13,685,180
Recognised during the year	–	7,439,943
Realised on disposal during the year - net of deferred tax	(22,544)	(10,560)
Transferred to unappropriated profit in respect of incremental depreciation charged during the year - net of deferred tax	(90,800)	(54,093)
Related deferred tax liability on incremental depreciation charged during the year	(48,892)	(29,127)
Related deferred tax liability on surplus realised on disposal	(12,139)	(87)
Surplus on revaluation of fixed assets as at December 31	20,856,881	21,031,256
Less: Related deferred tax liability on:		
- revaluation as at January 1	1,390,542	1,018,502
- recognised during the year	–	401,254
- surplus realised on disposal during the year	(12,139)	(87)
- incremental depreciation charged during the year	(48,892)	(29,127)
	1,329,511	1,390,542
	19,527,370	19,640,714

23.2 Surplus on revaluation of non-banking assets acquired in satisfaction of claims

	2020	2019
Surplus on revaluation as at January 1	677,660	480,871
Recognised during the year	183,915	255,040
Realised on disposal during the year - net of deferred tax	(4,774)	(37,863)
Related deferred tax liability on surplus realised on disposal	(2,570)	(20,388)
Surplus on revaluation as at December 31	854,231	677,660
Less: Related deferred tax liability on:		
- revaluation as at January 1	237,181	168,305
- revaluation recognised during the year	64,371	89,264
- surplus realised on disposal during the year	(2,570)	(20,388)
	298,982	237,181
	555,249	440,479

Notes To The Consolidated Financial Statements

For the year ended December 31, 2020

	Note	2020	2019
		(Rupees in '000)	
**24. CONTINGENCIES AND COMMITMENTS**			
- Guarantees	24.1	186,572,634	180,002,174
- Commitments	24.2	525,404,920	657,101,777
- Other contingent liabilities	24.3	28,397,749	28,352,091
		740,375,303	865,456,042
**24.1 Guarantees:**			
Financial guarantees		149,925,920	141,181,839
Performance guarantees		29,835,397	33,833,099
Other guarantees		6,811,317	4,987,236
		186,572,634	180,002,174
**24.2 Commitments:**			
Documentary credits and short-term trade-related transactions			
- letters of credit		180,272,534	153,036,304
Commitments in respect of:			
- forward foreign exchange contracts	24.2.1	327,646,242	404,891,089
- forward government securities transactions	24.2.2	11,089,775	87,696,638
- derivatives	24.2.3	4,471,383	10,244,806
- commitments to extent credit		1,007,451	307,755
Commitments for acquisition of:			
- operating fixed assets		710,570	859,953
- intangible assets		206,965	65,232
		525,404,920	657,101,777
**24.2.1 Commitments in respect of forward foreign exchange contracts**			
Purchase		172,137,589	216,647,656
Sale		155,508,653	188,243,433
		327,646,242	404,891,089
**24.2.2 Commitments in respect of government securities transactions**			
Purchase		11,089,775	82,284,304
Sale		–	5,412,334
		11,089,775	87,696,638

Notes To The Consolidated Financial Statements

For the year ended December 31, 2020

		2020	2019
		(Rupees in '000)	
**24.2.3**	**Commitments in respect of derivatives**		
	FX options (notional)		
	Purchase	182,800	431,449
	Sale	182,800	431,449
		365,600	862,898
	Cross Currency Swaps (notional)		
	Purchase	1,975,311	4,428,663
	Sale	2,130,472	4,636,745
		4,105,783	9,065,408
	Interest Rate Swaps (notional)		
	Purchase	–	316,500
	Sale	–	–
		–	316,500
		4,471,383	10,244,806
**24.2.4**	**Other commitments**		
	Commitments to extent credit	1,007,451	307,755

24.2.5 Other than those mentioned above, the group makes commitments to extend credit in the normal course of its business but these being revocable commitments do not attract any significant penalty or expense if the facility is unilaterally withdrawn.

		Note	2020	2019
			(Rupees in '000)	
**24.3**	**Other contingent liabilities**			
	Claims against the Bank not acknowledged as debts	24.3.1	28,397,749	28,352,091

24.3.1 These represent certain claims by third parties against the Group, which are being contested in the Courts of law. The management is of the view that these relate to the normal course of business and the possibility of an outflow of economic resources is remote.

24.4 For assessment year 1988-89 through tax year 2019, the tax department disputed Group treatment on certain issues, wherein the Group's appeals are pending at various appellate forums, entailing an additional tax liability of Rs. 6,033 million (2019: Rs. 1,487 million). Such issues inter alia principally include disallowance of expenses for non deduction of withholding tax and non availability of underlying records, provision for non performing loans, attribution of expenses to heads of income other than income from business and disallowance of credit for taxes paid in advance / deducted at source.

The Group has filed appeals which are pending at various appellate forums. In addition, certain decisions made in favour of the Group are being contested by the department at higher forums. No provision has been made in the financial statements regarding the aforesaid additional tax demand and already issued favourable decisions where the department is in appeal, as the management is of the view that the issues will be decided in the Group's favour as and when these are taken up by the Appellate Authorities.

24.5 Amortisation of goodwill and other intangibles amounting to Rs 28.08 billion of Ex. NIB

Issue of goodwill and other related assets amortization for few years has been assessed in Bank's favour at different appellate forums during the year, however the tax department has filed appeal against these decisions. The management has not recorded any tax benefit because the issue has not attained finality.

Notes To The Consolidated Financial Statements

For the year ended December 31, 2020

25. DERIVATIVE INSTRUMENTS

25.1 Product Analysis

Counterparties	2020					
	Cross currency swaps		Interest rate swaps		FX option	
	Notional principal	Mark to market gain/loss	Notional principal	Mark to market gain/loss	Notional principal	Mark to market gain/loss
	(Rupees in '000)					
**With Banks for**						
Hedging	1,975,311	512,508	–	–	182,800	4,525
Market Making	–	–	–	–	–	–
**With other entities for**						
Hedging	–	–	–	–	–	–
Market Making	2,130,472	(508,818)	–	–	182,800	(4,525)
**Total**						
Hedging	1,975,311	512,508	–	–	182,800	4,525
Market Making	2,130,472	(508,818)	–	–	182,800	(4,525)

Counterparties	2019					
	Cross currency swaps		Interest rate swaps		FX option	
	Notional principal	Mark to market gain/loss	Notional principal	Mark to market gain/loss	Notional principal	Mark to market gain/loss
	(Rupees in '000)					
**With Banks for**						
Hedging	4,428,663	1,218,634	316,500	11,510	431,449	2,683
Market Making	–	–	–	–	–	–
**With other entities for**						
Hedging	-	-	–	–	–	–
Market Making	4,636,745	(1,226,433)	–	–	431,449	(2,683)
**Total**						
Hedging	4,428,663	1,218,634	316,500	11,510	431,449	2,683
Market Making	4,636,745	(1,226,433)	–	–	431,449	(2,683)

Notes To The Consolidated Financial Statements
For the year ended December 31, 2020

25.2 Maturity Analysis

	2020				
	No. of contracts	Notional principal	Mark to Market		
			Negative	Positive	Net
	(Rupees in '000)				
**Remaining Maturity**					
Upto 1 month	8	924,787	(54,638)	54,840	202
1 to 3 months	4	120,859	(1,545)	1,545	-
3 to 6 months	4	421,010	(71,467)	71,915	448
6 month to 1 Year	3	329,626	(23,640)	18,177	(5,463)
1 to 2 Year	4	1,094,545	(220,328)	226,567	6,239
2 to 3 Years	0	–	–	–	–
3 to 5 Years	2	1,580,556	(141,725)	143,989	2,264
**Total**	25	4,471,383	(513,343)	517,033	3,690

	2019				
	No. of contracts	Notional principal	Mark to Market		
			Negative	Positive	Net
	(Rupees in '000)				
**Remaining Maturity**					
Upto 1 month	14	409,597	(518)	518	–
1 to 3 months	32	1,076,111	(154,368)	154,707	339
3 to 6 months	1	52,745	(25,888)	–	(25,888)
6 month to 1 Year	7	1,847,480	(298,299)	299,689	1,390
1 to 2 Year	9	3,199,475	(312,482)	323,222	10,740
2 to 3 Years	4	1,714,098	(340,100)	353,553	13,453
3 to 5 Years	2	1,945,300	(101,151)	104,828	3,677
**Total**	69	10,244,806	(1,232,806)	1,236,517	3,711

25.3 Disclosure relating to qualitative and quantitative information on exchange traded derivatives are disclosed in note 45.5.

25.4 Risk management related to derivatives is discussed in note 45.5.

	2020	2019
	(Rupees in '000)	
**26. MARK-UP/RETURN/INTEREST EARNED**		
Loans and advances	50,061,684	64,738,017
Investments	94,248,954	77,292,876
Lendings to financial institutions	1,092,986	5,361,703
Balances with banks	368,827	696,104
	145,772,451	148,088,700

Notes To The Consolidated Financial Statements

For the year ended December 31, 2020

			2020	2019
			(Rupees in '000)	
**27.**	**MARK-UP/RETURN/INTEREST EXPENSED**			
	Deposits		59,436,050	70,280,198
	Borrowings		6,610,704	9,361,322
	Subordinated debt		–	213,604
	Cost of foreign currency swaps against foreign currency deposits / borrowings		2,386,644	3,009,184
	Unwinding cost of liability against right-of-use assets		1,495,614	1,506,301
			69,929,012	84,370,609
**28.**	**FEE & COMMISSION INCOME**			
	Branch banking customer fees		1,924,884	1,822,700
	Consumer finance related fees		431,539	350,591
	Card related fees (debit and credit cards)		3,206,215	3,134,235
	Credit related fees		60,484	188,443
	Investment banking fee		161,192	181,177
	Commission on trade		1,353,430	1,477,458
	Commission on guarantees		608,822	607,844
	Commission on cash management		615,001	612,178
	Commission on remittances including home remittances		1,020,949	1,172,359
	Commission on utility bills		79,791	106,320
	Commission income - Bancassurance		1,384,111	1,502,899
	Rent on lockers		230,996	245,866
	Commission on investments services		694,750	646,560
	Other Commission		199,327	182,241
			11,971,491	12,230,871
**29.**	**GAIN ON SECURITIES, NET**			
	Realised	29.1	3,286,027	744,887
	Unrealised - held for trading	10.1	110,269	83,986
			3,396,296	828,873
	**29.1** Realised gain on:			
	Federal Government Securities		3,438,493	17,685
	Non Government Debt Securities		(76,066)	–
	Shares & units		(38,094)	727,202
			3,324,333	744,887
**30.**	**OTHER INCOME**			
	Rent on property		67,310	69,427
	Gain on sale of fixed assets-net		73,164	63,176
	Loss on termination of lease liability against right of use assets		(15,637)	–
	Gain on conversion of Ijarah agreements		63,290	–
	Gain on sale of non banking assets - net	14.1.2	3,976	111,948
			192,103	244,551

		Note	2020	2019
			(Rupees in '000)	
**31.**	**OPERATING EXPENSES**			
	**Total compensation expense**	31.1	18,201,007	17,017,561
	**Property expense**			
	Rent and taxes		214,770	395,237
	Insurance		66,237	57,198
	Utilities cost		1,350,228	1,338,457
	Fuel expense generators		334,634	534,428
	Security (including guards)		1,444,492	1,589,659
	Repair and maintenance (including janitorial charges)		758,318	924,232
	Depreciation on right-of-use assets	12.3	1,697,826	1,610,893
	Depreciation	12.2	845,654	659,926
			6,712,159	7,110,030
	**Information technology expenses**			
	Software maintenance		1,304,566	1,279,569
	Hardware maintenance		342,392	372,808
	Depreciation	12.2	688,155	732,943
	Amortisation	13	469,860	425,585
	Network charges		705,798	714,755
	Insurance		6,294	5,198
			3,517,065	3,530,858
	**Other operating expenses**			
	Directors' fees and allowances		50,060	57,479
	Fees and allowances to Sharia Board members		9,417	9,147
	Legal and professional charges		312,026	406,992
	Outsourced services costs	37.1	892,810	891,883
	Travelling and conveyance		322,162	412,033
	NIFT clearing charges		186,480	170,664
	Depreciation	12.2	869,411	840,139
	Depreciation on non-banking assets acquired in satisfaction of claims	14.1.1	30,049	45,456
	Training and development		43,062	73,194
	Postage and courier charges		286,548	355,306
	Communication		353,513	390,721
	Stationery and printing		624,477	710,930
	Marketing, advertisement & publicity		713,479	683,174
	Donations	31.2	112,596	100
	Auditors' remuneration	31.3	41,295	43,305
	Cash transportation charges		709,262	799,226
	Repair and maintenance		404,247	417,823
	Subscription		43,581	39,360
	Entertainment		225,692	275,532
	Remittance charges		211,631	197,223
	Brokerage expenses		69,542	48,342
	Card related expenses		829,055	1,182,346
	CNIC verification charges		128,614	206,509
	Insurance		1,570,511	1,511,988
	Others		294,166	258,110
			9,333,686	10,026,982
			37,763,917	37,685,431

Total cost for the year included in other operating expenses relating to outsourced activities is Rs 275.517 million (2019: Rs 307.993 million) which pertains to payments made to companies incorporated in Pakistan. This includes payments other than outsourced services costs, which are disclosed above. Total Cost of outsourced activities for the year given to related parties is Rs 275.517 million (2019: Rs 258.190 million). Outsourcing shall have the same meaning as specified in BPRD Circular No. 06 of 2019.

Notes To The Consolidated Financial Statements

For the year ended December 31, 2020

The Bank has material outsourcing arrangement with “Euronet Pakistan Private Limited” (a related party) for hosting of debit cards, prepaid cards, POS terminals and ATMs switch system. The annual cost of outsourcing is Rs 275.517 million (2019: 258.190 million).

	Note	2020	2019
		(Rupees in '000)	
**31.1 Total compensation expense**			
Fees and allowances		488,500	520,877
Managerial remuneration			
i) Fixed		12,913,952	12,380,700
ii) Variable - cash bonus / awards		2,640,316	2,229,669
Charge / (reversal) for defined benefit plan		74,827	(101,509)
Contribution to defined contribution plan		455,650	422,671
Commission		350,803	341,105
Staff group insurance		433,891	367,855
Rent and house maintenance		419,324	404,007
Medical		68,143	67,855
Conveyance		351,101	346,631
**Sub-total**		18,196,507	16,979,861
Sign-on bonus	31.1.1	3,700	100
Severance allowance	31.1.2	800	37,600
**Grand Total**		18,201,007	17,017,561

31.1.1 During the year sign on bonus was paid to 4 employees (2019: 1).

31.1.2 Severance allowance pertains to 1 employee (2019: 6).

31.2 Detail of donations made during the year is as follows:

	2020	2019
	(Rupees in '000)	
Saleem Memorial Trust Hospital	95,000	–
Specialized Healthcare and Medical Education Department, Government of Punjab - (COVID 19 relief)	9,996	–
Jahandad Society For Community Development	5,000	–
District Administration Lahore - (COVID 19 relief)	2,600	–
Murshid Hospital & Health Care Centre	–	100
	112,596	100

31.2.1 None of the directors, executives or their spouses had any interest in the donees, except for Mr. Yahya Saleem who is a director in the Saleem Memorial Trust Hospital.

	2020	2019
	(Rupees in '000)	
**31.3 Auditors’ remuneration**		
Audit fee	16,500	15,628
Fee for audit of foreign branches	10,272	9,905
Fee for audit of subsidiaries	11,575	13,361
Special certifications and sundry advisory services	473	348
Sales tax	825	2,500
Out-of-pocket expenses	1,650	1,563
	41,295	43,305

Notes To The Consolidated Financial Statements

For the year ended December 31, 2020

		Note	2020	2019
			(Rupees in '000)	
**32.**	**OTHER CHARGES**			
	Penalties of State Bank of Pakistan		192,356	70,455
	VAT & National Building tax & Crop Insurance Levy		92,502	179,643
	Education cess		13,129	10,645
			297,987	260,743
**33.**	**PROVISIONS / (REVERSALS) & WRITE OFFS - NET**			
	(Reversal) of provision against balance with Banks		(3,547)	(3,111)
	(Reversal) / provision for diminution in value of investments	10.3.1	(69,658)	3,000,801
	Provision / (Reversal) against loans and advances	11.4	7,558,293	(144,827)
	(Reversal) / provision against off balance sheet items	20	(448)	9,025
	Reversal of provision against other assets	14.2.1	(23,648)	(23,436)
	Recovery of written off / charged off bad debts		(130,948)	(164,860)
			7,330,044	2,673,592
**34.**	**TAXATION**			
	Current		20,384,542	15,580,790
	Prior years		(8,023)	450,785
	Deferred	19	(770,451)	55,718
	Share of tax of associates		149,951	119,089
			19,756,019	16,206,382
	**34.1** Relationship between tax expense and accounting profit			
	Accounting profit for the year		49,318,288	40,153,593
	Tax rate		35%	35%
	Tax on income		17,261,401	14,053,758
	Tax effect of permanent differences		67,325	24,659
	Tax effect of prior years reversals		(8,023)	450,785
	Others		2,435,317	1,677,180
	Tax charge for the year		19,756,019	16,206,382

		2020	2019
		(Rupees in '000)	
**35.**	**BASIC AND DILUTED EARNINGS PER SHARE**		
	Profit for the year after tax attributable to equity shareholders	29,410,227	23,868,249
		(Number)	
	Weighted average number of ordinary shares	1,185,060,006	1,185,060,006
		(Rupees)	
	Basic and diluted earnings per share	24.82	20.14

Notes To The Consolidated Financial Statements
For the year ended December 31, 2020

	Note	2020	2019
		(Rupees in '000)	
**36. CASH AND CASH EQUIVALENTS**			
Cash and balances with treasury banks	7	132,053,041	142,957,358
Balances with other banks	8	29,011,521	21,371,753
Overdrawn nostro accounts	17	(368,920)	(1,342,603)
		160,695,642	162,986,508

36.1 Reconciliation of movement of liabilities to cash flows arising from financing activities

	2020							2019						
	Liabilities		Equity					Liabilities		Equity				
	Sub-ordinated loan	Other liabilities	Share capital	Reserves	Unappropriated profit	Non-controlling interest	Total	Sub-ordinated loan	Other liabilities	Share capital	Reserves	Unappropriated profit	Non-controlling interest	Total
	(Rupees in '000)													
**Balance as at January 01,**	–	102,405,513	11,850,600	77,894,829	56,108,779	740,403	249,000,124	3,891,019	65,487,042	11,850,600	74,374,573	53,971,079	708,752	210,283,065
Changes from Financing cash flows														
Redemption of Subordinated loan	–	–	–	–	–	–	–	(3,891,019)	–	–	–	–	–	(3,891,019)
Payment of lease liability against right-of-use-assets	–	(2,327,634)	–	–	–	–	(2,327,634)	–	(1,987,479)	–	–	–	–	(1,987,479)
Dividend paid	–	–	–	–	(11,758,315)	(113,891)	(11,872,206)	–	–	–	–	(18,830,653)	(47,331)	(18,877,984)
Total changes from financing cash flows	–	(2,327,634)	–	–	(11,758,315)	(113,891)	(14,199,840)	(3,891,019)	(1,987,479)	–	–	(18,830,653)	(47,331)	(24,756,482)
**Liability related**														
Changes in Other liabilities														
- Cash based	–	(19,148,240)	–	–	–	–	(19,148,240)	–	13,998,645	–	–	–	–	13,998,645
- Dividend payable	–	92,285	–	–	(92,285)	–	–	–	130,307	–	–	(130,307)	–	–
- Non cash based	–	10,005,234	–	–	–	–	10,005,234	–	24,776,998	–	–	–	–	24,776,998
Total liability related other changes	–	(9,050,721)	–	–	(92,285)	–	(9,143,006)	–	38,905,950	–	–	(130,307)	–	38,775,643
Total equity related other changes	–	–	–	3,165,222	26,240,641	152,049	29,557,912	–	–	–	3,520,256	21,098,660	78,982	24,697,898
Balance as at December 31	–	91,027,158	11,850,600	81,060,051	70,498,820	778,561	255,215,190	–	102,405,513	11,850,600	77,894,829	56,108,779	740,403	249,000,124

	2020	2019
	(Number)	
**37. STAFF STRENGTH**		
Permanent	15,437	15,422
On Bank contract	130	142
Bank's own staff strength at end of the year	15,567	15,564

37.1 In addition to the above, 618 (2019: 728) employees of outsourcing services companies were assigned to the Group as at the end of the year to perform services other than guarding, tea and janitorial services. Outsourced staff includes 611 (2019: 718) working domestically and 7 (2019: 10) working abroad.

38. DEFINED BENEFIT PLAN

38.1 General description

The Group operates the following retirement benefits for its employees:

- Pension fund - funded
- Benevolent scheme - unfunded
- Post retirement medical benefits - unfunded
- Employees compensated absence - unfunded

The plan assets and defined benefit obligations are based in Pakistan.

38.2 Number of Employees under the scheme

The number of employees covered under the following defined benefit schemes are:

	2020	2019
	(Number)	
- Pension fund - funded	5,410	6,294
- Benevolent scheme - unfunded	1,108	1,692
- Post retirement medical benefits - unfunded	13,386	13,332
- Employees compensated absence - unfunded	13,386	13,332

38.3 Principal actuarial assumptions

The latest actuarial valuations of the pension fund, employees' contributory benevolent scheme, post retirement medical benefits and employee's compensated absences were carried out at December 31, 2020. The principal actuarial assumptions used are as follows:

	Approved Pension fund		Employees' contributory benevolent scheme		Post retirement medical benefits		Employees compensated absences	
	2020	2019	2020	2019	2020	2019	2020	2019
	(%)							
Discount rate	9.75	11.25	9.75	11.25	9.75	11.25	9.75	11.25
Expected rate of return on plan assets	9.75	11.25	–	–	–	–	–	–
Expected rate of salary increase	7.75	9.25	7.75	9.25	–	–	7.75	9.25
Expected rate of increase in pension	0-5	0-5	–	–	–	–	–	–
Expected rate of increase in medical benefit	–	–	–	–	7.75	9.25	–	–

Notes To The Consolidated Financial Statements
For the year ended December 31, 2020

38.4 Reconciliation of (receivable from) / payable to defined benefit plans

	Approved Pension fund		Employees' contributory benevolent scheme		Post retirement medical benefits		Employees compensated absences	
	2020	2019	2020	2019	2020	2019	2020	2019
	(Rupees in '000)							
Present value of obligations	5,097,744	5,182,991	222,084	221,193	2,004,122	1,921,348	919,407	939,495
Fair value of plan assets	(8,467,923)	(8,788,112)	–	–	–	–	–	–
(Receivable) / payable	(3,370,179)	(3,605,121)	222,084	221,193	2,004,122	1,921,348	919,407	939,495

38.5 Movement in defined benefit obligations

	Note	Approved Pension fund		Employees' contributory benevolent scheme		Post retirement medical benefits		Employees compensated absences	
		2020	2019	2020	2019	2020	2019	2020	2019
		(Rupees in '000)							
Obligations at the beginning of the year		5,182,991	4,690,587	221,193	214,252	1,921,348	1,730,409	939,495	1,028,129
Current service cost	38.8.1	64,350	60,879	21,742	22,341	53,018	43,653	24,428	24,740
Interest cost		560,303	592,985	22,931	26,035	208,381	219,145	98,224	128,046
Benefits paid		(405,032)	(430,453)	(34,721)	(35,528)	(138,149)	(152,964)	(132,782)	(123,486)
Re-measurement loss / (gain)	38.8.2	(304,868)	268,993	(9,061)	(5,907)	(40,476)	81,105	(9,958)	(117,934)
Obligations at end of the year		5,097,744	5,182,991	222,084	221,193	2,004,122	1,921,348	919,407	939,495

38.6 Movement in fair value of plan assets

	Note	Approved Pension fund		Employees' contributory benevolent scheme		Post retirement medical benefits		Employees compensated absences	
		2020	2019	2020	2019	2020	2019	2020	2019
		(Rupees in '000)							
Fair value at the beginning of the year		8,788,112	8,505,757	–	–	–	–	–	–
Interest income on plan assets		965,880	1,098,495	–	–	–	–	–	–
Benefits paid		(405,032)	(430,453)	–	–	–	–	–	–
Re-measurement loss	38.8.2	(881,037)	(385,687)	–	–	–	–	–	–
Fair value at end of the year		8,467,923	8,788,112	–	–	–	–	–	–

38.7 Movement in (receivable) / payable under defined benefit schemes

	Note	Approved Pension fund		Employees' contributory benevolent scheme		Post retirement medical benefits		Employees compensated absences	
		2020	2019	2020	2019	2020	2019	2020	2019
		(Rupees in '000)							
Opening balance		(3,605,121)	(3,815,170)	221,193	214,252	1,921,348	1,730,409	939,495	1,028,129
Charge / (reversal) for the year	38.8.1	(341,227)	(444,631)	41,961	45,472	261,399	262,798	112,694	34,852
Employees' contribution		–	–	2,712	2,904	–	–	–	–
Re-measurement loss / (gain) recognised in OCI		–	–	–	–	–	–	–	–
during the year	38.8.2	576,169	654,680	(9,061)	(5,907)	(40,476)	81,105	–	–
Benefits paid by the Group		–	–	(34,721)	(35,528)	(138,149)	(152,964)	(132,782)	(123,486)
Closing balance		(3,370,179)	(3,605,121)	222,084	221,193	2,004,122	1,921,348	919,407	939,495

38.8 Charge for defined benefit plans

38.8.1 Cost recognised in profit and loss

	Note	Approved Pension fund		Employees' contributory benevolent scheme		Post retirement medical benefits		Employees compensated absences	
		2020	2019	2020	2019	2020	2019	2020	2019
		(Rupees in '000)							
Current service cost		64,350	60,879	21,742	22,341	53,018	43,653	24,428	24,740
Net interest on defined benefit asset / liability		(405,577)	(505,510)	22,931	26,035	208,381	219,145	98,224	128,046
Employees' contribution		–	–	(2,712)	(2,904)	–	–	–	–
Actuarial (gain)		–	–	–	–	–	–	(9,958)	(117,934)
		(341,227)	(444,631)	41,961	45,472	261,399	262,798	112,694	34,852

38.8.2 Re-measurements recognised in OCI during the year

	Approved Pension fund		Employees' contributory benevolent scheme		Post retirement medical benefits		Employees compensated absences	
	2020	2019	2020	2019	2020	2019	2020	2019
	(Rupees in '000)							
Loss / (gain) on obligation - financial assumptions	(304,868)	268,993	(9,061)	(5,907)	(40,476)	81,105	–	–
Actual return on plan assets over expected interest income	881,037	385,687	–	–	–	–	–	–
Re-measurement loss / (gain) recognised in OCI	576,169	654,680	(9,061)	(5,907)	(40,476)	81,105	–	–

38.9 Components of plan assets

	Approved Pension fund		Employees' contributory benevolent scheme		Post retirement medical benefits		Employees compensated absences	
	2020	2019	2020	2019	2020	2019	2020	2019
	(Rupees in '000)							
Cash and cash equivalents - net	24,451	107,344	–	–	–	–	–	–
Shares	8,129,575	8,387,473	–	–	–	–	–	–
Open ended mutual funds units	313,897	293,295	–	–	–	–	–	–
	8,467,923	8,788,112	–	–	–	–	–	–

38.9.1 Significant risk associated with the plan assets

The Fund's investments in equity securities and units of mutual funds are subject to price risk. These risks are regularly monitored by Trustees of the employee funds.

Notes To The Consolidated Financial Statements

For the year ended December 31, 2020

38.10 Sensitivity analysis

Sensitivity analysis has been performed by varying one assumption keeping all other assumptions constant and calculating the impact on the present value of the defined benefit obligations under the various employee benefit schemes. The increase / (decrease) in the present value of defined benefit obligations as a result of change in each assumption is summarized below:

	Approved Pension fund	Employees' contributory benevolent scheme	Post retirement medical benefits	Employees compensated absences
	(Rupees in '000)			
1% increase in discount rate	(337,220)	(16,983)	(222,612)	(56,543)
1% decrease in discount rate	384,510	19,474	275,965	62,843
1 % increase in expected rate of salary increase	78,500	–	–	63,453
1 % decrease in expected rate of salary increase	(73,370)	–	–	(58,050)
1% increase in expected rate of pension increase	282,140	–	–	–
1% decrease in expected rate of pension increase	(252,260)	–	–	–
1% increase in expected rate of medical benefit increase	–	–	144,805	–
1% decrease in expected rate of medical benefit increase	–	–	(123,190)	–

38.11 Expected contributions to be paid to the funds in the next financial year

No contributions are being made to pension fund due to surplus of fair value of plan's assets over present value of defined obligation. No contribution to the pension fund is expected in the next year.

38.12 Expected charge / (reversal) for the next financial year

Based on actuarial advice, management estimates that the charge / (reversal) in respect of defined benefit plans for the year ending December 31, 2021 would be as follows:

	Approved Pension fund	Employees' contributory benevolent scheme	Post retirement medical benefits	Employees compensated absences
	(Rupees in '000)			
Expected charge / (reversal) for the next financial year	(265,940)	38,232	257,380	114,262

38.13 Maturity profile

The weighted average duration of the obligation (in years)	8.34	8.34	8.34	8.34

38.14 Funding Policy

The Bank endeavours to ensure that liabilities under the various employee benefit schemes are covered by the Fund on any valuation date having regards to the various actuarial assumptions such as projected future salary increase, expected future contributions to the fund, projected increase in liability associated with future service and the projected investment income of the Fund.

Notes To The Consolidated Financial Statements

For the year ended December 31, 2020

38.15 The defined benefit plans may expose the Group to actuarial risks such as longevity risk, investment risk, salary increase risk and withdrawal rate risk as described below;

Investment risk	The risk arises when the actual performance of the investments is lower than expectation and thus creating a shortfall in the funding objectives.
Longevity risk	The risk arises when the actual lifetime of retirees is longer than expectation. This risk is measured at the plan level over the entire retiree population.
Salary increase risk	The most common type of retirement benefit is one where the benefit is linked with final salary. The risk arises when the actual increases are higher than expectation and impacts the liability accordingly.
Withdrawal rate	The risk of actual withdrawals varying with the actuarial assumptions can impose a risk to the benefit obligation. The movement of the liability can go either way.

39. DEFINED CONTRIBUTION PLAN

MCB Bank Limited (holding company)

The Bank operates an approved contributory provident fund for 11,458 (2019: 11,056) employees where contributions are made by the Bank at 8.33% (2019: 8.33%) and employees ranging from 8.33% to 15% per annum (2019: 8.33% to 15% per annum) of the basic salary.

The Bank also operates an approved non-contributory provident fund for 687 (2019: 745) employees who have opted for the new scheme, where contributions are made by the employees ranging from 8.33% to 15% per annum (2019: 8.33% to 15% per annum) of the basic salary.

39.1 MCB Islamic Bank Limited

The Bank operates an approved contributory provident fund for 1,384 (2019: 1,304) employees. Equal monthly contributions are made both by the Bank and its permanent employees to the Fund at the rate of 8.33% of the basic salaries of employees. However, an employee has an option to increase his / her contribution upto 15% but the Bank will still contribute 8.33% of the employee's basic salary.

40. COMPENSATION OF DIRECTORS AND KEY MANAGEMENT PERSONNEL

40.1. Total compensation expense

		2020						
		Directors						
	Note	Chairman	Executive (other than CEO)	Non executive	Members shariah board	President/ CEO	Key management personnel	Other material risk takers/ controllers
		(Rupees in '000)						
Fee and allowances	40.2	7,210	–	42,850	–	–	112	10,941
Managerial Remuneration								
i) Fixed		–	–	–	5,681	72,362	342,162	861,129
ii) Cash Bonus / Awards	40.1.1	–	–	–	900	50,000	170,969	293,927
Contribution to defined contribution plan		–	–	–	177	–	9,608	34,877
Rent & house maintenance		–	–	–	957	240	28,190	84,950
Medical		–	–	–	–	1,828	2,414	11,639
Severance allowance		–	–	–	–	–	800	–
Overseas allowance		–	–	–	–	–	37,559	–
Security		–	–	–	–	804	–	–
Commission		–	–	–	–	–	300	43,960
Utilities		–	–	–	213	–	2,767	16,445
Special Pay		–	–	–	241	–	–	13,123
Conveyance		–	–	–	1,020	–	–	49,713
Club membership		–	–	–	–	1,872	–	300
Fuel Allowance		–	–	–	228	–	–	11,478
Leave fare assistance		–	–	–	–	–	1,213	11,679
Others		–	–	–	–	–	5,989	27,106
Total		7,210	–	42,850	9,417	127,106	602,083	1,471,267
Number of Persons		1	–	11	3	1	27	200

Notes To The Consolidated Financial Statements
For the year ended December 31, 2020

	Note	2019						
		Directors			Members shariah board	President/ CEO	Key management personnel	Other material risk takers/ controllers
		Chairman	Executive (other than CEO)	Non executive				
		(Rupees in '000)						
Fee and allowances	40.2	25,000	–	32,479	–	–	672	5,418
Managerial Remuneration								
i) Fixed		–	–	–	5,319	63,046	337,830	645,432
ii) Cash Bonus / Awards		–	–	–	1,000	40,000	163,711	199,635
Contribution to defined contribution plan		–	–	–	163	2,484	10,204	26,850
Rent & house maintenance		–	–	–	879	240	29,636	90,690
Medical		–	–	–	–	1,071	2,369	10,637
Severance allowance		–	–	–	–	–	18,500	8,800
Overseas allowance		–	–	–	–	–	38,947	7,416
Security		–	–	–	–	1,189	–	–
Commission		–	–	–	–	–	57	14,471
Utilities		–	–	–	195	–	3,175	18,282
Special pay		–	–	–	241	–	162	15,882
Conveyance		–	–	–	1,122	–	–	62,670
Club membership		–	–	–	–	70	–	–
Fuel Allowance		–	–	–	228	–	202	14,311
Leave fare assistance		–	–	–	–	–	4,290	16,170
Others		–	–	–	–	–	5,642	24,374
Total		25,000	–	32,479	9,147	108,100	615,397	1,161,038
Number of Persons		1	–	12	3	1	27	173

40.1.1 During the year 2020, Rs 26.06 million bonus has been deferred.

40.2 Remuneration paid to Directors for participation in Board and Committee meetings

	2020										
	Board meeting	For Board Committee									Total
		Board's Audit Committee	BS & DC	RM & PRC	HR & RC	ITC	PP & CA	CR & MC	Wo & WC	*As Board Chairman	
	(Rupees in '000)										
Mian Mohammad Mansha	3,840	–	300	–	300	–	400	–	–	2,370	7,210
Mr. S. M. Muneer	1,700	–	100	–	–	–	–	100	–	–	1,900
Mr. Mohammad Tariq Rafi	1,700	–	–	–	–	–	–	–	200	–	1,900
Mian Umer Mansha	1,700	500	400	500	–	500	400	–	300	–	4,300
Mrs. Iqraa Hassan Mansha	1,200	–	–	–	200	–	100	–	–	–	1,500
Mr. Muhammad Ali Zeb	1,700	500	–	500	300	–	300	400	300	–	4,000
Mr. Mohd Suhail Amar Suresh	4,460	–	400	500	–	500	–	–	–	–	5,860
Mr. Yahya Saleem	4,466	–	–	–	300	200	–	–	–	–	4,966
Mr. Salman Khalid Butt	4,586	–	400	500	–	500	–	400	–	–	6,386
Mr. Masood Ahmed Puri	4,466	–	400	–	–	–	–	–	–	–	4,866
Mr. Shahzad Hussain	1,700	500	–	–	–	–	–	–	–	–	2,200
Mr. Shariffuddin Bin Khalid	4,472	500	–	–	–	–	–	–	–	–	4,972
	35,990	2,000	2,000	2,000	1,100	1,700	1,200	900	800	2,370	50,060

Notes To The Consolidated Financial Statements

For the year ended December 31, 2020

	2019										
		For Board Committee									
	Board meeting	Board's Audit	BS & DC	RM & PRC	HR & RC	ITC	PP & CA	CR & MC	Wo & WC	*As Board Chairman	Total
	(Rupees in '000)										
Mian Mohammad Mansha	–	–	–	–	–	–	–	–	–	25,000	25,000
Mr. S. M. Muneer	130	–	30	–	–	–	–	30	–	–	190
Mr. Tariq Rafi	430	–	–	–	–	–	–	–	–	–	430
Mian Umer Mansha	300	330	330	300	–	330	330	–	–	–	1,920
Mrs. Iqraa Hassan Mansha	230	–	–	–	–	–	–	–	–	–	230
Mr. Muhammad Ali Zeb	330	330	–	230	330	–	300	330	–	–	1,850
Mr. Mohd Suhail Amar Suresh	4,620	–	230	230	–	230	–	–	–	–	5,310
Mr. Yahya Saleem	4,710	–	–	–	130	200	–	–	–	–	5,040
Mr. Salman Khalid Butt	5,814	–	330	230	–	330	–	300	–	–	7,004
Mr. Masood Ahmed Puri	3,503	–	200	–	–	–	–	–	–	–	3,703
Mr. Shahzad Hussain	300	300	–	–	–	–	–	–	–	–	600
Mr. Shariffuddin Bin Khalid	3,504	300	–	–	–	–	–	–	–	–	3,804
Mr. Nor Hizam Bin Hashim	2,268	130	–	–	–	–	–	–	–	–	2,398
	26,139	1,390	1,120	990	460	1,090	630	660	–	25,000	57,479

*During the year, the Board Chairman was paid proportionate amount of Rs 2.370 million in lieu of fixed annual remuneration (2019: Rs. 25 million) approved by the shareholders of the Group in its 62nd Annual General Meeting held on March 26, 2010. Effective from February 05, 2020 in accordance with BPRD Circular No. 03 of 2019 dated August 19, 2019, the remuneration to the Chairman for attending the Board and committee meetings was paid inline with the remuneration scale approved by the shareholders of the Group in its 72nd Annual General Meeting held on March 19, 2020.

40.3 The Chairman has been provided with free use of the Group maintained car. In addition to the above, the Chief Executive and certain executives are provided with free use of the Group maintained cars and household equipment in accordance with the terms of their employment.

40.4 Remuneration paid to Shariah Board Members

	2020			
	Chairman	Resident member	Non-Resident (member)	Total
	(Rupees in '000)			
Meeting Fees and Allowances	–	–	–	–
Other Heads				
Basic salary	2,263	2,128	1,290	5,681
House rent	–	957	–	957
Utilities	–	213	–	213
Conveyance	–	1,020	–	1,020
Fuel	–	228	–	228
Special pay	–	241	–	241
Bonus	–	900	–	900
PF Employer	–	177	–	177
Total Amount	2,263	5,864	1,290	9,417
Total Number of Persons	1	1	1	3

Notes To The Consolidated Financial Statements
For the year ended December 31, 2020

	2019			
	Chairman	Resident member	Non-Resident (member)	Total
	(Rupees in '000)			
Meeting Fees and Allowances	–	–	–	–
Other Heads				
Basic salary	2,166	1,953	1,200	5,319
House rent	–	879	–	879
Utilities	–	195	–	195
Conveyance	–	1,122	–	1,122
Fuel	–	228	–	228
Special pay	–	241	–	241
Bonus	–	1,000	–	1,000
PF Employer	–	163	–	163
Total Amount	2,166	5,781	1,200	9,147
Number of persons	1	1	1	3

41. FAIR VALUE MEASUREMENTS

The fair value of traded investments is based on quoted market prices, except for tradable securities classified by the Group as 'held to maturity'. Quoted securities classified as held to maturity are carried at amortised cost. Fair value of unquoted equity investments other than investments in associates is determined on the basis of break up value of these investments as per the latest available financial statements.

Fair value of fixed term loans, other assets, other liabilities, fixed term deposits and borrowings cannot be calculated with sufficient reliability due to absence of current and active market for such assets and liabilities and reliable data regarding market rates for similar instruments. The provision for impairment of loans and advances has been calculated in accordance with the Group accounting policy as stated in note 6.5 to these consolidated financial statements.

The maturity and repricing profile and effective rates are stated in note 45.

In the opinion of the management, the fair value of the financial assets and financial liabilities other than those carried at fair value and disclosed in note 41.1 are not significantly different from their carrying values since assets and liabilities are either short-term in nature or re-priced over short term.

41.1 Fair value of financial assets

The Group measures fair values using the following fair value hierarchy that reflects the significance of the inputs used in making the measurements:

Level 1: Fair value measurements using quoted prices (unadjusted) in active markets for identical assets or liabilities.

Level 2: Fair value measurements using inputs other than quoted prices included within Level 1 that are observable for the assets or liability, either directly (i.e. as prices) or indirectly (i.e. derived from prices).

Level 3: Fair value measurements using input for the asset or liability that are not based on observable market data (i.e. unobservable inputs).

Valuation techniques used in determination of fair valuation of financial instruments within level 2

Item	Valuation approach and input used
Federal Government securities	The fair values of Federal Government securities are determined using the PKRV rates.
Term Finance and Bonds	Investments in debt securities (comprising term finance certificates, bonds and any other security issued by a company or a body corporate for the purpose of raising funds in the form of redeemable capital) are valued on the basis of the rates announced by the Mutual Funds Association of Pakistan (MUFAP) in accordance with the methodology prescribed by the Securities and Exchange Commission of Pakistan.
Foreign exchange contracts	The valuation has been determined by interpolating the mid rates announced by the State Bank of Pakistan.
Derivatives	The fair values of derivatives which are not quoted in active markets are determined by using valuation techniques. The valuation techniques take into account the relevant underlying parameters including foreign currency involved, interest rates, yield curves, volatilities, contracts duration etc.
Operating fixed assets (land and building) & NBA	Land and buildings are revalued every three years using professional valuers on the panel of Pakistan Banker's Association. The valuation is based on their assessment of market value of the properties.

The table below analyses the financial and non-financial assets carried at fair values, by valuation methods. For financial assets, the Group essentially carries its investments in debt and equity securities at fair values. Valuation of investments is carried out as per guidelines specified by the SBP. In case of non-financial assets, the Group has adopted revaluation model (as per IAS 16) in respect of land and building.

	2020				
	Carrying value	Level 1	Level 2	Level 3	Total
	(Rupees in '000)				
**On balance sheet financial instruments**					
**Financial assets - measured at fair value**					
Investments					
Federal Government Securities	979,926,866	–	979,926,866	–	979,926,866
Shares	20,510,813	20,510,813	–	–	20,510,813
Non-Government Debt Securities	2,858,620	–	2,858,620	–	2,858,620
Foreign Securities	7,474,190	–	7,474,190	–	7,474,190
**Financial assets - disclosed but not measured at fair value**					
Investments (HTM, unlisted ordinary shares and associates)	25,447,046	–	–	–	–
Cash and balances with treasury banks	132,053,041	–	–	–	–
Balances with other banks	29,011,521	–	–	–	–
Lendings to financial institutions	17,968,243	–	–	–	–
Advances	547,685,708	–	–	–	–
Other assets	52,518,097	–	–	–	–
**Non - Financial Assets measured at fair value**					
Operating fixed assets (land and buildings)	45,595,081	–	45,595,081	–	45,595,081
Non-banking assets	4,036,914	–	4,036,914	–	4,036,914
**Off-balance sheet financial instruments - measured at fair value**					
Forward purchase of foreign exchange	172,137,589	–	4,011,602	–	4,011,602
Forward sale of foreign exchange	155,508,653	–	4,240,748	–	4,240,748
Derivatives purchase	2,158,111	–	517,033	–	517,033
Derivatives sale	2,313,272	–	513,343	–	513,343

Notes To The Consolidated Financial Statements

For the year ended December 31, 2020

	2019				
	Carrying value	Level 1	Level 2	Level 3	Total
	(Rupees in '000)				
**On balance sheet financial instruments**					
**Financial assets - measured at fair value**					
Investments					
Federal Government Securities	691,477,045	–	691,477,045	–	691,477,045
Shares	17,075,189	17,075,189	–	–	17,075,189
Non-Government Debt Securities	2,424,102	–	2,424,102	–	2,424,102
Foreign Securities	3,663,065	–	3,663,065	–	3,663,065
**Financial assets - disclosed but not measured at fair value**					
Investments (HTM, unlisted ordinary shares and associates)	42,802,189	–	–	–	–
Cash and balances with treasury banks	142,957,358	–	–	–	–
Balances with other banks	21,371,753	–	–	–	–
Lendings to financial institutions	6,060,869	–	–	–	–
Advances	548,472,860	–	–	–	–
Other assets	58,219,618	–	–	–	–
**Non - Financial Assets measured at fair value**					
Operating fixed assets (land and buildings)	44,882,204	–	44,882,204	–	44,882,204
Non-banking assets	3,838,230	–	3,838,230	–	3,838,230
**Off-balance sheet financial instruments - measured at fair value**					
Forward purchase of foreign exchange	216,647,656	–	4,142,227	–	4,142,227
Forward sale of foreign exchange	188,243,433	–	3,352,404	–	3,352,404
Derivatives purchase	5,176,612	–	1,232,827	–	1,232,827
Derivatives sale	5,068,194	–	1,229,116	–	1,229,116

The Group policy is to recognise transfers into and out of the different fair value hierarchy levels at the date the event or change in circumstances that caused the transfer occurred. There were no transfers between levels 1 and 2 during the year.

(a) Financial instruments in level 1

Financial instruments included in level 1 comprise of investments in listed ordinary shares and units of mutual funds.

(b) Financial instruments in level 2

Financial instruments included in level 2 comprise of Sukuk Bonds, Pakistan Investment Bonds, Market Treasury Bills, Term Finance certificates, FX options, Cross Currency Swaps, Interest Rate Swaps and Forward Exchange Contracts.

(c) Financial instruments in level 3

Currently, no financial instruments are classified in level 3.

Notes To The Consolidated Financial Statements

For the year ended December 31, 2020

42 SEGMENT INFORMATION

42.1 Segment details with respect to business activities

The segment analysis with respect to business activity is as follows:

2020

	Ratail Banking	Consumer Banking	Corporate Banking	Treasury	International Banking	Islamic Banking	Assets Management	Others	Sub-total	Elimination	Total
	(Rupees in '000)										
**Profit & Loss**											
Net mark-up/return/profit	(44,807,214)	2,788,656	28,085,051	83,819,922	1,448,075	4,395,446	(6,607)	120,110	75,843,439	–	75,843,439
Inter segment revenue - net	86,028,798	(486,242)	(22,360,468)	(69,939,999)	(171,994)	–	–	6,929,905	–	–	–
Non mark-up / return / interest income	6,306,651	2,118,001	3,282,442	5,618,895	813,588	678,153	833,565	190,310	19,841,605	–	19,841,605
Total Income	47,528,235	4,420,415	9,007,025	19,498,818	2,089,669	5,073,599	826,958	7,240,325	95,685,044	–	95,685,044
Segment direct expenses	20,460,118	1,462,911	586,415	368,025	1,309,898	4,665,250	407,873	9,776,222	39,036,712	–	39,036,712
Inter segment expense allocation	–	–	–	–	–	–	–	–	–	–	–
Total expenses	20,460,118	1,462,911	586,415	368,025	1,309,898	4,665,250	407,873	9,776,222	39,036,712	–	39,036,712
Provisions	2,306,248	113,643	2,763,155	(31,111)	129,368	15,270	–	2,033,471	7,330,044	–	7,330,044
Profit before tax	24,761,869	2,843,861	5,657,455	19,161,904	650,403	393,079	419,085	(4,569,368)	49,318,288	–	49,318,288
**Balance Sheet**											
Cash and Bank balances	58,362,119	317,242	394,030	64,577,425	21,166,578	14,868,799	25,853	1,352,516	161,064,562	–	161,064,562
Investments	–	–	10,578,310	978,209,256	14,571,071	27,617,997	1,269,444	3,971,457	1,036,217,535	–	1,036,217,535
Net inter segment lending	1,050,376,236	–	–	–	–	–	–	202,001,071	1,252,377,307	(1,252,377,307)	–
Lendings to financial institutions	–	–	–	6,137,258	11,002,195	828,790	–	–	17,968,243	–	17,968,243
Advances - performing	94,735,206	26,845,772	313,362,719	–	21,361,572	84,165,115	5,260	432,897	540,908,541	–	540,908,541
- non performing - net	171,804	177,613	2,382	–	4,973,954	731,076	–	720,338	6,777,167	–	6,777,167
Others	35,621,546	2,370,727	23,382,913	12,206,991	4,577,833	12,079,087	986,042	37,115,208	128,340,347	–	128,340,347
**Total Assets**	1,239,266,911	29,711,354	347,720,354	1,061,130,930	77,653,203	140,290,864	2,286,599	245,593,487	3,143,653,702	(1,252,377,307)	1,891,276,395
Borrowings	58,910,004	–	10,694,773	90,718,570	3,330,124	20,596,773	–	327,096	184,577,340	–	184,577,340
Deposits and other accounts	1,147,251,222	21,263,015	65,961,390	–	55,009,174	99,253,160	–	–	1,388,737,961	–	1,388,737,961
Net inter segment borrowing	–	4,816,853	252,358,835	969,573,590	13,301,145	10,727,772	1,599,112	–	1,252,377,307	(1,252,377,307)	–
Others	33,105,685	3,631,486	18,705,356	838,770	6,012,760	9,713,159	687,487	52,275,008	124,969,711	–	124,969,711
**Total liabilities**	1,239,266,911	29,711,354	347,720,354	1,061,130,930	77,653,203	140,290,864	2,286,599	52,602,104	2,950,662,319	(1,252,377,307)	1,698,285,012
Equity	–	–	–	–	–	–	–	192,991,383	192,991,383	–	192,991,383
**Total Equity & liabilities**	1,239,266,911	29,711,354	347,720,354	1,061,130,930	77,653,203	140,290,864	2,286,599	245,593,487	3,143,653,702	(1,252,377,307)	1,891,276,395
**Contingencies & Commitments**	55,974,597	–	288,001,956	320,068,131	20,930,195	29,021,132	–	26,379,292	740,375,303	–	740,375,303

2019

	Ratail Banking	Consumer Banking	Corporate Banking	Treasury	International Banking	Islamic Banking	Assets Management	Others	Sub-total	Elimination	Total
	(Rupees in '000)										
**Profit & Loss**											
Net mark-up/return/profit	(50,496,018)	3,938,893	36,263,272	68,147,338	1,762,729	3,993,758	(2,059)	110,178	63,718,091	–	63,718,091
Inter segment revenue - net	99,832,569	(2,583,003)	(31,413,118)	(73,135,260)	(180,860)	-	–	7,479,672	–	–	–
Non mark-up / return / interest income	6,318,956	2,111,319	3,414,776	3,519,577	782,895	343,319	722,042	644,430	17,857,314	–	17,857,314
Total Income	55,655,507	3,467,209	8,264,930	(1,468,345)	2,364,764	4,337,077	719,983	8,234,280	81,575,405	–	81,575,405
Segment direct expenses	21,762,153	1,330,299	525,336	311,976	1,284,816	4,503,022	499,336	8,531,282	38,748,220	–	38,748,220
Inter segment expense allocation	–	–	–	–	–	–	–	–	–	–	–
Total expenses	21,762,153	1,330,299	525,336	311,976	1,284,816	4,503,022	499,336	8,531,282	38,748,220	–	38,748,220
Provisions	345,610	(34,217)	947,291	2,772,041	75,176	199,814	–	(1,632,123)	2,673,592	–	2,673,592
Profit before tax	33,547,744	2,171,127	6,792,303	(4,552,362)	1,004,772	(365,759)	220,647	1,335,121	40,153,593	–	40,153,593
**Balance Sheet**											
Cash and Bank balances	50,154,942	21,483	676,139	65,572,277	28,149,362	19,075,532	28,347	651,029	164,329,111	–	164,329,111
Investments	–	–	10,638,292	710,593,710	15,011,692	16,309,796	1,055,226	3,832,874	757,441,590	–	757,441,590
Net inter segment lending	940,015,023	–	–	–	–	–	–	164,180,279	1,104,195,302	(1,104,195,302)	–
Lendings to financial institutions	–	–	–	–	209,206	5,851,663	–	–	6,060,869	–	6,060,869
Advances - performing	98,464,054	24,342,756	341,191,287	–	25,190,939	50,931,880	5,169	478,850	540,604,935	–	540,604,935
- non performing - net	1,074,787	113,148	1,237,494	–	4,836,865	378,087	–	227,544	7,867,925	–	7,867,925
Others	34,759,967	1,882,259	25,393,004	13,804,785	2,368,787	11,538,184	1,060,496	45,102,959	135,910,441	–	135,910,441
**Total Assets**	1,124,468,773	26,359,646	379,136,216	789,970,772	75,766,851	104,085,142	2,149,238	214,473,535	2,716,410,173	(1,104,195,302)	1,612,214,871
Borrowings	45,355,966	–	7,057,504	32,270,471	3,520,222	4,127,525	–	528,280	92,859,968	–	92,859,968
Deposits and other accounts	1,033,790,838	–	55,425,048	–	55,523,628	81,853,511	–	–	1,226,593,025	–	1,226,593,025
Net inter segment borrowing	–	24,124,908	299,650,838	755,967,249	12,464,722	10,466,801	1,520,784	–	1,104,195,302	(1,104,195,302)	–
Others	45,321,969	2,234,738	17,002,826	1,733,052	4,258,279	7,637,305	628,454	42,598,438	121,415,061	–	121,415,061
**Total liabilities**	1,124,468,773	26,359,646	379,136,216	789,970,772	75,766,851	104,085,142	2,149,238	43,126,718	2,545,063,356	(1,104,195,302)	1,440,868,054
Equity	–	–	–	–	–	–	–	171,346,817	171,346,817	–	171,346,817
**Total Equity & liabilities**	1,124,468,773	26,359,646	379,136,216	789,970,772	75,766,851	104,085,142	2,149,238	214,473,535	2,716,410,173	(1,104,195,302)	1,612,214,871
**Contingencies & Commitments**	48,973,361	–	262,455,692	496,556,621	14,015,586	18,397,458	–	25,057,324	865,456,042	–	865,456,042

Notes To The Consolidated Financial Statements

For the year ended December 31, 2020

42.2 Segment details with respect to geographical locations

GEOGRAPHICAL SEGMENT ANALYSIS

	2020						
	Pakistan	South Asia	Middle East	Eurasia	Sub-total	Eliminations	Total
	(Rupees in '000)						
**Profit & Loss**							
Net mark-up/return/profit	74,278,382	862,868	584,128	118,061	75,843,439	–	75,843,439
Inter segment revenue - net	111,160	(95,930)	(15,230)	–	–	–	–
Non mark-up / return / interest income	19,024,163	265,900	543,982	7,560	19,841,605	–	19,841,605
Total Income	93,413,705	1,032,838	1,112,880	125,621	95,685,044	–	95,685,044
Segment direct expenses	37,664,604	650,469	656,321	65,318	39,036,712	–	39,036,712
Inter segment expense allocation	–	–	–	–	–	–	–
Total expenses	37,664,604	650,469	656,321	65,318	39,036,712	–	39,036,712
Provisions	7,199,068	78,422	50,946	1,608	7,330,044	–	7,330,044
Profit before tax	48,550,033	303,947	405,613	58,695	49,318,288	–	49,318,288
**Balance Sheet**							
Cash and Bank balances	140,214,571	1,556,335	19,289,016	4,640	161,064,562	–	161,064,562
Investments	1,021,646,465	10,519,130	4,051,940	–	1,036,217,535	–	1,036,217,535
Net inter segment lendings	12,319,043	–	–	–	12,319,043	(12,319,043)	–
Lendings to financial institutions	6,966,047	1,864,193	9,138,003	–	17,968,243	–	17,968,243
Advances - performing	518,887,766	12,402,421	8,863,250	755,104	540,908,541	–	540,908,541
- non performing - net	6,765,263	11,904	–	–	6,777,167	–	6,777,167
Others	123,724,991	4,054,777	496,834	63,745	128,340,347	–	128,340,347
**Total Assets**	1,830,524,146	30,408,760	41,839,043	823,489	1,903,595,438	(12,319,043)	1,891,276,395
Borrowings	180,920,122	1,470,384	1,859,738	327,096	184,577,340	–	184,577,340
Deposits and other accounts	1,336,595,320	18,298,522	33,844,119	–	1,388,737,961	–	1,388,737,961
Net inter segment borrowing	–	6,525,907	5,793,136	–	12,319,043	(12,319,043)	–
Others	120,442,311	4,113,947	342,050	71,403	124,969,711	–	124,969,711
**Total liabilities**	1,637,957,753	30,408,760	41,839,043	398,499	1,710,604,055	(12,319,043)	1,698,285,012
Equity	192,566,393	–	–	424,990	192,991,383	–	192,991,383
**Total Equity & liabilities**	1,830,524,146	30,408,760	41,839,043	823,489	1,903,595,438	(12,319,043)	1,891,276,395
**Contingencies & Commitments**	719,445,109	17,479,001	3,451,193	–	740,375,303	–	740,375,303

	2019						
	Pakistan	South Asia	Middle East	Eurasia	Sub-total	Eliminations	Total
	(Rupees in '000)						
**Profit & Loss**							
Net mark-up/return/profit	61,849,240	1,085,296	675,651	107,904	63,718,091	–	63,718,091
Inter segment revenue - net	107,447	(119,613)	12,166	–	–	–	–
Non mark-up / return / interest income	17,088,129	251,485	512,276	5,424	17,857,314	–	17,857,314
Total Income	79,044,816	1,217,168	1,200,093	113,328	81,575,405	–	81,575,405
Segment direct expenses	37,396,274	737,142	552,539	62,265	38,748,220	–	38,748,220
Inter segment expense allocation	–	–	–	–	–	–	–
Total expenses	37,396,274	737,142	552,539	62,265	38,748,220	–	38,748,220
Provisions	2,588,709	29,232	65,407	(9,756)	2,673,592	–	2,673,592
Profit before tax	39,059,833	450,794	582,147	60,819	40,153,593	–	40,153,593
**Balance Sheet**							
Cash and Bank balances	136,384,595	2,169,276	25,769,312	5,928	164,329,111	–	164,329,111
Investments	742,429,899	5,571,042	9,440,649	–	757,441,590	–	757,441,590
Net inter segment lendings	11,906,306	–	–	–	11,906,306	(11,906,306)	–
Lendings to financial institutions	5,851,663	209,206	–	–	6,060,869	–	6,060,869
Advances - performing	514,539,167	17,771,048	7,401,837	892,883	540,604,935	–	540,604,935
- non performing - net	7,838,277	29,648	–	–	7,867,925	–	7,867,925
Others	133,505,520	2,193,220	173,639	38,062	135,910,441	–	135,910,441
**Total Assets**	1,552,455,427	27,943,440	42,785,437	936,873	1,624,121,177	(11,906,306)	1,612,214,871
Borrowings	88,811,466	845,703	2,674,519	528,280	92,859,968	–	92,859,968
Deposits and other accounts	1,173,585,208	18,431,297	34,576,520	–	1,226,593,025	–	1,226,593,025
Net inter segment borrowing	–	6,151,041	5,388,302	366,963	11,906,306	(11,906,306)	–
Others	118,711,936	2,515,399	146,096	41,630	121,415,061	–	121,415,061
**Total liabilities**	1,381,108,610	27,943,440	42,785,437	936,873	1,452,774,360	(11,906,306)	1,440,868,054
Equity	171,346,817	–	–	–	171,346,817	–	171,346,817
**Total Equity & liabilities**	1,552,455,427	27,943,440	42,785,437	936,873	1,624,121,177	(11,906,306)	1,612,214,871
Contingencies & Commitments	851,440,456	9,436,548	4,579,038	–	865,456,042	–	865,456,042

42.3 Transactions between reportable segments are based on an appropriate transfer pricing mechanism using agreed rates. Furthermore, segment assets and liabilities include inter segment balances. Costs which are not allocated to segments are included in the Head office. Income taxes are managed at bank level and are not allocated to operating segments.

42.4 No revenue from transactions with a single external customer or counterparty amounted to 10% or more of the Bank's total revenue in 2020 or 2019.

43 RELATED PARTY TRANSACTIONS

The Group has related party relationship with associates, employee benefit plans, its directors and key management personnel and their close family members. The detail of associates are stated in note 10.11 to the consolidated financial statements.

The Group enters into transactions with related parties in the ordinary course of business and on substantially the same terms as for comparable transactions with person of similar standing. Contributions to and accruals in respect of staff retirement benefits and other benefit plans are made in accordance with the actuarial valuations / terms of the contribution plan. Remuneration to the executives / officers is determined in accordance with the terms of their appointment. Remuneration to Chief Executive, Directors and Executives is disclosed in note 40 to the consolidated financial statements.

Details of transactions with related parties during the year, other than those which have been disclosed elsewhere in these consolidated financial statements are as follows:

	2020				2019			
	Directors	Key management personnel and shariah advisors	Associates	Other related Parties	Directors	Key management personnel and shariah advisors	Associates	Other related parties
	(Rupees in '000)							
**Balances with other banks**								
In current accounts	–	–	–	–	–	–	–	14,389
	–	–	–	–	–	–	–	14,389
**Investments**								
Opening balance	–	–	4,275,658	1,283,438	–	–	4,019,526	1,283,998
Equity method adjustments	–	–	203,702	–	–	–	256,132	–
Investment made during the year	–	–	–	10,609,883	–	–	–	8,906,732
Investment disposed off during the year	–	–	–	(10,516,123)	–	–	–	(8,907,292)
Closing balance	–	–	4,479,360	1,377,198	–	–	4,275,658	1,283,438
**Provision for diminution in value of investments**	–	–	–	5,000	–	–	–	5,000
**Advances**								
Opening balance	1,722	223,338	–	1,101,457	658	253,945	–	1,540,791
Addition / exchange adjustment during the year	18,202	95,758	–	3,282,404	29,148	77,133	–	4,275,492
Repaid during the year	(18,882)	(40,170)	–	(3,365,263)	(28,084)	(84,375)	–	(3,920,116)
Transfer in / (out)	–	(3,808)	–	244,397	–	(23,365)	–	(794,710)
Closing balance	1,042	275,118	–	1,262,995	1,722	223,338	–	1,101,457

Notes To The Consolidated Financial Statements
For the year ended December 31, 2020

	2020				2019			
	Directors	Key management personnel and shariah advisors	Associates	Other related Parties	Directors	Key management personnel and shariah advisors	Associates	Other related parties
	(Rupees in '000)							
**Other Assets**								
Markup receivable	-	3,149	895	25,883	-	4,948	-	21,627
Advances, deposits, advance rent and other prepayments	-	2,393	310,504	208,094	-	587	246,720	612,811
Receivable from Pension Fund	-	-	-	3,370,179	-	-	-	3,605,121
**Borrowings**								
Opening balance	-	-	-	69,166	-	-	-	-
Borrowings / exchange adjustment during the year	-	-	-	7,973	-	-	-	144,166
Settled during the year	-	-	-	-	-	-	-	(75,000)
Closing balance	-	-	-	77,139	-	-	-	69,166
**Deposits and other accounts**								
Opening balance	602,381	194,366	4,013,859	5,124,551	3,745,457	202,014	3,696,154	3,245,407
Received during the year	669,282	3,851,770	44,628,206	99,003,416	9,889,584	1,152,025	22,234,251	78,846,758
Withdrawn during the year	(969,533)	(3,257,022)	(43,469,978)	(97,417,931)	(13,025,336)	(1,139,761)	(21,916,546)	(77,299,321)
Transfer in / (out) - net	-	(45,994)	-	(648,365)	(7,324)	(19,912)	-	331,707
Closing balance	302,130	743,120	5,172,087	6,061,671	602,381	194,366	4,013,859	5,124,551
**Other Liabilities**								
Markup payable	50	3,159	42,549	16,004	7,263	818	50,535	75,118
Accrued expenses and other payable	-	-	62,624	18,002	15,326	2,640	62,402	7,629
Payable to MCB Employee Security Services	-	-	-	27,031	-	-	-	24,565
**Contingencies and Commitments**								
Commitments and contingent liabilities - outstanding	-	-	10,512	2,859,661	-	-	10,444	2,445,772

Notes To The Consolidated Financial Statements
For the year ended December 31, 2020

	2020				2019			
	Directors	Key management personnel and shariah advisors	Associates	Other related parties	Directors	Key management personnel and shariah advisors	Associates	Other related parties
	(Rupees in '000)							
**Income**								
Markup / return / interest earned	–	18,298	1,809	127,475	–	16,843	–	87,124
Fee and commission income	–	–	1,177,371	51,520	–	27	1,301,954	218,005
Dividend income	–	–	192,500	61,949	–	–	175,000	70,408
Gain on forward foreign exchange contracts matured during the year	–	–	–	53,120	–	–	–	12,850
Net gain on sale of securities	72	40	3,836	6,485	–	–	281	10,990
Gain on sale of assets	–	98	–	516	–	186	–	–
Rent income and reimbursement of other expenses	–	18	8,808	56,012	–	–	8,485	44,812
Management fee and Advisory income	–	–	–	698,356	–	–	–	677,735
**Expense**								
Markup / return / interest expensed	30,257	43,279	223,767	307,205	145,454	38,562	218,029	395,520
**Other Operating expenses**								
Clearing expenses paid to NIFT	–	–	–	166,175	–	–	–	152,009
Contribution to provident fund	–	–	–	518,600	–	–	–	479,670
Rent expenses	–	–	42,264	53,224	–	–	21,347	45,055
Cash sorting expenses	–	–	–	114,845	–	–	–	160,766
Stationery expenses	–	–	–	244,697	–	–	–	258,411
Security guards expenses	–	–	–	381,267	–	–	–	414,106
Remuneration to key executives, shariah advisors and non-executive directors fee	177,166	708,229	–	–	165,579	523,315	–	–
Outsourcing service expenses	–	–	275,517	–	–	–	258,190	–
Donation Expense	–	–	–	95,000	–	–	–	–
E-dividend processing fee and CDC charges	–	–	–	4,757	–	–	–	5,968
Travelling expenses	–	–	–	38,507	–	–	–	40,218
Hotel stay expenses	–	–	–	3,410	–	–	–	–
Repair and maintenance charges	–	–	–	1,989	–	–	–	2,125
Advertisement expenses	–	–	–	7,308	–	–	–	12,934
Miscellaneous expenses and payments	–	–	–	3,243	–	850	–	3,023
Selling and Marketing	–	–	–	287,916	–	–	–	153,648
Sharia Fee Paid	–	–	–	2,458	–	–	–	9,147
Insurance premium-net of refund	–	–	646,676	116,502	–	–	719,873	106,825
Insurance claim settled	–	–	46,067	6,420	–	–	57,566	291
**Other Transactions**								
Proceeds from sale of assets	–	399	–	–	–	466	–	–
Purchase of fixed assets	–	–	3,277	6,150	–	–	46,339	20,003
Sale of government securities	268,847	124,304	5,740,348	8,592,672	203,735	94,105	6,043,338	22,634,697
Purchase of government securities	19,827	999	1,232,917	653,148	–	58,956	6,410,544	17,302,184
Forward exchange contracts matured during the year	–	–	–	11,446,226	–	–	–	24,426,964
Insurance claim paid to related party	–	–	–	2,100,927	–	–	–	2,198,407
Letter of Guarantee and credit issued								

The Chairman has been provided with free use of the Group maintained car. The Chief Executive and certain executives are provided with free use of the Group maintained cars and household equipment in accordance with the terms of their employment.

Notes To The Consolidated Financial Statements
For the year ended December 31, 2020

	2020	2019
	(Rupees in '000)	
**44 CAPITAL ADEQUACY, LEVERAGE RATIO & LIQUIDITY REQUIREMENTS**		
**44.1 Capital Adequacy**		
**Minimum Capital Requirement (MCR):**		
Paid-up capital (net of losses)	11,850,600	11,850,600
**Capital Adequacy Ratio (CAR):**		
Eligible Common Equity Tier 1 (CET 1) Capital	149,417,496	132,504,188
Eligible Additional Tier 1 (ADT 1) Capital	–	–
Total Eligible Tier 1 Capital	149,417,496	132,504,188
Eligible Tier 2 Capital	36,710,001	28,503,091
**Total Eligible Capital (Tier 1 + Tier 2)**	186,127,497	161,007,279
**Risk Weighted Assets (RWAs):**		
Credit Risk	668,413,516	662,366,497
Market Risk	128,392,302	113,394,030
Operational Risk	148,348,258	126,966,330
Total	945,154,076	902,726,857
**Common Equity Tier 1 Capital Adequacy ratio**	15.81%	14.68%
**Tier 1 Capital Adequacy Ratio**	15.81%	14.68%
**Total Capital Adequacy Ratio**	19.69%	17.84%

The SBP through its BSD Circular No. 07 dated April 15, 2009 has prescribed the minimum paid up capital (net of losses) for all locally incorporated banks of Rs. 10 billion. The paid up capital of the Holding company for the year ended December 31, 2020 stood at Rs. 11.851 billion and is in compliance with the SBP requirements. Further, under Basel III instructions, banks are required to maintain minimum Capital Adequacy Ratio (CAR) of 11.50% (including a capital conservation buffer of 1.5% which has been revised downwards from 2.5% as per BPRD Circular No. 12 dated March 26, 2020), Common Equity Tier 1 (CET 1) ratio of 6.0% and Tier 1 ratio of 7.50% as at December 31, 2020. The Group is fully compliant with prescribed ratios.

Under the current capital adequacy regulations, credit risk and market risk exposures are measured using the Standardized Approach and operational risk is measured using the Basic Indicator Approach. Credit risk mitigants are also applied against the Group's exposures based on eligible collateral under simple approach.

	2020	2019
	(Rupees in '000)	
**44.2 Leverage Ratio (LR):**		
Eligible Tier-1 Capital	149,417,496	132,504,188
Total Exposures	2,323,456,613	2,037,241,480
Leverage Ratio	6.43%	6.50%

Notes To The Consolidated Financial Statements

For the year ended December 31, 2020

	2020	2019
	(Rupees in '000)	
**44.3 Liquidity Requirements**		
**Liquidity Coverage Ratio (LCR):**		
Total High Quality Liquid Assets	962,045,524	734,587,045
Total Net Cash Outflow	415,665,992	379,626,249
Liquidity Coverage Ratio	231.45%	193.50%
**Net Stable Funding Ratio (NSFR):**		
Total Available Stable Funding	1,212,910,470	1,045,877,006
Total Required Stable Funding	715,405,667	745,689,676
Net Stable Funding Ratio	169.54%	140.26%

The full disclosures on the Capital Adequacy, Leverage Ratio & Liquidity Requirements as per SBP instructions issued from time to time are available at https://www.mcb.com.pk/investor-relations/capital-adequacy-statements.

45 RISK MANAGEMENT

Risk is an inherent part of banking business activities. The risk management framework and governance structure of Groups helps to mitigate and counter any foreseeable risk in its various lines of business. Risk awareness forms an integral part of strategic and operational activities of risk management. Through its Global Risk Management Policy, Group sets the best course of action under uncertainty by identifying, prioritizing, mitigating and monitoring risk issues, with the goal of enhancing shareholders' value. Group's risk management structure is based on the following five guiding principles:

- Optimizing risk/return in a controlled manner
- Establishing clear responsibility and accountability
- Establishing independent and properly resourced risk management function.
- Promoting open risk culture
- Adopting international best practices in risk management

Keeping in view dynamics of internal and external environment, the group regularly reviews and updates policy manuals / frameworks and procedures in accordance with domestic regulatory environment and international standards.

The Group executes its risk strategy and undertakes controlled risk-taking activities within its risk management framework. The Board of Directors and its relevant committee, i.e. the Risk Management & Portfolio Review Committee (RM&PRC), the senior management and its relevant committees, i.e. the Management Credit and Risk Committee (MC&RC), Asset Liability Committee (ALCO), etc., are responsible to ensure formulation and implementation of comprehensive Risk Management Framework. This framework is based on prudent risk identification, measurement, management and monitoring processes which are closely aligned with the activities of the Group. The framework combines core policies, procedures and process designs with broad oversight and is supported by an efficient monitoring mechanism across the bank to ensure that risks are kept within an acceptable level.

The Group ensures that not only the relevant risks are identified but their implications are also considered and basis provided for managing and measuring the risks. Through Internal Control units, the Group ensures that effective controls are in place to mitigate each of the identified risk.

Independent from business groups, Head of Risk Management reports functionally to the Risk Management & Portfolio Review Committee (RM&PRC) and administratively to the President; the RM&PRC convenes regular meetings to evaluate bank's risk and portfolio concentrations. The Risk Management Group performs the following critical functions:

- Risk Management Policy Formulation
- Credit Risk Management
- Credit Review

Notes To The Consolidated Financial Statements
For the year ended December 31, 2020

- Credit Risk Control
- Market Risk Management
- Liquidity Risk Management
- Operational Risk Management
- IT Risk Management

Keeping in view the international best practices and SBP requirements, Board of Directors of the Group has approved a Risk Appetite Statement, which takes into account quantitative and qualitative risk indicators, covering target ratios, credit, market, operational, liquidity and business risks.

45.1 Credit Risk

Credit risk arises from Groups's dealings with individuals, corporate borrowers, financial institutions, sovereigns etc. The Group is exposed to credit risk through its lending and investment activities. Credit risk makes up the largest part of the Group's exposure and it stems from Group's both on and off-balance sheet activities. Purpose of Credit Risk Management function is to identify, measure, manage, monitor and mitigate credit risk. To manage adverse outcomes in terms of unfavorable scenarios, multiple control factors in the lending structure of the Group provide additional comfort and support. Such controls range from quality of eligible collateral, pre-disbursement safety measures to post disbursement monitoring.

The Group has adopted Standardized Approach to measure Credit risk regulatory capital charge in compliance with Basel requirements. The approach mainly takes into account the assessment of external credit rating agencies. In line with SBP guidelines on Internal Credit Risk Rating Systems, the Group has developed rating systems and all its borrowers are internally rated. In order to further enhance the credit risk analysis and the processes, Probability Default based internal credit risk rating (ICRR) system based on the statistical modeling and validation in line with Basel principles. The revamped ICRR is currently focused on Corporate Commercial and Corporate Large customer categories. The Internal Credit Risk Rating Model for rating of SME Customers has also been revamped to achieve more accurate results and to improve the quality of credit decisions.

In order to manage groups's credit risk, following policies and procedures are in place:

- Individuals who take or manage risks clearly understand them in order to protect the Bank from avoidable risks;
- The approval of credit limits to counter parties are subject to pre-fact review;
- Extension in credit facility or material change to the credit facility is subject to credit review;
- Approval and review process is reviewed by RM&PRC and internal audit;
- Management periodically reviews the powers of credit approving and credit reviewing authorities.

Ongoing administration of the credit portfolio is an essential part of the credit process that supports and controls extension and maintenance of credit. The Group's Credit Risk Control is responsible for performing following activities:

- Credit disbursement authorization
- Collateral coverage and monitoring
- Compliance of loan covenants/ terms of approval
- Maintenance/ custody of collateral and security documentation
- Credit Risk Limit Controls

Credit Risk Monitoring is based on a comprehensive reporting framework. Continuous monitoring of the credit portfolio and the risks attached thereto are carried out at different levels including businesses, Audit & Risk Assets Review, Credit Risk Control, Credit Risk Management Division, etc.

To ensure a prudent distribution of asset portfolio, the Group manages its lending and investment activities within an appropriate limits framework. Per party exposure limit is maintained in accordance with SBP Prudential Regulations.

The Group creates specific provision against Non-Performing Loans (NPLs) in accordance with the Prudential Regulations and other directives issued by the State Bank of Pakistan (SBP) and charged to the profit and loss account. Provisions are held against identified as well as unidentified losses. Provisions against unidentified losses include general provision against consumer loans and Small enterprise (SEs) made in accordance with the requirements of the Prudential Regulations issued by

SBP and provision based on historical loss experience on advances. General provisions pertaining to overseas advances are made in accordance with the requirements of the regulatory authorities of the respective countries. Please refer note No. 11.4 for reconciliation of changes in specific and general provisions.

The Risk Management function of the Group has further strengthened its credit review procedures in the light of COVID-19 and is regularly conducting assessments of the credit portfolio to identify borrowers most likely to be affected due to changes in the business and economic environment.

Management of Non Performing Loans

The Group has a Special Assets Management (SAM) function, which is responsible for management of non performing loans. SAM undertakes restructuring / rescheduling of problem loans, as well as litigation of both civil and criminal cases for collection of debt.

Stress Testing

Credit Risk stress testing is a regular exercise. Group's credit exposures including funded and non-funded facilities are subjected to stress tests. This exercise is conducted on a quarterly basis through assigning shocks to all assets of the Bank and assessing its resulting affect on capital adequacy inline with SBP requirements.

45.1.1 Lendings to financial institutions

Credit risk by public / private sector

	Gross lendings		Non - performing lending		Provision held	
	2020	2019	2020	2019	2020	2019
	(Rupees in '000)					
Public/ Government	16,804,051	3,580,870	–	–	–	–
Private	1,164,192	2,479,999	–	–	–	–
	17,968,243	6,060,869	–	–	–	–

45.1.2 Investment in debt securities

Credit risk by industry sector

	Gross Investments		Non - performing Investments		Provision held	
	2020	2019	2020	2019	2020	2019
	(Rupees in '000)					
Chemical and pharmaceuticals	1,750,000	1,750,000	–	–	–	–
Electricity, gas, steam and air conditioning supply	357,975	676,933	–	–	–	–
Financials including government securities	998,156,946	712,505,023	118	118	118	118
Manufacture of cement	285,000	285,000	285,000	285,000	285,000	285,000
Manufacture of sugar	145,656	145,656	145,656	145,656	145,656	72,828
Manufacture of textiles	53,531	53,531	53,531	53,531	53,531	53,531
Telecommunications	–	114,747	–	114,747	–	114,747
Others	81,666	14,726,579	6,154	7,682	6,154	7,682
	1,000,830,774	730,257,469	490,459	606,734	490,459	533,906
**Credit risk by public / private sector**						
Public/ Government	989,024,844	718,043,511	–	–	–	–
Private	11,805,930	12,213,958	490,459	606,734	490,459	533,906
	1,000,830,774	730,257,469	490,459	606,734	490,459	533,906

Notes To The Consolidated Financial Statements
For the year ended December 31, 2020

45.1.3 Advances

Credit risk by industry sector

	Gross Advances		Non - performing Advances		Provision held	
	2020	2019	2020	2019	2020	2019
	(Rupees in '000)					
Agriculture, forestry and fishing	6,212,703	7,582,097	1,001,746	652,580	437,355	362,867
Construction	20,255,633	20,852,864	289,791	289,435	278,464	287,840
Electricity, gas, steam and air conditioning supply	40,921,064	44,370,647	376,717	379,846	374,996	378,125
Electronics and electrical appliances	6,135,405	4,760,213	102,262	113,496	101,825	107,776
Financials	17,004,242	21,334,593	462,665	532,286	462,665	532,286
Footwear and Leather garments	3,972,108	4,566,500	170,131	173,985	169,736	173,590
Human health and social work activities	922,333	2,760,760	45,596	50,211	34,973	34,981
Individuals	49,897,371	44,438,103	4,371,180	4,397,242	3,930,762	4,001,136
Manufacture of basic metals and metal products	18,604,646	16,420,515	3,028,467	3,104,026	3,018,387	2,892,814
Manufacture of cement	12,103,828	12,095,610	392,862	392,862	392,862	392,862
Manufacture of chemicals and pharmaceutical products	44,424,431	48,727,242	275,980	334,485	273,047	331,551
Manufacture of coke and refined petroleum products	5,582,529	14,789,736	412,061	399,317	411,445	398,701
Manufacture of food & beverages products	46,620,208	55,638,390	3,432,991	3,014,962	3,058,910	2,786,691
Manufacture of machinery, equipment and transport Equipment	2,182,220	4,835,798	433,943	435,301	396,551	434,527
Manufacture of rubber and plastics products	4,562,138	5,376,391	665,778	661,498	662,506	658,226
Manufacture of sugar	38,895,908	26,450,203	4,658,087	4,497,643	4,655,219	3,434,211
Manufacture of textiles	86,056,461	87,196,612	13,475,285	13,537,492	13,322,828	13,088,287
Mining and quarrying	5,033,270	4,252,835	5,019	7,286	5,019	7,286
Manufacturing of Pulp, Paper, Paperboard	3,851,182	5,021,705	179,539	242,331	150,661	213,453
Ship Breaking	6,707,314	8,366,211	4,348,014	2,436,614	4,348,014	2,436,614
Services	15,202,429	13,688,812	480,993	596,857	465,504	588,408
Telecommunications	18,287,167	15,883,537	42,798	42,798	42,798	42,798
Transportation and storage	83,652,075	66,336,431	75,901	93,955	66,253	84,307
Wholesale and retail traders	54,747,657	47,946,182	12,522,417	12,562,812	7,472,473	7,574,356
Others	6,531,453	8,179,645	695,295	856,366	635,098	694,068
	598,365,775	591,871,632	51,945,518	49,805,686	45,168,351	41,937,761
**Credit risk by public / private sector**						
Public/ Government	105,422,741	95,096,713	639,825	639,825	639,825	639,825
Private	492,943,034	496,774,919	51,305,693	49,165,861	44,528,526	41,297,936
	598,365,775	591,871,632	51,945,518	49,805,686	45,168,351	41,937,761

Notes To The Consolidated Financial Statements

For the year ended December 31, 2020

	2020	2019
	(Rupees in '000)	
**45.1.4 Contingencies and Commitments**		
**Credit risk by industry sector**		
Agriculture, forestry and fishing	2,148,032	626,318
Construction	22,705,666	28,790,117
Electricity, gas, steam and air conditioning supply	25,280,013	16,318,961
Electronics and electrical appliances	6,827,749	4,574,959
Financials	399,373,812	578,498,188
Footwear and Leather garments	517,178	123,152
Human health and social work activities	1,612,922	409,816
Individuals	2,671,529	6,205,880
Manufacture of basic metals and metal products	10,694,842	6,414,150
Manufacture of cement	4,755,354	1,761,514
Manufacture of chemicals and pharmaceutical products	26,547,957	19,976,862
Manufacture of coke and refined petroleum products	8,329,298	15,369,784
Manufacture of food & beverages products	20,251,341	19,550,105
Manufacture of machinery, equipment and transport Equipment	8,946,430	6,790,046
Manufacture of rubber and plastics products	4,968,143	2,162,953
Manufacture of sugar	4,623,950	3,790,969
Manufacture of textiles	37,630,523	35,676,318
Mining and quarrying	144,328	460,490
Manufacturing of Pulp, Paper, Paperboard	1,616,529	1,165,844
Ship Breaking	556,614	2,535,412
Services	58,522,756	55,448,997
Telecommunications	17,006,854	14,669,698
Transportation and storage	10,648,735	4,076,724
Wholesale and retail traders	26,096,091	17,521,657
Others	37,898,657	22,537,128
	740,375,303	865,456,042
**Credit risk by public / private sector**		
Public/ Government	229,256,802	321,606,927
Private	511,118,501	543,849,115
	740,375,303	865,456,042

45.1.5 Concentration of Advances

The Group top 10 exposures on the basis of total (funded and non-funded exposures) aggregated to Rs 235,823.529 million (2019: Rs. 236,769.236 million) are as following:

	2020	2019
	(Rupees in '000)	
Funded	75,373,723	80,717,454
Non Funded	160,449,536	156,051,782
Total Exposure	235,823,259	236,769,236

The sanctioned limits against these top 10 exposures aggregated to Rs 311,201.617 million (2019: 291,923.228 million)

There is no provision against these top 10 exposures.

Notes To The Consolidated Financial Statements

For the year ended December 31, 2020

45.1.6 Advances - Province/Region-wise Disbursement & Utilization

2020

Province / Region	Disbursements	Utilization					
		Punjab	Sindh	KPK including FATA	Balochistan	Islamabad	AJK including Gilgit Balochistan
	(Rupees in '000)						
Punjab	731,022,299	683,463,985	39,386,981	6,212,721	337,894	1,620,718	–
Sindh	555,378,201	12,974,461	495,054,217	6,776,970	40,570,866	1,687	–
KPK including FATA	4,893,018	192,363	–	4,692,336	–	8,319	–
Balochistan	1,728,874	–	10,145	–	1,718,729	–	–
Islamabad	32,173,536	2,558,963	2,246	2,293,047	–	27,319,280	–
AJK including Gilgit-Baltistan	340,864	68,573	–	–	–	27,915	244,376
Total	1,325,536,792	699,258,345	534,453,589	19,975,074	42,627,489	28,977,919	244,376

2019

Province / Region	Disbursements	Utilization					
		Punjab	Sindh	KPK including FATA	Balochistan	Islamabad	AJK including Gilgit Balochistan
	(Rupees in '000)						
Punjab	753,218,687	678,763,640	60,879,964	10,039,632	323,686	3,210,918	847
Sindh	688,753,937	12,583,438	605,024,224	3,370,558	67,547,890	227,827	–
KPK including FATA	5,481,487	73,656	–	5,407,831	–	–	–
Balochistan	1,251,413	–	9,294	–	1,242,119	–	–
Islamabad	43,495,899	5,008,912	228,682	1,789,813	–	36,393,156	75,336
AJK including Gilgit-Baltistan	368,360	29,738	–	–	–	–	338,622
Total	1,492,569,783	696,459,384	666,142,164	20,607,834	69,113,695	39,831,901	414,805

45.2 Market Risk

Market Risk arises from changes in market rates such as Interest Rates, Foreign Exchange Rates, Equity Prices, credit spreads and/or commodity prices as well as their correlations and volatilities resulting in a loss to earnings and capital. Group is exposed to market risk primarily through its trading activities, which are centered in the Treasury and Foreign Exchange Group and the Capital Market Division. Market risk also arises from market-making, facilitation of client business and proprietary positions in equities, fixed income and interest rate products and foreign exchange, which exposes bank to interest rate risk, foreign exchange risk and equity price risk.

The Group Market Risk Management structure consists of Risk Management & Portfolio Review Committee (RM&PRC) of the Board, Management Credit and Risk Committee, ALCO and independent Market Risk Management Division reporting directly to Group Head Risk Management. Market Risk function works in close partnership with the business segments to identify and monitor market risks throughout the Bank and to define market risk policies and procedures. Market Risk seeks to facilitate efficient risk/return management decisions, reduce volatility in operating performance and provide transparency into the Bank's market risk profile for senior management, the Board of Directors and regulators. Market risk authority, including both approval of market risk limits and approval of market risks is vested in the ALCO.

In line with regulatory requirements, Group has clearly defined, in its Global Risk Management policy, the positions which shall be subject to market risk. The definition covers the accounting classifications as well as positions booked by different business groups under "Available for Sale" category. The assets subject to trading book treatment are frequently, mostly on daily basis, valued and actively managed. The positions which does not fulfill the criteria of Trading book falls under the Banking Book and are treated as per SBP requirements.

Notes To The Consolidated Financial Statements

For the year ended December 31, 2020

The Group measures and manages Market Risk by using different risk parameters with combinations of various limits. Board approved Global Risk Management Policy provides guidelines for assuming controlled market risk, its monitoring and management. The approved limits are compared with the numbers generated by the market risk management systems based on the trading activity and the outstanding positions.

Besides conventional methods, the Group also uses VaR (Value at Risk) technique for market risk assessment of positions assumed by its treasury and capital market groups. In-house based solutions are used for calculating mark to market value of positions and generating VaR (value at risk) and sensitivity numbers. Thresholds for different positions are established to compare the expected losses at a given confidence level and over a specified time horizon.

A framework of stress testing, scenario analysis and reverse stress tests covering both banking and trading books as per SBP guidelines is also in place. The results of the stress tests are reviewed by senior management and also reported to the SBP.

The Group is also exposed to interest rate risk both in trading and banking books. Risk parameters along with the marked to market values of government securities held by the Bank's treasury are generated on daily basis. The risk parameters include duration, PVBP, and VaR on individual security basis as well as on portfolio basis. These reports are presented to the senior management for review on a daily basis.

45.2.1 Balance sheet split by trading and banking books

	2020			2019		
	Banking book	Trading book	Total	Banking book	Trading book	Total
	(Rupees in '000)					
Cash and balances with treasury banks	132,053,041	–	132,053,041	142,957,358	–	142,957,358
Balances with other banks	29,011,521	–	29,011,521	21,371,753	–	21,371,753
Lendings to financial institutions	17,968,243	–	17,968,243	6,060,869	–	6,060,869
Investments	24,028,377	1,012,189,158	1,036,217,535	41,407,690	716,033,900	757,441,590
Advances	547,685,708	–	547,685,708	548,472,860	–	548,472,860
Fixed assets	63,679,312	–	63,679,312	64,201,807	–	64,201,807
Intangible assets	1,867,244	–	1,867,244	1,978,975	–	1,978,975
Other assets	62,793,791	–	62,793,791	69,729,659	–	69,729,659
	879,087,237	1,012,189,158	1,891,276,395	896,180,971	716,033,900	1,612,214,871

45.2.2 Foreign Exchange Risk

Foreign exchange risk exposes the Group to changes in the values of current holdings and future cash flows denominated in currencies other than home currency due to the exchange rate fluctuation and volatility. The types of instruments exposed to this risk include investments in foreign branches, foreign currency-denominated loans, foreign currency-denominated deposits, future cash flows in foreign currencies arising from foreign exchange transactions, etc.

The core objective of foreign exchange risk management is to ensure the foreign exchange exposure of the Group remain within defined risk appetite and insulate group against undue losses that may arise due to volatile movements in foreign exchange rates or interest rates.

Limit structure to manage Foreign exchange risk including gap limits on different tenors in major currencies are in place to control risk. Groips's net open position and foreign exchange exposure limit (FEEL) is monitored and reported on intra-day and day end basis. Foreign exchange risk parameters including VaR is generated and monitored on daily basis. Stress testing of foreign exchange portfolio and its reporting to senior management and RM&PRC of the Board is a regular feature.

Notes To The Consolidated Financial Statements

For the year ended December 31, 2020

	2020				2019			
	Foreign currency Assets	Foreign currency liabilities	Off--balance sheet items	Net foreign currency exposure	Foreign currency Assets	Foreign currency liabilities	Off--balance sheet items	Net foreign currency exposure
	(Rupees in '000)							
United States Dollar	49,879,275	58,098,426	8,304,216	85,065	42,949,125	61,958,946	20,204,447	1,194,626
Sri Lankan Rupees	144,228	–	–	144,228	–	164,290	148,959	(15,331)
Arab Emirates Dirham	–	248,645	39,598	(209,047)	194,163	949,404	590,192	(165,049)
Euro	2,846,513	7,017,270	4,194,638	23,881	438,134	4,960,868	4,564,076	41,342
Great Britain Pound Sterling	2,780,633	6,826,427	4,016,794	(29,000)	703,957	5,585,201	4,822,148	(59,096)
Japanese Yen	34,647	160	22,007	56,494	19,750	4,627	17,723	32,846
Other currencies	384,723	–	47,086	431,809	133,740	–	31,769	165,509
	56,070,019	72,190,928	16,624,339	503,430	44,438,869	73,623,336	30,379,314	1,194,847

	2020		2019	
	Banking book	Trading book	Banking book	Trading book
	(Rupees in '000)			
Impact of 1% change in foreign exchange rates on				
- Profit and loss account	-	5,034	-	11,948

45.2.3 Equity position Risk

Group's proprietary positions in the equity instruments exposes it to the equity price risk in its trading and banking books. Equity price risk is managed by applying trading limit, scrip-wise and portfolio wise nominal limits. VaR analysis and stress testing of the equity portfolio are also performed and reported to senior management on a daily basis. The stress test for equity price risk assesses the impact of the fall in the stock market index using certain assumptions. In addition to this stress testing, historical scenario analysis on equities is also performed periodically as advised by the State Bank of Pakistan through Guideline on Stress Testing.

	2020		2019	
	AFS	HFT	AFS	HFT
	(Rupees in '000)			
Impact of 5% change in equity prices on				
- Profit and loss account	–	63,472	–	57,548
- Other comprehensive income	962,069	–	796,211	–

45.2.4 Yield / Interest Rate Risk in the Banking Book (IRRBB)-Basel II Specific

Yield/ Interest rate sensitivity position for on-balance sheet instruments is based on the earlier of contractual re-pricing or maturity date and for off-balance sheet instruments is based on settlement date.

The increase / (decrease) in earnings due to change in the interest rate is as follows:

	2020		2019	
	Banking book	Trading book	Banking book	Trading book
	(Rupees in '000)			
Impact of 1% change in interest rates on				
- Profit and loss account	(3,870,506)	5,305,338	2,470,389	24,844
- Other comprehensive income	–	(7,497,582)	–	6,819,634

The Group has classified Available for Sale investments as Trading in Basel-II.

45.2.5 Mismatch of Interest Rate Sensitive Assets and Liabilities

	2020											
	Effective yield / interest rate	Total	Exposed to Yield/ Interest risk									Non-interest bearing financial instruments
			Upto 1 month	Over 1 to 3 months	Over 3 to 6 months	Over 6 months to 1 year	Over 1 to 2 years	Over 2 to 3 years	Over 3 to 5 years	Over 5 to 10 years	Above 10 years	
			(Rupees in '000)									
**On-balance sheet financial instruments**												
**Assets**												
Cash and balances with treasury banks	-	132,053,041	6,810,656	-	-	-	-	-	-	-	-	125,242,385
Balances with other banks	0.21%	29,011,521	2,557,720	799,172	-	-	-	-	-	-	-	25,654,629
Lending to financial institutions	7.27%	17,968,243	17,968,243	-	-	-	-	-	-	-	-	-
Investments	10.77%	1,036,217,535	183,723,956	426,084,702	170,130,910	62,818,947	24,457,188	41,949,392	30,794,310	71,118,956	-	25,139,174
Advances	9.34%	530,862,738	432,796,935	55,725,610	5,822,045	12,758,741	3,619,904	1,742,088	4,803,725	1,859,527	6,139,018	5,595,145
Other assets		52,518,097	-	-	-	-	-	-	-	-	-	52,518,097
		1,798,631,175	643,857,510	482,609,484	175,952,955	75,577,688	28,077,092	43,691,480	35,598,035	72,978,483	6,139,018	234,149,430
**Liabilities**												
Bills payable		26,451,513	-	-	-	-	-	-	-	-	-	26,451,513
Borrowings	5.30%	184,577,340	126,736,400	19,225,222	5,798,255	1,899,960	8,816,080	3,763,698	4,831,690	13,506,035	-	-
Deposits and other accounts	4.50%	1,388,737,961	796,398,749	21,243,238	13,630,588	30,543,903	1,998,833	115,810	1,689,628	212,000	-	522,905,212
Other liabilities		68,843,336	-	-	-	-	-	-	-	-	-	68,843,336
		1,668,610,150	923,135,149	40,468,460	19,428,843	32,443,863	10,814,913	3,879,508	6,521,318	13,718,035	-	618,200,061
**On-balance sheet gap**		130,021,025	(279,277,639)	442,141,024	156,524,112	43,133,825	17,262,179	39,811,972	29,076,717	59,260,448	6,139,018	(384,050,631)
**Off-balance sheet financial instruments**												
FX options purchase		182,800	122,370	60,430	-	-	-	-	-	-	-	-
Forward purchase of Government securities		11,089,775	11,089,775	-	-	-	-	-	-	-	-	-
Cross Currency Swaps purchase		1,975,311	339,938	-	210,505	87,318	547,272	-	790,278	-	-	-
Foreign exchange contracts purchase		172,137,589	69,994,741	67,331,082	26,239,931	8,571,835	-	-	-	-	-	-
		185,385,475	81,546,824	67,391,512	26,450,436	8,659,153	547,272	-	790,278	-	-	-
FX options sale		182,800	122,370	60,430	-	-	-	-	-	-	-	-
Cross Currency Swaps sale		2,130,472	340,109	-	210,505	242,308	547,272	-	790,278	-	-	-
Foreign exchange contracts sale		155,508,653	58,735,248	59,516,527	23,227,903	14,028,975	-	-	-	-	-	-
		157,821,925	59,197,727	59,576,957	23,438,408	14,271,283	547,272	-	790,278	-	-	-
**Off-balance sheet gap**		27,563,550	22,349,097	7,814,555	3,012,028	(5,612,130)	-	-	-	-	-	-
Total Yield/Interest Risk Sensitivity Gap			(256,928,542)	449,955,579	159,536,140	37,521,695	17,262,179	39,811,972	29,076,717	59,260,448	6,139,018	
Cumulative Yield/Interest Risk Sensitivity Gap			(256,928,542)	193,027,037	352,563,177	390,084,872	407,347,051	447,159,023	476,235,740	535,496,188	541,635,206	

Notes To The Consolidated Financial Statements

For the year ended December 31, 2020

	2019											
	Effective yield / interest rate	Total	Exposed to Yield/ Interest risk									Non-interest bearing financial instruments
			Upto 1 month	Over 1 to 3 months	Over 3 to 6 months	Over 6 months to 1 year	Over 1 to 2 years	Over 2 to 3 years	Over 3 to 5 years	Over 5 to 10 years	Above years	
	(Rupees in '000)											
**On-balance sheet financial instruments**												
**Assets**												
Cash and balances with treasury banks	0.70%	142,957,358	10,517,551	-	-	-	-	-	-	-	-	132,439,807
Balances with other banks	0.55%	21,371,753	7,176,640	1,340,374	-	-	-	-	-	-	-	12,854,739
Lending to financial institutions	10.91%	6,060,869	2,689,205	3,371,664	-	-	-	-	-	-	-	-
Investments	11.47%	757,441,590	180,997,048	58,772,960	43,600,582	187,051,328	105,606,890	14,900,121	60,307,907	83,531,045	-	22,673,709
Advances	11.70%	537,499,107	484,867,924	29,157,490	6,805,578	3,157,363	1,816,256	3,009,684	1,481,043	1,759,402	5,444,367	-
Other assets		58,219,618	-	-	-	-	-	-	-	-	-	58,219,618
		1,523,550,295	686,248,368	92,642,488	50,406,160	190,208,691	107,423,146	17,909,805	61,788,950	85,290,447	5,444,367	226,187,873
**Liabilities**												
Bills payable		12,795,325	-	-	-	-	-	-	-	-	-	12,795,325
Borrowings	7.84%	92,859,968	55,458,386	11,294,285	8,605,720	1,507,529	2,097,344	2,217,657	3,572,934	8,106,113	-	-
Deposits and other accounts	5.96%	1,226,593,025	686,055,880	35,105,817	22,527,727	24,408,630	3,459,232	2,002,175	1,270,783	-	-	451,762,781
Other liabilities		85,297,242	-	-	-	-	-	-	-	-	-	85,297,242
		1,417,545,560	741,514,266	46,400,102	31,133,447	25,916,159	5,556,576	4,219,832	4,843,717	8,106,113	-	549,855,348
**On-balance sheet gap**		106,004,735	(55,265,898)	46,242,386	19,272,713	164,292,532	101,866,570	13,689,973	56,945,233	77,184,334	5,444,367	(323,667,475)
**Off-balance sheet financial instruments**												
FX options purchase		431,449	204,798	226,651	-	-	-	-	-	-	-	-
Outright purchase of Government Securities		82,284,304	82,284,304	-	-	-	-	-	-	-	-	-
Cross currency swaps - purchase		4,428,663	-	311,405	-	765,490	1,522,069	857,049	972,650	-	-	-
Interest Rate Swaps - purchase		316,500	-	-	-	316,500	-	-	-	-	-	-
Foreign exchange contracts purchase		216,647,656	81,562,475	85,102,271	45,299,680	4,683,230	-	-	-	-	-	-
		304,108,572	164,051,577	85,640,327	45,299,680	5,765,220	1,522,069	857,049	972,650	-	-	-
FX options sale		431,449	204,798	226,651	-	-	-	-	-	-	-	-
Forward sale of Government securities		5,412,334	5,412,334	-	-	-	-	-	-	-	-	-
Cross Currency Swaps - sale		4,636,745	-	311,405	52,745	765,490	1,677,406	857,049	972,650	-	-	-
Foreign exchange contracts sale		188,243,433	69,140,866	67,523,912	50,136,199	1,442,456	-	-	-	-	-	-
		198,723,961	74,757,998	68,061,968	50,188,944	2,207,946	1,677,406	857,049	972,650	-	-	-
**Off-balance sheet gap**		105,384,611	89,293,579	17,578,359	(4,889,264)	3,557,274	(155,337)	-				
**Total Yield/Interest Risk Sensitivity Gap**			34,027,681	63,820,745	14,383,449	167,849,806	101,711,233	13,689,973	56,945,233	77,184,334	5,444,367	
**Cumulative Yield/Interest Risk Sensitivity Gap**			34,027,681	97,848,426	112,231,875	280,081,681	381,792,914	395,482,887	452,428,120	529,612,454	535,056,821	

	2020	2019
	(Rupees in '000)	
**Reconciliation to total assets**		
**Balance as per balance sheet**	1,891,276,395	1,612,214,871
Less: Non financial assets		
Islamic financing and related assets	16,822,970	10,973,753
Fixed assets	63,679,312	64,201,807
Intangible assets	1,867,244	1,978,975
Other assets	10,275,694	11,510,041
	92,645,220	88,664,576
Total financial assets	1,798,631,175	1,523,550,295

	2020	2019
	(Rupees in '000)	
**Reconciliation to total assets**		
**Balance as per balance sheet**	1,698,285,012	1,440,868,054
Less: Non financial liabilities		
Other liabilities	22,183,822	17,108,271
Deferred tax liability	7,491,040	6,214,223
	29,674,862	23,322,494
Total financial liabilities	1,668,610,150	1,417,545,560

Yield risk is the risk of decline in earnings due to adverse movement of the yield curve.

Interest rate risk is the risk that the value of the financial instrument will fluctuate due to changes in the market interest rates.

45.3 Operational Risk

Operational risk is the risk of loss resulting from inadequate or failed internal processes, people, and systems or from external events. This definition includes legal risks but excludes strategic and reputational risks.

The Group operational risk management framework, as laid down in the Global Risk Management Policy, duly approved by BOD, is flexible enough to implement in stages and permits the overall risk management approach to evolve in the light of organizational learning and the future needs of the Group. Operational loss events are reviewed and appropriate corrective actions taken on an ongoing basis, including measures to improve control procedures with respect to design and operative effectiveness.

Operational Risk Management helps the Group understand risks and improve mitigating controls so as to minimize operational risks that are inherent in almost all areas of the Group. Going forward, the Group will further strengthen its risk function, policies and procedures to facilitate its operations and improve quality of assets to safeguard interest of depositors.

45.3.1 Operational Risk-Disclosures Basel II Specific

Currently, the Group is reporting operational risk capital charge under Basic Indicator Approach (BIA). The Group took a number of initiative with respect to operational risk management like using Key Risk Indicators (Kris), Loss events database and Risk & Control Self Assessments (ROSA) to manage its operational risk effectively

In accordance with the Operational Risk Management (OR) regulations, policy and framework, a database covering operational risk events is being maintained using a state of the art software solution, which has enhanced features and a better workflow management. This new software has further augmented Group's capacity to capture and report operational risk events and Kris. The software is also capable of generating periodical regulatory and management reports. Periodical updates on Operational Risk events are presented to senior management and the Risk Management and Portfolio Review Committee of the Board.

Notes To The Consolidated Financial Statements

For the year ended December 31, 2020

45.4 Liquidity Risk

Liquidity represents the ability to fund assets and meet obligations as they become due. The Group understands that liquidity does not come for free, and surplus liquidity has an opportunity cost which needs to be recognized. Liquidity risk is a risk of not being able to obtain funds at a reasonable price within a reasonable time period to meet obligations as they become due. Liquidity is essential to the ability to operate financial services businesses and, therefore, the ability to maintain surplus levels of liquidity through economic cycles is crucial. Particularly during periods of adverse conditions, liquidity management is among the most important activities that the MCB conducts during both normal and stress periods. MCB recognizes that liquidity risk can arise from the Group activities and can be grouped into three categories:

- Inflows/Outflows from on-balance sheet items (other than marketable securities and wholesale borrowings) and off-balance sheet items;
- Marketability of trading securities; and
- Capacity to borrow from the wholesale markets for funding as well as trading activities.

Liquidity Management

The Asset Liability Management Committee of the group has the responsibility for the formulation of overall strategy and oversight of the Asset Liability Management function. Board has approved a comprehensive Liquidity Risk Policy (part of Risk Management Policy), which stipulates policies regarding maintenance of various ratios, funding preferences, and evaluation of group's liquidity under normal and stress scenarios. A framework to assess the maturity profile of non-contractual assets and liabilities is in place to supplement the liquidity management. Group comprehensive liquidity management framework assists it to closely watch the liquidity position through monitoring of early warning indicators and stress testing, to ensure effective and timely decision making.

Group liquidity risk management framework is designed to identify measure and manage in a timely manner the liquidity risk position of the Group. The underlying policies and procedures include: Global Risk Management policy, Global Treasury Policy, Investment policy, Contingency Funding Plan, Liquidity Strategy and Limit Structure which are reviewed and approved regularly by the senior management /Board members. Moreover; the Group also prepares a 'Contingency Funding Plan' (CFP) to address liquidity issues in time of stress/crises situation containing early warning indicators to pre-empt unforeseen liquidity crises. Group conducts Liquidity Risk Analysis on regular basis as well as Maturity of gaps are also reviewed in order to ensure diversification in terms of tenors. Group liquidity risk framework envisages to project the Group funding position during temporary and long-term liquidity changes, including those caused by liability erosion and explicitly identifying quantifying and ranking all sources of funding preferences, such as reducing assets, modifying or increasing liability structure; and using other alternatives for controlling statement of financial position changes. Group performs regular liquidity stress tests as part of its liquidity monitoring activities. The purpose of the liquidity stress tests is intended to ensure sufficient liquidity for the group under both idiosyncratic and systemic market stress conditions. Group liquidity risk management approach involves intraday liquidity management, managing funding sources and evaluation of structural imbalances in balance sheet structure.

In view of the relaxation granted by SBP for deferral of principal and markup and for rescheduling / restructuring of loans there will be an impact on the maturity profile of the Group. The Asset and Liability Committee (ALCO) of the Group is monitoring the liquidity position and the Group is confident that the liquidity buffer currently maintained is sufficient to cater to any adverse movement in the cash flow maturity profile

Intraday Liquidity Management

Intraday liquidity management is about managing the daily payments and cash flows. Group has policies to ensure that sufficient cash is maintained during the day to make payments through local payment system. The policy of the Group is to maintain adequate liquidity at all times, in all geographical locations and for all currencies and hence to be in a position, in the normal course of business, to meet obligations, repay depositors and fulfill commitments.

Managing Funding Sources

Managing funding sources, as per policy Group maintain a portfolio of marketable securities that can either be sold outright or sold through a repurchase agreement to generate cash flows for meeting unexpected liquidity requirement. As a part of liquidity management Group maintains borrowing relationships to ensure the continued access to diverse market of funding sources. Group's sound credit rating together with excellent market reputation has enabled Group to secure ample call lines with local and foreign groups. The level of liquidity reserves as per regulatory requirements also mitigates risks. Group's investment in marketable securities is much higher than the Statutory Liquidity requirements.

45.4.1 Maturities of Assets and Liabilities - based on contractual maturity of the assets and liabilities of the Group

	2020													
	Total	Upto 1 day	Over 1 to 7 days	Over 7 to 14 days	Over 14 days to 1 months	Over 1 or 2 months	Over 2 to 3 months	Over 3 to 6 months	Over 6 to 9 months	Over 9 months to 1 years	Over 1 to 2 years	Over 2 to 3 years	Over 3 to 5 years	Over 5 years
	(Rupees in '000)													
**Assets**														
Cash and balances with treasury banks	132,053,041	132,053,041	-	-	--	-	-	-	-	-	-	-	-	-
Balances with other banks	29,011,521	23,026,781	1,888,376	1,249,151	2,048,182	-	799,031	-	-	-	-	-	-	-
Lending to financial institutions	17,968,243	2,692,982	15,275,261	-	-	-	-	-	-	-	-	-	-	-
Investments	1,036,217,535	2,115,960	744,830	91,856,505	72,413,931	159,537,887	179,728,312	114,920,205	60,920,079	6,328,360	29,707,053	75,507,721	46,183,005	196,253,687
Advances	547,685,708	71,415,264	18,829,281	7,175,588	23,201,609	30,523,564	34,544,271	59,677,666	39,860,068	38,419,706	58,572,922	48,296,903	69,731,805	47,437,061
Fixed assets	63,679,312	9,138	54,832	63,971	273,734	501,067	398,581	1,195,045	1,193,172	1,188,330	4,288,513	4,258,016	5,135,853	45,119,060
Intangible assets	1,867,244	1,979	11,878	13,857	44,088	87,266	72,153	216,545	216,582	216,530	464,642	45,265	159,143	317,316
Deferred tax assets	3,925,760	7,623	45,740	53,364	129,597	222,416	236,324	693,727	705,676	693,727	631,898	505,668	-	-
Other assets	62,793,791	519,422	2,287,085	7,867,650	6,658,755	10,076,645	10,344,951	2,393,673	913,569	400,930	2,260,624	7,954,884	11,115,603	-
	1,895,202,155	231,842,190	39,137,283	108,280,086	104,769,896	200,948,845	226,123,623	179,096,861	103,809,146	47,247,583	95,925,652	136,568,457	132,325,409	289,127,124
**Liabilities**														
Bills payable	26,451,513	881,718	5,290,301	6,172,020	14,107,474	-	-	-	-	-	-	-	-	-
Borrowings	184,577,340	27,117,387	93,035,233	1,537,110	5,046,669	15,158,024	4,067,198	5,798,255	1,010,722	889,239	8,816,080	3,763,698	4,831,690	13,506,035
Deposits and other accounts	1,388,737,961	1,277,556,493	6,058,339	6,678,164	20,097,720	15,871,497	11,274,222	15,154,216	17,758,355	14,025,638	2,193,993	158,796	1,698,528	212,000
Deferred tax liabilities	11,416,800	33,252	35,714	30,577	10,994	202,719	28,870	98,971	739,824	212,623	698,992	1,954,940	2,506,244	4,863,080
Other liabilities	91,027,158	12,566,376	2,742,643	4,125,090	5,965,945	5,729,410	6,105,290	13,558,440	2,237,294	2,173,027	4,232,223	9,889,368	16,491,972	5,210,080
	1,702,210,772	1,318,155,226	107,162,230	18,542,961	45,228,802	36,961,650	21,475,580	34,609,882	21,746,195	17,300,527	15,941,288	15,766,802	25,528,434	23,791,195
**Net assets**	192,991,383	(1,086,313,036)	(68,024,947)	89,737,125	59,541,094	163,987,195	204,648,043	144,486,979	82,062,951	29,947,056	79,984,364	120,801,655	106,796,975	265,335,929
Share capital	11,850,600													
Reserves	81,060,051													
Surplus on revaluation of assets	28,803,351													
Unappropriated profit	70,498,820													
Minority interest	778,561													
	192,991,383													

Notes To The Consolidated Financial Statements

For the year ended December 31, 2020

	2019													
	Total	Upto 1 day	Over 1 to 7 days	Over 7 to 14 days	Over 14 days to 1 months	Over 1 or 2 months	Over 2 to 3 months	Over 3 to 6 months	Over 6 to 9 months	Over 9 months to 1 years	Over 1 to 2 years	Over 2 to 3 years	Over 3 to 5 years	Over 5 years
	(Rupees in '000)													
**Assets**														
Cash and balances with treasury banks	142,957,358	142,957,358	-	-	-	-	-	-	-	-	-	-	-	-
Balances with other banks	21,371,753	17,709,050	541,582	1,083,933	696,814	1,340,374	-	-	-	-	-	-	-	-
Lending to financial institutions	6,060,869	209,206	500,000	-	1,980,000	3,371,663	-	-	-	-	-	-	-	-
Investments	757,441,590	1,561,402	12,087,461	1,484,042	165,948,840	4,525,813	39,055,937	37,983,828	101,715,552	86,779,634	111,060,640	18,820,516	65,033,046	111,384,879
Advances	548,472,860	116,563,089	12,087,746	9,807,984	34,999,908	27,246,925	34,646,455	65,929,233	28,106,558	35,278,081	46,252,132	39,070,405	53,513,936	44,970,408
Fixed assets	64,201,807	9,316	55,896	65,212	254,848	404,607	404,874	1,212,956	1,213,009	1,214,158	4,062,375	4,108,763	5,466,376	45,729,417
Intangible assets	1,978,975	29,291	10,296	12,012	38,546	62,705	63,157	189,591	189,829	189,836	442,526	173,054	506,395	71,737
Deferred tax assets	2,918,483	11,761	28,361	33,088	80,356	137,078	146,531	430,140	435,730	430,140	210,356	974,942	-	-
Other assets	69,729,659	481,675	3,757,122	8,150,816	8,254,924	12,645,114	13,810,916	3,683,264	3,547,514	457,875	1,214,143	2,141,963	11,584,333	-
	1,615,133,354	279,532,148	29,068,464	20,637,087	212,254,236	49,734,279	88,127,870	109,429,012	135,208,192	124,349,724	163,242,172	65,289,643	136,104,086	202,156,441
**Liabilities**														
Bills payable	12,795,325	426,511	2,559,065	2,985,576	6,824,173	-	-	-	-	-	-	-	-	-
Borrowings	92,859,968	19,690,252	24,261,935	5,906,627	5,599,571	7,876,054	3,418,232	8,605,720	704,824	802,705	2,097,344	2,217,657	3,572,934	8,106,113
Deposits and other accounts	1,226,593,025	1,100,034,930	4,344,655	8,009,608	13,914,022	16,239,575	24,611,305	23,589,167	17,002,990	11,952,021	3,482,589	2,137,880	1,274,283	-
Deferred tax liabilities	9,132,706	10,685	16,352	12,337	(30,763)	19,132	(96,955)	81,403	71,253	(18,568)	594,557	321,640	2,874,074	5,277,559
Other liabilities	102,405,513	10,311,942	8,025,836	7,761,515	17,612,872	5,700,838	5,938,865	11,057,222	6,614,626	2,060,339	2,694,699	3,753,391	14,544,640	6,328,728
	1,443,786,537	1,130,474,320	39,207,843	24,675,663	43,919,875	29,835,599	33,871,447	43,333,512	24,393,693	14,796,497	8,869,189	8,430,568	22,265,931	19,712,400
**Net assets**	171,346,817	(850,942,172)	(10,139,379)	(4,038,576)	168,334,361	19,898,680	54,256,423	66,095,500	110,814,499	109,553,227	154,372,983	56,859,075	113,838,155	182,444,041
Share capital	11,850,600													
Reserves	77,894,829													
Surplus on revaluation of assets	24,752,206													
Unappropriated profit	56,108,779													
Non-controlling interest	740,403													
	171,346,817													

45.4.2 Maturities of assets and liabilities - based on expected maturities of the assets and liabilities of the Group

	2020									
	Total	Upto 1 month	Over 1 to 3 months	Over 3 to 6 months	Over 6 to months to 1 year	Over 1 to 2 years	Over 2 to 3 years	Over 3 to 5 years	Over 5 to 5 years	Above 10 years
	(Rupees in '000)									
**Assets**										
Cash and balances with treasury banks	132,053,041	132,053,041	–	–	-	-	–	–	–	–
Balances with other banks	29,011,521	28,212,490	799,031	–	-	-	–	–	–	–
Lending to financial institutions	17,968,243	17,968,243	–	–	-	-	–	–	–	–
Investments	1,036,217,535	166,114,420	339,389,280	114,979,116	67,384,316	29,832,802	75,633,470	46,243,587	189,391,593	7,248,951
Advances	547,685,708	76,970,968	45,417,484	33,943,819	64,487,430	86,858,208	78,919,201	96,374,067	54,063,767	10,650,764
Fixed assets	63,679,312	401,677	899,648	1,195,045	2,381,501	4,288,513	4,258,016	5,084,949	8,481,036	36,688,927
Intangible assets	1,867,244	71,802	159,419	216,545	433,112	509,907	–	159,143	43,190	274,126
Deferred tax assets	3,925,760	236,324	458,740	693,727	1,399,403	631,898	505,668	–	–	–
Other assets	62,793,791	17,332,911	20,411,437	2,403,832	1,314,500	2,260,624	7,954,884	11,115,603	–	–
	1,895,202,155	439,361,876	407,535,039	153,432,084	137,400,262	124,381,952	167,271,239	158,977,349	251,979,586	54,862,768
**Liabilities**										
Bills payable	26,451,513	26,451,513	–	–	–	-	-	–	–	–
Borrowings	184,577,340	126,736,400	19,225,222	5,798,255	1,899,960	8,816,080	3,763,698	4,831,690	13,506,035	–
Deposits and other accounts	1,388,737,961	91,320,307	60,203,123	59,578,918	57,889,894	333,580,267	331,545,069	333,084,802	121,535,581	–
Deferred tax liabilities	11,416,800	111,918	231,543	98,660	951,375	698,669	1,954,617	2,483,113	3,097,007	1,789,898
Other liabilities	91,027,158	25,400,055	11,830,725	13,562,414	4,410,321	4,232,223	9,889,368	16,491,972	4,748,747	461,333
	1,702,210,772	270,020,193	91,490,613	79,038,247	65,151,550	347,327,239	347,152,752	356,891,577	142,887,370	2,251,231
**Net assets**	192,991,383	169,341,683	316,044,426	74,393,837	72,248,712	(222,945,287)	(179,881,513)	(197,914,228)	109,092,216	52,611,537
Share capital	11,850,600									
Reserves	81,060,051									
Surplus on revaluation of assets	28,803,351									
Unappropriated profit	70,498,820									
Minority interest	778,561									
	192,991,383									

Notes To The Consolidated Financial Statements
For the year ended December 31, 2020

	2019									
	Total	Upto 1 month	Over 1 to 3 months	Over 3 to 6 months	Over 6 to months to 1 year	Over 1 to 2 years	Over 2 to 3 years	Over 3 to 5 years	Over 5 to 5 years	Above 10 years
	(Rupees in '000)									
**Assets**										
Cash and balances with treasury banks	142,957,358	142,957,358	–	–	–	–	–	–	–	–
Balances with other banks	21,371,753	20,031,379	1,340,374	–	–	–	–	–	–	–
Lending to financial institutions	6,060,869	2,689,205	3,371,664	–	–	–	–	–	–	–
Investments	757,441,590	180,171,442	43,831,910	38,108,079	187,726,109	111,338,352	19,095,957	65,310,758	105,613,987	6,244,996
Advances	548,472,860	100,517,088	63,393,356	57,113,936	53,862,562	72,777,509	65,172,731	81,649,276	44,506,083	9,480,319
Fixed assets	64,201,807	387,631	718,212	1,315,538	2,432,421	4,062,375	2,828,912	6,746,400	6,073,232	39,637,086
Intangible assets	1,978,975	90,144	125,863	189,591	379,665	442,526	173,054	506,395	71,737	–
Deferred tax assets	2,918,483	153,566	283,609	430,140	865,870	210,356	974,942	–	–	–
Other assets	69,729,659	20,644,536	26,430,940	3,719,452	4,005,389	1,214,143	2,141,963	11,573,236	–	–
	1,615,133,354	467,642,349	139,495,928	100,876,736	249,272,016	190,045,261	90,387,559	165,786,065	156,265,039	55,362,401
**Liabilities**										
Bills payable	12,795,325	12,795,325	–	–	–	–	–	–	–	–
Borrowings	92,859,968	55,458,386	11,294,285	8,605,720	1,507,529	2,097,344	2,217,657	3,572,934	8,106,113	–
Deposits and other accounts	1,226,593,025	80,369,330	60,509,999	51,460,105	65,341,453	289,229,540	287,884,831	287,021,234	104,776,533	–
Deferred tax liabilities	9,132,706	53,110	(78,304)	36,504	51,724	594,557	320,845	2,874,074	2,711,159	2,569,037
Other liabilities	102,405,513	43,712,165	11,639,703	11,060,721	8,671,466	2,694,699	3,753,391	14,544,640	5,790,477	538,251
	1,443,786,537	192,388,316	83,365,683	71,163,050	75,572,172	294,616,140	294,176,724	308,012,882	121,384,282	3,107,288
**Net assets**	171,346,817	275,254,033	56,130,245	29,713,686	173,699,844	(104,570,879)	(203,789,165)	(142,226,817)	34,880,757	52,255,113
Share capital	11,850,600									
Reserves	77,894,829									
Surplus on revaluation of assets	24,752,206									
Unappropriated profit	56,108,779									
Non-controlling interest	740,403									
	171,346,817									

Liquidity Gap Reporting

When an asset or liability does not have any contractual maturity date, the period in which these are assumed to mature has been taken as the expected date of maturity. Group regularly conducts an objective and systematic behavioral study using regression analysis technique to ascertain the maturity of its non-contractual assets and liabilities. Core and non-core parts of the non-contractual assets and liabilities are segregated through the behavioral study. Non Core part is placed among the short term maturity buckets i.e. up to 1 Year based on the model results, whereas core part is distributed among the longer terms buckets based on the discussion and decision by the ALCO. Following percentages are used to distribute the core assets and liabilities among longer term buckets:

Over 1 to 2 Years	Over 2 to 3 Years	Over 3 to 5 Years	Over 5 to 10 Years
30%	30%	30%	10%

Notes To The Consolidated Financial Statements

For the year ended December 31, 2020

45.5 Derivative Risk

Most business clients have either interest rate exposures arising from debt financing or currency exposures arising out of commercial transactions from import and export of goods. Businesses face the risk of sudden movements in interest rates or foreign exchange rates that may adversely affect their profitability. Group provides solutions to this problem through its derivatives desk in major types of derivative instruments i.e.; forwards, futures, swaps and options. As an Authorized Derivative Dealer (ADD), Group is an active participant in Derivative market and has flexibility in providing a broad range of derivatives products covering both hedging and market making to satisfy customers' needs. As an ADD, the Group offers derivative products which are permitted under the Financial Derivative Business Regulations (FDBR) or as permitted by the State Bank of Pakistan. Before executing Derivative transactions, the Group ensures that the clients understand the risk and reward associated with the derivative being offered. Derivative transactions are executed with appropriate clients only.

Risk management activities take place at the following different levels.

Strategic Level:

By senior management Assets and Liabilities Management Committee (ALCO), Management Credit and Risk Committee (MC&RC) and the Board of Directors to institute a risk management framework and to ensure provision of all resources and support required for effective risk management on Group-wide basis. The Board provides the overall limits/thresholds for derivatives business.

Macro Level

By Treasury and FX Group and Risk Management Group, responsible for policy formulation, procedure development and implementation, monitoring and reporting.

Micro Level:

Treasury Derivatives and Structured Product Desk where risks are actually created and Treasury Operations for settlements of the transactions.

Derivative Risk Management caters the following risks:-

Market Risk arises from changes in market rates such as Interest Rates, Foreign Exchange Rates, Equity Prices, credit spreads and/or commodity prices as well as their correlations and volatilities resulting in a loss to earnings and capital. In line with SBP's regulatory guidelines, Group hedges back-to-back all option transactions with other financial institutions. Group minimizes the exchange rate risk on its Cross Currency Swap Portfolio by hedging the exposure in interGroup market. Group also manages interest rate risk on its Interest Rate Derivatives and Cross Currency Swaps through various sensitivity limits approved by ALCO. Marked to market positions and sensitivity of the derivatives transactions are monitored on regular basis. All individual deals are approved at the appropriate level of authority after analyzing the risk and benefits associated with the deals.

Credit risk is a probable risk of loss resulting from customer's inability to meet contractual obligation that may have adverse impact on Group profitability. Group manages the risk by setting policies and limits for counterparty based on a pre-defined criteria linked with financial health of the customer. The exposure of each counterparty is monitored by Risk Management Function of the Group on daily basis.

Considering small Derivative portfolio, group is not exposed to any liquidity risk. However; Group manages its liquidity risk through Group liquidity risk framework which is defined in relevant Liquidity Risk Section.

Group have adequate system and controls for smooth execution of derivative transactions. Transactions are executed in line with well defined accounting and operational aspects to mitigate the operational risk. Policies and control functions are regularly reviewed on periodic basis. The Group uses a third party's Super Derivative System which provides front end sales and structuring capabilities, end to

Notes To The Consolidated Financial Statements
For the year ended December 31, 2020

end valuation solutions, risk management systems, back end processing and provides analytical tools to measure various risk exposures and carry out sensitivity analysis.

The Group uses a third party's Super Derivative System which provides front end sales and structuring capabilities, end to end valuation solutions, risk management systems, back end processing and provides analytical tools to measure various risk exposures and carry out sensitivity analysis.

The goal of asset/liability management (ALM) is to properly manage the risk related to changes in interest rates, the mix of balance sheet assets and liabilities, the holding of foreign currencies, and the use of derivatives. Due to thin liquidity in the derivative market, interest rate derivatives are not actively used to manage/alter the interest rate risk profile of the Group."

46 EVENTS AFTER THE REPORTING DATE

46.1 The Board of Directors in its meeting held on February 10, 2021 has announced a final cash dividend in respect of the year ended December 31, 2020 of Rs. 15.00 per share (2019: Rs. 5.00 per share). These consolidated financial statements for the year ended December 31, 2020 do not include the effect of these appropriations which will be accounted for subsequent to the year end.

47 GENERAL

Figures have been rounded off to the nearest thousand of rupees unless otherwise stated.

48 DATE OF AUTHORIZATION FOR ISSUE

These consolidated financial statements were authorized for issue by the Board of Directors of the Group in their meeting held on February 10, 2021.

Imran Maqbool	Hammad Khalid	S.M. Muneer	Mian Umer Mansha	Shahzad Hussain
President/Chief Executive	Chief Financial Officer	Director	Director	Director

Disposal of fixed assets

(refer note 12.2.6)

Description	Cost/ revalued amount	Accumu- lated depreciation	Book value	Sales pro- ceeds	Mode of disposal	Particulars of buyers	Location
		(Rupees '000)					
**Computers**							
Laptop	88	88	–	9	As per Bank's policy	Muhammad Ali	Lahore
Laptop	116	116	-	12	As per Bank's policy	Usman Hassan	Lahore
Laptop	334	100	244	258	As per Bank's policy	Nabeela Waheed	Lahore
Laptop	236	197	39	44	As per Bank's policy	Nadeem Afzal Khan	Lahore
	**784**	**501**	**283**	**323**			

Annexure I I

MCB Islamic Bank Limited (A Subsidiary of MCB Bank Limited)

Statement of Financial Position

AS AT DECEMBER 31, 2020

MCB Islamic bank (the 'Bank') is operating 187 Islamic banking branches in Pakistan (December 31, 2019: 185 branches).

	Note	2020	2019
		(Rupees in '000)	
**ASSETS**			
Cash and balances with treasury banks		9,872,197	10,252,547
Balances with other banks		4,996,602	8,822,985
Due from financial institutions	1	828,790	5,851,664
Investments - net	2	27,617,997	16,309,800
Islamic financing and related assets - net	3	84,896,191	51,309,967
Fixed assets		5,548,894	5,779,772
Intangible assets		639,428	719,723
Deferred tax assets - net		874,878	921,017
Other assets - net		5,895,909	5,049,786
		141,170,886	105,017,261
**LIABILITIES**			
Bills payable		2,470,821	973,627
Due to financial institutions		20,596,773	4,127,526
Deposits and other accounts	4	99,253,161	81,853,511
Liabilities against assets subject to finance lease		–	–
Sub-ordinated debts		–	–
Deferred tax liabilities - net		–	–
Other liabilities		8,122,359	7,595,796
		130,443,114	94,550,460
**NET ASSETS**		10,727,772	10,466,801
**REPRESENTED BY**			
Share capital		11,550,000	11,550,000
Reserves		68,107	26,444
Surplus on revaluation of assets - net of tax		473,316	422,326
Accumulated losses	6	(1,363,651)	(1,531,969)
		10,727,772	10,466,801
**CONTINGENCIES AND COMMITMENTS**	7		

MCB
Bank for Life

MCB Islamic Bank Limited (A Subsidiary of MCB Bank Limited)

Profit and Loss Account

For the year ended December 31, 2020

The profit and loss account of the bank's branches for the year ended 187 (December 31, 2019: 185 branches).

	Note	2020	2019
		(Rupees in '000)	
Profit / return earned	8	9,676,435	9,848,819
Profit / return expensed	9	5,280,989	5,855,061
Net spread earned		4,395,446	3,993,758
**OTHER INCOME**			
Fee and commission income		316,426	294,491
Dividend income		47,703	75,383
Foreign exchange income		209,888	63,685
(Loss) / gain on securities		14,274	(109,983)
Other income		89,862	19,743
Total other income		678,153	343,319
**TOTAL INCOME**		5,073,599	4,337,077
**OTHER EXPENSES**			
Operating expenses		4,654,830	4,478,636
Workers welfare fund		9,830	–
Other charges		590	24,386
Total other expenses		4,665,250	4,503,022
Profit / (loss) before provisions		408,349	(165,945)
Provisions and write offs - net		15,270	199,814
Extra ordinary / unusual items		–	–
**PROFIT / (LOSS) BEFORE TAXATION**		393,079	(365,759)
Taxation		184,763	(122,147)
**PROFIT / (LOSS) AFTER TAXATION**		208,316	(243,612)

Annexure I I

MCB Islamic Bank Limited (A Subsidiary of MCB Bank Limited)

Notes to the Financial Statements

For the year ended December 31, 2020

	Note	2020	2019
		(Rupees in '000)	
**1 DUE FROM FINANCIAL INSTITUTIONS**			
**Secured**			
Bai Muajjal receivable - with State Bank of Pakistan	1.1	828,790	3,371,664
**Unsecured**			
Musharaka arrangements		–	2,480,000
		828,790	5,851,664

1.1 This represents Bai Muajjal with State Bank of Pakistan carrying profit of 9.14% per annum (2019: average rate of 10.34%) and having maturity till January 29, 2021.

	2020	2019
	(Rupees in '000)	
1.3 Particulars of due from financial institutions - local currency	828,790	5,851,664

2 INVESTMENTS

2.1 Investments by segments:

	2020				2019			
	Cost/ amortised cost	Provision for diminution	Surplus / (deficit)	Carrying value	Cost/ amortised cost	Provision for diminution	Surplus / (deficit)	Carrying value
	(Rupees in '000)							
**Federal Government securities**								
GOP Ijarah Sukuks	22,475,798	–	(58,797)	22,417,001	10,842,666	–	(8,095)	10,834,571
WAPDA Sukuks	27,894	–	(781)	27,113	56,404	–	711	57,115
Bai Mu'ajjal	2,700,827	–	–	2,700,827	2,700,827	–	–	2,700,827
**Shares**			–					
Listed companies	1,815,977	1,039,436	292,987	1,069,528	1,908,146	1,059,172	175,625	1,024,599
**Non Government securities**			–					
Listed	549,000	–	4,119	553,119	854,000	–	1,309	855,309
Un listed	840,000	–	10,409	850,409	840,000	–	(2,621)	837,379
Total Investments	28,409,496	1,039,436	247,937	27,617,997	17,202,043	1,059,172	166,929	16,309,800

2.2 There were no investment given as colleteral as at December 31, 2020 (2019: Nil).

2.3 Provision for diminution in value of investments

	2020	2019
	(Rupees in '000)	
Opening balance	1,059,172	881,903
Charge for the year	36,975	177,269
Reversal on disposals	(56,711)	–
Closing balance	1,039,436	1,059,172

Notes to the Financial Statements

For the year ended December 31, 2020

	Note	2020	2019
		(Rupees in '000)	
**3 ISLAMIC FINANCING AND RELATED ASSETS - NET**			
Murabaha	3.1	12,055,820	7,609,830
Musawamah		–	2,000
Istisna		3,455,789	2,603,493
Salam		–	111,287
Ijarah	3.2	2,815,368	4,881,400
Running Musharaka		40,757,574	16,669,096
Diminishing Musharaka		24,693,571	18,176,482
Staff finance		1,184,311	1,293,082
Islamic financing and related assets - gross		84,962,433	51,346,670
Less: Provision against non-performing Islamic financing and related assets			
- Specific		(25,395)	(3,340)
- General		(40,847)	(33,363)
		(66,242)	(36,703)
**Islamic financing and related assets - net of provisions**		84,896,191	51,309,967
**3.1 Murabaha**			
- Murabaha financing	3.1.1	4,998,262	4,671,163
- Murabaha inventory		6,494,165	2,649,423
- Murabaha inventory - SBP's IRSPWS		27,939	–
- Advances against Murabaha financing		510,454	239,244
- Murabaha financing under Islamic export refinance scheme		5,000	–
- Advances against Murabaha financing under Islamic export refinance scheme		20,000	50,000
		12,055,820	7,609,830
**3.1.1 Murabaha receivable - gross**	3.1.1.1	5,232,085	5,005,449
Less: Deferred Murabaha income	3.1.1.3	233,823	334,286
Murabaha financing		4,998,262	4,671,163
**3.1.1.1 Movement in Murabaha receivable during the year**			
Opening balance		5,005,449	7,674,572
Sales during the year		20,865,558	25,845,273
Adjusted during the year		(20,638,922)	(28,514,396)
		5,232,085	5,005,449
**3.1.1.2 Murabaha sale price during the year**		20,865,558	25,845,273
Murabaha purchase price during the year		(19,838,369)	(24,447,190)
		1,027,189	1,398,083
**3.1.1.3 Deferred Murabaha income**			
Opening balance		334,286	340,551
Arising during the year		1,027,189	1,398,083
Recognised during the year		(1,127,652)	(1,404,348)
		233,823	334,286

MCB Islamic Bank Limited (A Subsidiary of MCB Bank Limited)
Notes to the Financial Statements
For the year ended December 31, 2020

		Note	2020	2019
			(Rupees in '000)	
3.2	**Ijarah financing and related assets**			
	- Net book value of assets in Ijarah under IFAS 2	3.2.1	2,783,440	4,190,369
	- Advances against Ijarah		31,928	691,031
			2,815,368	4,881,400

3.2.1 Net book value of assets in Ijarah under IFAS 2

2020

	As at January 1, 2020	Addition/ (disposal)	As at December 31, 2020	As at January 1, 2020	Charge (disposal)	As at December 31, 2020	Book Value as at December 31, 2020
	(Rupees in '000)						
Vehicles	3,999,646	250,956<br>(1,280,211)	2,970,391	1,039,780	603,062<br>(591,745)	1,051,097	1,919,294
Equipment and Plant and Machinery	1,689,096	798,286<br>(1,301,211)	1,186,171	458,593	349,730<br>(486,298)	322,025	864,146
Total	5,688,742	1,049,242<br>(2,581,422)	4,156,562	1,498,373	952,792<br>(1,078,043)	1,373,122	2,783,440

2019

	As at January 1, 2019	Addition/ (disposal)	As at December 31, 2019	As at January 1, 2019	Charge (disposal)	As at December 31, 2019	Book Value as at December 31, 2019
	(Rupees in '000)						
Vehicles	2,882,357	1,501,816<br>(384,527)	3,999,646	654,281	629,680<br>(244,181)	1,039,780	2,959,866
Equipment and Plant and Machinery	1,203,656	970,541<br>(485,101)	1,689,096	483,657	363,007<br>(388,071)	458,593	1,230,503
Total	4,086,013	2,472,357<br>(869,628)	5,688,742	1,137,938	992,687<br>(632,252)	1,498,373	4,190,369

3.2.2 Future Ijarah payments receivable

2020

	Not later than 1 year	Later than 1 year and not later than years	Over five years	Total
	(Rupees in '000)			
Ijarah rental receivables	1,100,022	1,782,989	3,456	2,886,467

2019

	Not later than 1 year	Later than 1 year and not later than years	Over five years	Total
	(Rupees in '000)			
Ijarah rental receivables	3,094,930	3,085,207	72,419	6,252,556

MCB Islamic Bank Limited (A Subsidiary of MCB Bank Limited)

Notes to the Financial Statements

For the year ended December 31, 2020

	2020	2019
	(Rupees in '000)	
**3.3 Particulars of Islamic financing and related assets - gross**		
In local currency	84,918,786	51,346,670
In foreign currency	43,647	–
	84,962,433	51,346,670

4 DEPOSITS AND OTHER ACCOUNTS

	2020			2019		
	In Local currency	In Foreign currency	Total	In Local currency	In Foreign currency	Total
	(Rupees in '000)					
**Customers**						
Current deposits	28,501,991	2,175,509	30,677,500	20,470,659	1,721,169	22,191,828
Savings deposits	39,831,137	1,971,071	41,802,208	31,630,709	2,085,896	33,716,605
Term deposits	17,861,765	79,103	17,940,868	15,049,977	72,099	15,122,076
Others	2,693,912	–	2,693,912	4,918,750	–	4,918,750
	88,888,805	4,225,683	93,114,488	72,070,095	3,879,164	75,949,259
Financial Institutions						
Current deposits	148,391	138	148,529	98,729	1,605	100,334
Savings deposits	2,462,010	1,634	2,463,644	1,986,733	1,554	1,988,287
Term deposits	3,526,500	–	3,526,500	3,815,631	–	3,815,631
	6,136,901	1,772	6,138,673	5,901,093	3,159	5,904,252
	95,025,706	4,227,455	99,253,161	77,971,188	3,882,323	81,853,511

	2020	2019
	(Rupees in '000)	
**4.1** Composition of deposits		
Individuals	43,739,410	35,007,567
Government (Federal and Provincial)	4,253,371	7,692,691
Public Sector Entities	4,861,494	268,859
Banking Companies	2,075	1,037,961
Non-Banking Financial Institutions	6,136,598	4,866,288
Private Sector	40,260,213	32,980,145
	99,253,161	81,853,511

4.2 This includes deposits eligible to be covered under takaful arrangements amounting to Rs.61,239.189 million (2019: 51,165.607 million).

		2020	2019
		(Rupees in '000)	
**5 Charity balance**			
Opening balance		57,782	25,402
Additions during the year			
- Received from customers against late payment		42,477	45,958
- Dividend purification amount		771	7,383
- Profit on charity saving account		2,085	1,539
		45,333	54,880
Charity paid during the year	5.1	(56,500)	(22,500)
Closing balance		46,615	57,782

MCB Islamic Bank Limited (A Subsidiary of MCB Bank Limited)
Notes to the Financial Statements
For the year ended December 31, 2020

		2020	2019
		(Rupees in '000)	
5.1	**Charity was paid to the following institutions:**		
	The Patients' Bahbood Society for AKUH	1,000	1,000
	Al-Khidmat Foundation	2,000	–
	Arthritis Care	2,000	1,000
	Aziz Jehan Begum Trust for the Blind	3,000	1,000
	Chiniot Anjuman Islamia	1,000	–
	Chiniot Blood Bank and Dialysis Centre	1,000	–
	Family Welfare Society	3,000	1,000
	Fatmid Foundation	3,000	1,000
	Indus Hospital	5,000	2,000
	Infaq Memorial Trust	3,000	1,000
	Layton Rehmatullah Benevolent Trust	4,000	2,000
	Mind Organization	2,000	1,000
	Pink Ribbon	2,000	5,000
	Saleem Memorial Trust Hospital	6,000	–
	Saylani Welfare	2,000	–
	Shaukat Khanam Memorial Cancer Hospital	3,000	2,000
	Sindh Institute of Urology & Transplantation (SIUT)	3,000	2,000
	The Citizens Foundation	4,000	2,000
	Jahandad Society for Community Development	1,500	–
	The Lahore Hospital Welfare Society	2,000	500
	Al - Mustafa Welfare Society	500	–
	Frontier Foundation blood transfusion Centre	500	–
	Zubaida Medical Center	1,000	–
	Mofad e Amma Chiniot Sheikh Association	1,000	–
		56,500	22,500

5.2 Charity was not paid to any staff of the Bank or to any individual / organisation in which a director or his spouse had any interest at any time during the year.

		Note	2020	2019
			(Rupees in '000)	
6	**ISLAMIC BANKING BUSINESS ACCUMULATED LOSS**			
	Opening balance		(1,531,969)	(1,289,202)
	Islamic banking profit / loss for the year		393,079	(365,759)
	Transfer to statutory reserve		(41,663)	–
	Taxation		(184,763)	122,147
	Other adjustments		1,665	845
	Closing balance		(1,363,651)	(1,531,969)
7	**CONTINGENCIES AND COMMITMENTS**			
	Guarantees	7.1	8,000,674	6,467,046
	Commitments	7.2	20,583,025	11,498,973
	Other contingent liabilities		437,433	431,439
			29,021,132	18,397,458
7.1	**Guarantees:**			
	Performance guarantees		3,935,124	3,431,726
	Other guarantees		4,065,550	3,035,320
			8,000,674	6,467,046

MCB Islamic Bank Limited (A Subsidiary of MCB Bank Limited)
Notes to the Financial Statements
For the year ended December 31, 2020

			2020	2019
			(Rupees in '000)	
7.2	**Commitments:**			
	Documentary credits and short-term trade-related transactions			
	Letters of credit		7,654,971	7,818,321
	Commitments in respect of:			
	Forward foreign exchange contracts	7.2.1	11,909,879	3,363,957
	Commitments for acquisition of:			
	Fixed assets		–	–
	Intangible assets		10,724	8,940
	Other commitments	7.2.2	1,007,451	307,755
			20,583,025	11,498,973
7.2.1	**Commitments in respect of forward foreign exchange contracts**			
	Purchase		5,046,837	882,210
	Sale		6,863,042	2,481,747
			11,909,879	3,363,957
7.2.2	**Other Commitments**			
	Commitments to extend credit	7.2.2.1	1,007,451	307,755

7.2.2.1 Other than those stated above, the Bank makes commitment(s) to extend credit in the normal course of business including related parties but these being revocable commitments do not attract any penalty or expense if the facility is unilaterally withdrawn.

		2020	2019
		(Rupees in '000)	
7.3	**Other contingent liabilities**		
	Claim against the Bank not acknowledged as debt	437,433	431,439

This includes claim by a third party against the Bank, amounting to Rs. 425.820 million which is being contested in the Court of law. The suit has been disposed off by the Court vide Order dated May 10, 2019 wherein the status quo has been ordered to be maintained with respect to bank guarantee and the matter has been referred to arbitration with the consent of the parties. However the Bank has not received any official notice to attend the arbitration proceedings till date.

In addition to the above, this includes claim by different parties against the bank amounting to Rs. 11.6 million which is pending before the court.

The Sindh Revenue Board (SRB) has issued order under "Sindh Sales Tax on Services Act, 2011", for the year 2016 thereby raising demand of Rs. 0.642 million. The Bank has filed appeal before Commissioner of Inland Revenue Appeals which is pending adjudication. The management of the Bank, in consultation with its tax advisor, is confident that the decision in respect of the above matter would be in the Bank's favor and accordingly no provision has been made in these financial statements with respect thereto.

MCB Islamic Bank Limited (A Subsidiary of MCB Bank Limited)
Notes to the Financial Statements
For the year ended December 31, 2020

		2020	2019
		(Rupees in '000)	
**8**	**PROFIT / RETURN EARNED**		
	Financings	7,044,066	7,288,776
	Investments in		
	- available for sale securities	1,853,889	1,411,649
	- held to maturity securities	411,594	400,202
		2,265,483	1,811,851
	Musharaka arrangements with financial institutions	215,060	555,462
	Deposits with financial institutions	151,826	192,730
		9,676,435	9,848,819
**9**	**PROFIT / RETURN EXPENSED**		
	Deposits and other accounts	4,340,687	4,936,521
	Musharaka arrangements with the State Bank of Pakistan under IERS	66,345	45,860
	Musharaka arrangements with other financial institutions	468,267	490,506
	Musharaka arrangements with other institution	16,900	7,973
	Unwinding of liability against right-of-use of asset	388,790	374,201
		5,280,989	5,855,061

10 PROFIT / (LOSS) DISTRIBUTION TO DEPOSITORS' AND SPECIFIC POOLS

10.1 The Bank is maintaining the following types of pools for profit declaration and distribution:

1) General Pool

2) IERS Musharaka Pool

3) Treasury Musharaka / Mudaraba Pools

4) Special Musharaka Pool

5) Equity Pool

Features, risks and rewards of each pool are given below:

1) General Pool

The Bank manages one general pool for its depositors' (Rabbul Mal) maintaining deposits in both local and foreign currencies and also commingled its equity in this pool. The income (gross income less direct expenses) generated from the pool is distributed between Bank's equity and depositors' fund in proportion to their respective share in the pool. Under the Mudaraba mechanism, the income so distributed to depositors' fund is shared between the Bank (Mudarib) and depositors' (Rabbul Mal) according to the pre-agreed profit sharing ratios and assigned weightages.

The deposits and funds accepted under the General Pool are deployed to diversified sectors and avenues of the economy / business mainly to 'Agriculture, Forestry & Fishing', 'Textile & Allied', 'Food & Allied', 'Distribution & Trade', 'Investment in Government of Pakistan Ijarah Sukuk', etc.

Parameters associated with risk and rewards

Following are the consideration attached with risk and reward of general pool:

- Period, return, safety, security and liquidity of investment.
- Financing proposals under process at various stages and likely to be extended in the near future.
- Expected amount of procurement of deposit during coming days as a result of concerted marketing efforts of the Bank.
- Element of risk attached to various types of investments.
- SBP rules and Shari'ah clearance.

2) Islamic Export Refinance Scheme (IERS) Musharaka Pool

The Bank manages IERS Musharaka Pool for funds accepted from SBP under IERS. Under the PLS mechanism, the Bank generates revenues from the pool funds which are shared with the SBP according to the pre-agreed profit sharing ratios. Musharaka investments from the SBP under IERS are channeled towards the export sector of the economy and other financings as per SBP guidelines.

3) Treasury Musharaka / Mudaraba Pools

The Bank accepts funds from other banks to manage its liquidity under Musharaka / Mudaraba mode. The funds accepted are tagged to remunerative assets having maturity on or after the period for which funds are accepted. The revenue generated from the pool asset is shared between the bank and other member of the pool according to pre-agreed profit sharing ratios or assigned weightages accordingly.

4) Special Musharaka Pool

The Bank under Musharaka agreement accepts funds from institution(s) (other than banks). The comingled funds under this arrangement are deployed in remunerative assets as per the terms of agreement. The revenue generated from these assets are then shared as per pre-agreed profit sharing ratio.

5) Equity Pool

The Equity Pool consists of Bank's equity and funds accepted on Qard (non-remunerative current deposit account) basis. The funds of this pool are invested in various assets or ventures which are higher in risk or having longer funding period. In addition to that all staff financings are financed by this pool. The risk of assets in the pool is borne by the Bank.

Charging of expenses

Direct expenses are being charged to respective pools, while indirect expenses such as general and administrative expenses are being borne by the Bank as Mudarib. No provision expense is charged to the pool unless it is written off. The direct expenses charged to the pool may include depreciation of Ijarah assets, premium amortization on Sukuk, impairment losses due to physical damages to specific assets in pools etc. However, this is not an exhaustive list; the Bank's pool management framework and the respective pool creation memo may identify and specify these and any other similar expenses to be charged to the pool.

Annexure I I

MCB Islamic Bank Limited (A Subsidiary of MCB Bank Limited)
Notes to the Financial Statements
For the year ended December 31, 2020

10.2 Following are the detail of profit distribution among different pool maintained by the Bank:

2020

Pool Description	Profit Rate & weightage announcement period	Profit Rate Earned	Profit Sharing Ratio of Mudarib	Mudarib Fee	Profit Rate return distributed	General Hiba	Amount of General Hiba
		%	%	Rupees in thousands	%	%	Rupees in thousands
General Pool	Monthly	10.84	50.00	3,558,858	6.53	20.30	722,410

Pool Description	Profit Rate & weightage announcement period	Profit Rate Earned	Profit Sharing Ratio of Mudarib	Mudarib Fee	Profit Rate return distributed	General Hiba	Amount of General Hiba
		%	%	Rupees in thousands	%	%	Rupees in thousands
Islamic Export Refinance (IERS) Pool	Monthly	6.90	–	–	2.00	–	–
Treasury Musharaka/ Mudaraba Pool	As required	9.60	–	–	7.25	–	–
Special Musharakh Pool	Monthly	10.48	–	–	9.21	–	–
Equity Pool	Monthly	5.33	–	–	–	–	–

2019

Pool Description	Profit Rate & weightage announcement period	Profit Rate Earned	Profit Sharing Ratio of Mudarib	Mudarib Fee	Profit Rate return distributed	General Hiba	Amount of General Hiba
		%	%	Rupees in thousands	%	%	Rupees in thousands
General Pool	Monthly	13.36	50.00	3,710,740	8.82	31.84	1,181,467

Pool Description	Profit Rate & weightage announcement period	Profit Rate Earned	Profit Sharing Ratio of Mudarib	Mudarib Fee	Profit Rate return distributed	General Hiba	Amount of General Hiba
		%	%	Rupees in thousands	%	%	Rupees in thousands
Islamic Export Refinance (IERS) Pool	Monthly	7.92	–	–	2.00	–	–
Treasury Musharaka/ Mudaraba Pool	As required	11.51	–	–	9.88	–	–
Special Musharakh Pool	Monthly	8.69	–	–	8.08	–	–
Equity Pool	Monthly	1.58	–	–	–	–	–

Branch Network 2020

As of December 31, 2020

Retail Banking Group - Central			
**Circle/ No. of Branches**	**Region**	**No. of Branches**	**No. of Sub-Branches**
GUJRANWALA - 122	Gujranwala	32	–
	Gujrat	27	–
	Mandi Bahauddin	28	–
	Sialkot	34	–
LAHORE EAST - 72	Lahore Defence	25	–
	Lahore Gulberg	25	–
	Lahore Johar Town	22	–
LAHORE WEST - 92	Lahore City	32	–
	Lahore The Mall	32	–
	Shiekhupura	27	–
**Total RBG - Central**		**286**	**–**

Retail Banking Group - North			
**Circle/ No. of Branches**	**Region**	**No. of Branches**	**No. of Sub-Branches**
ABBOTTABAD - 93	Abbottabad	27	–
	Attock	20	–
	Muzaffarabad A.K.	22	–
	Swat	24	1
ISLAMABAD - 93	Fateh Jang	22	–
	Islamabad	26	–
	Rawalpindi Cantt	24	–
	Rawalpindi City	21	–
PESHAWAR - 98	Kohat	27	–
	Mardan	23	–
	Peshawar East	22	1
	Peshawar West	26	–
**Total RBG - North**		**284**	**02**

Retail Banking Group - West			
**Circle/ No. of Branches**	**Region**	**No. of Branches**	**No. of Sub-Branches**
FAISALABAD - 87	Faisalabad	30	–
	Faisalabad City	28	1
	Toba Tek Singh	29	–
JHELUM - 78	Chakwal	26	–
	Jhelum	26	–
	Mirpur A.K.	26	–
SARGODHA - 94	Jhang	28	2
	Mianwali	31	–
	Sargodha	35	1
**Total RBG - West**		**259**	**04**

Branch Network 2020

As of December 31, 2020

Retail Banking Group - East			
**Circle/ No. of Branches**	**Region**	**No. of Branches**	**No. of Sub-Branches**
BAHAWALPUR - 104	Bahawalnagar	18	–
	Bahawalpur	28	–
	Rahim Yar Khan	31	–
	Vehari	27	–
MULTAN - 114	Dera Ghazi Khan	20	–
	Multan	30	1
	Muzaffargarh	20	–
	Okara	21	1
	Sahiwal	23	–
**Total RBG - East**		**218**	**02**
**Retail Banking Group - Karachi**			
**Circle/ No. of Branches**	**Region**	**No. of Branches**	**No. of Sub-Branches**
KARACHI CITY - 58	Karachi City	29	–
	Karachi North	29	–
KARACHI EAST - 46	Karachi East	24	–
	Karachi South	22	–
KARACHI WEST - 46	Karachi Central	22	–
	Karachi West	24	–
**Total RBG - Karachi**		**150**	**–**
**Retail Banking Group - South**			
**Circle/ No. of Branches**	**Region**	**No. of Branches**	**No. of Sub-Branches**
HYDERABAD - 77	Hyderabad	28	–
	Mirpurkhas	25	1
	Nawabshah	24	–
QUETTA - 50	Khuzdar	14	–
	Makran	10	–
	Quetta	26	3
SUKKUR - 75	Larkana	24	–
	Naushero Feroze	24	–
	Sukkur	27	–
**Total RBG - South**		**202**	**4**
**Wholesale Banking Group**			
WBG Branches		11*	–
*EPZ not included			
**Consumer Banking Group**			
Privilege Banking		8	–

Branch Network 2020

As of December 31, 2020

Overseas Branches / International Banking	No. of Branches
Colombo	1
Galle	1
Kandy	1
Kattankudy	1
Kollupitiya	1
Maradana	1
Pettah	1
Wellawatte	1
MCB Offshore Banking Unit (OBU) Bahrain	1
MCB Dubai Wholesale Branch (UAE)	1
EPZ	1
**TOTAL**	**11**

Groupwise			
**Group**	**Circles**	**No. of Branches**	**No. of Sub-Branches**
RBG–Central	3	286	–
RBG–North	3	284	2
RBG–West	3	259	4
RBG–East	2	218	2
RBG–Karachi	3	150	–
RBG–South	3	202	4
WBG	3	11	–
CBG - Privilege Banking	–	8	–
**Total**	**20**	**1,418**	**12**
Overseas	–	10	–
EPZ	–	1	–
**Grand Total**	**20**	**1,429**	**12**

Province-Wise			
**Provinces/Territories/AJK**	**Branches**	**Sub-Branches**	**Total**
Azad Jammu & Kashmir	40	–	40
Balochistan	52	3	55
Federal Capital Territory	34	–	34
Gilgit–Baltistan	6	–	6
Khyber Pakhtunkhwa	154	2	156
Punjab	827	6	833
Sindh	305	1	306
**Domestic Total**	**1,418**	**12**	**1,430**
Overseas Branches / International Banking	10	–	10
EPZ	1	–	1
**Grand Total**	**1,429**	**12**	**1,441**

Pattern of Shareholding

As of December 31, 2020

No. of Shareholders	Shareholdings		Total Shares Held
	From	To	
27,680	1	100	885,188
13,568	101	500	3,316,749
5,713	501	1,000	4,245,735
7,589	1,001	5,000	13,183,013
449	5,001	10,000	3,268,384
477	10,001	50,000	10,612,463
138	50,001	100,000	9,763,934
171	100,001	500,000	41,249,898
49	500,001	1,000,000	35,286,220
64	1,000,001	5,000,000	129,262,103
8	5,000,001	10,000,000	51,302,800
4	10,000,001	15,000,000	48,532,209
3	15,000,001	25,000,000	54,066,041
12	25,000,001	Above	780,085,269
**55,925**			**1,185,060,006**

Categories of Shareholders

As of December 31, 2020

Categories of Shareholders	Shares Held	Percentage
Directors, Chief Executive Officer, their Spouses and Minor Children	119,654,330	10.0969%
Associated Companies, Undertakings and Related Parties	218,402,532	18.4297%
NIT and ICP	912	0.0001%
Banks, Development Financial Institutions, Non Banking Financial Institutions	20,267,864	1.7103%
Insurance Companies	106,815,039	9.0135%
Modarabas and Mutual Funds	11,629,076	0.9813%
Shareholders Holding 10%	222,606,147	18.7844%
General Public Local	137,247,548	11.5815%
General Public Foreign	4,874,244	0.4113%
Others	343,562,314	28.9911%
**Grand Total**	**1,185,060,006**	**100.0000%**

Categories of Shareholders

As of December 31, 2020

Categories of Shareholders	Shares Held	Percentage
**Directors, Chief Executive Officer, their Spouses and Minor Children**		
Mian Mohammad Mansha	7,834	0.0007%
Naz Mansha	6,424,057	0.5421%
S. M. Muneer	2,059	0.0002%
Muhammad Tariq Rafi	32,094,304	2.7082%
Mrs. Nighat Tariq	5,715,093	0.4823%
Mian Umer Mansha	31,986,378	2.6991%
Iqraa Hassan Mansha	8,000	0.0007%
Mian Hassan Mansha	43,363,671	3.6592%
Muhammad Ali Zeb	550	0.0000%
Mohd Suhail Amar Suresh bin Abdullah	884	0.0001%
Yahya Saleem	500	0.0000%
Salman Khalid Butt	49,000	0.0041%
Shahzad Hussain	500	0.0000%
Masood Ahmed Puri	1,000	0.0001%
Shariffuddin Bin Khalid	500	0.0000%
	**119,654,330**	**10.0969%**
**Associated Companies, Undertakings and Related Parties**		
Nishat Mills Limited	88,015,291	7.4271%
Adamjee Insurance Company Limited	47,827,287	4.0359%
Siddiqsons Limited	14,276,462	1.2047%
Adamjee Life Assurance Company Ltd. - IMF	5,740,538	0.4844%
Adamjee Life Assurance Company Limited - NUIL Fund	446,311	0.0377%
Adamjee Life Assurance Co. Ltd. - DGF	63,500	0.0054%
Nishat (Aziz Avenue) Hotels and Properties Limited	141,950	0.0120%
Nishat Real Estates Development Company (Private) Limited	54,500	0.0046%
Trustee - MCB Provident Fund Pak Staff	34,166,060	2.8831%
Trustee - MCB Employees Pension Fund	17,793,759	1.5015%
Trustee Nishat (Chunian) Limited Employees Provident Fund	8,857	0.0007%
Nishat Mills Limited Employees Provident Fund Trust	8,284,390	0.6991%
Trustees of Adamjee Insurance Company Ltd. Employees Provident Fund	66,138	0.0056%
CDC - Trustee MCB Pakistan Asset Allocation Fund	196,586	0.0166%
CDC - Trustee Pakistan Capital Market Fund	65,038	0.0055%
CDC - Trustee MCB Pakistan Stock Market Fund	1,255,865	0.1060%
	**218,402,532**	**18.4297%**
**NIT and ICP**		
Investment Corporation of Pakistan	912	0.0001%
	**912**	**0.0001%**
**Banks, Development Financial Institutions, Non Banking Financial Institutions**		
The Bank of Punjab	614,379	0.0518%
Prudential Investment Bank Ltd.	1,393	0.0001%
Crescent Investment Bank Ltd.	590	0.0000%
Saudi Pak Industrial & Agricultural Investment Co. (Pvt) Ltd	607,992	0.0513%
Trust Leasing Corporation Ltd.	101	0.0000%
Universal Leasing Corporation Ltd.	1	0.0000%
Islamic Investment Bank Ltd.	4	0.0000%
National Development Finance Corporation	433	0.0000%
M/s. Al Faysal Investment Bank Ltd.	49	0.0000%
Interasia Leasing Company Limited	950	0.0001%
Pakistan Kuwait Investment Co. (Pvt) Ltd.	2,400,000	0.2025%
Allied Bank Limited	2,821,600	0.2381%
Habib Bank Limited	1,603,481	0.1353%
Faysal Bank Limited	2,855,170	0.2409%
Habib Metropolitan Bank Limited	491,900	0.0415%
Bank Al Habib Limited	700,000	0.0591%
Soneri Bank Limited	1,500,000	0.1266%
Saudi Pak Leasing Company Limited	495	0.0000%
Bank Alfalah Limited	2,100,000	0.1772%

The Punjab Provincial Cooperative Bank	1,558	0.0001%
Escorts Investment Bank Limited	225	0.0000%
National Bank of Pakistan	2,399,436	0.2025%
Askari Bank Limited	1,051,600	0.0887%
House Building Finance Company Limited	41,951	0.0035%
Pair Investment Company Limited	174,570	0.0147%
Sindh Bank Limited	870,530	0.0735%
Samba Bank Limited - MT	29,456	0.0025%
	**20,267,864**	**1.7103%**
**Insurance Companies**		
National General Insurance Co. Ltd.	1,359	0.0001%
Business & Industrial Insurance Co. Ltd.	9	0.0000%
M/s. New Jubilee Insurance Co. Ltd.	2	0.0000%
The South British Insurance Company Ltd.	1,864	0.0002%
M/s. Beema Pakistan Company Ltd.	69	0.0000%
E.F.U. General Insurance Ltd.	713	0.0001%
Orient Insurance Co. Ltd.	3	0.0000%
Premier Insurance Limited	125,204	0.0106%
Jubilee General Insurance Company Limited	1,042,654	0.0880%
State Life Insurance Corp. of Pakistan	18,059,087	1.5239%
EFU Life Assurance Ltd.	2,384,197	0.2012%
Pakistan Reinsurance Company Limited	500,000	0.0422%
Excel Insurance Co. Ltd.	52,500	0.0044%
The Crescent Star Insurance Co. Ltd.	3	0.0000%
Jubilee Life Insurance Company Limited	25,130,511	2.1206%
East West Insurance Co. Ltd.	25,000	0.0021%
Century Insurance Company Ltd.	32,235	0.0027%
Atlas Insurance Limited	140,000	0.0118%
Security General Insurance Co. Ltd.	59,136,076	4.9901%
The Pakistan General Insurance Co. Limited	106	0.0000%
IGI Life Insurance Limited	4,700	0.0004%
Alfalah Insurance Company Limited	175,000	0.0148%
Askari General Insurance Company	3,747	0.0003%
	**106,815,039**	**9.0135%**
**Modarabas and Mutual Funds**		
First Hajveri Modaraba	60	0.0000%
Crescent Modaraba Managment Co. Ltd	17	0.0000%
First Elite Capital Modaraba	39	0.0000%
First Crescent Modaraba	4,030	0.0003%
Trust Modaraba	313	0.0000%
UNICAP Modaraba	15	0.0000%
First Interfund Modaraba	2	0.0000%
Industrial Capital Modaraba	4	0.0000%
Safeway Fund (Pvt) Ltd	1,665	0.0001%
Pak Asian Fund Limited	162	0.0000%
Safeway Mutual Fund Ltd	16	0.0000%
Golden Arrow Selected Stocks Fund Ltd	15	0.0000%
Prudential Stocks Funds Limited	233	0.0000%
M/s. Asian Stock Fund Ltd	4	0.0000%
PICIC Benovelent Fund-2	29	0.0000%
MCBFSL - Trustee JS Value Fund	66,000	0.0056%
CDC - Trustee PICIC Investment Fund	182,253	0.0154%
CDC - Trustee JS Large CAP. Fund	51,350	0.0043%
CDC - Trustee PICIC Growth Fund	235,004	0.0198%
CDC - Trustee Atlas Stock Market Fund	1,443,415	0.1218%
CDC - Trustee Alfalah GHP Value Fund	118,223	0.0100%
CDC - Trustee Unit Trust of Pakistan	126,600	0.0107%
CDC - Trustee AKD Index Tracker Fund	98,678	0.0083%
Tri-Star Mutual Fund Limited	754	0.0001%
Crescent Standard Business Management (Pvt) Limited	1	0.0000%
CDC - Trustee NBP Stock Fund	1,240,422	0.1047%

CDC - Trustee NBP Balanced Fund	92,500	0.0078%
CDC - Trustee Askari Asset Allocation Fund	12,500	0.0011%
CDC - Trustee APF-Equity Sub Fund	75,000	0.0063%
CDC - Trustee JS Pension Savings Fund - Equity Account	20,500	0.0017%
CDC - Trustee HBL - Stock Fund	290,420	0.0245%
MC FSL - Trustee JS Growth Fund	455,800	0.0385%
CDC - Trustee HBL Multi - Asset Fund	23,900	0.0020%
CDC - Trustee Alfalah GHP Stock Fund	442,234	0.0373%
CDC - Trustee Alfalah GHP Alpha Fund	203,604	0.0172%
CDC - Trustee NIT-Equity Market Opportunity Fund	1,158,176	0.0977%
CDC - Trustee ABL Stock Fund	1,116,832	0.0942%
CDC - Trustee First Habib Stock Fund	17,970	0.0015%
CDC - Trustee Lakson Equity Fund	1,190,221	0.1004%
CDC - Trustee NBP Sarmaya Izafa Fund	92,800	0.0078%
CDC - Trustee NBP Mahana Amdani Fund - MT	144,411	0.0122%
CDC - Trustee HBL PF Equity Sub Fund	44,600	0.0038%
CDC - Trustee First Capital Mutual Fund	30,000	0.0025%
CDC - Trustee UBL Retirement Savings Fund - Equity Sub Fund	213,019	0.0180%
CDC - Trustee National Investment (Unit) Trust	870,735	0.0735%
CDC - Trustee ABL Pension Fund - Equity Sub Fund	28,219	0.0024%
CDC - Trustee NBP Income Opportunity Fund - MT	12	0.0000%
CDC - Trustee NITPF Equity Sub-Fund	26,000	0.0022%
CDC - Trustee NBP Savings Fund - MT	15,262	0.0013%
CDC - Trustee Faysal MTS Fund - MT	2,805	0.0002%
CDC - Trustee Lakson Tactical Fund	136,125	0.0115%
CDC - Trustee NBP Financial Sector Fund	233,700	0.0197%
CDC - Trustee UBL Financial Sector Fund	868,817	0.0733%
CDC - Trustee Allied Finergy Fund	103,939	0.0088%
CDC - Trustee NIT Asset Allocation Fund	60,000	0.0051%
CDC - Trustee NIT Pakistan Gateway Exchange Traded Fund	23,256	0.0020%
CDC - Trustee UBL Pakistan Enterprise Exchange Traded Fund	29,240	0.0025%
CDC - Trustee NBP Pakistan Growth Exchange Traded Fund	18,836	0.0016%
CDC - Trustee NBP Financial Sector Income Fund - MT	18,339	0.0015%
	**11,629,076**	**0.9813%**
**Share Holders Holding 10%**		
Maybank International Trust (Labuan) Berhad	222,606,147	18.7844%
	**222,606,147**	**18.7844%**
**General Public**	**142,121,792**	**11.9928%**
- Local	137,247,548	11.5815%
- Foreign	4,874,244	0.4113%
**Others**	**343,562,314**	**28.9911%**
- Foreign Companies	131,171,748	11.0688%
- Local Companies	45,092,084	3.8050%
- D. G. Khan Cement Company Limited	102,277,232	8.6306%
- Nishat Mills Limited *(as disclosed in Associated Companies, Undertakings and Related Parties)*	–	–
- Bugis Investments (Mauritius) PTE Ltd.	65,020,947	5.4867%
- Executives *(as per the threshold determined by Board of Directors)*	303	0.0000%
**Total**	**1,185,060,006**	**100.0000%**

All the trades in shares carried out by Directors, CEO, CFO, Secretary, Executives, their Spouses and Minor Children during the year 2020 are given below:

Name	Status	No. of Shares	Purchase/Sale/Transfer
Mr. Shahzad Hussain	Director	500	Purchased
Mr. Shariffuddin Bin Khalid	Director	500	Purchased
Mr. Salman Khalid Butt	Director	48,000	Purchased

Notice of 73rd Annual General Meeting

Notice is hereby given that 73rd Annual General Meeting of **MCB Bank Limited** (the "Bank" or "MCB") will be held on **Saturday, March 27, 2021 at 11:00 AM (PST)** at Grand Ball Room-D, The Nishat Hotel, Emporium Mall, Near Expo Center, Abdul Haq Road, Johar Town, Lahore with Video Link facility to transact the following business:

Ordinary Business:

1. To confirm/approve the Minutes of Annual General Meeting held on March 19, 2020.

2. To receive, consider and adopt the Annual Audited Separate and Consolidated Financial Statements of the Bank together with the Directors' Report and Auditors' Report thereon and the Chairman's Review Report for the year ended December 31, 2020.

3. To appoint Auditors of the Bank and fix their remuneration. The Members are hereby notified that the Board's Audit Committee and the Board have recommended the name of M/s A.F. Ferguson & Co., Chartered Accountants, as auditors of the Bank in place of retiring auditors M/s KPMG Taseer Hadi & Co., Chartered Accountants.

4. To approve, as recommended by the Board of Directors, payment of Final Cash Dividend @ 150% i.e., PKR 15.00 per share having face value of PKR 10/- in addition to 50% i.e., PKR 5.00 per share Interim Cash Dividend already declared and paid, thus total 200% i.e., PKR 20.00 per share for the year ended December 31, 2020.

5. To elect twelve (12) Directors of the Bank as fixed by its Board of Directors under Section 159(1) of the Companies Act, 2017 for a period of three (3) years commencing from March 27, 2021. The following are names of retiring directors who are eligible for re-election:
   1. Mian Mohammad Mansha.
   2. Mr. S. M. Muneer.
   3. Mr. Muhammad Tariq Rafi.
   4. Mian Umer Mansha.
   5. Mrs. Iqraa Hassan Mansha.
   6. Mr. Muhammad Ali Zeb.
   7. Mr. Mohd Suhail Amar Suresh bin Abdullah.
   8. Mr. Yahya Saleem.
   9. Mr. Salman Khalid Butt.
   10. Mr. Masood Ahmed Puri.
   11. Mr. Shahzad Hussain.
   12. Mr. Shariffuddin Bin Khalid.

Special Business:

6. To consider and, if deemed fit, to pass an Ordinary Resolution, with or without amendments, in terms of Section 183(3)(b) of the Companies Act, 2017 to approve the voluntary winding up of M/s Financial & Management Services (Pvt.) Limited, a subsidiary of the Bank.

By Order of the Board,

-Sd-

FIDA ALI MIRZA
Company Secretary

March 05, 2021
Lahore.

Statement of Material Facts under Section 134(3) of the Companies Act, 2017 ("Act") pertaining to the above mentioned Special Business and the Statement under Section 166(3) of the Act are annexed to this Notice of 73rd Annual General Meeting circulated to the Members of the Bank.

Notes:

1. Minutes of the Annual General Meeting held on March 19, 2020 of MCB Bank Limited (the "Bank" or "MCB") were available for inspection by Members as required under Section 152 of the Companies Act, 2017 (the "Act").

2. The Shares Transfer Books of the Bank will remain closed from **March 16, 2021 to March 27, 2021** (both days inclusive). Transfers received at the Bank's Share Registrar and Transfer Agent's Office at the below mentioned address, at the close of business hours on **March 15, 2021** will be treated as being in time for the purpose of entitlement of Final Cash Dividend and also to attend, speak and vote at the 73rd Annual General Meeting (the "Meeting" or "AGM") of the Bank.

3. All Members are entitled to attend and vote at the Meeting. A member entitled to attend and vote at AGM may appoint another member as a proxy to attend and vote on his/her behalf. No person shall act as a proxy, who is not a member of the Bank. A corporate entity, being a member, may authorize through resolution of its board or other governing body, an individual to act as its representative and the individual so authorized shall be entitled to exercise the same powers on behalf of the corporate entity which he represents.

4. The proxies and in case of corporate entity, the power of attorney or resolution of the board of directors or other governing body (if any) under which it is signed, a notarized/certified copy of the same in order to be effective must be deposited at the Share Registrar and Transfer Agent Office of the Bank not less than 48 hours (no account shall be taken of any part of the day that is not a working day) before the time for holding the Meeting, and must be duly stamped, signed and witnessed.

5. If a Member appoints more than one proxy, and more than one instrument of proxy is deposited by a Member, all such instruments of proxy shall be rendered invalid.

6. Members having physical scrip of shares are requested to immediately notify the change, if any, in their registered and/or e-mail addresses, in writing, to the Share Registrar and Transfer Agent of the Bank, whereas, CDC Account holders are requested to contact their CDC Participant/CDC Account Services.

7. Central Depository Company of Pakistan ("CDC") Accountholders will further have to follow the under mentioned guidelines as laid down by Circular No. 01, dated January 26, 2000, issued by the Securities and Exchange Commission of Pakistan ("SECP"):

   For Attending the Meeting:

   i. In case of individuals, the account-holder or sub-accountholder and/or the person whose securities are in group account and their registration details are uploaded as per the Regulations, shall authenticate his identity by showing his original Computerized National Identity Card ("CNIC") or original passport at the time of attending the Meeting.

   ii. In case of corporate entity, the Board of Directors' resolution/power of attorney with specimen signature of the nominee shall be produced (unless it has been provided earlier) at the time of the Meeting.

   For Appointing of Proxies:

   i. In case of individuals, the account-holder or sub-accountholder and/or the person whose securities are in group account and their registration details are uploaded as per the CDC Regulations, shall submit the proxy form as per the above requirement.

   ii. The proxy form shall be witnessed by the two persons whose names, addresses and CNIC numbers shall be mentioned on the form.

   iii. Attested copy of CNIC or the passport of the beneficial owners and the proxy shall be furnished with the proxy form.

   iv. The proxy shall produce his/her original CNIC or passport at the time of the meeting.

   v. In case of corporate entity, the Board of Directors resolution/power of attorney with specimen signature shall be submitted along with proxy form of the Bank.

8. A Proxy Form, both in English and Urdu language, is being separately sent to the Members, along with Notice of AGM.

9. Copies of the Notice of AGM, the latest annual audited/quarterly financial statements and the Memorandum & Articles of Association of the Bank have been kept at the Registered Office of the Bank which can be obtained and/or inspected during the business hours on any working day from the date of publication of this Notice of AGM till the conclusion of the Meeting by the Shareholders and other persons entitled to attend and vote at the Meeting. Notice of AGM and the latest annual audited/quarterly financial statements have also been placed on website of the Bank, namely, www.mcb.com.pk.

10. The existing term of office of the Board of Directors of the Bank will expire on March 27, 2021. The Board of Directors in its Meeting held on February 10, 2021 has fixed the number of elected directors at twelve (12) to be elected in AGM for the period of next three years commencing from March 27, 2021 in accordance with the provisions of Section 159(1) read with Section 161 of the Act.

11. Any person who seeks to contest the election to the office of a Director, whether he is retiring director or otherwise, shall file the following documents with the Company Secretary at the Registered Office of the Bank located at MCB Building, 15-Main Gulberg, Jail Road, Lahore, not later than fourteen days before the date of AGM:

    i. His/her complete Folio Number/CD Account Number including Participant ID etc.;
    ii. Notice of his/her intention to offer him/herself for the election of directors in terms of Section 159(3) of the Act together with the consent to act as a director of the Bank in Form 28 prescribed under the Act;
    iii. A detailed profile along with office address as required under SECP's SRO 1196 (I)/2019, dated October 03, 2019;
    iv. Declaration under Clause 3 of the Listed Companies (Code of Corporate Governance) Regulations, 2019;
    v. Declaration that he/she is not ineligible to become a director of MCB Bank in terms of Section 153 of the Act;
    vi. A director must be holding 500 shares of MCB at the time of filing of his/her consent to act as director in terms of Articles of Association of the Bank; and
    vii. Subject to the requirements of BPRD Circular No. 04 of 2007, dated April 23, 2007 regarding Fit & Proper Test ("FPT") and the Prudential Regulations (G-1) of the State Bank of Pakistan ("SBP"), the following documents, duly filled in and signed, will also be required:

        - Proforma-FPT ("Annexure-I");
        - Affidavit on Non-Judicial Stamp Paper ("Annexure-II");
        - Answered Questionnaire for assessing FPT;
        - Declaration that he is not ineligible to become a director under any provisions of the Banking Companies Ordinance, 1962 ("BCO"), Prudential Regulations or any directives of the SBP;
        - Declaration of Fidelity and Secrecy under Section 33 of the BCO;
        - Attested copy of valid CNIC/NICOP/Passport, recent Photographs;
        - Copies of all academic & professional degrees / certificates;
        - Copies of employment certificates received from previous employers; and
        - Detailed CV having date-wise employment history, if any.

    b) In terms of the criteria prescribed by the SBP, association of the following person as director is undesirable and against public interest:

        - A person who is / has been associated with any illegal activity, especially relating to banking business;
        - A person who is in his individual capacity or a proprietary concern of any partnership firm or any company (of which he has been a proprietor, partner, director or shareholder), has been in default of payment of dues owed to any financial institution, Government duties and / or in default of payment of any taxes; and
        - Further, a person is not permitted to be a director of more than one Bank/DFI.

    c) Independent director(s) will be elected through the process of election of directors in terms of Section 159 of the Act; however, they shall meet the criteria laid down in BPRD Circular No. 15 of 2016, dated December 28, 2016 and the Section 166 of the Act; accordingly, the following additional documents are to be submitted by the candidates intended to act as an independent director:

        - Declaration of Independence under BPRD Circular No. 15 of 2016, dated December 28, 2016 and the Section 166 of the Act; and
        - Declaration under Clause 6(3) of the Listed Companies (Code of Corporate Governance) Regulations, 2019 that he/she qualified the criteria of eligibility and independence stipulated under the Companies Act, 2017 and rules and regulations framed thereunder.

12. The Members will be allowed to exercise their voting right through postal ballot, that is voting by post, in case the number of candidates is more than the number of directors to be elected.

<u>Coronavirus ("COVID-19") Contingency Planning for AGM</u>

As per the requirements of the SECP Circular No. EMD/MISC/82/2012-77, dated February 15, 2021 the Bank is providing video Link facility for participation in the AGM. This facility is in addition to holding physical AGM at designated venue. Keeping in view the COVID-19 related Standard Operating Procedures ("SOPs") issued by the Provisional and/or the Federal Government, the Members are encouraged to participate in the meeting through Video Link. The Members or their proxies who wish to attend the AGM through Video-link are required to register themselves by providing the following information along with valid copy of the CNIC / Passport (both sides)/attested copy of board resolution/power of attorney as applicable through email at: cat@mcb.com.pk, (Tel: +92 42 3604 1621, +92 21 3264 1091) of the Bank by close of business on March 25, 2021.

Name of Shareholder	Folio/CDC Account Number	Number of Shares Held	Valid CNIC/NTN/ Passport Number	Mobile Number and Email Address

The Members or their proxies who are registered after necessary verification shall be provided a Video Link facility by the Bank on their email addresses provided to the Bank. The Login facility shall remain open from the start of the AGM till its proceedings are concluded.

The Members can also provide their comments/suggestions for the proposed agenda items of the AGM at email, i.e., cat@mcb.com.pk.

Agenda Item No. 5
<u>Statement under Section 166(3) of the Companies Act, 2017 in respect of Independent Director(s)</u>
Independent Directors will be elected through the process of election of directors in terms of Section 159 of the Companies Act, 2017 (the "Act") and they shall meet the criteria laid down under BPRD Circular No. 15 of 2016, dated December 28, 2016 issued by the SBP as well as comply with the requirements of Section 166(2) of the Act.

Section 166(3) of the Act requires that a statement of material facts is required to be annexed to the notice of the general meeting called for the purpose of election of directors which shall indicate the justification for choosing independent directors. Accordingly, it will be ensured that the independent directors to be elected must meet the criteria of independence laid down under section 166 of the Act and the Companies (Manner and Selection of Independent Directors) Regulations, 2018 and his/her name is included in the data bank of independent directors maintained by the Pakistan Institute of Corporate Governance ("PICG") duly authorized by the Securities and Exchange Commission of Pakistan. Further, their selection will be made due to their respective competencies, skill, knowledge and experience.

The present Directors of the Company have no interest in the above said business except being eligible for re-election as director of the Company.

<u>STATEMENT UNDER SECTION 134(3) OF THE COMPANIES ACT, 2017</u>
<u>IN RESPECT OF SPECIAL BUSINESS</u>

This statement sets out the material facts under Section 134(3) of the Companies Act, 2017 pertaining to the Special Business to be transacted at AGM of the Bank scheduled to be held on March 27, 2021 along with the information as required under SRO 423(I)/2018, dated April 03, 2018 issued by the SECP and applicable to the Bank:

Agenda Item No. 6
<u>Winding Up of a Subsidiary of MCB Bank Limited:</u>

M/s Financial & Management Services (Pvt.) Limited ("FMSL" or the "Company") is a subsidiary company of MCB Bank Limited ("MCB" or the "Bank"). MCB holds 95.90% of total issued paid up share capital of FMSL, transferred from NIB Bank Limited ("Ex-NIB") by virtue of amalgamation with and into MCB on July 07, 2017. FMSL has no assets and liabilities and is in dormant status with no accounting transaction(s) since March 25, 2009. MCB, being a holding company is bearing regulatory expenses including audit fee since then; therefore, the Board of Directors of the Bank has approved the winding up of the Company. As per the requirements of Section 183(3)(b) of the Companies Act, 2017 (the "Act"), the Bank is seeking approval of the members in forthcoming Annual General Meeting of the Bank scheduled to be held on March 27, 2021.

"RESOLVED THAT pursuant to the provisions of the Section 183 (3)(b) of the Companies Act, 2017 ('Act') and other applicable provisions of the laws, rules & regulations, for the time being in force, the approval of the Members of MCB Bank Limited (the 'Bank') be and is hereby accorded to voluntary winding up of M/s Financial & Management Services (Pvt.) Limited ('FMSL'), a subsidiary of the Bank, subject to compliance with all applicable legal requirements by the subsidiary."

"FURTHER RESOLVED THAT the Company Secretary and/or Chief Financial Officer be and are hereby jointly/severally empowered and authorized to do all acts, matters, deeds and things, take any or all necessary actions including signing and execution of agreement(s) and to complete all the regulatory, legal and other formalities including filing of application(s) to the SECP or any other authority as may be necessary or incidental or expedient for the purpose of implementing the aforesaid resolutions."

The Directors of the Bank have no personal interest, directly or indirectly, in the above special business, save to the extent of their respective shareholding in the Bank.

Attention of Shareholders is drawn towards Circulars/Notifications:

The following Circulars/Notifications require special attention of Members of the Bank:

1. Requirement of Valid CNIC and IBAN:

   As per the requirements of the Companies (Distribution of Dividends) Regulations, 2017 issued by the Securities and Exchange Commission of Pakistan ("**SECP**") and SRO 275 (I)/2016, dated March 31, 2016 issued by the SECP, the payment of cash dividend shall only be made to the Shareholders who have provided copies of their valid CNIC/ NICOP/ Passport (in the case of Individuals) and NTN (in the case of Corporate Entities) and valid details of designated International Bank Account Number ("**IBAN**"). Therefore, in order to comply with the above regulatory requirements, the Bank has withheld and shall be withholding the payment of cash dividend to those shareholders who have not yet provided copies of their valid CNIC/NICOP/NTN/Passport and details of valid IBAN.

2. Requirement of FBR's Approval or Valid Tax Exemption Certificate for Claim of Exemption U/S 150 of the Income Tax Ordinance, 2001:

   The Honorable Lahore High Court, Lahore, in its decision has advised that the Mutual Funds as approved by the Federal Board of Revenue ("**FBR**"), would not be required to provide exemption certificate under Section 159 of the Income Tax Ordinance, 2001 ("**Ordinance**") to claim tax immunity as per clause (47B) of Part-IV of the Second Schedule to the Ordinance. It is, therefore, requested to provide either approval certificate from FBR or in the absence of the said certificate, valid exemption certificate under Section 159 (1) of the Ordinance issued by the concerned Commissioner of the Income Tax, Inland Revenue, FBR. In case of non-availability of approval / exemption certificate(s), the deduction of advance tax on dividend shall be made as per the relevant provisions of the Ordinance.

3. Deduction of Withholding Tax on the Amount of Dividend:

   As per the provisions of the Section 150 of the Income Tax Ordinance, 2001, the details of withholding tax on dividend are as follows:

   - For Filers of Income Tax Returns: 15%
   - For Non-Filers of Income Tax Returns: 30%

   In case of joint-shareholders, tax is to be deducted as per ratio of their respective share in the ownership.

   Federal Board of Revenue ("**FBR**") has provided the Active Tax-Payer List ("**ATL**"), for identification on the basis of NTN/CNIC number; hence, in case of non-availability of valid NTN/CNIC number with MCB's Share Registrar and Transfer Agent, it would not be possible to identify the same in ATL and the Bank will be constrained to treat the Shareholder as 'Non-Filer' to qualify for tax at higher rate of 30%. Therefore, shareholders who have not yet provided such information are requested to ensure that their valid NTN/CNIC number is available with the Share Registrar and Transfer Agent of MCB. In case of shares held as book-entry securities, the said information would be required to be provided to CDS, through CDS Participants.

4. **Deduction of Withholding Tax on Joint Account Holder(s):**

The Joint Account Holders whose shareholding details as to Principal Shareholder have not yet been determined for deduction of withholding tax on the upcoming dividend of the Bank, are requested to please furnish to the Share Registrar and Transfer Agent of the Bank; the shareholding details of yourself as Principal Shareholder and your Joint Holder(s) in the following manner, enabling MCB to compute withholding tax of each shareholder accordingly:

CDC Account No./ Folio No.	Name of Principal Shareholder/Joint Holders	Shareholding Proportion	CNIC No. (copy attached)	Signature

Kindly note that in case of non-receipt of the information each Account Holder will be assumed to hold equal proportion of shares and the deduction will be made accordingly.

5. **Circulation of Annual Audited Financial Statements and Notice of AGM to Members:**

SECP through its Notification No. SRO 787 (I)/2014, dated September 08, 2014, has allowed companies to circulate Annual Audited Financial Statements along with Notice of Annual General Meeting to its members through email. Further, as per the requirements of the SECP Notification No. SRO 470 (I)/2016, dated May 31, 2016, the Bank is circulating its Annual Accounts to shareholders through electronic medium, i.e., CD/DVD at their registered addresses instead of transmitting the said annual accounts in hard copies. However, in case a shareholder is required hard copy of the Annual Accounts, the same can be obtained, free of cost, within one week of the request. In this regard, a Standard Request Form has been placed on website of the Bank, namely, www.mcb.com.pk, for shareholders to communicate the need of hard copies of the Annual Accounts instead of sending the same through CD/DVD. A shareholder may also prefer to receive hard copies for all future Annual Accounts.

6. **Unclaimed Dividends and Shares Certificates:**

The shareholders who have not yet claimed their cash dividends, right and bonus shares which are kept with the Share Registrar of the Bank, namely, M/s THK Associates (Pvt.) Limited, as undelivered/unclaimed dividends and shares certificates or which are in their custody are requested that they should either en-cash their dividend warrants already available with them, or make a claim for cash dividends, right and bonus shares kept by MCB's Share Registrar and Transfer Agent.

7. **Postal Ballot / E-voting:**

Pursuant to the provisions of the Companies (Postal Ballot) Regulations, 2018 (the "Regulations"), for any other agenda item subject to the requirements of Section 143 and 144 of the Act, Members present in person, through video-link or by proxy, and having not less than one-tenth of the total voting power can also demand a poll and exercise their right of vote through postal ballot, that is voting by Ballot Paper or e-voting, in accordance with requirements and procedure contained in the Regulations.

8. **Deposit of Physical Shares into CDC Account:**

The Shareholders having physical shareholding may open CDC sub-account with any of the brokers or Investor Account directly with CDC to place their physical shares into book-entry form, this will facilitate them in many ways including safe custody and sale of shares, any time they want, as the trading of physical shares is not permitted as per existing regulations of the Stock Exchange. Further, Section 72 of the Act states that after the commencement of the Act from a date notified by SECP, a company having share capital, shall have shares in book-entry form only. Every existing company shall be required to replace its physical shares with book-entry form in a manner as may be specified and from the date notified by SECP, within a period not exceeding four years from the commencement of the Act.

9. **Merger of Different Folios into One Folio:**

As per record, some of the shareholders are maintaining more than one folio(s) under the same particulars. Carrying more than one folio may be a hassle for the shareholders to reconcile and receive different benefits in the shape of dividends/ bonus. In order to provide better services and convenience, such shareholders are requested to send

requests to the MCB's Share Registrar and Transfer Agent to merge their folios into one folio. While sending the copy of NTN/CNIC/NICOP number, shareholders are requested to quote their respective Folio/CDC Account Numbers for identification purpose.

10. **Zakat Declaration (CZ-50):**

    Zakat will be deducted from the dividends at source at the rate of 2.5 % of the paid-up value of the share, i.e., PKR 10/- each and will be deposited within the prescribed period with the relevant authority. In case of claiming exemption, please submit your Zakat Declarations under Zakat and Ushr Ordinance, 1980 and Rule 4 of Zakat (Deduction and Refund) Rules, 1981, "CZ-50 Form" with the Share Registrar and Transfer Agent of the Bank at the below mentioned address and the respective Folio and CDC Account details.

11. **Placement of Notice of AGM on the Website:**

    Notice of 73rd AGM of the Bank has been placed on the Website of the Bank, i.e., www.mcb.com.pk, in addition to its dispatch to the Shareholders through CD/DVDs, printed copies as well as publication in the newspapers.

While sending the copy of NTN/CNIC/NICOP number, shareholders are requested to quote their respective Folio/CDC Account Numbers for identification purpose.

M/s THK Associates (Pvt) Ltd.,
Share Registrar and Transfer Agent-MCB Bank Limited
Plot No. 32-C, Jami Commercial Street 2, D.H.A., Phase VII, Karachi-75500. Pakistan.
P.O. Box No. 8533, UAN: +92 (21) 111-000-322,
Fax: +92 (21) 35310191.
Email: sfc@thk.com.pk
Website: www.thk.com.pk

Glossary of Terms

Important terms and formulae used for calculation in Financial Statements are briefly described here;

Accrual Basis
Recognizing the effects of transactions and other events when they occur without waiting for receipt or payment of cash or its equivalent.

Basis point
One hundredth of a per cent i.e. 0.01 per cent. 100 basis points is 1 per cent. Used when quoting movements in interest rates or yields on securities.

Breakup Value per share
Represents the total worth (equity) of the business per share, calculated as shareholders' equity or Net Assets excluding the impact of revaluation on fixed assets, divided by the total number of share outstanding at year end.

BSD
Banking Surveillance Department of State Bank of Pakistan

BPRD
Banking Policy and Regulation Department of State Bank of Pakistan

CAGR
An abbreviation for Compound Annual Growth Rate.

Capital Adequacy Ratio (CAR)
The relationship between capital and risk weighted assets as defined in the framework developed by the State Bank of Pakistan.

Cash Reserve Ratio (CRR)
Cash Reserve Ratio is the amount of funds that the banks have to keep with SBP.

Cash Equivalents
Short-term highly liquid investments that are readily convertible to known amounts of cash and which are subject to an insignificant risk of changes in value.

Commitment to Extend Credit
Credit facilities approved but not yet utilized by the client as at the Balance Sheet date.

Cost to Income Ratio
The proportion of admin expenses to total income, represented as combination of net interest income and non interest income.

Credit Risk Spread
The credit spread is the yield spread between securities with the same coupon rate and maturity structure but with different associated credit risks, with the yield spread rising as the credit rating worsens. It is the premium over the benchmark or risk-free rate required by the market to take on a lower credit quality.

Discount rate
Discount is the rate at which SBP provides three-day Repo facility to banks, acting as the lender of last resort.

Dividend Payout Ratio
Dividends (cash dividend plus bonus shares) paid per share as a fraction of earnings per share (EPS).

Dividend Yield Ratio
Dividend per share divided by the market value of share.

Earnings Per Share
Profit after taxation divided by the weighted average number of ordinary shares in issue

Fixed Deposits
Deposits having fixed maturity dates and a rate of return.

Forced Sale Value (FSV)
Forced Sale Value means the value which fully reflects the possibility of price fluctuations and can currently be obtained by selling the mortgaged / pledged assets in a forced / distressed sale conditions.

Government Securities
Government Securities shall include such types of Pak. Rupee obligations of the Federal Government or a Provincial Government or of a Corporation wholly owned or controlled, directly or indirectly, by the Federal Government or a Provincial Government and guaranteed by the Federal Government as the Federal Government may, by notification in the Official Gazette, declare, to the extent determined from time to time, to be Government Securities.

Impairment allowances
A provision held on the balance sheet as a result of the raising of a charge against profit for the incurred loss inherent in the lending book. An impairment allowance may either be identified or unidentified and individual or collective.

Foreign Exchange Options (FX Options)
Contracts that give the buyer the right, but not the obligation, to buy or sell one currency against the other, at a predetermined price and on or before a predetermined date. The buyer of a call/ put FX option has the right to buy/sell a currency against another at a specified rate.

Forward Purchase Contract
Forward purchase contract is one in which the exporter enters into the forward booking contract to protect himself from the exchange rate fluctuation at the time of receiving payment.

Forward Sale Contract
In a forward sale contract the importer enters into a transaction to buy foreign currency from the Bank at the predetermined rate to protect himself from the exchange fluctuation at the date the payment.

Glossary of Terms

Historical Cost Convention
Recording transactions at the actual value received or paid.

IAS
International Accounting Standards

IFRS
International Financial Reporting Standards

IFRIC
International Financial Reporting Interpretation Committee

Interest Spread
Represents the difference between the average interest rate earned and the average interest rate paid on funds.

Loan losses and provisions
Amount set aside against identified and possible losses on loans, advances and other credit facilities as a result of their becoming party or wholly uncollectible.

Liquid Assets
The assets which are readily convertible into cash without recourse to a court of law and mean encashment / realizable value of government securities, bank deposits, certificates of deposit, shares of listed companies which are actively traded on the stock exchange, NIT Units, certificates of mutual funds, Certificates of Investment (COIs) issued by DFIs / NBFCs rated at least 'A' by a credit rating agency on the approved panel of State Bank of Pakistan, listed TFCs rated at least 'A' by a credit rating agency on the approved panel of State Bank of Pakistan and certificates of asset management companies for which there is a book maker quoting daily offer and bid rates and there is active secondary market trading. These assets with appropriate margins should be in possession of the banks / DFIs with perfected lien.

Market Capitalization
Number of ordinary shares in issue multiplied by the market value of share as at any cut-off date.

Materiality
The relative significance of a transaction or an event the omission or misstatement of which could influence the economic decisions of users of financial statements.

Net Interest Income (NII)
Net interest income is the difference between the interest earned on assets and interest expensed on liabilities.

Non-Performing Loan
A non-performing loan is a loan that is in default or close to being in default. Loans become non-performing in accordance with provision of prudential regulations issued by SBP.

NPLs to Gross Advances/Loans
Represents the infected portfolio of the bank and is calculated by dividing the total non-performing loans by gross advances.

Non Performing Loan-Substandard Category
Where markup/interest or principal is overdue by 90 days or more from the due date.

Non Performing Loan-Doubtful Category
Where markup/interest or principal is overdue by 180 days or more from the due date.

Non Performing Loan-Loss Category
Where mark-up/interest or principal is overdue by one year or more from the due date and Trade Bill (Import/ Export or Inland Bills) are not paid/adjusted within 180 days of the due date.

Off Balance Sheet Transactions
Transactions that are not recognized as assets or liabilities in the statement of financial position but which give rise to contingencies and commitments.

Price Earnings Ratio (P/E Ratio)
Market price of a share divided by earnings per share.
Repo / Reverse Repo A repurchase agreement, or repo, is a short term funding agreements which allow a borrower to sell a financial asset, such as ABS or government bonds as collateral for cash. As part of the agreement the borrower agrees to repurchase the security at some later date, usually less than 30 days, repaying the proceeds of the loan. For the party on the other end of the transaction (buying the security and agreeing to sell in the future) it is a reverse repurchase agreement or reverse repo.

Return on Equity (ROE)
Represents the ratio of the current year's profit available for distribution to the weighted average shareholders equity over the period under review, calculated by dividing the net profit (profit after tax) to the average equity (before surplus) for the period.

Return on Assets (ROA)
Indicator of profitability of the business relative to the value of its assets, calculated by dividing the net profit (profit after tax) to the average total assets for the period.

Statutory Reserve Funds
A capital reserve created as per the provisions of the section 21 of Banking Companies Ordinance, 1962.

Small Enterprise
A Small Enterprise (SE) is a business entity which meets both the following parameters:

Number of Employees	Annual Sales Turnover
*Up to 50	Up to Rs. 150 million

*including contract employees.

Glossary of Terms

Basel III
Basel III (or the Third Basel Accord) is a global, voluntary regulatory standard on bank capital adequacy, stress testing and market liquidity risk.

Strategic Investment
Strategic Investment is an investment which a bank / DFI makes with the intention to hold it for a period of minimum 5 years.

The following must be noted further in respect of strategic investment:

- The bank should mark strategic investment as such at the time of investment
- If there are a series of purchases of stocks of a company, the minimum retention period of 5 years shall be counted from the date of the last purchase.

SRO
Statutory Regulatory Order

KIBOR – (Karachi Interbank Offered Rate)
KIBOR is the interbank lending rate between banks in Pakistan and is used as a benchmark for lending.

LIBOR (London Interbank Offered Rate)
An interest rate at which banks can borrow funds, in marketable size, from other banks in the London interbank market. The LIBOR is fixed on a daily basis by the British Bankers' Association.

VaR
Value at Risk is an estimate of the potential loss which might arise from market movements under normal market conditions, if the current positions were to be held unchanged for one business day, measured to a confidence level of 97.5 per cent.

Weighted Average Cost of Deposits
Percentage of the total interest expense on average deposit of the bank for the period.

Form of Proxy
73rd Annual General Meeting

I/We ____ of ______________ being a member of **MCB Bank Limited**, holder of ____________________________ Ordinary Share(s) as per Folio No. and/or CDC Account No. ________________________ do hereby appoint Mr./Mrs./Miss ________________________ having Folio No. /CDC Account No. ____________________ or failing him/her, Mr./Mrs./Miss _________________________ having Folio No. /CDC Account No. ___________ as my/our proxy to attend, speak and vote for me/us on my/our behalf at the **73rd Annual General Meeting ("AGM")** of the Bank to be held on **Saturday,** the **27th** day of **March, 2021 at 11:00 AM (PST)** at Grand Ball Room-D, The Nishat Hotel, Emporium Mall, Near Expo Center, Abdul Haq Road, Johar Town, Lahore, and at any adjournment thereof in the same manner as I/we/myself/ ourselves would vote if personally present at such meeting.

As witness my / our hand/Seal this __________________________ day of _____________________________ 2021.

Signed by ____

Folio No.	CDC Account No.	
	Participant I.D.	Account No.

Signature on
Five-Rupees
Revenue Stamp

The signature should agree with the specimen registered with the Bank.

Witnesses:

1. Name : ______________________________
Address : ______________________________
CNIC No. : ______________________________
Signature : ______________________________

2. Name : ______________________________
Address : ______________________________
CNIC No. : ______________________________
Signature : ______________________________

Note:

1. A member eligible to attend, speak and vote at the AGM may appoint another member as his/her proxy who shall have such rights as narrated in Section 137 of the Companies Act, 2017.
2. This Proxy Form, duly completed and signed, must be deposited in the office of M/s THK Associates (Pvt) Limited, the Share Registrar and Transfer Agent of the Bank, situated at Plot No. 32-C, Jami Commercial Street 2, D.H.A., Phase VII, Karachi-75400, not less than 48 hours before the time of holding the meeting.
3. If a member appoints more than one proxy and more than one instrument of proxies are deposited by a member with the Share Registrar and Transfer Agent of the Bank, all such instruments of proxy shall be rendered invalid.
4. **For CDC Account Holders / Corporate Entities**
    - Attested copies of CNIC/ NICOP or the passport of the beneficial owners and the proxy shall be provided with the Proxy Form.
    - The proxy shall produce his/her original CNIC/ NICOP or passport at the time of the meeting.
    - In case of a corporate entity, the Board of Directors' resolution / power of attorney with specimen signature shall be submitted along with Proxy Form to the Share Registrar and Transfer Agent of the Bank.

MCB
Bank for Life

پراکسی فارم

73واں سالانہ عام اجلاس

میں/ہم ____________________

ساکن ____________ بحیثیت ممبر ایم سی بی بینک لمیٹڈ اور ____________ عام حصص کا مالک ____________ فولیو/سی ڈی سی اکاؤنٹ نمبر کے تحت

مسمی/ مسماۃ ____________ کو جس کا فولیو/سی ڈی سی اکاؤنٹ نمبر ____________ ہے یا اس کی عدم دستیابی کی صورت میں

مسمی/ مسماۃ ____________ جس کا فولیو/سی ڈی سی اکاؤنٹ نمبر ____________ ہے بطور پراکسی مقرر کرتا ہوں/کرتی ہوں/کرتے ہیں

تاکہ وہ میری/ہماری غیر موجودگی میں میری/ہماری طرف سے بینک کے **73** ویں سالانہ عام اجلاس جو بروز ہفتہ، مورخہ **27** مارچ **2021**، بوقت **11:00** بجے صبح، بمقام گرینڈ بال روم۔ ڈی، دی نشاط ہوٹل، ایمپوریم مال، نزد ایکسپو سینٹر، عبدالحق روڈ، جوہر ٹاؤن، لاہور منعقد ہو رہا ہے اس میں اور اس کے کسی ملتوی شدہ اجلاس میں، اس طرح سے شرکت کرے، بات کرے، اور ووٹ ڈالے کہ جس طرح میں/ہم خود اس اجلاس میں موجود ہوں۔

میرے/ہمارے دستخط، بتاریخ ____________ 2021ء

دستخط ____________

فولیو نمبر	سی ڈی سی اکاؤنٹ	
	پارٹیسپنٹ ID	اکاؤنٹ نمبر

پانچ روپیہ کے ریونیو اسٹامپ پر دستخط

دستخط بینک میں رجسٹرڈ نمونے سے مطابقت رکھتے ہوں

گواہان:

1) نام: ____________
پتہ: ____________
شناختی کارڈ نمبر: ____________
دستخط: ____________

2) نام: ____________
پتہ: ____________
شناختی کارڈ نمبر: ____________
دستخط: ____________

نوٹ:

1۔ اے جی ایم میں شرکت کرنے، بولنے اور ووٹ دینے کا اہل رکن کسی دوسرے رکن کو اپنا/اپنی پراکسی مقرر کر سکتا ہے جس کو وہ تمام حقوق حاصل ہونگے جیسا کہ کمپنیز ایکٹ 2017ء کے سیکشن 137 میں بیان کئے گئے ہیں۔

2۔ مکمل پُر کردہ اور دستخط شدہ پراکسی فارم بینک کے شیئر رجسٹرار اور ٹرانسفر ایجنٹ میسرز ٹی ایچ کے ایسوسی ایٹس (پرائیویٹ) لمیٹڈ، بمقام پلاٹ نمبر 32-C، جامی کمرشل اسٹریٹ 2، ڈی ایچ اے، فیز VII، کراچی-75400 کے دفتر میں اجلاس کے انعقاد سے 48 گھنٹے پہلے جمع کروانا ہوگا۔

3۔ اگر ایک رکن ایک سے زیادہ پراکسی مقرر کرتا ہے اور بینک کے شیئر رجسٹرار اور شیئر ٹرانسفر ایجنٹ کے پاس رکن کی طرف سے پراکسی فارم کی ایک سے زائد دستاویزات جمع کرائی جائیں تو پراکسی کی ایسی تمام دستاویزات کالعدم تصور ہوں گی۔

4۔ سی ڈی سی اکاؤنٹ رکھنے والوں / کارپوریٹ اداروں کے لیے:

- مستفید مالکان اور پراکسی کے سی این آئی سی/این آئی سی او پی یا پاسپورٹ کی تصدیق شدہ کاپیاں پراکسی فارم کے ساتھ مہیا کی جائیں گی۔
- اجلاس کے موقع پر پراکسی اپنا اصل سی این آئی سی/این آئی سی او پی یا پاسپورٹ مہیا کرے گا۔
- کارپوریٹ ادارہ ہونے کی صورت میں، بورڈ آف ڈائریکٹرز کی قرارداد/ مختار نامہ نمونے کے مطابق دستخط کے ساتھ بینک کے رجسٹرار اور ٹرانسفر ایجنٹ کے پاس پراکسی فارم کے ساتھ جمع کروانا ہوگا۔

Investors' Awareness

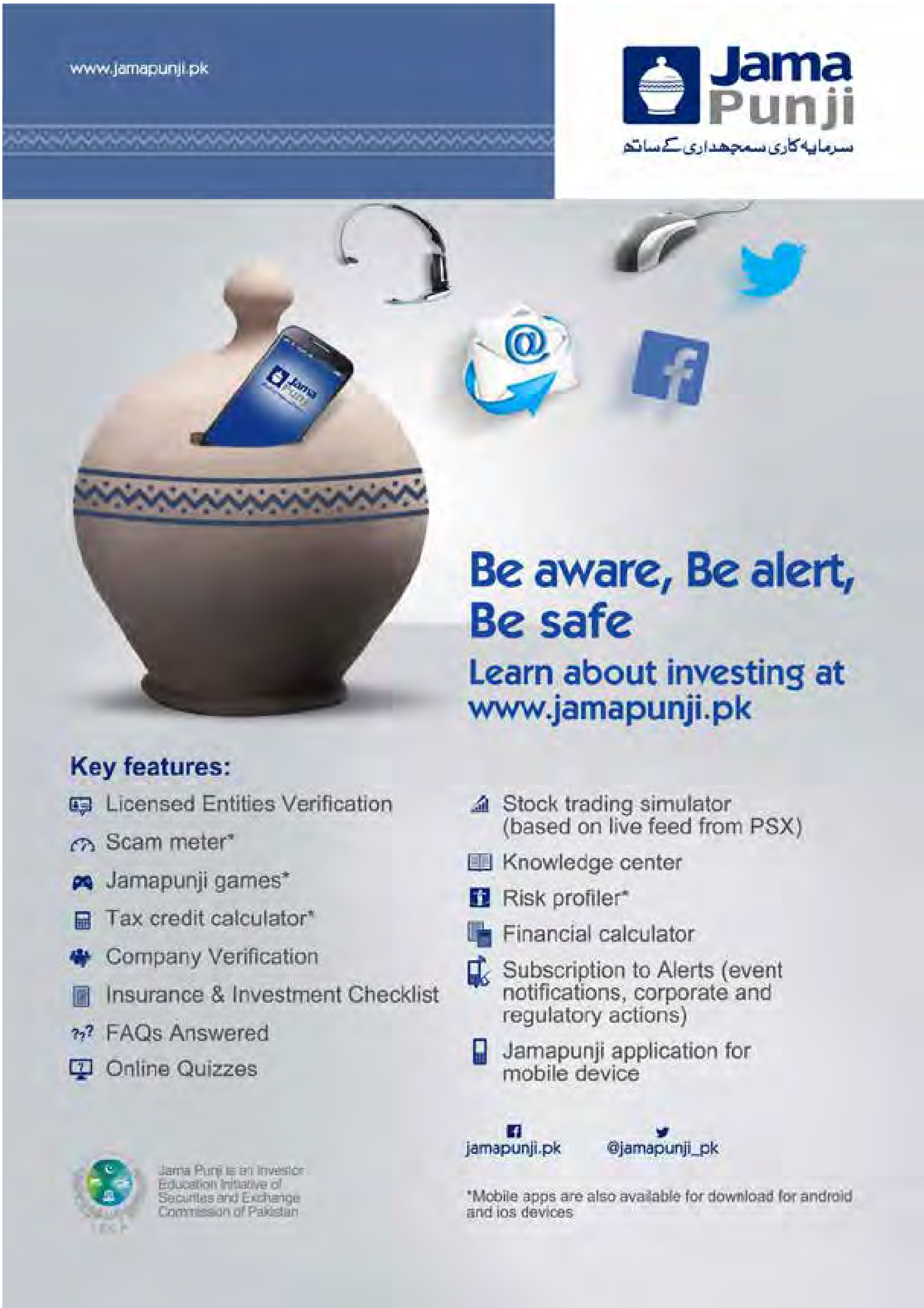

MCB

Bank for Life

MCB

Bank for Life

Registered Office: MCB House, 15 Main Gulberg, Jail Road, Lahore, Pakistan
U: +92 42 111 000 622 T: +92 42 36041998-9 E: info@mcb.com.pk www.mcb.com.pk

VANTAGE
